# Integration of Viral Genome to Human Genomic DNA in Nails of Patients with Chronic Hepatitis B Virus Infection

**DOI:** 10.31662/jmaj.2023-0082

**Published:** 2023-09-29

**Authors:** Haruki Komatsu, Ayano Inui, Hiroki Hoshino, Shuichiro Umetsu, Tomoo Fujisawa

**Affiliations:** 1 Department of Pediatrics, Toho University, Sakura Medical Center, Chiba, Japan; 2 Komatsu Children’s Clinic, Chiba, Japan; 3 Department of Pediatric Hepatology and Gastroenterology, Eastern Yokohama Hospital, Kanagawa, Japan

**Keywords:** hepatitis B virus, human herpes virus 7, host, chromosome, next-generation sequencing, capture, PCR

## Abstract

**Introduction:**

Hepatitis B virus (HBV) DNA and cytomegalovirus (CMV) DNA can be detected in patient genomes. However, it remains unknown whether viral DNA can be integrated into host genomic DNA and detected in fingernails.

**Methods:**

Nails from patients with chronic HBV infection were investigated. A total of 60 patients (male/female = 20/40, age range from 2 years to 59 years, median 15 years) were included in this study. The viral DNA levels of herpes simplex virus 1 (HSV-1), herpes simplex virus 2 (HSV-2), varicella-zoster virus (VZV), Epstein‒Barr virus (EBV), cytomegalovirus (CMV), human herpes virus 6 (HHV-6), human herpes virus 7 (HHV-7), and HBV in nails were measured with real-time PCR. Viral DNA integration into host genomic DNA was analyzed by capture-based next-generation sequencing (NGS). Moreover, virus/host chimeric sequences, which were detected by capture-based NGS, were confirmed by Sanger sequencing.

**Results:**

Of the 60 patients, 37 (62%) were positive for nail HBV DNA. All 60 patients were negative for nail HSV-1, HSV-2, VZV, CMV, EBV, or HHV-6 DNA. However, three patients were positive for nail HHV-7 DNA. All three nail HHV-7-positive patients were also positive for nail HBV DNA. The three nail samples that were positive for both HBV and HHV-7 DNA were used for viral integration analysis by capture-based NGS. One of the three nail samples showed HBV/host chimeric sequences. In addition, all three nail samples showed HHV-7/host chimeric sequences. However, these viral integration breakpoints were not confirmed by Sanger sequencing.

**Conclusions:**

Viral integrations were detected in nails by capture-based NGS. However, Sanger sequencing did not confirm any virus/host chimeric sequences. This study could not show reliable evidence of viral integration in nails.

## Introduction

The integration of hepatitis B virus (HBV) DNA into host genomic DNA occurs in the liver tissue of patients with chronic HBV infection ^(1), (2), (3), (4), (5)^. Moreover, the integration of HBV DNA into host genomic DNA is observed in peripheral blood cells ^(6), (7)^. Recent studies have shown that HBV DNA can be detected in the nails and hair of patients with acute and chronic HBV infection ^(8), (9), (10)^. In addition to HBV, cytomegalovirus (CMV) DNA was detected in the nails of a child with congenital CMV infection ^(11)^. These findings suggest that viral DNA might be integrated into host genomic DNA in nails under certain circumstances. However, it remains unclear whether viral DNA integrated into host genomic DNA can be detected in nails. Capture-based next-generation sequencing (NGS), wherein viral DNA is targeted using viral-specific probes for the construction of a library, is a highly sensitive and effective method for the detection of viral DNA integration sites ^(12), (13), (14)^.

In this study, we investigated the presence of HBV DNA and human herpes virus DNA in the nails of patients with chronic HBV infection. Moreover, capture-based NGS was used to evaluate whether viral DNA is integrated into host genomic DNA and can be detected in nails.

## Materials and Methods

### Patients and materials

Patients with chronic HBV infection were included. Patients who used fingernail polish on their fingernails were excluded. Samples of the patients’ blood and fingernails were collected at the outpatient department. Fingernails were obtained using a stainless steel clipper. The ethics committee of Toho University Sakura Medical Center (no. 2015-073) and Eastern Yokohama Hospital (no. 2015010) approved the study protocols. This study was conducted in accordance with the ethical guidelines of the 1975 Declaration of Helsinki. Written informed consent was obtained from all patients or legal guardians prior to sample collection.

A total of 60 patients with chronic HBV infection (male/female = 20/40, age range from 2 years to 59 years, median 15 years) were enrolled in this study. Of these patients, 47 were positive for hepatitis B e-antigen (HBeAg) and 13 were positive for anti-HBe antibodies. The levels of serum HBV DNA ranged from <2.1 Log copies/mL to >9.0 Log copies/mL (median 8.8 Log copies/mL).

### DNA extraction and real-time PCR

Extraction of peripheral blood cell DNA from 2 mL of whole blood was performed with the Gentra Puregene Blood Kit (Qiagen, Hilden, Germany). Extraction of DNA from approximately 1.0 mg of fingernails was performed with the DNA Extractor FM Kit (Wako Pure Chemical Industries, Osaka, Japan). The primers for real-time PCR were based on reported methods. The viral DNA of herpes simplex virus 1 (HSV-1) ^(15)^, herpes simplex virus 2 (HSV-2) ^(15)^, varicella-zoster virus (VZV) ^(16)^, Epstein‒Barr virus (EBV) ^(17)^, cytomegalovirus (CMV) ^(17)^, human herpes virus 6 (HHV-6) ^(17)^, human herpes virus 7 (HHV-7) ^(18)^, and HBV ^(19)^ was detected by real-time PCR. This assay was standardized using recombinant plasmid controls.

### Targeted enrichment of NGS

#### DNA probe

We designed an individual set of biotinylated DNA probes for targeted enrichment of the HBV and HHV-7 genetic materials by hybrid capture. The DNA probes targeting the entirety of HBV and HHV-7 were 120 bp in length with a tiling of 60 bp (Supplementary Fig. 1). A total of 53 (Supplementary Table 1) and 2,408 (Supplementary Table 2) xGen Lockdown Probes (Integrated DNA Technologies) were made based on the HBV (GenBank accession number: AB033550.1) and HHV-7 (GenBank accession number: U43400.1) sequences, respectively.

#### DNA extraction and DNA library construction

DNA extraction from fingernails, peripheral blood lymphocytes, and liver tissue was performed using the DNA extraction DNA Extractor FM Kit (WAKO), Puregene Blood Core Kit (QIAGEN), and QIAamp DNA Mini (QIAGEN), respectively. DNA samples were quantified using a Qubit fluorometer (Life Technologies, Carlsbad, CA, USA) and a NanoDrop system (Thermo Fisher, Waltham, MA). Moreover, quality control of each sample was performed using an Agilent 2200 TapeStation System (Agilent Technologies, Santa Clara, CA, USA). First, 29-845 ng of genomic DNA was fragmented to an average length of approximately 150 bp using a Covaris S220 sonication device. The fragments were purified, end-repaired, A-tailed, and ligated to adaptors. The libraries were constructed by the KAPA HyperPlus Library Preparation Kit (Kapa Biosystems, Wilmington, MA, USA) or NEBNext Ultra II DNA Library Prep Kit for Illumina (New England BioLabs, Ipswich, MA, USA). Quality control of the libraries was performed using an Agilent 2200 TapeStation System (Agilent Technologies, Santa Clara, CA, USA).

#### Viral DNA enrichment

Enrichment with hybridization with virus-specific probes was performed using an xGen Hybridization and Wash Kit (Integrated DNA Technologies). First, 54-500 ng of library DNA was mixed with human Cot-1 DNA and xGen Universal Blockers (Integrated DNA Technologies) and dried. The dried DNA was resuspended in xGen Lockdown buffer. After incubation at 95°C for 10 min, virus-specific probes were added to the dissolved DNA and hybridized at 65°C for 4 hr. Streptavidin-coated magnetic beads were added to the hybridized mixture, and further incubation was performed at 65°C for 45 min. After the washing step, the captured DNA was amplified by PCR (18 cycles) with xGen Library Amplification Primer Mix (Integrated DNA Technologies). The amplicons were purified, and pair-end 100-bp (Illumina HiSeq 2500) or 150-bp (Illumina NovaSeq 6000) read-length sequencing was performed according to the manufacturer’s instructions (Illumina, Sa Diego, CA, USA).

### Analysis of the HBV and HHV-7 integration breakpoints

After obtaining FASTQ files from Illumina HiSeq or Illumina NovaSeq systems, we performed an adaptor-trimming step and removed low-quality sequencing reads using Trimmomatic ^(20)^. The sequencing quality score (Q score) of each base read was calculated. After trimming and removing low-quality reads, sequencing reads covering 70 bases or more were used for mapping. The integration analysis was based on the read alignment of the human immunodeficiency virus integration project ^(21), (22)^. The cleaned sequencing reads were mapped onto HBV (GenBank: AB033550.1), HHV-7 (GenBank: U43400.1), and the human reference (Genome Reference Consortium Human Build 38) using the BWA-MEM algorithm ^(23)^. PCR duplicates were removed by the Picard tool. For the viral-human chimeric sequencing reads, the viral portion would be aligned as mapped sequences (at least 32 bases), leaving the unaligned human sequences as overhanging (Supplementary Fig. 2). These overhangs are soft-clipped, and the sequences are retained in the alignment file. Integration reads were extracted from all soft-clipped and mapped sequences. Finally, the viral breakpoints were determined using FLAG, CIGAR, and the soft-clipped information (algorithm of integration data analysis: Supplementary Fig. 3).

### Confirmation by Sanger sequencing

To confirm the integration site, Sanger sequencing was performed. PCR primers were designed on the basis of the integration reads, which were suggested as virus/host chimeric sequences by Mega BLAST, a basic local alignment search tool of National Library of Medicine. All viral DNA samples were amplified by nested PCR using two primer pairs with sequences corresponding to regions of the human and viral genomes, which encompassed an integration site. PCR products were used for direct sequencing. When direct sequencing was not successful, PCR cloning was performed using a TOPO TA cloning kit for sequencing (Invitrogen).

### Statistical analysis

Categorical variables were compared between groups, using the Yates corrected χ2 test or the Fisher exact test.

## Results

### HBV DNA and herpes virus DNA detection in nails

Beta-actin DNA was detected in all 60 nail samples by real-time PCR. Table 1 shows the frequency of nail HBV DNA-positive patients. Of the 60 patients, 37 (62%) (HBeAg positive, n = 34; anti-HBe positive, n = 3) were positive for nail HBV DNA. All 60 patients were negative for nail HSV-1 DNA, HSV-2 DNA, VZV DNA, CMV DNA, EBV DNA, or HHV-6 DNA. Of the 60 patients, 3 (5%) were positive for nail HHV-7 DNA. The detection rate of HBV DNA is significantly higher than that of other viral DNA (P < 0.001). However, there is no statistical significance in the detection rate of viral DNA among HSV-1, HSV-2, VSV, EBV, CMV, HHV-6, and HHV-7. In addition, all three of the nail HHV-7-positive patients were also positive for nail HBV DNA. HBV DNA and HHV-7 DNA were confirmed by virus-specific nested PCR and Sanger sequencing ^(24), (25)^. Table 2 shows the characteristics of the three patients who were positive for both nail HBV DNA and nail HHV-7 DNA.

**Table 1.** Detection of Viral DNA in Nails by Real-Time PCR.

Virus	Number of patients (%)
Hepatitis B virus	37 (62)
Herpes simplex virus 1	0
Herpes simplex virus 2	0
Varicella-zoster virus	0
Epstein‒Barr virus	0
Cytomegalovirus	0
Human herpes virus 6	0
Human herpes virus 7	3 (5)

**Table 2.** Nail Samples for Capture-Based Next-Generation Sequencing.

ID	Gender	Age	HBeAg	HBV genotype	Level of HBV DNA in serum (Log copies/mL)	Level of HBV DNA in nail (Log copies/mL)	Level of HHV-7 DNA in nail (Log copies/mL)	Concentration of extracted tissue DNA (ng/μL)^*^
Ig18203	M	5	Positive	C	7.8	3.3	2.7	21
Ig18204	M	10	Positive	C	>9.0	2.3	3.0	10.3
Ig18205	M	8	Positive	B	8.9	3.8	2.8	8.2

^*^NanoDrop system

### Positive and negative controls

#### Capture-based NGS

To investigate the sensitivity and specificity of capture-based NGS to detect viral integration breakpoints, three positive control samples (two liver samples and one peripheral whole blood sample) and two negative control samples (one nail sample and one liver sample) were applied for capture-based NGS. Supplementary Table 3 and Supplementary Table 4 show the characteristics of the positive and negative control samples, respectively. Of the three positive control samples, two were positive for both HBV DNA and HHV-7 DNA, and one was positive for only HBV DNA. In addition to real-time PCR, the presence of HHV-7 DNA was confirmed by virus-specific nested PCR ^(25)^.

Supplementary Table 5 shows the summary of paired-end reads in the positive and negative controls. Supplementary Table 6 shows the summary of mapped and unmapped reads in the positive and negative controls. Supplementary Table 7 shows the summary of the mapped deduplicated reads in the positive and negative controls. The number of mapped deduplicated reads was 2,744,417, 2,971,358, 1,566,333, 1,170,606, and 1,596,927 for Ig18206 (positive control), Ig18807 (positive control), Ig18207 (positive control), Ig18208 (negative control), and Ig18808 (negative control), respectively. In the positive controls, the number of deduplicated reads mapped to the HBV reference sequence was 134,951 (Ig18206), 1,065,952 (Ig18807), and 116,346 (Ig18207). Although the real-time PCR results showed that Ig18206 was negative for HHV-7 DNA, 216 deduplicated reads were mapped to the HHV-7 reference sequence. In Ig18208 and Ig18207, 919 and 7,889 deduplicated reads were mapped to the HHV-7 reference sequence, respectively. In the negative controls, theoretically, no deduplicated read was mapped to the HBV and HHV-7 reference sequences. In Ig18208, however, 247 and 82 reads were mapped to the HBV and HHV-7 reference sequences, respectively. Moreover, four reads were mapped to the HHV-7 sequence in Ig18808. These findings indicate that capture-based NGS cannot avoid the possibility of false-positive mapping.

Table 3 shows the results of viral integration breakpoints, which are junction sites of virus DNA on host DNA, in the positive controls. A total of 618, 15,620, and 48 integration breakpoints of HBV were identified in Ig18206, Ig18807, and Ig18207, respectively. Moreover, nine and six viral integration breakpoints of HHV-7 were detected in Ig18807 and Ig18207, respectively. There was no HHV-7 integration breakpoint in Ig18206, which was negative for HHV-7 DNA. Supplementary Table 8, Table 9, Table 10, Table 11 show integration breakpoints. Since the data size of Ig18807 HBV integration breakpoints was large, we could not show it in the supplementary data. Table 4 shows the results of viral integration breakpoints in the negative controls. One integration breakpoint of HBV (chromosome 2: 215,418,186, integration read: AGATGCACCTTTTCTAGGAACTTGGAAGATTCTGCTTTTCCCAACCCTCAGACACTTTTTCACCTCTGCCTAATCATCTCATGTTCATGTCCTACTGTTC) and HHV-7 (chromosome 4: 190,123,115, integration read: CCCTAACCCTAACCCTAACCCTAACCCTAACCCCGAACCCGAACCCGAACCCGAACCCGAACCCGAACCCGAACCCTAACCCTAACCCTAACCCTAACC) was identified in Ig18208 and Ig18808, respectively.

**Table 3.** Integration Breakpoints of the Positive Controls.

ID	Total number of integration breakpoints	
	HBV	HHV-7
Ig18206 (liver)	618	0
	A breakpoint was detected in 1 read: 488 points	
	The same breakpoint was detected in	
	2 reads: 84 points	
	3 reads: 39 points	
	4 reads: 20 points	
	5 reads: 12 points	
	6 reads: 4 points	
	7 reads: 5 points	
	8 reads: 1 point	
	9 reads: 2 points	
	10 reads: 1 point	
	13 reads: 1 point	
	14 reads: 1 point	
Ig18807 (liver)	15,620	9
	A breakpoint was detected in 1 read: 14,950 points	A breakpoint was detected in 1 read: 9 points
	The same breakpoint was detected in	
	2 reads: 288 points	
	3 reads: 34 points	
	4 reads: 10 points	
	5 reads: 7 points	
	6 reads: 7 points	
	7 reads: 3 points	
	8 reads: 5 points	
	10 reads: 4 points	
	11 reads: 1 point	
	2 reads: 1 point	
	4 reads: 1 point	
	5 reads: 2 points	
	22 reads: 1 point	
	24 reads: 1 point	
	30 reads: 1 point	
	42 reads: 1 point	
	51 reads: 1 point	
	53 reads: 1 point	
	73 reads: 1 point	
Ig18207 (whole blood)	48	6
	A breakpoint was detected in 1 read: 42 points	A breakpoint was detected in 1 read: 6 points
	The same breakpoint was detected in 2 reads: 6 points	

**Table 4.** Integration Breakpoints of the Negative Controls.

ID	Total number of integration breakpoints	
	HBV	HHV-7
Ig18208 (nails)	1	0
	A breakpoint was detected in 1 read: 1 point	
Ig18808 (liver)	0	1
		A breakpoint was detected in 1 read: 1 point

#### Sanger sequencing

To confirm the viral integration breakpoints, conventional PCR and Sanger sequencing were performed. Supplementary Table 12 shows the primers for conventional PCR of the selected integration breakpoints.

#### HBV

We randomly selected integration breakpoints of HBV from the positive controls. Eleven (chromosome 1: 62,606,503; 10 read, chromosome 2: 215,418,187; 13 reads, chromosome 14: 68,792,458; 7 reads, chromosome 16: 14,545,694; 9 reads, chromosome 4: 180,952,665; 1 read, chromosome 4: 189,939,923; 1 read, chromosome 12: 95,875,424; 1 read, chromosome 6: 107,828,698; 1 read, chromosome 8: 15,444,425; 1 read, chromosome 1: 85,585,553; 1 read, chromosome 20: 3,976,073; 2 reads）of 618 breakpoints in Ig18206 (Supplementary Table 8), 2 (chromosome 18: 59,672,521; 73 reads, chromosome 10: 114,442,363; 42 reads) of 15,620 breakpoints in Ig18807, and 22 (chromosome 1: 197,394,924; 1 read, chromosome 1: 229,564,071; 1 read, chromosome 2: 25,251,880; 2 read, chromosome 2: 37,252,888; 1 read, chromosome 2: 123,855,129; 1 read, chromosome 4: 92 946,155; 1 read, chromosome 4: 128,649,683; 1 read, chromosome 4: 137,637,347; 1 read, chromosome 5: 44,013,798; 1 read, chromosome 5: 83,326,648; 1 read, chromosome 6: 15,085,893; 1 read, chromosome 6: 72,714,913; 1 read, chromosome 8: 89,410,201; 1 read, chromosome 11: 20,457,398; 1 read, chromosome 14: 96,321,592; 1 read, chromosome 17: 12,963,681; 1 read, chromosome 17: 44,543,785; 1 read, chromosome 17: 52,457,871; 1 read, chromosome 18: 29,629,322; 2 read, chromosome 21: 9,039,828; 1 read, chromosome X: 101,366,104; 1 read, chromosome Y: 11,204,217; 1 read) of 48 breakpoints in Ig18207 (Supplementary Table 10) were selected for conventional PCR and Sanger sequencing.

**Figure 1.**
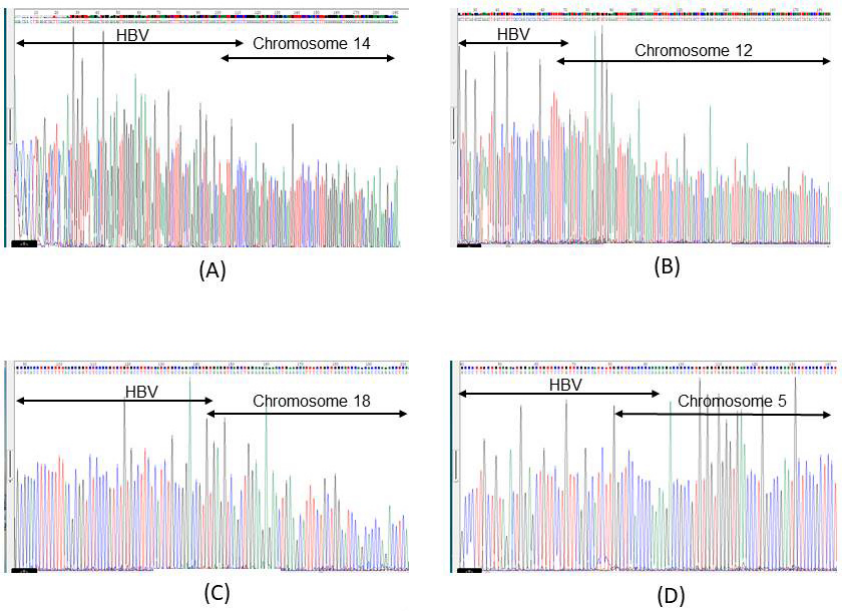
Electropherogram of chimeric reads by Sanger sequencing A) Ig18206 liver. Electropherogram of the conventional PCR results for chromosome 14: 68,792,458 +HBV (total read number: 7 reads). The chimeric read detected by capture-based next-generation sequencing (NGS) is as follows: TTGTACTAGGAGGCTGTAGGCATAAATTGGTCCCTCGGGGAAGTGAGTCTCGGGACAATGTTTTCCTCCACTCTTTGGGGGGAGCTGGGGATATGCGA B) Ig18206 liver. Electropherogram of the conventional PCR results for chromosome 12: 95,875,424 +HBV (total read number: 1 read). The chimeric read detected by capture-based NGS is as follows: GGCATAAATTGGTCTGTTCACCAGCACCATGCAACTTTTTCAAGTATCATTAAGAGTGTGGAAGTTTTGAAAGACTAAAATTCATTTCATACTGATAGCTC C) Ig18807 liver. Electropherogram of the conventional PCR results for chromosome 18: 59,672,521 +HBV (total read number: 73 reads). The chimeric read detected by capture-based NGS is as follows: CACGGGGCGCACCTCTCTTTACGCGGTCTCCCCGTCTGTGCCTTCTCATCTGCCGGACCGTGTGAGGTCAGCTGGAAGAGAACTGAAGCATTTCTGCTGGCATCCAGCACCCAGACCTCAGACACAAGAGTGAGGCCCT D) Ig18207 whole blood cells. Electropherogram of the conventional PCR results for chromosome 5: 44,013,798, +HBV (total read number: 1 read). The chimeric read detected by capture-based NGS is as follows: GGAGCATTCGGGCCAGGGTTCACCCCACCACACGGCGGTCTTTTGGGGTGGAGCCAAGATGGACAAATAGGAACAGCTCCAGTCTACAGCTCCCAGCTTGA However, Sanger sequencing showed that the integration breakpoint of the chimeric read was chromosome 5: 75,175,747. The chimeric read detected by Sanger sequencing is as follows: TCAAATCAGGTAGGAGCGGGAGCATTCGGGCCAGGGTTCACCCCACCACACGGCGGTCTTTTGGGGTGGAGCCAAGATGGCCGAACAGGAACAGCTCCAGTCTACAGC The underline indicates the difference in nucleotides between next-generation and Sanger sequencing.

Of the 11 selected breakpoints of HBV in Ig18206, 7 (chromosome 1: 62,606,503; chromosome 2: 215,418,187; chromosome 14: 68,792,458; chromosome 12: 95,875,424; chromosome 6: 107,828,698; chromosome 1: 85,585,553; chromosome 20: 3,976,073) were confirmed by Sanger sequencing (Figure 1A: chromosome 14: 68,792,458) (Figure 1B: chromosome 12; 95,857,424). Of the two selected breakpoints of HBV in Ig18807, two were confirmed by Sanger sequencing (Figure 1C: chromosome 18: 59,672,521). Only one (chromosome 5: 44,013,798) of the 22 selected breakpoints in Ig18207 was positive using conventional PCR. However, Sanger sequencing showed that the integration breakpoint was chromosome 5: 75,175,747 (Figure 1D). The DNA sequences encompassed with nested PCR primers were different in 2 nucleotides between 44,013,798 and 75,175,747 chimeric reads. In the negative controls, one integration breakpoint was detected in Ig18208 by capture-based NGS. Although the Mega BLAST results suggested that the integration read was an HBV/host chimeric sequence, Sanger sequencing could not find the chimeric sequence.

#### HHV-7

Since there was no report of HHV-7 integration into host genomic DNA, we evaluated the HHV-7 integration reads of positive controls using Mega BLAST software. The Mega BLAST results suggested that there was no HHV-7/host chimeric sequence in the nine integration reads of Ig18807 (five reads: a part of chromosome or mitochondria, four reads: unknown). The results of Mega BLAST indicated that the integration reads of Ig18807 could be false positives. In contrast, the Mega BLAST results suggested that all six reads of Ig18207 were HHV-7/host chimeric sequences. Two (chromosome 5: 73,571,363, chromosome 17: 80,061,876) of the nine integration points in Ig18807 (Supplementary Table 9) and five (chromosome 4: 40,983,878, chromosome 7: 153,676,002, chromosome 9: 104,407,448, chromosome 12: 107,555,401, chromosome 19: 53,455,783) of the six integration points in Ig18207 (Supplementary Table 11) were selected for conventional PCR. However, Sanger sequencing did not detect any HHV-7/host chimeric sequence in the positive controls. In the negative controls, one integration breakpoint was detected in Ig18808 by capture-based NGS. The Mega BLAST results suggested that the integration read was an unknown sequence. Therefore, we could not confirm the HHV-7 integration read by Sanger sequencing.

### Three nail samples

#### Capture-based NGS

Supplementary Table 13 shows the summary of the paired-end reads in the three nail samples. Supplementary Table 14 shows the summary of mapped and unmapped reads in the nail samples. Supplementary Table 15 shows that the number of mapped deduplicated reads was 918,580, 1,024,551, and 1,341,055 in Ig18203, Ig18204, and Ig18205, respectively.

Table 5 shows the results of the viral integration breakpoint of the three nail samples. In Ig18203, seven HBV and seven HHV-7 integration breakpoints were detected. The integration sites of HBV on chromosomes were three protein coding regions, two noncoding regions, and two “to be experimentally confirmed regions.” The integration sites of HHV-7 on chromosomes were two protein coding regions and five noncoding regions. However, the Mega BLAST results suggested that the HBV integration reads including six of the seven breakpoints were a part of the HBV genome (S region: n = 1, X region: n = 1, core region; n = 4), and one read including the remaining breakpoint was an HBV/host chimeric sequence (chromosome 13: 60,418,226). Moreover, the Mega BLAST results suggested that the HHV-7 integration reads included 5 (chromosome 2: 87,648,765 [noncoding region], chromosome 2: 87,648,781 [noncoding region], chromosome 2: 111,302,137 [noncoding region], chromosome 10: 5,744,298 [coding region], chromosome 21: 15,650,878 [noncoding region]) of the 7 breakpoints that were HHV-7/host chimeric sequences and 2 integration reads including the remaining 2 breakpoints that were unknown sequences. There were no HBV integration breakpoints in Ig18204 or Ig18205. In contrast, 37 and 59 integration breakpoints of HHV-7 were detected in Ig18204 and Ig18205, respectively. Supplementary Table 16, Table 17, Table 18, and Table 19 show the integration breakpoints. The Mega BLAST results suggested that one read including one breakpoint was an HHV-7/host chimeric sequence (chromosome 11: 4,493,831 [coding region]), one read including one breakpoint was a part of the HHV-7 genome, one read including one breakpoint was a part of the chromosome, and the remaining reads were unknown sequences in the 37 integration breakpoints of Ig18204. In addition, the Mega BLAST results suggested that 3 reads, including 1 individual breakpoint (chromosome 10: 109,841,707 [protein coding region]; HHV-7: 142,250 and 3,303) (chromosome 20: 25,781,706 [noncoding region]), were HHV-7/host genome chimeric sequences, 2 reads were a part of the chromosome, and the remaining reads, including 54 breakpoints, were unknown sequences in the 59 integration points of Ig18205.

**Table 5.** Integration Breakpoints of the Three Nail Samples.

No.	Total number of integration breakpoint	
	HBV	HHV-7
Ig18203	7	7
	A breakpoint was detected in 1 read: 5 points	A breakpoint was detected in 1 read: 6 points
	The same breakpoint was detected in 2 reads: 1 point	The same breakpoint was detected in 2 reads: 1 point
	The same breakpoint was detected in 3 reads: 1 point	
Ig18204	0	37
		A breakpoint was detected in 1 read: 29 points
		The same breakpoint was detected in 2 reads: 7 points
		The same single breakpoint was detected in 3 reads: 1 point
Ig18205	0	59
		A breakpoint was detected in 1 read: 50 points
		The same breakpoint was detected in 2 reads: 7 points
		The same breakpoint was detected in 4 reads: 2 points

#### Sanger sequencing

To confirm these integration breakpoints in the three nail samples, conventional PCR and Sanger sequencing were performed. Integration breakpoints, which were suggested as virus/host chimeric sequences by Mega BLAST, were selected for nested PCR (Ig18203 HBV chromosome 13: 60,418,226;

TGGACTCATAAGGTGGGAAACTTTACTGGGCTTTATTCTTCTACTGTACCATTTCTGATCTTGTCCTTTCTGCTTGGATTTTTTTCTCCATTTAAGTTA) (Ig18203 HHV-7 chromosome 2: 87,648,781; GGCAAGAGGCATCTCAAATTAAACCCAACTAAAAGAGATGTGCCTTGGAAAAGTCATGAAATTTAGCATAGCCACGTCCACGTCTGTTGTAAGGTCTGTG) (Ig18204 HHV-7, chromosome 11: 4,493,831; GACAGGAAGTGATACCAAATTAATGCTAGGAAGAAAAAAGGCAAAAAGTAGACAACACAAGCATGCCAATATTAATAATGCCAGGGACAACAATGATCCTC) (Ig18205 HHV-7, chromosome 20: 25,781,706; GTGATGATGATGGTGAGGATGGTGATGGTGAAGTCTGTTGTGAAGCTTGCATGCTTGAGGTGCCTCGAGTAGATGGAAAAAATTTTGTTGGT). Supplementary Table 12 shows the primers for conventional PCR. However, we could not confirm any of these integration breakpoints by Sanger sequencing.

## Discussion

HBV DNA was detected in 62% of patients with chronic HBV. Except for HBV, only HHV-7 DNA was detected in three (5%) patients. HHV-7 has a tropism for CD4+ T lymphocytes and is transmissible through saliva. HHV-7 infects lymphoid tissue, salivary gland, tonsils, liver, kidney, lungs, and skin ^(26)^. To the best of our knowledge, there is no report of HHV-7 DNA being detected in nails.

Nails are unique skin appendages, and HHV-6 can infect the skin ^(27)^. Moreover, chromosomal integration of HHV-6 is well-known inherited chromosomally integrated HHV-6: iciHHV-6, which is detected in approximately 0.2%-1% of the general human population ^(26), (28), (29), (30), (31)^. It is hypothesized that the telomere-like repeat sequence of HHV-6, which is identical to the human telomere sequence (TTAGGG), causes the initial integration of the HHV-6 genome into host somatic cells ^(26), (28), (29), (31)^. This study showed that HHV-6 DNA was undetectable in nails. However, HHV-6 and HHV-7 share many common properties. HHV-6 and HHV-7 share approximately 50% amino acid identity ^(26)^. Since HHV-7 has a telomere-like sequence, there is a possibility that chromosomal integration of HHV-7 DNA can be detected in nails. A previous study reported clinical cases of chromosomal integration of HHV-7 in hair follicles and peripheral blood cells ^(32)^.

As positive samples, two liver samples and one peripheral blood sample were used for capture-based NGS. The integration breakpoint of HBV and HHV-7 was detected in the positive samples. Most of the integration breakpoints of HBV in the liver were confirmed by Sanger sequencing. Even one read of the HBV integration breakpoint was confirmed by Sanger sequencing. These findings suggest that the sensitivity of capture-based NGS is high. Although the sequence was not identical to the virus/human chimeric read, which was detected by capture-based NGS, Sanger sequencing showed the integration of HBV DNA in peripheral blood cells. However, the number of integration breakpoints of HHV-7 was much fewer than that of HBV detected by the capture-based NGS. All the integration breakpoints of HHV-7 were detected in a single read. Moreover, there was no integration breakpoint of HHV-7, which was confirmed by Sanger sequencing. These findings indicate two possibilities. One is that the integrations of HHV-7 DNA into genomic DNA are false-positive results. Previous articles suggest that NGS methods have the potential for artifacts, which are generated by PCR during library preparation ^(33), (34)^. Foster et al. reported that HHV-7 is a typical false-positive virus/human chimera candidate. The false-positive virus/human chimera is presumed to be caused by short tandem repeat (e.g., human telomerase) ^(33)^. The other is that the detection of HHV-7/human chimeric sequences is difficult by conventional PCR due to the low frequency of integration. However, this speculation contradicts the fact that a single read of the HBV/human chimeric sequence could be confirmed by conventional PCR in this study. In the negative controls, deduplicated reads were mapped to the HBV and HHV-7 references as exogenous DNA. In addition, one HBV/human and one HHV-7/human chimeric read were detected in negative controls by capture-based NGS. These findings suggest that false-positive results cannot be ruled out in capture-based NGS.

Finally, three nail samples, which were positive for both HBV DNA and HHV-7 DNA, were analyzed by capture-based NGS. Most of the viral integration breakpoints were detected in a single read. Unfortunately, these chimeric sequences were not suggested as virus/host chimeric sequences by Mega BLAST. Every unknown read, which had no similar sequence in Mega BLAST analysis, contained a TTAGGG short repeat sequence or homopolymer. HBV integration sites are usually randomly distributed among chromosomes ^(5)^. Moreover, iciHHV-6 integration is normally observed in the telomeric region of chromosomes ^(29)^. Although only one report shows HHV-7 integration into host chromosomes, HHV-7 integration was also detected in the telomeric region ^(32)^. In this study, however, HHV-7 integration breakpoints were not located in the telomeric region. Since the amount of nail DNA for conventional PCR was extremely small, we tried to confirm only one integration breakpoint, which was suggested as a virus/host chimeric sequence by Mega BLAST, in each nail sample. Unfortunately, the confirmation of viral integration sites by Sanger sequencing was not successful. Similar to the positive control, the low specificity of capture-based NGS, low frequency of viral integration, and unsuitable design of PCR primers might have caused these failures in finding viral breakpoints. In addition to nail HBV DNA and CMV DNA, which were reported in the previous studies ^(8), (9), (11)^, this study shows that HHV-7 DNA is also detected in fingernails. Although the clinical usefulness of nail viral DNA is unclear, fingernails might be valuable materials to clarify the interactions between human genomic and viral DNA.

In conclusion, HHV-7 DNA and HBV DNA were detected by real-time PCR in the nails of patients with chronic HBV infection. Capture-based NGS demonstrated that the integration of HBV DNA and HHV-7 DNA into host genomic DNA occurred in nails. However, Sanger sequencing could not confirm the virus/host chimeric sequences.

## Article Information

### Conflicts of Interest

None

### Sources of Funding

This work was supported by JSPS KAKENHI (Grant-in-Aid for Scientific Research) grant numbers 18K08451 and 21K08517.

### Author Contributions

HK contributed to the design of this study and drafted this manuscript. AI, HH, SU, and TF participated in the data collection and critically revised the manuscript. All of the authors concurred with the submission and take responsibility for the manuscript.

### Approval by Institutional Review Board (IRB)

The study protocols were approved by the ethics committee of Toho University Sakura Medical Center (no. 2015-073) and Eastern Yokohama Hospital (no. 2015010). This study was performed in accordance with the ethical guidelines of the 1975 Declaration of Helsinki. Written informed consent was obtained from all patients or legal guardians prior to sample collection.

## Supplement

Supplementary Figure 1 DNA probe for targeted enrichment. The probes were designed to cover the entire HBV and HHV-7 genomes. The length of the probe is 120 bp. Each probe overlapped by 60 bp

Supplementary Figure 2 A soft-clipped read is a chimeric read of the human and viral DNA sequences

Supplementary Figure 3 Algorithm of data analysis of next-generation sequencing

Supplementary Table 1

Supplementary Table 2

Supplementary Table 3

Supplementary Table 4

Supplementary Table 5

Supplementary Table 6

Supplementary Table 7

Supplementary Table 8

Supplementary Table 9

Supplementary Table 10

Supplementary Table 11

Supplementary Table 12

Supplementary Table 13

Supplementary Table 14

Supplementary Table 15

Supplementary Table 16

Supplementary Table 17

Supplementary Table 18

Supplementary Table 19
