# Supplementary figures and images for: Integration of Viral Genome to Human Genomic DNA in Nails of Patients with Chronic Hepatitis B Virus Infection
Source: JMA J. 2023 Sep 29;6(4):426–36. doi: 10.31662/jmaj.2023-0082 (PMC10628332; doi:10.31662/jmaj.2023-0082)

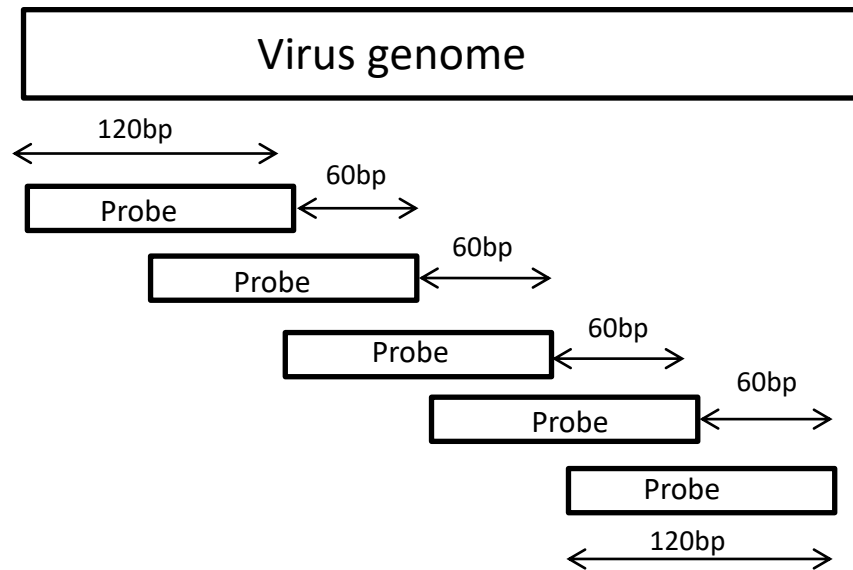

Supplementary figure 1

Supplement: Supplementary Figure 1 — DNA probe for targeted enrichment. The probes were designed to cover the entire HBV and HHV-7 genomes. The length of the probe is 120 bp. Each probe overlapped by 60 bp [file 2433-3298-6-4-426-s001.pdf]

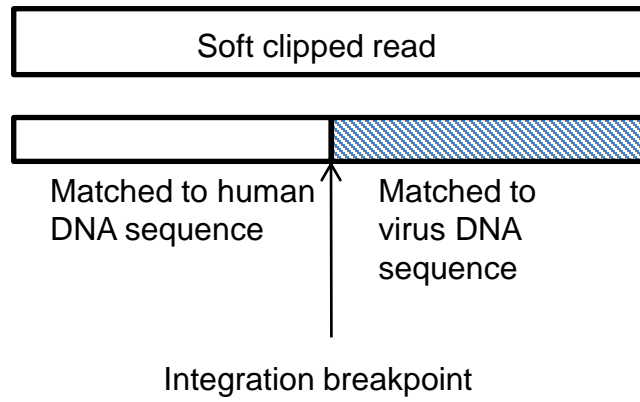

Supplementary figure 2

Supplement: Supplementary Figure 2 — A soft-clipped read is a chimeric read of the human and viral DNA sequences [file 2433-3298-6-4-426-s002.pdf]

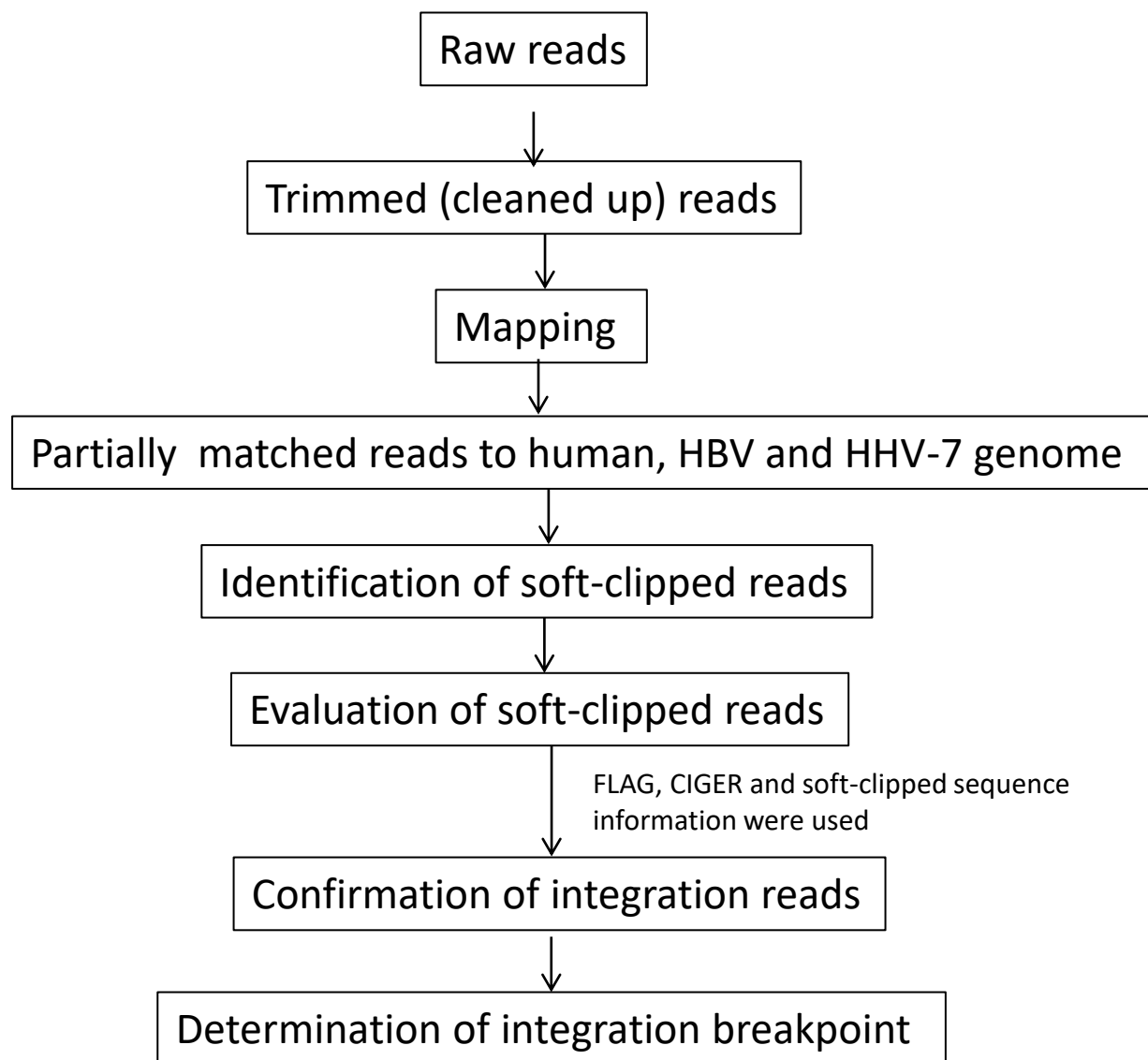

Supplementary figure 3

Supplement: Supplementary Figure 3 — Algorithm of data analysis of next-generation sequencing [file 2433-3298-6-4-426-s003.pdf]
