# Supplementary material for: Integration of Viral Genome to Human Genomic DNA in Nails of Patients with Chronic Hepatitis B Virus Infection
Source: JMA J. 2023 Sep 29;6(4):426–36. doi: 10.31662/jmaj.2023-0082 (PMC10628332; doi:10.31662/jmaj.2023-0082)
Supplement: Supplementary Table 1 [file 2433-3298-6-4-426-s004.pdf]

Supplementary Table 1. HBV xGen Lockdown probe

Sequence Name	Sequence
663987_30659705_HBV_sequence_AB033550.1_pNDR260_1_1	CTCCACCACATTCCACCAAGCTCTGCTAGATCCCAGAGTGAGGGGCCTATATTTTCCTGCTGGTGGCTCCAGTTCCGGAACAGTAAACCCTGTTCCGACTACTGCCTCACCCATATCGTC
663987_30659705_HBV_sequence_AB033550.1_pNDR260_1_2	TGGTGGCTCCAGTTCCGGAACAGTAAACCCTGTTCCGACTACTGCCTCACCCATATCGTCAATCTTCTCGAGGACTGGGGACCCTGCACAGAACATGGAGAACACAACATCAGGATTCCT
663987_30659705_HBV_sequence_AB033550.1_pNDR260_1_3	AATCTTCTCGAGGACTGGGGACCCTGCACAGAACATGGAGAACACAACATCAGGATTCCTAGGACCCCTGCTCGTGTTACAGGCGGGGTTTTTCTTGTTGACAAAAATCCTCACAATACC
663987_30659705_HBV_sequence_AB033550.1_pNDR260_1_4	AGGACCCCTGCTCGTGTTACAGGCGGGGTTTTTCTTGTTGACAAAAATCCTCACAATACCACAGAGTCTAGACTCGTGGTGGACTTCTCTCAATTTTCTAGGGGGAGCACCCACGTGTCC
663987_30659705_HBV_sequence_AB033550.1_pNDR260_1_5	ACAGAGTCTAGACTCGTGGTGGACTTCTCTCAATTTTCTAGGGGGAGCACCCACGTGTCCTGGCCAAAATTCGCAGTCCCCAACCTCCAATCACTCACCAACCTCTTGTCCTCCAATTTG
663987_30659705_HBV_sequence_AB033550.1_pNDR260_1_6	TGGCCAAAATTCGCAGTCCCCAACCTCCAATCACTCACCAACCTCTTGTCCTCCAATTTGTCCTGGCTATCGCTGGATGTGTCTGCGGCGTTTTATCATATTCCTCTTCATCCTGCTGCT
663987_30659705_HBV_sequence_AB033550.1_pNDR260_1_7	TCCTGGCTATCGCTGGATGTGTCTGCGGCGTTTTATCATATTCCTCTTCATCCTGCTGCTATGCCTCATCTTCTTGTTGGTTCTTCTGGACTACCAAGGTATGTTGCCCGTTTGTCCTCT
663987_30659705_HBV_sequence_AB033550.1_pNDR260_1_8	ATGCCTCATCTTCTTGTTGGTTCTTCTGGACTACCAAGGTATGTTGCCCGTTTGTCCTCTACTTCCAGGAACAACAACTACCAGCACGGGACCATGCAAGACCTGCACGATTCCTGCTCA

663987_30659705_HBV_sequence_AB033550.1_pNDR260_1_9	ACTTCCAGGAACAACAACTACCAGCACGGGACCATGCAAGACCTGCACGATTCCTGCTCAAGGAACCTCTATGTTTCCCTCTTGTTGCTGTACAAAACCTTCGGACGGAAACTGCACTTG
663987_30659705_HBV_sequence_AB033550.1_pNDR260_1_10	AGGAACCTCTATGTTTCCCTCTTGTTGCTGTACAAAACCTTCGGACGGAAACTGCACTTGTATTCCCATCCCATCATCCTGGGCTTTCGCAAGATTCCTATGGGAGTGGGCCTCAGTCCG
663987_30659705_HBV_sequence_AB033550.1_pNDR260_1_11	TATTCCCATCCCATCATCCTGGGCTTTCGCAAGATTCCTATGGGAGTGGGCCTCAGTCCGTTTCTCCTGGCTCAGTTTACTAGTGCCATTTGTTCAGTGGTTCGTAGGGCTTTCCCCCAC
663987_30659705_HBV_sequence_AB033550.1_pNDR260_1_12	TTTCTCCTGGCTCAGTTTACTAGTGCCATTTGTTCAGTGGTTCGTAGGGCTTTCCCCCACTGTTTGGCTTTCAGTTATATGGATGATGTGGTATTGGGGGCCAAGTCTGTACAACATCTT
663987_30659705_HBV_sequence_AB033550.1_pNDR260_1_13	TGTTTGGCTTTCAGTTATATGGATGATGTGGTATTGGGGGCCAAGTCTGTACAACATCTTGAGTCCCTTTTTACCTCTATTACCAATTTTCTTTTGTCTTTGGGTATACATTTGAATCCT
663987_30659705_HBV_sequence_AB033550.1_pNDR260_1_14	GAGTCCCTTTTTACCTCTATTACCAATTTTCTTTTGTCTTTGGGTATACATTTGAATCCTAATAAAACCAAACGTTGGGGCTACTCCCTTAACTTCATGGGATATGTAATTGGAAGTTGG
663987_30659705_HBV_sequence_AB033550.1_pNDR260_1_15	AATAAAACCAAACGTTGGGGCTACTCCCTTAACTTCATGGGATATGTAATTGGAAGTTGGGGTACTTTACCACAGGAACATATTGTACGGAAACTCAAGCAATGTTTTCGAAAACTGCCT
663987_30659705_HBV_sequence_AB033550.1_pNDR260_1_16	GGTACTTTACCACAGGAACATATTGTACGGAAACTCAAGCAATGTTTTCGAAAACTGCCTGTAAATAGACCTATTGATTGGAAAGTATGTCAAAGAATTGTGGGTCTTTTGGGCTTTGCT
663987_30659705_HBV_sequence_AB033550.1_pNDR260_1_17	GTAAATAGACCTATTGATTGGAAAGTATGTCAAAGAATTGTGGGTCTTTTGGGCTTTGCTGCCCCTTTTACACAATGTGGCTATCCTGCCTTGATGCCTTTATATGCATGTATACACTCT

663987_30659705_HBV_sequence_AB 033550.1_pNDR260_1_18	GCCCCTTTTACACAATGTGGCTATCCTGCCTTGATGCCTTTATATGCATGTATACACTCTAAGCAGGCTTTCACTT TCTCGCCAACTTACAAGGCCTTTCTGTGTAAACAATATCTGCAC
663987_30659705_HBV_sequence_AB 033550.1_pNDR260_1_19	AAGCAGGCTTTCACTTTCTCGCCAACTTACAAGGCCTTTCTGTGTAAACAATATCTGCACCTTTACCCCGTTGCC CGGCAACGGTCAGGTCTCTGCCAAGTGTTTGCTGACGCAACCCCC
663987_30659705_HBV_sequence_AB 033550.1_pNDR260_1_20	CTTTACCCCGTTGCCCGGCAACGGTCAGGTCTCTGCCAAGTGTTTGCTGACGCAACCCCCACTGGATGGGGC TTGGCCATAGGCCATCGGCGCATGCGCGGAACCTTTGTGGCTCCTCTG
663987_30659705_HBV_sequence_AB 033550.1_pNDR260_1_21	ACTGGATGGGGCTTGGCCATAGGCCATCGGCGCATGCGCGGAACCTTTGTGGCTCCTCTGCCGATCCATACTG CGGAACTCCTAGCAGCTTGTTTTGCTCGCAGCCGGTCTGGAGCAAAA
663987_30659705_HBV_sequence_AB 033550.1_pNDR260_1_22	CCGATCCATACTGCGGAACTCCTAGCAGCTTGTTTTGCTCGCAGCCGGTCTGGAGCAAAACTTATCGGGACTG ACAACTCTGTTGTCCTCTCTCGGAAATACACCTCCTTCCCATGGCTG
663987_30659705_HBV_sequence_AB 033550.1_pNDR260_1_23	CTTATCGGGACTGACAACTCTGTTGTCCTCTCTCGGAAATACACCTCCTTCCCATGGCTGCTCGGGTGTGCTGC CAACTGGATCCTTCGCGGGACGTCCTTTGTCTACGTCCCGTCGGCG
663987_30659705_HBV_sequence_AB 033550.1_pNDR260_1_24	CTCGGGTGTGCTGCCAACTGGATCCTTCGCGGGACGTCCTTTGTCTACGTCCCGTCGGCGCTGAATCCCGCG GACGACCCGTCTCGGGGCCGTTTGGGGCTCTATCGTCCCCTTCTTCAT
663987_30659705_HBV_sequence_AB 033550.1_pNDR260_1_25	CTGAATCCCGCGGACGACCCGTCTCGGGGCCGTTTGGGGCTCTATCGTCCCCTTCTTCATCTGCCGTTCCGG CCGACCACGGGGCGCACCTCTCTTTACGCGGTCTCCCCGTCTGTGCCT
663987_30659705_HBV_sequence_AB 033550.1_pNDR260_1_26	CTGCCGTTCCGGCCGACCACGGGGCGCACCTCTCTTTACGCGGTCTCCCCGTCTGTGCCTTCTCATCTGCCG GACCGTGTGCACTTCGCTTCACCTCTGCACGTCGCATGGAGACCACCG

663987_30659705_HBV_sequence_AB033550.1_pNDR260_1_27	TCTCATCTGCCGGACCGTGTGCACTTCGCTTCACCTCTGCACGTCGCATGGAGACCACCGTGAACGCCCACCAGGTCTTGCCCAAGGTCTTACATAAGAGGACTCTTGGACTCTCATCAA
663987_30659705_HBV_sequence_AB033550.1_pNDR260_1_28	TGAACGCCCACCAGGTCTTGCCCAAGGTCTTACATAAGAGGACTCTTGGACTCTCATCAATGTCAACGACCGACCTTGAGGCATACTTCAAAGACTGTTTGTTTAAGGACTGGGAGGAGT
663987_30659705_HBV_sequence_AB033550.1_pNDR260_1_29	TGTCAACGACCGACCTTGAGGCATACTTCAAAGACTGTTTGTTTAAGGACTGGGAGGAGTTGGGGGAGGAGATTAGGTTAAAGGTCTTTGTACTAGGAGGCTGTAGGCATAAATTGGTCT
663987_30659705_HBV_sequence_AB033550.1_pNDR260_1_30	TGGGGGAGGAGATTAGGTTAAAGGTCTTTGTACTAGGAGGCTGTAGGCATAAATTGGTCTGTTCACCAGCACCATGCAACTTTTTCACCTCTGCCTAATCATCTCATGTTCATGTCCTAC
663987_30659705_HBV_sequence_AB033550.1_pNDR260_1_31	GTTCACCAGCACCATGCAACTTTTTCACCTCTGCCTAATCATCTCATGTTCATGTCCTACTGTTCAAGCCTCCAAGCTGTGCCTTGGGTGGCTTTGGGGCATGGACATTGACCCGTATAA
663987_30659705_HBV_sequence_AB033550.1_pNDR260_1_32	TGTTCAAGCCTCCAAGCTGTGCCTTGGGTGGCTTTGGGGCATGGACATTGACCCGTATAAAGAATTTGGAGCTTCTGTGGAGTTACTCTCTTTTTTGCCTTCTGACTTCTTTCCTTCTAT
663987_30659705_HBV_sequence_AB033550.1_pNDR260_1_33	AGAATTTGGAGCTTCTGTGGAGTTACTCTCTTTTTTGCCTTCTGACTTCTTTCCTTCTATTCGAGATCTCCTCGACACCGCCTCTGCTCTGTATCGGGAGGCCTTAGAGTCTCCGGAACA
663987_30659705_HBV_sequence_AB033550.1_pNDR260_1_34	TCGAGATCTCCTCGACACCGCCTCTGCTCTGTATCGGGAGGCCTTAGAGTCTCCGGAACATTGTTCACCTCACCATACAGCACTCAGGCAAGCTATTCTGTGTTGGGGTGAGTTGATGAA
663987_30659705_HBV_sequence_AB033550.1_pNDR260_1_35	TTGTTCACCTCACCATACAGCACTCAGGCAAGCTATTCTGTGTTGGGGTGAGTTGATGAATCTGGCCACCTGGGTGGGAAGTAATTTGGAAGACCCAGCATCCAGGGAATTAGTAGTCAG

663987_30659705_HBV_sequence_AB033550.1_pNDR260_1_36	TCTGGCCACCTGGGTGGGAAGTAATTTGGAAGACCCAGCATCCAGGGAATTAGTAGTCAGCTATGTCAATGTTAATATGGGCCTAAAAATCAGACAACTATTGTGGTTTCACATTTCCTG
663987_30659705_HBV_sequence_AB033550.1_pNDR260_1_37	CTATGTCAATGTTAATATGGGCCTAAAAATCAGACAACTATTGTGGTTTCACATTTCCTGTCTTACTTTTGGAAGAGAAACTGTTCTTGAGTATTTGGTATCTTTTGGAGTGTGGATTCG
663987_30659705_HBV_sequence_AB033550.1_pNDR260_1_38	TCTTACTTTTGGAAGAGAAACTGTTCTTGAGTATTTGGTATCTTTTGGAGTGTGGATTCGCACTCCTCCAGCTTACAGACCACCAAATGCCCCTATCTTATCAACACTTCCGGAAACTAC
663987_30659705_HBV_sequence_AB033550.1_pNDR260_1_39	CACTCCTCCAGCTTACAGACCACCAAATGCCCCTATCTTATCAACACTTCCGGAAACTACTGTTGTTAGACGACGAGGCAGGTCCCCTAGAAGAAGAACTCCCTCGCCTCGCAGACGAAG
663987_30659705_HBV_sequence_AB033550.1_pNDR260_1_40	TGTTGTTAGACGACGAGGCAGGTCCCCTAGAAGAAGAACTCCCTCGCCTCGCAGACGAAGGTCTCAATCGCCGCGTCGCAGAAGATCTCAATCTCGGGAATCTCAATGTTAGTATCCCTT
663987_30659705_HBV_sequence_AB033550.1_pNDR260_1_41	GTCTCAATCGCCGCGTCGCAGAAGATCTCAATCTCGGGAATCTCAATGTTAGTATCCCTTGGACTCATAAGGTGGGAAACTTTACTGGGCTTTATTCTTCTACTGTACCTGTCTTTAATC
663987_30659705_HBV_sequence_AB033550.1_pNDR260_1_42	GGACTCATAAGGTGGGAAACTTTACTGGGCTTTATTCTTCTACTGTACCTGTCTTTAATCCTGAGTGGCAAACTCCCTCCTTTCCTAACATTCATTTACAGGAGGACATTATTAATAGAT
663987_30659705_HBV_sequence_AB033550.1_pNDR260_1_43	CTGAGTGGCAAACTCCCTCCTTTCCTAACATTCATTTACAGGAGGACATTATTAATAGATGTGAACAATATGTGGGCCCTCTTACAACTAATGAAAAAAGGAGATTAAAATTAATTATGC
663987_30659705_HBV_sequence_AB033550.1_pNDR260_1_44	GTGAACAATATGTGGGCCCTCTTACAACTAATGAAAAAAGGAGATTAAAATTAATTATGCCTGCTAGGTTTTATCCTAACCTTACCAAATACTTGCCCTTGGATAAAGGCATTAAACCTT

663987_30659705_HBV_sequence_AB033550.1_pNDR260_1_45	CTGCTAGGTTTTATCCTAACCTTACCAAATACTTGCCCTTGGATAAAGGCATTAAACCTTATTATCCTGAACATGCAGTTAATCATTACTTCAAAACTAGGCATTATTTACATACTCTGT
663987_30659705_HBV_sequence_AB033550.1_pNDR260_1_46	ATTATCCTGAACATGCAGTTAATCATTACTTCAAAACTAGGCATTATTTACATACTCTGTGGAAGGCCGGCATTCTATATAAGAGAGAAACTACACGCAGCGCTTCATTTTGTGGGTCAC
663987_30659705_HBV_sequence_AB033550.1_pNDR260_1_47	GGAAGGCCGGCATTCTATATAAGAGAGAAACTACACGCAGCGCTTCATTTTGTGGGTCACCATATTCTTGGGAACAAGAGCTACAGCATGGGAGGTTGGTCTTCCAAACCTCGACAAGGC
663987_30659705_HBV_sequence_AB033550.1_pNDR260_1_48	CATATTCTTGGGAACAAGAGCTACAGCATGGGAGGTTGGTCTTCCAAACCTCGACAAGGCATGGGGACGAATCTTTCTGTTCCCAATCCTCTGGGATTTTTTCCCGATCACCAGTTGGAC
663987_30659705_HBV_sequence_AB033550.1_pNDR260_1_49	ATGGGGACGAATCTTTCTGTTCCCAATCCTCTGGGATTTTTTCCCGATCACCAGTTGGACCCTGCGTTCGGAGCCAACTCAAACAATCCAGATTGGGACTTCAACCCCAACAAGGATCAC
663987_30659705_HBV_sequence_AB033550.1_pNDR260_1_50	CCTGCGTTCGGAGCCAACTCAAACAATCCAGATTGGGACTTCAACCCCAACAAGGATCACTGGCCAGAGGCAAATCAGGTAGGAGCGGGAGCATTCGGGCCAGGGTTCACCCCACCACAC
663987_30659705_HBV_sequence_AB033550.1_pNDR260_1_51	TGGCCAGAGGCAAATCAGGTAGGAGCGGGAGCATTCGGGCCAGGGTTCACCCCACCACACGGCGGTCTTTTGGGGTGGAGCCCTCAGGCTCAGGGCATATTGACCACAGTGCCAGCAGCG
663987_30659705_HBV_sequence_AB033550.1_pNDR260_1_52	GGCGGTCTTTTGGGGTGGAGCCCTCAGGCTCAGGGCATATTGACCACAGTGCCAGCAGCGCCTCCTCCTGCCTCCACCAATCGGCAGTCAGGAAGACAGCCTACTCCCATCTCTCCACCT
663987_30659705_HBV_sequence_AB033550.1_pNDR260_1_53	CCTCCTCCTGCCTCCACCAATCGGCAGTCAGGAAGACAGCCTACTCCCATCTCTCCACCTCTAAGAGACAGTCATCCTCAGGCCATGCAGTGGAA
