# Supplementary material for: Integration of Viral Genome to Human Genomic DNA in Nails of Patients with Chronic Hepatitis B Virus Infection
Source: JMA J. 2023 Sep 29;6(4):426–36. doi: 10.31662/jmaj.2023-0082 (PMC10628332; doi:10.31662/jmaj.2023-0082)
Supplement: Supplementary Table 2 [file 2433-3298-6-4-426-s005.pdf]

Supplementary Table 2. HHV-7 xGen Lockdown probe

Sequence name	Sequence
664157_30677280_HHV-7_JI_U43400.1_144861bp_1_2	CTAACCCTAACCCTAACCCTAACCCTAGCTCTAAGCCTAACCCCAGCCCTAACCCTAACCCTAGCTCTAAGCCTAACCCCAACCCTAACCCTAGCTCTCACTGTCACCCTAACACTAGCT
664157_30677280_HHV-7_JI_U43400.1_144861bp_1_1	CCCCCCCGTTTCGTATTTCAAATCCTAAATAACCCCCGGGGGGTAAAAAAAGGGGGGGAGCTAACCCTAACCCTAACCCTAACCCTAGCTCTAAGCCTAACCCCAGCCCTAACCCTAACC
664157_30677280_HHV-7_JI_U43400.1_144861bp_1_3	CTAGCTCTAAGCCTAACCCCAACCCTAACCCTAGCTCTCACTGTCACCCTAACACTAGCTCCAAGTCATCTGTTCTAGATCCTATCCATATCTGCCCTGACTCCTGGTTCCTTACCGCTC
664157_30677280_HHV-7_JI_U43400.1_144861bp_1_4	CCAAGTCATCTGTTCTAGATCCTATCCATATCTGCCCTGACTCCTGGTTCCTTACCGCTCCGAGCCCCACCCTCCGTCCCGCCCTCCTCCTGTTCTCCATGCCCTGCCTTCTCAACCCTT
664157_30677280_HHV-7_JI_U43400.1_144861bp_1_5	CGAGCCCCACCCTCCGTCCCGCCCTCCTCCTGTTCTCCATGCCCTGCCTTCTCAACCCTTCCTCTTCCACGCCCACATTGCCTCTGCACTCCGCGCTCTCTTGGCTGTGCGCCCTGCCTT
664157_30677280_HHV-7_JI_U43400.1_144861bp_1_6	CCTCTTCCACGCCCACATTGCCTCTGCACTCCGCGCTCTCTTGGCTGTGCGCCCTGCCTTTCCGTGACCTACTGGGAGCGCCGCCAAATCTGTTTTGCCCCGCCCCTGCGCGCGCGGGAA
664157_30677280_HHV-7_JI_U43400.1_144861bp_1_7	TCCGTGACCTACTGGGAGCGCCGCCAAATCTGTTTTGCCCCGCCCCTGCGCGCGCGGGAACTGTCGGCGCCGCGCTGCTGCTAGCCCGCCTTCCAGAGCTCCCTCCCTCCGTCTGCCTCC
664157_30677280_HHV-7_JI_U43400.1_144861bp_1_8	CTGTCGGCGCCGCGCTGCTGCTAGCCCGCCTTCCAGAGCTCCCTCCCTCCGTCTGCCTCCTCACCCTTGCCACTCACCCTTCCATCTCTTCTATCACAGACTCTGTGTTACACCACCTAT
664157_30677280_HHV-7_JI_U43400.1_144861bp_1_9	TCACCCTTGCCACTCACCCTTCCATCTCTTCTATCACAGACTCTGTGTTACACCACCTATGACTGCTGCAACCACAGAACATTTTGCTCTCCGCGCGGCACTCAATCGTTACTGGTGGCT
664157_30677280_HHV-7_JI_U43400.1_144861bp_1_10	GACTGCTGCAACCACAGAACATTTTGCTCTCCGCGCGGCACTCAATCGTTACTGGTGGCTGCTTCTGGGACGACACAAGCTCAGTTTGGTATGCAACTACGTCACAGCTCATCGCCAACA
664157_30677280_HHV-7_JI_U43400.1_144861bp_1_11	GCTTCTGGGACGACACAAGCTCAGTTTGGTATGCAACTACGTCACAGCTCATCGCCAACAGTTACTGCCGCTGCCGTGGCCCGAACAGGAATTTCTCCAACTTGACCCGGCCCCCTACTC

664157_30677280_HHV-7_JI_U43400.1_144861bp_1_12	GTTACTGCCGCTGCCGTGGCCCGAACAGGAATTTCTCCAACTTGACCCGGCCCCCTACTCCAATCTCCGCAACCGTGTCGCTCACCATCTCCATCGCGGCTGGCCAGCGGCACACAACAC
664157_30677280_HHV-7_JI_U43400.1_144861bp_1_13	CAATCTCCGCAACCGTGTCGCTCACCATCTCCATCGCGGCTGGCCAGCGGCACACAACACATGTAAGCTACCGTACATCTCTTTCACAAACCCAGGGCTCACATAGAGACAAGCACAAGC
664157_30677280_HHV-7_JI_U43400.1_144861bp_1_14	ATGTAAGCTACCGTACATCTCTTTCACAAACCCAGGGCTCACATAGAGACAAGCACAAGCTCGCGCAATGACATTAAAACCTCCCATCATTGTCCTTTCCTGTCGCTTTGCCGATAACGT
664157_30677280_HHV-7_JI_U43400.1_144861bp_1_15	TCGCGCAATGACATTAAAACCTCCCATCATTGTCCTTTCCTGTCGCTTTGCCGATAACGTCTTTGCTCTATCGCAGGTTTCGACCCCCGTCCTTACTTCCCCAATGCTAAAGTCAAGCTG
664157_30677280_HHV-7_JI_U43400.1_144861bp_1_16	CTTTGCTCTATCGCAGGTTTCGACCCCCGTCCTTACTTCCCCAATGCTAAAGTCAAGCTGCTTCCGCTCGGCTCCATCACCCTTACCAGATCATTCTCCAGTGACGAGCCTCATCCTATT
664157_30677280_HHV-7_JI_U43400.1_144861bp_1_17	CTTCCGCTCGGCTCCATCACCCTTACCAGATCATTCTCCAGTGACGAGCCTCATCCTATTGGTGATGATGTGCATCACAGTCATGACCGGGGTGACTACCATACTGTTATCTGCAGCTGG
664157_30677280_HHV-7_JI_U43400.1_144861bp_1_18	GGTGATGATGTGCATCACAGTCATGACCGGGGTGACTACCATACTGTTATCTGCAGCTGGCTCACAGGAACCTCCCCGATCCTAGTGCTGCTTCAAGGACCGGACGGCAGCATCTATTGC
664157_30677280_HHV-7_JI_U43400.1_144861bp_1_19	CTCACAGGAACCTCCCCGATCCTAGTGCTGCTTCAAGGACCGGACGGCAGCATCTATTGCCACGACGTGTACCGCGGCCGATTGTATCTCGTGGCCCACTCTGTATCGTTGTTCGCCAGG
664157_30677280_HHV-7_JI_U43400.1_144861bp_1_20	CACGACGTGTACCGCGGCCGATTGTATCTCGTGGCCCACTCTGTATCGTTGTTCGCCAGGCTAGGCCTTCGCCACTGCGAACCTTTATATGCGGCACCCAGATGGAAGCACGTTCCTCTG
664157_30677280_HHV-7_JI_U43400.1_144861bp_1_21	CTAGGCCTTCGCCACTGCGAACCTTTATATGCGGCACCCAGATGGAAGCACGTTCCTCTGCCCAACATGTGGGTGGCGAGCCCGCCAGCGTCCGCCACCCTCACGCAAACACTCGCCGTG
664157_30677280_HHV-7_JI_U43400.1_144861bp_1_22	CCCAACATGTGGGTGGCGAGCCCGCCAGCGTCCGCCACCCTCACGCAAACACTCGCCGTGAGTGCCACGCACGGTCTGGACGCGTTATACTCGCTGCTAAAAATCCACAGAGGAACTCCG
664157_30677280_HHV-7_JI_U43400.1_144861bp_1_23	AGTGCCACGCACGGTCTGGACGCGTTATACTCGCTGCTAAAAATCCACAGAGGAACTCCGTGTTCGCTAATCCACCCCGTGAACGGCTACGTCCTGGACATGATACTGACGGGCCGCTCA

664157_30677280_HHV-7_JI_U43400.1_144861bp_1_24	TGTTCGCTAATCCACCCCGTGAACGGCTACGTCCTGGACATGATACTGACGGGCCGCTCATTCCAAGAAGCACCCTGCCAAAACACTCGCACGTCCGTTAAAACAACGCCACATGTAATG
664157_30677280_HHV-7_JI_U43400.1_144861bp_1_25	TTCCAAGAAGCACCCTGCCAAAACACTCGCACGTCCGTTAAAACAACGCCACATGTAATGGACGCAGTCTGCGGTGGCCGCGGGTCATGGCTGTCCATCGGCTACCTAGTAAAGATGCCG
664157_30677280_HHV-7_JI_U43400.1_144861bp_1_26	GACGCAGTCTGCGGTGGCCGCGGGTCATGGCTGTCCATCGGCTACCTAGTAAAGATGCCGCACATTCACCTGGCGGTGACCCGAACATGTCTGGTCACCGCCATAGATGTCCGACAAAAC
664157_30677280_HHV-7_JI_U43400.1_144861bp_1_27	CACATTCACCTGGCGGTGACCCGAACATGTCTGGTCACCGCCATAGATGTCCGACAAAACTTTCTGTGGCGCGTGGCGGACGACGCGCTGCTATTCCTGGTCACCGGTAGTCTTTTACTA
664157_30677280_HHV-7_JI_U43400.1_144861bp_1_28	TTTCTGTGGCGCGTGGCGGACGACGCGCTGCTATTCCTGGTCACCGGTAGTCTTTTACTACTGTCGCGGCCGACCGCAGACTTGACGTCTTGGTCATGTTTACAGCAAGAACCTGTGTGG
664157_30677280_HHV-7_JI_U43400.1_144861bp_1_29	CTGTCGCGGCCGACCGCAGACTTGACGTCTTGGTCATGTTTACAGCAAGAACCTGTGTGGAGGAACTGTCTAGATACGCGCGGAGAACAGGATGAGACAGAAGACCAAGAGATGAAACAA
664157_30677280_HHV-7_JI_U43400.1_144861bp_1_30	AGGAACTGTCTAGATACGCGCGGAGAACAGGATGAGACAGAAGACCAAGAGATGAAACAAAGCACAAGCAAAAAGCAAAATGAGAATAAAAAACTCAACACCTCAAAAAAACACACCCGC
664157_30677280_HHV-7_JI_U43400.1_144861bp_1_31	AGCACAAGCAAAAAGCAAAATGAGAATAAAAAACTCAACACCTCAAAAAAACACACCCGCGTATCGTCGGCAATTCCGACCTTTCCCCTCAGTCTCCGAGAAACGCCGCCAGAAGCCAGG
664157_30677280_HHV-7_JI_U43400.1_144861bp_1_32	GTATCGTCGGCAATTCCGACCTTTCCCCTCAGTCTCCGAGAAACGCCGCCAGAAGCCAGGAGCCCAGCCGTCCTCGCCGCCGCCACCCAGTCTCACAAAACTCGAGCGATCTCGACGCAT
664157_30677280_HHV-7_JI_U43400.1_144861bp_1_33	AGCCCAGCCGTCCTCGCCGCCGCCACCCAGTCTCACAAAACTCGAGCGATCTCGACGCATAATGCCACGACAACAATAAGAATACCGCGCCTTCCCAGTTACCTGCTGGAAGCGCGTCTC
664157_30677280_HHV-7_JI_U43400.1_144861bp_1_34	AATGCCACGACAACAATAAGAATACCGCGCCTTCCCAGTTACCTGCTGGAAGCGCGTCTCTTGTCCGTGACAGCTATCCTGAAAGACACAAAGAAAAAAAAAACCCAGCCTCAGGCGTAG
664157_30677280_HHV-7_JI_U43400.1_144861bp_1_35	TTGTCCGTGACAGCTATCCTGAAAGACACAAAGAAAAAAAAAACCCAGCCTCAGGCGTAGCAGCTGCGACGTCAGCGCGGTGTCTGAAAGCTCGCCAAGGTCTCGCGTAAAAGAACAGAT

664157_30677280_HHV-7_JI_U43400.1_144861bp_1_36	CAGCTGCGACGTCAGCGCGGTGTCTGAAAGCTCGCCAAGGTCTCGCGTAAAAGAACAGATGTGAACTTCAGATGTACCAACCAAATAATACGGGTTCCGCTATAAAAAGTGCACCTCTAT
664157_30677280_HHV-7_JI_U43400.1_144861bp_1_37	GTGAACTTCAGATGTACCAACCAAATAATACGGGTTCCGCTATAAAAAGTGCACCTCTATTCCCGTTCTTATCCCCGTTCTAACTCTTCCTTGTATCATACCTTGCATGTTAACCGGATC
664157_30677280_HHV-7_JI_U43400.1_144861bp_1_38	TCCCGTTCTTATCCCCGTTCTAACTCTTCCTTGTATCATACCTTGCATGTTAACCGGATCCCGTGGATCTTACACACATACACACACACACAAACTTGGTGAGGTAAACACAGAGATCTC
664157_30677280_HHV-7_JI_U43400.1_144861bp_1_39	CCGTGGATCTTACACACATACACACACACACAAACTTGGTGAGGTAAACACAGAGATCTCACTAACTCATAATCCCCTACACGCTTACCACCACCTAAAAATGGTTACAACCAAAACTGG
664157_30677280_HHV-7_JI_U43400.1_144861bp_1_40	ACTAACTCATAATCCCCTACACGCTTACCACCACCTAAAAATGGTTACAACCAAAACTGGCAATAGTCTATCTTCTTTTTCTTTCCATTCACAGCCAATGTGCAGTACTCGTGGGTCCAC
664157_30677280_HHV-7_JI_U43400.1_144861bp_1_41	CAATAGTCTATCTTCTTTTTCTTTCCATTCACAGCCAATGTGCAGTACTCGTGGGTCCACAACAACGAAAGAGACTGTAGAGACAGTTCCTTTAAGTAGACCTTAGAGACACACCAAATA
664157_30677280_HHV-7_JI_U43400.1_144861bp_1_42	AACAACGAAAGAGACTGTAGAGACAGTTCCTTTAAGTAGACCTTAGAGACACACCAAATACAACCACAACCAAAAAAAAAACAGAAAACAACACAAAGCCAATGAGTGCAGAAATGCTCC
664157_30677280_HHV-7_JI_U43400.1_144861bp_1_43	CAACCACAACCAAAAAAAAAACAGAAAACAACACAAAGCCAATGAGTGCAGAAATGCTCCGCGCTGTTCAGCTCCAGCCAAGACGCCGGGGACATTCCTCATCTCCCACTTCCCCTCCAC
664157_30677280_HHV-7_JI_U43400.1_144861bp_1_44	GCGCTGTTCAGCTCCAGCCAAGACGCCGGGGACATTCCTCATCTCCCACTTCCCCTCCACTCGAAGGAGAGCCCAGTCCCAAGAGACTCCAATCGAGCGACAGTCACCAAGGGCGTAGAG
664157_30677280_HHV-7_JI_U43400.1_144861bp_1_45	TCGAAGGAGAGCCCAGTCCCAAGAGACTCCAATCGAGCGACAGTCACCAAGGGCGTAGAGGCAGACCTAAACCCAGAGCTAAAACATGGAGCGAAGCTTTATCCCACCGGTCCTTCCTCA
664157_30677280_HHV-7_JI_U43400.1_144861bp_1_46	GCAGACCTAAACCCAGAGCTAAAACATGGAGCGAAGCTTTATCCCACCGGTCCTTCCTCAACATTTACGCGTGGCTGTCTTTGAGTCGAGGGTCTCCGCGAAAAGTGTACGGATATGCCT
664157_30677280_HHV-7_JI_U43400.1_144861bp_1_47	ACATTTACGCGTGGCTGTCTTTGAGTCGAGGGTCTCCGCGAAAAGTGTACGGATATGCCTTCAGGCACAGAGGAGAACTCGTAGCATTGCCATGGCCGCCTAACTGGAGCCTGGAACTTC

664157_30677280_HHV-7_JI_U43400.1_144861bp_1_48	TCAGGCACAGAGGAGAACTCGTAGCATTGCCATGGCCGCCTAACTGGAGCCTGGAACTTCACCACGATCCCTATCGAGACGCCAGAGCACAAACCGTTTGGAGTCACCGCTGGGGATGGC
664157_30677280_HHV-7_JI_U43400.1_144861bp_1_49	ACCACGATCCCTATCGAGACGCCAGAGCACAAACCGTTTGGAGTCACCGCTGGGGATGGCCTGCAACACACGTGACAGCTCGCACGGTGCGGGACTGCGGTGAGTGTAAGCAGTGTGACA
664157_30677280_HHV-7_JI_U43400.1_144861bp_1_50	CTGCAACACACGTGACAGCTCGCACGGTGCGGGACTGCGGTGAGTGTAAGCAGTGTGACACATTGTTATCGCAATTGTCTTACCCGATTAACTTTTTATTAATGTATTAAGCACTCTTTC
664157_30677280_HHV-7_JI_U43400.1_144861bp_1_51	CATTGTTATCGCAATTGTCTTACCCGATTAACTTTTTATTAATGTATTAAGCACTCTTTCTTCACGTGTGACTGTTGTGTTTTTTGTTGTTATCTACATCCCGGCAGCCCTCGACACGCA
664157_30677280_HHV-7_JI_U43400.1_144861bp_1_52	TTCACGTGTGACTGTTGTGTTTTTTGTTGTTATCTACATCCCGGCAGCCCTCGACACGCATATGTACGTGTGCTGCGGACGCGGAGAAAAGTTGCAGCCCGTCGGATACGTACGCAACAG
664157_30677280_HHV-7_JI_U43400.1_144861bp_1_53	TATGTACGTGTGCTGCGGACGCGGAGAAAAGTTGCAGCCCGTCGGATACGTACGCAACAGAGCCGCGCCTTCAGACCTGAACTCGTTACGCGTCCTCCTCATAGCCAGGGACGGAGCAAT
664157_30677280_HHV-7_JI_U43400.1_144861bp_1_54	AGCCGCGCCTTCAGACCTGAACTCGTTACGCGTCCTCCTCATAGCCAGGGACGGAGCAATGTATGTGCATCACATGAGAACGGCGCGACTGTGCCGCCTGGCCAGCAGTGTGACCGAATT
664157_30677280_HHV-7_JI_U43400.1_144861bp_1_55	GTATGTGCATCACATGAGAACGGCGCGACTGTGCCGCCTGGCCAGCAGTGTGACCGAATTCGCGCGACGAGGGCTGCAGCGAGAATCCGAGGTTTATGAAGATGATGTTTCCTTGCCAGA
664157_30677280_HHV-7_JI_U43400.1_144861bp_1_56	CGCGCGACGAGGGCTGCAGCGAGAATCCGAGGTTTATGAAGATGATGTTTCCTTGCCAGACCGTCGAGTAGGTTCGGCAACGGCCATTCACCTGTTTGACGTAATTACCCAGGCAGCCGA
664157_30677280_HHV-7_JI_U43400.1_144861bp_1_57	CCGTCGAGTAGGTTCGGCAACGGCCATTCACCTGTTTGACGTAATTACCCAGGCAGCCGATGTCCACGACCTACTCACCGTGGCCGGACTGTGTCAGACTCACACCGGCGTCAGCTGCCA
664157_30677280_HHV-7_JI_U43400.1_144861bp_1_58	TGTCCACGACCTACTCACCGTGGCCGGACTGTGTCAGACTCACACCGGCGTCAGCTGCCAACTGTGGTATACAGACCACGATCCCCACACCGTCGCTGGGGCGGCACGCTTCACACTGAC
664157_30677280_HHV-7_JI_U43400.1_144861bp_1_59	ACTGTGGTATACAGACCACGATCCCCACACCGTCGCTGGGGCGGCACGCTTCACACTGACGGTCGCACGGCAGCAGTATCGATTGTGGCCAAACGCACGACGCAAACTGCTGCAGCACCT

664157_30677280_HHV-7_JI_U43400.1_144861bp_1_60	GGTCGCACGGCAGCAGTATCGATTGTGGCCAAACGCACGACGCAAACTGCTGCAGCACCTACATCCGGACCACCCACTTGGGCTGTGGCTGTTGTGTGCCGTGCTCACGTACGATGCAAA
664157_30677280_HHV-7_JI_U43400.1_144861bp_1_61	ACATCCGGACCACCCACTTGGGCTGTGGCTGTTGTGTGCCGTGCTCACGTACGATGCAAAAGAGACGAATCGCGCAGTGCCACCCGTAACGCCAGGGGCCGAAACCGTGTGGGTGATAGT
664157_30677280_HHV-7_JI_U43400.1_144861bp_1_62	AGAGACGAATCGCGCAGTGCCACCCGTAACGCCAGGGGCCGAAACCGTGTGGGTGATAGTTACTGGCAGGGGTGCCATTCTAGGATTCTGGCCAGAGAGCGCCAAAATGTGCAGATTGGC
664157_30677280_HHV-7_JI_U43400.1_144861bp_1_63	TACTGGCAGGGGTGCCATTCTAGGATTCTGGCCAGAGAGCGCCAAAATGTGCAGATTGGCCTCGTCTATGAAAGGACTCTGGAAAAACGGAGCGCGGGCGCTAAAAGGTCACTGGACATA
664157_30677280_HHV-7_JI_U43400.1_144861bp_1_64	CTCGTCTATGAAAGGACTCTGGAAAAACGGAGCGCGGGCGCTAAAAGGTCACTGGACATACGCAGCACCCGGCCGGCATAGAGCGGGAGAGGCCTGGCCTTTGTGTGCACACTACCAATC
664157_30677280_HHV-7_JI_U43400.1_144861bp_1_65	CGCAGCACCCGGCCGGCATAGAGCGGGAGAGGCCTGGCCTTTGTGTGCACACTACCAATCTCCTAGATAGAACAAAATTAAAAAGATTAAAAAAAAAAGAAAAAAAGTACAAGAGTGTTA
664157_30677280_HHV-7_JI_U43400.1_144861bp_1_66	TCCTAGATAGAACAAAATTAAAAAGATTAAAAAAAAAAGAAAAAAAGTACAAGAGTGTTATCGCGAAACAGCGTGTCAAAAAAAAAACAATCCACATACTCTAGAACAAACTGTACCCAA
664157_30677280_HHV-7_JI_U43400.1_144861bp_1_67	TCGCGAAACAGCGTGTCAAAAAAAAAACAATCCACATACTCTAGAACAAACTGTACCCAAAAATAAGTCCGTGTGCAAAACTGGGAAAAAAAAATCACCTTCCTCGTTGCCACTAGAGGG
664157_30677280_HHV-7_JI_U43400.1_144861bp_1_68	AAATAAGTCCGTGTGCAAAACTGGGAAAAAAAAATCACCTTCCTCGTTGCCACTAGAGGGAGTACCGAAAGTGTAGGCAAGAAGGCCACGCTGTAAATGACTGTCAGCGTTTGGCGCTGA
664157_30677280_HHV-7_JI_U43400.1_144861bp_1_69	AGTACCGAAAGTGTAGGCAAGAAGGCCACGCTGTAAATGACTGTCAGCGTTTGGCGCTGAAAACATTGCTGTTCTTGCTGGCTCAAGCACAATCACGTGATTAAGATTCCTTTCGTTTTC
664157_30677280_HHV-7_JI_U43400.1_144861bp_1_70	AAACATTGCTGTTCTTGCTGGCTCAAGCACAATCACGTGATTAAGATTCCTTTCGTTTTCAAAGTGTGCCCGGGAGGCAGACATGCCCTTTCTCGTGAGACATTATGAGATTTGCCTGCC
664157_30677280_HHV-7_JI_U43400.1_144861bp_1_71	AAAGTGTGCCCGGGAGGCAGACATGCCCTTTCTCGTGAGACATTATGAGATTTGCCTGCCAGAGAACCACGTGACTTGGACTTACTTTCGTTTTCTAAACGTGCCCTCTAGGCATGAATG

664157_30677280_HHV-7_JI_U43400.1_144861bp_1_72	AGAGAACCACGTGACTTGGACTTACTTTCGTTTTCTAAACGTGCCCTCTAGGCATGAATGCTCTTTAGCGTTAGCCATGAGGCTAGCGTGATCCTGTATAGTACATAAGTTTCTAAGAAT
664157_30677280_HHV-7_JI_U43400.1_144861bp_1_73	CTCTTTAGCGTTAGCCATGAGGCTAGCGTGATCCTGTATAGTACATAAGTTTCTAAGAATATGTTTTTAACAATAATCATGTCCCAAAAAGTCGCGAGTGACTAAAATTCTCTGTAAATG
664157_30677280_HHV-7_JI_U43400.1_144861bp_1_74	ATGTTTTTAACAATAATCATGTCCCAAAAAGTCGCGAGTGACTAAAATTCTCTGTAAATGAAGGCAAATTAAACAGGATACAGACAGTTGTGGCAGTGGTCCGTTTCGTCTTTCTGTGTT
664157_30677280_HHV-7_JI_U43400.1_144861bp_1_75	AAGGCAAATTAAACAGGATACAGACAGTTGTGGCAGTGGTCCGTTTCGTCTTTCTGTGTTTTCCTTACGCGGCTGACGAGGTAAAGTGTCTCAGTCCATATTGTTGTCTGTGCCACCGTA
664157_30677280_HHV-7_JI_U43400.1_144861bp_1_76	TTCCTTACGCGGCTGACGAGGTAAAGTGTCTCAGTCCATATTGTTGTCTGTGCCACCGTAGTTAGCGGTGGCATACTAAAAACTCCGATAGATGCAGAACAATAACACCGAAAACCACGC
664157_30677280_HHV-7_JI_U43400.1_144861bp_1_77	GTTAGCGGTGGCATACTAAAAACTCCGATAGATGCAGAACAATAACACCGAAAACCACGCTGTGGAACCAGACCACACTTTATAAACAAAACGGCCTTATCACCTGGAAAAAAAACTAAA
664157_30677280_HHV-7_JI_U43400.1_144861bp_1_78	TGTGGAACCAGACCACACTTTATAAACAAAACGGCCTTATCACCTGGAAAAAAAACTAAAAATAAGGCAATGATACACCTGACTTTCCATTGGAAACCTGCCGTAACCCTGACCACAAAT
664157_30677280_HHV-7_JI_U43400.1_144861bp_1_79	AATAAGGCAATGATACACCTGACTTTCCATTGGAAACCTGCCGTAACCCTGACCACAAATCCCATGCTAAATCCCCTGAAACACTGCCAAACGTCGCTACAAGGTTTTTCCGGGATCGAG
664157_30677280_HHV-7_JI_U43400.1_144861bp_1_80	CCCATGCTAAATCCCCTGAAACACTGCCAAACGTCGCTACAAGGTTTTTCCGGGATCGAGCCGCAGCAAGCTTAAACTGAGGTCACACACGACTTTAATTACGGCAACGCACAGCTGTAA
664157_30677280_HHV-7_JI_U43400.1_144861bp_1_81	CCGCAGCAAGCTTAAACTGAGGTCACACACGACTTTAATTACGGCAACGCACAGCTGTAAGCTGCAGGAAAGATACGATCGTAAGCAAATGTAGTCCTACAATCAAGCGAGGTTGTAGAC
664157_30677280_HHV-7_JI_U43400.1_144861bp_1_82	GCTGCAGGAAAGATACGATCGTAAGCAAATGTAGTCCTACAATCAAGCGAGGTTGTAGACGTTACCTACAATGAACTACACCTCTAAGCATAACCTGTCGGGCACAGTGAGACACGCAGC
664157_30677280_HHV-7_JI_U43400.1_144861bp_1_83	GTTACCTACAATGAACTACACCTCTAAGCATAACCTGTCGGGCACAGTGAGACACGCAGCCGTAAATTCAAAACTCAACCCAAACCGAAGTCTAAGTCTCACCCTAATCGTAACAGTAAC

664157_30677280_HHV-7_JI_U43400.1_144861bp_1_84	CGTAAATTCAAAACTCAACCCAAACCGAAGTCTAAGTCTCACCCTAATCGTAACAGTAACCCTACAACTCTAATCCTAGTCCGTAACCGTAACCCCAATCCTAGCCCTTAGCCCTAACCC
664157_30677280_HHV-7_JI_U43400.1_144861bp_1_85	CCTACAACTCTAATCCTAGTCCGTAACCGTAACCCCAATCCTAGCCCTTAGCCCTAACCCTAGCCCTAACCCTAGCTCTAACCTTAGCTCTAACTCTGACCCTAGGCCTAACCCTAAGCC
664157_30677280_HHV-7_JI_U43400.1_144861bp_1_86	TAGCCCTAACCCTAGCTCTAACCTTAGCTCTAACTCTGACCCTAGGCCTAACCCTAAGCCTAACCCTAACCGTAGCTCTAAGTTTAACCCTAACCCTAACCCTAACCATGACCCTGACCC
664157_30677280_HHV-7_JI_U43400.1_144861bp_1_87	TAACCCTAACCGTAGCTCTAAGTTTAACCCTAACCCTAACCCTAACCATGACCCTGACCCTAACCCTAGGCTGCGGCCCTAACCCTAGCCCTAACCCTAACCCTAATCCTAATCCTAGCC
664157_30677280_HHV-7_JI_U43400.1_144861bp_1_88	TAACCCTAGGCTGCGGCCCTAACCCTAGCCCTAACCCTAACCCTAATCCTAATCCTAGCCCTAACCCTAGGGCTGCGGCCCTAACCCTAGCCCTAACCCTAACCCTAACCCTAGGGCTGC
664157_30677280_HHV-7_JI_U43400.1_144861bp_1_89	CTAACCCTAGGGCTGCGGCCCTAACCCTAGCCCTAACCCTAACCCTAACCCTAGGGCTGCGGCCCTAACCCTAACCCTAGGGCTGCGGCCCGAACCCTAACCCTAACCCTAACCCTAACC
664157_30677280_HHV-7_JI_U43400.1_144861bp_1_90	GGCCCTAACCCTAACCCTAGGGCTGCGGCCCGAACCCTAACCCTAACCCTAACCCTAACCCTAGGGCTGCGGCCCTAACCCTAACCCTAGGGCTGCGGCCCTAACCCTAACCCTAGGGCT
664157_30677280_HHV-7_JI_U43400.1_144861bp_1_91	CTAGGGCTGCGGCCCTAACCCTAACCCTAGGGCTGCGGCCCTAACCCTAACCCTAGGGCTGCGGCCCGAACCCTAACCCTAACCCTAACCCTAGGGCTGCGGCCCTAACCCTAACCCTAG
664157_30677280_HHV-7_JI_U43400.1_144861bp_1_92	GCGGCCCGAACCCTAACCCTAACCCTAACCCTAGGGCTGCGGCCCTAACCCTAACCCTAGGGCTGCGGCCCTAACCCTAACCCTAACTCTAGGGCTGCGGCCCTAACCCTAACCCTAACC
664157_30677280_HHV-7_JI_U43400.1_144861bp_1_93	GGCTGCGGCCCTAACCCTAACCCTAACTCTAGGGCTGCGGCCCTAACCCTAACCCTAACCCTAACCCTAGGGCTGCGGCCCGAACCCTAGCCCTAACCCTAACCCTGACCCTGACCCTAA
664157_30677280_HHV-7_JI_U43400.1_144861bp_1_96	CCCTAACCCTAACCCTAACCCTAACCCTAACCCCGCCCCCACTGGCAGCCAATGTCTTGTAATGCCTTCAAGGCACTTTTTCTGCGAGCCGCGCGCAGCACTCAGTGAAAAACAAGTTTG
664157_30677280_HHV-7_JI_U43400.1_144861bp_1_97	AATGCCTTCAAGGCACTTTTTCTGCGAGCCGCGCGCAGCACTCAGTGAAAAACAAGTTTGTGCACGAGAAAGACGCTGCCAAACCGCAGCTGCAGCATGAAGGCTGAGTGCACAATTTTG

664157_30677280_HHV-7_JI_U43400.1_144861bp_1_98	TGCACGAGAAAGACGCTGCCAAACCGCAGCTGCAGCATGAAGGCTGAGTGCACAATTTTGGCTTTAGTCCCATAAAGGCGCGGCTTCCCGTAGAGTAGAAAACCGCAGCGCGGCGCACAG
664157_30677280_HHV-7_JI_U43400.1_144861bp_1_99	GCTTTAGTCCCATAAAGGCGCGGCTTCCCGTAGAGTAGAAAACCGCAGCGCGGCGCACAGAGCGAAGGCAGCGGCTTTCAGACTGTTTGCCAAGCGCAGTCTGCATCTTACCAATGATGA
664157_30677280_HHV-7_JI_U43400.1_144861bp_1_100	AGCGAAGGCAGCGGCTTTCAGACTGTTTGCCAAGCGCAGTCTGCATCTTACCAATGATGATCGCAAGCAAGAAAAATGTTCTTTCTTAGCATATGCGTGGTTAATCCTGTTGTGGTCATC
664157_30677280_HHV-7_JI_U43400.1_144861bp_1_101	TCGCAAGCAAGAAAAATGTTCTTTCTTAGCATATGCGTGGTTAATCCTGTTGTGGTCATCACTAAGTTTTCAAGCTTTTGGCAAGGCATGAAAAATAACATTACTATTGGACTGTTTATA
664157_30677280_HHV-7_JI_U43400.1_144861bp_1_102	ACTAAGTTTTCAAGCTTTTGGCAAGGCATGAAAAATAACATTACTATTGGACTGTTTATACTTATCTTCAAATGTTCACTCACAGCAGCGAAGGGGACACTAGAAAACACTCCCAAGTAG
664157_30677280_HHV-7_JI_U43400.1_144861bp_1_103	CTTATCTTCAAATGTTCACTCACAGCAGCGAAGGGGACACTAGAAAACACTCCCAAGTAGAACTACGAGGCGGAACAGCAATGGAAACTCAGACGTGTTTTTACTTTTATTTTCTGAAAT
664157_30677280_HHV-7_JI_U43400.1_144861bp_1_104	AACTACGAGGCGGAACAGCAATGGAAACTCAGACGTGTTTTTACTTTTATTTTCTGAAATCATTTAATAGCATACAAGTACGGCCTCTCCGAATCGAAGCTAATCTGAGAGCAAAGACAA
664157_30677280_HHV-7_JI_U43400.1_144861bp_1_105	CATTTAATAGCATACAAGTACGGCCTCTCCGAATCGAAGCTAATCTGAGAGCAAAGACAAAGAGCACTGTAAGACTGAAGGCAAAAAAACCCCCCGCTTAAAAAAGAATTTCATAATTCC
664157_30677280_HHV-7_JI_U43400.1_144861bp_1_106	AGAGCACTGTAAGACTGAAGGCAAAAAAACCCCCCGCTTAAAAAAGAATTTCATAATTCCGCAGCTCTTCCGAAGCCCCCCGAGAACAACACAGATCGCTAAGGGTGTCTGGACTGAGCT
664157_30677280_HHV-7_JI_U43400.1_144861bp_1_107	GCAGCTCTTCCGAAGCCCCCCGAGAACAACACAGATCGCTAAGGGTGTCTGGACTGAGCTCAGTATCCCGGTAAGCCTCCAGAGCAGATGGACATAAACTATATTCTCGTGGTAATTGAA
664157_30677280_HHV-7_JI_U43400.1_144861bp_1_108	CAGTATCCCGGTAAGCCTCCAGAGCAGATGGACATAAACTATATTCTCGTGGTAATTGAACTACCGGAACGTGGATGCAAGTGACGGGTAACTGCATGGCCCAAGCGTTTCTATCAGGAA
664157_30677280_HHV-7_JI_U43400.1_144861bp_1_109	CTACCGGAACGTGGATGCAAGTGACGGGTAACTGCATGGCCCAAGCGTTTCTATCAGGAAAGCGATAGTTTTTAAAGAGTCTCCTGGCGCCCATGGCCAAAAAAACCCTAAAACTACAAG

664157_30677280_HHV-7_JI_U43400.1_144861bp_1_110	AGCGATAGTTTTTAAAGAGTCTCCTGGCGCCCATGGCCAAAAAAACCCTAAAACTACAAGCCAAGCTAACGACGGCTCCTCCGCGAACATCCAGAGCGTAGACAGCACCCAAGCGATCCA
664157_30677280_HHV-7_JI_U43400.1_144861bp_1_111	CCAAGCTAACGACGGCTCCTCCGCGAACATCCAGAGCGTAGACAGCACCCAAGCGATCCATAAGAACCCGTTGCTTTCGAAGAAGACCACGACCGAGGCATCTTCCGACAAACTTTCCGA
664157_30677280_HHV-7_JI_U43400.1_144861bp_1_112	TAAGAACCCGTTGCTTTCGAAGAAGACCACGACCGAGGCATCTTCCGACAAACTTTCCGATAACCACGGCAACGCCACGCCTAGGAATGAACCTCCACCGCTCTCCATGGTCCTCCGGGA
664157_30677280_HHV-7_JI_U43400.1_144861bp_1_113	TAACCACGGCAACGCCACGCCTAGGAATGAACCTCCACCGCTCTCCATGGTCCTCCGGGACGGAAAATTCGAACAGCACGCCTTTAGGCCACGGAAGAGGCACAAGAGCCTTGTCGAAAG
664157_30677280_HHV-7_JI_U43400.1_144861bp_1_114	CGGAAAATTCGAACAGCACGCCTTTAGGCCACGGAAGAGGCACAAGAGCCTTGTCGAAAGCAACAGCGAAATTCAAAACATCCTCCACCCCGCACATAGGGCCGAAGAAAAAACCCCCCG
664157_30677280_HHV-7_JI_U43400.1_144861bp_1_115	CAACAGCGAAATTCAAAACATCCTCCACCCCGCACATAGGGCCGAAGAAAAAACCCCCCGAAACGATGGACTCGAAAAAAGCGTACACAGGGTCAAATCTGCGCAGACCACGCTTCCAGA
664157_30677280_HHV-7_JI_U43400.1_144861bp_1_116	AAACGATGGACTCGAAAAAAGCGTACACAGGGTCAAATCTGCGCAGACCACGCTTCCAGAGACCATAAGGGGTGTCCGCAACTCTGTAAATGGCGTTGTCCACGGAACGGTTAAATGCAT
664157_30677280_HHV-7_JI_U43400.1_144861bp_1_117	GACCATAAGGGGTGTCCGCAACTCTGTAAATGGCGTTGTCCACGGAACGGTTAAATGCATAAAATTCACCCCGATCTCCAATAAGCATCAAAGCAGATAAATCAGAATAGAGCACCAGAC
664157_30677280_HHV-7_JI_U43400.1_144861bp_1_118	AAAATTCACCCCGATCTCCAATAAGCATCAAAGCAGATAAATCAGAATAGAGCACCAGACCGATGACCCAAACATCCACAAAAGGACAACAGACAAAATTTCTGGTTAACTTGCAGACTT
664157_30677280_HHV-7_JI_U43400.1_144861bp_1_119	CGATGACCCAAACATCCACAAAAGGACAACAGACAAAATTTCTGGTTAACTTGCAGACTTCGTCTTCCGAAATGCCAAGCATCTCCGAAGAGGCAAACCTCAACTCAGCGCCGGAAGGCC
664157_30677280_HHV-7_JI_U43400.1_144861bp_1_120	CGTCTTCCGAAATGCCAAGCATCTCCGAAGAGGCAAACCTCAACTCAGCGCCGGAAGGCCACACGAGAGGAAGTCGATGGTGAGCATATTGCAAAACAAAATGAGCAACGTCTCCGAAAG
664157_30677280_HHV-7_JI_U43400.1_144861bp_1_121	ACACGAGAGGAAGTCGATGGTGAGCATATTGCAAAACAAAATGAGCAACGTCTCCGAAAGATACGGTTTCCGCAGCGAAGCTCTGCAAAAACGTGGTCCAGTAGACCTCCGGAACAGGTG

664157_30677280_HHV-7_JI_U43400.1_144861bp_1_122	ATACGGTTTCCGCAGCGAAGCTCTGCAAAAACGTGGTCCAGTAGACCTCCGGAACAGGTGATTCAACTGTATTCGATTCCAGATTAAACAAACCGTTCGATGCATTCGACTCAACAGCCG
664157_30677280_HHV-7_JI_U43400.1_144861bp_1_123	ATTCAACTGTATTCGATTCCAGATTAAACAAACCGTTCGATGCATTCGACTCAACAGCCGTTTCCACACCGGAATCCAAAAGCGGAGAATCTGACATACTAGCAACATGCAAATGATCAG
664157_30677280_HHV-7_JI_U43400.1_144861bp_1_124	TTTCCACACCGGAATCCAAAAGCGGAGAATCTGACATACTAGCAACATGCAAATGATCAGAGAAAAAAAACAAGTGAAATGCACAGAGACAAATACAATCAAACTCATACTCTAAAGCAC
664157_30677280_HHV-7_JI_U43400.1_144861bp_1_125	AGAAAAAAAACAAGTGAAATGCACAGAGACAAATACAATCAAACTCATACTCTAAAGCACGAACGCTGTTTATTAATTACCATGTCAATTTCAAAACCCACCAGCAAACAAGGCAATAAA
664157_30677280_HHV-7_JI_U43400.1_144861bp_1_126	GAACGCTGTTTATTAATTACCATGTCAATTTCAAAACCCACCAGCAAACAAGGCAATAAAAAAAAAGAGTCACAAACTTAGCAACGAAAAATCTGGGTTCCAACATCAGGAAACGCTGCC
664157_30677280_HHV-7_JI_U43400.1_144861bp_1_127	AAAAAAGAGTCACAAACTTAGCAACGAAAAATCTGGGTTCCAACATCAGGAAACGCTGCCCGCTCATCTGCTCTTGCAGCCAAGAAGAGAAGGTCACAGTCGCCCTGTCTGGCAGTTTTC
664157_30677280_HHV-7_JI_U43400.1_144861bp_1_128	CGCTCATCTGCTCTTGCAGCCAAGAAGAGAAGGTCACAGTCGCCCTGTCTGGCAGTTTTCAGATAAAAGCGCGACGAAAAATGACACAATGGTTTCTGGCACCATTTGGGCTGAGCCGCG
664157_30677280_HHV-7_JI_U43400.1_144861bp_1_129	AGATAAAAGCGCGACGAAAAATGACACAATGGTTTCTGGCACCATTTGGGCTGAGCCGCGTAAAAAGTGTGATGTTTATAGAAGTTACGCACACCGACAAACCTAAAAACCTGAAAACTC
664157_30677280_HHV-7_JI_U43400.1_144861bp_1_130	TAAAAAGTGTGATGTTTATAGAAGTTACGCACACCGACAAACCTAAAAACCTGAAAACTCTCTGCGATGAAAGTCAAGTTTCCGTCGGGATCGTTATCATTAAACCCATAAATTTTTCCC
664157_30677280_HHV-7_JI_U43400.1_144861bp_1_131	TCTGCGATGAAAGTCAAGTTTCCGTCGGGATCGTTATCATTAAACCCATAAATTTTTCCCGAGCAGTCAGCGTAAAGGGAAATTCTAGCTCCGTCTGAAAAACATTTCAGTCCGAAGCTC
664157_30677280_HHV-7_JI_U43400.1_144861bp_1_132	GAGCAGTCAGCGTAAAGGGAAATTCTAGCTCCGTCTGAAAAACATTTCAGTCCGAAGCTCGCAAAGAAACACATATCAGCTAAAACACTTTTTATCTCCGGGTCAGAAACAAAATGAAAA
664157_30677280_HHV-7_JI_U43400.1_144861bp_1_133	GCAAAGAAACACATATCAGCTAAAACACTTTTTATCTCCGGGTCAGAAACAAAATGAAAACTGTCTTTCCTGTGATGAAACAGGATATTCTCTCTCGCCGGCCATCGCAACTCAAATTTT

664157_30677280_HHV-7_JI_U43400.1_144861bp_1_134	CTGTCTTTCCTGTGATGAAACAGGATATTCTCTCTCGCCGGCCATCGCAACTCAAATTTTTCACCAAGATGCCTGTCCCGAAATGCCAAAATTTCAGCGCATGTTGTGATAGCTTCAATC
664157_30677280_HHV-7_JI_U43400.1_144861bp_1_135	TCACCAAGATGCCTGTCCCGAAATGCCAAAATTTCAGCGCATGTTGTGATAGCTTCAATCTCTGGATCTGATACCTCAAATTGCAGACACTTACACAAAACGTATGCGTACAGACATCTT
664157_30677280_HHV-7_JI_U43400.1_144861bp_1_136	TCTGGATCTGATACCTCAAATTGCAGACACTTACACAAAACGTATGCGTACAGACATCTTTTGAGACCTTTCCTCAAAAAACTGTCAATAGTGTCACCCACGCGACATAGTTGATCAGGA
664157_30677280_HHV-7_JI_U43400.1_144861bp_1_137	TTGAGACCTTTCCTCAAAAAACTGTCAATAGTGTCACCCACGCGACATAGTTGATCAGGAAATAACGTTGTATGTGCATAAACGCGGCCAACTTTCCCAAGTAAGATAACCACATCATCA
664157_30677280_HHV-7_JI_U43400.1_144861bp_1_138	AATAACGTTGTATGTGCATAAACGCGGCCAACTTTCCCAAGTAAGATAACCACATCATCAGTCTCATACTCAAGTTCGTAAACAGTCGTCACAGTATGTTTTATGTAACCAAGAACAGTA
664157_30677280_HHV-7_JI_U43400.1_144861bp_1_139	GTCTCATACTCAAGTTCGTAAACAGTCGTCACAGTATGTTTTATGTAACCAAGAACAGTAAGTTCTTCAGGACAACACACCAACGCGGCGTACTTTTTGAGCTTATCTTCCTCATATCCC
664157_30677280_HHV-7_JI_U43400.1_144861bp_1_140	AGTTCTTCAGGACAACACACCAACGCGGCGTACTTTTTGAGCTTATCTTCCTCATATCCCTCCACACCATAAGTCAAAACAAGTTCAAACCCCTCTGGAAAAACCAAAGGCACCCTCAAA
664157_30677280_HHV-7_JI_U43400.1_144861bp_1_141	TCCACACCATAAGTCAAAACAAGTTCAAACCCCTCTGGAAAAACCAAAGGCACCCTCAAATGCGGATTCAGAAACAAAAACATTTTTAGACTATTAAAATTTGTATAAACACGTAGAAAA
664157_30677280_HHV-7_JI_U43400.1_144861bp_1_142	TGCGGATTCAGAAACAAAAACATTTTTAGACTATTAAAATTTGTATAAACACGTAGAAAATCCACGAGTTTAGCCTTAGCAGATTCAAAGATTTTCTCTTTTCTTTTTTTATCAGATAAA
664157_30677280_HHV-7_JI_U43400.1_144861bp_1_143	TCCACGAGTTTAGCCTTAGCAGATTCAAAGATTTTCTCTTTTCTTTTTTTATCAGATAAAAACGTTTTCTCAACGTTGGCAACCTTCAAATCCTTTTTCCCGTCAACATGAGCAACATAT
664157_30677280_HHV-7_JI_U43400.1_144861bp_1_144	AACGTTTTCTCAACGTTGGCAACCTTCAAATCCTTTTTCCCGTCAACATGAGCAACATATTTTCGTATAAACGAATTTTCCTTTTTTAAATCTTTTGCCACAACTCTCAAAACACGCTCT
664157_30677280_HHV-7_JI_U43400.1_144861bp_1_145	TTTCGTATAAACGAATTTTCCTTTTTTAAATCTTTTGCCACAACTCTCAAAACACGCTCTTCAAAATTGAAGTCAGACCCCAAATCTGCCATACTTAAATCGTACCTAGCGTATTAATAA

664157_30677280_HHV-7_JI_U43400.1_144861bp_1_146	TCAAAATTGAAGTCAGACCCCAAATCTGCCATACTTAAATCGTACCTAGCGTATTAATAATCTTTGTGCACCGGTTTCGCAAAAGGGCAGAAAGCCAACCGCGGAATGCGACGCGACTGG
664157_30677280_HHV-7_JI_U43400.1_144861bp_1_147	TCTTTGTGCACCGGTTTCGCAAAAGGGCAGAAAGCCAACCGCGGAATGCGACGCGACTGGCGAATGGTAAATCCGCGGTGCTGAGACTCTTTAAAAAGCCTTTTCCTCACCGAAACTCTC
664157_30677280_HHV-7_JI_U43400.1_144861bp_1_148	CGAATGGTAAATCCGCGGTGCTGAGACTCTTTAAAAAGCCTTTTCCTCACCGAAACTCTCCTTTGTCGCATCCTGTGTTCAAAGATCCTAACCTCAAAAGAACCTCCACCCCTAACCGCA
664157_30677280_HHV-7_JI_U43400.1_144861bp_1_149	CTTTGTCGCATCCTGTGTTCAAAGATCCTAACCTCAAAAGAACCTCCACCCCTAACCGCATGCGGCACTACGTTTGGAAACAAACCACTCCAAAGATGCGTCCCATATAAATTGCAATCT
664157_30677280_HHV-7_JI_U43400.1_144861bp_1_150	TGCGGCACTACGTTTGGAAACAAACCACTCCAAAGATGCGTCCCATATAAATTGCAATCTTGACACCGAAAAAAACGCACTGGATTGTCCATGTAGTGCAGCTTTAAAGGATTCGTAAAT
664157_30677280_HHV-7_JI_U43400.1_144861bp_1_151	TGACACCGAAAAAAACGCACTGGATTGTCCATGTAGTGCAGCTTTAAAGGATTCGTAAATCCTTCCAAAATATCGTGCCTTAACTCCCGAGCTAAGTGTAAAAGAAAGCACGAACAAGTT
664157_30677280_HHV-7_JI_U43400.1_144861bp_1_152	CCTTCCAAAATATCGTGCCTTAACTCCCGAGCTAAGTGTAAAAGAAAGCACGAACAAGTTAAAGCTACCTTCTTTTGACACAAGTGCTCTAGGAGAGCTCTCACTCTATGATTCTGTTTA
664157_30677280_HHV-7_JI_U43400.1_144861bp_1_153	AAAGCTACCTTCTTTTGACACAAGTGCTCTAGGAGAGCTCTCACTCTATGATTCTGTTTACTGTAGGAAGCTACTAGAATAGAAGCCACTTCAAGAAAAGCGGAACAGTGCCGAGGAATG
664157_30677280_HHV-7_JI_U43400.1_144861bp_1_154	CTGTAGGAAGCTACTAGAATAGAAGCCACTTCAAGAAAAGCGGAACAGTGCCGAGGAATGGACATCACATTGTGACTGCTAAAGTGACTACCTAAAACTGCTAACTTCCTACAAACGTTT
664157_30677280_HHV-7_JI_U43400.1_144861bp_1_155	GACATCACATTGTGACTGCTAAAGTGACTACCTAAAACTGCTAACTTCCTACAAACGTTTTCAACTCCTTCAAAAGGTAGTATCCTTATTTCTAAAAAGACTAAAGACTGCGCGAAAAAA
664157_30677280_HHV-7_JI_U43400.1_144861bp_1_156	TCAACTCCTTCAAAAGGTAGTATCCTTATTTCTAAAAAGACTAAAGACTGCGCGAAAAAACGCGCTATTTTGTCCAAATCACGAGAGATGCGTTCTCTTACTCTGCGCGCCAGGAAAAGA
664157_30677280_HHV-7_JI_U43400.1_144861bp_1_157	CGCGCTATTTTGTCCAAATCACGAGAGATGCGTTCTCTTACTCTGCGCGCCAGGAAAAGACATAAAATGATATTACAATAAATCTTTAAAATGTTTCTTTCTAGATGACTATTCCCTAAA

664157_30677280_HHV-7_JI_U43400.1_144861bp_1_158	CATAAAATGATATTACAATAAATCTTTAAAATGTTTCTTTCTAGATGACTATTCCCTAAAATTTCATCATGTAGAATTGGCAATGTTTTCGATTCCATTGCTTTCAGTATTAACTCCGCG
664157_30677280_HHV-7_JI_U43400.1_144861bp_1_159	ATTTCATCATGTAGAATTGGCAATGTTTTCGATTCCATTGCTTTCAGTATTAACTCCGCGCCGAGATCTTTGTCAATACTCTCTCTAACGCGTAAAGCTTCATCATTAGTTAAATGTAAT
664157_30677280_HHV-7_JI_U43400.1_144861bp_1_160	CCGAGATCTTTGTCAATACTCTCTCTAACGCGTAAAGCTTCATCATTAGTTAAATGTAATTGCTGGAGAATCAAGTCCTGTGGACTAAAGTCCTGTATATCGGACAAATGTAGGACGCCC
664157_30677280_HHV-7_JI_U43400.1_144861bp_1_161	TGCTGGAGAATCAAGTCCTGTGGACTAAAGTCCTGTATATCGGACAAATGTAGGACGCCCCTTTCTCTCCGCTTCAAAAAACGAGAAAATAGCGCCTTGCGACATTCCCCGCAACTACAA
664157_30677280_HHV-7_JI_U43400.1_144861bp_1_162	CTTTCTCTCCGCTTCAAAAAACGAGAAAATAGCGCCTTGCGACATTCCCCGCAACTACAAAGATTAGATACAACAAAGTGATAATTCATCACGAATGAAAAAAAGATTTTGTCGTCTAAG
664157_30677280_HHV-7_JI_U43400.1_144861bp_1_163	AGATTAGATACAACAAAGTGATAATTCATCACGAATGAAAAAAAGATTTTGTCGTCTAAGAAATTTTTTAATAGGAGATTGGAAGGCGCTACGACTTCACCAGCTATTTCCTTCGGATTT
664157_30677280_HHV-7_JI_U43400.1_144861bp_1_164	AAATTTTTTAATAGGAGATTGGAAGGCGCTACGACTTCACCAGCTATTTCCTTCGGATTTGGAAGTAACTCAAACAAACTCAACAATATATTTGAATCACAGCGATTTCTAAGCATAAAA
664157_30677280_HHV-7_JI_U43400.1_144861bp_1_165	GGAAGTAACTCAAACAAACTCAACAATATATTTGAATCACAGCGATTTCTAAGCATAAAAAATTGTAAAAACAATGAATCTAAGAGCTCCATTAAACGATTAAAGTGAGACGAAAGAGTG
664157_30677280_HHV-7_JI_U43400.1_144861bp_1_166	AATTGTAAAAACAATGAATCTAAGAGCTCCATTAAACGATTAAAGTGAGACGAAAGAGTGTCGCGCTTTTCTCCAGCTACAAAATATTCCGCTATTGTAAAATGAGCCAAAGCTGTAGCT
664157_30677280_HHV-7_JI_U43400.1_144861bp_1_167	TCGCGCTTTTCTCCAGCTACAAAATATTCCGCTATTGTAAAATGAGCCAAAGCTGTAGCTTTAGTCAGAACTCGATAAAACGGAGAAGTGCACTCTTCAAACCTTAGACTTTGAAGAAAC
664157_30677280_HHV-7_JI_U43400.1_144861bp_1_168	TTAGTCAGAACTCGATAAAACGGAGAAGTGCACTCTTCAAACCTTAGACTTTGAAGAAACGCGAGAAAACCGTCTAAAGTCATTGTTGCCTTGTCCCTCCTTTTAAATGAAACGGAGTTT
664157_30677280_HHV-7_JI_U43400.1_144861bp_1_169	GCGAGAAAACCGTCTAAAGTCATTGTTGCCTTGTCCCTCCTTTTAAATGAAACGGAGTTTTTCTCCACTAAAACAGCCCATAGTAGACTGTCATCGTAAAATTTTAAGCCTCGAGTTTGG

664157_30677280_HHV-7_JI_U43400.1_144861bp_1_170	TTCTCCACTAAAACAGCCCATAGTAGACTGTCATCGTAAAATTTTAAGCCTCGAGTTTGGAACAAATCTTCTGAAGTGAGATGACCGCTATCCGCAATCATCTCCTCTGTCAGCTCATTC
664157_30677280_HHV-7_JI_U43400.1_144861bp_1_171	AACAAATCTTCTGAAGTGAGATGACCGCTATCCGCAATCATCTCCTCTGTCAGCTCATTCAAGTTGACGTCTAACTCGCATGAGTTAAATGCCGTCCATGACAGATAAGGGTGATTCGGT
664157_30677280_HHV-7_JI_U43400.1_144861bp_1_172	AAGTTGACGTCTAACTCGCATGAGTTAAATGCCGTCCATGACAGATAAGGGTGATTCGGTATAACATACGTAATGCGATTCCTTAGCGGCCCTTTACAAACATCATAATCGATTAAATCC
664157_30677280_HHV-7_JI_U43400.1_144861bp_1_173	ATAACATACGTAATGCGATTCCTTAGCGGCCCTTTACAAACATCATAATCGATTAAATCCATTACGCAAATCCTTGTCAAAAATGTTTATTCATTTCCATCACTAGCATCGACTCCAACA
664157_30677280_HHV-7_JI_U43400.1_144861bp_1_174	ATTACGCAAATCCTTGTCAAAAATGTTTATTCATTTCCATCACTAGCATCGACTCCAACACAGGCTAACGTTTCTACATTGGCAAAGTAACACAAGTCATTCTGAATCTTGTCAAGACCC
664157_30677280_HHV-7_JI_U43400.1_144861bp_1_175	CAGGCTAACGTTTCTACATTGGCAAAGTAACACAAGTCATTCTGAATCTTGTCAAGACCCCCCAGCTTATACCCTACAGCTCTTACATCTTTTTCTGGACTGGTCTGGAAATTTCCATTC
664157_30677280_HHV-7_JI_U43400.1_144861bp_1_176	CCCAGCTTATACCCTACAGCTCTTACATCTTTTTCTGGACTGGTCTGGAAATTTCCATTCCCAAAATTTCCAAAATATTTGAGACCACTGAAAAAAAGTGCCATTGCTCCATTAATGTTT
664157_30677280_HHV-7_JI_U43400.1_144861bp_1_177	CCAAAATTTCCAAAATATTTGAGACCACTGAAAAAAAGTGCCATTGCTCCATTAATGTTTTTTGTGTTACTTAAATATTCAGCTTCGATGTTCACGATCGATCTCTCCAGCACATCTATG
664157_30677280_HHV-7_JI_U43400.1_144861bp_1_178	TTTGTGTTACTTAAATATTCAGCTTCGATGTTCACGATCGATCTCTCCAGCACATCTATGAAGCTCTTTTCCTTAGCATTCACTATGAAGAAACCTGGTGCTAGATTATTTGCAGCAACC
664157_30677280_HHV-7_JI_U43400.1_144861bp_1_179	AAGCTCTTTTCCTTAGCATTCACTATGAAGAAACCTGGTGCTAGATTATTTGCAGCAACCAAGTCCGTGTGCTTCGCAGGCTGCCTTTGGATGTAAAGATGATGCAAATAAAAGCTTTCC
664157_30677280_HHV-7_JI_U43400.1_144861bp_1_180	AAGTCCGTGTGCTTCGCAGGCTGCCTTTGGATGTAAAGATGATGCAAATAAAAGCTTTCCATGAAGTTTGTTTCATCATAGTTTTTGTAATACGGAATTTCAAAGCTCATCTTTATCACT
664157_30677280_HHV-7_JI_U43400.1_144861bp_1_181	ATGAAGTTTGTTTCATCATAGTTTTTGTAATACGGAATTTCAAAGCTCATCTTTATCACTTCTAAGCAGTTGAAAATAAAGTTTGAGGATGTGCCAGGATTTCTGACAATATCTTTAACC

664157_30677280_HHV-7_JI_U43400.1_144861bp_1_182	TCTAAGCAGTTGAAAATAAAGTTTGAGGATGTGCCAGGATTTCTGACAATATCTTTAACCTGCTTCAACATATTGAAATAAACGATATTGTTCATACAACCCTTACTGCCCAGTTTCATT
664157_30677280_HHV-7_JI_U43400.1_144861bp_1_183	TGCTTCAACATATTGAAATAAACGATATTGTTCATACAACCCTTACTGCCCAGTTTCATTTCACATTCCATGCTAGACTCCAAGTTCTTTAATTCATCTAAGTACACATCATAATACATT
664157_30677280_HHV-7_JI_U43400.1_144861bp_1_184	TCACATTCCATGCTAGACTCCAAGTTCTTTAATTCATCTAAGTACACATCATAATACATTTTCAGTTCAGTAAACAAAACACTGTTAAGAGTTTGAATTATGAAAAGCAAAAACACAATG
664157_30677280_HHV-7_JI_U43400.1_144861bp_1_185	TTCAGTTCAGTAAACAAAACACTGTTAAGAGTTTGAATTATGAAAAGCAAAAACACAATGTTACTAAGAATATTCAACAGATTTCTTTTGTCCTGACCCATATCCACAGTAACCGAGATA
664157_30677280_HHV-7_JI_U43400.1_144861bp_1_186	TTACTAAGAATATTCAACAGATTTCTTTTGTCCTGACCCATATCCACAGTAACCGAGATAGGAAACCTGGAATGTTCAATCTGCCCCTCAATAAAACTTAAACACAAATCTTTGGCTACA
664157_30677280_HHV-7_JI_U43400.1_144861bp_1_187	GGAAACCTGGAATGTTCAATCTGCCCCTCAATAAAACTTAAACACAAATCTTTGGCTACAAACATAGAAAGATTAGAATATTTCTCAGATAAATGTGGAAAAATAGGTAAATCGATCTTA
664157_30677280_HHV-7_JI_U43400.1_144861bp_1_188	AACATAGAAAGATTAGAATATTTCTCAGATAAATGTGGAAAAATAGGTAAATCGATCTTACCCAAAGCTGGAAAGAAATGTAATTTGACAGAGCCAGGATTCTTTTTCATTGTGCCTACA
664157_30677280_HHV-7_JI_U43400.1_144861bp_1_189	CCCAAAGCTGGAAAGAAATGTAATTTGACAGAGCCAGGATTCTTTTTCATTGTGCCTACATTTTTAAACCTGAAAAAATTGTATCGACATTCTTCGCAACCACAAAATAGTAAAAAGTGC
664157_30677280_HHV-7_JI_U43400.1_144861bp_1_190	TTTTTAAACCTGAAAAAATTGTATCGACATTCTTCGCAACCACAAAATAGTAAAAAGTGCTCAAGTAAGTACAACTGAAAACGATCAAAAAAATCGCGTCTGAAGAATGATTCTGTTTTG
664157_30677280_HHV-7_JI_U43400.1_144861bp_1_191	TCAAGTAAGTACAACTGAAAACGATCAAAAAAATCGCGTCTGAAGAATGATTCTGTTTTGTTTTGCATTTCTGTCTTAAAAGATTCGCTCCAAAATGCACAGCTAAAGCCCCCTAACTTC
664157_30677280_HHV-7_JI_U43400.1_144861bp_1_192	TTTTGCATTTCTGTCTTAAAAGATTCGCTCCAAAATGCACAGCTAAAGCCCCCTAACTTCTGCTTAGATTCTTTACTCAATTCCTTCAATTTTCCATCTAGTTTAAAAAATAACATAGAA
664157_30677280_HHV-7_JI_U43400.1_144861bp_1_193	TGCTTAGATTCTTTACTCAATTCCTTCAATTTTCCATCTAGTTTAAAAAATAACATAGAAAGGTACTCACATAGGTAGCGACACCAGTATCCAATTCCAGGCTTAGCACCCAAATTAAAC

664157_30677280_HHV-7_JI_U43400.1_144861bp_1_194	AGGTACTCACATAGGTAGCGACACCAGTATCCAATTCCAGGCTTAGCACCCAAATTAAACAATGATGCCCCAGAATAGAACCCTACAGCTGCAAGTTTAGTAATGCACTGCCATTTGGGG
664157_30677280_HHV-7_JI_U43400.1_144861bp_1_195	AATGATGCCCCAGAATAGAACCCTACAGCTGCAAGTTTAGTAATGCACTGCCATTTGGGGTCAAAATCTTCCATAAATTTTAAATCGCGCAACTGCAAAAGAAATTTATCAGCCCTTGTG
664157_30677280_HHV-7_JI_U43400.1_144861bp_1_196	TCAAAATCTTCCATAAATTTTAAATCGCGCAACTGCAAAAGAAATTTATCAGCCCTTGTGAAAGTTGATGGATACTTGAACATTTCGTACTCATAGTCTATCAACCTATTCCAAATTACA
664157_30677280_HHV-7_JI_U43400.1_144861bp_1_197	AAAGTTGATGGATACTTGAACATTTCGTACTCATAGTCTATCAACCTATTCCAAATTACACCATCTGAACATAATCTTAGCCCTTTTATTCCAAAGCGTCCAGCAGCTGATGCTTGAAGG
664157_30677280_HHV-7_JI_U43400.1_144861bp_1_198	CCATCTGAACATAATCTTAGCCCTTTTATTCCAAAGCGTCCAGCAGCTGATGCTTGAAGGCCGTTGATTAGTCTGGATTCTGTCACCGGAACAATGTACTTCAGTTCCGTGTCTCCGTTT
664157_30677280_HHV-7_JI_U43400.1_144861bp_1_199	CCGTTGATTAGTCTGGATTCTGTCACCGGAACAATGTACTTCAGTTCCGTGTCTCCGTTTGGAATGATGATTGGATGTAGAAACTTTGTAACACTTTGAACAAGAGGATTACTGATTTCA
664157_30677280_HHV-7_JI_U43400.1_144861bp_1_200	GGAATGATGATTGGATGTAGAAACTTTGTAACACTTTGAACAAGAGGATTACTGATTTCAGATGAGTGCACTGCAAACAGAATACTTGGCATGTTCTTCAATAATCTGAAAAAGAACATC
664157_30677280_HHV-7_JI_U43400.1_144861bp_1_201	GATGAGTGCACTGCAAACAGAATACTTGGCATGTTCTTCAATAATCTGAAAAAGAACATCCATGGAACCATCAGTAAGAGTATTGTGTAGAGAATACCGAAAAGAAGAACTTTGATCTTT
664157_30677280_HHV-7_JI_U43400.1_144861bp_1_202	CATGGAACCATCAGTAAGAGTATTGTGTAGAGAATACCGAAAAGAAGAACTTTGATCTTTACATACCTATCACACCTTGGATACATATGCCGTGGTTTCTTCCGAAGAATCGGAGACTCT
664157_30677280_HHV-7_JI_U43400.1_144861bp_1_203	ACATACCTATCACACCTTGGATACATATGCCGTGGTTTCTTCCGAAGAATCGGAGACTCTCCATTTAACATTCTGTTCAAATTTTCCGCCTCACTTTCACTCACATAAATCAAACTGTCA
664157_30677280_HHV-7_JI_U43400.1_144861bp_1_204	CCATTTAACATTCTGTTCAAATTTTCCGCCTCACTTTCACTCACATAAATCAAACTGTCATCATGCCAGTTCAGGGCATATCTTTCTTTGTATAAGAGTTCTTCAAACCCTCGCCTGATA
664157_30677280_HHV-7_JI_U43400.1_144861bp_1_205	TCATGCCAGTTCAGGGCATATCTTTCTTTGTATAAGAGTTCTTCAAACCCTCGCCTGATAAATTCTCTAATAGACCTTGCAACTTTGTTGATCTTGTTATCACAATACACATAAATGTGA

664157_30677280_HHV-7_JI_U43400.1_144861bp_1_206	AATTCTCTAATAGACCTTGCAACTTTGTTGATCTTGTTATCACAATACACATAAATGTGAAAATTGGGACCTAGTAGGATGACAGCTCTACAATTTGGCTCTACCTGACATCTCGCGCAA
664157_30677280_HHV-7_JI_U43400.1_144861bp_1_207	AAATTGGGACCTAGTAGGATGACAGCTCTACAATTTGGCTCTACCTGACATCTCGCGCAAGTTCCTATGACAACATACTTTCTTCTTAGTATTTTTTCGAGAATACCAGAACCTGTGATG
664157_30677280_HHV-7_JI_U43400.1_144861bp_1_208	GTTCCTATGACAACATACTTTCTTCTTAGTATTTTTTCGAGAATACCAGAACCTGTGATGTAATGGATGTCATTCGAAGAACAGAAAGACAAATCAAAATTTTGAAAAGCATCGATACGA
664157_30677280_HHV-7_JI_U43400.1_144861bp_1_209	TAATGGATGTCATTCGAAGAACAGAAAGACAAATCAAAATTTTGAAAAGCATCGATACGAATTTTTTGACCACATTGGCGATTGACAAAATTCTTTAAGGCTGGAATATTTTTTTGTAAG
664157_30677280_HHV-7_JI_U43400.1_144861bp_1_210	ATTTTTTGACCACATTGGCGATTGACAAAATTCTTTAAGGCTGGAATATTTTTTTGTAAGAGTACGAACTCCTCACCGCTGCATTTGGTTTCTCCGTACCACAGTCTGTCTAGCTCAATA
664157_30677280_HHV-7_JI_U43400.1_144861bp_1_211	AGTACGAACTCCTCACCGCTGCATTTGGTTTCTCCGTACCACAGTCTGTCTAGCTCAATATCTAATTCAATCCTTGCAAAAGGTGGGAAATTTCTCAGCCCGATACTGCAAAAATGTGCT
664157_30677280_HHV-7_JI_U43400.1_144861bp_1_212	TCTAATTCAATCCTTGCAAAAGGTGGGAAATTTCTCAGCCCGATACTGCAAAAATGTGCTGGAGATTCCGCTATGATGTACAATTCATCTGTGACCGTATCTAATGCAAGCATCTTATTT
664157_30677280_HHV-7_JI_U43400.1_144861bp_1_213	GGAGATTCCGCTATGATGTACAATTCATCTGTGACCGTATCTAATGCAAGCATCTTATTTGATGAGCAGTACATCACAAATGGATCAAAGTCCGGGTCCGGTTCTCGGTAATTTGGATGT
664157_30677280_HHV-7_JI_U43400.1_144861bp_1_214	GATGAGCAGTACATCACAAATGGATCAAAGTCCGGGTCCGGTTCTCGGTAATTTGGATGTGCATAACCAACAGCGCAAAGAAATTCGTCTGCTCCTACCAAATTACTCCAATCATAAGTG
664157_30677280_HHV-7_JI_U43400.1_144861bp_1_215	GCATAACCAACAGCGCAAAGAAATTCGTCTGCTCCTACCAAATTACTCCAATCATAAGTGATGACACTACGTCTACATTTGAAATGATAAAATTCGCCAAAAATAAGCACATAGTTTGGC
664157_30677280_HHV-7_JI_U43400.1_144861bp_1_216	ATGACACTACGTCTACATTTGAAATGATAAAATTCGCCAAAAATAAGCACATAGTTTGGCGGCCAAGCAAGACAGCACTTTATACCATTGCGAACTATCTCCATGCCTTCTCCATATGAA
664157_30677280_HHV-7_JI_U43400.1_144861bp_1_217	GGCCAAGCAAGACAGCACTTTATACCATTGCGAACTATCTCCATGCCTTCTCCATATGAAGACAGCAGCAACTCAACGGCAAAATCAAAAGACGGTAAATCCGGAGTCGGTTCAATGCAA

664157_30677280_HHV-7_JI_U43400.1_144861bp_1_218	GACAGCAGCAACTCAACGGCAAAATCAAAAGACGGTAAATCCGGAGTCGGTTCAATGCAAACATCATTGCACACAGGAACAATTTCTGCAAAATTAACTTGTGATTAGTACTTCCGTCAC
664157_30677280_HHV-7_JI_U43400.1_144861bp_1_219	ACATCATTGCACACAGGAACAATTTCTGCAAAATTAACTTGTGATTAGTACTTCCGTCACTTTCAGAAAAATTAAAAACCATAAAAAGTACTGTTACTTACTTAGATACCAGGAGTACTG
664157_30677280_HHV-7_JI_U43400.1_144861bp_1_220	TTTCAGAAAAATTAAAAACCATAAAAAGTACTGTTACTTACTTAGATACCAGGAGTACTGTTGAACCGGTGCACTTCCAAGAGTTGATAATCTTCCAAATCTGGCAGTGACTTTTCCAAT
664157_30677280_HHV-7_JI_U43400.1_144861bp_1_221	TTGAACCGGTGCACTTCCAAGAGTTGATAATCTTCCAAATCTGGCAGTGACTTTTCCAATTTTATCATAACCAAAATCTCCACAATATCTCGCGAAGCCGATCCTTGCAAAAGTCAGTAA
664157_30677280_HHV-7_JI_U43400.1_144861bp_1_222	TTTATCATAACCAAAATCTCCACAATATCTCGCGAAGCCGATCCTTGCAAAAGTCAGTAAACAATCAGCAATCCGCGTCAACACTTTATCAATCCAATCATAATAATAAATTGCTCCTGT
664157_30677280_HHV-7_JI_U43400.1_144861bp_1_223	ACAATCAGCAATCCGCGTCAACACTTTATCAATCCAATCATAATAATAAATTGCTCCTGTTTCTCCAATGCATAGAATTGGTCTCAAACCATAGTCTTCGGCTGAAATATTTAAAAAACC
664157_30677280_HHV-7_JI_U43400.1_144861bp_1_224	TTCTCCAATGCATAGAATTGGTCTCAAACCATAGTCTTCGGCTGAAATATTTAAAAAACCTAAAACAACTACTTTTTCAAACAGTGCCTCTTGTTCTTCAAATCTTTTTTGGTCTGAGGT
664157_30677280_HHV-7_JI_U43400.1_144861bp_1_225	TAAAACAACTACTTTTTCAAACAGTGCCTCTTGTTCTTCAAATCTTTTTTGGTCTGAGGTGCCACAGTGTATGTTTTTCCTGTCTTGCAGGATTAACGCTGTAAAGATTGGAGTTTCCAA
664157_30677280_HHV-7_JI_U43400.1_144861bp_1_226	GCCACAGTGTATGTTTTTCCTGTCTTGCAGGATTAACGCTGTAAAGATTGGAGTTTCCAAACGAAGTTTTAAACCAGTGTTTATAGAAACGAAGTGTTCAATCTCTGCTAACGTTTTACA
664157_30677280_HHV-7_JI_U43400.1_144861bp_1_227	ACGAAGTTTTAAACCAGTGTTTATAGAAACGAAGTGTTCAATCTCTGCTAACGTTTTACACCTTGCCAATTCATCTAATTCGGAGAGCATTGAAATATAATTTCTCCTCAAAGCTTTTAG
664157_30677280_HHV-7_JI_U43400.1_144861bp_1_228	CCTTGCCAATTCATCTAATTCGGAGAGCATTGAAATATAATTTCTCCTCAAAGCTTTTAGTGCCAAAGCTGTTTTCCTGTTAAATCTTAGACTAGAATTCAAATTAAGTGGTTCCTGTAA
664157_30677280_HHV-7_JI_U43400.1_144861bp_1_229	TGCCAAAGCTGTTTTCCTGTTAAATCTTAGACTAGAATTCAAATTAAGTGGTTCCTGTAATAATCCTAAGTCTTTAAGAGTCTCCAATTTTTCAAACTTTGGACCATCATAATAAAAGCG

664157_30677280_HHV-7_JI_U43400.1_144861bp_1_230	TAATCCTAAGTCTTTAAGAGTCTCCAATTTTTCAAACTTTGGACCATCATAATAAAAGCGAGTATAAACAGGTTCTGAGCAAGTGATTACATTTGTACCTAGAATACCAAATCTAACAAA
664157_30677280_HHV-7_JI_U43400.1_144861bp_1_231	AGTATAAACAGGTTCTGAGCAAGTGATTACATTTGTACCTAGAATACCAAATCTAACAAATTTGTCAAAGTCTTCAGCTATGTAATACAAAACATCATCCTCCAAATCGTGACAAAAAAT
664157_30677280_HHV-7_JI_U43400.1_144861bp_1_232	TTTGTCAAAGTCTTCAGCTATGTAATACAAAACATCATCCTCCAAATCGTGACAAAAAATTCGACTCGATTTTCCCACAAAAATTGGCACGTCTTTCCTTCCAATTTGAATCGCTCCAAT
664157_30677280_HHV-7_JI_U43400.1_144861bp_1_233	TCGACTCGATTTTCCCACAAAAATTGGCACGTCTTTCCTTCCAATTTGAATCGCTCCAATCAAGCACAAATTTTCCTTAAATCCTTGGTAATGGTCATTCAGTTCTGCCAATTGTTGTTC
664157_30677280_HHV-7_JI_U43400.1_144861bp_1_234	CAAGCACAAATTTTCCTTAAATCCTTGGTAATGGTCATTCAGTTCTGCCAATTGTTGTTCTGAGTAACCAGCAACGTCAACTGGCTGTGCAAGCCTTAACCACAAATTCTTTGGCCAAAT
664157_30677280_HHV-7_JI_U43400.1_144861bp_1_235	TGAGTAACCAGCAACGTCAACTGGCTGTGCAAGCCTTAACCACAAATTCTTTGGCCAAATCAAAGACAAGCATTCATTTCGATGTCGTTCAGTAAGAAGGCGAATTCCAGGGAAATCAGT
664157_30677280_HHV-7_JI_U43400.1_144861bp_1_236	CAAAGACAAGCATTCATTTCGATGTCGTTCAGTAAGAAGGCGAATTCCAGGGAAATCAGTAAAACGACATAAATTCTTCAATTCCCTTAACAATCCATCATTTAGTTGTCCAACACAAGG
664157_30677280_HHV-7_JI_U43400.1_144861bp_1_237	AAAACGACATAAATTCTTCAATTCCCTTAACAATCCATCATTTAGTTGTCCAACACAAGGCAAAGTTTTATATTGTTCTGCCATCGTCTCAAAATTTTTCATTGAAGTGAATGTGGTAAG
664157_30677280_HHV-7_JI_U43400.1_144861bp_1_238	CAAAGTTTTATATTGTTCTGCCATCGTCTCAAAATTTTTCATTGAAGTGAATGTGGTAAGAAGTTTGTATTCTCATCCATTTCTCAAAAACTCAACCAGATAAATATAAGAGAAAAAAAT
664157_30677280_HHV-7_JI_U43400.1_144861bp_1_239	AAGTTTGTATTCTCATCCATTTCTCAAAAACTCAACCAGATAAATATAAGAGAAAAAAATCACATGACAGGAAATAAATTTTAAAGCTTTGCAGATTTTTTATTTCCCATTACAGGAAAC
664157_30677280_HHV-7_JI_U43400.1_144861bp_1_240	CACATGACAGGAAATAAATTTTAAAGCTTTGCAGATTTTTTATTTCCCATTACAGGAAACATTAGAGTGATTAAAACCATAAGCCTGGTGTTATCGGATTAAAAACCTCTTGTCGAAAGT
664157_30677280_HHV-7_JI_U43400.1_144861bp_1_241	ATTAGAGTGATTAAAACCATAAGCCTGGTGTTATCGGATTAAAAACCTCTTGTCGAAAGTAGGAATCGGATTTTTACCTTTTTTAAAGATCGATTTTTAGAACATTCACGGTAACAATAC

664157_30677280_HHV-7_JI_U43400.1_144861bp_1_242	AGGAATCGGATTTTTACCTTTTTTAAAGATCGATTTTTAGAACATTCACGGTAACAATACAAAACAAACAGTGTCATTAATATTACAAACTCAGAATCTGCCACTGAAAGGAAAATCCCT
664157_30677280_HHV-7_JI_U43400.1_144861bp_1_243	AAAACAAACAGTGTCATTAATATTACAAACTCAGAATCTGCCACTGAAAGGAAAATCCCTTTTCGCCCTAAGTAACTACACTTAAAAATGGCTATAGCAGAAAGCGGAGCGTTCGAAATA
664157_30677280_HHV-7_JI_U43400.1_144861bp_1_244	TTTCGCCCTAAGTAACTACACTTAAAAATGGCTATAGCAGAAAGCGGAGCGTTCGAAATAAATGTCAATCTTGGAAAATCCATCGCTTCCATACAAAAAAATCCTATAATCTACATGCGT
664157_30677280_HHV-7_JI_U43400.1_144861bp_1_245	AATGTCAATCTTGGAAAATCCATCGCTTCCATACAAAAAAATCCTATAATCTACATGCGTAGACATCTCTCTTTTTATGTGGAGTTATTAAAATTTATCATTCATCAATATGAGCAATGT
664157_30677280_HHV-7_JI_U43400.1_144861bp_1_246	AGACATCTCTCTTTTTATGTGGAGTTATTAAAATTTATCATTCATCAATATGAGCAATGTTTTTTACCACCGAAAGGAACGATACTTTACCACAATGGCTTAATCGAACTAAATACTTTA
664157_30677280_HHV-7_JI_U43400.1_144861bp_1_247	TTTTTACCACCGAAAGGAACGATACTTTACCACAATGGCTTAATCGAACTAAATACTTTAATTATCGATCTGAATCAACAAATTACATCGAAACAGCAGATCTATAGTTGGACGAGCATT
664157_30677280_HHV-7_JI_U43400.1_144861bp_1_248	ATTATCGATCTGAATCAACAAATTACATCGAAACAGCAGATCTATAGTTGGACGAGCATTACTTTGCCAAAAATATTTTCGACTAAAGAATTATATTTTATTGTCGCGTCACCAGAGTCG
664157_30677280_HHV-7_JI_U43400.1_144861bp_1_249	ACTTTGCCAAAAATATTTTCGACTAAAGAATTATATTTTATTGTCGCGTCACCAGAGTCGGAAAACATAACTCTCAATCCAGCTGTTACTAAAGGAGGATGGTTATCTGGGAGTTTTAGC
664157_30677280_HHV-7_JI_U43400.1_144861bp_1_250	GAAAACATAACTCTCAATCCAGCTGTTACTAAAGGAGGATGGTTATCTGGGAGTTTTAGCTTTCCATTGAGTTTAAGTTGTGCATACGCCCTCACTGGCGTATCTTCAACAATCTATATG
664157_30677280_HHV-7_JI_U43400.1_144861bp_1_251	TTTCCATTGAGTTTAAGTTGTGCATACGCCCTCACTGGCGTATCTTCAACAATCTATATGTTACCATTTATTCCATATAAATTCCCAATGACTTACGTAGACTTTTCGACACTTCGAACA
664157_30677280_HHV-7_JI_U43400.1_144861bp_1_252	TTACCATTTATTCCATATAAATTCCCAATGACTTACGTAGACTTTTCGACACTTCGAACATACGAGGTAACAAGTGAATATGGATCAATCCAAATTATAAAACAGCGGAATTTTTTATTT
664157_30677280_HHV-7_JI_U43400.1_144861bp_1_253	TACGAGGTAACAAGTGAATATGGATCAATCCAAATTATAAAACAGCGGAATTTTTTATTTTTGGGAATTATACGGGATCTGTCATGGAAAAGTCAGAGGGATAACAAGAATTTTATCCTG

664157_30677280_HHV-7_JI_U43400.1_144861bp_1_254	TTGGGAATTATACGGGATCTGTCATGGAAAAGTCAGAGGGATAACAAGAATTTTATCCTGAAAGCAATGTTCGTTGGAAACTGGTTAGGGATACAAATTCCAGAAGCTTTTGCATTGAGA
664157_30677280_HHV-7_JI_U43400.1_144861bp_1_255	AAAGCAATGTTCGTTGGAAACTGGTTAGGGATACAAATTCCAGAAGCTTTTGCATTGAGACTTTTTAATAACACACGGTTTTCTATTCAGGATTTTGAATTCTCCATTAACATACAAAAT
664157_30677280_HHV-7_JI_U43400.1_144861bp_1_256	CTTTTTAATAACACACGGTTTTCTATTCAGGATTTTGAATTCTCCATTAACATACAAAATATAAACCTTACTAGGGACAATAAAATTTTGGGCTCTCTTTCCACGGTTTCTTGTGATCAG
664157_30677280_HHV-7_JI_U43400.1_144861bp_1_257	ATAAACCTTACTAGGGACAATAAAATTTTGGGCTCTCTTTCCACGGTTTCTTGTGATCAGATGCCACCAAACTTGTCTCCAGAGAATCTCCCAAACTATCTAGTTATTCAGTTCGAATTG
664157_30677280_HHV-7_JI_U43400.1_144861bp_1_258	ATGCCACCAAACTTGTCTCCAGAGAATCTCCCAAACTATCTAGTTATTCAGTTCGAATTGGTTTCAACTCTCGCAAATCCTGATCATCTACTGTTTTCTTGCAATCCCAAATTGTTTTTC
664157_30677280_HHV-7_JI_U43400.1_144861bp_1_259	GTTTCAACTCTCGCAAATCCTGATCATCTACTGTTTTCTTGCAATCCCAAATTGTTTTTCACAGGAGATATTCTGAACAGCGCTATAAATTTACAACATAGTCCTAATCATTATGAGCTT
664157_30677280_HHV-7_JI_U43400.1_144861bp_1_260	ACAGGAGATATTCTGAACAGCGCTATAAATTTACAACATAGTCCTAATCATTATGAGCTTACAGTGTACGCACCACATAATTTACATTTCTATCCCAGCTGTTTTCATATAGTAACATTA
664157_30677280_HHV-7_JI_U43400.1_144861bp_1_261	ACAGTGTACGCACCACATAATTTACATTTCTATCCCAGCTGTTTTCATATAGTAACATTACCAATTCAGTTTTCATCCAGAAATGATAGACAGATGTTGGTGTCAAGCTATCCTAATGAA
664157_30677280_HHV-7_JI_U43400.1_144861bp_1_262	CCAATTCAGTTTTCATCCAGAAATGATAGACAGATGTTGGTGTCAAGCTATCCTAATGAAGGCTACTTTGAAGTACAAATGTGCCCATGGGTACAGAATTCTCCTCTTCAAATTGTTATT
664157_30677280_HHV-7_JI_U43400.1_144861bp_1_263	GGCTACTTTGAAGTACAAATGTGCCCATGGGTACAGAATTCTCCTCTTCAAATTGTTATTAAATCTTTTTCAAAAAATCTAGTGCTACCGCAAGGCACCCCTATTGCAATTCTCTTATAT
664157_30677280_HHV-7_JI_U43400.1_144861bp_1_264	AAATCTTTTTCAAAAAATCTAGTGCTACCGCAAGGCACCCCTATTGCAATTCTCTTATATATGGAAAAAATGACAACAGGAAAAAATTCTTTACGGGATCAAGAATTAAAAATTAATAAA
664157_30677280_HHV-7_JI_U43400.1_144861bp_1_265	ATGGAAAAAATGACAACAGGAAAAAATTCTTTACGGGATCAAGAATTAAAAATTAATAAAGACATTACCCGAATAGGAAACGTAAACCTTCCAAAAGAAAATTTCTTACATTATAATAGC

664157_30677280_HHV-7_JI_U43400.1_144861bp_1_266	GACATTACCCGAATAGGAAACGTAAACCTTCCAAAAGAAAATTTCTTACATTATAATAGCTAATCTTTATTCAACTGTGTTTTAGACGACGTCTTCTTCTGAGTGTGTTAAATGCGATAC
664157_30677280_HHV-7_JI_U43400.1_144861bp_1_267	TAATCTTTATTCAACTGTGTTTTAGACGACGTCTTCTTCTGAGTGTGTTAAATGCGATACTAGTTTATTAACTATATCATCATCTTTTTGAACAGAATTTTCAAGATCGATTAAATCTTT
664157_30677280_HHV-7_JI_U43400.1_144861bp_1_268	TAGTTTATTAACTATATCATCATCTTTTTGAACAGAATTTTCAAGATCGATTAAATCTTTATCGCCATTTTCTTGAGCCTTGCTAGCACCGAAAATGGTATTTTGCGTCTCTTTGCCTGT
664157_30677280_HHV-7_JI_U43400.1_144861bp_1_269	ATCGCCATTTTCTTGAGCCTTGCTAGCACCGAAAATGGTATTTTGCGTCTCTTTGCCTGTTAAAAGTGAACCTGAATCAAAATCCTGGATTTCAGCATTTGGTAAAAAAAATGGAGACCG
664157_30677280_HHV-7_JI_U43400.1_144861bp_1_270	TAAAAGTGAACCTGAATCAAAATCCTGGATTTCAGCATTTGGTAAAAAAAATGGAGACCGCACAGGCTGCATCTTTTCAGTGAAGCTAAAATTTTGAAATAAAGGATCTCTCTTTCTGGA
664157_30677280_HHV-7_JI_U43400.1_144861bp_1_271	CACAGGCTGCATCTTTTCAGTGAAGCTAAAATTTTGAAATAAAGGATCTCTCTTTCTGGAGTTCAGTAAATTCTTCAGGTTGTTGTCATTGAAAGTCGCAATGTTAGAAACATCTGTTTT
664157_30677280_HHV-7_JI_U43400.1_144861bp_1_272	GTTCAGTAAATTCTTCAGGTTGTTGTCATTGAAAGTCGCAATGTTAGAAACATCTGTTTTTTTCGGGGGTTCGAGTACGTCTTTTAAGCTTCGCTCTTCTAAACTAATTGTATCCGTAAA
664157_30677280_HHV-7_JI_U43400.1_144861bp_1_273	TTTCGGGGGTTCGAGTACGTCTTTTAAGCTTCGCTCTTCTAAACTAATTGTATCCGTAAAATCCGTTAATAATTTCTTAACTTTTTCACCTAGTCCTGATTCAAAAAGTTCTTTTTGAAG
664157_30677280_HHV-7_JI_U43400.1_144861bp_1_274	ATCCGTTAATAATTTCTTAACTTTTTCACCTAGTCCTGATTCAAAAAGTTCTTTTTGAAGAAGATCGTTCCTTCTACTAAGATCCAGTAATGAAGAACTCGGAGTCCACTTCACATCTTT
664157_30677280_HHV-7_JI_U43400.1_144861bp_1_275	AAGATCGTTCCTTCTACTAAGATCCAGTAATGAAGAACTCGGAGTCCACTTCACATCTTTGACTAATTGATTTTTTTTAGCTGAAGAAGCACTCTCTTTTTCCTTCATTGAAGCGTTTAC
664157_30677280_HHV-7_JI_U43400.1_144861bp_1_276	GACTAATTGATTTTTTTTAGCTGAAGAAGCACTCTCTTTTTCCTTCATTGAAGCGTTTACGTCATTAGTACCTATCATATTTAAAGTCTTGGTGGAAGAATTTGCTTTATTTCCATTTTT
664157_30677280_HHV-7_JI_U43400.1_144861bp_1_277	GTCATTAGTACCTATCATATTTAAAGTCTTGGTGGAAGAATTTGCTTTATTTCCATTTTTTTGCTTAATGCTTTTTTGATCTTGTGGAGGCAATGACATTTCATTATGCTCATCGTCAAA

664157_30677280_HHV-7_JI_U43400.1_144861bp_1_278	TTGCTTAATGCTTTTTTGATCTTGTGGAGGCAATGACATTTCATTATGCTCATCGTCAAAACTTTGTAAGTTCTCGTGGACAATTTCTTTTCTGCGTTTTTCTGAAGGAAGTTTGAAAAT
664157_30677280_HHV-7_JI_U43400.1_144861bp_1_279	ACTTTGTAAGTTCTCGTGGACAATTTCTTTTCTGCGTTTTTCTGAAGGAAGTTTGAAAATGTCTTCATTATTTTGTTTAATTGTTTTGATTCGTTCTCGTAGCTCCAAAATTTTCTGCAA
664157_30677280_HHV-7_JI_U43400.1_144861bp_1_280	GTCTTCATTATTTTGTTTAATTGTTTTGATTCGTTCTCGTAGCTCCAAAATTTTCTGCAAGTCATTGGTCGAGTTTTTAGACAGTTGATCGCGTGATTGCACTCCGTTTTGTCTGTTTAC
664157_30677280_HHV-7_JI_U43400.1_144861bp_1_281	GTCATTGGTCGAGTTTTTAGACAGTTGATCGCGTGATTGCACTCCGTTTTGTCTGTTTACTTCGTTTTCTGGCAGAAAAAAGGTAGAGTGATAATCAATTTTATTTTTGGGTAGCTGAAC
664157_30677280_HHV-7_JI_U43400.1_144861bp_1_282	TTCGTTTTCTGGCAGAAAAAAGGTAGAGTGATAATCAATTTTATTTTTGGGTAGCTGAACTACGTTAACTTTTGGTGAGCTAACAATGTCTATTACTTTGTTTGAGTTGTGTAGATCTAG
664157_30677280_HHV-7_JI_U43400.1_144861bp_1_283	TACGTTAACTTTTGGTGAGCTAACAATGTCTATTACTTTGTTTGAGTTGTGTAGATCTAGTTTGCTCAAGGTATCTATAACTTGGTTAATACCGTCTGGTGCCACAGCAATTTCATTGAA
664157_30677280_HHV-7_JI_U43400.1_144861bp_1_284	TTTGCTCAAGGTATCTATAACTTGGTTAATACCGTCTGGTGCCACAGCAATTTCATTGAATTGGTTTACTTTGTCCTCTGGTCTGTGCATAGTTTGATCAGTTAAATCAAGTATTTCAGT
664157_30677280_HHV-7_JI_U43400.1_144861bp_1_285	TTGGTTTACTTTGTCCTCTGGTCTGTGCATAGTTTGATCAGTTAAATCAAGTATTTCAGTCTGTAGTATTCTGTGCATTTTTTTAGAATTGTTTATAGACGTGGAATTTCTTGGCTCGTC
664157_30677280_HHV-7_JI_U43400.1_144861bp_1_286	CTGTAGTATTCTGTGCATTTTTTTAGAATTGTTTATAGACGTGGAATTTCTTGGCTCGTCCAATATCATGGAATCCTTTTTTACCGAGGATAACAACCCATCTTCAGAGCCTACAAATAA
664157_30677280_HHV-7_JI_U43400.1_144861bp_1_287	CAATATCATGGAATCCTTTTTTACCGAGGATAACAACCCATCTTCAGAGCCTACAAATAAATCTTTGGATAGCGTGTTGTTACTCCGTTGTTCAGTTTTTATAAATGGTTCTGGATTAAA
664157_30677280_HHV-7_JI_U43400.1_144861bp_1_288	ATCTTTGGATAGCGTGTTGTTACTCCGTTGTTCAGTTTTTATAAATGGTTCTGGATTAAATGATGAACCATGCGAAGTTTTTTTAGAACTACTCTCAAATTGATTGCTTAAAGACGATTT
664157_30677280_HHV-7_JI_U43400.1_144861bp_1_289	TGATGAACCATGCGAAGTTTTTTTAGAACTACTCTCAAATTGATTGCTTAAAGACGATTTCGACAAATCTGTTAAAACTTTGAATGTCTGAATCGGTTCAGGAGTAAATTTTACACGATC

664157_30677280_HHV-7_JI_U43400.1_144861bp_1_290	CGACAAATCTGTTAAAACTTTGAATGTCTGAATCGGTTCAGGAGTAAATTTTACACGATCATCCCATGTACTCTTTACGGGAAGAGCATGTAACTCAAGACTTTTTGTTATAACATCAAA
664157_30677280_HHV-7_JI_U43400.1_144861bp_1_291	ATCCCATGTACTCTTTACGGGAAGAGCATGTAACTCAAGACTTTTTGTTATAACATCAAAGTCTTCGGTTAAAATTTTAAAAAAAGAAGTTACTCTATGACTAGTTAATGGCAAACTTAA
664157_30677280_HHV-7_JI_U43400.1_144861bp_1_292	GTCTTCGGTTAAAATTTTAAAAAAAGAAGTTACTCTATGACTAGTTAATGGCAAACTTAAAATTTGATAAGAATATATCGAAACAAAATTTTTGTTGTTTTCAATCGCCTTTAAAATTGC
664157_30677280_HHV-7_JI_U43400.1_144861bp_1_293	AATTTGATAAGAATATATCGAAACAAAATTTTTGTTGTTTTCAATCGCCTTTAAAATTGCATTTTTCTTTTCATTAATGGTATTTAATGCGAGTTTCTCCATGTTCATCCAGGATCCATG
664157_30677280_HHV-7_JI_U43400.1_144861bp_1_294	ATTTTTCTTTTCATTAATGGTATTTAATGCGAGTTTCTCCATGTTCATCCAGGATCCATGTAATGTAATTAATAAATTCCCAAGATAAAGCAATCGATTGATGTTAGTAACATAGTAGCC
664157_30677280_HHV-7_JI_U43400.1_144861bp_1_295	TAATGTAATTAATAAATTCCCAAGATAAAGCAATCGATTGATGTTAGTAACATAGTAGCCATTTTCATTCCACGGATCAACTATTTGGTAAATTGTGAAAGAAAGCATATTGTTTTTTGC
664157_30677280_HHV-7_JI_U43400.1_144861bp_1_296	ATTTTCATTCCACGGATCAACTATTTGGTAAATTGTGAAAGAAAGCATATTGTTTTTTGCCTTTTGTAAATTCTCGCCAATTTCCGCTCTTTCTTCTGTGGTAAGATTGACGTAATCGGC
664157_30677280_HHV-7_JI_U43400.1_144861bp_1_297	CTTTTGTAAATTCTCGCCAATTTCCGCTCTTTCTTCTGTGGTAAGATTGACGTAATCGGCTGCGTGTGTCACAGTGCCCCAACGAAGAAAACTGCCCAGTTTAAGCAAATCTTTTGCTAC
664157_30677280_HHV-7_JI_U43400.1_144861bp_1_298	TGCGTGTGTCACAGTGCCCCAACGAAGAAAACTGCCCAGTTTAAGCAAATCTTTTGCTACGTTTTTAAATTCTGTTTCAAAGCCAACTTTGTTAAAAGATGTTAAACTAGAATAATCAGT
664157_30677280_HHV-7_JI_U43400.1_144861bp_1_299	GTTTTTAAATTCTGTTTCAAAGCCAACTTTGTTAAAAGATGTTAAACTAGAATAATCAGTAATCATCTGTCTTTGCTCTAAGTAATCTTTCAGAATTTTTTGCACCTTTAGTATTTCTTG
664157_30677280_HHV-7_JI_U43400.1_144861bp_1_300	AATCATCTGTCTTTGCTCTAAGTAATCTTTCAGAATTTTTTGCACCTTTAGTATTTCTTGCCAAACTTCCTCATAGTCTGGTTTCTTTTTACACAAGGTTTGATGAAAATAAATCCAAAG
664157_30677280_HHV-7_JI_U43400.1_144861bp_1_301	CCAAACTTCCTCATAGTCTGGTTTCTTTTTACACAAGGTTTGATGAAAATAAATCCAAAGAATCAAATTGTTGTAAAGTGTTTTTACATTGTTAATGGAATTTCCAGTTTTAACGATACA

664157_30677280_HHV-7_JI_U43400.1_144861bp_1_302	AATCAAATTGTTGTAAAGTGTTTTTACATTGTTAATGGAATTTCCAGTTTTAACGATACATTGACTCAAAATCCATGGATTTTCGCGAATATCAACAACTGGTAAAGACGATATGTTTTC
664157_30677280_HHV-7_JI_U43400.1_144861bp_1_303	TTGACTCAAAATCCATGGATTTTCGCGAATATCAACAACTGGTAAAGACGATATGTTTTCAAAAAATCGAGATAAAAAACACTTTGCTTCATCCGAAATCCAAGCAAAGGGTAAATGTGA
664157_30677280_HHV-7_JI_U43400.1_144861bp_1_304	AAAAAATCGAGATAAAAAACACTTTGCTTCATCCGAAATCCAAGCAAAGGGTAAATGTGACATTTTCATGACTCGTTTTTTCTGTTGAGCATTATTGAACACTTCTTAAAAAATTAGTTT
664157_30677280_HHV-7_JI_U43400.1_144861bp_1_305	CATTTTCATGACTCGTTTTTTCTGTTGAGCATTATTGAACACTTCTTAAAAAATTAGTTTAATAGTCTCTATTTTCTCTTTGATTAGATACATGCGCTGCCGACATGGACACTCTAATTG
664157_30677280_HHV-7_JI_U43400.1_144861bp_1_306	AATAGTCTCTATTTTCTCTTTGATTAGATACATGCGCTGCCGACATGGACACTCTAATTGATTTCCAAAAAATCCTGGTATGATAATCTAATGTTATGATTTGCTACAGCTTTGCTAAAA
664157_30677280_HHV-7_JI_U43400.1_144861bp_1_307	ATTTCCAAAAAATCCTGGTATGATAATCTAATGTTATGATTTGCTACAGCTTTGCTAAAAATGTTACCTTTGCATTTTTAATCATCTTGCAAAACTTTTTTTCACAGCATGATGAAGAGT
664157_30677280_HHV-7_JI_U43400.1_144861bp_1_308	ATGTTACCTTTGCATTTTTAATCATCTTGCAAAACTTTTTTTCACAGCATGATGAAGAGTACAAGTACAATTATACGTGTATTACGCCAACAGTACGGAAAGCCCAAAGACTTGAAAGCG
664157_30677280_HHV-7_JI_U43400.1_144861bp_1_309	ACAAGTACAATTATACGTGTATTACGCCAACAGTACGGAAAGCCCAAAGACTTGAAAGCGTAATTAACGGAATTATGCTAACGCTGATACTTCCTGTTAGTACTGTTGTCATATGCACTC
664157_30677280_HHV-7_JI_U43400.1_144861bp_1_310	TAATTAACGGAATTATGCTAACGCTGATACTTCCTGTTAGTACTGTTGTCATATGCACTCTGCTAATCTACTACAAATGGACAAAACAGACAATTACTTCTCCATATCTTATCACACTCT
664157_30677280_HHV-7_JI_U43400.1_144861bp_1_311	TGCTAATCTACTACAAATGGACAAAACAGACAATTACTTCTCCATATCTTATCACACTCTTTATTAGTGATTCTTTACATTCATTGACTGTGTTACTTCTCACATTGAACCGAGAAGCTC
664157_30677280_HHV-7_JI_U43400.1_144861bp_1_312	TTATTAGTGATTCTTTACATTCATTGACTGTGTTACTTCTCACATTGAACCGAGAAGCTCTCACAAACCTTAATCAGGCTTTGTGTCAATGTGTGCTTTTTGTATACAGTGCGTCCTGCA
664157_30677280_HHV-7_JI_U43400.1_144861bp_1_313	TCACAAACCTTAATCAGGCTTTGTGTCAATGTGTGCTTTTTGTATACAGTGCGTCCTGCACATACAGTCTGTGCATGCTAGCAGTAATATCCACAATACGCTATCGAACCCTGCAAAGAA

664157_30677280_HHV-7_JI_U43400.1_144861bp_1_314	CATACAGTCTGTGCATGCTAGCAGTAATATCCACAATACGCTATCGAACCCTGCAAAGAAGGACATTAAACGACAAAAACAATAATCATATTAAAAGGAACGTTGGAATTTTATTTCTGT
664157_30677280_HHV-7_JI_U43400.1_144861bp_1_315	GGACATTAAACGACAAAAACAATAATCATATTAAAAGGAACGTTGGAATTTTATTTCTGTCTTCTGCCATGTGTGCCATTCCAGCAGTATTATATGTTCAAGTGGAAAAGAAAAAAGGCA
664157_30677280_HHV-7_JI_U43400.1_144861bp_1_316	CTTCTGCCATGTGTGCCATTCCAGCAGTATTATATGTTCAAGTGGAAAAGAAAAAAGGCAATTATGGAAAATGTAATATACACATCTCAACGCAAAAAGCATATGACTTGTTTATAGGAA
664157_30677280_HHV-7_JI_U43400.1_144861bp_1_317	ATTATGGAAAATGTAATATACACATCTCAACGCAAAAAGCATATGACTTGTTTATAGGAATTAAAATTGTCTATTGTTTTCTCTGGGGAATTTTTCCAACTGTCATTTTCAGCTATTTTT
664157_30677280_HHV-7_JI_U43400.1_144861bp_1_318	TTAAAATTGTCTATTGTTTTCTCTGGGGAATTTTTCCAACTGTCATTTTCAGCTATTTTTATGTGATTTTTGGTAAGACCTTGCGTGCCTTGACCCAAAGTAAACATAACAAAACTCTGT
664157_30677280_HHV-7_JI_U43400.1_144861bp_1_319	ATGTGATTTTTGGTAAGACCTTGCGTGCCTTGACCCAAAGTAAACATAACAAAACTCTGTCATTCATTAGCTTACTGATACTATCCTTTTTATGTATTCAAATACCAAATCTCCTAGTAA
664157_30677280_HHV-7_JI_U43400.1_144861bp_1_320	CATTCATTAGCTTACTGATACTATCCTTTTTATGTATTCAAATACCAAATCTCCTAGTAATGTCTGTGGAAATTTTTTTTTGTATATAGCAAATACTTCCTGCTTAGGCACCATACAAA
664157_30677280_HHV-7_JI_U43400.1_144861bp_1_321	TGTCTGTGGAAATTTTTTTTTGTATATAGCAAATACTTCCTGCTTAGGCACCATACAAAGAGAAATTGTGCAAATAATATCTAGATTAATGCCAGAAATACACTGCTTGTCTAATCCGC
664157_30677280_HHV-7_JI_U43400.1_144861bp_1_322	GAGAAATTGTGCAAATAATATCTAGATTAATGCCAGAAATACACTGCTTGTCTAATCCGCTAGTATATGCATTCACTAGAACAGATTTCCGATTACGATTTTACGATTTCATTAAATGTA
664157_30677280_HHV-7_JI_U43400.1_144861bp_1_323	TAGTATATGCATTCACTAGAACAGATTTCCGATTACGATTTTACGATTTCATTAAATGTAATTTGTGTAATTCATCTTTAAAGAGAAAGAGAAATCCTCTGACAATAAAAAATTGAACAG
664157_30677280_HHV-7_JI_U43400.1_144861bp_1_324	ATTTGTGTAATTCATCTTTAAAGAGAAAGAGAAATCCTCTGACAATAAAAAATTGAACAGTGAAGCTTGCTTAAAACTTTGAAAATTTTTTGTGATTTTAAATAAAATTTAAAATCAATT
664157_30677280_HHV-7_JI_U43400.1_144861bp_1_325	TGAAGCTTGCTTAAAACTTTGAAAATTTTTTGTGATTTTAAATAAAATTTAAAATCAATTTATCCTAAGCTTTTCAGGCTATGACTTTGAATCAACAGCCATCTAATTGCAGACTAATTA

664157_30677280_HHV-7_JI_U43400.1_144861bp_1_326	TATCCTAAGCTTTTCAGGCTATGACTTTGAATCAACAGCCATCTAATTGCAGACTAATTACAGCTAACGATCCAGTACTTGCATCGAATTTTACAATGCAGCCTACTTTTAAAATAGCCG
664157_30677280_HHV-7_JI_U43400.1_144861bp_1_327	CAGCTAACGATCCAGTACTTGCATCGAATTTTACAATGCAGCCTACTTTTAAAATAGCCGATAAGAAAGTTGTACTCAGAGATCATAATTACATTGCAGTCAAAGATTTTGTTCTATCAA
664157_30677280_HHV-7_JI_U43400.1_144861bp_1_328	ATAAGAAAGTTGTACTCAGAGATCATAATTACATTGCAGTCAAAGATTTTGTTCTATCAAGGTCTTTCATGCATTGCATTCGATGTCAGGAAAAAATTGAAAAGAAGACATCCGATAGCA
664157_30677280_HHV-7_JI_U43400.1_144861bp_1_329	GGTCTTTCATGCATTGCATTCGATGTCAGGAAAAAATTGAAAAGAAGACATCCGATAGCATTAGAGCATATTCCATTAAGCCAGATTTCATGTTTTATGACTCAGACGACGAAGTTCGTT
664157_30677280_HHV-7_JI_U43400.1_144861bp_1_330	TTAGAGCATATTCCATTAAGCCAGATTTCATGTTTTATGACTCAGACGACGAAGTTCGTTCGTCACCATTTTTATAACAATATTTAAAGTAGCTGAATTGTACGTTCTGAAACAGTCTGA
664157_30677280_HHV-7_JI_U43400.1_144861bp_1_331	CGTCACCATTTTTATAACAATATTTAAAGTAGCTGAATTGTACGTTCTGAAACAGTCTGAACGGATCCGCTGAACGCAGACACAATGGAACAGCAAAAAGGATTTTCGATTCCATTTTTT
664157_30677280_HHV-7_JI_U43400.1_144861bp_1_332	ACGGATCCGCTGAACGCAGACACAATGGAACAGCAAAAAGGATTTTCGATTCCATTTTTTGTCACTGACGAAAACTGCAACTTTGTGCCTGAAATATTACCTCGTATACATACTAAATTT
664157_30677280_HHV-7_JI_U43400.1_144861bp_1_333	GTCACTGACGAAAACTGCAACTTTGTGCCTGAAATATTACCTCGTATACATACTAAATTTCTTAAAGATGTCTTAATTGCCGATTCCTACAACTCTGTTAGTTGGGCAAACAGTTTTATT
664157_30677280_HHV-7_JI_U43400.1_144861bp_1_334	CTTAAAGATGTCTTAATTGCCGATTCCTACAACTCTGTTAGTTGGGCAAACAGTTTTATTCCCATGCCTATTCAAACGCTTGAACAAATTATGGTTCTTATAACAAAGTTCAAGTTTTCT
664157_30677280_HHV-7_JI_U43400.1_144861bp_1_335	CCCATGCCTATTCAAACGCTTGAACAAATTATGGTTCTTATAACAAAGTTCAAGTTTTCTCGCTCGCGTGATTTTTTATTCCCAGTAATTCGATTAGCTGTTCATATCAATAGGTTCCAC
664157_30677280_HHV-7_JI_U43400.1_144861bp_1_336	CGCTCGCGTGATTTTTTATTCCCAGTAATTCGATTAGCTGTTCATATCAATAGGTTCCACACAGGGAAGAAACAGCTGAAAACCATGATAGAAATTATGAAAAGCTTGTTTAACACCGAA
664157_30677280_HHV-7_JI_U43400.1_144861bp_1_337	ACAGGGAAGAAACAGCTGAAAACCATGATAGAAATTATGAAAAGCTTGTTTAACACCGAAGAGGCTATGCGACGATTCGATGAAGCATTGATGATTCTATTTTCTAATGAGCAAACCAAT

664157_30677280_HHV-7_JI_U43400.1_144861bp_1_338	GAGGCTATGCGACGATTCGATGAAGCATTGATGATTCTATTTTCTAATGAGCAAACCAATACTTACATGACAAACATAGCTTTATCGATGCATGAGAATGGTCTTCCAGATTCAAAATTT
664157_30677280_HHV-7_JI_U43400.1_144861bp_1_339	ACTTACATGACAAACATAGCTTTATCGATGCATGAGAATGGTCTTCCAGATTCAAAATTTATGAATGCTCTAAAAATGATTTACAGAGCTGGAAATTCTTTTGATAATCAACCAGACAAT
664157_30677280_HHV-7_JI_U43400.1_144861bp_1_340	ATGAATGCTCTAAAAATGATTTACAGAGCTGGAAATTCTTTTGATAATCAACCAGACAATGATATAGAAAGCTACAACGAGAAATTAAAAATCTACAACTACCTAATTAAAATACCTAAG
664157_30677280_HHV-7_JI_U43400.1_144861bp_1_341	GATATAGAAAGCTACAACGAGAAATTAAAAATCTACAACTACCTAATTAAAATACCTAAGTACACACTAAAAGCTGGAGTTGATTTATATAATGAAAATATAAAAGATCTTTCGATTGGA
664157_30677280_HHV-7_JI_U43400.1_144861bp_1_342	TACACACTAAAAGCTGGAGTTGATTTATATAATGAAAATATAAAAGATCTTTCGATTGGAATCCAAAGACAACCTACTTTATTATTCACATCTCGTAATGATTTTTCATTGAAAGCTATT
664157_30677280_HHV-7_JI_U43400.1_144861bp_1_343	ATCCAAAGACAACCTACTTTATTATTCACATCTCGTAATGATTTTTCATTGAAAGCTATTTATAATGACGTGTTATTTCTAGTTTCTGCATGGAATATGATCATCAACTATAAAAAAGAA
664157_30677280_HHV-7_JI_U43400.1_144861bp_1_344	TATAATGACGTGTTATTTCTAGTTTCTGCATGGAATATGATCATCAACTATAAAAAAGAACAAAGAAGACTCTTTAGTTGGATAACATTTGAAATAAACTCTTTAATGGAGAATGTTGTG
664157_30677280_HHV-7_JI_U43400.1_144861bp_1_345	CAAAGAAGACTCTTTAGTTGGATAACATTTGAAATAAACTCTTTAATGGAGAATGTTGTGCTTGCAGCCTTTCAGTTACCAGATTTAAAAGAAATGACACTTGATTTAAGCGCTTTGATT
664157_30677280_HHV-7_JI_U43400.1_144861bp_1_346	CTTGCAGCCTTTCAGTTACCAGATTTAAAAGAAATGACACTTGATTTAAGCGCTTTGATTGCCAACATGAATCTTTTAAAACCAAATGATGATTACAGCCCCCATTTTAAACTAATTATC
664157_30677280_HHV-7_JI_U43400.1_144861bp_1_347	GCCAACATGAATCTTTTAAAACCAAATGATGATTACAGCCCCCATTTTAAACTAATTATCAACAAATTTTTTGAAATCGGAATTTTTGTCACGAAATCATATATTTGTATTTTGCCTTCT
664157_30677280_HHV-7_JI_U43400.1_144861bp_1_348	AACAAATTTTTTGAAATCGGAATTTTTGTCACGAAATCATATATTTGTATTTTGCCTTCTTTTGTCAAAAGCCAACTTATTTCGTTTGAAAACGTTTTAAGTTCAAACAGACATGCGGAA
664157_30677280_HHV-7_JI_U43400.1_144861bp_1_349	TTTGTCAAAAGCCAACTTATTTCGTTTGAAAACGTTTTAAGTTCAAACAGACATGCGGAAGATGTGACTTTCATACTAACATCTTCCAAGGAATCTGATGATGAATATGATGAAGATAAA

664157_30677280_HHV-7_JI_U43400.1_144861bp_1_350	GATGTGACTTTCATACTAACATCTTCCAAGGAATCTGATGATGAATATGATGAAGATAAACCTCCACGACAAGTAGATCCAGACAGAGTGGACAACATTTTAATGGAATCAGATTTTTTT
664157_30677280_HHV-7_JI_U43400.1_144861bp_1_351	CCTCCACGACAAGTAGATCCAGACAGAGTGGACAACATTTTAATGGAATCAGATTTTTTTAATGTGAAACCGGAAAACGCCTTTTCAGAAATCGCATTAATGCCAATTTCACATGACAAA
664157_30677280_HHV-7_JI_U43400.1_144861bp_1_352	AATGTGAAACCGGAAAACGCCTTTTCAGAAATCGCATTAATGCCAATTTCACATGACAAAATTATAGATGTCAACAACTCAAACATACAAGTTCTTGAAACTGAATTGGCACATACAAAT
664157_30677280_HHV-7_JI_U43400.1_144861bp_1_353	ATTATAGATGTCAACAACTCAAACATACAAGTTCTTGAAACTGAATTGGCACATACAAATTTATTTGTGTATAGCGCAATAGCTCAAAAATATGATTTGCCTTTAAAAGAATACGTAGAG
664157_30677280_HHV-7_JI_U43400.1_144861bp_1_354	TTATTTGTGTATAGCGCAATAGCTCAAAAATATGATTTGCCTTTAAAAGAATACGTAGAGCGCTTAAACGTCTACAATCCTGATCTATCATCTGGAAACTCTACACCCGCGCGGAATTCT
664157_30677280_HHV-7_JI_U43400.1_144861bp_1_355	CGCTTAAACGTCTACAATCCTGATCTATCATCTGGAAACTCTACACCCGCGCGGAATTCTAATTCTATTCATACAACTCCAGTTCTAAATATATCCAGACCAGGAAGCACTACACCCTCT
664157_30677280_HHV-7_JI_U43400.1_144861bp_1_356	AATTCTATTCATACAACTCCAGTTCTAAATATATCCAGACCAGGAAGCACTACACCCTCTGGGAACTCTGCAAGATATGGGAATAACACACCTAGAAGTATAACTCCGGTTTTAGAGATT
664157_30677280_HHV-7_JI_U43400.1_144861bp_1_357	GGGAACTCTGCAAGATATGGGAATAACACACCTAGAAGTATAACTCCGGTTTTAGAGATTTCAAGATCTAGAAGTGCTACCCCTTCTGGAAATTCTGAAATCTATGAGAATAGAACATCC
664157_30677280_HHV-7_JI_U43400.1_144861bp_1_358	TCAAGATCTAGAAGTGCTACCCCTTCTGGAAATTCTGAAATCTATGAGAATAGAACATCCCCAACGTTCCGTGTTTCTAGGAGTGCAACTCCAATAGAAAGAAGTTCGAGATCTGCTAGT
664157_30677280_HHV-7_JI_U43400.1_144861bp_1_359	CCAACGTTCCGTGTTTCTAGGAGTGCAACTCCAATAGAAAGAAGTTCGAGATCTGCTAGTATAATTTCTGGAGAATCTGTTCCTGGTTTTTTTAATGACCAAGAACGTTTGAGCACCAAT
664157_30677280_HHV-7_JI_U43400.1_144861bp_1_360	ATAATTTCTGGAGAATCTGTTCCTGGTTTTTTTAATGACCAAGAACGTTTGAGCACCAATTCGCCTATTTCCATAAATGGTAATACTCCAAGACAGCAAAGCCATGGGGACAATGAAATA
664157_30677280_HHV-7_JI_U43400.1_144861bp_1_361	TCGCCTATTTCCATAAATGGTAATACTCCAAGACAGCAAAGCCATGGGGACAATGAAATACAAACTATAGATTCCACGGATGAAGACTCAATGAATGCCCCACAATCACCCCAATCCATC

664157_30677280_HHV-7_JI_U43400.1_144861bp_1_362	CAAACTATAGATTCCACGGATGAAGACTCAATGAATGCCCCACAATCACCCCAATCCATCTATTCAATTTCTAGCTATGTTTCAACAGACGATCAACTTCTACATTCACCGACAAATAGC
664157_30677280_HHV-7_JI_U43400.1_144861bp_1_363	TATTCAATTTCTAGCTATGTTTCAACAGACGATCAACTTCTACATTCACCGACAAATAGCCCATTTAATCTTTTTGACTCTGTCTCGGAAATGCAAGAAGACACCGAATAATTAATTGTT
664157_30677280_HHV-7_JI_U43400.1_144861bp_1_364	CCATTTAATCTTTTTGACTCTGTCTCGGAAATGCAAGAAGACACCGAATAATTAATTGTTTTTTATTTAGCTAATTCATTGTATGACAACACAAGGGCAATTGTTTGATTAATTTCCACG
664157_30677280_HHV-7_JI_U43400.1_144861bp_1_365	TTTTATTTAGCTAATTCATTGTATGACAACACAAGGGCAATTGTTTGATTAATTTCCACGTTTTTTAGCTTTTGAATAATCTCAATTAAAACATCTCTGGTCAGTTGGAGTCGTTCTATG
664157_30677280_HHV-7_JI_U43400.1_144861bp_1_366	TTTTTTAGCTTTTGAATAATCTCAATTAAAACATCTCTGGTCAGTTGGAGTCGTTCTATGTATGAAAAAAACATTATCGCGTTTTCATGCTCATCGTCTCCAAGCCTCAGATGACTTTCT
664157_30677280_HHV-7_JI_U43400.1_144861bp_1_367	TATGAAAAAAACATTATCGCGTTTTCATGCTCATCGTCTCCAAGCCTCAGATGACTTTCTTCTAGAAACTAAAGAAAAAAAAAGTTAAAAGATCTTTTACAGAAGAACATTTATTATTTT
664157_30677280_HHV-7_JI_U43400.1_144861bp_1_368	TCTAGAAACTAAAGAAAAAAAAAGTTAAAAGATCTTTTACAGAAGAACATTTATTATTTTTAAAAATGATTTTTCTTACCGCATAAATCATGTTTAAGAAAAGTTGCTTTTCATGCTTTG
664157_30677280_HHV-7_JI_U43400.1_144861bp_1_369	TAAAAATGATTTTTCTTACCGCATAAATCATGTTTAAGAAAAGTTGCTTTTCATGCTTTGTTGTCATCATATACAATAATGCTGTGATTTTCCTGAAAATAAAAGAGCGGGGTTAGACAA
664157_30677280_HHV-7_JI_U43400.1_144861bp_1_370	TTGTCATCATATACAATAATGCTGTGATTTTCCTGAAAATAAAAGAGCGGGGTTAGACAATAAAATTTTGAAACTCGTTTGTGTAGAAATAATAGAGGTGGACTCACCGTTTTACAGGTA
664157_30677280_HHV-7_JI_U43400.1_144861bp_1_371	TAAAATTTTGAAACTCGTTTGTGTAGAAATAATAGAGGTGGACTCACCGTTTTACAGGTATGTGTGTGAATTGAGCGACGATATAACAATCCTCAGTTTTGTGAACATCTAGATTGAAAA
664157_30677280_HHV-7_JI_U43400.1_144861bp_1_372	TGTGTGTGAATTGAGCGACGATATAACAATCCTCAGTTTTGTGAACATCTAGATTGAAAATCTGCTTGATTTCTTGCCATCTCTTAATAATAAACAAAGCTTCAATCACTACAGACTTTG
664157_30677280_HHV-7_JI_U43400.1_144861bp_1_373	TCTGCTTGATTTCTTGCCATCTCTTAATAATAAACAAAGCTTCAATCACTACAGACTTTGTCACAGTTGTGGTTAATGTTAATCCAGAAAAAATAAATCTATATGTTGTATTAAAATCCA

664157_30677280_HHV-7_JI_U43400.1_144861bp_1_374	TCACAGTTGTGGTTAATGTTAATCCAGAAAAAATAAATCTATATGTTGTATTAAAATCCATTTGAAAAAGATATTTTATATAGATTGTCTCCACTTTAGATATAATATTAGATAACGTCA
664157_30677280_HHV-7_JI_U43400.1_144861bp_1_375	TTTGAAAAAGATATTTTATATAGATTGTCTCCACTTTAGATATAATATTAGATAACGTCATCAATATCTGTAGTGGACACAGCTCGCGAAATTCCTGTAGTCGTTGTCTTCTCCAGGTTT
664157_30677280_HHV-7_JI_U43400.1_144861bp_1_376	TCAATATCTGTAGTGGACACAGCTCGCGAAATTCCTGTAGTCGTTGTCTTCTCCAGGTTTCCATTTCTCACAAAGAAGAGTCTACCCTCGTAGAAGAACACTAACTAAATGAATTTTTTT
664157_30677280_HHV-7_JI_U43400.1_144861bp_1_377	CCATTTCTCACAAAGAAGAGTCTACCCTCGTAGAAGAACACTAACTAAATGAATTTTTTTAGGCGAACGCTTAAAAAACTCTTTTACTGGCACACCGCCTATAAAAACCCCCAAAGGAAA
664157_30677280_HHV-7_JI_U43400.1_144861bp_1_378	AGGCGAACGCTTAAAAAACTCTTTTACTGGCACACCGCCTATAAAAACCCCCAAAGGAAATTTTTATGTAACCACACAGAACCAAAAAAAGATTAACCGTTATTAGAAAGTGTTCGTTTT
664157_30677280_HHV-7_JI_U43400.1_144861bp_1_379	TTTTTATGTAACCACACAGAACCAAAAAAAGATTAACCGTTATTAGAAAGTGTTCGTTTTTAACGATTTTTATTGGCACATTTTGACCGGGTTAAATTTCGTCTTCATCAGATGTATCCA
664157_30677280_HHV-7_JI_U43400.1_144861bp_1_380	TAACGATTTTTATTGGCACATTTTGACCGGGTTAAATTTCGTCTTCATCAGATGTATCCACTTCTCTGAAATCCTCACTTAAATATATAGCTAATCCGGCAGCGTGTGGGCAGGTCGGAT
664157_30677280_HHV-7_JI_U43400.1_144861bp_1_381	CTTCTCTGAAATCCTCACTTAAATATATAGCTAATCCGGCAGCGTGTGGGCAGGTCGGATAAATTTCATGAGTAATTGTTAAATTCCTTGGAAAAAATTTGTATGTGCGATATGTTCTAA
664157_30677280_HHV-7_JI_U43400.1_144861bp_1_382	AAATTTCATGAGTAATTGTTAAATTCCTTGGAAAAAATTTGTATGTGCGATATGTTCTAACTATCCCCTGTCTTAATAAACATTCAAAATTTTCTGCAATTTTGACACAGGGACCGATCC
664157_30677280_HHV-7_JI_U43400.1_144861bp_1_383	CTATCCCCTGTCTTAATAAACATTCAAAATTTTCTGCAATTTTGACACAGGGACCGATCCCGGTTCCAATTGCGTCAATACCATAAATAAAACCTTTTAAATCCATCATTAAAAATAAAC
664157_30677280_HHV-7_JI_U43400.1_144861bp_1_384	CGGTTCCAATTGCGTCAATACCATAAATAAAACCTTTTAAATCCATCATTAAAAATAAACTTTTAGGAAACATTAAGTTTTTACCGACGCTGCCAATGATATGTCCATAGTATGGCATAT
664157_30677280_HHV-7_JI_U43400.1_144861bp_1_385	TTTTAGGAAACATTAAGTTTTTACCGACGCTGCCAATGATATGTCCATAGTATGGCATATTGTCATAAACGTCAGCTAGAAAATTCCATTCTGTGCGATTAGGGAATTCTTCTTTTCTGG

664157_30677280_HHV-7_JI_U43400.1_144861bp_1_386	TGTCATAAACGTCAGCTAGAAAATTCCATTCTGTGCGATTAGGGAATTCTTCTTTTCTGGTTAGAAACAAAACTAGTTTTTCATCCATCTTTTCCCTCTTCAAACGAAGAGTGTACACTT
664157_30677280_HHV-7_JI_U43400.1_144861bp_1_387	TTAGAAACAAAACTAGTTTTTCATCCATCTTTTCCCTCTTCAAACGAAGAGTGTACACTTTCCCACTGTTAGCCGCTATTATTTCAGCAAACTTCACAAGATTATACTGTGCATGAATGA
664157_30677280_HHV-7_JI_U43400.1_144861bp_1_388	TCCCACTGTTAGCCGCTATTATTTCAGCAAACTTCACAAGATTATACTGTGCATGAATGAGACTTTGCAAAACGTGATCGATAGACGTTAGGAAGGGAATGTCACTATCTTGATAAATCG
664157_30677280_HHV-7_JI_U43400.1_144861bp_1_389	GACTTTGCAAAACGTGATCGATAGACGTTAGGAAGGGAATGTCACTATCTTGATAAATCGGATCAAAACGGTGCAACCCGAAATAAGCAAAGCGCTCTGTTGATTCAGCAAGCAAATATA
664157_30677280_HHV-7_JI_U43400.1_144861bp_1_390	GATCAAAACGGTGCAACCCGAAATAAGCAAAGCGCTCTGTTGATTCAGCAAGCAAATATAAATTTTCTTTTTCGAAGTCCAAAGCATAGAAACGCTCTTCTTCTCCGAGAAGAATGATGG
664157_30677280_HHV-7_JI_U43400.1_144861bp_1_391	AATTTTCTTTTTCGAAGTCCAAAGCATAGAAACGCTCTTCTTCTCCGAGAAGAATGATGGTTTTAGCAGCTGCTGTTGTTACGTCGTTTTTAGCACTCAGAAAACCGATGACTTGTAACC
664157_30677280_HHV-7_JI_U43400.1_144861bp_1_392	TTTTAGCAGCTGCTGTTGTTACGTCGTTTTTAGCACTCAGAAAACCGATGACTTGTAACCTTTCATTGCAACATAGGAGATAGCAACCTATGAAAACAACAAAAACTTATTAGAACATCA
664157_30677280_HHV-7_JI_U43400.1_144861bp_1_393	TTTCATTGCAACATAGGAGATAGCAACCTATGAAAACAACAAAAACTTATTAGAACATCATGACACATTGGAAATGTTTTGACTGCTTTTGCAACTTACATAGGTCTTGCAGCTCGGTTT
664157_30677280_HHV-7_JI_U43400.1_144861bp_1_394	TGACACATTGGAAATGTTTTGACTGCTTTTGCAACTTACATAGGTCTTGCAGCTCGGTTTCACTAGTAGTTCTGAAAGGCATATCCCGCAAAGGCGAAATGATTATTTTCCTACTCTGAG
664157_30677280_HHV-7_JI_U43400.1_144861bp_1_395	CACTAGTAGTTCTGAAAGGCATATCCCGCAAAGGCGAAATGATTATTTTCCTACTCTGAGGCCATCTTATGAAGATTTCACACCCACTATTTTGAGTAACGAAGTCACTAAGAGAATCGC
664157_30677280_HHV-7_JI_U43400.1_144861bp_1_396	GCCATCTTATGAAGATTTCACACCCACTATTTTGAGTAACGAAGTCACTAAGAGAATCGCGATAAAAGGCTTGTTTAACAAGGCTTAAATTTGCGAGTGCATGCCTAAATCGAACTTTTT
664157_30677280_HHV-7_JI_U43400.1_144861bp_1_397	GATAAAAGGCTTGTTTAACAAGGCTTAAATTTGCGAGTGCATGCCTAAATCGAACTTTTTGTTCACTTTCGGCCATGTTGAAGCACACTCTAAGCTTATGAAAACAGCAGTTGTGACTAG

664157_30677280_HHV-7_JI_U43400.1_144861bp_1_398	GTTCACTTTCGGCCATGTTGAAGCACACTCTAAGCTTATGAAAACAGCAGTTGTGACTAGGTTTTTATATATTACTGAAGCATGACGTCGCTTTATGCTTAAGCTGGAAAGTTCTGAAAA
664157_30677280_HHV-7_JI_U43400.1_144861bp_1_399	GTTTTTATATATTACTGAAGCATGACGTCGCTTTATGCTTAAGCTGGAAAGTTCTGAAAATATACAGCTATCTTAAACAAGTCTGTTTACAATAGGGATTTCCTTTTCCTGTAAATGCTG
664157_30677280_HHV-7_JI_U43400.1_144861bp_1_400	TATACAGCTATCTTAAACAAGTCTGTTTACAATAGGGATTTCCTTTTCCTGTAAATGCTGCCTGATACTAGAAATCATGTCCGTGAATTCCAGTATGGAAATCTGGGAAATTCCAAAATA
664157_30677280_HHV-7_JI_U43400.1_144861bp_1_401	CCTGATACTAGAAATCATGTCCGTGAATTCCAGTATGGAAATCTGGGAAATTCCAAAATATATGATATATAAATTCCTACTTTTCCATGCAGCATCATTAACGCCGAATCGAATATACAC
664157_30677280_HHV-7_JI_U43400.1_144861bp_1_402	TATGATATATAAATTCCTACTTTTCCATGCAGCATCATTAACGCCGAATCGAATATACACATTTTCAGAGCTGCAGTTGACATACTAAAATATGGCTATTAAGATCACCGTATGTCTTCG
664157_30677280_HHV-7_JI_U43400.1_144861bp_1_403	ATTTTCAGAGCTGCAGTTGACATACTAAAATATGGCTATTAAGATCACCGTATGTCTTCGATACTTTAATCGAGATCGTAACGAACTTGGAAACATTACTTTTAATATGGAATACAAAGA
664157_30677280_HHV-7_JI_U43400.1_144861bp_1_404	ATACTTTAATCGAGATCGTAACGAACTTGGAAACATTACTTTTAATATGGAATACAAAGACAGGAGAGTCGCTGTGACATCGAGTTATATTCGGGTACATTTTGTTTTTAATCAGCGTTA
664157_30677280_HHV-7_JI_U43400.1_144861bp_1_405	CAGGAGAGTCGCTGTGACATCGAGTTATATTCGGGTACATTTTGTTTTTAATCAGCGTTAACTGATCAAACTTAGAAATGTTTCCTTTAATACAAAATGCGGAAGAAAAAGCTTACACTT
664157_30677280_HHV-7_JI_U43400.1_144861bp_1_406	ACTGATCAAACTTAGAAATGTTTCCTTTAATACAAAATGCGGAAGAAAAAGCTTACACTTACCTCTTCTTTATATTGAGTAAAAATGGACCGCGGGGGGAACAGATTTTAACAGTTCTAG
664157_30677280_HHV-7_JI_U43400.1_144861bp_1_407	ACCTCTTCTTTATATTGAGTAAAAATGGACCGCGGGGGGAACAGATTTTAACAGTTCTAGATTACACTCGGTTTAAATTTTTTGATGGCTCAGTGACCATTGCACACTCAAAAAAAAATT
664157_30677280_HHV-7_JI_U43400.1_144861bp_1_408	ATTACACTCGGTTTAAATTTTTTGATGGCTCAGTGACCATTGCACACTCAAAAAAAAATTATTTTCTTTCAGGTTCTCTCAGATATAAGAAAGATGATTATGACGTTATTCAAAAACAAA
664157_30677280_HHV-7_JI_U43400.1_144861bp_1_409	ATTTTCTTTCAGGTTCTCTCAGATATAAGAAAGATGATTATGACGTTATTCAAAAACAAAAAGGTAAGCGAATTACATGTAAACATTCAAAATTTATTGTTTGTTAAACATGTAGAAACT

664157_30677280_HHV-7_JI_U43400.1_144861bp_1_410	AAGGTAAGCGAATTACATGTAAACATTCAAAATTTATTGTTTGTTAAACATGTAGAAACTCACAATGTTTTCTCTATGATCCAGCAAAGACTTGCAAAGACAGAAATTGAAATGAGCCTG
664157_30677280_HHV-7_JI_U43400.1_144861bp_1_411	CACAATGTTTTCTCTATGATCCAGCAAAGACTTGCAAAGACAGAAATTGAAATGAGCCTGACAGTCACGTAAAAGTGTTCAAAAAAGATTCCTGATGATAATCGAGAATAAATCAACCAA
664157_30677280_HHV-7_JI_U43400.1_144861bp_1_412	ACAGTCACGTAAAAGTGTTCAAAAAAGATTCCTGATGATAATCGAGAATAAATCAACCAACATTCTTGTCAACTTTGCGCCGATTTACTATCGACAAACAGAAAAACAAAAAAAGTACCA
664157_30677280_HHV-7_JI_U43400.1_144861bp_1_413	CATTCTTGTCAACTTTGCGCCGATTTACTATCGACAAACAGAAAAACAAAAAAAGTACCATTGCTGATCCGCCGACAAGAAACACTACACCAATAGTTGTCGCTTGGGTTTGATCGAGGA
664157_30677280_HHV-7_JI_U43400.1_144861bp_1_414	TTGCTGATCCGCCGACAAGAAACACTACACCAATAGTTGTCGCTTGGGTTTGATCGAGGAAGCTTTCGTAATCATTCAAGAAGATGACATTTTTACCTCCATATGAAGCGCTGAGATCTT
664157_30677280_HHV-7_JI_U43400.1_144861bp_1_415	AGCTTTCGTAATCATTCAAGAAGATGACATTTTTACCTCCATATGAAGCGCTGAGATCTTCCAATTCTTTGTACTTGTACCAATCAAGCCACGATGACTTCCAAGTTTTGCGCAAAAATC
664157_30677280_HHV-7_JI_U43400.1_144861bp_1_416	CCAATTCTTTGTACTTGTACCAATCAAGCCACGATGACTTCCAAGTTTTGCGCAAAAATCTTTTATAAATAGTTTCATTGCGTACTATTGTAAAGTTTGGATTTAAGACTTTCCTTTGAT
664157_30677280_HHV-7_JI_U43400.1_144861bp_1_417	TTTTATAAATAGTTTCATTGCGTACTATTGTAAAGTTTGGATTTAAGACTTTCCTTTGATATATTTCATCAGATAAATGAGGACATATCATTGCTACGAAATAACAATCATAATTAAATT
664157_30677280_HHV-7_JI_U43400.1_144861bp_1_418	ATATTTCATCAGATAAATGAGGACATATCATTGCTACGAAATAACAATCATAATTAAATTTTTGATTTAGAATTTCAAGTGTTTTGTTTTTATCTTTTCCGACGCTTCTCCACTTAACTG
664157_30677280_HHV-7_JI_U43400.1_144861bp_1_419	TTTGATTTAGAATTTCAAGTGTTTTGTTTTTATCTTTTCCGACGCTTCTCCACTTAACTGCAACTATGCCAGATCTTAGCTGCAAAAGAAACTTTTGACCCCCTGTCATAATAGTGATGG
664157_30677280_HHV-7_JI_U43400.1_144861bp_1_420	CAACTATGCCAGATCTTAGCTGCAAAAGAAACTTTTGACCCCCTGTCATAATAGTGATGGAACCACCCGAATTATGACCTTCTAACCAACATTCAAGTGTACTGTTAGCCCGAGAAGCAA
664157_30677280_HHV-7_JI_U43400.1_144861bp_1_421	AACCACCCGAATTATGACCTTCTAACCAACATTCAAGTGTACTGTTAGCCCGAGAAGCAAAGTGCCATTTTTTGGCAATTAGTGCTCTTAACATAACTCCTCTAAAGTAATAATTGATCA

664157_30677280_HHV-7_JI_U43400.1_144861bp_1_422	AGTGCCATTTTTTGGCAATTAGTGCTCTTAACATAACTCCTCTAAAGTAATAATTGATCAGCACTCTCAAATAGGTTAATTTATGAGATGTTGTAGTAAGATTTACTAGACTCATTTCGC
664157_30677280_HHV-7_JI_U43400.1_144861bp_1_423	GCACTCTCAAATAGGTTAATTTATGAGATGTTGTAGTAAGATTTACTAGACTCATTTCGCGAAATCCTTCAGTTTTTAAAGACAAAGAAATCAGAAAAGCTGTTCTTACAGATAAACAAC
664157_30677280_HHV-7_JI_U43400.1_144861bp_1_424	GAAATCCTTCAGTTTTTAAAGACAAAGAAATCAGAAAAGCTGTTCTTACAGATAAACAACTAGAATTCCCTATTAGCATTTCATTAACATAACACGTGGTGACACAAACAGCTTCTAATT
664157_30677280_HHV-7_JI_U43400.1_144861bp_1_425	TAGAATTCCCTATTAGCATTTCATTAACATAACACGTGGTGACACAAACAGCTTCTAATTTATTATCTAGAAATATTATATTCTGGTGACATTTCAAATTTGACATGAGTGTGAGAGATA
664157_30677280_HHV-7_JI_U43400.1_144861bp_1_426	TATTATCTAGAAATATTATATTCTGGTGACATTTCAAATTTGACATGAGTGTGAGAGATAAGCAAATCAAAAAGACAAAAAACATCATCTCTTTTCCCATGGTCCTTTATAAAGACTTCA
664157_30677280_HHV-7_JI_U43400.1_144861bp_1_427	AGCAAATCAAAAAGACAAAAAACATCATCTCTTTTCCCATGGTCCTTTATAAAGACTTCACAATTTCTTCAAGAAAGTCCACGGTACTTCTATAAAACAAAAAAAAACACAATTAACTAT
664157_30677280_HHV-7_JI_U43400.1_144861bp_1_428	CAATTTCTTCAAGAAAGTCCACGGTACTTCTATAAAACAAAAAAAAACACAATTAACTATTGATGAACATTGCAGCATTTTTCCAATGCATTGCCATACATACCCCGAAGAAATTTTTCC
664157_30677280_HHV-7_JI_U43400.1_144861bp_1_429	TGATGAACATTGCAGCATTTTTCCAATGCATTGCCATACATACCCCGAAGAAATTTTTCCTTTGAAAAGATACTCTAGATGTGAAAATTGTGTTGAAGAAAGAAGTTCCGTCTTAGTGGA
664157_30677280_HHV-7_JI_U43400.1_144861bp_1_430	TTTGAAAAGATACTCTAGATGTGAAAATTGTGTTGAAGAAAGAAGTTCCGTCTTAGTGGACATACATTCAAACGCAGGCTCAGGGAGTTTTTGAATGGTTGATATTCTGCTATCTTGTTC
664157_30677280_HHV-7_JI_U43400.1_144861bp_1_431	CATACATTCAAACGCAGGCTCAGGGAGTTTTTGAATGGTTGATATTCTGCTATCTTGTTCTTTCCCCCTTTAAATGGCCATAAAAAAGAAATTATCAGTTAAAGACAGCGAAAAATTTAA
664157_30677280_HHV-7_JI_U43400.1_144861bp_1_432	TTTCCCCCTTTAAATGGCCATAAAAAAGAAATTATCAGTTAAAGACAGCGAAAAATTTAAAATAAGTAAAAAACAATAAGGCACCTACTCGGTAAATTGTTCGAAATTCACCGGGAAACC
664157_30677280_HHV-7_JI_U43400.1_144861bp_1_433	AATAAGTAAAAAACAATAAGGCACCTACTCGGTAAATTGTTCGAAATTCACCGGGAAACCCGAAGTTGAAGTGTGTTCCAAGTTTTTCAGTGGCGAGTTTGCTATTTCTTCATAGGTTTC

664157_30677280_HHV-7_JI_U43400.1_144861bp_1_434	CGAAGTTGAAGTGTGTTCCAAGTTTTTCAGTGGCGAGTTTGCTATTTCTTCATAGGTTTCCAATGAGAAGTTTGAACTCTCTTCTAGCATATCTAAAGGTGCTGTCTCTGTCTCGTTAGC
664157_30677280_HHV-7_JI_U43400.1_144861bp_1_435	CAATGAGAAGTTTGAACTCTCTTCTAGCATATCTAAAGGTGCTGTCTCTGTCTCGTTAGCTGTGTTTACCTCTTTATAGGGATACATTTCCGCTGTGGTTTGCGGTTTGTAACACCGTTC
664157_30677280_HHV-7_JI_U43400.1_144861bp_1_436	TGTGTTTACCTCTTTATAGGGATACATTTCCGCTGTGGTTTGCGGTTTGTAACACCGTTCTTTAAAAAAAATGTCATTTTGTGATGTTTGAGTACTGCGAGTAAAATGTCCGAGTCCCGT
664157_30677280_HHV-7_JI_U43400.1_144861bp_1_437	TTTAAAAAAAATGTCATTTTGTGATGTTTGAGTACTGCGAGTAAAATGTCCGAGTCCCGTTTGCGTAGATCTGGACACTAAAATTGTCACACCAGGTGTATGACACATTTTGTAAATCTT
664157_30677280_HHV-7_JI_U43400.1_144861bp_1_438	TTGCGTAGATCTGGACACTAAAATTGTCACACCAGGTGTATGACACATTTTGTAAATCTTGCAATTATTTGGAAGATTGTCATTTTCTGAGTATCCTATGGACATATACATTTTTTCTGC
664157_30677280_HHV-7_JI_U43400.1_144861bp_1_439	GCAATTATTTGGAAGATTGTCATTTTCTGAGTATCCTATGGACATATACATTTTTTCTGCCTGTGTCTCTCTATCATTCGAAAGCTTGTGACCAGGAAGAACTCTTTGAAATCGCGCAGG
664157_30677280_HHV-7_JI_U43400.1_144861bp_1_440	CTGTGTCTCTCTATCATTCGAAAGCTTGTGACCAGGAAGAACTCTTTGAAATCGCGCAGGTAGTTTTCCACTAATATTGATTGTTTTTTGTCGTATTCGATGATAGTAATTGATAAATCT
664157_30677280_HHV-7_JI_U43400.1_144861bp_1_441	TAGTTTTCCACTAATATTGATTGTTTTTTGTCGTATTCGATGATAGTAATTGATAAATCTTTCATGGCATTCATCTCTCGACATCTTCTTTGACATGCCATGTGTAATTGCATTTTGACA
664157_30677280_HHV-7_JI_U43400.1_144861bp_1_442	TTCATGGCATTCATCTCTCGACATCTTCTTTGACATGCCATGTGTAATTGCATTTTGACAGCTAAATTCCAATTTCGTGGTCCAGAAATCTTCAAATGTGGGCGATAAATAGAACATCAC
664157_30677280_HHV-7_JI_U43400.1_144861bp_1_443	GCTAAATTCCAATTTCGTGGTCCAGAAATCTTCAAATGTGGGCGATAAATAGAACATCACACCATCTTCATAAGCAAACACATGCTGAGACAAGGACAGTAAAAAAACTATTTGTCTTCG
664157_30677280_HHV-7_JI_U43400.1_144861bp_1_444	ACCATCTTCATAAGCAAACACATGCTGAGACAAGGACAGTAAAAAAACTATTTGTCTTCGCGGCGTCCCAGAAGCTGACACCCATTCTTCGGCAACCCCAAACATCACCATCTTTCCCTC
664157_30677280_HHV-7_JI_U43400.1_144861bp_1_445	CGGCGTCCCAGAAGCTGACACCCATTCTTCGGCAACCCCAAACATCACCATCTTTCCCTCAAATGGATAAAATCCGATGTTAACTAAACGTTTAAGTCTCCTTTCTGGAAGAATGTCAGT

664157_30677280_HHV-7_JI_U43400.1_144861bp_1_446	AAATGGATAAAATCCGATGTTAACTAAACGTTTAAGTCTCCTTTCTGGAAGAATGTCAGTCATATCACAAATTCGAATATTGATACACATTATGTGTTGAATGGACACGCCATCCTTCAT
664157_30677280_HHV-7_JI_U43400.1_144861bp_1_447	CATATCACAAATTCGAATATTGATACACATTATGTGTTGAATGGACACGCCATCCTTCATTATTTCTTGAACTGTTTTTTCAAAAAACTTTAAATCTTTCGAAGCTGCAATTAATTTTTC
664157_30677280_HHV-7_JI_U43400.1_144861bp_1_448	TATTTCTTGAACTGTTTTTTCAAAAAACTTTAAATCTTTCGAAGCTGCAATTAATTTTTCTTTGCAGCGCAGCATCGTCGTTAAGCCACATTCCTTAGACATAATCGTCTCTTCATAAAA
664157_30677280_HHV-7_JI_U43400.1_144861bp_1_449	TTTGCAGCGCAGCATCGTCGTTAAGCCACATTCCTTAGACATAATCGTCTCTTCATAAAAAATACTGAATTTTAAGCGTATATATTCAACCCCCCGGATATGGGCGTGGAAATTAATAAA
664157_30677280_HHV-7_JI_U43400.1_144861bp_1_450	AATACTGAATTTTAAGCGTATATATTCAACCCCCCGGATATGGGCGTGGAAATTAATAAAAACACTTCAAAAAGCAAACTTTTTATTGAAACTCATTAATGACACATGAAATCTAAGGTC
664157_30677280_HHV-7_JI_U43400.1_144861bp_1_451	AACACTTCAAAAAGCAAACTTTTTATTGAAACTCATTAATGACACATGAAATCTAAGGTCCCGTGAATTTGAAGATCTGGAGAATCCTTAGACAGGCAGTCTGAATCTGAAATTTCCATT
664157_30677280_HHV-7_JI_U43400.1_144861bp_1_452	CCGTGAATTTGAAGATCTGGAGAATCCTTAGACAGGCAGTCTGAATCTGAAATTTCCATTTTTTCAGTTGCACACAAATTTTCCACAGATATATAATCACAGGGATCAATTTCCATGATT
664157_30677280_HHV-7_JI_U43400.1_144861bp_1_453	TTTTCAGTTGCACACAAATTTTCCACAGATATATAATCACAGGGATCAATTTCCATGATTACTTTAACACTATCAGATCTATCAAGGTCCTTACGCTTATTAAGAACAGCCATAACATGC
664157_30677280_HHV-7_JI_U43400.1_144861bp_1_454	ACTTTAACACTATCAGATCTATCAAGGTCCTTACGCTTATTAAGAACAGCCATAACATGCAGCCAAATTTCAAAAAACATTTTTTTAACAGTGATTATAGAAAAAATAAAGGCAAGTATA
664157_30677280_HHV-7_JI_U43400.1_144861bp_1_455	AGCCAAATTTCAAAAAACATTTTTTTAACAGTGATTATAGAAAAAATAAAGGCAAGTATAGGAATGATTAAAAGTATCCAAAAAACTTCAAAGTAATTCCCGTGTTTGTAAGTTTTGTGA
664157_30677280_HHV-7_JI_U43400.1_144861bp_1_456	GGAATGATTAAAAGTATCCAAAAAACTTCAAAGTAATTCCCGTGTTTGTAAGTTTTGTGAAACAAATTTTGAAAATATTCATCTGAATTATTAAATTCAAGGACGTTAAAAGAGTAATTA
664157_30677280_HHV-7_JI_U43400.1_144861bp_1_457	AACAAATTTTGAAAATATTCATCTGAATTATTAAATTCAAGGACGTTAAAAGAGTAATTACATTTTTGATGAATTACCACACATTGATAATTAGCGGACTCCGTGTTAATAGATTTAAAA

664157_30677280_HHV-7_JI_U43400.1_144861bp_1_458	CATTTTTGATGAATTACCACACATTGATAATTAGCGGACTCCGTGTTAATAGATTTAAAAAAAAAGTAGAATTTTTTCCCTTTTAAGATGTCCGATGCCAAATATTCTTCTGTCAAATCA
664157_30677280_HHV-7_JI_U43400.1_144861bp_1_459	AAAAAGTAGAATTTTTTCCCTTTTAAGATGTCCGATGCCAAATATTCTTCTGTCAAATCATCAGGAACCAGTTTATTTTCTAGCTTCCATTGAATACTCAATCCCAGTGGACTATAAAGC
664157_30677280_HHV-7_JI_U43400.1_144861bp_1_460	TCAGGAACCAGTTTATTTTCTAGCTTCCATTGAATACTCAATCCCAGTGGACTATAAAGCTGCGCCGAACACGTTATGTTAGATCCACGTTTTGTGATGGACACCACAGGTGGCCGATAT
664157_30677280_HHV-7_JI_U43400.1_144861bp_1_461	TGCGCCGAACACGTTATGTTAGATCCACGTTTTGTGATGGACACCACAGGTGGCCGATATCCATCTTGACCGAAGGTCACATGTGCAGAAAAATCTGCGAGTTGTTTAACACAAAACTGC
664157_30677280_HHV-7_JI_U43400.1_144861bp_1_462	CCATCTTGACCGAAGGTCACATGTGCAGAAAAATCTGCGAGTTGTTTAACACAAAACTGCTGCACATAAAATTTGAGAATAAGTAATAATTCAACATTAGCATACATGTTTTCGTCAAAA
664157_30677280_HHV-7_JI_U43400.1_144861bp_1_463	TGCACATAAAATTTGAGAATAAGTAATAATTCAACATTAGCATACATGTTTTCGTCAAAACCACTGCTAGAAAAATCCAACAGGTTGACATTCAGTGTCGTGTCATTATGAACTTTTAAC
664157_30677280_HHV-7_JI_U43400.1_144861bp_1_464	CCACTGCTAGAAAAATCCAACAGGTTGACATTCAGTGTCGTGTCATTATGAACTTTTAACAAATAATGACTTTTCAAAACGGTGTCAATTTCACAAATGAATTCAGCGTTAAAATTGTGG
664157_30677280_HHV-7_JI_U43400.1_144861bp_1_465	AAATAATGACTTTTCAAAACGGTGTCAATTTCACAAATGAATTCAGCGTTAAAATTGTGGTGATAATGAACACTGCGATTCCTATGTGACCAGACAAAATAAGAGAAAATGCTGTTTAAT
664157_30677280_HHV-7_JI_U43400.1_144861bp_1_466	TGATAATGAACACTGCGATTCCTATGTGACCAGACAAAATAAGAGAAAATGCTGTTTAATGTAGATTCGTCCTTGTTGGTAGAAAAGACGTAAGACAAAGCATTAAATTTCGTTGGAAAA
664157_30677280_HHV-7_JI_U43400.1_144861bp_1_467	GTAGATTCGTCCTTGTTGGTAGAAAAGACGTAAGACAAAGCATTAAATTTCGTTGGAAAATCTGTGAGGAAAGACGAAATACTAGTATTGCCGACAGACAGATTCTTATAATGTGCCAGT
664157_30677280_HHV-7_JI_U43400.1_144861bp_1_468	TCTGTGAGGAAAGACGAAATACTAGTATTGCCGACAGACAGATTCTTATAATGTGCCAGTTCATAGTTATCCATCATCAATTCATATTTCTCAAGATGTTCTTTATTGATTATTGACATT
664157_30677280_HHV-7_JI_U43400.1_144861bp_1_469	TCATAGTTATCCATCATCAATTCATATTTCTCAAGATGTTCTTTATTGATTATTGACATTGTCAACTTATGTGCCACATATTTGGTAAAATGTACAATGAAAACTAAAAGACATGTTTTT

664157_30677280_HHV-7_JI_U43400.1_144861bp_1_470	GTCAACTTATGTGCCACATATTTGGTAAAATGTACAATGAAAACTAAAAGACATGTTTTTTTCACAAACATTGCTGTTTGAGAGATTCTTTTTTAGGTAACGGTTCTTGTCCAAACATCA
664157_30677280_HHV-7_JI_U43400.1_144861bp_1_471	TTCACAAACATTGCTGTTTGAGAGATTCTTTTTTAGGTAACGGTTCTTGTCCAAACATCAAGTTTGTTAGATTGATTATTTTCTCTGATACTGGTTCTTTTTGTATGTGCTGTAAAGGCC
664157_30677280_HHV-7_JI_U43400.1_144861bp_1_472	AGTTTGTTAGATTGATTATTTTCTCTGATACTGGTTCTTTTTGTATGTGCTGTAAAGGCCACGTCAGAACTTTCTGAACACCGTCTAAACAGGAAACTCCTAATAAAACTACCGCTGTAA
664157_30677280_HHV-7_JI_U43400.1_144861bp_1_473	ACGTCAGAACTTTCTGAACACCGTCTAAACAGGAAACTCCTAATAAAACTACCGCTGTAACAAATGCAAGGAATAATATAGCAATCAATGTGTAGCAAAGCATGTCTACGAAAACATTAC
664157_30677280_HHV-7_JI_U43400.1_144861bp_1_474	CAAATGCAAGGAATAATATAGCAATCAATGTGTAGCAAAGCATGTCTACGAAAACATTACTAGACGTTTTTTCACTATCTAATAGATCCAAATCCACATCAGGTAATGTTACCTGTATTT
664157_30677280_HHV-7_JI_U43400.1_144861bp_1_475	TAGACGTTTTTTCACTATCTAATAGATCCAAATCCACATCAGGTAATGTTACCTGTATTTGTGTTCCTGTGTTCTTATTCGTACAGGTACAAACATATGTGGTCATTTCTACTCTTAGAA
664157_30677280_HHV-7_JI_U43400.1_144861bp_1_476	GTGTTCCTGTGTTCTTATTCGTACAGGTACAAACATATGTGGTCATTTCTACTCTTAGAAAAATTAGACCCCAAAGCACATCACTACCATACAAAGGAGTAAAAGCACCCGTAGAGTTGA
664157_30677280_HHV-7_JI_U43400.1_144861bp_1_477	AAATTAGACCCCAAAGCACATCACTACCATACAAAGGAGTAAAAGCACCCGTAGAGTTGAAACCAACTCTTTTCCACTCTTCTCCAAACGGACTTTTTATCTTTTGCAGAACTTCTAAGG
664157_30677280_HHV-7_JI_U43400.1_144861bp_1_478	AACCAACTCTTTTCCACTCTTCTCCAAACGGACTTTTTATCTTTTGCAGAACTTCTAAGGAGCCGAAACCGCTAACACCCCATCCTGTATATTTACAAACTGCTACCTTTACACCATTCT
664157_30677280_HHV-7_JI_U43400.1_144861bp_1_479	AGCCGAAACCGCTAACACCCCATCCTGTATATTTACAAACTGCTACCTTTACACCATTCTGTCTAAATTTCGAAAATGAACAATCTATGTTATTAGTTCTATATCGTTGTTCATTTCTTT
664157_30677280_HHV-7_JI_U43400.1_144861bp_1_480	GTCTAAATTTCGAAAATGAACAATCTATGTTATTAGTTCTATATCGTTGTTCATTTCTTTGTTCAATCATAGGATTAAAGCCTTGCTTAAATGTAGCTTCGAATGTTTTCGCGAGTGATA
664157_30677280_HHV-7_JI_U43400.1_144861bp_1_481	GTTCAATCATAGGATTAAAGCCTTGCTTAAATGTAGCTTCGAATGTTTTCGCGAGTGATAGGTACTTTCTTTGCCAAAAAAAAAGTTGATTGAAGTCATTTTCACAAGAATTGATCAGAA

664157_30677280_HHV-7_JI_U43400.1_144861bp_1_482	GGTACTTTCTTTGCCAAAAAAAAAGTTGATTGAAGTCATTTTCACAAGAATTGATCAGAAAGTTTTTAGCGTGTGTCAAAGTGTGCCAAATTCGATAAGTGAAGGGATCATCTTCACCTA
664157_30677280_HHV-7_JI_U43400.1_144861bp_1_483	AGTTTTTAGCGTGTGTCAAAGTGTGCCAAATTCGATAAGTGAAGGGATCATCTTCACCTAAATAGTTTTTAATCGCTACCCACGTGCTGTTTCTTTTATTTTGCAAGTAGAATGTGGTTT
664157_30677280_HHV-7_JI_U43400.1_144861bp_1_484	AATAGTTTTTAATCGCTACCCACGTGCTGTTTCTTTTATTTTGCAAGTAGAATGTGGTTTCAAGAATTCTAGGAGAAATTCCAGTTTCATTCATACGTCTCAGACGGATAAAACTTTTAA
664157_30677280_HHV-7_JI_U43400.1_144861bp_1_485	CAAGAATTCTAGGAGAAATTCCAGTTTCATTCATACGTCTCAGACGGATAAAACTTTTAAAGGGTGAACAGATAATTTGAATATGAATGTAAGACTCGACATCAAGACAGAGTTTATCAT
664157_30677280_HHV-7_JI_U43400.1_144861bp_1_486	AGGGTGAACAGATAATTTGAATATGAATGTAAGACTCGACATCAAGACAGAGTTTATCATATCGCTTCATCAGTCCTTTTGTTTTTTTAAAGTTCTTAAACAAAAAATGATTTAGTGTAG
664157_30677280_HHV-7_JI_U43400.1_144861bp_1_487	ATCGCTTCATCAGTCCTTTTGTTTTTTTAAAGTTCTTAAACAAAAAATGATTTAGTGTAGAAAATACTTGATAACAGTTGAAAGCCAAATATTTGCCAAAATCGGTTTCAATTTGCGTTT
664157_30677280_HHV-7_JI_U43400.1_144861bp_1_488	AAAATACTTGATAACAGTTGAAAGCCAAATATTTGCCAAAATCGGTTTCAATTTGCGTTTCGCTGTTGCCAAAAAAGAAAGCATTGTCTGTCATTGCCGCGACTGCAGAGTGTCTGATTT
664157_30677280_HHV-7_JI_U43400.1_144861bp_1_489	CGCTGTTGCCAAAAAAGAAAGCATTGTCTGTCATTGCCGCGACTGCAGAGTGTCTGATTTTATCGTCTGAACATTGTTTGCTCAAAGATATGTCGAACAATAGCAAATCATCCACTGTAG
664157_30677280_HHV-7_JI_U43400.1_144861bp_1_490	TATCGTCTGAACATTGTTTGCTCAAAGATATGTCGAACAATAGCAAATCATCCACTGTAGCATTCACATCAAAATCCACAACTGCTAAGGGACAGAAATCCGGATAGAGTTCTCCATAAA
664157_30677280_HHV-7_JI_U43400.1_144861bp_1_491	CATTCACATCAAAATCCACAACTGCTAAGGGACAGAAATCCGGATAGAGTTCTCCATAAATCACAGGAACGCAAAACAGCAAGATAGTCCACATGTTGAATTAGAAAAGAGTGATGAGAA
664157_30677280_HHV-7_JI_U43400.1_144861bp_1_492	TCACAGGAACGCAAAACAGCAAGATAGTCCACATGTTGAATTAGAAAAGAGTGATGAGAAAGAAAGCTTTTATTTCTTAATATATATTTGACGACTGAACGTCACAGTTACGCCCATTTT
664157_30677280_HHV-7_JI_U43400.1_144861bp_1_493	AGAAAGCTTTTATTTCTTAATATATATTTGACGACTGAACGTCACAGTTACGCCCATTTTACGGTGATAAACGTTAGGGGTTCCGCAAAAATGTGTGCATTGATCTTTCTGTGATGACAC

664157_30677280_HHV-7_JI_U43400.1_144861bp_1_494	ACGGTGATAAACGTTAGGGGTTCCGCAAAAATGTGTGCATTGATCTTTCTGTGATGACACAGTGGTTAGCAAAGCACATCTAACATAATAAGTCTGTTTTTCTCAGTCATAGGATTCCAC
664157_30677280_HHV-7_JI_U43400.1_144861bp_1_495	AGTGGTTAGCAAAGCACATCTAACATAATAAGTCTGTTTTTCTCAGTCATAGGATTCCACAACAGGATAAGACATTTGTGCTGGTCTGATTCCTAATTACATGAGCTGTATTTACGCCAG
664157_30677280_HHV-7_JI_U43400.1_144861bp_1_496	AACAGGATAAGACATTTGTGCTGGTCTGATTCCTAATTACATGAGCTGTATTTACGCCAGAATTTTGTTTATTTCCTTTAATTACCCTCAAAAAATGAAAACATTGATGAAACATACTTC
664157_30677280_HHV-7_JI_U43400.1_144861bp_1_497	AATTTTGTTTATTTCCTTTAATTACCCTCAAAAAATGAAAACATTGATGAAACATACTTCCCAGTGAAACGCTTTCAACGGAGATCATGCGTTGATGGTAGATAAAACACAACAAGGAGA
664157_30677280_HHV-7_JI_U43400.1_144861bp_1_498	CCAGTGAAACGCTTTCAACGGAGATCATGCGTTGATGGTAGATAAAACACAACAAGGAGAACTTTGCCCAAAAAAACATTTATTAATCCCAGACACCAATAAAGGCTTGGTTATTGTATG
664157_30677280_HHV-7_JI_U43400.1_144861bp_1_499	ACTTTGCCCAAAAAAACATTTATTAATCCCAGACACCAATAAAGGCTTGGTTATTGTATGCATTTTTCGGTTTCTTAGAATGGTTGCGTTTGGGGTTTTTAATTAGTAAAACTAACAACG
664157_30677280_HHV-7_JI_U43400.1_144861bp_1_500	CATTTTTCGGTTTCTTAGAATGGTTGCGTTTGGGGTTTTTAATTAGTAAAACTAACAACGCTAACGCTACAATACCAATAAAACTAACTGCTGCAATTAAAATCACAAGGAACAAAACGA
664157_30677280_HHV-7_JI_U43400.1_144861bp_1_501	CTAACGCTACAATACCAATAAAACTAACTGCTGCAATTAAAATCACAAGGAACAAAACGAACTTTTTTAAAGGGCTATTCAAAAGTTGAGGTTTTGTCAGTTCTGGAGTTGAAATTGTCA
664157_30677280_HHV-7_JI_U43400.1_144861bp_1_502	ACTTTTTTAAAGGGCTATTCAAAAGTTGAGGTTTTGTCAGTTCTGGAGTTGAAATTGTCAGTGTGAAAAATTCAGATGCATTCCCACTGTTTTCATGATTAGTAGTACTGCCACTGGAGA
664157_30677280_HHV-7_JI_U43400.1_144861bp_1_503	GTGTGAAAAATTCAGATGCATTCCCACTGTTTTCATGATTAGTAGTACTGCCACTGGAGAATGTCGAGGTCTTTGTAAAATTTAAGGATGTAGATTCGACATTCTCTTTAGTTGTTGCGA
664157_30677280_HHV-7_JI_U43400.1_144861bp_1_504	ATGTCGAGGTCTTTGTAAAATTTAAGGATGTAGATTCGACATTCTCTTTAGTTGTTGCGATAGTCGTAGGTATACTCATAGACACAGACATTAAATCGCTCGTACTCTCTGTATCTGATG
664157_30677280_HHV-7_JI_U43400.1_144861bp_1_505	TAGTCGTAGGTATACTCATAGACACAGACATTAAATCGCTCGTACTCTCTGTATCTGATGTGATTGACGTGGAACTAAAATCTGAAGTTCTTGACATTTCCAGAGTGGGCGTACTAGAAA

664157_30677280_HHV-7_JI_U43400.1_144861bp_1_506	TGATTGACGTGGAACTAAAATCTGAAGTTCTTGACATTTCCAGAGTGGGCGTACTAGAAACAGTACTCATGTTAACAAAGGTAACCATTTCTAAAAAAAATAAAATTCCAAATAACATTT
664157_30677280_HHV-7_JI_U43400.1_144861bp_1_507	CAGTACTCATGTTAACAAAGGTAACCATTTCTAAAAAAAATAAAATTCCAAATAACATTTTAAACACTGCGCAATTAGAAGAAGCCTAGTTTAATATTGTACGTGTGCCGACTAAATTAC
664157_30677280_HHV-7_JI_U43400.1_144861bp_1_508	TAAACACTGCGCAATTAGAAGAAGCCTAGTTTAATATTGTACGTGTGCCGACTAAATTACTGTTTTTAAGCAACGCAAAAAAGTGCTGGAATCGGTGTAAAATTCAGGGCTTCGTACCCC
664157_30677280_HHV-7_JI_U43400.1_144861bp_1_509	TGTTTTTAAGCAACGCAAAAAAGTGCTGGAATCGGTGTAAAATTCAGGGCTTCGTACCCCCATATCGAACATTGAAGATGTGTAGAATAAGAATCAGACATAAGATAACCGATATTATGA
664157_30677280_HHV-7_JI_U43400.1_144861bp_1_510	CATATCGAACATTGAAGATGTGTAGAATAAGAATCAGACATAAGATAACCGATATTATGAGACATGCCAGTATTATAAAAGTCCCTCTTCGGCGCACATTTTTGATTTCAGAACTAGAAG
664157_30677280_HHV-7_JI_U43400.1_144861bp_1_511	GACATGCCAGTATTATAAAAGTCCCTCTTCGGCGCACATTTTTGATTTCAGAACTAGAAGTTGAATTTATTGTTCTTGGTGATAGATTTTCCTGATGCAAAAAAACGGAATGATCGTGGA
664157_30677280_HHV-7_JI_U43400.1_144861bp_1_512	TTGAATTTATTGTTCTTGGTGATAGATTTTCCTGATGCAAAAAAACGGAATGATCGTGGAACATTTGCAACATTACATCATTATAAGAAGGTGGCGGTGTCTCATGAGTCATCTTCTAGG
664157_30677280_HHV-7_JI_U43400.1_144861bp_1_513	ACATTTGCAACATTACATCATTATAAGAAGGTGGCGGTGTCTCATGAGTCATCTTCTAGGGATATTTAGAGCACCTGATAAATGATAACATATTCTCTGCTATTGAAGGACTTATATAAT
664157_30677280_HHV-7_JI_U43400.1_144861bp_1_514	GATATTTAGAGCACCTGATAAATGATAACATATTCTCTGCTATTGAAGGACTTATATAATCATGATATGCAATGCAAGCCAAAATAAACAGAACAAACAAATAATAAATCCCTTTCCAAC
664157_30677280_HHV-7_JI_U43400.1_144861bp_1_515	CATGATATGCAATGCAAGCCAAAATAAACAGAACAAACAAATAATAAATCCCTTTCCAACAATCTTCATAGGTCTCTGGGAAAAAAATGGTTGTACTTATAAAACATTATGTGCAGAATTA
664157_30677280_HHV-7_JI_U43400.1_144861bp_1_516	AATCTTCATAGGTCTCTGGGAAAAAAATGGTTGTACTTATAAAACATTATGTGCAGAATTATTTTTTCTACCAACACTTTTCCATAGTAATGGTTGTTTAAATATGTTCCTTTTTTTCCTT
664157_30677280_HHV-7_JI_U43400.1_144861bp_1_517	TTTTTTCTACCAACACTTTTCCATAGTAATGGTTGTTTAAATATGTTCCTTTTTTTCCTTTTACAAGTATTTCCCCATGGACAGTTGATCTGCCGTGAAAGCTCTTCTTCCACATTTCTA

664157_30677280_HHV-7_JI_U43400.1_144861bp_1_518	TTACAAGTATTTCCCCATGGACAGTTGATCTGCCGTGAAAGCTCTTCTTCCACATTTCTAACATCGTTTGTTCTTTCAAACCAATACCCATGTAGTCGACGATTTTCATTAAATCGTAAA
664157_30677280_HHV-7_JI_U43400.1_144861bp_1_519	ACATCGTTTGTTCTTTCAAACCAATACCCATGTAGTCGACGATTTTCATTAAATCGTAAACAACCGATTTTCAAAAATTCAGTTATGTTATGTGCGAGAAAAATGATTTGTTCATCATCT
664157_30677280_HHV-7_JI_U43400.1_144861bp_1_520	CAACCGATTTTCAAAAATTCAGTTATGTTATGTGCGAGAAAAATGATTTGTTCATCATCTACACCAAAAATTTTCCCATTCACATCGAATATTATCACAAGTTCAAACCACTTCCCAAAA
664157_30677280_HHV-7_JI_U43400.1_144861bp_1_521	ACACCAAAAATTTTCCCATTCACATCGAATATTATCACAAGTTCAAACCACTTCCCAAAAACGTTGTGTTTTACACAAAAAAGAACATCTACAATGTTGCAACGAATGACCCTTTTCCAA
664157_30677280_HHV-7_JI_U43400.1_144861bp_1_522	ACGTTGTGTTTTACACAAAAAAGAACATCTACAATGTTGCAACGAATGACCCTTTTCCAACGGTGAAGGTCTTGATCAGTATAAGGTGTCATGGGAAGTCTGCAAAATCGTGTGTATGTT
664157_30677280_HHV-7_JI_U43400.1_144861bp_1_523	CGGTGAAGGTCTTGATCAGTATAAGGTGTCATGGGAAGTCTGCAAAATCGTGTGTATGTTTTATAAGGACTACTTTTCAATATAAAACAAACGTTTATATTTTCATCCCTATAAATGATC
664157_30677280_HHV-7_JI_U43400.1_144861bp_1_524	TTATAAGGACTACTTTTCAATATAAAACAAACGTTTATATTTTCATCCCTATAAATGATCATATCAGAGAAAGATTTGCTATGCTCCACGATTTTGCTACCAGTTTCAAAGTCCACGATA
664157_30677280_HHV-7_JI_U43400.1_144861bp_1_525	ATATCAGAGAAAGATTTGCTATGCTCCACGATTTTGCTACCAGTTTCAAAGTCCACGATAAAATCACACAATTCGTAGATGGGGTAGAAAAACTTCAATCCTACTAAAAAGAAATCTTGT
664157_30677280_HHV-7_JI_U43400.1_144861bp_1_526	AAATCACACAATTCGTAGATGGGGTAGAAAAACTTCAATCCTACTAAAAAGAAATCTTGTAAGTTTGATGAAACATAGTAAAGACTTTTGTCAAAAAAACCATTGTAGACATACAAATGC
664157_30677280_HHV-7_JI_U43400.1_144861bp_1_527	AAGTTTGATGAAACATAGTAAAGACTTTTGTCAAAAAAACCATTGTAGACATACAAATGCCCTCTGTCACCTTGTAAAAGAATTGGTCCTCTGCAAGTTGATTTTTTAACATGATGTAAC
664157_30677280_HHV-7_JI_U43400.1_144861bp_1_528	CCTCTGTCACCTTGTAAAAGAATTGGTCCTCTGCAAGTTGATTTTTTAACATGATGTAACACACCAACAACGATCAACGTTTTTTCACAACACAAGTAAGTGTTTCGAATTTCTTCGATG
664157_30677280_HHV-7_JI_U43400.1_144861bp_1_529	ACACCAACAACGATCAACGTTTTTTCACAACACAAGTAAGTGTTTCGAATTTCTTCGATGTCTTCCTTTGAAATTTCTGGGATTGTTTCTAATGAGTGAAATGTAACTCGCCATTCTTCT

664157_30677280_HHV-7_JI_U43400.1_144861bp_1_530	TCTTCCTTTGAAATTTCTGGGATTGTTTCTAATGAGTGAAATGTAACTCGCCATTCTTCTGGGAAAGGCAATACGAAAGATGTACCTTCCTTACTTAAGATGAATTCTTCGAGAAGAGTA
664157_30677280_HHV-7_JI_U43400.1_144861bp_1_531	GGGAAAGGCAATACGAAAGATGTACCTTCCTTACTTAAGATGAATTCTTCGAGAAGAGTAAGTTTATTGACACAGCACAAATTCGCTAAGAAATCCATGCTTCAGTTTTAAAATTACCCA
664157_30677280_HHV-7_JI_U43400.1_144861bp_1_532	AGTTTATTGACACAGCACAAATTCGCTAAGAAATCCATGCTTCAGTTTTAAAATTACCCAAGAAAAAACAAAGGCATAAATATTTCAATTTTTTTTATTTAGCACATTTAGAAGCTGCAA
664157_30677280_HHV-7_JI_U43400.1_144861bp_1_533	AGAAAAAACAAAGGCATAAATATTTCAATTTTTTTTATTTAGCACATTTAGAAGCTGCAAATTTTCTTTAGAAAAAAATTACAAAGCAAGCTGAACAATAAAAAAGGTTCAACAAATCGC
664157_30677280_HHV-7_JI_U43400.1_144861bp_1_534	ATTTTCTTTAGAAAAAAATTACAAAGCAAGCTGAACAATAAAAAAGGTTCAACAAATCGCCTGTAAAAATATCTTTTCTACCCACTGTTTTTGAAATTTGAGCAAATAGAGCGCTATAAC
664157_30677280_HHV-7_JI_U43400.1_144861bp_1_535	CTGTAAAAATATCTTTTCTACCCACTGTTTTTGAAATTTGAGCAAATAGAGCGCTATAACCTAAGCCAAGAAAGAACTCAAACAACAATGAAAAGAAACAATGTTCTGTTATTACCCCTG
664157_30677280_HHV-7_JI_U43400.1_144861bp_1_536	CTAAGCCAAGAAAGAACTCAAACAACAATGAAAAGAAACAATGTTCTGTTATTACCCCTGCTAACAAACACAGAATACTACACAGAACAAGTTGATATAGTTTAGTGATAAACAAATTAC
664157_30677280_HHV-7_JI_U43400.1_144861bp_1_537	CTAACAAACACAGAATACTACACAGAACAAGTTGATATAGTTTAGTGATAAACAAATTACTATGTAAGACTGCCAAGAAAAATAAATGCTGGCAACTTTTTCTTGGTAAATACAATTTTC
664157_30677280_HHV-7_JI_U43400.1_144861bp_1_538	TATGTAAGACTGCCAAGAAAAATAAATGCTGGCAACTTTTTCTTGGTAAATACAATTTTCCAAACCAAACCAGCAGCGTTAAGGTTAAAAATTGCAGCAAATAAACAATAACATCTTTTA
664157_30677280_HHV-7_JI_U43400.1_144861bp_1_539	CAAACCAAACCAGCAGCGTTAAGGTTAAAAATTGCAGCAAATAAACAATAACATCTTTTATAGCCATTTGGTACGGGCCATTCGGTTTATAGATCGGTTTGTATCGCTGAAGACACATGG
664157_30677280_HHV-7_JI_U43400.1_144861bp_1_540	TAGCCATTTGGTACGGGCCATTCGGTTTATAGATCGGTTTGTATCGCTGAAGACACATGGCAAACTGATGAGAGTTAACCAGAAACTTATAACTGGTTACATATGGCGTTATCAGAAACA
664157_30677280_HHV-7_JI_U43400.1_144861bp_1_541	CAAACTGATGAGAGTTAACCAGAAACTTATAACTGGTTACATATGGCGTTATCAGAAACACTGTAACAAGCAATCTGTAATGAACCAAATCTGATATTTTTAACACAATGGCAATTAGGG

664157_30677280_HHV-7_JI_U43400.1_144861bp_1_542	CTGTAACAAGCAATCTGTAATGAACCAAATCTGATATTTTTAACACAATGGCAATTAGGGTGCCGAAATAAGATGCCATTGAATGCGTTGGAAATATTAGCATGCATCTACAGACTATGG
664157_30677280_HHV-7_JI_U43400.1_144861bp_1_543	TGCCGAAATAAGATGCCATTGAATGCGTTGGAAATATTAGCATGCATCTACAGACTATGGCTCTCAAGCGGATTTCAGAAAAAAATAAAAACGAGCAGATTACCAAAAAAAATTCATCGG
664157_30677280_HHV-7_JI_U43400.1_144861bp_1_544	CTCTCAAGCGGATTTCAGAAAAAAATAAAAACGAGCAGATTACCAAAAAAAATTCATCGGTGATTTGCAACACGAGTAATGATAAAGTTATAATTTTGAGATCGTTGAAAGGACAATAGT
664157_30677280_HHV-7_JI_U43400.1_144861bp_1_545	TGATTTGCAACACGAGTAATGATAAAGTTATAATTTTGAGATCGTTGAAAGGACAATAGTTAAACAAATTCACCCGACGGTAAGTCAATAGCAAACCTCCGCTAAAAAGGAATATTAGCC
664157_30677280_HHV-7_JI_U43400.1_144861bp_1_546	TAAACAAATTCACCCGACGGTAAGTCAATAGCAAACCTCCGCTAAAAAGGAATATTAGCCTACTTGTTCAAACAGCGTTTTCTTTATCATAAGATGGAAACAGCTTTCTCCCGGAATTAC
664157_30677280_HHV-7_JI_U43400.1_144861bp_1_547	TACTTGTTCAAACAGCGTTTTCTTTATCATAAGATGGAAACAGCTTTCTCCCGGAATTACATTCCCTAAAACAAGGCCCATAGAAAAACTTATGATTACATTCCACATCTTTGTCTTTTC
664157_30677280_HHV-7_JI_U43400.1_144861bp_1_548	ATTCCCTAAAACAAGGCCCATAGAAAAACTTATGATTACATTCCACATCTTTGTCTTTTCGAATTTGGGGTATAGTCAAAATGTGCAGAATCGTCACTTTCGCTGTCCTCTTTCTCATTA
664157_30677280_HHV-7_JI_U43400.1_144861bp_1_549	GAATTTGGGGTATAGTCAAAATGTGCAGAATCGTCACTTTCGCTGTCCTCTTTCTCATTAAAAAAATTTGTTCCTGTGCCACCATTTTTAGTGGGCACCATGTAACTTGTAATTTTGTGT
664157_30677280_HHV-7_JI_U43400.1_144861bp_1_550	AAAAAATTTGTTCCTGTGCCACCATTTTTAGTGGGCACCATGTAACTTGTAATTTTGTGTTGCGTTTCCTGTTTACAAAAAAAATTATTTCCTTCCTCCACCTTGTGATTTTTCTCTCCT
664157_30677280_HHV-7_JI_U43400.1_144861bp_1_551	TGCGTTTCCTGTTTACAAAAAAAATTATTTCCTTCCTCCACCTTGTGATTTTTCTCTCCTTTTTCCTCTGTTTTTGCTTGACGTTCAAATTTTGGGTCGTCTTTAACCTGCTCTTGACTT
664157_30677280_HHV-7_JI_U43400.1_144861bp_1_552	TTTTCCTCTGTTTTTGCTTGACGTTCAAATTTTGGGTCGTCTTTAACCTGCTCTTGACTTAAATAAGCTTCTACGGTCAGAAAAACATTTTTAGCGATTAAAAATAATTTGTATTCTTTG
664157_30677280_HHV-7_JI_U43400.1_144861bp_1_553	AAATAAGCTTCTACGGTCAGAAAAACATTTTTAGCGATTAAAAATAATTTGTATTCTTTGCCAGCTATTGCTCTAAGACTACACGAAGACTTTAAAACTGCACAAGTACTAAATGCTTGC

664157_30677280_HHV-7_JI_U43400.1_144861bp_1_554	CCAGCTATTGCTCTAAGACTACACGAAGACTTTAAAACTGCACAAGTACTAAATGCTTGCTGTAGATTTTTGGAAGATAGTACAAGTCTCATGCATTTTGCGTCGTGAAACACAACTTTC
664157_30677280_HHV-7_JI_U43400.1_144861bp_1_555	TGTAGATTTTTGGAAGATAGTACAAGTCTCATGCATTTTGCGTCGTGAAACACAACTTTCCCGCTATGAGCAAATTCTATTTCGTTTAAGTCGGTTAGAAATTTTATACTTGGCGGGTTA
664157_30677280_HHV-7_JI_U43400.1_144861bp_1_556	CCGCTATGAGCAAATTCTATTTCGTTTAAGTCGGTTAGAAATTTTATACTTGGCGGGTTAACTTGCACAACTATTTGTGCCAACGTCATTTCATTTTTATTACTGCGTTTGTTCTTTGTG
664157_30677280_HHV-7_JI_U43400.1_144861bp_1_557	ACTTGCACAACTATTTGTGCCAACGTCATTTCATTTTTATTACTGCGTTTGTTCTTTGTGACCGGAGCAAGCCATTTTAAAATCTCCGTTACAGTTGAATGGTCCAAGTCTATTCGAAGT
664157_30677280_HHV-7_JI_U43400.1_144861bp_1_558	ACCGGAGCAAGCCATTTTAAAATCTCCGTTACAGTTGAATGGTCCAAGTCTATTCGAAGTGCAGATTTTCCGCTTTCTCTTACAATTTCTTGACCATTGACACAAGGAATAGATGCTTGT
664157_30677280_HHV-7_JI_U43400.1_144861bp_1_559	GCAGATTTTCCGCTTTCTCTTACAATTTCTTGACCATTGACACAAGGAATAGATGCTTGTGCACAAATATCTGATGCCGTGACTAAAAACCGCGTATAGAGATCGCTATCATGTTGAATA
664157_30677280_HHV-7_JI_U43400.1_144861bp_1_560	GCACAAATATCTGATGCCGTGACTAAAAACCGCGTATAGAGATCGCTATCATGTTGAATATACAGTTTAGTGACATCAGGATTTGATATAATGCCCATAAAACTATCAAATAATGGAACA
664157_30677280_HHV-7_JI_U43400.1_144861bp_1_561	TACAGTTTAGTGACATCAGGATTTGATATAATGCCCATAAAACTATCAAATAATGGAACAAAGTTATTTATCGTTTTAGTCGAAAAATGATCTGTATCGGTTATATATAAACATTCTGCA
664157_30677280_HHV-7_JI_U43400.1_144861bp_1_562	AAGTTATTTATCGTTTTAGTCGAAAAATGATCTGTATCGGTTATATATAAACATTCTGCATGAATAATCAGCTTTAAAACCAAATGGTTTTTTGCAGATTGAATTATGATTGCCGGTTGA
664157_30677280_HHV-7_JI_U43400.1_144861bp_1_563	TGAATAATCAGCTTTAAAACCAAATGGTTTTTTGCAGATTGAATTATGATTGCCGGTTGAGGAGTAAAAGTAACTGTAGTATTTTCTCGCAAAAGCTTTGCAAAAGCTCTTAGAGGCTTA
664157_30677280_HHV-7_JI_U43400.1_144861bp_1_564	GGAGTAAAAGTAACTGTAGTATTTTCTCGCAAAAGCTTTGCAAAAGCTCTTAGAGGCTTATTGATTGTTTTCCAAGTTTTCATGTGAAAAGCCATGGTGGGAGGCTCTTTGGAATCTCGA
664157_30677280_HHV-7_JI_U43400.1_144861bp_1_565	TTGATTGTTTTCCAAGTTTTCATGTGAAAAGCCATGGTGGGAGGCTCTTTGGAATCTCGATGTTCGCGATGATCTCGGTGACTATGGTGTTCTCTGTTGCTACGATCCATGCTGCACCAA

664157_30677280_HHV-7_JI_U43400.1_144861bp_1_566	TGTTCGCGATGATCTCGGTGACTATGGTGTTCTCTGTTGCTACGATCCATGCTGCACCAAAAATAGATATTAACAGGAAGAGTTGTATGAGAATACTAAAAAATCACAGAGATATTCAAA
664157_30677280_HHV-7_JI_U43400.1_144861bp_1_567	AAATAGATATTAACAGGAAGAGTTGTATGAGAATACTAAAAAATCACAGAGATATTCAAACCATTATATACTGTGTTTTTTTGGCGCCAAATTTTTATTACATCACTGAAATACATGCAT
664157_30677280_HHV-7_JI_U43400.1_144861bp_1_568	CCATTATATACTGTGTTTTTTTGGCGCCAAATTTTTATTACATCACTGAAATACATGCATAGTACTCATCACCACACCATCAGAAAAACCACTCAGCAAATCAAATTTTTTTGATGGATT
664157_30677280_HHV-7_JI_U43400.1_144861bp_1_569	AGTACTCATCACCACACCATCAGAAAAACCACTCAGCAAATCAAATTTTTTTGATGGATTGACACCGTCACACTTTTGATTTTTATACACAGCTTGATAAATTCCTGTTTTAAACCCATT
664157_30677280_HHV-7_JI_U43400.1_144861bp_1_570	GACACCGTCACACTTTTGATTTTTATACACAGCTTGATAAATTCCTGTTTTAAACCCATTTTGATACAAACATACTAAAATGTCCCTCATATTTTCTGTCTTATTTATGAAAAATACATT
664157_30677280_HHV-7_JI_U43400.1_144861bp_1_571	TTGATACAAACATACTAAAATGTCCCTCATATTTTCTGTCTTATTTATGAAAAATACATTTGCTTGTCCATGATCTGTAAACGGCAACGCACCGACGTACATATTTAGCATTTCATTCAC
664157_30677280_HHV-7_JI_U43400.1_144861bp_1_572	TGCTTGTCCATGATCTGTAAACGGCAACGCACCGACGTACATATTTAGCATTTCATTCACAGAATAGTCAAAAGCATTGAGAAAAATTGTCATTTCCTGCATTTCTTCTTCGGAAAATAA
664157_30677280_HHV-7_JI_U43400.1_144861bp_1_573	AGAATAGTCAAAAGCATTGAGAAAAATTGTCATTTCCTGCATTTCTTCTTCGGAAAATAAATTTTTGTTGACTTTGGACACATCATAATTTACAGAGCTTAAGTACTCTAAATGGTTTTT
664157_30677280_HHV-7_JI_U43400.1_144861bp_1_574	ATTTTTGTTGACTTTGGACACATCATAATTTACAGAGCTTAAGTACTCTAAATGGTTTTTAAATCTGTTTAAGAACAGGCCATTGTACACTGGTATGTATAATGTCTGTAATTCAGTAGA
664157_30677280_HHV-7_JI_U43400.1_144861bp_1_575	AAATCTGTTTAAGAACAGGCCATTGTACACTGGTATGTATAATGTCTGTAATTCAGTAGAATAGACAGACAGATCACTATGGAAAACAGGATTAGGATGCAAAACTTTTATACTAGATCG
664157_30677280_HHV-7_JI_U43400.1_144861bp_1_576	ATAGACAGACAGATCACTATGGAAAACAGGATTAGGATGCAAAACTTTTATACTAGATCGCTTTAAAATCTTATTTCCCTCTATGGGACAAAAAGATTGTGAGCAATTTAACAAATCGAA
664157_30677280_HHV-7_JI_U43400.1_144861bp_1_577	CTTTAAAATCTTATTTCCCTCTATGGGACAAAAAGATTGTGAGCAATTTAACAAATCGAACTCTTTTGACATGGCCGAACCAGACACAAAGGTTAGATTTCTAAGCCCATATTTTTTTAT

664157_30677280_HHV-7_JI_U43400.1_144861bp_1_578	CTCTTTTGACATGGCCGAACCAGACACAAAGGTTAGATTTCTAAGCCCATATTTTTTTATGTCGCTTCTTAAATTCGTCCATAAAACATTTGGTAATGTATATTCAACATTATCATACAA
664157_30677280_HHV-7_JI_U43400.1_144861bp_1_579	GTCGCTTCTTAAATTCGTCCATAAAACATTTGGTAATGTATATTCAACATTATCATACAAATCGCAGTGCAAAATCCCTTGTGCATATTTGGATCTTTGAAAAAAACTGCAAGGTTCCGC
664157_30677280_HHV-7_JI_U43400.1_144861bp_1_580	ATCGCAGTGCAAAATCCCTTGTGCATATTTGGATCTTTGAAAAAAACTGCAAGGTTCCGCTCCTTTTTTGCAACAATTCATACTTGTTCTAACACTAGAATAGTAAATGTTCTCGCATAC
664157_30677280_HHV-7_JI_U43400.1_144861bp_1_581	TCCTTTTTTGCAACAATTCATACTTGTTCTAACACTAGAATAGTAAATGTTCTCGCATACTATTCGATATAATTGGCATCCAAGTTGAGAATTAAATGGCAGATTCATTGACATTAATGC
664157_30677280_HHV-7_JI_U43400.1_144861bp_1_582	TATTCGATATAATTGGCATCCAAGTTGAGAATTAAATGGCAGATTCATTGACATTAATGCTGAATGTAATCCAGTAACACAGATAGCCAAGCTTCTTGCATTGACTATTCCATCCATTAA
664157_30677280_HHV-7_JI_U43400.1_144861bp_1_583	TGAATGTAATCCAGTAACACAGATAGCCAAGCTTCTTGCATTGACTATTCCATCCATTAAAAAATCCCTATTTTCCAAAGCATAATCTATAACAGCATTCCCAATCAAAACTAACTCAGT
664157_30677280_HHV-7_JI_U43400.1_144861bp_1_584	AAAATCCCTATTTTCCAAAGCATAATCTATAACAGCATTCCCAATCAAAACTAACTCAGTCACTGTTTTTCTCATTTTCTTTAAATTGAAAATTTTATCTCTATAATGAAACATATCTAT
664157_30677280_HHV-7_JI_U43400.1_144861bp_1_585	CACTGTTTTTCTCATTTTCTTTAAATTGAAAATTTTATCTCTATAATGAAACATATCTATGTTAGTATGGGACTTTTGTTCTTCTGAAACGTTTTCGACAAAATAAGAAAGATTTAAGGC
664157_30677280_HHV-7_JI_U43400.1_144861bp_1_586	GTTAGTATGGGACTTTTGTTCTTCTGAAACGTTTTCGACAAAATAAGAAAGATTTAAGGCAATTCTGAAAGCTGTATTCAGGCCATATTCAAAAGGAATTACATCCATAGAATTTCCAAG
664157_30677280_HHV-7_JI_U43400.1_144861bp_1_587	AATTCTGAAAGCTGTATTCAGGCCATATTCAAAAGGAATTACATCCATAGAATTTCCAAGACAGTAGGAAGCTATTTGAGGTAACATGGAGTACTTATGTATATTTTTCCTAAAGATGAA
664157_30677280_HHV-7_JI_U43400.1_144861bp_1_588	ACAGTAGGAAGCTATTTGAGGTAACATGGAGTACTTATGTATATTTTTCCTAAAGATGAATCCCATCTGTCCTCTGCGTAGGCAGCGACAAATATTATCCAGCAACGTTTCAGTTTTGAT
664157_30677280_HHV-7_JI_U43400.1_144861bp_1_589	TCCCATCTGTCCTCTGCGTAGGCAGCGACAAATATTATCCAGCAACGTTTCAGTTTTGATTCTGGCATGTTCAGCGGTGCGTTCACATTCAAGATATTTACTTGTAAAATCAGATTCATC

664157_30677280_HHV-7_JI_U43400.1_144861bp_1_590	TCTGGCATGTTCAGCGGTGCGTTCACATTCAAGATATTTACTTGTAAAATCAGATTCATCATATAACCCTAAATTAATTGCCAGAGCTTTGTGGAAAACTGACCAATAAGAATTTTCAGT
664157_30677280_HHV-7_JI_U43400.1_144861bp_1_591	ATATAACCCTAAATTAATTGCCAGAGCTTTGTGGAAAACTGACCAATAAGAATTTTCAGTTTTCAGCCGTTCCATAAAAATTTCTGGCAGTATTAGACAAAACTTGACTTGAACAGTTGT
664157_30677280_HHV-7_JI_U43400.1_144861bp_1_592	TTTCAGCCGTTCCATAAAAATTTCTGGCAGTATTAGACAAAACTTGACTTGAACAGTTGTTGGCAAAATTTCACAGAAGTCCAGCCACATTAATAATGACAGGTGCCAAATTTCAAGATA
664157_30677280_HHV-7_JI_U43400.1_144861bp_1_593	TGGCAAAATTTCACAGAAGTCCAGCCACATTAATAATGACAGGTGCCAAATTTCAAGATAAATTGTTAGACCAGTTGTTTTATTTGTGTCCTTCTCTACAATTTGTAACTGAGACAATAG
664157_30677280_HHV-7_JI_U43400.1_144861bp_1_594	AATTGTTAGACCAGTTGTTTTATTTGTGTCCTTCTCTACAATTTGTAACTGAGACAATAGCAATTCCATAAGAGACTCTGCCTCGTAGTGATAACGAGTCAGATTTACACTAACATATGA
664157_30677280_HHV-7_JI_U43400.1_144861bp_1_595	CAATTCCATAAGAGACTCTGCCTCGTAGTGATAACGAGTCAGATTTACACTAACATATGATCCATTTAACATCGCAGGCAAGAAAAAACCACTGTTAATGTCCAAAAAATCTTCCAGAGA
664157_30677280_HHV-7_JI_U43400.1_144861bp_1_596	TCCATTTAACATCGCAGGCAAGAAAAAACCACTGTTAATGTCCAAAAAATCTTCCAGAGAAAAGTTACTCACATTATGAAAGACAGTGTCAAAAACACTCTTTTTTTCAGACACTAAACC
664157_30677280_HHV-7_JI_U43400.1_144861bp_1_597	AAAGTTACTCACATTATGAAAGACAGTGTCAAAAACACTCTTTTTTTCAGACACTAAACCCAAATTACTCATACAAGGAAAAGGTAACATACAAACTCTCTTATACAATAAAATCAAAAT
664157_30677280_HHV-7_JI_U43400.1_144861bp_1_598	CAAATTACTCATACAAGGAAAAGGTAACATACAAACTCTCTTATACAATAAAATCAAAATTTCTTCCACTAGAGGGCATTTGTGACTAACAAAACTTTTTTGTAAATATTCGATTTTTTT
664157_30677280_HHV-7_JI_U43400.1_144861bp_1_599	TTCTTCCACTAGAGGGCATTTGTGACTAACAAAACTTTTTTGTAAATATTCGATTTTTTTTATTTGACTGGCCAATGTCACACTGATTCTTAGAAAAATCGTTGGGATGTTTTCTTCGAT
664157_30677280_HHV-7_JI_U43400.1_144861bp_1_600	TATTTGACTGGCCAATGTCACACTGATTCTTAGAAAAATCGTTGGGATGTTTTCTTCGATTCCGTTAAATGAATTTGAAATCTCATCGAGAAATCTCGATGCAGCTAATACTCCTGGTGA
664157_30677280_HHV-7_JI_U43400.1_144861bp_1_601	TCCGTTAAATGAATTTGAAATCTCATCGAGAAATCTCGATGCAGCTAATACTCCTGGTGAAAACTTCGCTCTCCAGACATGGATAGCTGCTTGCAAGATTTTTTCAAGTTCTACTTTGGA

664157_30677280_HHV-7_JI_U43400.1_144861bp_1_602	AAACTTCGCTCTCCAGACATGGATAGCTGCTTGCAAGATTTTTTCAAGTTCTACTTTGGAGTCACGTACAAATTCAATCAATTCTTTTTGTAAAATATTTTCACTTTCTAAAATAAGTGA
664157_30677280_HHV-7_JI_U43400.1_144861bp_1_603	GTCACGTACAAATTCAATCAATTCTTTTTGTAAAATATTTTCACTTTCTAAAATAAGTGAGATGGAATCAAATTCCGAAGCAGAGTTTGTTTTGAGATAGAACTGGCCTAACAAACGATC
664157_30677280_HHV-7_JI_U43400.1_144861bp_1_604	GATGGAATCAAATTCCGAAGCAGAGTTTGTTTTGAGATAGAACTGGCCTAACAAACGATCAACGTGCGGTGCATCATGCATTGCTCCCAGTGCTGAAATTATGGCATCACGTAAGCACCA
664157_30677280_HHV-7_JI_U43400.1_144861bp_1_605	AACGTGCGGTGCATCATGCATTGCTCCCAGTGCTGAAATTATGGCATCACGTAAGCACCACATATCTAAGCCCGAATTTAAGCGCCGGAGAATTTTTGAAATTTCATATAACTGCTTATA
664157_30677280_HHV-7_JI_U43400.1_144861bp_1_606	CATATCTAAGCCCGAATTTAAGCGCCGGAGAATTTTTGAAATTTCATATAACTGCTTATAAAGAAGAGTTTTGTGATGGAAGCGTGAATAGCAAAACCGTTTTTTGTTATATTCATCATG
664157_30677280_HHV-7_JI_U43400.1_144861bp_1_607	AAGAAGAGTTTTGTGATGGAAGCGTGAATAGCAAAACCGTTTTTTGTTATATTCATCATGACAAAGACATTTCCTCAAGAGACCCATGTTTTTATTTATGGAAACTTTATTGTTAGGATC
664157_30677280_HHV-7_JI_U43400.1_144861bp_1_608	ACAAAGACATTTCCTCAAGAGACCCATGTTTTTATTTATGGAAACTTTATTGTTAGGATCCATAATCACAAGAAAATTTTTCTTTCACACAATTCGACAAAGACGTCTTTGAGTCTTATA
664157_30677280_HHV-7_JI_U43400.1_144861bp_1_609	CATAATCACAAGAAAATTTTTCTTTCACACAATTCGACAAAGACGTCTTTGAGTCTTATAATGGTTAATGAGATACCCTGTAAGTCTAAAATTCCTGATTTTATGTTTTGGGTTTTACAA
664157_30677280_HHV-7_JI_U43400.1_144861bp_1_610	ATGGTTAATGAGATACCCTGTAAGTCTAAAATTCCTGATTTTATGTTTTGGGTTTTACAATGACCAATTCGTTGAAGGCTTCCAAAATAGTTTAGATTTCCATCACCGTTGATCAAATAG
664157_30677280_HHV-7_JI_U43400.1_144861bp_1_611	TGACCAATTCGTTGAAGGCTTCCAAAATAGTTTAGATTTCCATCACCGTTGATCAAATAGTTCATAAACATAAATCGTTCTTTGGAAAACTTGTTTCTTAATAAATCTATCAATACTTCT
664157_30677280_HHV-7_JI_U43400.1_144861bp_1_612	TTCATAAACATAAATCGTTCTTTGGAAAACTTGTTTCTTAATAAATCTATCAATACTTCTTCATGTGTCAGCTCAGATAAAACAATGTAGATCCCGTATTCTATTTTATTAAATGCTTGC
664157_30677280_HHV-7_JI_U43400.1_144861bp_1_613	TCATGTGTCAGCTCAGATAAAACAATGTAGATCCCGTATTCTATTTTATTAAATGCTTGCCTATAGATATAACAGAGGTAAATGTACTGAATTGCATTTTGTCTTGGTTCCCAGTCAGGT

664157_30677280_HHV-7_JI_U43400.1_144861bp_1_614	CTATAGATATAACAGAGGTAAATGTACTGAATTGCATTTTGTCTTGGTTCCCAGTCAGGTTCCAATAATAAATCTTTCAATATGACCAATCCATATATATTTTCCTTTCTTACGTCATAT
664157_30677280_HHV-7_JI_U43400.1_144861bp_1_615	TCCAATAATAAATCTTTCAATATGACCAATCCATATATATTTTCCTTTCTTACGTCATATACATGACCTTGCATTACACAGGATATTAGACAATTAGTCCCTTGAGAGATATAATTAGTT
664157_30677280_HHV-7_JI_U43400.1_144861bp_1_616	ACATGACCTTGCATTACACAGGATATTAGACAATTAGTCCCTTGAGAGATATAATTAGTTACTCCAAACAAAGACACCTTTTCTTCTAAGTAAGAACAACTATCTAACATTGTGTTGATA
664157_30677280_HHV-7_JI_U43400.1_144861bp_1_617	ACTCCAAACAAAGACACCTTTTCTTCTAAGTAAGAACAACTATCTAACATTGTGTTGATACTGTCAACTAATGAAGTTTTATACAGATTAGCTAAAGAAGTCCTCGTGCAGAAAGACACA
664157_30677280_HHV-7_JI_U43400.1_144861bp_1_618	CTGTCAACTAATGAAGTTTTATACAGATTAGCTAAAGAAGTCCTCGTGCAGAAAGACACATTGAAGGCATTCATGGCTAAAAACAATACACTAATCAACAAAAATTTTGGACTCTCCGCG
664157_30677280_HHV-7_JI_U43400.1_144861bp_1_619	TTGAAGGCATTCATGGCTAAAAACAATACACTAATCAACAAAAATTTTGGACTCTCCGCGTCTGTTTCAAGTCTGAACATAAGCATTCCTAAATGGTCTCGTTCAGGTCCTAAAAGTTCG
664157_30677280_HHV-7_JI_U43400.1_144861bp_1_620	TCTGTTTCAAGTCTGAACATAAGCATTCCTAAATGGTCTCGTTCAGGTCCTAAAAGTTCGCAAGGAGTAAAACTTCGCGAAAGCACACACTCTTTGGGAACACCAAAAGCTAGCAAACTT
664157_30677280_HHV-7_JI_U43400.1_144861bp_1_621	CAAGGAGTAAAACTTCGCGAAAGCACACACTCTTTGGGAACACCAAAAGCTAGCAAACTTTTGATCTTTTTTTTCTCATTTTCAATGAGATTATTTAAGGAAGTTTCATCAATTGTCTGA
664157_30677280_HHV-7_JI_U43400.1_144861bp_1_622	TTGATCTTTTTTTTCTCATTTTCAATGAGATTATTTAAGGAAGTTTCATCAATTGTCTGATGTTTCCTTTTTTGAAGCATTTCCATTGTTCGCAAGCGAAATGGATTGTACAAATTTACA
664157_30677280_HHV-7_JI_U43400.1_144861bp_1_623	TGTTTCCTTTTTTGAAGCATTTCCATTGTTCGCAAGCGAAATGGATTGTACAAATTTACAATTTGAGTCAATATTGAATTTACTTAAAACAGATGTGTCCGATAATACATTCTTAACTAT
664157_30677280_HHV-7_JI_U43400.1_144861bp_1_624	ATTTGAGTCAATATTGAATTTACTTAAAACAGATGTGTCCGATAATACATTCTTAACTATAACAGCGAAAATTGAAATTTTTTCTCTTGCGGCTGAAAGTATCACAGCAGATAAGATATT
664157_30677280_HHV-7_JI_U43400.1_144861bp_1_625	AACAGCGAAAATTGAAATTTTTTCTCTTGCGGCTGAAAGTATCACAGCAGATAAGATATTGTCATTTGCGAAATTATTACCAATACACAATTATCATTTCAACTTTATTAAAAATCATAT

664157_30677280_HHV-7_JI_U43400.1_144861bp_1_626	GTCATTTGCGAAATTATTACCAATACACAATTATCATTTCAACTTTATTAAAAATCATATGGTGTTTTATATATTAAATTATAGTACTCTAGGATTTGCCAAAAGATTTTCAATAGCTGA
664157_30677280_HHV-7_JI_U43400.1_144861bp_1_627	GGTGTTTTATATATTAAATTATAGTACTCTAGGATTTGCCAAAAGATTTTCAATAGCTGAAACACTATGTAGAGAATTAAAAATTTTCCACAGAACATTCCAATCAACGTCTAAATTACA
664157_30677280_HHV-7_JI_U43400.1_144861bp_1_628	AACACTATGTAGAGAATTAAAAATTTTCCACAGAACATTCCAATCAACGTCTAAATTACAAAACTTAAATAATGCAGAAGTCCTTTATCAATTTGAAAAACTTATTGAATCCATTCAAGT
664157_30677280_HHV-7_JI_U43400.1_144861bp_1_629	AAACTTAAATAATGCAGAAGTCCTTTATCAATTTGAAAAACTTATTGAATCCATTCAAGTATTCAAGGCGGAATTGGCATCGCCTATTGGTAAACTTCTACGTCAAGAAACAATGATCTA
664157_30677280_HHV-7_JI_U43400.1_144861bp_1_630	ATTCAAGGCGGAATTGGCATCGCCTATTGGTAAACTTCTACGTCAAGAAACAATGATCTATGACAAACCCACAGAAAAATCAGTTAAATATGTCATTATTCAGCAAAATTTGATAAAAAT
664157_30677280_HHV-7_JI_U43400.1_144861bp_1_631	TGACAAACCCACAGAAAAATCAGTTAAATATGTCATTATTCAGCAAAATTTGATAAAAATTAATAACTTAATTCAAGACTGTGAATCGATAAAAAACTGTAGACTCATAGCGGAGCTGAT
664157_30677280_HHV-7_JI_U43400.1_144861bp_1_632	TAATAACTTAATTCAAGACTGTGAATCGATAAAAAACTGTAGACTCATAGCGGAGCTGATAGATGAATTATATCAAAAAGTTTATAGGTGGTTTATGCAAATTTTCACATACGACGATAT
664157_30677280_HHV-7_JI_U43400.1_144861bp_1_633	AGATGAATTATATCAAAAAGTTTATAGGTGGTTTATGCAAATTTTCACATACGACGATATTATTTTCCCCAATGACAATTTCTTAGATAGACTTTTAAAAATGGATTTCTGCTATACATA
664157_30677280_HHV-7_JI_U43400.1_144861bp_1_634	TATTTTCCCCAATGACAATTTCTTAGATAGACTTTTAAAAATGGATTTCTGCTATACATATTACACGGCATCTAATCAGCATTTATTATCACTTTTTGAACAGACAATTGACAATCAAAT
664157_30677280_HHV-7_JI_U43400.1_144861bp_1_635	TTACACGGCATCTAATCAGCATTTATTATCACTTTTTGAACAGACAATTGACAATCAAATTTTTATCGACCCTTCTCCATACTTCGAAATAAATCCAGTATATTCTCCCGAATTACAATT
664157_30677280_HHV-7_JI_U43400.1_144861bp_1_636	TTTTATCGACCCTTCTCCATACTTCGAAATAAATCCAGTATATTCTCCCGAATTACAATTTATGAGCACATTTAGCTTAAAGATATTTTCAAAAAATATTTCAGCGAAAAACGAAGATCT
664157_30677280_HHV-7_JI_U43400.1_144861bp_1_637	TATGAGCACATTTAGCTTAAAGATATTTTCAAAAAATATTTCAGCGAAAAACGAAGATCTATATATTTATCCACTTTTAAAAACAAACTTTTCCATACTTACCTTTCTGAGTTTAGAAAA

664157_30677280_HHV-7_JI_U43400.1_144861bp_1_638	ATATATTTATCCACTTTTAAAAACAAACTTTTCCATACTTACCTTTCTGAGTTTAGAAAATATTTTTTTCCATCATGGATTTATATACCACATCTTGCATAGAACAAATATCACACCAAC
664157_30677280_HHV-7_JI_U43400.1_144861bp_1_639	TATTTTTTTCCATCATGGATTTATATACCACATCTTGCATAGAACAAATATCACACCAACTGAAGAAAAAAAAATTAGGAAAAATAAATGGATTCTTAAACACGGTTATTCAGCAAGTCCT
664157_30677280_HHV-7_JI_U43400.1_144861bp_1_640	TGAAGAAAAAAAAATTAGGAAAAATAAATGGATTCTTAAACACGGTTATTCAGCAAGTCCTAATTAAAAAGAATAACTTACAAATCACTTTCCCGGAGTTGCTTGATAAAATCTATCATTT
664157_30677280_HHV-7_JI_U43400.1_144861bp_1_641	AATTAAAAAGAATAACTTACAAATCACTTTCCCGGAGTTGCTTGATAAAATCTATCATTTGCATAGAATAGGCTTGAATATCGAAACGGCACAAATTTTTTTACAAATGTTAACAACATA
664157_30677280_HHV-7_JI_U43400.1_144861bp_1_642	GCATAGAATAGGCTTGAATATCGAAACGGCACAAATTTTTTTACAAATGTTAACAACATACAAACCAGCGACGACAAATAATAAGTTGCAGACATTTTTCACGAATTTTTCAATAATAAT
664157_30677280_HHV-7_JI_U43400.1_144861bp_1_643	CAAACCAGCGACGACAAATAATAAGTTGCAGACATTTTTCACGAATTTTTCAATAATAATTTTTTCGGCATATATTTTTTTTCTATGTATTGAGTTATTCAGCCCAACTTTTATTTTCCA
664157_30677280_HHV-7_JI_U43400.1_144861bp_1_644	TTTTTCGGCATATATTTTTTTTCTATGTATTGAGTTATTCAGCCCAACTTTTATTTTCCATAATAAAAAAAAACTTATCCTCGAGAAACAAAAATCCATCATACTAATTCTCGGTGAACA
664157_30677280_HHV-7_JI_U43400.1_144861bp_1_645	TAATAAAAAAAAACTTATCCTCGAGAAACAAAAATCCATCATACTAATTCTCGGTGAACAGTTCTCTTTCATTTGGAAAGAGGTAAATGAAATTGTAGATTTGTTATTTAGTAGCACAGT
664157_30677280_HHV-7_JI_U43400.1_144861bp_1_646	GTTCTCTTTCATTTGGAAAGAGGTAAATGAAATTGTAGATTTGTTATTTAGTAGCACAGTTACAGAAACCTATTTCAAATTTTATTCTAAAGGTGCCGATGACTACGAAAAAGATTTTCT
664157_30677280_HHV-7_JI_U43400.1_144861bp_1_647	TACAGAAACCTATTTCAAATTTTATTCTAAAGGTGCCGATGACTACGAAAAAGATTTTCTTTATAAAGACTTAATGGAAAAATGGGGAGAATTGTTTTTCCCATTAACTTATTCAATGAC
664157_30677280_HHV-7_JI_U43400.1_144861bp_1_648	TTATAAAGACTTAATGGAAAAATGGGGAGAATTGTTTTTCCCATTAACTTATTCAATGACAACGCCACAGAAATATACCGACAAGCATGTATCAAGTACAGTTTTGAAAAATTTATGTGA
664157_30677280_HHV-7_JI_U43400.1_144861bp_1_649	AACGCCACAGAAATATACCGACAAGCATGTATCAAGTACAGTTTTGAAAAATTTATGTGATACAGCATATCAAAGTAAGATGGAAACAGCGTATGAAAGTCTATTACCATATATTACTCA

664157_30677280_HHV-7_JI_U43400.1_144861bp_1_650	TACAGCATATCAAAGTAAGATGGAAACAGCGTATGAAAGTCTATTACCATATATTACTCACCCAGAGTTCAAATTCATTTTTATTACACATTACGTCAGACCTTCTTTGTCATTAATCAC
664157_30677280_HHV-7_JI_U43400.1_144861bp_1_651	CCCAGAGTTCAAATTCATTTTTATTACACATTACGTCAGACCTTCTTTGTCATTAATCACAAACTTAACCTTTGAAGAAATAAAAGATAATCGAAGATTACTAATACTAATATTCGCGTG
664157_30677280_HHV-7_JI_U43400.1_144861bp_1_652	AAACTTAACCTTTGAAGAAATAAAAGATAATCGAAGATTACTAATACTAATATTCGCGTGTAAACTGCTAATGCCATCAAATTATCTTTTATCACATTACTTATTATTGTTACACGCTTT
664157_30677280_HHV-7_JI_U43400.1_144861bp_1_653	TAAACTGCTAATGCCATCAAATTATCTTTTATCACATTACTTATTATTGTTACACGCTTTCACACTACAAATTTTCAAAGTAGATCTTGGCCATTTTTCAATTATACATGCAATTACTCA
664157_30677280_HHV-7_JI_U43400.1_144861bp_1_654	CACACTACAAATTTTCAAAGTAGATCTTGGCCATTTTTCAATTATACATGCAATTACTCAAAAAATTTTTGACAATATTAATTCTCTGACCCAAACTATTTTTATTCCAAAAACAAATTT
664157_30677280_HHV-7_JI_U43400.1_144861bp_1_655	AAAAATTTTTGACAATATTAATTCTCTGACCCAAACTATTTTTATTCCAAAAACAAATTTTTTAGTCAGCCTTTTATTAACAGCATACACTGTGCATATGCAAACTTATGTGAATCCTTG
664157_30677280_HHV-7_JI_U43400.1_144861bp_1_656	TTTAGTCAGCCTTTTATTAACAGCATACACTGTGCATATGCAAACTTATGTGAATCCTTGGATACAAAAAACAATCAGTGAAAATATAGCCCTTCTTAAAGAATACATCGATTTCACAAA
664157_30677280_HHV-7_JI_U43400.1_144861bp_1_657	GATACAAAAAACAATCAGTGAAAATATAGCCCTTCTTAAAGAATACATCGATTTCACAAAGAAATGTTCAAGTACTTTGGCAACTACGTGCTATTTGAACTTAGAAAATTTTGCAGTAAA
664157_30677280_HHV-7_JI_U43400.1_144861bp_1_658	GAAATGTTCAAGTACTTTGGCAACTACGTGCTATTTGAACTTAGAAAATTTTGCAGTAAATATGTATTTTGGGAAAAACAAGGTTGGAAGTACTTCACTATCAGCTTTTTATAGAACTTG
664157_30677280_HHV-7_JI_U43400.1_144861bp_1_659	TATGTATTTTGGGAAAAACAAGGTTGGAAGTACTTCACTATCAGCTTTTTATAGAACTTGCTCTAAGCTAATTGAGGAATCTAAACTGTTTAAAGATAGGCTTCAAGAAATCAAAGTTTC
664157_30677280_HHV-7_JI_U43400.1_144861bp_1_660	CTCTAAGCTAATTGAGGAATCTAAACTGTTTAAAGATAGGCTTCAAGAAATCAAAGTTTCAAAAACATTGTTTATAGAGATGCTACAAAATGTTGTGAAAAACATCACAAAATTTAAGGA
664157_30677280_HHV-7_JI_U43400.1_144861bp_1_661	AAAAACATTGTTTATAGAGATGCTACAAAATGTTGTGAAAAACATCACAAAATTTAAGGATTTAGTCTCAAATCAAACTTTGCAAAATTTTATAATTATTGTTGAAAGAATCTCCTCTCA

664157_30677280_HHV-7_JI_U43400.1_144861bp_1_662	TTTAGTCTCAAATCAAACTTTGCAAAATTTTATAATTATTGTTGAAAGAATCTCCTCTCATGCAAATACAACATATCAAGATGTTCTCAATAGCATAGATGAATGCCATTTTTCAAACAT
664157_30677280_HHV-7_JI_U43400.1_144861bp_1_663	TGCAAATACAACATATCAAGATGTTCTCAATAGCATAGATGAATGCCATTTTTCAAACATGCAACTTATCCAATCTTTTAAAAACATCGTGTATGTCATTGATGTTCTGAATACTAAAAA
664157_30677280_HHV-7_JI_U43400.1_144861bp_1_664	GCAACTTATCCAATCTTTTAAAAACATCGTGTATGTCATTGATGTTCTGAATACTAAAAACATCTTTAACTTTTCTCTCGCATCACAATTAATTGAAGCGAAAAAACTTGTAAAAAAACA
664157_30677280_HHV-7_JI_U43400.1_144861bp_1_665	CATCTTTAACTTTTCTCTCGCATCACAATTAATTGAAGCGAAAAAACTTGTAAAAAAACAGGACACCTATAATCAATTAAATGTGCAAGATGATTTTGTCACTGTATTAAAGTCACATCT
664157_30677280_HHV-7_JI_U43400.1_144861bp_1_666	GGACACCTATAATCAATTAAATGTGCAAGATGATTTTGTCACTGTATTAAAGTCACATCTAAATAATCTGTTTGAAAAGCAGAAGCCTACAATTAATATTGAAAGAAGATTTATGTTAGA
664157_30677280_HHV-7_JI_U43400.1_144861bp_1_667	AAATAATCTGTTTGAAAAGCAGAAGCCTACAATTAATATTGAAAGAAGATTTATGTTAGAAGGAATACCCGACATAAAACAGATTCCATTCTTGGATGTTTTTGATGAAAGATATAGACT
664157_30677280_HHV-7_JI_U43400.1_144861bp_1_668	AGGAATACCCGACATAAAACAGATTCCATTCTTGGATGTTTTTGATGAAAGATATAGACTAATACCACAAATTGAAAAGTATCTGCATTGGTACATTGCATATAGTGAAGCTGCGCAGGC
664157_30677280_HHV-7_JI_U43400.1_144861bp_1_669	AATACCACAAATTGAAAAGTATCTGCATTGGTACATTGCATATAGTGAAGCTGCGCAGGCTGATTTGGTCGAGCCTTTACTCTTAAAACTTGGTTAGATGAGAATCATAGCAGGAAGCAC
664157_30677280_HHV-7_JI_U43400.1_144861bp_1_670	TGATTTGGTCGAGCCTTTACTCTTAAAACTTGGTTAGATGAGAATCATAGCAGGAAGCACAAATCAAAACGATCCTAAATACGGACCAAGAGCCGGAAAGCAATGTATGTCAAATTGTTT
664157_30677280_HHV-7_JI_U43400.1_144861bp_1_671	AAATCAAAACGATCCTAAATACGGACCAAGAGCCGGAAAGCAATGTATGTCAAATTGTTTTTCTTTCTTGCATACAGTTTATTTGAACGGAATAAACAATGTGTTAAATAAAGAGTCTAT
664157_30677280_HHV-7_JI_U43400.1_144861bp_1_672	TTCTTTCTTGCATACAGTTTATTTGAACGGAATAAACAATGTGTTAAATAAAGAGTCTATTGACATAATCATGGAAAATGGAGCATTATTGGATAATATCAGTACAACGACATTGAAACT
664157_30677280_HHV-7_JI_U43400.1_144861bp_1_673	TGACATAATCATGGAAAATGGAGCATTATTGGATAATATCAGTACAACGACATTGAAACTCGAAACTGGCAATATCCCAGAATATCGATTTTTCACAGAAATCCCAAAAAAAATTAGTTC

664157_30677280_HHV-7_JI_U43400.1_144861bp_1_674	CGAAACTGGCAATATCCCAGAATATCGATTTTTCACAGAAATCCCAAAAAAAATTAGTTCTAATTTTGGCGAAACAATACATGAATTATCTAGACCCTTTAATGGTACCTTAGAATCACA
664157_30677280_HHV-7_JI_U43400.1_144861bp_1_675	TAATTTTGGCGAAACAATACATGAATTATCTAGACCCTTTAATGGTACCTTAGAATCACAACATATAGATAATGAAGTTTATCTTGGACTGTTAGACTTTCTATTGTATGGGAAAAATAA
664157_30677280_HHV-7_JI_U43400.1_144861bp_1_676	ACATATAGATAATGAAGTTTATCTTGGACTGTTAGACTTTCTATTGTATGGGAAAAATAAGAAACCAGCTTTTATTGTCATCACTATAGGGGTAATGGCACGAGCTATATTTATAGTTGA
664157_30677280_HHV-7_JI_U43400.1_144861bp_1_677	GAAACCAGCTTTTATTGTCATCACTATAGGGGTAATGGCACGAGCTATATTTATAGTTGATGAATTGTTTTACCTTTTTGATTCACATGCATCAGACACAGAAAACTCTGCAGCCATCTA
664157_30677280_HHV-7_JI_U43400.1_144861bp_1_678	TGAATTGTTTTACCTTTTTGATTCACATGCATCAGACACAGAAAACTCTGCAGCCATCTATATCTGTGAGGATATTGACGAATTATATGCTCTATTGGCCATAGAGAATGTTGCGGAATT
664157_30677280_HHV-7_JI_U43400.1_144861bp_1_679	TATCTGTGAGGATATTGACGAATTATATGCTCTATTGGCCATAGAGAATGTTGCGGAATTTTACTATGATGCAGTTTTTTCATATTTCATTGAAACGACTGATTTATCTCTTGAAGACGG
664157_30677280_HHV-7_JI_U43400.1_144861bp_1_680	TTACTATGATGCAGTTTTTTCATATTTCATTGAAACGACTGATTTATCTCTTGAAGACGGAGATGCAACAATTTTAATTTTAAAGACTTACAAAGATCCAGATATAGCTCTTAGTTTGAA
664157_30677280_HHV-7_JI_U43400.1_144861bp_1_681	AGATGCAACAATTTTAATTTTAAAGACTTACAAAGATCCAGATATAGCTCTTAGTTTGAATGATTTTTTAACAATGTATTCATCTACATCCTCAACAAAGACAGCGGAAACAAACACTTT
664157_30677280_HHV-7_JI_U43400.1_144861bp_1_682	TGATTTTTTAACAATGTATTCATCTACATCCTCAACAAAGACAGCGGAAACAAACACTTTAATTTCAAAACAATCACCAAGCAAACGCAAACAAGAAAAAACCAGTCTAAATTCAAATTC
664157_30677280_HHV-7_JI_U43400.1_144861bp_1_683	AATTTCAAAACAATCACCAAGCAAACGCAAACAAGAAAAAACCAGTCTAAATTCAAATTCTCTAGAAAAAAAAAGGAAGCAGGGCTCATCACTCAAATACTATAACAATGAAGTAGATTT
664157_30677280_HHV-7_JI_U43400.1_144861bp_1_684	TCTAGAAAAAAAAAGGAAGCAGGGCTCATCACTCAAATACTATAACAATGAAGTAGATTTAGTACCAAGTTTTTATGAGCTTAGACCTCAATTTAACAATATTTTATTTGAGCTTTCTAA
664157_30677280_HHV-7_JI_U43400.1_144861bp_1_685	AGTACCAAGTTTTTATGAGCTTAGACCTCAATTTAACAATATTTTATTTGAGCTTTCTAATTTCCCAATTGTAAAGGAAAATGTAAATTGGACCCTTTACATACAGAAATTTGCAACAAA

664157_30677280_HHV-7_JI_U43400.1_144861bp_1_686	TTTCCCAATTGTAAAGGAAAATGTAAATTGGACCCTTTACATACAGAAATTTGCAACAAAGTCTACACAGCCATTTACAAAACCTTTTATATGGAATAGGGTATTCCATCTATTTTCTCA
664157_30677280_HHV-7_JI_U43400.1_144861bp_1_687	GTCTACACAGCCATTTACAAAACCTTTTATATGGAATAGGGTATTCCATCTATTTTCTCAAGTGGTTGACGCCTTAATTATGATTAAAAACGATCATTGGGATGAGACACAACAGCAAAA
664157_30677280_HHV-7_JI_U43400.1_144861bp_1_688	AGTGGTTGACGCCTTAATTATGATTAAAAACGATCATTGGGATGAGACACAACAGCAAAAACAATTCTTCACACATTTCTTGCCGTTCAAAGAATTTTCTGAGGAATTTGAAAACGCTAT
664157_30677280_HHV-7_JI_U43400.1_144861bp_1_689	ACAATTCTTCACACATTTCTTGCCGTTCAAAGAATTTTCTGAGGAATTTGAAAACGCTATAGAGGCTTGCCGAGAAAATAATCTTGATCTAATATTGCTTTATAAAAACTATCTTTCGAA
664157_30677280_HHV-7_JI_U43400.1_144861bp_1_690	AGAGGCTTGCCGAGAAAATAATCTTGATCTAATATTGCTTTATAAAAACTATCTTTCGAAAACTACTGCATTCAAAAACCTTGAAAGAATTTTATTAACGAAGTTTAGCGCCATTGTCAG
664157_30677280_HHV-7_JI_U43400.1_144861bp_1_691	AACTACTGCATTCAAAAACCTTGAAAGAATTTTATTAACGAAGTTTAGCGCCATTGTCAGTCCGGTGCATGAAAAACACTACACGCTTGTAAACACATGGCTAACTAACTTAATACAAAA
664157_30677280_HHV-7_JI_U43400.1_144861bp_1_692	TCCGGTGCATGAAAAACACTACACGCTTGTAAACACATGGCTAACTAACTTAATACAAAAACTAGTCAAACATCCCGAGGATACCAATGCTTTCATCAATGACTACGTGTTAAAAAACCC
664157_30677280_HHV-7_JI_U43400.1_144861bp_1_693	ACTAGTCAAACATCCCGAGGATACCAATGCTTTCATCAATGACTACGTGTTAAAAAACCCCTTAAATCATTTCATTTGTTTGAATAAGAAGGAAAAGCAGAGCATCGCTCTACTACTGAA
664157_30677280_HHV-7_JI_U43400.1_144861bp_1_694	CTTAAATCATTTCATTTGTTTGAATAAGAAGGAAAAGCAGAGCATCGCTCTACTACTGAATAAAAAAAGAATGAGTATGCTAAAAGATGTGGAAATCGAAAAGAATGGTTTTGTTCAACT
664157_30677280_HHV-7_JI_U43400.1_144861bp_1_695	TAAAAAAAGAATGAGTATGCTAAAAGATGTGGAAATCGAAAAGAATGGTTTTGTTCAACTCCAAGCATTCATCGAGAACATAGGAGAAGCTCCAGCAAATTATTTAGATCCGGAAAATGC
664157_30677280_HHV-7_JI_U43400.1_144861bp_1_696	CCAAGCATTCATCGAGAACATAGGAGAAGCTCCAGCAAATTATTTAGATCCGGAAAATGCACGCAAAGTGAATGTTGAAGAAGTCTCAGAAAAAGACATCCCAACATTATCCACAGATAA
664157_30677280_HHV-7_JI_U43400.1_144861bp_1_697	ACGCAAAGTGAATGTTGAAGAAGTCTCAGAAAAAGACATCCCAACATTATCCACAGATAAAGTTTCCATACCCAATGAAAGTATGTTCACATCAAATAAAAAACACAGCATAGAAAAATT

664157_30677280_HHV-7_JI_U43400.1_144861bp_1_698	AGTTTCCATACCCAATGAAAGTATGTTCACATCAAATAAAAAACACAGCATAGAAAAATTAATACATGCTAAGCTGAAAGCCATTTTGAGTACAATGGGGCAAAGATTAACTAGAATTAT
664157_30677280_HHV-7_JI_U43400.1_144861bp_1_699	AATACATGCTAAGCTGAAAGCCATTTTGAGTACAATGGGGCAAAGATTAACTAGAATTATTCAAGAAAATTATAATAACATCGCTGCAGGTTTTCTGCCGGTGAACGATCTTAATAATCT
664157_30677280_HHV-7_JI_U43400.1_144861bp_1_700	TCAAGAAAATTATAATAACATCGCTGCAGGTTTTCTGCCGGTGAACGATCTTAATAATCTGTTTGCCTATTTGGTCAAACTCTATTTTGATGTCTATAGCATCACCATTAACGGATTTGT
664157_30677280_HHV-7_JI_U43400.1_144861bp_1_701	GTTTGCCTATTTGGTCAAACTCTATTTTGATGTCTATAGCATCACCATTAACGGATTTGTGGTGGAAAACGAATTGATAAAAAATATTGAACAAATTTACGACAATACGCAATATCTGAG
664157_30677280_HHV-7_JI_U43400.1_144861bp_1_702	GGTGGAAAACGAATTGATAAAAAATATTGAACAAATTTACGACAATACGCAATATCTGAGATTTGGATTGACACGCTTCAATATGCAAAATTTGACACCGTTTACTATATCTGTCCGCAA
664157_30677280_HHV-7_JI_U43400.1_144861bp_1_703	ATTTGGATTGACACGCTTCAATATGCAAAATTTGACACCGTTTACTATATCTGTCCGCAAAATGTTCCTGGATTTTTTTCTATCACAAAAAACTCTGATAGATAGAGCTGAAGAAATTAT
664157_30677280_HHV-7_JI_U43400.1_144861bp_1_704	AATGTTCCTGGATTTTTTTCTATCACAAAAAACTCTGATAGATAGAGCTGAAGAAATTATAGAGAACCTTGAGTTTAAATCAGTTACACCAGAAGGAAAACAAAAACTTGCCACAAAGAA
664157_30677280_HHV-7_JI_U43400.1_144861bp_1_705	AGAGAACCTTGAGTTTAAATCAGTTACACCAGAAGGAAAACAAAAACTTGCCACAAAGAATATGCTCAGAGAACAATTAGAACAGTTGAATGCTATGGATGTGGATGATACAATAAATCT
664157_30677280_HHV-7_JI_U43400.1_144861bp_1_706	TATGCTCAGAGAACAATTAGAACAGTTGAATGCTATGGATGTGGATGATACAATAAATCTGAAAACAGACACATTAACACATCAAGTATTATTTTCAGACCAAGAATTACGCATGATACA
664157_30677280_HHV-7_JI_U43400.1_144861bp_1_707	GAAAACAGACACATTAACACATCAAGTATTATTTTCAGACCAAGAATTACGCATGATACAAGACTTCATTTTACAACTCTCCATTCACAATATTCCAAGCATTAACTTTGTGAAATCTTT
664157_30677280_HHV-7_JI_U43400.1_144861bp_1_708	AGACTTCATTTTACAACTCTCCATTCACAATATTCCAAGCATTAACTTTGTGAAATCTTTGAAATTACATATTATTTTAGAAAAAAGACCTGATATACTATTAGCTCTACAAGAAAAAGT
664157_30677280_HHV-7_JI_U43400.1_144861bp_1_709	GAAATTACATATTATTTTAGAAAAAAGACCTGATATACTATTAGCTCTACAAGAAAAAGTCCAGAATATTCTATATTTTTATTTTCAAGATCTAGTTAACGAGATACCTGCTCAAGAAAA

664157_30677280_HHV-7_JI_U43400.1_144861bp_1_710	CCAGAATATTCTATATTTTTATTTTCAAGATCTAGTTAACGAGATACCTGCTCAAGAAAATGTTTTGTCAACAATGTTATTTATAATAGAGCTTTTTCCAGCCGACAGTAGAATACATCT
664157_30677280_HHV-7_JI_U43400.1_144861bp_1_711	TGTTTTGTCAACAATGTTATTTATAATAGAGCTTTTTCCAGCCGACAGTAGAATACATCTACTAGAAACCGGATATATTTCCAGACATATTGTAAAAAAATGGCTAAACATGAAATCATT
664157_30677280_HHV-7_JI_U43400.1_144861bp_1_712	ACTAGAAACCGGATATATTTCCAGACATATTGTAAAAAAATGGCTAAACATGAAATCATTGCAAGATGCTGAGGATTTAATTCGATTTATAAATATTAATAAAGAACAACTAGGAAAATT
664157_30677280_HHV-7_JI_U43400.1_144861bp_1_713	GCAAGATGCTGAGGATTTAATTCGATTTATAAATATTAATAAAGAACAACTAGGAAAATTTGAACATCAGCCATTTGGAAAAGAAATTCAAAAACTAATTGAAAAAATACACTTGTTCTA
664157_30677280_HHV-7_JI_U43400.1_144861bp_1_714	TGAACATCAGCCATTTGGAAAAGAAATTCAAAAACTAATTGAAAAAATACACTTGTTCTATAAGCAAAAAGTAATCGAGTATCAGGAAGATGTCTGGAGTGAAATGGCTAAAAATATAAT
664157_30677280_HHV-7_JI_U43400.1_144861bp_1_715	TAAGCAAAAAGTAATCGAGTATCAGGAAGATGTCTGGAGTGAAATGGCTAAAAATATAATTTTAACTTCACCCTCTGAATTATCTCAATTTTTAGCTTCAGCTCCCACTCAACGTATCAT
664157_30677280_HHV-7_JI_U43400.1_144861bp_1_716	TTTAACTTCACCCTCTGAATTATCTCAATTTTTAGCTTCAGCTCCCACTCAACGTATCATACAAAAACACAAAAATAATTTAGATCAGAAACTTTTAATACATATGGAAAACCAAGCCAA
664157_30677280_HHV-7_JI_U43400.1_144861bp_1_717	ACAAAAACACAAAAATAATTTAGATCAGAAACTTTTAATACATATGGAAAACCAAGCCAAGCAAGCAATGGAAGATGACAAAAAAAGAGTTGCCTGTTCTAAAATTAATCTGGAACGACA
664157_30677280_HHV-7_JI_U43400.1_144861bp_1_718	GCAAGCAATGGAAGATGACAAAAAAAGAGTTGCCTGTTCTAAAATTAATCTGGAACGACACCTGAATGATTTACTGCTCTTATTGAAAGACAGACAATTTGCTTCCATACAGGCTTCTGT
664157_30677280_HHV-7_JI_U43400.1_144861bp_1_719	CCTGAATGATTTACTGCTCTTATTGAAAGACAGACAATTTGCTTCCATACAGGCTTCTGTTTTGATTGTGTGCGAAAATATATTTAAAACGATACCAGATGATAACCTAATTATTCAATT
664157_30677280_HHV-7_JI_U43400.1_144861bp_1_720	TTTGATTGTGTGCGAAAATATATTTAAAACGATACCAGATGATAACCTAATTATTCAATTTTCACATGCTCTGCTTTCAGTTTTACTTGACATTGAAAAGGATTTAAAAAGCTATTCATC
664157_30677280_HHV-7_JI_U43400.1_144861bp_1_721	TTCACATGCTCTGCTTTCAGTTTTACTTGACATTGAAAAGGATTTAAAAAGCTATTCATCAGAAATATTAGAGAAAATACTAATAAATAGGCCCCTCGAAACCAGTAGATTATTAGTGTT

664157_30677280_HHV-7_JI_U43400.1_144861bp_1_722	AGAAATATTAGAGAAAATACTAATAAATAGGCCCCTCGAAACCAGTAGATTATTAGTGTTTAAAGACGCTTATGGTAATCTGAAAGAGTTTTTAAACGCCTTAAAACAATCACTTTTTGC
664157_30677280_HHV-7_JI_U43400.1_144861bp_1_723	TAAAGACGCTTATGGTAATCTGAAAGAGTTTTTAAACGCCTTAAAACAATCACTTTTTGCCACAGCGGATGTTCAAAACAAGGCTGATTTTCTTATCCAAATTTTAGATTTTACCTATAA
664157_30677280_HHV-7_JI_U43400.1_144861bp_1_724	CACAGCGGATGTTCAAAACAAGGCTGATTTTCTTATCCAAATTTTAGATTTTACCTATAAATTTAGACATAAGACAAATAAAGGTAAACTTCTACATTCCATTTATAATGAGGATTTCAA
664157_30677280_HHV-7_JI_U43400.1_144861bp_1_725	ATTTAGACATAAGACAAATAAAGGTAAACTTCTACATTCCATTTATAATGAGGATTTCAAACTATACGAAGAAACATTAACAGAGTTAAGAAAAAAAGCAACAGATGCAAAAGAGTCGTT
664157_30677280_HHV-7_JI_U43400.1_144861bp_1_726	ACTATACGAAGAAACATTAACAGAGTTAAGAAAAAAAGCAACAGATGCAAAAGAGTCGTTAACTAAACTTTTTAAAGCATCCGAACAAAAGATCGAGCTGTCGCGTACGATTCCGTTAAA
664157_30677280_HHV-7_JI_U43400.1_144861bp_1_727	AACTAAACTTTTTAAAGCATCCGAACAAAAGATCGAGCTGTCGCGTACGATTCCGTTAAAGGAAATCTACCTGAACATAGAAACTGTTAATTTCCAAGGTTATGGCAACGTAGTTTTTCG
664157_30677280_HHV-7_JI_U43400.1_144861bp_1_728	GGAAATCTACCTGAACATAGAAACTGTTAATTTCCAAGGTTATGGCAACGTAGTTTTTCGAGAAAGTGCTTTTAAACGCGCAATAGAGGTAGAAATAAAAAATTACGAAATGAAATTAAA
664157_30677280_HHV-7_JI_U43400.1_144861bp_1_729	AGAAAGTGCTTTTAAACGCGCAATAGAGGTAGAAATAAAAAATTACGAAATGAAATTAAACGATCTAATAAAACACTTTAATTCACACTTAAAAACAAAAATTGACCACATACAGATTCT
664157_30677280_HHV-7_JI_U43400.1_144861bp_1_730	CGATCTAATAAAACACTTTAATTCACACTTAAAAACAAAAATTGACCACATACAGATTCTTAATCTATCTTTTGATAACAAATGGAAAGATTTTGTCTCCAAGTCAAAAATATCTTTCCC
664157_30677280_HHV-7_JI_U43400.1_144861bp_1_731	TAATCTATCTTTTGATAACAAATGGAAAGATTTTGTCTCCAAGTCAAAAATATCTTTCCCACCAGAACTGACAATAAGTTCACAAGAGTTGATCAAGGATCCCATTAAAGTTATAACTGA
664157_30677280_HHV-7_JI_U43400.1_144861bp_1_732	ACCAGAACTGACAATAAGTTCACAAGAGTTGATCAAGGATCCCATTAAAGTTATAACTGAAACTCTAAACAAAGCCTCAAACGATTTAGCGTATGTGATTAGTGAGAAAATATTGAAGTG
664157_30677280_HHV-7_JI_U43400.1_144861bp_1_733	AACTCTAAACAAAGCCTCAAACGATTTAGCGTATGTGATTAGTGAGAAAATATTGAAGTGGTTAATAGTTTTTGTCAAAGAACTGAATACCTTTTTTGTAGCTACAATGTCAGAATTTGG

664157_30677280_HHV-7_JI_U43400.1_144861bp_1_734	GTTAATAGTTTTTGTCAAAGAACTGAATACCTTTTTTGTAGCTACAATGTCAGAATTTGGAGAAGTTATCCCCTTTGACTATAAACATTTCAGAGCTTTGGAATACGAAATTAATTCTAA
664157_30677280_HHV-7_JI_U43400.1_144861bp_1_735	AGAAGTTATCCCCTTTGACTATAAACATTTCAGAGCTTTGGAATACGAAATTAATTCTAAGTACATAGAGATTGAAAATAAAATAATCTGCAACGAAATTATCGAAAATACTGACAATAT
664157_30677280_HHV-7_JI_U43400.1_144861bp_1_736	GTACATAGAGATTGAAAATAAAATAATCTGCAACGAAATTATCGAAAATACTGACAATATAGAAAAACTCTCAACCTTGATAAAACAAATAGATCCAAATCGTATTGCTGGTGGTAAACA
664157_30677280_HHV-7_JI_U43400.1_144861bp_1_737	AGAAAAACTCTCAACCTTGATAAAACAAATAGATCCAAATCGTATTGCTGGTGGTAAACAGAAATTTCAGGATTATCTGAGCAAAATTCTAACAGCTGAAACGAACCAGCAACAAACACG
664157_30677280_HHV-7_JI_U43400.1_144861bp_1_738	GAAATTTCAGGATTATCTGAGCAAAATTCTAACAGCTGAAACGAACCAGCAACAAACACGCTATAAAGAACAGTTAAAAAAACAGTACTTTGACCTTTTAGATAACATCGCCCATTTCCG
664157_30677280_HHV-7_JI_U43400.1_144861bp_1_739	CTATAAAGAACAGTTAAAAAAACAGTACTTTGACCTTTTAGATAACATCGCCCATTTCCGGTTCGCATTCGATTTTAACCATCAACAAAATTTAATTTTAAAACTGAAAGACAAATTCAA
664157_30677280_HHV-7_JI_U43400.1_144861bp_1_740	GTTCGCATTCGATTTTAACCATCAACAAAATTTAATTTTAAAACTGAAAGACAAATTCAAAACTCTTAGAACAGACACTGTATTTGAAAGATTTCCAAATTTAGATGATACTTTTGTCAG
664157_30677280_HHV-7_JI_U43400.1_144861bp_1_741	AACTCTTAGAACAGACACTGTATTTGAAAGATTTCCAAATTTAGATGATACTTTTGTCAGTTCAATGAATGTCGAGAACTTTTTGCAGGCACTTGAGGCTTTAAGCCATTTCGTGCAGGC
664157_30677280_HHV-7_JI_U43400.1_144861bp_1_742	TTCAATGAATGTCGAGAACTTTTTGCAGGCACTTGAGGCTTTAAGCCATTTCGTGCAGGCAGCACAAAATTTTCTACAAAACGTTTTAACAGAGCAAGCGGATTTATTTCCACAGACGAA
664157_30677280_HHV-7_JI_U43400.1_144861bp_1_743	AGCACAAAATTTTCTACAAAACGTTTTAACAGAGCAAGCGGATTTATTTCCACAGACGAATTTCATTCCTGTCGAACTTTCCACCGTCAAAACAATTCCAAAATCAGATATAAATTTACG
664157_30677280_HHV-7_JI_U43400.1_144861bp_1_744	TTTCATTCCTGTCGAACTTTCCACCGTCAAAACAATTCCAAAATCAGATATAAATTTACGTATGAAAATACACACACCCCAAACTTTTTTTCAGGTTGATTCAGTTTTTAATACACAGTT
664157_30677280_HHV-7_JI_U43400.1_144861bp_1_745	TATGAAAATACACACACCCCAAACTTTTTTTCAGGTTGATTCAGTTTTTAATACACAGTTGATAGTTGATGAGAAAGGAATTCCAGTCCAATTTTACAATGTTTTCCACAATATTGTTTT

664157_30677280_HHV-7_JI_U43400.1_144861bp_1_746	GATAGTTGATGAGAAAGGAATTCCAGTCCAATTTTACAATGTTTTCCACAATATTGTTTTCAAGTTTTTTGCTCTAAATTATAAGAAAATTATCGTACCTGATAAAGTGCTGAACTTAGT
664157_30677280_HHV-7_JI_U43400.1_144861bp_1_747	CAAGTTTTTTGCTCTAAATTATAAGAAAATTATCGTACCTGATAAAGTGCTGAACTTAGTATCAACCAAGTATAAGATCTTAACCACATTAAAAAGCATTCTGAGTGTTGTAAAAAGCTT
664157_30677280_HHV-7_JI_U43400.1_144861bp_1_748	ATCAACCAAGTATAAGATCTTAACCACATTAAAAAGCATTCTGAGTGTTGTAAAAAGCTTTTGGAAAGAGATTATAAATTTCGATTTAACTTCTTATTTCCAAGGGAAAGCAGAATTTAC
664157_30677280_HHV-7_JI_U43400.1_144861bp_1_749	TTGGAAAGAGATTATAAATTTCGATTTAACTTCTTATTTCCAAGGGAAAGCAGAATTTACTTTTCAAAATGTTTTCCCAATAATTAATCTTAAAATATTTATTTACATTATTACTCAGGC
664157_30677280_HHV-7_JI_U43400.1_144861bp_1_750	TTTTCAAAATGTTTTCCCAATAATTAATCTTAAAATATTTATTTACATTATTACTCAGGCCTGGTCAGTCACATCTGATGAAACACAGCATTCGTTTGAACTGCCACTAGAAAAATTTTC
664157_30677280_HHV-7_JI_U43400.1_144861bp_1_751	CTGGTCAGTCACATCTGATGAAACACAGCATTCGTTTGAACTGCCACTAGAAAAATTTTCTCTTTTAATTATAGCAAATAATCCAGAGTTTCTTTTTGGTTCTCTGCAGTGTCCAGTGGA
664157_30677280_HHV-7_JI_U43400.1_144861bp_1_752	TCTTTTAATTATAGCAAATAATCCAGAGTTTCTTTTTGGTTCTCTGCAGTGTCCAGTGGACCTAGCTATTAATTCTCTAATACCCTTATTGGAAAAGAAAAAATATTTCACTGCATTTAC
664157_30677280_HHV-7_JI_U43400.1_144861bp_1_753	CCTAGCTATTAATTCTCTAATACCCTTATTGGAAAAGAAAAAATATTTCACTGCATTTACCATCTCTGACAATCCACCCAAGCTATCTATGGATGAATTAAAAATTGTGTGTTTGGATTT
664157_30677280_HHV-7_JI_U43400.1_144861bp_1_754	CATCTCTGACAATCCACCCAAGCTATCTATGGATGAATTAAAAATTGTGTGTTTGGATTTGAACACCTGGAGTGAAATAACATTAGAAAAATACACTTTTAAAAAGAACAGTTTGATGCA
664157_30677280_HHV-7_JI_U43400.1_144861bp_1_755	GAACACCTGGAGTGAAATAACATTAGAAAAATACACTTTTAAAAAGAACAGTTTGATGCAGTTATGTATGGGCAAAGAGAAATTTTTTATTTACCTTTTATCAGCGTTGGTTCTTCCTCA
664157_30677280_HHV-7_JI_U43400.1_144861bp_1_756	GTTATGTATGGGCAAAGAGAAATTTTTTATTTACCTTTTATCAGCGTTGGTTCTTCCTCAAAATTTTTTGAATTACATTTGGATTCAATACAAACCTTCGTGCTGTGCTCAGGATTCATT
664157_30677280_HHV-7_JI_U43400.1_144861bp_1_757	AAATTTTTTGAATTACATTTGGATTCAATACAAACCTTCGTGCTGTGCTCAGGATTCATTCCAACAACTCATTCAAGATTTATGTTTCGAGTATACACACCAGAATCACATAAAGCCCAT

664157_30677280_HHV-7_JI_U43400.1_144861bp_1_758	CCAACAACTCATTCAAGATTTATGTTTCGAGTATACACACCAGAATCACATAAAGCCCATTTCATTAAATCTCCAAGAACCAAATGCATTAAAACATGGTGAAAGAATCCTTTCGAAATT
664157_30677280_HHV-7_JI_U43400.1_144861bp_1_759	TTCATTAAATCTCCAAGAACCAAATGCATTAAAACATGGTGAAAGAATCCTTTCGAAATTCGTGCTGGAAAAAAATGCAAACACTTCTCTTTTTAGTATCTTTTTAGGCAAGCAGTTTTT
664157_30677280_HHV-7_JI_U43400.1_144861bp_1_760	CGTGCTGGAAAAAAATGCAAACACTTCTCTTTTTAGTATCTTTTTAGGCAAGCAGTTTTTATTAGATTATCTATTATTTTCATACTTAACCGCAACTGAAATGACTTTTTCATATTATGT
664157_30677280_HHV-7_JI_U43400.1_144861bp_1_761	ATTAGATTATCTATTATTTTCATACTTAACCGCAACTGAAATGACTTTTTCATATTATGTGGATTCCATCAAAAATTTTCTTCTTACGATCCGACATTTGGAAAATGTCCAGCAAAACGT
664157_30677280_HHV-7_JI_U43400.1_144861bp_1_762	GGATTCCATCAAAAATTTTCTTCTTACGATCCGACATTTGGAAAATGTCCAGCAAAACGTAGACTTTAGAACGATATTGCAATCTCGAAATTTCGATCTAAAATATCTTTTAACACAATC
664157_30677280_HHV-7_JI_U43400.1_144861bp_1_763	AGACTTTAGAACGATATTGCAATCTCGAAATTTCGATCTAAAATATCTTTTAACACAATCATGGACACAAAATGTTTTAGAACAGTCCATCTTTCACGTGCAACTTGATAAAATCATTGC
664157_30677280_HHV-7_JI_U43400.1_144861bp_1_764	ATGGACACAAAATGTTTTAGAACAGTCCATCTTTCACGTGCAACTTGATAAAATCATTGCTGACATCAAACAACCACAACTAAGTCTGAAGAAAATTCCACTGGTTTTGTTTAACGGTGA
664157_30677280_HHV-7_JI_U43400.1_144861bp_1_765	TGACATCAAACAACCACAACTAAGTCTGAAGAAAATTCCACTGGTTTTGTTTAACGGTGACAACGAAGTCGTGTCAACTTATGTGCCCCCTGAACAAGCAAGCCAGACAGAGCAGAGCTT
664157_30677280_HHV-7_JI_U43400.1_144861bp_1_766	CAACGAAGTCGTGTCAACTTATGTGCCCCCTGAACAAGCAAGCCAGACAGAGCAGAGCTTTCGCATTAAGAATATTTTCCCAAATCCTGTGCAAGAGTATAGTAGCAAAAATGTGATTCT
664157_30677280_HHV-7_JI_U43400.1_144861bp_1_767	TCGCATTAAGAATATTTTCCCAAATCCTGTGCAAGAGTATAGTAGCAAAAATGTGATTCTTTTTACGAACTATCCAAAAAACACCAAATTTTTATTTAACTCTCCTCCACCTAAAACAGC
664157_30677280_HHV-7_JI_U43400.1_144861bp_1_768	TTTTACGAACTATCCAAAAAACACCAAATTTTTATTTAACTCTCCTCCACCTAAAACAGCGGCAAAAAGTTACAAACTACCAGACACTACCGATGACATAAACACGGAAACATTATCAAG
664157_30677280_HHV-7_JI_U43400.1_144861bp_1_769	GGCAAAAAGTTACAAACTACCAGACACTACCGATGACATAAACACGGAAACATTATCAAGTCCCACGATTCAAAGAATCCCTATCAAAGGACTTGTACCCAAAGAAAACGAAATTGTCTT

664157_30677280_HHV-7_JI_U43400.1_144861bp_1_770	TCCCACGATTCAAAGAATCCCTATCAAAGGACTTGTACCCAAAGAAAACGAAATTGTCTTTTTACCAGAAAAAAACACTGCACACACAGACTCTAAAGAGACAAAAACGCATTTAATAGA
664157_30677280_HHV-7_JI_U43400.1_144861bp_1_771	TTTACCAGAAAAAAACACTGCACACACAGACTCTAAAGAGACAAAAACGCATTTAATAGACACCTTCAATATATTGTCTCAAACAAAGGGTGAAATCAAAACATTTTCTACAGATTTTGA
664157_30677280_HHV-7_JI_U43400.1_144861bp_1_772	CACCTTCAATATATTGTCTCAAACAAAGGGTGAAATCAAAACATTTTCTACAGATTTTGATCAAACAATTTCCAAATTAAAACATTTGTACTTTTAAGATGATTCGCTAACTTTGCTATT
664157_30677280_HHV-7_JI_U43400.1_144861bp_1_773	TCAAACAATTTCCAAATTAAAACATTTGTACTTTTAAGATGATTCGCTAACTTTGCTATTTAATCTATGCAAAGCAAGCATGCGTAAAAAATCAAGTCTGAAGGCTGCTTTTTCAATGTT
664157_30677280_HHV-7_JI_U43400.1_144861bp_1_774	TAATCTATGCAAAGCAAGCATGCGTAAAAAATCAAGTCTGAAGGCTGCTTTTTCAATGTTTCCGGTATTTTGTTTTGCATATTTTGCTAGAAACGTCCCTGTGACAGGGTGTGAACCCAT
664157_30677280_HHV-7_JI_U43400.1_144861bp_1_775	TCCGGTATTTTGTTTTGCATATTTTGCTAGAAACGTCCCTGTGACAGGGTGTGAACCCATAGAAGAATGCAAGCCTAGAATATTCGTAAACATTTGTTGCTTTTTCTTTTCTTCTTCTTT
664157_30677280_HHV-7_JI_U43400.1_144861bp_1_776	AGAAGAATGCAAGCCTAGAATATTCGTAAACATTTGTTGCTTTTTCTTTTCTTCTTCTTTTTTTGAACTGTTGCCAGAAATCTGGTTGATTTGAGCAGTGATTTCATCACCACGTGTCGC
664157_30677280_HHV-7_JI_U43400.1_144861bp_1_777	TTTTGAACTGTTGCCAGAAATCTGGTTGATTTGAGCAGTGATTTCATCACCACGTGTCGCAGCCATGTTGCGTGCACTTAGCTAATTGAGAAATTTTACAGCCTGTACAAATTTCTCCAC
664157_30677280_HHV-7_JI_U43400.1_144861bp_1_778	AGCCATGTTGCGTGCACTTAGCTAATTGAGAAATTTTACAGCCTGTACAAATTTCTCCACAGTCCTCTAGATCAAGACACGTTTCTCTGTAGATATTTTGACAATCTTGGCATTGAAATT
664157_30677280_HHV-7_JI_U43400.1_144861bp_1_779	AGTCCTCTAGATCAAGACACGTTTCTCTGTAGATATTTTGACAATCTTGGCATTGAAATTCTGTGGAGTGAGAAATTAGTTTCAACAGTTTATTTATCGTTAGATTCTGCAGATGGATCA
664157_30677280_HHV-7_JI_U43400.1_144861bp_1_780	CTGTGGAGTGAGAAATTAGTTTCAACAGTTTATTTATCGTTAGATTCTGCAGATGGATCATAGAGTAACAAGTTCTGCAAGAACATGCAACAATGGCATCAAATTTAACACTATCGTTTA
664157_30677280_HHV-7_JI_U43400.1_144861bp_1_781	TAGAGTAACAAGTTCTGCAAGAACATGCAACAATGGCATCAAATTTAACACTATCGTTTAAAATAGTGCAAGCGGAATTAGTTCCAATTATAGCTTTCCATGATACACTTTTTACTTGAA

664157_30677280_HHV-7_JI_U43400.1_144861bp_1_782	AAATAGTGCAAGCGGAATTAGTTCCAATTATAGCTTTCCATGATACACTTTTTACTTGAATGCCTGAAACACTATATTCATACAATTCATATACTTTTTCAAGTGTCAAACGTTGCCCGC
664157_30677280_HHV-7_JI_U43400.1_144861bp_1_783	TGCCTGAAACACTATATTCATACAATTCATATACTTTTTCAAGTGTCAAACGTTGCCCGCCACATAATGAACAATAAAGCAAATCTGTGTGCATACTGTAAATAATATTTTTTTCCTGTC
664157_30677280_HHV-7_JI_U43400.1_144861bp_1_784	CACATAATGAACAATAAAGCAAATCTGTGTGCATACTGTAAATAATATTTTTTTCCTGTCTATCTCGATAATAAAACATCGAGCTTAAAGAAAAAGTTTGTTTCCCTTTAAGCCTCTCTT
664157_30677280_HHV-7_JI_U43400.1_144861bp_1_785	TATCTCGATAATAAAACATCGAGCTTAAAGAAAAAGTTTGTTTCCCTTTAAGCCTCTCTTTGCCGGAATTTAAACAATGTCCACATTTAGAACATATCACAGTGAGATAACTATCAATTT
664157_30677280_HHV-7_JI_U43400.1_144861bp_1_786	TGCCGGAATTTAAACAATGTCCACATTTAGAACATATCACAGTGAGATAACTATCAATTTTAGCATATCTGAGACACTTTGTTTTGCAAAAACATAATACCGGTAAAGAGATGATACTTG
664157_30677280_HHV-7_JI_U43400.1_144861bp_1_787	TAGCATATCTGAGACACTTTGTTTTGCAAAAACATAATACCGGTAAAGAGATGATACTTGAAGTTAAGCTTCTTTTCAAGACTGTCGTCAAAGCAAAAGTTAGAATTTTTGGAAGTTGAT
664157_30677280_HHV-7_JI_U43400.1_144861bp_1_788	AAGTTAAGCTTCTTTTCAAGACTGTCGTCAAAGCAAAAGTTAGAATTTTTGGAAGTTGATGTTTTTCTTGTTTGTTCAAAACAATCGGCACCAAGTTATTATTTTCTAGCGGGATTAAAA
664157_30677280_HHV-7_JI_U43400.1_144861bp_1_789	GTTTTTCTTGTTTGTTCAAAACAATCGGCACCAAGTTATTATTTTCTAGCGGGATTAAAAAACGATCGAAACTGTAACAATAATTTTCTATCATGGCCATGGCCATAGCAAAGATAAAAT
664157_30677280_HHV-7_JI_U43400.1_144861bp_1_790	AACGATCGAAACTGTAACAATAATTTTCTATCATGGCCATGGCCATAGCAAAGATAAAATTTCCACATGGAACTGTTAAAGAACAACCATGAAATATATCTTGATATGTCAGGTTGCTTG
664157_30677280_HHV-7_JI_U43400.1_144861bp_1_791	TTCCACATGGAACTGTTAAAGAACAACCATGAAATATATCTTGATATGTCAGGTTGCTTGCTCTATGAGCGTATCGCAGTGTGATGTCATTACATTGTTCAAAGTTACGTAAACAAAAGA
664157_30677280_HHV-7_JI_U43400.1_144861bp_1_792	CTCTATGAGCGTATCGCAGTGTGATGTCATTACATTGTTCAAAGTTACGTAAACAAAAGAGTTTCATACAAGGAATATTACAATGAGTTGAAATTGGCAACATTAACTGTTGCTGAAATA
664157_30677280_HHV-7_JI_U43400.1_144861bp_1_793	GTTTCATACAAGGAATATTACAATGAGTTGAAATTGGCAACATTAACTGTTGCTGAAATAAATAATAATAGAGATCATTAAAAGACACAAACGACGGTATTTCCAACAACTGATTTAACT

664157_30677280_HHV-7_JI_U43400.1_144861bp_1_794	AATAATAATAGAGATCATTAAAAGACACAAACGACGGTATTTCCAACAACTGATTTAACTTATGTATTGGGTATTGTTTGAATCTCTCCACAATTTGTTTCCAAGTAAGACGTTGAATTT
664157_30677280_HHV-7_JI_U43400.1_144861bp_1_795	TATGTATTGGGTATTGTTTGAATCTCTCCACAATTTGTTTCCAAGTAAGACGTTGAATTTTGTAAAGAGCCAGAATTTTTAGAACCTTGCATTGCCTGTACAAAAAATACAAATATGCCA
664157_30677280_HHV-7_JI_U43400.1_144861bp_1_796	TGTAAAGAGCCAGAATTTTTAGAACCTTGCATTGCCTGTACAAAAAATACAAATATGCCAAAAGCAAAAAGAAATTTTTTGTCATCATCATTTTATTAAAAATAGCTTTAATCTGATTTC
664157_30677280_HHV-7_JI_U43400.1_144861bp_1_797	AAAGCAAAAAGAAATTTTTTGTCATCATCATTTTATTAAAAATAGCTTTAATCTGATTTCTTGAATCCAGATCACACTTTAACCAAACTTTTAAAAACTTCCGGTAGAAATTATCAGTTA
664157_30677280_HHV-7_JI_U43400.1_144861bp_1_798	TTGAATCCAGATCACACTTTAACCAAACTTTTAAAAACTTCCGGTAGAAATTATCAGTTAAATCTTTACTCAAAAACTTTTTATTCGTTACGAACATCGATAATACGCAATTTTCAGATT
664157_30677280_HHV-7_JI_U43400.1_144861bp_1_799	AATCTTTACTCAAAAACTTTTTATTCGTTACGAACATCGATAATACGCAATTTTCAGATTTGCTGACATCTCCAATAAGAAATAACTGCAAATGACTGTACGAACCATGTTTACACAGCG
664157_30677280_HHV-7_JI_U43400.1_144861bp_1_800	TGCTGACATCTCCAATAAGAAATAACTGCAAATGACTGTACGAACCATGTTTACACAGCGGAGAAATCAGCCGACAAAGCAAATTCCTTAAAGATGACATAGCATACAATTAGTAAGGCG
664157_30677280_HHV-7_JI_U43400.1_144861bp_1_801	GAGAAATCAGCCGACAAAGCAAATTCCTTAAAGATGACATAGCATACAATTAGTAAGGCGGCAACCAAAAAACCACAAAGAATATTATTTCTCCGTGCAAAAATAGGAACAGTAACTGGG
664157_30677280_HHV-7_JI_U43400.1_144861bp_1_802	GCAACCAAAAAACCACAAAGAATATTATTTCTCCGTGCAAAAATAGGAACAGTAACTGGGAATACTGCTGCGTCTTTTGTGTTTCCCATGTAATTTATACTCCTGAATGATGTCATCTCT
664157_30677280_HHV-7_JI_U43400.1_144861bp_1_803	AATACTGCTGCGTCTTTTGTGTTTCCCATGTAATTTATACTCCTGAATGATGTCATCTCTCCTACTGACTTCAACTGTAACCTCAATGGTTTTTTTATGTGCCTTGCAGCTCTATGTTTC
664157_30677280_HHV-7_JI_U43400.1_144861bp_1_804	CCTACTGACTTCAACTGTAACCTCAATGGTTTTTTTATGTGCCTTGCAGCTCTATGTTTCTCTAAATCTTGGCGCTCTAAAAATTTTTTCTTTAAGATTATCGCTTTAAGAAGAGCGATA
664157_30677280_HHV-7_JI_U43400.1_144861bp_1_805	TCTAAATCTTGGCGCTCTAAAAATTTTTTCTTTAAGATTATCGCTTTAAGAAGAGCGATATACTCAGATTCACTCTCGGGACCAAAAGCCACAAATTCAACTATAAAAGCCGCATCTCGG

664157_30677280_HHV-7_JI_U43400.1_144861bp_1_806	TACTCAGATTCACTCTCGGGACCAAAAGCCACAAATTCAACTATAAAAGCCGCATCTCGGTTTCGTCTAATAATGCACTTTGTCAGTACACCACTTGAAGAAGGTTTAATAGCTTCAATG
664157_30677280_HHV-7_JI_U43400.1_144861bp_1_807	TTTCGTCTAATAATGCACTTTGTCAGTACACCACTTGAAGAAGGTTTAATAGCTTCAATGTCATTCAAACACACAGATAAACACTCATTGACATTTAAGATGTTGCATTCTTTGACAATG
664157_30677280_HHV-7_JI_U43400.1_144861bp_1_808	TCATTCAAACACACAGATAAACACTCATTGACATTTAAGATGTTGCATTCTTTGACAATGTTCTGACTTTCTTGTTTTATGGGAGCGTGGAGATAACAAGACATGGAAACAGCACCTCCC
664157_30677280_HHV-7_JI_U43400.1_144861bp_1_809	TTCTGACTTTCTTGTTTTATGGGAGCGTGGAGATAACAAGACATGGAAACAGCACCTCCCGTATTTTTAAAAATAAATCTCGCTTCTTGTTTTGTCAAATCTTCCCAGAGACTCAGCAAA
664157_30677280_HHV-7_JI_U43400.1_144861bp_1_810	GTATTTTTAAAAATAAATCTCGCTTCTTGTTTTGTCAAATCTTCCCAGAGACTCAGCAAATATTCCAGGCTATATGCAAAATCCAATTTTAAAAGTATATCACATAAAGGAAATTTAGGA
664157_30677280_HHV-7_JI_U43400.1_144861bp_1_811	TATTCCAGGCTATATGCAAAATCCAATTTTAAAAGTATATCACATAAAGGAAATTTAGGATTTTTAGAAAATAAATTTTTATCTGTGACACGAAAGTCTGCGGGACCCAATTTTAAAACG
664157_30677280_HHV-7_JI_U43400.1_144861bp_1_812	TTTTTAGAAAATAAATTTTTATCTGTGACACGAAAGTCTGCGGGACCCAATTTTAAAACGCGGCACGTGGAAAGAATTAATTCATCGTACATCTTTTCTTTCAACATGTTTATTTTCTCT
664157_30677280_HHV-7_JI_U43400.1_144861bp_1_813	CGGCACGTGGAAAGAATTAATTCATCGTACATCTTTTCTTTCAACATGTTTATTTTCTCTTTAAAAATTTCTGCAGTTGTATATTATGTATTTAAATATTCCTGATGTTCTAAAATTCCA
664157_30677280_HHV-7_JI_U43400.1_144861bp_1_814	TTAAAAATTTCTGCAGTTGTATATTATGTATTTAAATATTCCTGATGTTCTAAAATTCCATTCACATTTAACAGTTTCGATAAAATTGAAGTGAGTAGTTTGATTTTTTTTTTGATAATC
664157_30677280_HHV-7_JI_U43400.1_144861bp_1_815	TTCACATTTAACAGTTTCGATAAAATTGAAGTGAGTAGTTTGATTTTTTTTTTGATAATCTCCGAGTGACTGCATTGATTTGCTCTGTGATGTAAATATTTTAAATAGGTTAAGTGTCTG
664157_30677280_HHV-7_JI_U43400.1_144861bp_1_816	TCCGAGTGACTGCATTGATTTGCTCTGTGATGTAAATATTTTAAATAGGTTAAGTGTCTGTATCCAACATTAATAAAATGTAGTCGGGCATCTAATGAAGGCAGCATTATATCGTACAGT
664157_30677280_HHV-7_JI_U43400.1_144861bp_1_817	TATCCAACATTAATAAAATGTAGTCGGGCATCTAATGAAGGCAGCATTATATCGTACAGTTTTGGTAACATAGGGAGAAAAGCTATTTCAATTTCAACTCGGGAAGATAATTGTTCATAA

664157_30677280_HHV-7_JI_U43400.1_144861bp_1_818	TTTGGTAACATAGGGAGAAAAGCTATTTCAATTTCAACTCGGGAAGATAATTGTTCATAAAGAATTTCTATATCTGTTTTGGCATTGTCGTTAGACATGGCTTATAAGGGATGGAACTCC
664157_30677280_HHV-7_JI_U43400.1_144861bp_1_819	AGAATTTCTATATCTGTTTTGGCATTGTCGTTAGACATGGCTTATAAGGGATGGAACTCCGATTCCTTTTCTATGAACTCAGAACTTTTTAATGAAATTCTTTTATATGCACACTTAGAT
664157_30677280_HHV-7_JI_U43400.1_144861bp_1_820	GATTCCTTTTCTATGAACTCAGAACTTTTTAATGAAATTCTTTTATATGCACACTTAGATTCAAGTGGAATAGATTCTGATGATCTTAACACAAATCCCAATACACTTGAAAATGAAATT
664157_30677280_HHV-7_JI_U43400.1_144861bp_1_821	TCAAGTGGAATAGATTCTGATGATCTTAACACAAATCCCAATACACTTGAAAATGAAATTAACTCCGTTGAAAAAACTTTAAATATTGAAGAACTGAAAAAAATTACAACTGCTTTAAAT
664157_30677280_HHV-7_JI_U43400.1_144861bp_1_822	AACTCCGTTGAAAAAACTTTAAATATTGAAGAACTGAAAAAAATTACAACTGCTTTAAATATTGACAACCGATGCAATATATGTTCAATTATAAACATCTGTTTACGACATGAAACCGAT
664157_30677280_HHV-7_JI_U43400.1_144861bp_1_823	ATTGACAACCGATGCAATATATGTTCAATTATAAACATCTGTTTACGACATGAAACCGATAAAATGTGGATCTATGACTATGCTCTTTTGTGTTACAAATGTAATGCTGCACCTAGAACT
664157_30677280_HHV-7_JI_U43400.1_144861bp_1_824	AAAATGTGGATCTATGACTATGCTCTTTTGTGTTACAAATGTAATGCTGCACCTAGAACTCCCTTGGCTGTCGTAATAATCGCCACCGAATTTATGCAGTTGATTCAAAAGCATTTCTTA
664157_30677280_HHV-7_JI_U43400.1_144861bp_1_825	CCCTTGGCTGTCGTAATAATCGCCACCGAATTTATGCAGTTGATTCAAAAGCATTTCTTAAACATAAATTTCGATGGGTTATTTTTAAACAACATTTTGTCAATACTCGATTTTCATGTA
664157_30677280_HHV-7_JI_U43400.1_144861bp_1_826	AACATAAATTTCGATGGGTTATTTTTAAACAACATTTTGTCAATACTCGATTTTCATGTACACTTTTTCATAAACAGGTGTTTCTCAAACACCAACGATGATCTATTACATAATGAAAAC
664157_30677280_HHV-7_JI_U43400.1_144861bp_1_827	CACTTTTTCATAAACAGGTGTTTCTCAAACACCAACGATGATCTATTACATAATGAAAACATAACCTTATATCACATGGCCATATTAAAATCACTTCTATTGGAAGACGAATCTATACCA
664157_30677280_HHV-7_JI_U43400.1_144861bp_1_828	ATAACCTTATATCACATGGCCATATTAAAATCACTTCTATTGGAAGACGAATCTATACCAAATATAAGAATAAAGAAATTTAAATTAAAAGGAAAACCAACGAAAAAACAGCATGGAAAT
664157_30677280_HHV-7_JI_U43400.1_144861bp_1_829	AATATAAGAATAAAGAAATTTAAATTAAAAGGAAAACCAACGAAAAAACAGCATGGAAATGCTATCCTTGAAAAACAAACTCTTCCACTTAACACGCATTTTACACATTTAATTTTTTAT

664157_30677280_HHV-7_JI_U43400.1_144861bp_1_830	GCTATCCTTGAAAAACAAACTCTTCCACTTAACACGCATTTTACACATTTAATTTTTTATATGTGGGCTGGGACAAACATATTCGATCGCATTTCACTAACTGATCTAGCCATCAAGAAA
664157_30677280_HHV-7_JI_U43400.1_144861bp_1_831	ATGTGGGCTGGGACAAACATATTCGATCGCATTTCACTAACTGATCTAGCCATCAAGAAACGCCAAATTTTAAAAGCCATTTACTCTACTAAAAATGAGCTCAATTGTTCTGCGGGACCA
664157_30677280_HHV-7_JI_U43400.1_144861bp_1_832	CGCCAAATTTTAAAAGCCATTTACTCTACTAAAAATGAGCTCAATTGTTCTGCGGGACCAATCCTACTATCTCAAATACCGATCTCCATCACTAAGAACGCCACCAGTAGCGTATGCTTA
664157_30677280_HHV-7_JI_U43400.1_144861bp_1_833	ATCCTACTATCTCAAATACCGATCTCCATCACTAAGAACGCCACCAGTAGCGTATGCTTATTATGTGAACTAATGACATCTTCTCAGAAAAATTTCGACTTGCTACAGTTCATCTACACC
664157_30677280_HHV-7_JI_U43400.1_144861bp_1_834	TTATGTGAACTAATGACATCTTCTCAGAAAAATTTCGACTTGCTACAGTTCATCTACACCAGTGTCATTAATTACTGTCAAAATAATTTGAAGATGATTGACAGAATTCAATTCGTACTC
664157_30677280_HHV-7_JI_U43400.1_144861bp_1_835	AGTGTCATTAATTACTGTCAAAATAATTTGAAGATGATTGACAGAATTCAATTCGTACTCGCAAATCTTTTAGATTTAGCTAGAATATATACTAACGTTAAAACCACATCAGATTGCTCA
664157_30677280_HHV-7_JI_U43400.1_144861bp_1_836	GCAAATCTTTTAGATTTAGCTAGAATATATACTAACGTTAAAACCACATCAGATTGCTCAAAAAATTGTATTAGCCAATGAACAAGAATTTTCAAACTCTGATTTTGTAATTGATTGTCAT
664157_30677280_HHV-7_JI_U43400.1_144861bp_1_837	AAAATTGTATTAGCCAATGAACAAGAATTTTCAAACTCTGATTTTGTAATTGATTGTCATAGTTTTTTAATTCTAAAGCAGGTTGGACCTGTGGGATTATACAAACATTTCTTTTGTGAT
664157_30677280_HHV-7_JI_U43400.1_144861bp_1_838	AGTTTTTTAATTCTAAAGCAGGTTGGACCTGTGGGATTATACAAACATTTCTTTTGTGATCCACTGTGCATAGCAAACATTAAGACAATAAAACCCCATATTTTATTCTATACAACAGAA
664157_30677280_HHV-7_JI_U43400.1_144861bp_1_839	CCACTGTGCATAGCAAACATTAAGACAATAAAACCCCATATTTTATTCTATACAACAGAAAGCTGTATACTACAAGACTTCAAAGTTGCAATTTGTTATCAGAATGAGTATTTAAACAGT
664157_30677280_HHV-7_JI_U43400.1_144861bp_1_840	AGCTGTATACTACAAGACTTCAAAGTTGCAATTTGTTATCAGAATGAGTATTTAAACAGTGTTGAAAAACATGTTTGGTTAGCCATTCATTTTTTTAAAGCATTTCAGGTTTCAAAATTA
664157_30677280_HHV-7_JI_U43400.1_144861bp_1_841	GTTGAAAAACATGTTTGGTTAGCCATTCATTTTTTTAAAGCATTTCAGGTTTCAAAATTAAACCACAAAAATAAAACGCTTATATCTGATTTCTTAAAGGATTTCACACAGCTCTTAGCA

664157_30677280_HHV-7_JI_U43400.1_144861bp_1_842	AACCACAAAAATAAAACGCTTATATCTGATTTCTTAAAGGATTTCACACAGCTCTTAGCAGATCAAAACTTTGAAATTGTCGATCCTACATTTACCATTCATTATTACGTTTAGCATGGC
664157_30677280_HHV-7_JI_U43400.1_144861bp_1_843	GATCAAAACTTTGAAATTGTCGATCCTACATTTACCATTCATTATTACGTTTAGCATGGCTATTCAAAGTACACGTAGACTGAGACGAGCATCTAGCTTGTTAAAGAAAAGCAAACCTTA
664157_30677280_HHV-7_JI_U43400.1_144861bp_1_844	TATTCAAAGTACACGTAGACTGAGACGAGCATCTAGCTTGTTAAAGAAAAGCAAACCTTACAACAAAGAAAAAACTAACTTATCTTTATCTTTGTCACTTAAAGAACTCCATTCGGTTTT
664157_30677280_HHV-7_JI_U43400.1_144861bp_1_845	CAACAAAGAAAAAACTAACTTATCTTTATCTTTGTCACTTAAAGAACTCCATTCGGTTTTCAAATTATTTCCAGAGTATGAATTGAAATTTCTAAATATGATGAAACTTCCAATAACCGG
664157_30677280_HHV-7_JI_U43400.1_144861bp_1_846	CAAATTATTTCCAGAGTATGAATTGAAATTTCTAAATATGATGAAACTTCCAATAACCGGTAAAGAACCTATCAAAATTCCATTCGATCTAAGTCTACATCATCAACATACGTGCTTAGA
664157_30677280_HHV-7_JI_U43400.1_144861bp_1_847	TAAAGAACCTATCAAAATTCCATTCGATCTAAGTCTACATCATCAACATACGTGCTTAGACTTATCACCATATGCCAATGAACAAGTTTCAAAAAGTGCATGTGTTAATTGTGGTACAAC
664157_30677280_HHV-7_JI_U43400.1_144861bp_1_848	CTTATCACCATATGCCAATGAACAAGTTTCAAAAAGTGCATGTGTTAATTGTGGTACAACAAACATTCCAACAGCTTCAGATGCTATGGTGGCATATATGAATCAAATTTCAAACGTAAT
664157_30677280_HHV-7_JI_U43400.1_144861bp_1_849	AAACATTCCAACAGCTTCAGATGCTATGGTGGCATATATGAATCAAATTTCAAACGTAATGCAAAATAGATTATATTACTACGGTTTTCAGAAAAAGGTTGAACTGATTCGTATGTCAGC
664157_30677280_HHV-7_JI_U43400.1_144861bp_1_850	GCAAAATAGATTATATTACTACGGTTTTCAGAAAAAGGTTGAACTGATTCGTATGTCAGCCAAGCAACCAACGCTTTTTCAAATTTTTTATATTCTTTCAAGCATAGCAAGTAATTTTTT
664157_30677280_HHV-7_JI_U43400.1_144861bp_1_851	CAAGCAACCAACGCTTTTTCAAATTTTTTATATTCTTTCAAGCATAGCAAGTAATTTTTTACCAATCATGTTTGAAAATAACGAGAAATTAAATATGTATGTTGTTTTCCAAACAAGAAC
664157_30677280_HHV-7_JI_U43400.1_144861bp_1_852	ACCAATCATGTTTGAAAATAACGAGAAATTAAATATGTATGTTGTTTTCCAAACAAGAACTCTTCATATTCCTTGCGAATGTATCAATCAGATTATGACAGTCTCTTCCGGATACACTGT
664157_30677280_HHV-7_JI_U43400.1_144861bp_1_853	TCTTCATATTCCTTGCGAATGTATCAATCAGATTATGACAGTCTCTTCCGGATACACTGTACTTTTAGACATCTTACACGACAGCATTGTACTACATGTTCTTTGTAAAACTATTGAGAC

664157_30677280_HHV-7_JI_U43400.1_144861bp_1_854	ACTTTTAGACATCTTACACGACAGCATTGTACTACATGTTCTTTGTAAAACTATTGAGACTAGCAATATTCAAATTGATATTAATGTTCTTCAGCGAAAAATTGAGGAAATGGACGTACC
664157_30677280_HHV-7_JI_U43400.1_144861bp_1_855	TAGCAATATTCAAATTGATATTAATGTTCTTCAGCGAAAAATTGAGGAAATGGACGTACCCGATGAAATAGGTGACAAGTTCGAAAAGCTGAAACACATTCTACCGTTTATTTAAAACAG
664157_30677280_HHV-7_JI_U43400.1_144861bp_1_856	CGATGAAATAGGTGACAAGTTCGAAAAGCTGAAACACATTCTACCGTTTATTTAAAACAGATCACAGAACATCTGTCAGGTCACAAAAGCTTTGATCTACATATACTTTTAAAGGTAGGA
664157_30677280_HHV-7_JI_U43400.1_144861bp_1_857	ATCACAGAACATCTGTCAGGTCACAAAAGCTTTGATCTACATATACTTTTAAAGGTAGGATTGAAAGTAAAAACTTTTCTTTCTTAATGCCCGTTTTCGGAAATATTGGAGAGATAGCAT
664157_30677280_HHV-7_JI_U43400.1_144861bp_1_858	TTGAAAGTAAAAACTTTTCTTTCTTAATGCCCGTTTTCGGAAATATTGGAGAGATAGCATTTGTTACAGCTTTCATTATTTGTTCAAAATATTTATCTGCATTGATTTTTAAGTTGTTCT
664157_30677280_HHV-7_JI_U43400.1_144861bp_1_859	TTGTTACAGCTTTCATTATTTGTTCAAAATATTTATCTGCATTGATTTTTAAGTTGTTCTCAATAGCATATGCTGGATCTTCTGCTAACTCATAATTATGAAATGTTTTTTTTACGGTTT
664157_30677280_HHV-7_JI_U43400.1_144861bp_1_860	CAATAGCATATGCTGGATCTTCTGCTAACTCATAATTATGAAATGTTTTTTTTACGGTTTCTGTGGGTGCAATTAAAATGTACATAACTCTATCTCCTATGTTTGGCAACTCTTCCTTTC
664157_30677280_HHV-7_JI_U43400.1_144861bp_1_861	CTGTGGGTGCAATTAAAATGTACATAACTCTATCTCCTATGTTTGGCAACTCTTCCTTTCTTTGAGCTAACCTTTTAACAACAGCCAAATGTGGTAAATTTGCTTGCTTGTAGGCAGAAA
664157_30677280_HHV-7_JI_U43400.1_144861bp_1_862	TTTGAGCTAACCTTTTAACAACAGCCAAATGTGGTAAATTTGCTTGCTTGTAGGCAGAAATGTCTTTTGATAAGACTGAAGACAAAACCAAACTTTTAATGTTAACCCTGTTTAAGAAAA
664157_30677280_HHV-7_JI_U43400.1_144861bp_1_863	TGTCTTTTGATAAGACTGAAGACAAAACCAAACTTTTAATGTTAACCCTGTTTAAGAAAAGTGCGTCTCGTGCGTCACACAACTTTTTAATTATTTTTTGGATACCATCGGGAACTCCAT
664157_30677280_HHV-7_JI_U43400.1_144861bp_1_864	GTGCGTCTCGTGCGTCACACAACTTTTTAATTATTTTTTGGATACCATCGGGAACTCCATTTTCATAGATTTCTTGTATTGTCATATTAGAGAGCTTTTCAGCAGACTTTTGTACATCGA
664157_30677280_HHV-7_JI_U43400.1_144861bp_1_865	TTTCATAGATTTCTTGTATTGTCATATTAGAGAGCTTTTCAGCAGACTTTTGTACATCGACATCCCAAAAGAGAAGATCGATGATGTCTTTAACTACCACTTTAACAAAATCGCAAGAAG

664157_30677280_HHV-7_JI_U43400.1_144861bp_1_866	CATCCCAAAAGAGAAGATCGATGATGTCTTTAACTACCACTTTAACAAAATCGCAAGAAGTCTTGCGAACTAACTCCACACCTTTAAAAACAAGCGTTGCATCATCTAACTTTCCAATGT
664157_30677280_HHV-7_JI_U43400.1_144861bp_1_867	TCTTGCGAACTAACTCCACACCTTTAAAAACAAGCGTTGCATCATCTAACTTTCCAATGTAACGTTTCTTGCATATTAAAATTAATGGAAATAGGATCTTTTCAAATTCTAATTTGATAG
664157_30677280_HHV-7_JI_U43400.1_144861bp_1_868	AACGTTTCTTGCATATTAAAATTAATGGAAATAGGATCTTTTCAAATTCTAATTTGATAGGATGTTTAAATAACGTGTTTGTTATGTGGCTTGCAATAGAAGGAGCTATTCTTTTCAAAG
664157_30677280_HHV-7_JI_U43400.1_144861bp_1_869	GATGTTTAAATAACGTGTTTGTTATGTGGCTTGCAATAGAAGGAGCTATTCTTTTCAAAGCTTTGGGACAAACATTTTTGAAAGTAACAAAAAGACTGTCAGTATCACCATAAATCACCT
664157_30677280_HHV-7_JI_U43400.1_144861bp_1_870	CTTTGGGACAAACATTTTTGAAAGTAACAAAAAGACTGTCAGTATCACCATAAATCACCTCTATTCCAAATGTCCCTGAAAAATCACCGCGTGTCAATCCAAATTTCTCAATGAAAAAAG
664157_30677280_HHV-7_JI_U43400.1_144861bp_1_871	CTATTCCAAATGTCCCTGAAAAATCACCGCGTGTCAATCCAAATTTCTCAATGAAAAAAGTGTCCGAATACATGGCGCTGTCAACGTAATCAACAGTTTTACAAAGCATTTCACGCCCAA
664157_30677280_HHV-7_JI_U43400.1_144861bp_1_872	TGTCCGAATACATGGCGCTGTCAACGTAATCAACAGTTTTACAAAGCATTTCACGCCCAAGACATGTGACAGAAGCTGCTATTGCCACACATGGAAGTAAGCTATGTGTTGCCCCGGTGA
664157_30677280_HHV-7_JI_U43400.1_144861bp_1_873	GACATGTGACAGAAGCTGCTATTGCCACACATGGAAGTAAGCTATGTGTTGCCCCGGTGACTCCGTAGACTGAATTACAAGTAGTTTTTAATGCAAGCTGCTTCTTATCTAAAAGCATTT
664157_30677280_HHV-7_JI_U43400.1_144861bp_1_874	CTCCGTAGACTGAATTACAAGTAGTTTTTAATGCAAGCTGCTTCTTATCTAAAAGCATTTCCATCACGGGGTTGTTACACATTTTCATCTGCATTTTTACTTCTTTTCTTTTATCCAGCC
664157_30677280_HHV-7_JI_U43400.1_144861bp_1_875	CCATCACGGGGTTGTTACACATTTTCATCTGCATTTTTACTTCTTTTCTTTTATCCAGCCATTTTTTCAATAAACTCGCTAAAATTGATTCCCTGACTGTCTTTTTAACAAACCTGTGCG
664157_30677280_HHV-7_JI_U43400.1_144861bp_1_876	ATTTTTTCAATAAACTCGCTAAAATTGATTCCCTGACTGTCTTTTTAACAAACCTGTGCGTTACAGGTCCAACATGCACAGTGAGAATGTCATCTGCATGTAAACCAATTACTGCATTTT
664157_30677280_HHV-7_JI_U43400.1_144861bp_1_877	TTACAGGTCCAACATGCACAGTGAGAATGTCATCTGCATGTAAACCAATTACTGCATTTTCATCAACAACCAGGGTACTATAACACAGATTATGAGCCATCATAATACTTGGATACAAAC

664157_30677280_HHV-7_JI_U43400.1_144861bp_1_878	CATCAACAACCAGGGTACTATAACACAGATTATGAGCCATCATAATACTTGGATACAAACTTTGGAAATCAAAAACTACTGTTGGAGTAGCATAATAACCTATTTTCGGTTCTAAAACTG
664157_30677280_HHV-7_JI_U43400.1_144861bp_1_879	TTTGGAAATCAAAAACTACTGTTGGAGTAGCATAATAACCTATTTTCGGTTCTAAAACTGTAGCACCTTTATAGCCCACATTTTCTTTGCCTTTATTAAAGTTAGTGTTCATACTTGGTA
664157_30677280_HHV-7_JI_U43400.1_144861bp_1_880	TAGCACCTTTATAGCCCACATTTTCTTTGCCTTTATTAAAGTTAGTGTTCATACTTGGTAAAATCATATTTAAATTTTTAGCTTCGTGTAAAATGCATGGGAAAATTTTTTTTTGTTGTC
664157_30677280_HHV-7_JI_U43400.1_144861bp_1_881	AAATCATATTTAAATTTTTAGCTTCGTGTAAAATGCATGGGAAAATTTTTTTTTGTTGTCCCTCAAATACGGCGCAACGAATAGTAATATAAGCGAGACTTGCAACCTCAGCCATTTCAT
664157_30677280_HHV-7_JI_U43400.1_144861bp_1_882	CCTCAAATACGGCGCAACGAATAGTAATATAAGCGAGACTTGCAACCTCAGCCATTTCATAGTGATAATTAATCTTTGTAAAAAGCTGAACAACCAATAGAGAATCTTGTATACAATATC
664157_30677280_HHV-7_JI_U43400.1_144861bp_1_883	AGTGATAATTAATCTTTGTAAAAAGCTGAACAACCAATAGAGAATCTTGTATACAATATCTGCCAACCACAGCTCTACCTTTGGAACCATCAATAAATTTTTTAGGAATTTCCTTGTAAG
664157_30677280_HHV-7_JI_U43400.1_144861bp_1_884	TGCCAACCACAGCTCTACCTTTGGAACCATCAATAAATTTTTTAGGAATTTCCTTGTAAGAGAGATTTTCTTTTTCCTGATTTAAACAAAGTTTGGCTATAGTGTCTAATTTATAATTCT
664157_30677280_HHV-7_JI_U43400.1_144861bp_1_885	AGAGATTTTCTTTTTCCTGATTTAAACAAAGTTTGGCTATAGTGTCTAATTTATAATTCTGAGCTGAGATTTTAGATGAATACACATTATACATGTCGAAGCATAAAATCCCAGACATGT
664157_30677280_HHV-7_JI_U43400.1_144861bp_1_886	GAGCTGAGATTTTAGATGAATACACATTATACATGTCGAAGCATAAAATCCCAGACATGTTAACTTTAGTTAAAGAATTCAAAAACTTCTTATGCTGTTCGTAGGGAACGATGACAGAAA
664157_30677280_HHV-7_JI_U43400.1_144861bp_1_887	TAACTTTAGTTAAAGAATTCAAAAACTTCTTATGCTGTTCGTAGGGAACGATGACAGAAAACTTTCCACGTTTTAGTTTCGAAAAACCCCCTATCTCGAAATTATATATTTTTTCCATTC
664157_30677280_HHV-7_JI_U43400.1_144861bp_1_888	ACTTTCCACGTTTTAGTTTCGAAAAACCCCCTATCTCGAAATTATATATTTTTTCCATTCTCGTACATAAATACTTTAAATCGAAATTGATGATGTTGTATCCAGTAAGGATTTCTGGTG
664157_30677280_HHV-7_JI_U43400.1_144861bp_1_889	TCGTACATAAATACTTTAAATCGAAATTGATGATGTTGTATCCAGTAAGGATTTCTGGTGATTTACATTTTAAAAAAAGGAAAAAAGCATATAGAAGTTCAAATTCTGATGCAAACTCAT

664157_30677280_HHV-7_JI_U43400.1_144861bp_1_890	ATTTACATTTTAAAAAAAGGAAAAAAGCATATAGAAGTTCAAATTCTGATGCAAACTCATAGATGAATACCCCTTCGATTTGCTCGCAAGTGCCCAAAGTAAACAAATGTTTACTTTGGT
664157_30677280_HHV-7_JI_U43400.1_144861bp_1_891	AGATGAATACCCCTTCGATTTGCTCGCAAGTGCCCAAAGTAAACAAATGTTTACTTTGGTAATTTCCTTCAGAATCAAAATCGATGACCGAAATTAATGATGATGTCACCATTTGCTCAG
664157_30677280_HHV-7_JI_U43400.1_144861bp_1_892	AATTTCCTTCAGAATCAAAATCGATGACCGAAATTAATGATGATGTCACCATTTGCTCAGCATCTGGAAAGTTTCCATTTTGGCTTAGACATTCAATATCAAAAGAACAACAATCATATA
664157_30677280_HHV-7_JI_U43400.1_144861bp_1_893	CATCTGGAAAGTTTCCATTTTGGCTTAGACATTCAATATCAAAAGAACAACAATCATATAATGGCCAAGAATCTTCCTTTAACAAAAATAAATCTGACACATGACAATTTAGTTCAATTT
664157_30677280_HHV-7_JI_U43400.1_144861bp_1_894	ATGGCCAAGAATCTTCCTTTAACAAAAATAAATCTGACACATGACAATTTAGTTCAATTTCAACATTACTGCTTTTTGCAAATTCTTGAATAGATAGATAATTAATTTGGTACCAACCAA
664157_30677280_HHV-7_JI_U43400.1_144861bp_1_895	CAACATTACTGCTTTTTGCAAATTCTTGAATAGATAGATAATTAATTTGGTACCAACCAAAGCTTTTTAAGTTGTTATCAATGAAGAAACGATTGAGAATCTCAACTTCTGCTTCATAAA
664157_30677280_HHV-7_JI_U43400.1_144861bp_1_896	AGCTTTTTAAGTTGTTATCAATGAAGAAACGATTGAGAATCTCAACTTCTGCTTCATAAACAGAAACGCCCTCATTTAGTAAAATTTTTCCAATTCGATTACTGATATAAAAGTTTGAAA
664157_30677280_HHV-7_JI_U43400.1_144861bp_1_897	CAGAAACGCCCTCATTTAGTAAAATTTTTCCAATTCGATTACTGATATAAAAGTTTGAAAATGATAATTTAAATAAATTTTTGATAGGTTCCGTGTTATAACCGTAAAAATTGTATTTAG
664157_30677280_HHV-7_JI_U43400.1_144861bp_1_898	ATGATAATTTAAATAAATTTTTGATAGGTTCCGTGTTATAACCGTAAAAATTGTATTTAGTTACAGATTCAATAGAAAATGAGCATGACATTTTTATTTCAGAACTATTCAGAAGTGAAC
664157_30677280_HHV-7_JI_U43400.1_144861bp_1_899	TTACAGATTCAATAGAAAATGAGCATGACATTTTTATTTCAGAACTATTCAGAAGTGAACAGATTCTAGAATTCAGCTCTTTTTTACATTGATATTCACAGTAGAAATAACTATTTTGTC
664157_30677280_HHV-7_JI_U43400.1_144861bp_1_900	AGATTCTAGAATTCAGCTCTTTTTTACATTGATATTCACAGTAGAAATAACTATTTTGTCCAAATACATTGATACAAACTTTTTTACCACATTCTGTTTTTCCAAAAAGTTTTATGACAT
664157_30677280_HHV-7_JI_U43400.1_144861bp_1_901	CAAATACATTGATACAAACTTTTTTACCACATTCTGTTTTTCCAAAAAGTTTTATGACATTACCAGATGGAATAACGAAATGTCTATATTGGAAAGGGATGTTTTCTATAGAATCAGTAA

664157_30677280_HHV-7_JI_U43400.1_144861bp_1_902	TACCAGATGGAATAACGAAATGTCTATATTGGAAAGGGATGTTTTCTATAGAATCAGTAAATAATAAAGATTCGGATGCATCATAGATATGAAATTTTAAAGGCGCACGCAATTCTTTTT
664157_30677280_HHV-7_JI_U43400.1_144861bp_1_903	ATAATAAAGATTCGGATGCATCATAGATATGAAATTTTAAAGGCGCACGCAATTCTTTTTCGGCTACTTGAGAAAGCGATGGCCATGTCATATCATTTTTGAGAATGTATTGCTTATCTT
664157_30677280_HHV-7_JI_U43400.1_144861bp_1_904	CGGCTACTTGAGAAAGCGATGGCCATGTCATATCATTTTTGAGAATGTATTGCTTATCTTGATAGAACATTCTCGGTTCCGAGTCACAAAGAGTCTTCATTAAGCCGGGTGCTCCATCGT
664157_30677280_HHV-7_JI_U43400.1_144861bp_1_905	GATAGAACATTCTCGGTTCCGAGTCACAAAGAGTCTTCATTAAGCCGGGTGCTCCATCGTGCATTATACCGCGCGGAAAAATCCGGAGAAAAGTAGATTTTGTTTTTTTCTTAGTTCGTA
664157_30677280_HHV-7_JI_U43400.1_144861bp_1_906	GCATTATACCGCGCGGAAAAATCCGGAGAAAAGTAGATTTTGTTTTTTTCTTAGTTCGTACATTCTCTAGATACGGATTAAAGAAGGACACCAGATCCATCACAGTTCTTCTGTTGAAAG
664157_30677280_HHV-7_JI_U43400.1_144861bp_1_907	CATTCTCTAGATACGGATTAAAGAAGGACACCAGATCCATCACAGTTCTTCTGTTGAAAGTTTCTGATAGCCCCTGTATTGAATCCTTTCAAGTAGGCTTGGTCTCTGTGATTTTTTTGC
664157_30677280_HHV-7_JI_U43400.1_144861bp_1_908	TTTCTGATAGCCCCTGTATTGAATCCTTTCAAGTAGGCTTGGTCTCTGTGATTTTTTTGCCTCAGCGATTTCCGCTTTTTTATATGACTCATCTAATAATTTAATAGCTTTCAACATTTT
664157_30677280_HHV-7_JI_U43400.1_144861bp_1_909	CTCAGCGATTTCCGCTTTTTTATATGACTCATCTAATAATTTAATAGCTTTCAACATTTTTAAAGCTTCCACTTGTGAATATTCATTAGTGTGTGTTATGTCATCACCTTGCGGTTCTGT
664157_30677280_HHV-7_JI_U43400.1_144861bp_1_910	TAAAGCTTCCACTTGTGAATATTCATTAGTGTGTGTTATGTCATCACCTTGCGGTTCTGTGTCTTTGTTAGTTTTTAAATAAGGCGGAGATGAATCTAAAGGTTCTTTCACTTGCGCTTG
664157_30677280_HHV-7_JI_U43400.1_144861bp_1_911	GTCTTTGTTAGTTTTTAAATAAGGCGGAGATGAATCTAAAGGTTCTTTCACTTGCGCTTGAACCGATTGCGTTGCTTGAAGTACCGAATTTGTTGATTTTGATGTATAAGGAAATAAAAT
664157_30677280_HHV-7_JI_U43400.1_144861bp_1_912	AACCGATTGCGTTGCTTGAAGTACCGAATTTGTTGATTTTGATGTATAAGGAAATAAAATATCAATCGGTGTTTGTGCTAATCGTTTATGTCTTAAAAATAAGAAAATAACTAAGCCAAT
664157_30677280_HHV-7_JI_U43400.1_144861bp_1_913	ATCAATCGGTGTTTGTGCTAATCGTTTATGTCTTAAAAATAAGAAAATAACTAAGCCAATCACTCCCAAAGTTAGTAAGATAGTTAGTGCTCCACCAAATGGATTTTTAAGAAAAGAGAA

664157_30677280_HHV-7_JI_U43400.1_144861bp_1_914	CACTCCCAAAGTTAGTAAGATAGTTAGTGCTCCACCAAATGGATTTTTAAGAAAAGAGAAAACTCCATTCACCACATCTCCCAAAGCACCAGCCGTTACACCCAGCACAGCCCCCAAACC
664157_30677280_HHV-7_JI_U43400.1_144861bp_1_915	AACTCCATTCACCACATCTCCCAAAGCACCAGCCGTTACACCCAGCACAGCCCCCAAACCAAGACCAAGAGCGCCAAGACCTTTGAAAAATGTGTCAATCCCTCCTATATATGTTGGAGT
664157_30677280_HHV-7_JI_U43400.1_144861bp_1_916	AAGACCAAGAGCGCCAAGACCTTTGAAAAATGTGTCAATCCCTCCTATATATGTTGGAGTAACAGTTGCAATTTTTGTTTCTATATGGTGTAGCGCACTTTTGTAAGAATTGTATTCTCT
664157_30677280_HHV-7_JI_U43400.1_144861bp_1_917	AACAGTTGCAATTTTTGTTTCTATATGGTGTAGCGCACTTTTGTAAGAATTGTATTCTCTAAGAATGGTTTCCAAATCAAAAACGTTCGCTTTGCTCAGTTCATCTTTTGTATACAATTC
664157_30677280_HHV-7_JI_U43400.1_144861bp_1_918	AAGAATGGTTTCCAAATCAAAAACGTTCGCTTTGCTCAGTTCATCTTTTGTATACAATTCCAATAATGTAAAATCTGCATTTTCTAAAGGATCTATGTTTAAGTCAACAAACGCATCTAA
664157_30677280_HHV-7_JI_U43400.1_144861bp_1_919	CAATAATGTAAAATCTGCATTTTCTAAAGGATCTATGTTTAAGTCAACAAACGCATCTAAAGCTTCTATTTCTGTTATCAAACTAGAATTAACGTACGTATAGTCTTTAAAAATATGAGC
664157_30677280_HHV-7_JI_U43400.1_144861bp_1_920	AGCTTCTATTTCTGTTATCAAACTAGAATTAACGTACGTATAGTCTTTAAAAATATGAGCGAATTTACCAGACAAAAAAATTTTCGTGTTAGATTGTTCACATTCTTCTGTTCTGTGATT
664157_30677280_HHV-7_JI_U43400.1_144861bp_1_921	GAATTTACCAGACAAAAAAATTTTCGTGTTAGATTGTTCACATTCTTCTGTTCTGTGATTTCCCAGTAATATCTCATTGTCTAAACCAAGTTGTCCTAAATAAGTTTCCTTCGAAGAATT
664157_30677280_HHV-7_JI_U43400.1_144861bp_1_922	TCCCAGTAATATCTCATTGTCTAAACCAAGTTGTCCTAAATAAGTTTCCTTCGAAGAATTTGCAAATGAATATGTTAACAATGGCCGAGAATAACATCTTAGAGCATCGTAACTTGAATC
664157_30677280_HHV-7_JI_U43400.1_144861bp_1_923	TGCAAATGAATATGTTAACAATGGCCGAGAATAACATCTTAGAGCATCGTAACTTGAATCTTTTGTCAATCTCATACTTTTATGTAGCTGAACAGAAGTCTGGTTGACTTCAATGCACTT
664157_30677280_HHV-7_JI_U43400.1_144861bp_1_924	TTTTGTCAATCTCATACTTTTATGTAGCTGAACAGAAGTCTGGTTGACTTCAATGCACTTTGAGACTGCTAATACATCACCAATTAATTTAGCGGACATGGGTTTACCGTACACTGCTGA
664157_30677280_HHV-7_JI_U43400.1_144861bp_1_925	TGAGACTGCTAATACATCACCAATTAATTTAGCGGACATGGGTTTACCGTACACTGCTGAAATGATTCCAGAAGGACTTATTTTACTAAGCTCGTGCAACACTGTGATAGTGCGTTTTTG

664157_30677280_HHV-7_JI_U43400.1_144861bp_1_926	AATGATTCCAGAAGGACTTATTTACTAAGCTCGTGCAACACTGTGATAGTGCGTTTTTGATTTAAACACCAGGCTTCAGCAAGCTTACCTAGTGCTGTGTTAATGTAATCTTTCAGAGT
664157_30677280_HHV-7_JI_U43400.1_144861bp_1_927	ATTTAAACACCAGGCTTCAGCAAGCTTACCTAGTGCTGTGTTAATGTAATCTTTCAGAGTGTCGTATAGGTATTGTAGTTGAACATATACAATGTCTTTGTTAGTCTCTAATTCCCTCTT
664157_30677280_HHV-7_JI_U43400.1_144861bp_1_928	GTCGTATAGGTATTGTAGTTGAACATATACAATGTCTTTGTTAGTCTCTAATTCCCTCTTCCTCCTTTTTCTAGAAGCATTAGAAAAATTTTCCAAAACCGTTAATTTCCGTTGTATTAA
664157_30677280_HHV-7_JI_U43400.1_144861bp_1_929	CCTCCTTTTTCTAGAAGCATTAGAAAAATTTTCCAAAACCGTTAATTTCCGTTGTATTAACGGTTGCCAAATCAGAATTAAATCTCCCTCAGTTTTAAAAATTTCATAACTACCATTTTT
664157_30677280_HHV-7_JI_U43400.1_144861bp_1_930	CGGTTGCCAAATCAGAATTAAATCTCCCTCAGTTTTAAAAATTTCATAACTACCATTTTTGTCATGCGAATTGTTGTATTGCGTTTGGTAAATCTCATCCAAAATTGAAGTATAATTGCT
664157_30677280_HHV-7_JI_U43400.1_144861bp_1_931	GTCATGCGAATTGTTGTATTGCGTTTGGTAAATCTCATCCAAAATTGAAGTATAATTGCTGTTAATACAATTATATTTTGAATCAGAAAGCGTATAATTACTTTTTCCTGTGACAAAAGA
664157_30677280_HHV-7_JI_U43400.1_144861bp_1_932	GTTAATACAATTATATTTTGAATCAGAAAGCGTATAATTACTTTTTCCTGTGACAAAAGAAGCAGTTAGTTCCTGCGCAATAAAGTGAAAACTGTTAGCATTTTCTGCCCGTAAAGCGTG
664157_30677280_HHV-7_JI_U43400.1_144861bp_1_933	AGCAGTTAGTTCCTGCGCAATAAAGTGAAAACTGTTAGCATTTTCTGCCCGTAAAGCGTGTGGGATTGTCATCCAATGCTTTAACAAACAAATGGAAGACTCTTCATTCTGCACTTCCCA
664157_30677280_HHV-7_JI_U43400.1_144861bp_1_934	TGGGATTGTCATCCAATGCTTTAACAAACAAATGGAAGACTCTTCATTCTGCACTTCCCATGAAAAAATAGTATTTCCCTTTTCCAGAAAAGCCATTTTAGTTAACGTCACAAAATTTCC
664157_30677280_HHV-7_JI_U43400.1_144861bp_1_935	TGAAAAAATAGTATTTCCCTTTTCCAGAAAAGCCATTTTAGTTAACGTCACAAAATTTCCTTTTGATCCATCATCAAACGTTTTCAGCATAGTATAATTATTTCTAAACAAAATTTTTTC
664157_30677280_HHV-7_JI_U43400.1_144861bp_1_936	TTTTGATCCATCATCAAACGTTTTCAGCATAGTATAATTATTTCTAAACAAAATTTTTTCCGTTGGTTCATTAAATGTTTTAGAATTAATACCATTATAAAACGGTGACCCTTCCACGGT
664157_30677280_HHV-7_JI_U43400.1_144861bp_1_937	CGTTGGTTCATTAAATGTTTTAGAATTAATACCATTATAAAACGGTGACCCTTCCACGGTTTCCCCTGTTGATAAAGCAAAAAAATCAAAAGGATATTTAGTCTTTGCAATGCAGTCTGT

664157_30677280_HHV-7_JI_U43400.1_144861bp_1_938	TTCCCCTGTTGATAAAGCAAAAAAATCAAAAGGATATTTAGTCTTTGCAATGCAGTCTGTAACTATGCAATTTATCGATGTAGATGTTGAATAAAACCAGAGGGGACCATTTCTTAAAAA
664157_30677280_HHV-7_JI_U43400.1_144861bp_1_939	AACTATGCAATTTATCGATGTAGATGTTGAATAAAACCAGAGGGGACCATTTCTTAAAAATGGTTCTTTAGTAGTTATATATCTTCTGACAGTATCAGATTTAAAATTTAACGGAATCAA
664157_30677280_HHV-7_JI_U43400.1_144861bp_1_940	TGGTTCTTTAGTAGTTATATATCTTCTGACAGTATCAGATTTAAAATTTAACGGAATCAAATCCAACGTTTTATTCACATATTCATCTTTATGATATGCAACATACTCCTCTTCCTCTGA
664157_30677280_HHV-7_JI_U43400.1_144861bp_1_941	ATCCAACGTTTTATTCACATATTCATCTTTATGATATGCAACATACTCCTCTTCCTCTGAACGTTTTACAGAGATAGAGGACAAACAACGCGCCTCGGTGTTTACCATGTGCACTTCTTC
664157_30677280_HHV-7_JI_U43400.1_144861bp_1_942	ACGTTTTACAGAGATAGAGGACAAACAACGCGCCTCGGTGTTTACCATGTGCACTTCTTCAATTGGCATTGGCAAAGTTGTAACAGTTCGATCTAAGAAATACACAGTGCCAACATCGCG
664157_30677280_HHV-7_JI_U43400.1_144861bp_1_943	AATTGGCATTGGCAAAGTTGTAACAGTTCGATCTAAGAAATACACAGTGCCAACATCGCGATATGTTGTTTGAAATGTAAGTTCTTTTTTAAAAGTTCTGACAGAAAACGTGTGTGCTTC
664157_30677280_HHV-7_JI_U43400.1_144861bp_1_944	ATATGTTGTTTGAAATGTAAGTTCTTTTTTAAAAGTTCTGACAGAAAACGTGTGTGCTTCAATCTTTGTTTTGTAAATTATCAAAATACCTTCGGTAGTTTTAATGTTAGAGCCGTAAGA
664157_30677280_HHV-7_JI_U43400.1_144861bp_1_945	AATCTTTGTTTTGTAAATTATCAAAATACCTTCGGTAGTTTTAATGTTAGAGCCGTAAGACGCGCAAGAAACCTCCCTGTCAAAACGCACTAAATCTGTCCCGGTGGCAATTGAACAAAT
664157_30677280_HHV-7_JI_U43400.1_144861bp_1_946	CGCGCAAGAAACCTCCCTGTCAAAACGCACTAAATCTGTCCCGGTGGCAATTGAACAAATTCGAAATGGTAAATGCTGATTGTGTCCAGTCATGACAAAGTCAGCTTCTGTTTGTAGAGA
664157_30677280_HHV-7_JI_U43400.1_144861bp_1_947	TCGAAATGGTAAATGCTGATTGTGTCCAGTCATGACAAAGTCAGCTTCTGTTTGTAGAGATAGCTGTAAGCTAAAAGTTATAAAAACACTCAGGAATAGAATTTTCATTTTGTCTACATA
664157_30677280_HHV-7_JI_U43400.1_144861bp_1_948	TAGCTGTAAGCTAAAAGTTATAAAAACACTCAGGAATAGAATTTTCATTTTGTCTACATAGATCTAACAGATTTGGAGCCGGTATATGGGAAACTTGCTTAGTTTGTGAAATTTGAACAG
664157_30677280_HHV-7_JI_U43400.1_144861bp_1_949	GATCTAACAGATTTGGAGCCGGTATATGGGAAACTTGCTTAGTTTGTGAAATTTGAACAGGATCGCATTGAGTCTTTAAATCGAAATACAATAAAGAGTACAAATCATTAAATTTTGTCG

664157_30677280_HHV-7_JI_U43400.1_144861bp_1_950	GATCGCATTGAGTCTTTAAATCGAAATACAATAAAGAGTACAAATCATTAAATTTTGTCGTGGTCGTTCCACGCTTGAATAGTAAAGGAGAACCACTATCAAAAGTTAAAAGTATTTTTT
664157_30677280_HHV-7_JI_U43400.1_144861bp_1_951	TGGTCGTTCCACGCTTGAATAGTAAAGGAGAACCACTATCAAAAGTTAAAAGTATTTTTTCGACAGTTTCTGTCTCTTCGCCAAACTTGACGATTGATAATTGTTTTCCAAATACATCAT
664157_30677280_HHV-7_JI_U43400.1_144861bp_1_952	CGACAGTTTCTGTCTCTTCGCCAAACTTGACGATTGATAATTGTTTTCCAAATACATCATTATATAGTGCAACAGACAAAACTAATTCCCTGATAAAATTCCACATTTGTCTTTGCATAT
664157_30677280_HHV-7_JI_U43400.1_144861bp_1_953	TATATAGTGCAACAGACAAAACTAATTCCCTGATAAAATTCCACATTTGTCTTTGCATATCGTTGATATTATGCAAACCATCAAAATTGAAAAATTTATTATATGTACACAGCATCCAAT
664157_30677280_HHV-7_JI_U43400.1_144861bp_1_954	CGTTGATATTATGCAAACCATCAAAATTGAAAAATTTATTATATGTACACAGCATCCAATTTTCCGGGTAGATCGTGCCTTCAACACATTTAGCCAGATCTTCTTTCATATGTGGTAAAA
664157_30677280_HHV-7_JI_U43400.1_144861bp_1_955	TTTCCGGGTAGATCGTGCCTTCAACACATTTAGCCAGATCTTCTTTCATATGTGGTAAAAAATCTGCAACATCGCAAGCATACGCCATGGATATGTTGCTGGACAATGGAAAAGAAGCGG
664157_30677280_HHV-7_JI_U43400.1_144861bp_1_956	AATCTGCAACATCGCAAGCATACGCCATGGATATGTTGCTGGACAATGGAAAAGAAGCGGTGTGATAATTCGACAATGGACCAGTCAACAATCGATACATTTCTTGACTTAAATTAGGTA
664157_30677280_HHV-7_JI_U43400.1_144861bp_1_957	TGTGATAATTCGACAATGGACCAGTCAACAATCGATACATTTCTTGACTTAAATTAGGTAAAAGTTCCTGAGGCAATTTTTTTGAAAGTAGATTGTTTTTAATGTATAAGTGATCATCAT
664157_30677280_HHV-7_JI_U43400.1_144861bp_1_958	AAAGTTCCTGAGGCAATTTTTTTGAAAGTAGATTGTTTTTAATGTATAAGTGATCATCATAAGTAAATGGATCAGCAGAGAAATTTTCTAGTGACTGCAGTTGTCTATTTGCAAATGCGT
664157_30677280_HHV-7_JI_U43400.1_144861bp_1_959	AAGTAAATGGATCAGCAGAGAAATTTTCTAGTGACTGCAGTTGTCTATTTGCAAATGCGTTCATCATTTTCGAAAGACTTTCGAAAATGTGATTTCCTAGGATATTAATATTCATTAACT
664157_30677280_HHV-7_JI_U43400.1_144861bp_1_960	TCATCATTTTCGAAAGACTTTCGAAAATGTGATTTCCTAGGATATTAATATTCATTAACTTATTTATTAATGCTTCTTGCTCTTTGATACACAGCATTACTTTACTATAACCCGATTCGG
664157_30677280_HHV-7_JI_U43400.1_144861bp_1_961	TATTTATTAATGCTTCTTGCTCTTTGATACACAGCATTACTTTACTATAACCCGATTCGGAAATTTTTTGGTAATAAGCTTTTTTTCTAATGTGAATCTCTTGTTGTAAATTGTGATCTT

664157_30677280_HHV-7_JI_U43400.1_144861bp_1_962	AAATTTTTTGGTAATAAGCTTTTTTTCTAATGTGAATCTCTTGTTGTAAATTGTGATCTTGTGTCATTGAATGCGTTACGTATTTTTTTGGGACAGTTTCATGAGCTGAATTTTCAAGTT
664157_30677280_HHV-7_JI_U43400.1_144861bp_1_963	GTGTCATTGAATGCGTTACGTATTTTTTTGGGACAGTTTCATGAGCTGAATTTTCAAGTTCATTTAATAAAGTTTTTATTTTAGTTTGTATTTCATCTTCCTCTGCCAGCTTTGTAAAAA
664157_30677280_HHV-7_JI_U43400.1_144861bp_1_964	CATTTAATAAAGTTTTTATTTTAGTTTGTATTTCATCTTCCTCTGCCAGCTTTGTAAAAACCGGATCTTCTTCTAAGTTTTTTATACTAACATTGGTAATAATATCAACGAGTCTGCAAG
664157_30677280_HHV-7_JI_U43400.1_144861bp_1_965	CCGGATCTTCTTCTAAGTTTTTTATACTAACATTGGTAATAATATCAACGAGTCTGCAAGGGGAAATAAATCTCGATCCGATGTATAATTTGTCATCATTTGTAACATTTTCTTCGTTAA
664157_30677280_HHV-7_JI_U43400.1_144861bp_1_966	GGGAAATAAATCTCGATCCGATGTATAATTTGTCATCATTTGTAACATTTTCTTCGTTAAAATTGAAAACATCTTGAATAGTTTCACCATATAAAAACTTACTAATTTCATTTTGGAGAT
664157_30677280_HHV-7_JI_U43400.1_144861bp_1_967	AATTGAAAACATCTTGAATAGTTTCACCATATAAAAACTTACTAATTTCATTTTGGAGATCAGCATAAAGTGAATGACAAAGATTTAGTTGATTTAGAGTTATTGAAGCTTTTTGAATAA
664157_30677280_HHV-7_JI_U43400.1_144861bp_1_968	CAGCATAAAGTGAATGACAAAGATTTAGTTGATTTAGAGTTATTGAAGCTTTTTGAATAACAGATTCTGAAATTTTAGACCAATAGGTGAATTCACTCAACGAATAAATTGTATCTGGAA
664157_30677280_HHV-7_JI_U43400.1_144861bp_1_969	CAGATTCTGAAATTTTAGACCAATAGGTGAATTCACTCAACGAATAAATTGTATCTGGAATTTTGCTAAAAAGGTCAAAGTTGATTAAAGTGTTTTCAGCTTCTTGTAAATGTGGAGGTT
664157_30677280_HHV-7_JI_U43400.1_144861bp_1_970	TTTTGCTAAAAAGGTCAAAGTTGATTAAAGTGTTTTCAGCTTCTTGTAAATGTGGAGGTTTTGAATATAGTTGCTGTTGAAAAATATTTGTGATTTTCGCCATATACTCTTTTACAAGAC
664157_30677280_HHV-7_JI_U43400.1_144861bp_1_971	TTGAATATAGTTGCTGTTGAAAAATATTTGTGATTTTCGCCATATACTCTTTTACAAGACCATATTGTTTTGTGGAAAAATCCAAATCTTTCTCAATGATGTCAACGTTTTTTTCTAAAT
664157_30677280_HHV-7_JI_U43400.1_144861bp_1_972	CATATTGTTTTGTGGAAAAATCCAAATCTTTCTCAATGATGTCAACGTTTTTTTCTAAATTGGATACCATATGTGTTTCAGTTAGATGATTACAAAATTTGCCAGCTAGTCTTTTACGAA
664157_30677280_HHV-7_JI_U43400.1_144861bp_1_973	TGGATACCATATGTGTTTCAGTTAGATGATTACAAAATTTGCCAGCTAGTCTTTTACGAATAGACTTTCCTTGATTGGGCACTAAAGATAATTCTTCATAACATTTTAAACATGTAGTAG

664157_30677280_HHV-7_JI_U43400.1_144861bp_1_974	TAGACTTTCCTTGATTGGGCACTAAAGATAATTCTTCATAACATTTTAAACATGTAGTAGTTTCAGAAAAAACTTCTGGCTTCATCACTACGCAAACGCCACACAAAATTGTTAGAAATT
664157_30677280_HHV-7_JI_U43400.1_144861bp_1_975	TTTCAGAAAAAACTTCTGGCTTCATCACTACGCAAACGCCACACAAAATTGTTAGAAATTCAATAATTTGACTGCAAGCATTAATGCCATCCAGTGGTGAAATTAAGCCAAAAATACAAT
664157_30677280_HHV-7_JI_U43400.1_144861bp_1_976	CAATAATTTGACTGCAAGCATTAATGCCATCCAGTGGTGAAATTAAGCCAAAAATACAATTCATCTTACATAAAAGTTTTTCAATATCATTGATCGTAGAAATGTCTACAGAAAAAGTCA
664157_30677280_HHV-7_JI_U43400.1_144861bp_1_977	TCATCTTACATAAAAGTTTTTCAATATCATTGATCGTAGAAATGTCTACAGAAAAAGTCAAATAATTATTAAGAGCCAAGTCCAATTTTAAGTGTTGCTTGCATTCATTGAGTTGGAATA
664157_30677280_HHV-7_JI_U43400.1_144861bp_1_978	AATAATTATTAAGAGCCAAGTCCAATTTTAAGTGTTGCTTGCATTCATTGAGTTGGAATATATTTTCAAAATATTTTTGTTTGTCATTATCTAATAGAAATTGGTGAATTGAAGCATCGA
664157_30677280_HHV-7_JI_U43400.1_144861bp_1_979	TATTTTCAAAATATTTTTGTTTGTCATTATCTAATAGAAATTGGTGAATTGAAGCATCGAGTAATAAGACTTGATTATACATAGCTTTTAATAAAATCTGTAAATAAATGGTTACAGGAG
664157_30677280_HHV-7_JI_U43400.1_144861bp_1_980	GTAATAAGACTTGATTATACATAGCTTTTAATAAAATCTGTAAATAAATGGTTACAGGAGATGCACAATTAAAATTTTGATGACAGAGTTCAGCAAATAATGTCTTAAACAAATGAATTA
664157_30677280_HHV-7_JI_U43400.1_144861bp_1_981	ATGCACAATTAAAATTTTGATGACAGAGTTCAGCAAATAATGTCTTAAACAAATGAATTATTACAACACCGTTTTTCCTGAAATTTGTCATATCTGGAATAAGTGTGACAGGGTCACAAA
664157_30677280_HHV-7_JI_U43400.1_144861bp_1_982	TTACAACACCGTTTTTCCTGAAATTTGTCATATCTGGAATAAGTGTGACAGGGTCACAAAATCGTAAGCACTCTAATTCTAAAGCACATTCACTTAACCTTGAACACAAGACACACAATG
664157_30677280_HHV-7_JI_U43400.1_144861bp_1_983	ATCGTAAGCACTCTAATTCTAAAGCACATTCACTTAACCTTGAACACAAGACACACAATGATTGCAAAGAATTCATTTTTACAATCTAGCTTTTTTTAAGGAGGGTTCCTCATTTTCATG
664157_30677280_HHV-7_JI_U43400.1_144861bp_1_984	ATTGCAAAGAATTCATTTTTACAATCTAGCTTTTTTTAAGGAGGGTTCCTCATTTTCATGCTCAGTCTCTTCACATTTTAATATACCAAAACCTTGCTCATTACCTTCTACCAAAAGTTC
664157_30677280_HHV-7_JI_U43400.1_144861bp_1_985	CTCAGTCTCTTCACATTTTAATATACCAAAACCTTGCTCATTACCTTCTACCAAAAGTTCTGAAAAATCATAGATGGCAGCACTATCTTCTACTTGTTCTTCAAAAACTGACAATAAAGT

664157_30677280_HHV-7_JI_U43400.1_144861bp_1_986	TGAAAAATCATAGATGGCAGCACTATCTTCTACTTGTTCTTCAAAAACTGACAATAAAGTTGAAAGAGAAAAATCATGTACATTTAAATCCGTTAAATGCTGAATCTTGTTCATTACGGA
664157_30677280_HHV-7_JI_U43400.1_144861bp_1_987	TGAAAGAGAAAAATCATGTACATTTAAATCCGTTAAATGCTGAATCTTGTTCATTACGGATTTTGCTAGAGGCTCTCTACCATCCACGTAGAATAACATATCATCCATAGTAGGCTTACT
664157_30677280_HHV-7_JI_U43400.1_144861bp_1_988	TTTTGCTAGAGGCTCTCTACCATCCACGTAGAATAACATATCATCCATAGTAGGCTTACTATCTCTATCTTTCATCACTTCAGCTAATATGAGTAGTTCTGGATTTACAGAATTGGATAA
664157_30677280_HHV-7_JI_U43400.1_144861bp_1_989	ATCTCTATCTTTCATCACTTCAGCTAATATGAGTAGTTCTGGATTTACAGAATTGGATAAACTTTCAATAATGTTGAGCACCTTTCCTCGAATTACTTCCACATCATAAACAATGGCTTC
664157_30677280_HHV-7_JI_U43400.1_144861bp_1_990	ACTTTCAATAATGTTGAGCACCTTTCCTCGAATTACTTCCACATCATAAACAATGGCTTCTCTCTTAACTTTCTTCACAATAATGTCACACAGTTTTGTAGCAATCACAAATTTCTGACG
664157_30677280_HHV-7_JI_U43400.1_144861bp_1_991	TCTCTTAACTTTCTTCACAATAATGTCACACAGTTTTGTAGCAATCACAAATTTCTGACGCATAAAACGGAAATCTTGCAAGCCGGAGGAAGGAGGGTTTAAATTGCGATCTACACCACT
664157_30677280_HHV-7_JI_U43400.1_144861bp_1_992	CATAAAACGGAAATCTTGCAAGCCGGAGGAAGGAGGGTTTAAATTGCGATCTACACCACTTCCAGCTATCCAACCTAACTGACCAAAGTGGAAATAATCTCTACTAGCAACACTCACAAA
664157_30677280_HHV-7_JI_U43400.1_144861bp_1_993	TCCAGCTATCCAACCTAACTGACCAAAGTGGAAATAATCTCTACTAGCAACACTCACAAATTTTTCGATCATTAAACCAAAGGTAATAATAGATCTTCCTGTGAAAATGCTCCCAAAATT
664157_30677280_HHV-7_JI_U43400.1_144861bp_1_994	TTTTTCGATCATTAAACCAAAGGTAATAATAGATCTTCCTGTGAAAATGCTCCCAAAATTTGGTTCTTTTGTTAATGATGCAGTCATAGGTTCTTTAAGTCCATTCCCTACCAACTTTTT
664157_30677280_HHV-7_JI_U43400.1_144861bp_1_995	TGGTTCTTTTGTTAATGATGCAGTCATAGGTTCTTTAAGTCCATTCCCTACCAACTTTTTTACGTGCATGACGTATTCCTGCACAGAAGAAAATGTTGGATGTTCTTTAAGAAAAACTGG
664157_30677280_HHV-7_JI_U43400.1_144861bp_1_996	TACGTGCATGACGTATTCCTGCACAGAAGAAAATGTTGGATGTTCTTTAAGAAAAACTGGGAATAACATAGGGTTTTGAGGAACTAGTTTAGAAGTTAATGCATGCAAACTTGTTAGATA
664157_30677280_HHV-7_JI_U43400.1_144861bp_1_997	GAATAACATAGGGTTTTGAGGAACTAGTTTAGAAGTTAATGCATGCAAACTTGTTAGATAATCTCTATAACCAAGAACTGATAATAATTTATTATGAAAGTAATACTCGATAAAGGATAG

664157_30677280_HHV-7_JI_U43400.1_144861bp_1_998	ATCTCTATAACCAAGAACTGATAATAATTTATTATGAAAGTAATACTCGATAAAGGATAGTAAGCATTCTGGTTGAATATCTAACAAATCTATTTCATCATATGAATTGGTGACACGGAA
664157_30677280_HHV-7_JI_U43400.1_144861bp_1_999	TAAGCATTCTGGTTGAATATCTAACAAATCTATTTCATCATATGAATTGGTGACACGGAAAGTAAACTTGATGAAAGAACGCATGTCTTCAACATTACCTATATCTATGGTTTTTGGCAT
664157_30677280_HHV-7_JI_U43400.1_144861bp_1_1000	AGTAAACTTGATGAAAGAACGCATGTCTTCAACATTACCTATATCTATGGTTTTTGGCATGTTATTAAGCAAAATACGCTGCCAAAATTCTAGGCAGGATATTTTTAAATTTGGAAAGAG
664157_30677280_HHV-7_JI_U43400.1_144861bp_1_1001	GTTATTAAGCAAAATACGCTGCCAAAATTCTAGGCAGGATATTTTTAAATTTGGAAAGAGCCGTTCATGATATTTAAACAGCAAAAATGATAAGCAACCAGAAAGTGGGTTCTTTTTCTG
664157_30677280_HHV-7_JI_U43400.1_144861bp_1_1002	CCGTTCATGATATTTAAACAGCAAAAATGATAAGCAACCAGAAAGTGGGTTCTTTTTCTGTTTGACCTTTTTGAAAAAAGGATTATTTTGTGTGTTTTTGTTTGGCTTATTGAATGGTCT
664157_30677280_HHV-7_JI_U43400.1_144861bp_1_1003	TTTGACCTTTTTGAAAAAAGGATTATTTTGTGTGTTTTTGTTTGGCTTATTGAATGGTCTATTTTTTACTCGAAAATCTTTCAAACATTGCATGGAAAGGCGACAGAGTTTAGCTTGGAT
664157_30677280_HHV-7_JI_U43400.1_144861bp_1_1004	ATTTTTTACTCGAAAATCTTTCAAACATTGCATGGAAAGGCGACAGAGTTTAGCTTGGATAGAGGTTTTAGCATATTTACCCGATTTAAACAAATTGAAATCAATGTATTGTTCCATGTC
664157_30677280_HHV-7_JI_U43400.1_144861bp_1_1005	AGAGGTTTTAGCATATTTACCCGATTTAAACAAATTGAAATCAATGTATTGTTCCATGTCTACATTTGAGGCTATTTTTACAGTTTTCATATTCAAAAAACCTTTTTTAAAATGACTGTT
664157_30677280_HHV-7_JI_U43400.1_144861bp_1_1006	TACATTTGAGGCTATTTTTACAGTTTTCATATTCAAAAAACCTTTTTTAAAATGACTGTTGTAAAATGCTCCGTAGAGAGATTGGTATTGTTGCATTAACCATTTAGAAATTAAGTTTCC
664157_30677280_HHV-7_JI_U43400.1_144861bp_1_1007	GTAAAATGCTCCGTAGAGAGATTGGTATTGTTGCATTAACCATTTAGAAATTAAGTTTCCAGTACAAGGCCTATCTACAACATAGCCCGTACCGATTATTAAAGCCAAGTTCTGTAAAAC
664157_30677280_HHV-7_JI_U43400.1_144861bp_1_1008	AGTACAAGGCCTATCTACAACATAGCCCGTACCGATTATTAAAGCCAAGTTCTGTAAAACAATTAGTATAACTTTGTAATACGTGAAAACAAAGAATGGAGAAAAAGCCAAAGACAGAGG
664157_30677280_HHV-7_JI_U43400.1_144861bp_1_1009	AATTAGTATAACTTTGTAATACGTGAAAACAAAGAATGGAGAAAAAGCCAAAGACAGAGGTGTCGTATCAATGTTGAAAGATTGTAGACAGTTTTCGATTTGTTCTCTGGATGTCTGCGT

664157_30677280_HHV-7_JI_U43400.1_144861bp_1_1010	TGTCGTATCAATGTTGAAAGATTGTAGACAGTTTTCGATTTGTTCTCTGGATGTCTGCGTTTTTCTCATTTCTGAAATACATTTTGAAACTGCTTCCTCAATGCATTGAATTAAATCATT
664157_30677280_HHV-7_JI_U43400.1_144861bp_1_1011	TTTTCTCATTTCTGAAATACATTTTGAAACTGCTTCCTCAATGCATTGAATTAAATCATTAATCATATTCACAAAATCATTTTGACTTTTAAAATTTAAATTGTCTTCCCCAGTTATTGC
664157_30677280_HHV-7_JI_U43400.1_144861bp_1_1012	AATCATATTCACAAAATCATTTTGACTTTTAAAATTTAAATTGTCTTCCCCAGTTATTGCATCAATCAAATTATTTTTCTTACAATAATCGTGAATCATTCCTAAGAATTTGAATCTATC
664157_30677280_HHV-7_JI_U43400.1_144861bp_1_1013	ATCAATCAAATTATTTTTCTTACAATAATCGTGAATCATTCCTAAGAATTTGAATCTATCTAAGGAAGGTGTGGTTTGAGCTTCTTTCATTGGATCTTTGCTTTCATTAACTCCCTTTTT
664157_30677280_HHV-7_JI_U43400.1_144861bp_1_1014	TAAGGAAGGTGTGGTTTGAGCTTCTTTCATTGGATCTTTGCTTTCATTAACTCCCTTTTTCCCAAAAGAACCTAGAACATCTACATCTGCATAAAAACGAGAAAACATTGTCATAACAAT
664157_30677280_HHV-7_JI_U43400.1_144861bp_1_1015	CCCAAAAGAACCTAGAACATCTACATCTGCATAAAAACGAGAAAACATTGTCATAACAATAGGTTCTTTTTTAGTCGTTTTAGAAATCTGAGGTAATCTGCTGTTGATTCTGATCAATGC
664157_30677280_HHV-7_JI_U43400.1_144861bp_1_1016	AGGTTCTTTTTTAGTCGTTTTAGAAATCTGAGGTAATCTGCTGTTGATTCTGATCAATGCTGTTCCGATACACGAATGGCAACACTTACCTTCACAAAACTCACAGAGATTAGAGGAACA
664157_30677280_HHV-7_JI_U43400.1_144861bp_1_1017	TGTTCCGATACACGAATGGCAACACTTACCTTCACAAAACTCACAGAGATTAGAGGAACAATTTACGATGTGGTTATATATTTCAGAATTTCCACAGTTCGTGTTATAAATAGACATTCT
664157_30677280_HHV-7_JI_U43400.1_144861bp_1_1018	ATTTACGATGTGGTTATATATTTCAGAATTTCCACAGTTCGTGTTATAAATAGACATTCTGTTCAAATTCCATATTATATGCGATAAGATTTGTGGACATGTCGCACATGCGTAAGCTAG
664157_30677280_HHV-7_JI_U43400.1_144861bp_1_1019	GTTCAAATTCCATATTATATGCGATAAGATTTGTGGACATGTCGCACATGCGTAAGCTAGATGAAAAGCGGAAAATTTATCTTCTTTACAAGGTGAAGAATTACACTTTATCAATTTTCT
664157_30677280_HHV-7_JI_U43400.1_144861bp_1_1020	ATGAAAAGCGGAAAATTTATCTTCTTTACAAGGTGAAGAATTACACTTTATCAATTTTCTGGCATCGTTATAAAAATCTTCGTTAAGAGATGACAAGGAATTACAAAACTGGATTGAACG
664157_30677280_HHV-7_JI_U43400.1_144861bp_1_1021	GGCATCGTTATAAAAATCTTCGTTAAGAGATGACAAGGAATTACAAAACTGGATTGAACGCAGTAATGTTTCTTGAGTGATCGCAGAGTTAAAAGCTTGTTTTGTGTTAGATATATAGGC

664157_30677280_HHV-7_JI_U43400.1_144861bp_1_1022	CAGTAATGTTTCTTGAGTGATCGCAGAGTTAAAAGCTTGTTTTGTGTTAGATATATAGGCCAATTTGTTACTGTAAAGTATCGAATTCGCAGCAAAGACTAAAGCAGCTACATGGCTAGA
664157_30677280_HHV-7_JI_U43400.1_144861bp_1_1023	CAATTTGTTACTGTAAAGTATCGAATTCGCAGCAAAGACTAAAGCAGCTACATGGCTAGATAGATGGAGCCTCAATTTTTTCAGTTCCACGATTTTCTCTTCGTGGGTTTCAGCCGATTT
664157_30677280_HHV-7_JI_U43400.1_144861bp_1_1024	TAGATGGAGCCTCAATTTTTTCAGTTCCACGATTTTCTCTTCGTGGGTTTCAGCCGATTTGATGATGGGCCACTCTGAAAAATTCATGATTTGATTATTTTCATAAATAGAATCAAGATA
664157_30677280_HHV-7_JI_U43400.1_144861bp_1_1025	GATGATGGGCCACTCTGAAAAATTCATGATTTGATTATTTTCATAAATAGAATCAAGATACTCTGCTGTGTAACTGAAGGTTAGTTCCGTGATTACAGCGTCCACTAACATTAAATGATC
664157_30677280_HHV-7_JI_U43400.1_144861bp_1_1026	CTCTGCTGTGTAACTGAAGGTTAGTTCCGTGATTACAGCGTCCACTAACATTAAATGATCTTTTTCAATCGGTGATAGTTTTTGACCAGGAATACCGTGATATGTTTTATTCGGTGCAAG
664157_30677280_HHV-7_JI_U43400.1_144861bp_1_1027	TTTTTCAATCGGTGATAGTTTTTGACCAGGAATACCGTGATATGTTTTATTCGGTGCAAGCTTGACAGTTTGTTCAGTATCTTGGATAAATTGTTTCAGACCGGCTTTAATCAATTCCAG
664157_30677280_HHV-7_JI_U43400.1_144861bp_1_1028	CTTGACAGTTTGTTCAGTATCTTGGATAAATTGTTTCAGACCGGCTTTAATCAATTCCAGGGTATTATTAAATCGCAAAGTCATTCCCCATGATGTAAAGATTAAGTAGAACAATACTTC
664157_30677280_HHV-7_JI_U43400.1_144861bp_1_1029	GGTATTATTAAATCGCAAAGTCATTCCCCATGATGTAAAGATTAAGTAGAACAATACTTCGCTGATCTGAGGCGCATAAAAGCCGTTTTCTTGAATGAATTCAGTGCAAGTATCGATGCA
664157_30677280_HHV-7_JI_U43400.1_144861bp_1_1030	GCTGATCTGAGGCGCATAAAAGCCGTTTTCTTGAATGAATTCAGTGCAAGTATCGATGCACAAAGTTTCTTCCGATTCAAACAAGGTAGCCGAATATAACGGAATTTTTATTGCCAAGCA
664157_30677280_HHV-7_JI_U43400.1_144861bp_1_1031	CAAAGTTTCTTCCGATTCAAACAAGGTAGCCGAATATAACGGAATTTTTATTGCCAAGCATTCACCAACCTGGACTTGAATCTGCTCTTCTACACATGGGATTAATAATCCAGCAAATAA
664157_30677280_HHV-7_JI_U43400.1_144861bp_1_1032	TTCACCAACCTGGACTTGAATCTGCTCTTCTACACATGGGATTAATAATCCAGCAAATAAAAGTTCTTTGAAACCATTCCCAACAACTATATGACATAGAACGTCATCTGCATTTCTTCC
664157_30677280_HHV-7_JI_U43400.1_144861bp_1_1033	AAGTTCTTTGAAACCATTCCCAACAACTATATGACATAGAACGTCATCTGCATTTCTTCCAACACTTTCACAGATCTTTCCTATATCCACAGACTTTCGATTTCCGTTAACGACAAATTC

664157_30677280_HHV-7_JI_U43400.1_144861bp_1_1034	AACACTTTCACAGATCTTTCCTATATCCACAGACTTTCGATTTCCGTTAACGACAAATTCTTGTAGATTGAATTTTTCTCTTGTTTGTTTGCACAATTTATCAAGATTTCCATGGTCTTC
664157_30677280_HHV-7_JI_U43400.1_144861bp_1_1035	TTGTAGATTGAATTTTTCTCTTGTTTGTTTGCACAATTTATCAAGATTTCCATGGTCTTCTGCTTCTTTTAAAAAAACATCTGTACCGTGGAAAAAGAAGCATACAGGCATAAACGATGT
664157_30677280_HHV-7_JI_U43400.1_144861bp_1_1036	TGCTTCTTTTAAAAAAACATCTGTACCGTGGAAAAAGAAGCATACAGGCATAAACGATGTGATTTTGGTAATGACTGTTCCGCCGTAATTAATAATGGGTGTTTTAACGGTTGGGAAGAA
664157_30677280_HHV-7_JI_U43400.1_144861bp_1_1037	GATTTTGGTAATGACTGTTCCGCCGTAATTAATAATGGGTGTTTTAACGGTTGGGAAGAAGTCATTTTCAACCGTTAGATTAAGTAACAAGGGTGAGATCACTACTGATTTTCTTTTTTC
664157_30677280_HHV-7_JI_U43400.1_144861bp_1_1038	GTCATTTTCAACCGTTAGATTAAGTAACAAGGGTGAGATCACTACTGATTTTCTTTTTTCCATTAACGATAATGTAGTCAGAATTTCTATTAATTTTTGTTCTTTCGGCAGAATGTACAG
664157_30677280_HHV-7_JI_U43400.1_144861bp_1_1039	CATTAACGATAATGTAGTCAGAATTTCTATTAATTTTTGTTCTTTCGGCAGAATGTACAGCCATGCTGCGGTACATATTGGGGCCGAAACAACAGTTTCATTATCATCAGCCATTCTTGA
664157_30677280_HHV-7_JI_U43400.1_144861bp_1_1040	CCATGCTGCGGTACATATTGGGGCCGAAACAACAGTTTCATTATCATCAGCCATTCTTGAAACATGAAGACCCCAGTTTCTGCAAGGGGTGTTTCTGCTAAACCTTATATATTTTAGTTG
664157_30677280_HHV-7_JI_U43400.1_144861bp_1_1041	AACATGAAGACCCCAGTTTCTGCAAGGGGTGTTTCTGCTAAACCTTATATATTTTAGTTGACGTAACAAAACTTAGCGTCATCATACAACGGAAATTAAGTTTTCTCGTTCTACTGTTTT
664157_30677280_HHV-7_JI_U43400.1_144861bp_1_1042	ACGTAACAAAACTTAGCGTCATCATACAACGGAAATTAAGTTTTCTCGTTCTACTGTTTTAGTGCGGAGAACTAAAAACGATATACTGACTCGGATTTGATAGTTTATGCAAATTAGCGT
664157_30677280_HHV-7_JI_U43400.1_144861bp_1_1043	AGTGCGGAGAACTAAAAACGATATACTGACTCGGATTTGATAGTTTATGCAAATTAGCGTCCACCTCACTCGTAATAGTATTTATTTTTCATGGTTTTTAAAATAGAATTGCAGGCAAGG
664157_30677280_HHV-7_JI_U43400.1_144861bp_1_1044	CCACCTCACTCGTAATAGTATTTATTTTTCATGGTTTTTAAAATAGAATTGCAGGCAAGGAGGATCAATGAACCCTCCCACTTCCGCAAAGTAAGCAACGACCAATCAGAAGTTTGCATT
664157_30677280_HHV-7_JI_U43400.1_144861bp_1_1045	AGGATCAATGAACCCTCCCACTTCCGCAAAGTAAGCAACGACCAATCAGAAGTTTGCATTGCAAGTATTTAATTTGTTGCAGCAAAGCATTAGCTAGAGGACAGCCACAGAGATGCTACA

664157_30677280_HHV-7_JI_U43400.1_144861bp_1_1046	GCAAGTATTTAATTTGTTGCAGCAAAGCATTAGCTAGAGGACAGCCACAGAGATGCTACATATTGGGAAATTAATCCGAATCATCAGAATCAGTGTCATTAATGTCGGTCTCACAAGTTT
664157_30677280_HHV-7_JI_U43400.1_144861bp_1_1047	TATTGGGAAATTAATCCGAATCATCAGAATCAGTGTCATTAATGTCGGTCTCACAAGTTTCTACATCTTCATAATCTAAATCCAGTTCCATTTCATTATCAGAGTTTTCCTCTCCATTGT
664157_30677280_HHV-7_JI_U43400.1_144861bp_1_1048	CTACATCTTCATAATCTAAATCCAGTTCCATTTCATTATCAGAGTTTTCCTCTCCATTGTTTTCTTTAATTTTCGCTGATTTCATAGAAGTATCATTTTCAATAAATACAAAGCGAGTTT
664157_30677280_HHV-7_JI_U43400.1_144861bp_1_1049	TTTCTTTAATTTTCGCTGATTTCATAGAAGTATCATTTTCAATAAATACAAAGCGAGTTTCATCTTCCTGTAGAGTCGTGTTTGTTTCAAAATCTATAGAATCAACTTCTTTACTCCAGT
664157_30677280_HHV-7_JI_U43400.1_144861bp_1_1050	CATCTTCCTGTAGAGTCGTGTTTGTTTCAAAATCTATAGAATCAACTTCTTTACTCCAGTCTTTTTTCAGTCTGTTTTCGCGTAGCTCATTTGTTTTCTGTCCTTTATTCGTTTTATCCG
664157_30677280_HHV-7_JI_U43400.1_144861bp_1_1051	CTTTTTTCAGTCTGTTTTCGCGTAGCTCATTTGTTTTCTGTCCTTTATTCGTTTTATCCGCAGTAAGAAGCTTTTTACATTTGGCTTGCTTTTTGGTTTGTAAATTCAATTGGACGGTTT
664157_30677280_HHV-7_JI_U43400.1_144861bp_1_1052	CAGTAAGAAGCTTTTTACATTTGGCTTGCTTTTTGGTTTGTAAATTCAATTGGACGGTTTGCTTAGATTGCTGTGAAGCAAAGCTGCAAGACGGAGTTGTAGAACATGCAACATTAAAGC
664157_30677280_HHV-7_JI_U43400.1_144861bp_1_1053	GCTTAGATTGCTGTGAAGCAAAGCTGCAAGACGGAGTTGTAGAACATGCAACATTAAAGCTGTTGTACTCTTCAGGCATGTTAGGATGCAGACCAAACTCCACAAATTCTTTGGGAAGAT
664157_30677280_HHV-7_JI_U43400.1_144861bp_1_1054	TGTTGTACTCTTCAGGCATGTTAGGATGCAGACCAAACTCCACAAATTCTTTGGGAAGATAGGTACAGAAATATAAGCTTGGTTTCCAAGGTGACATCATGCGTTGGATTCTAGTTTGGC
664157_30677280_HHV-7_JI_U43400.1_144861bp_1_1055	AGGTACAGAAATATAAGCTTGGTTTCCAAGGTGACATCATGCGTTGGATTCTAGTTTGGCAACCGATGTCATCACATTGATGATTGTTAAATTGGTCTCCGACTAATCTGGAAATCATGC
664157_30677280_HHV-7_JI_U43400.1_144861bp_1_1056	AACCGATGTCATCACATTGATGATTGTTAAATTGGTCTCCGACTAATCTGGAAATCATGCCACATTGATATTGTTCACTTACACGATCTTGGTCAAAATCGCGTATGAACTGAGGTTGTT
664157_30677280_HHV-7_JI_U43400.1_144861bp_1_1057	CACATTGATATTGTTCACTTACACGATCTTGGTCAAAATCGCGTATGAACTGAGGTTGTTTAGTTTGAAGAATACGTTCCTTTATTTGACGTAAAATCCTTGCAAAAGTTACTATAAATG

664157_30677280_HHV-7_JI_U43400.1_144861bp_1_1058	TAGTTTGAAGAATACGTTCCTTTATTTGACGTAAAATCCTTGCAAAAGTTACTATAAATGATAAAGCTTGCTGTGCTGGATATTGAAAAGATCCAGGAGTCTGTTGCTGTACTTCATATT
664157_30677280_HHV-7_JI_U43400.1_144861bp_1_1059	ATAAAGCTTGCTGTGCTGGATATTGAAAAGATCCAGGAGTCTGTTGCTGTACTTCATATTCCGTGTTAGGCATATCACGGTCATATACCGGTTTTCCACAAAGTTGTCTGAAAATACGGT
664157_30677280_HHV-7_JI_U43400.1_144861bp_1_1060	CCGTGTTAGGCATATCACGGTCATATACCGGTTTTCCACAAAGTTGTCTGAAAATACGGTATTCTATGTCACCATGTCCATTGTCATTAAAAACACAACAAAGTTTTGCCATATTCAACT
664157_30677280_HHV-7_JI_U43400.1_144861bp_1_1061	ATTCTATGTCACCATGTCCATTGTCATTAAAAACACAACAAAGTTTTGCCATATTCAACTTGTTGAAGTATTCAAACTTCATTATGGTAATAGTATACATAAACAGATCTTGAGGGTCAA
664157_30677280_HHV-7_JI_U43400.1_144861bp_1_1062	TGTTGAAGTATTCAAACTTCATTATGGTAATAGTATACATAAACAGATCTTGAGGGTCAATAGGTAAGTTGTGCACAAAATGATATTTATAGTAAATGCAAGCTTCCTCAATAATCGCAA
664157_30677280_HHV-7_JI_U43400.1_144861bp_1_1063	TAGGTAAGTTGTGCACAAAATGATATTTATAGTAAATGCAAGCTTCCTCAATAATCGCAAGAGAAAATGCTAACGATGGAGAATTCGTCAATGAAAAAACTCGGTCTCCGAGTATCTGTT
664157_30677280_HHV-7_JI_U43400.1_144861bp_1_1064	GAGAAAATGCTAACGATGGAGAATTCGTCAATGAAAAAACTCGGTCTCCGAGTATCTGTTGATGTTGTTCTCTATTGAGGGAGTTTAAGCAGTTCTTATATCGTAGTAACAAGTCTGTTG
664157_30677280_HHV-7_JI_U43400.1_144861bp_1_1065	GATGTTGTTCTCTATTGAGGGAGTTTAAGCAGTTCTTATATCGTAGTAACAAGTCTGTTGTCATTAGTTCTTCTTTTTCCAAATAGATTTTTTCATGATTCCGGGGAAATCGATCTCGAA
664157_30677280_HHV-7_JI_U43400.1_144861bp_1_1066	TCATTAGTTCTTCTTTTTCCAAATAGATTTTTTCATGATTCCGGGGAAATCGATCTCGAATCCTATGAGCTGGATCTCGTGCTTCACCGGAATACTTGCGTAAACCAAGGGCTTCCGCGG
664157_30677280_HHV-7_JI_U43400.1_144861bp_1_1067	TCCTATGAGCTGGATCTCGTGCTTCACCGGAATACTTGCGTAAACCAAGGGCTTCCGCGGTGTAGCTAGAGCCAAAAGGTGCTGTTGGTAAATTGGCGAAAAAAGTCAATGCATGCGTCT
664157_30677280_HHV-7_JI_U43400.1_144861bp_1_1068	TGTAGCTAGAGCCAAAAGGTGCTGTTGGTAAATTGGCGAAAAAAGTCAATGCATGCGTCTGTGCACGCTTTTCTTCCTGCTTTTCTTCAATGATGAGTTTCAGCTGTTCTAGTTCTCTGA
664157_30677280_HHV-7_JI_U43400.1_144861bp_1_1069	GTGCACGCTTTTCTTCCTGCTTTTCTTCAATGATGAGTTTCAGCTGTTCTAGTTCTCTGAGACTCAAAGCGTCTAATTTTGATTCATCAAGCTGCTTAATAGGAATGATGCGTCTGTGAA

664157_30677280_HHV-7_JI_U43400.1_144861bp_1_1070	GACTCAAAGCGTCTAATTTTGATTCATCAAGCTGCTTAATAGGAATGATGCGTCTGTGAAATTGTTTCGTAAAGCGGCTGACATACTTGTTAGCAGGTTTATGAACAAGAGTTCGTTTAA
664157_30677280_HHV-7_JI_U43400.1_144861bp_1_1071	ATTGTTTCGTAAAGCGGCTGACATACTTGTTAGCAGGTTTATGAACAAGAGTTCGTTTAATGGTTTTCAAACCATATCGTTGCCGCCCTAATACATTCTTTTTAACCCCGCGTGGATACA
664157_30677280_HHV-7_JI_U43400.1_144861bp_1_1072	TGGTTTTCAAACCATATCGTTGCCGCCCTAATACATTCTTTTTAACCCCGCGTGGATACATGTTTCTGTCGAAAATGAGAACCTTGTTCATAAACGGCCTTTTATATTATTTTTGTCTCG
664157_30677280_HHV-7_JI_U43400.1_144861bp_1_1073	TGTTTCTGTCGAAAATGAGAACCTTGTTCATAAACGGCCTTTTATATTATTTTTGTCTCGTGTAGCGGTGACGCCTCTGTGGTTTTTAATATAAGGAACATTTGACATATAAAGCTAGAC
664157_30677280_HHV-7_JI_U43400.1_144861bp_1_1074	TGTAGCGGTGACGCCTCTGTGGTTTTTAATATAAGGAACATTTGACATATAAAGCTAGACACGAATTAAGGTGCGTCTAAAATTTTATTTACAAAACACAAATAAATAACATAAAAATGT
664157_30677280_HHV-7_JI_U43400.1_144861bp_1_1075	ACGAATTAAGGTGCGTCTAAAATTTTATTTACAAAACACAAATAAATAACATAAAAATGTCACATATTCAAAGAATCTAAAACGACAGATGAATGCACTTGCTTACTATTAGATTTACAT
664157_30677280_HHV-7_JI_U43400.1_144861bp_1_1076	CACATATTCAAAGAATCTAAAACGACAGATGAATGCACTTGCTTACTATTAGATTTACATTTCGTAGTAAAACATTTGCTCCATAATATTATCAAAATGCGATTGTTATCTACCTTAAAT
664157_30677280_HHV-7_JI_U43400.1_144861bp_1_1077	TTCGTAGTAAAACATTTGCTCCATAATATTATCAAAATGCGATTGTTATCTACCTTAAATTCTAAGTACACTAAGACATTTTCACGGTTTCCCTTATGATTCCTTGTTAAACAACTGAAA
664157_30677280_HHV-7_JI_U43400.1_144861bp_1_1078	TCTAAGTACACTAAGACATTTTCACGGTTTCCCTTATGATTCCTTGTTAAACAACTGAAATCACTTAGTTTTCCGTTTTGCAATTTTTTTAGTTGTATATTTTTGTGATCAATTTTTAGA
664157_30677280_HHV-7_JI_U43400.1_144861bp_1_1079	TCACTTAGTTTTCCGTTTTGCAATTTTTTTAGTTGTATATTTTTGTGATCAATTTTTAGAAAAGTTACATGTTGAAATTGTGTAGCTGTTCGTGCTTCACAGTGTTGGATGTTCTTTTTT
664157_30677280_HHV-7_JI_U43400.1_144861bp_1_1080	AAAGTTACATGTTGAAATTGTGTAGCTGTTCGTGCTTCACAGTGTTGGATGTTCTTTTTTAACATTTGTGGCCAGGCAATTGAACGAGTAAAAGTCATTAACAAATCATCACGGGAAAGA
664157_30677280_HHV-7_JI_U43400.1_144861bp_1_1081	AACATTTGTGGCCAGGCAATTGAACGAGTAAAAGTCATTAACAAATCATCACGGGAAAGACAACTTGTTTCCGAAAAACCATTGACATGTAAATATTTCAAACATAAAGTCTCCAGTGAA

664157_30677280_HHV-7_JI_U43400.1_144861bp_1_1082	CAACTTGTTTCCGAAAAACCATTGACATGTAAATATTTCAAACATAAAGTCTCCAGTGAACTTTGTTTTCTGTGATACAGTTGCTTAGTCCTAGAATCTATAAAAGAAAACTCTGTTGGA
664157_30677280_HHV-7_JI_U43400.1_144861bp_1_1083	CTTTGTTTTCTGTGATACAGTTGCTTAGTCCTAGAATCTATAAAAGAAAACTCTGTTGGACAGGCCTTATCAAAAATGCTGTAGATTATCTCTGTAATGGTTTGGTCTTTAGTTCCATGA
664157_30677280_HHV-7_JI_U43400.1_144861bp_1_1084	CAGGCCTTATCAAAAATGCTGTAGATTATCTCTGTAATGGTTTGGTCTTTAGTTCCATGATGTAAAATATTATTGAGATCTAGTTGCATGCTAACGAAATCTTGAAAATTCTTTTTCATT
664157_30677280_HHV-7_JI_U43400.1_144861bp_1_1085	TGTAAAATATTATTGAGATCTAGTTGCATGCTAACGAAATCTTGAAAATTCTTTTTCATTTTTTCCGGGACAATAAAAATAGGCTTCAGACGACCATACAAAAATCTGTTATTCTCTTCG
664157_30677280_HHV-7_JI_U43400.1_144861bp_1_1086	TTTTCCGGGACAATAAAAATAGGCTTCAGACGACCATACAAAAATCTGTTATTCTCTTCGTCAACTTTATACATGAAACCTAACCGTAAACAACGTCCCGTATTATAGATTCCTGTATCA
664157_30677280_HHV-7_JI_U43400.1_144861bp_1_1087	TCAACTTTATACATGAAACCTAACCGTAAACAACGTCCCGTATTATAGATTCCTGTATCAAAACATTCACCAGGAAAAATAACCGAGTTAAGAATCTGCATTAAATCGTGGTTTAGACAC
664157_30677280_HHV-7_JI_U43400.1_144861bp_1_1088	AAACATTCACCAGGAAAAATAACCGAGTTAAGAATCTGCATTAAATCGTGGTTTAGACACATTAAATGATTGAATATTTTAGAAAGTTGTTTTAATGGCTCACTTCCAATTATTGCAGTA
664157_30677280_HHV-7_JI_U43400.1_144861bp_1_1089	ATTAAATGATTGAATATTTTAGAAAGTTGTTTTAATGGCTCACTTCCAATTATTGCAGTACCTTGAGGGATTGGAATAGCAATTCGAAGACCAATTTTTTTTTTGCAAACACAAAAAGCT
664157_30677280_HHV-7_JI_U43400.1_144861bp_1_1090	CCTTGAGGGATTGGAATAGCAATTCGAAGACCAATTTTTTTTTTGCAAACACAAAAAGCTGTTTCAACTTCATTGTAACATACATTGTGGTTCAGGGGATTCTCCGGATTACTACAGGTA
664157_30677280_HHV-7_JI_U43400.1_144861bp_1_1091	GTTTCAACTTCATTGTAACATACATTGTGGTTCAGGGGATTCTCCGGATTACTACAGGTAGTTTTGAAAAAAAAGATGGGGTAGGCGTCTTTGTCAATAAACGGAAATATTTTTTGCCTG
664157_30677280_HHV-7_JI_U43400.1_144861bp_1_1092	GTTTTGAAAAAAAAGATGGGGTAGGCGTCTTTGTCAATAAACGGAAATATTTTTTGCCTGCATTGATCAGCACATTCCGCATCAATTTACACATAGAGAAAAATAACGATTCCGTTATTA
664157_30677280_HHV-7_JI_U43400.1_144861bp_1_1093	CATTGATCAGCACATTCCGCATCAATTTACACATAGAGAAAAATAACGATTCCGTTATTATAGCTGAATCCTGTAATGGCAGATCTAAATCACCAATATAATTTGTAACCGGAAGGTATT

664157_30677280_HHV-7_JI_U43400.1_144861bp_1_1094	TAGCTGAATCCTGTAATGGCAGATCTAAATCACCAATATAATTTGTAACCGGAAGGTATTCATTAAAGATTTCATGCCTGGTTACGTAAAACTGTTTTTCTATTTCGTCGCTTAAAAAGG
664157_30677280_HHV-7_JI_U43400.1_144861bp_1_1095	CATTAAAGATTTCATGCCTGGTTACGTAAAACTGTTTTTCTATTTCGTCGCTTAAAAAGGTCGTGTCCTGCAACCAAAATGTACTTGTTAAAGATTCATCCGAAATGTACTCATTGGGTA
664157_30677280_HHV-7_JI_U43400.1_144861bp_1_1096	TCGTGTCCTGCAACCAAAATGTACTTGTTAAAGATTCATCCGAAATGTACTCATTGGGTAAAAATTGCATAACCTGGTCGAAACCCATATTTTTGTACCAACTTTCTGCACTTCCAAAAC
664157_30677280_HHV-7_JI_U43400.1_144861bp_1_1097	AAAATTGCATAACCTGGTCGAAACCCATATTTTTGTACCAACTTTCTGCACTTCCAAAACAATAAAGATTTTTGTAAGACATTTGAACTCTAAACATAGGAATGGGGTTGCTGAATTGGT
664157_30677280_HHV-7_JI_U43400.1_144861bp_1_1098	AATAAAGATTTTTGTAAGACATTTGAACTCTAAACATAGGAATGGGGTTGCTGAATTGGTCATGTAGATATTGTCTCCTTGTAACAGGTTTACCATCTATCAAAATAGATTTATTAGATG
664157_30677280_HHV-7_JI_U43400.1_144861bp_1_1099	CATGTAGATATTGTCTCCTTGTAACAGGTTTACCATCTATCAAAATAGATTTATTAGATGCCAAAATTTTTAAAAATCCTTGTAAGCCTAATTTAATTGGTGTAAAATACCCTTCACCGT
664157_30677280_HHV-7_JI_U43400.1_144861bp_1_1100	CCAAAATTTTTAAAAATCCTTGTAAGCCTAATTTAATTGGTGTAAAATACCCTTCACCGTGTTTATTGCATTTGTTAACCATCTTTGAAATTTGGTCTGAATTTATTACAAGCATGATAT
664157_30677280_HHV-7_JI_U43400.1_144861bp_1_1101	GTTTATTGCATTTGTTAACCATCTTTGAAATTTGGTCTGAATTTATTACAAGCATGATATTTCTATTACTGTTTATGTCATAAACGTACTGTTGCATTTGAAATTTGTTGTTTAACATCC
664157_30677280_HHV-7_JI_U43400.1_144861bp_1_1102	TTCTATTACTGTTTATGTCATAAACGTACTGTTGCATTTGAAATTTGTTGTTTAACATCCTTTTTATTTCTACATAATTTTGAAAATAACTGTCTTCGTTAAAGTACTGTTTCATGATTG
664157_30677280_HHV-7_JI_U43400.1_144861bp_1_1103	TTTTTATTTCTACATAATTTTGAAAATAACTGTCTTCGTTAAAGTACTGTTTCATGATTGAAATGAGATCCGAGTGTAAAGAATTTCCTAGGATGTACATATTTTCCTGACATTGAGAAG
664157_30677280_HHV-7_JI_U43400.1_144861bp_1_1104	AAATGAGATCCGAGTGTAAAGAATTTCCTAGGATGTACATATTTTCCTGACATTGAGAAGTATCAGATCTGGTAGGATCATAAGATGTCAGTTGACTATACTTGATGAATTTATTTTTTT
664157_30677280_HHV-7_JI_U43400.1_144861bp_1_1105	TATCAGATCTGGTAGGATCATAAGATGTCAGTTGACTATACTTGATGAATTTATTTTTTTCTAATGCTGTACTGTAAGCAATGTAGATATAGTGGACTCGAAAACTGTTTGTGAGATGCA

664157_30677280_HHV-7_JI_U43400.1_144861bp_1_1106	CTAATGCTGTACTGTAAGCAATGTAGATATAGTGGACTCGAAAACTGTTTGTGAGATGCACGCTGTTTCTAAAAATATCTATTTGCTTATCAAGATAAAGAAATTCTTTTCGATCTCTTA
664157_30677280_HHV-7_JI_U43400.1_144861bp_1_1107	CGCTGTTTCTAAAAATATCTATTTGCTTATCAAGATAAAGAAATTCTTTTCGATCTCTTATGTTTTTTTGTTTTGCATATTCAGAAAAACTAACAAATTTTTTCCGATAGTTAGATTTCA
664157_30677280_HHV-7_JI_U43400.1_144861bp_1_1108	TGTTTTTTTGTTTTGCATATTCAGAAAAACTAACAAATTTTTTCCGATAGTTAGATTTCAAAGTAATATTAATGAATTCAGACATGGAGGAATAAGTAAATACCATATCTCCTAATTTAT
664157_30677280_HHV-7_JI_U43400.1_144861bp_1_1109	AAGTAATATTAATGAATTCAGACATGGAGGAATAAGTAAATACCATATCTCCTAATTTATCAGTGAAACACTGAACATAATTCTTAGTTGTTGCTATGGTGCTTTTCTGCTCTTCAAATA
664157_30677280_HHV-7_JI_U43400.1_144861bp_1_1110	CAGTGAAACACTGAACATAATTCTTAGTTGTTGCTATGGTGCTTTTCTGCTCTTCAAATAAATAATAATACATCGTTAATAGTAGCATTCCTTCTGTTTGACCGTATGTTGATAAAAACC
664157_30677280_HHV-7_JI_U43400.1_144861bp_1_1111	AATAATAATACATCGTTAATAGTAGCATTCCTTCTGTTTGACCGTATGTTGATAAAAACCAAAACGGTGACGTGGGTAAGAGATACTTATTTGTTAGATGTTTAATGATTATAGTCTTGC
664157_30677280_HHV-7_JI_U43400.1_144861bp_1_1112	AAAACGGTGACGTGGGTAAGAGATACTTATTTGTTAGATGTTTAATGATTATAGTCTTGCAAAAAAAGACTAAGCTTTTAAATTCTATATTTTCACTTCCTTCTGGAACATGAGAAAGTA
664157_30677280_HHV-7_JI_U43400.1_144861bp_1_1113	AAAAAAAGACTAAGCTTTTAAATTCTATATTTTCACTTCCTTCTGGAACATGAGAAAGTAAAAAATCTTTATACAGATTTTCTGTTTCTTCCGTATTTAAACAGTGAATAATATCCTCAG
664157_30677280_HHV-7_JI_U43400.1_144861bp_1_1114	AAAAATCTTTATACAGATTTTCTGTTTCTTCCGTATTTAAACAGTGAATAATATCCTCAGACTTAATGGGTAATGCATTTTTTACATAATAGCTGAAATTCAAAGATTCTGCGTCGCAAA
664157_30677280_HHV-7_JI_U43400.1_144861bp_1_1115	ACTTAATGGGTAATGCATTTTTTACATAATAGCTGAAATTCAAAGATTCTGCGTCGCAAACAAATACCGTCTCAATTCTTTTGGAAAACTTGCATTTCTGTGTCTGCATACAAAAATATA
664157_30677280_HHV-7_JI_U43400.1_144861bp_1_1116	CAAATACCGTCTCAATTCTTTTGGAAAACTTGCATTTCTGTGTCTGCATACAAAAATATACATTTTTTGATGGTTTATATTTAACGATTATTGGAAAAATTAAATGTTCACTAGGGTTCT
664157_30677280_HHV-7_JI_U43400.1_144861bp_1_1117	CATTTTTTGATGGTTTATATTTAACGATTATTGGAAAAATTAAATGTTCACTAGGGTTCTTACATAAGATATTTACTATCACGTGCGCGGGGTCATATTCCGTTGCAAACACAGTAATAG

664157_30677280_HHV-7_JI_U43400.1_144861bp_1_1118	TACATAAGATATTTACTATCACGTGCGCGGGGTCATATTCCGTTGCAAACACAGTAATAGTCATTGTGAATGTCTTTCAGTTTATCTTCCAACAGCTTTTAATATAAGACAATAGGAATC
664157_30677280_HHV-7_JI_U43400.1_144861bp_1_1119	TCATTGTGAATGTCTTTCAGTTTATCTTCCAACAGCTTTTAATATAAGACAATAGGAATCATGGGAAATTGCTTTGTAAAAAAAATCTCTTCTGACATCTTCAACTCTAACTACCAGATG
664157_30677280_HHV-7_JI_U43400.1_144861bp_1_1120	ATGGGAAATTGCTTTGTAAAAAAAATCTCTTCTGACATCTTCAACTCTAACTACCAGATGCTCTATTCTGAGCTCTCAGAGCAAGAGGATTTACTGGATTTTTTAGAGACAAAATATACA
664157_30677280_HHV-7_JI_U43400.1_144861bp_1_1121	CTCTATTCTGAGCTCTCAGAGCAAGAGGATTTACTGGATTTTTTAGAGACAAAATATACAGATTTTGGAATTTTAAAAACCGATATTCTCAACTATGAAAGAGACTCCGAAACATTCAAA
664157_30677280_HHV-7_JI_U43400.1_144861bp_1_1122	GATTTTGGAATTTTAAAAACCGATATTCTCAACTATGAAAGAGACTCCGAAACATTCAAAACTTTGTTGCAAGTGTTACCTATCTATAAAAAAACAAAGCTGAGGTATAATTTGATTGAA
664157_30677280_HHV-7_JI_U43400.1_144861bp_1_1123	ACTTTGTTGCAAGTGTTACCTATCTATAAAAAAACAAAGCTGAGGTATAATTTGATTGAACGCTGTTTAAATAATTGTCCCCCTCACGTTAAAGATGCATTGATTATTGAAATCATGAAA
664157_30677280_HHV-7_JI_U43400.1_144861bp_1_1124	CGCTGTTTAAATAATTGTCCCCCTCACGTTAAAGATGCATTGATTATTGAAATCATGAAAGCTAAGAAAATTTTAGAGACTCTGGATGTTGTGTTCATGAAAATTATGATTGGAGAATTT
664157_30677280_HHV-7_JI_U43400.1_144861bp_1_1125	GCTAAGAAAATTTTAGAGACTCTGGATGTTGTGTTCATGAAAATTATGATTGGAGAATTTACAATTTGCAGTGACAATGTGAATCAATTGCTCAATAAATTTTCAATAGACCAAACAACA
664157_30677280_HHV-7_JI_U43400.1_144861bp_1_1126	ACAATTTGCAGTGACAATGTGAATCAATTGCTCAATAAATTTTCAATAGACCAAACAACATTATGTGACATGGAAAAAATAAATACTTTAATTGACTTAGACGAAGAAAATAGCAAGCGT
664157_30677280_HHV-7_JI_U43400.1_144861bp_1_1127	TTATGTGACATGGAAAAAATAAATACTTTAATTGACTTAGACGAAGAAAATAGCAAGCGTCTTTTGACAGAGATCGATCCTTTGTTACATCAAGAAACAGGCCTGTATCAAGCGCTGCCT
664157_30677280_HHV-7_JI_U43400.1_144861bp_1_1128	CTTTTGACAGAGATCGATCCTTTGTTACATCAAGAAACAGGCCTGTATCAAGCGCTGCCTAATGCAGTTACGGATCCACCAAGCGAACAGAGAGCCGCAACTAAAAAATGTTACGAAGGA
664157_30677280_HHV-7_JI_U43400.1_144861bp_1_1129	AATGCAGTTACGGATCCACCAAGCGAACAGAGAGCCGCAACTAAAAAATGTTACGAAGGATTTACCAAATAAAATTTTATTCCGTGCTGTACTGAAACCAAAGCATCGATGTTTTATCTA

664157_30677280_HHV-7_JI_U43400.1_144861bp_1_1130	TTTACCAAATAAAATTTTATTCCGTGCTGTACTGAAACCAAAGCATCGATGTTTTATCTAACACTGCAAGCTGATTCAACACTCGATTAGGTAATAGCTTTAAAATAAAATGACCGCATG
664157_30677280_HHV-7_JI_U43400.1_144861bp_1_1131	ACACTGCAAGCTGATTCAACACTCGATTAGGTAATAGCTTTAAAATAAAATGACCGCATGTGAAAATGATGGTTGCAATACAAATTCCTGCTTGCACAAAGATCCTCTGAGAAGTGGGAT
664157_30677280_HHV-7_JI_U43400.1_144861bp_1_1132	TGAAAATGATGGTTGCAATACAAATTCCTGCTTGCACAAAGATCCTCTGAGAAGTGGGATTAAAAATCTTTACACTTGGAGTTGTGTTCTTTAACCAGATATTAGTTTCAACCACTAATC
664157_30677280_HHV-7_JI_U43400.1_144861bp_1_1133	TAAAAATCTTTACACTTGGAGTTGTGTTCTTTAACCAGATATTAGTTTCAACCACTAATCCATGACAGAGAATTTCTTTGTTTGCTATTATTAAAGCTTTCAATTCATCAGGACAATCAA
664157_30677280_HHV-7_JI_U43400.1_144861bp_1_1134	CATGACAGAGAATTTCTTTGTTTGCTATTATTAAAGCTTTCAATTCATCAGGACAATCAAACATGTAATCAAAATTTTGGATTGTGTAATTATTGGATTCTACATAGAAATCTTTATTGG
664157_30677280_HHV-7_JI_U43400.1_144861bp_1_1135	ACATGTAATCAAAATTTTGGATTGTGTAATTATTGGATTCTACATAGAAATCTTTATTGGTAATTAGTGAAAACTTGATAAAATTTGGAGAAGGAAAATTATAAGTTTCCCAGGGTTCCG
664157_30677280_HHV-7_JI_U43400.1_144861bp_1_1136	TAATTAGTGAAAACTTGATAAAATTTGGAGAAGGAAAATTATAAGTTTCCCAGGGTTCCGGTATATAGCATGGAAGGATGTTAATCACTAATTCTATATCACCAGGTAACAACATTACTG
664157_30677280_HHV-7_JI_U43400.1_144861bp_1_1137	GTATATAGCATGGAAGGATGTTAATCACTAATTCTATATCACCAGGTAACAACATTACTGATTCACTTGACAGGTTTATTAAAGAAATGGAGATAACGTTTTCTGTTGTGATGAGTTCTG
664157_30677280_HHV-7_JI_U43400.1_144861bp_1_1138	ATTCACTTGACAGGTTTATTAAAGAAATGGAGATAACGTTTTCTGTTGTGATGAGTTCTGTTACACATTCGCAGAGAGTGTCGTTAACTGTTCCAGTCAAAATCCCAAAAAGTCCGTCGG
664157_30677280_HHV-7_JI_U43400.1_144861bp_1_1139	TTACACATTCGCAGAGAGTGTCGTTAACTGTTCCAGTCAAAATCCCAAAAAGTCCGTCGGGAATGTTTAGTGACACATTTAAATTAAAAATGCGTACTTGTTCACAAGGCACCCATATAA
664157_30677280_HHV-7_JI_U43400.1_144861bp_1_1140	GAATGTTTAGTGACACATTTAAATTAAAAATGCGTACTTGTTCACAAGGCACCCATATAAGTTCTCTATTTACGAGTACAATTTTATTTGTATTTAGAATTACATCCATCCGACTTTTCT
664157_30677280_HHV-7_JI_U43400.1_144861bp_1_1141	GTTCTCTATTTACGAGTACAATTTTATTTGTATTTAGAATTACATCCATCCGACTTTTCTCAAAATGATTTATTAAAATTGGAAGTTCGTTTACTCGAGGAACATTTTCAAAACCAAATC

664157_30677280_HHV-7_JI_U43400.1_144861bp_1_1142	CAAAATGATTTATTAAAATTGGAAGTTCGTTTACTCGAGGAACATTTTCAAAACCAAATCTGCTAGATCTGTTTCTTGGAAAGGTGAGATATTTCTTTTCATTGTCCATATGTTCTGCAA
664157_30677280_HHV-7_JI_U43400.1_144861bp_1_1143	TGCTAGATCTGTTTCTTGGAAAGGTGAGATATTTCTTTTCATTGTCCATATGTTCTGCAAAGAACTCATCGTAAATTTTTTCACAGGATTTGAATAAACCGTTTTGAGGAATTCCAAGTA
664157_30677280_HHV-7_JI_U43400.1_144861bp_1_1144	AGAACTCATCGTAAATTTTTTCACAGGATTTGAATAAACCGTTTTGAGGAATTCCAAGTAAAGAAAATGGTCGTTGATAAACCGTCAATTCAGTGTTCACATATTCATTTAATTGCTTTG
664157_30677280_HHV-7_JI_U43400.1_144861bp_1_1145	AAGAAAATGGTCGTTGATAAACCGTCAATTCAGTGTTCACATATTCATTTAATTGCTTTGCATCGATTTTAGGCCATGTAAGAGAGCTCTCTTTTGTTTCGCTAAAGTTCCAGATATCAT
664157_30677280_HHV-7_JI_U43400.1_144861bp_1_1146	CATCGATTTTAGGCCATGTAAGAGAGCTCTCTTTTGTTTCGCTAAAGTTCCAGATATCATCCACATCCGTTACAGTAAACAGGAAAAAGTGCAAGTATCTGGACATAGGCAATTTGTTAA
664157_30677280_HHV-7_JI_U43400.1_144861bp_1_1147	CCACATCCGTTACAGTAAACAGGAAAAAGTGCAAGTATCTGGACATAGGCAATTTGTTAAGTGTAATTGTTTCAGAGATTTTTTCAGAAATTATTTTCTTGTTTGTTTCAGATCTTCTGG
664157_30677280_HHV-7_JI_U43400.1_144861bp_1_1148	GTGTAATTGTTTCAGAGATTTTTTCAGAAATTATTTTCTTGTTTGTTTCAGATCTTCTGGAGGGTAGACTTTGCATTTTCGTTGAAGCCTTCGGATGATTAGGAAAAATGACTCTATACA
664157_30677280_HHV-7_JI_U43400.1_144861bp_1_1149	AGGGTAGACTTTGCATTTTCGTTGAAGCCTTCGGATGATTAGGAAAAATGACTCTATACAAGATTGTTTCCAAGCCAATTATATTGCTGGCATTTTTTTTTACCCGAGTTGTTTTCACAA
664157_30677280_HHV-7_JI_U43400.1_144861bp_1_1150	AGATTGTTTCCAAGCCAATTATATTGCTGGCATTTTTTTTTACCCGAGTTGTTTTCACAAATGAAGTCGACGGGGAGGAGCTGTTTTATAAGCCTACTTGTCATTCAGATACATATGAAA
664157_30677280_HHV-7_JI_U43400.1_144861bp_1_1151	ATGAAGTCGACGGGGAGGAGCTGTTTTATAAGCCTACTTGTCATTCAGATACATATGAAATTATTTTAAAAAAATTTTCTTCTATTTGGATATTGGTCAACACATTTATTTTATTATGTT
664157_30677280_HHV-7_JI_U43400.1_144861bp_1_1152	TTATTTTAAAAAAATTTTCTTCTATTTGGATATTGGTCAACACATTTATTTTATTATGTTCATTTTCATTATTTTTAAAGTATTGGTGTTTTAAGACTCTCGCCAAAGAAACTGTAAAAG
664157_30677280_HHV-7_JI_U43400.1_144861bp_1_1153	CATTTTCATTATTTTTAAAGTATTGGTGTTTTAAGACTCTCGCCAAAGAAACTGTAAAAGGGTATTAAATCTATATGAAGAATGTATGAAAATGCCCTCAGAACTGTTTTAATGTATTTT

664157_30677280_HHV-7_JI_U43400.1_144861bp_1_1154	GGTATTAAATCTATATGAAGAATGTATGAAAATGCCCTCAGAACTGTTTTAATGTATTTTTCTCCATGCATATTTGAACATAAATATTCTAAGCCAAATATTTTTCTCGTGATTAGTCTG
664157_30677280_HHV-7_JI_U43400.1_144861bp_1_1155	TCTCCATGCATATTTGAACATAAATATTCTAAGCCAAATATTTTTCTCGTGATTAGTCTGCGTTACTTCTGTCACCACACTCATATTTTTCATGGTATTTGTTAGAATATCTGTGGCTTT
664157_30677280_HHV-7_JI_U43400.1_144861bp_1_1156	CGTTACTTCTGTCACCACACTCATATTTTTCATGGTATTTGTTAGAATATCTGTGGCTTTTGCTTTCCATCATCACGTCATGCTGTATGTTGAAGTTGAACAATGGTGTGTCTGTGGTTG
664157_30677280_HHV-7_JI_U43400.1_144861bp_1_1157	TGCTTTCCATCATCACGTCATGCTGTATGTTGAAGTTGAACAATGGTGTGTCTGTGGTTGAATTTGTTACGAACGCTTAATAGAAATTATTGTACATTTTTCTATTAGTATGAGTGCTAT
664157_30677280_HHV-7_JI_U43400.1_144861bp_1_1158	AATTTGTTACGAACGCTTAATAGAAATTATTGTACATTTTTCTATTAGTATGAGTGCTATGAAAATTTCCTACAAAAGTTCTGTTTTGAGTGTTAATTAAAGTTGCATTGAAAAGAATGG
664157_30677280_HHV-7_JI_U43400.1_144861bp_1_1159	GAAAATTTCCTACAAAAGTTCTGTTTTGAGTGTTAATTAAAGTTGCATTGAAAAGAATGGCGGTTGTACTGTAATTTCTCAAATCAAAGTTTTAAAGTATGCTTTGTCACGGAGATACTC
664157_30677280_HHV-7_JI_U43400.1_144861bp_1_1160	CGGTTGTACTGTAATTTCTCAAATCAAAGTTTTAAAGTATGCTTTGTCACGGAGATACTCTACACTACGAAGTTATAAGTCCGTGTTTTGCTGATTTATGTGCTTGTGTTACTATGGTTT
664157_30677280_HHV-7_JI_U43400.1_144861bp_1_1161	TACACTACGAAGTTATAAGTCCGTGTTTTGCTGATTTATGTGCTTGTGTTACTATGGTTTGTCGTGAAAAAGGTCGTAGGAAATGATGAAGTCTGTTGTGAAGCTTGCATGCTTGAGGTG
664157_30677280_HHV-7_JI_U43400.1_144861bp_1_1162	GTCGTGAAAAAGGTCGTAGGAAATGATGAAGTCTGTTGTGAAGCTTGCATGCTTGAGGTGCCTCGAGTAGATGGAAAAAATTTTGTTGGTTCGGAAGTAACAGCGTTCGGAATTGCTGTA
664157_30677280_HHV-7_JI_U43400.1_144861bp_1_1163	CCTCGAGTAGATGGAAAAAATTTTGTTGGTTCGGAAGTAACAGCGTTCGGAATTGCTGTAGGCTTAGTAACTTTGGTCTTATTTTCTAAGAACATCAAGTTCCCCTTAAAACTTTGTATT
664157_30677280_HHV-7_JI_U43400.1_144861bp_1_1164	GGCTTAGTAACTTTGGTCTTATTTTCTAAGAACATCAAGTTCCCCTTAAAACTTTGTATTGGTGACATGATTCCCCTTACCAAGGAGAAGTTGGTAACACCAAATGGTGTTGACACATTC
664157_30677280_HHV-7_JI_U43400.1_144861bp_1_1165	GGTGACATGATTCCCCTTACCAAGGAGAAGTTGGTAACACCAAATGGTGTTGACACATTCCGCGTGTTCACAGAATTATAGAAAAATTTTGAAATAGAATTCTTGCAATCTGTTCGATTG

664157_30677280_HHV-7_JI_U43400.1_144861bp_1_1166	CGCGTGTTCACAGAATTATAGAAAAATTTTGAAATAGAATTCTTGCAATCTGTTCGATTGTAAACAAAGTAGTAAATAAAATTCGTAAAAGAAAAAAGTTGCTCAGAATTTTCGCGTGAA
664157_30677280_HHV-7_JI_U43400.1_144861bp_1_1167	TAAACAAAGTAGTAAATAAAATTCGTAAAAGAAAAAAGTTGCTCAGAATTTTCGCGTGAAAATAATTGAGCTTCAGTATGGTTTTGGAGATCTTGGAAAATGGTTTCAATCTTGTTTTGG
664157_30677280_HHV-7_JI_U43400.1_144861bp_1_1168	AATAATTGAGCTTCAGTATGGTTTTGGAGATCTTGGAAAATGGTTTCAATCTTGTTTTGGCTAAAGTTAATTTTAAACAAATGTGAAGCAAGTACTTCAAACGGTATTTTATTGACACAA
664157_30677280_HHV-7_JI_U43400.1_144861bp_1_1169	CTAAAGTTAATTTTAAACAAATGTGAAGCAAGTACTTCAAACGGTATTTTATTGACACAAGAGTCATCCATCGTTTTGAATGCTAGTGTAGCAAAAACTAAATACAAAAAGGAAATATTA
664157_30677280_HHV-7_JI_U43400.1_144861bp_1_1170	GAGTCATCCATCGTTTTGAATGCTAGTGTAGCAAAAACTAAATACAAAAAGGAAATATTATCTAAGTAAACGTGACGTTTTCTGTTTGTTTTAGTGGGAAATTCAAAATGTAATGTCCAT
664157_30677280_HHV-7_JI_U43400.1_144861bp_1_1171	TCTAAGTAAACGTGACGTTTTCTGTTTGTTTTAGTGGGAAATTCAAAATGTAATGTCCATCGCGGAAAATTATCTAGATAGAGCATATTTATTTTAGAAAGGCAAATATTCTTTTTCTGG
664157_30677280_HHV-7_JI_U43400.1_144861bp_1_1172	CGCGGAAAATTATCTAGATAGAGCATATTTATTTTAGAAAGGCAAATATTCTTTTTCTGGGATAGACGGAATGACCAAAATAAAATCTGTTTAAAACAGTTCTTAGAGATGGGTATGTCG
664157_30677280_HHV-7_JI_U43400.1_144861bp_1_1173	GATAGACGGAATGACCAAAATAAAATCTGTTTAAAACAGTTCTTAGAGATGGGTATGTCGCAACTCTGAATATATGTTTTTTCAAAAGACTCATAATGAAATTTTGCGAATTTCGGCCAC
664157_30677280_HHV-7_JI_U43400.1_144861bp_1_1174	CAACTCTGAATATATGTTTTTTCAAAAGACTCATAATGAAATTTTGCGAATTTCGGCCACATGGAAAAATTCGTTAAATTTACAAACGAGATTTTTTCAGAGCAAACACTTAATACAGGA
664157_30677280_HHV-7_JI_U43400.1_144861bp_1_1175	ATGGAAAAATTCGTTAAATTTACAAACGAGATTTTTTCAGAGCAAACACTTAATACAGGAGTCCATATGATCAGATAACTATAAAAAAGAAAAGAAATCAACGTGAACACTAGCATTGAA
664157_30677280_HHV-7_JI_U43400.1_144861bp_1_1176	GTCCATATGATCAGATAACTATAAAAAAGAAAAGAAATCAACGTGAACACTAGCATTGAATACATTTTGTTTTTCATAAATGTGAATTATTTAGGAACTGTCAATATCCTAAAATAGTTA
664157_30677280_HHV-7_JI_U43400.1_144861bp_1_1177	TACATTTTGTTTTTCATAAATGTGAATTATTTAGGAACTGTCAATATCCTAAAATAGTTATTCATTTTTTTAACCAGGATCATGATTGCAAAATGTACCTTGTGTTGGACAGTATTTGTT

664157_30677280_HHV-7_JI_U43400.1_144861bp_1_1178	TTCATTTTTTTAACCAGGATCATGATTGCAAAATGTACCTTGTGTTGGACAGTATTTGTTCACGCGCTAATCATTTTAATGATACTTTTATTAATTCTTCGTTCTCTAATTGGATTTAAC
664157_30677280_HHV-7_JI_U43400.1_144861bp_1_1179	CACGCGCTAATCATTTTAATGATACTTTTATTAATTCTTCGTTCTCTAATTGGATTTAACAACGATCTTTGGTCGCAATCACAACTAGCTTTTACTTGTCTGAATAAAAGTTTATCTCAT
664157_30677280_HHV-7_JI_U43400.1_144861bp_1_1180	AACGATCTTTGGTCGCAATCACAACTAGCTTTTACTTGTCTGAATAAAAGTTTATCTCATGTTCCAGCATAATATCAAAACAATCTAAATACATGATAAATACCAAACAAAACTATAATT
664157_30677280_HHV-7_JI_U43400.1_144861bp_1_1181	GTTCCAGCATAATATCAAAACAATCTAAATACATGATAAATACCAAACAAAACTATAATTATGATAATTAAATACAGAAGAACTAACATGATTGAAACTTGACTTGATTTTAGATCTAAT
664157_30677280_HHV-7_JI_U43400.1_144861bp_1_1182	ATGATAATTAAATACAGAAGAACTAACATGATTGAAACTTGACTTGATTTTAGATCTAATGCGGTAATTTCGAAAACAGAGCCATTTCTTAAAAACAATAAATAATGTGTTCGTGGATTC
664157_30677280_HHV-7_JI_U43400.1_144861bp_1_1183	GCGGTAATTTCGAAAACAGAGCCATTTCTTAAAAACAATAAATAATGTGTTCGTGGATTCACATCGATAAAATTTGTGTCCGGATCTATTAATTTTAATAGTTGCTTGTCATCCATTATG
664157_30677280_HHV-7_JI_U43400.1_144861bp_1_1184	ACATCGATAAAATTTGTGTCCGGATCTATTAATTTTAATAGTTGCTTGTCATCCATTATGTATACAAATTGGATAATTCCGTCTATGTCGTCATATTCCACAACCAAGCTTTCACAAAAC
664157_30677280_HHV-7_JI_U43400.1_144861bp_1_1185	TATACAAATTGGATAATTCCGTCTATGTCGTCATATTCCACAACCAAGCTTTCACAAAACACACAATCGTGGGATGAAATATTATAGATTGGTTTTATATTTTTCACCGAATATTTATAA
664157_30677280_HHV-7_JI_U43400.1_144861bp_1_1186	ACACAATCGTGGGATGAAATATTATAGATTGGTTTTATATTTTTCACCGAATATTTATAATTAGTAGGTGTGCAGGTAGAGTTTAAGGGAACAACAGTGATAATAATTGTAGTGCTTATG
664157_30677280_HHV-7_JI_U43400.1_144861bp_1_1187	TTAGTAGGTGTGCAGGTAGAGTTTAAGGGAACAACAGTGATAATAATTGTAGTGCTTATGACAGTACTCGTGACACTGAAACTAGTACCGGCTAAAATATATTTTGAAGAGATGGTGTAA
664157_30677280_HHV-7_JI_U43400.1_144861bp_1_1188	ACAGTACTCGTGACACTGAAACTAGTACCGGCTAAAATATATTTTGAAGAGATGGTGTAAGTAATATTTGGTAATGTGAGGAAGGCAGCTTGTTCATTTGAAACACAGGAAATGCTTTTA
664157_30677280_HHV-7_JI_U43400.1_144861bp_1_1189	GTAATATTTGGTAATGTGAGGAAGGCAGCTTGTTCATTTGAAACACAGGAAATGCTTTTAAACATATCTAATCTCTTTTTTCTATATACAGAAAGTGTGTTCATCACCTTGTTTGTGTAT

664157_30677280_HHV-7_JI_U43400.1_144861bp_1_1190	AACATATCTAATCTCTTTTTTCTATATACAGAAAGTGTGTTCATCACCTTGTTTGTGTATGCGTCTAGGTTTTTATAGGATAAAATGTTGTTGACAACTCTTTGAGTTAAATCTTTGCGC
664157_30677280_HHV-7_JI_U43400.1_144861bp_1_1191	GCGTCTAGGTTTTTATAGGATAAAATGTTGTTGACAACTCTTTGAGTTAAATCTTTGCGCGTGGCTGTTGCACAAGGAGAAAACATCGTTTCAATGTCCATGACATTATCAAGCATATGT
664157_30677280_HHV-7_JI_U43400.1_144861bp_1_1192	GTGGCTGTTGCACAAGGAGAAAACATCGTTTCAATGTCCATGACATTATCAAGCATATGTGTCCAAAAATACATTTCCAAAGGATTGCACAGACTTGCGAGCAAAACATGAATCTTTCGC
664157_30677280_HHV-7_JI_U43400.1_144861bp_1_1193	GTCCAAAAATACATTTCCAAAGGATTGCACAGACTTGCGAGCAAAACATGAATCTTTCGCTTGATCTTTTCTTGGAAGACTAGACTGTTTCCAATTTCTGAGAGTAAAAACAAATTTGCA
664157_30677280_HHV-7_JI_U43400.1_144861bp_1_1194	TTGATCTTTTCTTGGAAGACTAGACTGTTTCCAATTTCTGAGAGTAAAAACAAATTTGCACGTTCCGGTTGGGTTAAATTTGTAAACAATTTATGTTTTTCATATAATGTTTCAATATTG
664157_30677280_HHV-7_JI_U43400.1_144861bp_1_1195	CGTTCCGGTTGGGTTAAATTTGTAAACAATTTATGTTTTTCATATAATGTTTCAATATTGTTTTCTATTTCTTTCCATTTGTTTTGTGGAACTGTAACGATTCTTCCATCTGCTAAAGAT
664157_30677280_HHV-7_JI_U43400.1_144861bp_1_1196	TTTTCTATTTCTTTCCATTTGTTTTGTGGAACTGTAACGATTCTTCCATCTGCTAAAGATAAAAATGCTAGCTTAGCAGAGATAGGAATTGTCAGATCAGATGTAATATAAGAATAATCT
664157_30677280_HHV-7_JI_U43400.1_144861bp_1_1197	AAAAATGCTAGCTTAGCAGAGATAGGAATTGTCAGATCAGATGTAATATAAGAATAATCTACTAACTGGAAATTCTGAAGTTTTTCCATCTCAGGATATAATAAATGACTCTTAGTCATT
664157_30677280_HHV-7_JI_U43400.1_144861bp_1_1198	ACTAACTGGAAATTCTGAAGTTTTTCCATCTCAGGATATAATAAATGACTCTTAGTCATTTTGAAGCACTTTTCTAAAAACCTACCCATCGTAATGATGCTTTGGTGAGTATTAAGGATT
664157_30677280_HHV-7_JI_U43400.1_144861bp_1_1199	TTGAAGCACTTTTCTAAAAACCTACCCATCGTAATGATGCTTTGGTGAGTATTAAGGATTTCCAACAGAGGGAGGAGTCCATCTCCTCGAGTACTATATAGAAAATGAGTTAAGCCGAAT
664157_30677280_HHV-7_JI_U43400.1_144861bp_1_1200	TCCAACAGAGGGAGGAGTCCATCTCCTCGAGTACTATATAGAAAATGAGTTAAGCCGAATAAAAACATTAAATGGAATGTGTCTTTGTTAACTGGTTTTCCGCAAAATCCCTCCATGATA
664157_30677280_HHV-7_JI_U43400.1_144861bp_1_1201	AAAAACATTAAATGGAATGTGTCTTTGTTAACTGGTTTTCCGCAAAATCCCTCCATGATATAAACAGTTGCTAACTTATTGAATTTGGCTATTAAAAACGAGATGTCATAATTTTGTTTC

664157_30677280_HHV-7_JI_U43400.1_144861bp_1_1202	TAAACAGTTGCTAACTTATTGAATTTGGCTATTAAAAACGAGATGTCATAATTTTGTTTCCAAATATCATCCAGAAAGTCTATTTTGAGAAACGGATAAAATCTTTCAATTGTTGTTTTT
664157_30677280_HHV-7_JI_U43400.1_144861bp_1_1203	CAAATATCATCCAGAAAGTCTATTTTGAGAAACGGATAAAATCTTTCAATTGTTGTTTTTTTTGTAATTAACAGGAGATCGTCTGAGACAGTCTGTCTATATATGAATGAACTTTTTCGA
664157_30677280_HHV-7_JI_U43400.1_144861bp_1_1204	TTTGTAATTAACAGGAGATCGTCTGAGACAGTCTGTCTATATATGAATGAACTTTTTCGAAAAGGTGCTTTAAAAAAAATACGACTAACATTTCCAAAGATAAAAACGACATCATCCATT
664157_30677280_HHV-7_JI_U43400.1_144861bp_1_1205	AAAGGTGCTTTAAAAAAAATACGACTAACATTTCCAAAGATAAAAACGACATCATCCATTCCTGTTTCCACTGAAATTATAAAAAACGATGGTGTCAAAGCTAGCATGAACACAGTATTT
664157_30677280_HHV-7_JI_U43400.1_144861bp_1_1206	CCTGTTTCCACTGAAATTATAAAAAACGATGGTGTCAAAGCTAGCATGAACACAGTATTTAAGTAATTATAATGTCTCATAACACATCTTTCTGTTATTGGGAATAGATAATCAGTCAAG
664157_30677280_HHV-7_JI_U43400.1_144861bp_1_1207	AAGTAATTATAATGTCTCATAACACATCTTTCTGTTATTGGGAATAGATAATCAGTCAAGCCATTTGAAAGTGTGCAAATGTATTGGTTGTAATAGAGTTTGCGAATTTCCGAAACATTT
664157_30677280_HHV-7_JI_U43400.1_144861bp_1_1208	CCATTTGAAAGTGTGCAAATGTATTGGTTGTAATAGAGTTTGCGAATTTCCGAAACATTTGCAAAGTAGACATCCTTTTCATAAAATAAAGGTAAGAAAGACACTGTAATCGTTTTTGTA
664157_30677280_HHV-7_JI_U43400.1_144861bp_1_1209	GCAAAGTAGACATCCTTTTCATAAAATAAAGGTAAGAAAGACACTGTAATCGTTTTTGTATGCGAAACAGGTTTGATATTTGTCTGAAATTTTTGTGCATAGATTTTCACAGCTTTCTCT
664157_30677280_HHV-7_JI_U43400.1_144861bp_1_1210	TGCGAAACAGGTTTGATATTTGTCTGAAATTTTTGTGCATAGATTTTCACAGCTTTCTCTGTTGATGGGTTAAAAAAACGTGTAATCCGATGTTTATATTGATCAAATGATTCATCGAAA
664157_30677280_HHV-7_JI_U43400.1_144861bp_1_1211	GTTGATGGGTTAAAAAAACGTGTAATCCGATGTTTATATTGATCAAATGATTCATCGAAATTCACAGAATCAAATAAATTCGAGACAAAAGTATATCCGAATAAGCATTTGGGAATCTGA
664157_30677280_HHV-7_JI_U43400.1_144861bp_1_1212	TTCACAGAATCAAATAAATTCGAGACAAAAGTATATCCGAATAAGCATTTGGGAATCTGATAAACCCGAGTTTCGTTATAATCATGGAAATTAAACGTTATTAAACCAGGTTGAATAATA
664157_30677280_HHV-7_JI_U43400.1_144861bp_1_1213	TAAACCCGAGTTTCGTTATAATCATGGAAATTAAACGTTATTAAACCAGGTTGAATAATAGTTTGATTTGTTTTTTCATTTACGCAAATTGAAGAATTGAGTATATTCCAGTGTTTCCAT

664157_30677280_HHV-7_JI_U43400.1_144861bp_1_1214	GTTTGATTTGTTTTTTCATTTACGCAAATTGAAGAATTGAGTATATTCCAGTGTTTCCATCCATTTATTGACACGATGAGAAGTAGACTATTTATGTAAAAATACATTTTCTCCAATTTA
664157_30677280_HHV-7_JI_U43400.1_144861bp_1_1215	CCATTTATTGACACGATGAGAAGTAGACTATTTATGTAAAAATACATTTTCTCCAATTTAGAGCCTGGAAGATAAACGGGAAGTTTGTTGTAGGCTACGCCTTAAACTTTACATATTAAA
664157_30677280_HHV-7_JI_U43400.1_144861bp_1_1216	GAGCCTGGAAGATAAACGGGAAGTTTGTTGTAGGCTACGCCTTAAACTTTACATATTAAATACATAACGGGATAGGAATCATAGAGATATTTTGGGTAATATGTCTTTAGAATACTTGCC
664157_30677280_HHV-7_JI_U43400.1_144861bp_1_1217	TACATAACGGGATAGGAATCATAGAGATATTTTGGGTAATATGTCTTTAGAATACTTGCCCCCTGTTAGAAGACGTATTGGTCAATATAATCATTTGAGAATCTACAAAAAAATTCTGTT
664157_30677280_HHV-7_JI_U43400.1_144861bp_1_1218	CCCTGTTAGAAGACGTATTGGTCAATATAATCATTTGAGAATCTACAAAAAAATTCTGTTGTTAAAATCCAATTTTGAAAAATTAAATTTTTTTTTGGGCAATCTTTTTCCAGAAGAACT
664157_30677280_HHV-7_JI_U43400.1_144861bp_1_1219	GTTAAAATCCAATTTTGAAAAATTAAATTTTTTTTTGGGCAATCTTTTTCCAGAAGAACTGCATGATTCAAAAATACATGTATATTTTGAAGTTAGATTAGGGTGCCGTATTCCGGATTG
664157_30677280_HHV-7_JI_U43400.1_144861bp_1_1220	GCATGATTCAAAAATACATGTATATTTTGAAGTTAGATTAGGGTGCCGTATTCCGGATTGCATTATTGTTTTTAGGCATTTTGGTGAGAAATTATTAAAAACTTTCCATTGTTATTTTTT
664157_30677280_HHV-7_JI_U43400.1_144861bp_1_1221	CATTATTGTTTTTAGGCATTTTGGTGAGAAATTATTAAAAACTTTCCATTGTTATTTTTTGAATTCAAAACTACTTTTGCCAAATCAAATCTGTTTTCAATTCAAAAAAATAGGACACA
664157_30677280_HHV-7_JI_U43400.1_144861bp_1_1222	TGAATTCAAAACTACTTTTGCCAAATCAAATCTGTTTTCAATTCAAAAAAATAGGACACAAAAAATTCAATATTTACAGGGACTAAGGCAACTACGACAAGCAACAGATTATCTACAGCA
664157_30677280_HHV-7_JI_U43400.1_144861bp_1_1223	AAAAATTCAATATTTACAGGGACTAAGGCAACTACGACAAGCAACAGATTATCTACAGCAATTTGTCATCAAAAATGAAAGCTTATGTAAGGTAAACCCTGTAATCTGCTTTTTTAGACA
664157_30677280_HHV-7_JI_U43400.1_144861bp_1_1224	ATTTGTCATCAAAAATGAAAGCTTATGTAAGGTAAACCCTGTAATCTGCTTTTTTAGACAGCATGGTTTAAAATTGGACTTTGTGAAAACTTTCATAGCTAAAGAATTGCAACTGTCTTC
664157_30677280_HHV-7_JI_U43400.1_144861bp_1_1225	GCATGGTTTAAAATTGGACTTTGTGAAAACTTTCATAGCTAAAGAATTGCAACTGTCTTCCACGTTTCTTTGCAACCTTTTTACGAAATATCAAAATGACACAGTTAAGTCTATTTTATC

664157_30677280_HHV-7_JI_U43400.1_144861bp_1_1226	CACGTTTCTTTGCAACCTTTTTACGAAATATCAAAATGACACAGTTAAGTCTATTTTATCAATTTCCAATCCAACCAATTTTCGAAGGGCATGTCAGAAATACTCTAATTTGTACCGAGG
664157_30677280_HHV-7_JI_U43400.1_144861bp_1_1227	AATTTCCAATCCAACCAATTTTCGAAGGGCATGTCAGAAATACTCTAATTTGTACCGAGGAAGATATGCAACAACTCCAAAACTTGGGAATTCGAAAACTTCGAAAAGAAAAAGAAGAAA
664157_30677280_HHV-7_JI_U43400.1_144861bp_1_1228	AAGATATGCAACAACTCCAAAACTTGGGAATTCGAAAACTTCGAAAAGAAAAAGAAGAAATTCAAAAAAACAAGATTTTAAAAAACTTGTTAAAAACTGAACTTGATGTTTTGCAAGCAC
664157_30677280_HHV-7_JI_U43400.1_144861bp_1_1229	TTCAAAAAAACAAGATTTTAAAAAACTTGTTAAAAACTGAACTTGATGTTTTGCAAGCACATGTACAGACAGAATGCCAGAAATTAAATACAAATTTAAGAGACATTGAAAATGCATTGC
664157_30677280_HHV-7_JI_U43400.1_144861bp_1_1230	ATGTACAGACAGAATGCCAGAAATTAAATACAAATTTAAGAGACATTGAAAATGCATTGCTTTTAGAAAATCAAAAAATAATTCCTTCTAGTGAAACACATTCTGTCTTGGAAGAGTCTC
664157_30677280_HHV-7_JI_U43400.1_144861bp_1_1231	TTTTAGAAAATCAAAAAATAATTCCTTCTAGTGAAACACATTCTGTCTTGGAAGAGTCTCTCCAGGCTAAAACTGTCACGCAAGTCACAATTACACAAATTGATCCTGCTATTCATTTCA
664157_30677280_HHV-7_JI_U43400.1_144861bp_1_1232	TCCAGGCTAAAACTGTCACGCAAGTCACAATTACACAAATTGATCCTGCTATTCATTTCACGGAAAATTTCCGACCTGAGATGATAAAAACTTTTTATAATAATACTCAGATGTGGAGCT
664157_30677280_HHV-7_JI_U43400.1_144861bp_1_1233	CGGAAAATTTCCGACCTGAGATGATAAAAACTTTTTATAATAATACTCAGATGTGGAGCTATACGTTTGGAGCTTGGTTTTACAAATTAAAGCGAGCTTTCTTTACCGATTCTAAATTAA
664157_30677280_HHV-7_JI_U43400.1_144861bp_1_1234	ATACGTTTGGAGCTTGGTTTTACAAATTAAAGCGAGCTTTCTTTACCGATTCTAAATTAAAAAGAATGCTAAAGTTAACATATGTTGATTCTCTCTCTATTACACAGGAATTGCTGTCTA
664157_30677280_HHV-7_JI_U43400.1_144861bp_1_1235	AAAGAATGCTAAAGTTAACATATGTTGATTCTCTCTCTATTACACAGGAATTGCTGTCTATTTCAATTAATGCATTGGAACAAATTACTATTTATCCTATGCATGATAATTTAGTGTCCG
664157_30677280_HHV-7_JI_U43400.1_144861bp_1_1236	TTTCAATTAATGCATTGGAACAAATTACTATTTATCCTATGCATGATAATTTAGTGTCCGATTTAGAAGCTGGTTTGTGTCTACTGACTGCTTTTTTTGCATCGTATCCTGGAACTTTCT
664157_30677280_HHV-7_JI_U43400.1_144861bp_1_1237	ATTTAGAAGCTGGTTTGTGTCTACTGACTGCTTTTTTTGCATCGTATCCTGGAACTTTCTTAACCGAAAATATTAAATTCGTGGATGTCATTCAAAATTTGTCTCAAATTTTTAGATATC

664157_30677280_HHV-7_JI_U43400.1_144861bp_1_1238	TAACCGAAAATATTAAATTCGTGGATGTCATTCAAAATTTGTCTCAAATTTTTAGATATCTAAACACCGAAATTTTAGCTACGAAAAACGCGTCACCACAGGATTTTTATTTTGGATTCA
664157_30677280_HHV-7_JI_U43400.1_144861bp_1_1239	TAAACACCGAAATTTTAGCTACGAAAAACGCGTCACCACAGGATTTTTATTTTGGATTCAATGATCCAGACAAAATGAAATATTTTATTCCGTTGTGTAAAGGGAGGCATTATGCAATAA
664157_30677280_HHV-7_JI_U43400.1_144861bp_1_1240	ATGATCCAGACAAAATGAAATATTTTATTCCGTTGTGTAAAGGGAGGCATTATGCAATAAATACATTCAGTAATCATATCTTAATTAAAATTTTTATAAAAAAAGGTGTGATAAAGCAAG
664157_30677280_HHV-7_JI_U43400.1_144861bp_1_1241	ATACATTCAGTAATCATATCTTAATTAAAATTTTTATAAAAAAAGGTGTGATAAAGCAAGTTCCCGGAGATCAAATGTCGAAAGGACATGTTGTAATAGAATCGAAGTTGACAGGCACGT
664157_30677280_HHV-7_JI_U43400.1_144861bp_1_1242	TTCCCGGAGATCAAATGTCGAAAGGACATGTTGTAATAGAATCGAAGTTGACAGGCACGTTAACAGATGATAAATTATTATATTGGACTCAAATTTTATTGCAACCGAAATTGGGAAAAG
664157_30677280_HHV-7_JI_U43400.1_144861bp_1_1243	TAACAGATGATAAATTATTATATTGGACTCAAATTTTATTGCAACCGAAATTGGGAAAAGAGGTGCCGATATTTGTCCATCAGCAGCAGTATTTGCGTTCTGGAATTGTTGCCATAGAAT
664157_30677280_HHV-7_JI_U43400.1_144861bp_1_1244	AGGTGCCGATATTTGTCCATCAGCAGCAGTATTTGCGTTCTGGAATTGTTGCCATAGAATCCTTATATTTACTTTGGCAAATTCTAAACTCAGAAAGCATTTTTGGAAAGAGAACTGGGA
664157_30677280_HHV-7_JI_U43400.1_144861bp_1_1245	CCTTATATTTACTTTGGCAAATTCTAAACTCAGAAAGCATTTTTGGAAAGAGAACTGGGAAATTTTATCTAACAACAATTTTCCCCCATGTAAATGCAGAAGATGTTACAGAAACAGAAT
664157_30677280_HHV-7_JI_U43400.1_144861bp_1_1246	AATTTTATCTAACAACAATTTTCCCCCATGTAAATGCAGAAGATGTTACAGAAACAGAATTTTCTTCTGTCAATATCCAGAATTTTGAGTTTCTCATGAAAAACTATGTCGTCCCCACAT
664157_30677280_HHV-7_JI_U43400.1_144861bp_1_1247	TTTCTTCTGTCAATATCCAGAATTTTGAGTTTCTCATGAAAAACTATGTCGTCCCCACATATTTAGCCAATAATGAAAGTACTATTTCCACTTTATTTCCGGGGTTAATCAGCATCGTAG
664157_30677280_HHV-7_JI_U43400.1_144861bp_1_1248	ATTTAGCCAATAATGAAAGTACTATTTCCACTTTATTTCCGGGGTTAATCAGCATCGTAGTTAACGAAAGTGTTCGTTTAGGCTGGGATCATAATCAAAATACTTTAACACAAACTAATG
664157_30677280_HHV-7_JI_U43400.1_144861bp_1_1249	TTAACGAAAGTGTTCGTTTAGGCTGGGATCATAATCAAAATACTTTAACACAAACTAATGCTTTGCATAGCCAAACAAAAGACAATCCGTTTGTTGAATATATCCGTTCACAATTAGAAG

664157_30677280_HHV-7_JI_U43400.1_144861bp_1_1250	CTTTGCATAGCCAAACAAAAGACAATCCGTTTGTTGAATATATCCGTTCACAATTAGAAGAAACGGCAGAACTTGCGGTACTCGAGAAACATGATAAAATTCTTTTTCATTTTGAAAATG
664157_30677280_HHV-7_JI_U43400.1_144861bp_1_1251	AAACGGCAGAACTTGCGGTACTCGAGAAACATGATAAAATTCTTTTTCATTTTGAAAATGGCTTGAATGTGACACTCTCATTAGCACTTCCACGACATAGGTTGTTCGCTATGGCATCCT
664157_30677280_HHV-7_JI_U43400.1_144861bp_1_1252	GCTTGAATGTGACACTCTCATTAGCACTTCCACGACATAGGTTGTTCGCTATGGCATCCTCATTATTTAATGTGGCTGATTTGTATGATTTCTTGTATTTTTTGGTATTAGGTTTTATTC
664157_30677280_HHV-7_JI_U43400.1_144861bp_1_1253	CATTATTTAATGTGGCTGATTTGTATGATTTCTTGTATTTTTTGGTATTAGGTTTTATTCCAGTAGCAACTGTTATTTGAAGACCAGGTGACGTCTAGACATTTGTTTTGGATATAAATA
664157_30677280_HHV-7_JI_U43400.1_144861bp_1_1254	CAGTAGCAACTGTTATTTGAAGACCAGGTGACGTCTAGACATTTGTTTTGGATATAAATACGCGTGGAAGTGTTAAGGAGCTTCATTTTAAAAAAGACACCTTTCTTAAGCTTTTGTAAA
664157_30677280_HHV-7_JI_U43400.1_144861bp_1_1255	CGCGTGGAAGTGTTAAGGAGCTTCATTTTAAAAAAGACACCTTTCTTAAGCTTTTGTAAACATGAAAAATATCGATTTAACAAATTGGAAATTACTGGCAGAAATATATGAATATCTGTT
664157_30677280_HHV-7_JI_U43400.1_144861bp_1_1256	CATGAAAAATATCGATTTAACAAATTGGAAATTACTGGCAGAAATATATGAATATCTGTTTTTTTTCTCGTTTTTCTTTCTTTGTCTGCTGGTAATAATAGTGGTGAAATTTAATAACAG
664157_30677280_HHV-7_JI_U43400.1_144861bp_1_1257	TTTTTTCTCGTTTTTCTTTCTTTGTCTGCTGGTAATAATAGTGGTGAAATTTAATAACAGCACCGTTGGTCGAGAATACACATTTAGTACTTTTTCTGGAATGTTGGTTTATATTTTATT
664157_30677280_HHV-7_JI_U43400.1_144861bp_1_1258	CACCGTTGGTCGAGAATACACATTTAGTACTTTTTCTGGAATGTTGGTTTATATTTTATTGTTACCGGTTAAAATGGGAATGTTAACCAAAATGTGGGATGTATCCACCGATTACTGTAT
664157_30677280_HHV-7_JI_U43400.1_144861bp_1_1259	GTTACCGGTTAAAATGGGAATGTTAACCAAAATGTGGGATGTATCCACCGATTACTGTATAATCTTAATGTTTCTGAGTGATTTTTCATTTATCTTTTCGTCCTGGGCGTTAACACTGTT
664157_30677280_HHV-7_JI_U43400.1_144861bp_1_1260	AATCTTAATGTTTCTGAGTGATTTTTCATTTATCTTTTCGTCCTGGGCGTTAACACTGTTGGCACTGGAACGTATCAACAATTTTTCTTTTTCTGAGATTAAGGTAAACGAAACTAAAAT
664157_30677280_HHV-7_JI_U43400.1_144861bp_1_1261	GGCACTGGAACGTATCAACAATTTTTCTTTTTCTGAGATTAAGGTAAACGAAACTAAAATTCTAAAACAAATGTCTTTCCCAATTATCTGGGTTACTTCCATCTTTCAAGCTGTACAAAT

664157_30677280_HHV-7_JI_U43400.1_144861bp_1_1262	TCTAAAACAAATGTCTTTCCCAATTATCTGGGTTACTTCCATCTTTCAAGCTGTACAAATTTCAATGAAATATAAGAAATCGCAAATGAATTTAGAAGATGATTACTGTTTATTGGCTAT
664157_30677280_HHV-7_JI_U43400.1_144861bp_1_1263	TTCAATGAAATATAAGAAATCGCAAATGAATTTAGAAGATGATTACTGTTTATTGGCTATTGAACGCTCTGCAGAAGAAGCCTGGATCTTATTAATGTATACCGTTGTCATCCCAACTTT
664157_30677280_HHV-7_JI_U43400.1_144861bp_1_1264	TGAACGCTCTGCAGAAGAAGCCTGGATCTTATTAATGTATACCGTTGTCATCCCAACTTTCATTGTTTTTTTCTACGTGTTAAATAAGCGATTTCTCTTTTTAGAAAGAGACCTGAACTC
664157_30677280_HHV-7_JI_U43400.1_144861bp_1_1265	CATTGTTTTTTTCTACGTGTTAAATAAGCGATTTCTCTTTTTAGAAAGAGACCTGAACTCAATTGTTACACATTTAAGCTTGTTTTTATTTTTTGGTGCTTTATGCTTTTTCCCTGCTTC
664157_30677280_HHV-7_JI_U43400.1_144861bp_1_1266	AATTGTTACACATTTAAGCTTGTTTTTATTTTTTGGTGCTTTATGCTTTTTCCCTGCTTCGGTGCTTAACGAATTCAACTGCAATCGATTGTTTTACGGTCTGCATGAATTGCTCATTGT
664157_30677280_HHV-7_JI_U43400.1_144861bp_1_1267	GGTGCTTAACGAATTCAACTGCAATCGATTGTTTTACGGTCTGCATGAATTGCTCATTGTTTGTTTAGAGCTAAAAATTTTTTATGTTCCCACTATGACATATATAATAAGCTGTGAGAA
664157_30677280_HHV-7_JI_U43400.1_144861bp_1_1268	TTGTTTAGAGCTAAAAATTTTTTATGTTCCCACTATGACATATATAATAAGCTGTGAGAATTATAGGCTAGCTGCCAAAGCTTTTTTTTGTAAATGTTTTAAACCTTGTTTCTTGATGCC
664157_30677280_HHV-7_JI_U43400.1_144861bp_1_1269	TTATAGGCTAGCTGCCAAAGCTTTTTTTTGTAAATGTTTTAAACCTTGTTTCTTGATGCCTTCGTTAAGAAAATTGCAACAACCTACAAAATCTACACAGTTTTAAAATTGTAGAGGTCC
664157_30677280_HHV-7_JI_U43400.1_144861bp_1_1270	TTCGTTAAGAAAATTGCAACAACCTACAAAATCTACACAGTTTTAAAATTGTAGAGGTCCAATAGAAAACAAATCTGATAAATTACACTCTGTTACTTTTGCTTCTTTCTCCACTATGCC
664157_30677280_HHV-7_JI_U43400.1_144861bp_1_1271	AATAGAAAACAAATCTGATAAATTACACTCTGTTACTTTTGCTTCTTTCTCCACTATGCCACAAAAAACTTTGAAATCATTCTGAATAAAAGTAAATAATCCATTCCTGTGTTCTGGTTT
664157_30677280_HHV-7_JI_U43400.1_144861bp_1_1272	ACAAAAAACTTTGAAATCATTCTGAATAAAAGTAAATAATCCATTCCTGTGTTCTGGTTTCTGTTGACTAAAAGCACCGTGAGAAATTTTGATCTTGTCAAAAAAAAATTAAATAACCAAA
664157_30677280_HHV-7_JI_U43400.1_144861bp_1_1273	CTGTTGACTAAAAGCACCGTGAGAAATTTTGATCTTGTCAAAAAAAAATTAAATAACCAAACCACAGATGCTTACAAATTTTTCGGATAAAAGGTTCATATACTTCTTGCCCCCAGAACTT

664157_30677280_HHV-7_JI_U43400.1_144861bp_1_1274	CCACAGATGCTTACAAATTTTTCGGATAAAAGGTTCATATACTTCTTGCCCCCAGAACTTGTTGAAATAGCGACATAATTTTAATAAACGGTGATTGTAGATGGCACCAATAGAAAACAT
664157_30677280_HHV-7_JI_U43400.1_144861bp_1_1275	GTTGAAATAGCGACATAATTTTAATAAACGGTGATTGTAGATGGCACCAATAGAAAACATTATATTCCCATCGTGAATAATTTCAGCTGGAATTGTAATTGAAAGACCAAATTCATCCAA
664157_30677280_HHV-7_JI_U43400.1_144861bp_1_1276	TATATTCCCATCGTGAATAATTTCAGCTGGAATTGTAATTGAAAGACCAAATTCATCCAAAAAGAAACTTTCTATGATTTGTTTAACAATTCCAGTATGATGTAATTTTTTCGAGAGCTG
664157_30677280_HHV-7_JI_U43400.1_144861bp_1_1277	AAAGAAACTTTCTATGATTTGTTTAACAATTCCAGTATGATGTAATTTTTTCGAGAGCTGAGCTGCATTTTCTTCACTGAAGATATGTTTAAGGATTTCTGATCCAGATCTTTGTTTAAT
664157_30677280_HHV-7_JI_U43400.1_144861bp_1_1278	AGCTGCATTTTCTTCACTGAAGATATGTTTAAGGATTTCTGATCCAGATCTTTGTTTAATCATGCAAAAGATCTTAAACCAAAATTCATCAGGTACCTTTCGATTCAGAACCACTAAATC
664157_30677280_HHV-7_JI_U43400.1_144861bp_1_1279	CATGCAAAAGATCTTAAACCAAAATTCATCAGGTACCTTTCGATTCAGAACCACTAAATCACTCACTCCGATATTGTGCAAAGTCTCTCTCAACAGTAAGATTTGGATGCCGTGAGAAAA
664157_30677280_HHV-7_JI_U43400.1_144861bp_1_1280	ACTCACTCCGATATTGTGCAAAGTCTCTCTCAACAGTAAGATTTGGATGCCGTGAGAAAACATTAGGGTACACACATTCTGGATGATCAAAATCTCTTCTTGTTTTAGATTAAATAAAGA
664157_30677280_HHV-7_JI_U43400.1_144861bp_1_1281	CATTAGGGTACACACATTCTGGATGATCAAAATCTCTTCTTGTTTTAGATTAAATAAAGATTCACCGCTTACCAATTTCTTAGTGATATGTAATATTAAATTGGAGGTTGAGTTGTTCGA
664157_30677280_HHV-7_JI_U43400.1_144861bp_1_1282	TTCACCGCTTACCAATTTCTTAGTGATATGTAATATTAAATTGGAGGTTGAGTTGTTCGAGATTATGTATTGACCTACTTGTGTCATCGTAATAATGGAAACTGTGCTAGTGGCAGGCTT
664157_30677280_HHV-7_JI_U43400.1_144861bp_1_1283	GATTATGTATTGACCTACTTGTGTCATCGTAATAATGGAAACTGTGCTAGTGGCAGGCTTCCTATGTGTGTATGATGATAATGATATAAATGATAACTTTTATCTGCCAAGGAGGACGAT
664157_30677280_HHV-7_JI_U43400.1_144861bp_1_1284	CCTATGTGTGTATGATGATAATGATATAAATGATAACTTTTATCTGCCAAGGAGGACGATACAAGAAGAAATCAATTCCGGAAATGGTTTGAATATTCCATTGAATATAAACCACAATGA
664157_30677280_HHV-7_JI_U43400.1_144861bp_1_1285	ACAAGAAGAAATCAATTCCGGAAATGGTTTGAATATTCCATTGAATATAAACCACAATGAAAATGCCGTTATAGGCACAGTCTCTTCTTTAAGTGTTTACAGCACGGTTTGTTTCGTTGC

664157_30677280_HHV-7_JI_U43400.1_144861bp_1_1286	AAATGCCGTTATAGGCACAGTCTCTTCTTTAAGTGTTTACAGCACGGTTTGTTTCGTTGCCCGTGTTCAATCAAAGGAATTCCTTACAATAATTAAGAAAATAGCTGCAAAATCTAAGCT
664157_30677280_HHV-7_JI_U43400.1_144861bp_1_1287	CCGTGTTCAATCAAAGGAATTCCTTACAATAATTAAGAAAATAGCTGCAAAATCTAAGCTGATAACCAACACGGAAGAAAAAACTCTGCCACCAGATCCCGAAATAGAGTGTTTAAATTC
664157_30677280_HHV-7_JI_U43400.1_144861bp_1_1288	GATAACCAACACGGAAGAAAAAACTCTGCCACCAGATCCCGAAATAGAGTGTTTAAATTCAATTTTCCCAGGTTTGTCATTATCGAACAGGGTTGGTGGCAATGAACGTGATCCGTTTTT
664157_30677280_HHV-7_JI_U43400.1_144861bp_1_1289	AATTTTCCCAGGTTTGTCATTATCGAACAGGGTTGGTGGCAATGAACGTGATCCGTTTTTAAACATGTCTCTATCTGTGGCGTTGGTCGTCGACCTGGAACAATAGCTATTTTTGGACG
664157_30677280_HHV-7_JI_U43400.1_144861bp_1_1290	TAAACATGTCTCTATCTGTGGCGTTGGTCGTCGACCTGGAACAATAGCTATTTTTGGACGAAATTTAAATTGGATTCTGGATAGATTTTCTTCTATTACAGAAGCGGAAAAAGAAAAGAT
664157_30677280_HHV-7_JI_U43400.1_144861bp_1_1291	AAATTTAAATTGGATTCTGGATAGATTTTCTTCTATTACAGAAGCGGAAAAAGAAAAGATTCTGAGCACAGATCAAAGCTGTGTTCAGTTTTTTGCCGAGGAACAATTTAAAGTGGATTT
664157_30677280_HHV-7_JI_U43400.1_144861bp_1_1292	TCTGAGCACAGATCAAAGCTGTGTTCAGTTTTTTGCCGAGGAACAATTTAAAGTGGATTTATATGATCTGCTAGCGGATAGTTTAGACACTTCGTACATAAAAGTACGTTTTCCAAAATT
664157_30677280_HHV-7_JI_U43400.1_144861bp_1_1293	ATATGATCTGCTAGCGGATAGTTTAGACACTTCGTACATAAAAGTACGTTTTCCAAAATTACAGTCTGATAAGCAACTTAGTGGAATTTCAAAATCTACTTATATTAAAGCGAGTGAAAA
664157_30677280_HHV-7_JI_U43400.1_144861bp_1_1294	ACAGTCTGATAAGCAACTTAGTGGAATTTCAAAATCTACTTATATTAAAGCGAGTGAAAATTTAACGGCTAACAACCATACTATTAACGTGAATTCAAAAGTCACAAAGGAGACAGAAGC
664157_30677280_HHV-7_JI_U43400.1_144861bp_1_1295	TTTAACGGCTAACAACCATACTATTAACGTGAATTCAAAAGTCACAAAGGAGACAGAAGCAACAGACAGCGTTTCACAAGATGATTGCGCAGTCCATGCACCAGATTTGATAAGTACGAT
664157_30677280_HHV-7_JI_U43400.1_144861bp_1_1296	AACAGACAGCGTTTCACAAGATGATTGCGCAGTCCATGCACCAGATTTGATAAGTACGATTTGCTCGACAACGCACACAACGCATCACGACCTAGTCAGAATGAATGGCTCAGCTACTGG
664157_30677280_HHV-7_JI_U43400.1_144861bp_1_1297	TTGCTCGACAACGCACACAACGCATCACGACCTAGTCAGAATGAATGGCTCAGCTACTGGCAACTCAGCTAGTCTTCCCGCTCCTCAGTTTTCCGAATGTGTTTTTTTACCGAAAGATAC

664157_30677280_HHV-7_JI_U43400.1_144861bp_1_1298	CAACTCAGCTAGTCTTCCCGCTCCTCAGTTTTCCGAATGTGTTTTTTACCGAAAGATACATTTTGCTCCTTACTGAATGCTACAGCTGGGGCCCAAAATAAAAACGTAACGCCAGCTGC
664157_30677280_HHV-7_JI_U43400.1_144861bp_1_1299	ATTTTGCTCCTTACTGAATGCTACAGCTGGGGCCCAAAATAAAAACGTAACGCCAGCTGCTCCGATATTTAAAACTGATGAATATATAACTCCGTACCCAGAATCTCTGAGCAGAGTGGA
664157_30677280_HHV-7_JI_U43400.1_144861bp_1_1300	TCCGATATTTAAAACTGATGAATATATAACTCCGTACCCAGAATCTCTGAGCAGAGTGGATTATGGAAATAGAATGAATTATCACATACCACCTCCATATTGGTATCCCTCTATGCCTGG
664157_30677280_HHV-7_JI_U43400.1_144861bp_1_1301	TTATGGAAATAGAATGAATTATCACATACCACCTCCATATTGGTATCCCTCTATGCCTGGATTTAATTATAAATCTTATCGTGGTTCCCAAAAACGGTGCGCTCCAACAGATTCGGATGA
664157_30677280_HHV-7_JI_U43400.1_144861bp_1_1302	ATTTAATTATAAATCTTATCGTGGTTCCCAAAAACGGTGCGCTCCAACAGATTCGGATGATGAAATGAGCTTTCCAGGAGATCCTGACTATACGACAAAGAAAAAGAAGCGGTATAGAGA
664157_30677280_HHV-7_JI_U43400.1_144861bp_1_1303	TGAAATGAGCTTTCCAGGAGATCCTGACTATACGACAAAGAAAAAGAAGCGGTATAGAGAAGATGACGATCGTGAACTCACTAAAGACAAAAATGATATTAAAGAACTAGTTGATGCAAT
664157_30677280_HHV-7_JI_U43400.1_144861bp_1_1304	AGATGACGATCGTGAACTCACTAAAGACAAAAATGATATTAAAGAACTAGTTGATGCAATAGGGATGCTGCGTCATGAAATTTCAGCGCTAAAGTATATTCGGTCTCAGTCTCCTCAGAG
664157_30677280_HHV-7_JI_U43400.1_144861bp_1_1305	AGGGATGCTGCGTCATGAAATTTCAGCGCTAAAGTATATTCGGTCTCAGTCTCCTCAGAGACAGCATTGTACAGCGGTAGATACAATGCCAACGATTGAAGAAAAAAACGTGGCATCACC
664157_30677280_HHV-7_JI_U43400.1_144861bp_1_1306	ACAGCATTGTACAGCGGTAGATACAATGCCAACGATTGAAGAAAAAAACGTGGCATCACCTAAACCATCGGTTGTAAATGCCTCGCTAACTCCAGGTCAAGACAGGAATCAAAATTTGAT
664157_30677280_HHV-7_JI_U43400.1_144861bp_1_1307	TAAACCATCGGTTGTAAATGCCTCGCTAACTCCAGGTCAAGACAGGAATCAAAATTTGATGCAAAGTGATCAATCTTTATTGAGTTTGAACAAAAAATTGTTTGTAGAAGCTTTGAATAA
664157_30677280_HHV-7_JI_U43400.1_144861bp_1_1308	GCAAAGTGATCAATCTTTATTGAGTTTGAACAAAAAATTGTTTGTAGAAGCTTTGAATAAAATGGACAATTGAATACAAATTATGCGTTAATGTCTCTGTTCCCACTGGGACAAGGAACC
664157_30677280_HHV-7_JI_U43400.1_144861bp_1_1309	AATGGACAATTGAATACAAATTATGCGTTAATGTCTCTGTTCCCACTGGGACAAGGAACCGTTTGCAGCCCAATGTTGCCTTCTAAAACAGTTAATGTGTGTGGTAAGGCAGTGATTGGT

664157_30677280_HHV-7_JI_U43400.1_144861bp_1_1310	GTTTGCAGCCCAATGTTGCCTTCTAAAACAGTTAATGTGTGTGGTAAGGCAGTGATTGGTAGAAATGTGCGAAGGTTGTCTGTTCTCATGCGATATCTGACTGGTACAAGGTGTTCCTTA
664157_30677280_HHV-7_JI_U43400.1_144861bp_1_1311	AGAAATGTGCGAAGGTTGTCTGTTCTCATGCGATATCTGACTGGTACAAGGTGTTCCTTAGCTATGTTTAAATGAAGAGAATTTAGATTAATAGATACGTTAGTTGCAGCTGTGATATTT
664157_30677280_HHV-7_JI_U43400.1_144861bp_1_1312	GCTATGTTTAAATGAAGAGAATTTAGATTAATAGATACGTTAGTTGCAGCTGTGATATTTGGTACTGTAGATAGTACAGGACTATCAGCGACAGGTGTTGTTTCCGATCTAAAGACACTT
664157_30677280_HHV-7_JI_U43400.1_144861bp_1_1313	GGTACTGTAGATAGTACAGGACTATCAGCGACAGGTGTTGTTTCCGATCTAAAGACACTTATTTGATTATTTTGATTGGAAATCAAAATTCTGCTTGTCTCTGTCCAGCCAAAATCTATA
664157_30677280_HHV-7_JI_U43400.1_144861bp_1_1314	ATTTGATTATTTTGATTGGAAATCAAAATTCTGCTTGTCTCTGTCCAGCCAAAATCTATATTTGATTGATCGTGTGTTACTATTTGACTGGGTAGGAAAATAAGTTTTCCCAGTTCCTGA
664157_30677280_HHV-7_JI_U43400.1_144861bp_1_1315	TTTGATTGATCGTGTGTTACTATTTGACTGGGTAGGAAAATAAGTTTTCCCAGTTCCTGAGAATAATCAATACGGCAGTTTTTTTTGTAGGCTCTCATGGTTACTTGAAGATGAGTTCTC
664157_30677280_HHV-7_JI_U43400.1_144861bp_1_1316	GAATAATCAATACGGCAGTTTTTTTTGTAGGCTCTCATGGTTACTTGAAGATGAGTTCTCTCTTTCCAAGTGAAAGTGTTTAATGAAATCCCGTTTATTAATCTCGGAATAAAAAGTCCT
664157_30677280_HHV-7_JI_U43400.1_144861bp_1_1317	TCTTTCCAAGTGAAAGTGTTTAATGAAATCCCGTTTATTAATCTCGGAATAAAAAGTCCTGCAAATTTCTGTTTAGAATCAAAAAATGTTGGAAATGTACATGAGAAGGGGTCAGTGGTA
664157_30677280_HHV-7_JI_U43400.1_144861bp_1_1318	GCAAATTTCTGTTTAGAATCAAAAAATGTTGGAAATGTACATGAGAAGGGGTCAGTGGTATTCGAATTAATGTTTTTTGTAGAACCTACGGTTATGCATGTGCCATCGTACGTCATTACA
664157_30677280_HHV-7_JI_U43400.1_144861bp_1_1319	TTCGAATTAATGTTTTTTGTAGAACCTACGGTTATGCATGTGCCATCGTACGTCATTACAGGTTCTTGATTTCTTTCCATCACAATGTGAGGAATGGTTTGGATAAAATTTACTTGGAAA
664157_30677280_HHV-7_JI_U43400.1_144861bp_1_1320	GGTTCTTGATTTCTTTCCATCACAATGTGAGGAATGGTTTGGATAAAATTTACTTGGAAAAAGATGTCGCTAGGAAAATTATCAGGATTTAAAAGATAATTGTTTTCATAAGATGCTCTT
664157_30677280_HHV-7_JI_U43400.1_144861bp_1_1321	AAGATGTCGCTAGGAAAATTATCAGGATTTAAAAGATAATTGTTTTCATAAGATGCTCTTATAACAAGGATTTTTTCATGCAGCATTATTTGCTTAAAATAAACATTTCTGCTGGATATA

664157_30677280_HHV-7_JI_U43400.1_144861bp_1_1322	ATAACAAGGATTTTTTCATGCAGCATTATTTGCTTAAAATAAACATTTCTGCTGGATATAAATTCCACTTTTGCACTGTTAAAAATTTGATCTGGTGGAGTTGTTTGTAAATCTATCCAA
664157_30677280_HHV-7_JI_U43400.1_144861bp_1_1323	AATTCCACTTTTGCACTGTTAAAAATTTGATCTGGTGGAGTTGTTTGTAAATCTATCCAAAATTCACACGCGTACATGTAATGCGTTGGTTCTTCGTGTTGTAATTCTGTCCAGCGTATT
664157_30677280_HHV-7_JI_U43400.1_144861bp_1_1324	AATTCACACGCGTACATGTAATGCGTTGGTTCTTCGTGTTGTAATTCTGTCCAGCGTATTCTCTTAATATGGGCTCTTAATTTAGTTTGAGATCCATAAAGAATAAATTGAGCACGGCAC
664157_30677280_HHV-7_JI_U43400.1_144861bp_1_1325	CTCTTAATATGGGCTCTTAATTTAGTTTGAGATCCATAAAGAATAAATTGAGCACGGCACTCTCCATGTGGAATGTAATTGTGCTCCATGCGCTCAACTATTTGGACAGGAAGTGATACC
664157_30677280_HHV-7_JI_U43400.1_144861bp_1_1326	TCTCCATGTGGAATGTAATTGTGCTCCATGCGCTCAACTATTTGGACAGGAAGTGATACCAAATTAATGCTAGGAAGAAAAAAGGCAAAAAGATTAATAATTAAAGGAAATTGTTTTAAA
664157_30677280_HHV-7_JI_U43400.1_144861bp_1_1327	AAATTAATGCTAGGAAGAAAAAAGGCAAAAAGATTAATAATTAAAGGAAATTGTTTTAAATCTAAATCTACATCAGATATATTTTGAACAGCCAAGGTTACATTGCCACAATCTCTTAGA
664157_30677280_HHV-7_JI_U43400.1_144861bp_1_1328	TCTAAATCTACATCAGATATATTTTGAACAGCCAAGGTTACATTGCCACAATCTCTTAGATCGATATACGTAAAGTAGGCTGCTGTAAAGCTTCCTTCATCTCTGCACATACACACAAAT
664157_30677280_HHV-7_JI_U43400.1_144861bp_1_1329	TCGATATACGTAAAGTAGGCTGCTGTAAAGCTTCCTTCATCTCTGCACATACACACAAATGTTGATTGTTGCGAAAGTATGCTTAGCCCTGTCCTTAAGCATCTTATTTCACGAGGTTTT
664157_30677280_HHV-7_JI_U43400.1_144861bp_1_1330	GTTGATTGTTGCGAAAGTATGCTTAGCCCTGTCCTTAAGCATCTTATTTCACGAGGTTTTAAAAGGATTGGTGCTGTCAATTTTAGTTGTAGAAAATATGAAGACCAGACCACATAACAG
664157_30677280_HHV-7_JI_U43400.1_144861bp_1_1331	AAAAGGATTGGTGCTGTCAATTTTAGTTGTAGAAAATATGAAGACCAGACCACATAACAGGATTCCATTCTTACTTTAAATCTTTTATTGTTGTAAAATACTTAAATAGTTGATATTACA
664157_30677280_HHV-7_JI_U43400.1_144861bp_1_1332	GATTCCATTCTTACTTTAAATCTTTTATTGTTGTAAAATACTTAAATAGTTGATATTACAATTGCATACGTTTGAAGCGGGGTGTTATGTTCATAGAGTTATGTCTAATGTTTTCTGTCT
664157_30677280_HHV-7_JI_U43400.1_144861bp_1_1333	ATTGCATACGTTTGAAGCGGGGTGTTATGTTCATAGAGTTATGTCTAATGTTTTCTGTCTCTTGTATTTGTTCGTAGGGAAAGTCTGTCATGGGATGAACTGTCAAAATTGGGAGAGAGT

664157_30677280_HHV-7_JI_U43400.1_144861bp_1_1334	CTTGTATTTGTTCGTAGGGAAAGTCTGTCATGGGATGAACTGTCAAAATTGGGAGAGAGTCTGAAAAAAAATCGTTCCCTAAAAAGTTAATTCGAAATGAATTGAAAGCGGCTTCAACTT
664157_30677280_HHV-7_JI_U43400.1_144861bp_1_1335	CTGAAAAAAAATCGTTCCCTAAAAAGTTAATTCGAAATGAATTGAAAGCGGCTTCAACTTTTGATTTGCAATCTGCGTGTGCTGTAGGATGGAAAGAATGTGGAAATTGTTCTCTACTGA
664157_30677280_HHV-7_JI_U43400.1_144861bp_1_1336	TTGATTTGCAATCTGCGTGTGCTGTAGGATGGAAAGAATGTGGAAATTGTTCTCTACTGATAAAGTAGACATGACCAATTATATCATGCTCCGTTACAAATGTCTCATTTCGGCAGTACA
664157_30677280_HHV-7_JI_U43400.1_144861bp_1_1337	TAAAGTAGACATGACCAATTATATCATGCTCCGTTACAAATGTCTCATTTCGGCAGTACACGTAAATTCTAAAAGTCTCACGCGGTTGCCAAATGAAAGGAAATGAGAGTACATTAGATT
664157_30677280_HHV-7_JI_U43400.1_144861bp_1_1338	CGTAAATTCTAAAAGTCTCACGCGGTTGCCAAATGAAAGGAAATGAGAGTACATTAGATTTCTTTGGAATGAATAGCCCTACAGCAGCAGGATTCTCAAAGGTTCCTCGTAAATTGATTT
664157_30677280_HHV-7_JI_U43400.1_144861bp_1_1339	TCTTTGGAATGAATAGCCCTACAGCAGCAGGATTCTCAAAGGTTCCTCGTAAATTGATTTGTGTGATGTGATTAGGTGTCAGTGTCATTCTTTCAGGGAAATAAATTTCCATTGTTTTGA
664157_30677280_HHV-7_JI_U43400.1_144861bp_1_1340	GTGTGATGTGATTAGGTGTCAGTGTCATTCTTTCAGGGAAATAAATTTCCATTGTTTTGAATTCTTCAGAAATATTGAAATACGGCATGCAAATGTTAAGTGCAACTGATTGCATATTTG
664157_30677280_HHV-7_JI_U43400.1_144861bp_1_1341	ATTCTTCAGAAATATTGAAATACGGCATGCAAATGTTAAGTGCAACTGATTGCATATTTGGTTTAGAAAGTCTTAAGCGTAAAGAACACTCATCCACACAGAAACGTGAATTCATTACTT
664157_30677280_HHV-7_JI_U43400.1_144861bp_1_1342	GTTTAGAAAGTCTTAAGCGTAAAGAACACTCATCCACACAGAAACGTGAATTCATTACTTTAAGATACCGTAGAAAGACAAATAACACCGGTGATCGATATTCAATCTTGTACACCGAAA
664157_30677280_HHV-7_JI_U43400.1_144861bp_1_1343	TAAGATACCGTAGAAAGACAAATAACACCGGTGATCGATATTCAATCTTGTACACCGAAAGATTGTGTTCTGATATCATTTCCACGTTGCAGATTGGCGTTGCGCTCGCGAAAAAAACAT
664157_30677280_HHV-7_JI_U43400.1_144861bp_1_1344	GATTGTGTTCTGATATCATTTCCACGTTGCAGATTGGCGTTGCGCTCGCGAAAAAAACATTCCAATATTTGCAGTATGTTGTATTGAAGGTGAAGATGCAGTACATGGTTTCCAGTTTTT
664157_30677280_HHV-7_JI_U43400.1_144861bp_1_1345	TCCAATATTTGCAGTATGTTGTATTGAAGGTGAAGATGCAGTACATGGTTTCCAGTTTTTGATCATTGGAGTTTTTCAGTAAAGGTTGAAATTCATTCTTTTCCCAAGTAATTTTAGTTA

664157_30677280_HHV-7_JI_U43400.1_144861bp_1_1346	GATCATTGGAGTTTTTCAGTAAAGGTTGAAATTCATTCTTTTCCCAAGTAATTTTAGTTACTGTGGCATTTACGATCAGATCACCATTTTTCACAGAATATAAAATTATAGGAGTTGTTC
664157_30677280_HHV-7_JI_U43400.1_144861bp_1_1347	CTGTGGCATTTACGATCAGATCACCATTTTTCACAGAATATAAAATTATAGGAGTTGTTCCTAAACCCTTTTCAAGATAGGCAAATTGATGAAATCTAAGAGGATCCATATTTTCTTTAA
664157_30677280_HHV-7_JI_U43400.1_144861bp_1_1348	CTAAACCCTTTTCAAGATAGGCAAATTGATGAAATCTAAGAGGATCCATATTTTCTTTAAAGTAACAATTATCTTGTATCAGCAGGGATGACGTAATGTTAAAAATTGGCAGGGCAAAAA
664157_30677280_HHV-7_JI_U43400.1_144861bp_1_1349	AGTAACAATTATCTTGTATCAGCAGGGATGACGTAATGTTAAAAATTGGCAGGGCAAAAATATTGACAAAGTTTATCGATTGTATCATAGGAAAAACATTAATTTTGATCACTTTTTTGT
664157_30677280_HHV-7_JI_U43400.1_144861bp_1_1350	TATTGACAAAGTTTATCGATTGTATCATAGGAAAAACATTAATTTTGATCACTTTTTTGTTTTTTAGAACGTGACCCACCAAAGTTACGCATTCTGCATTGTTTTCAGTTCCATCGGTAA
664157_30677280_HHV-7_JI_U43400.1_144861bp_1_1351	TTTTTAGAACGTGACCCACCAAAGTTACGCATTCTGCATTGTTTTCAGTTCCATCGGTAATTAGCAACAGTACAGGTCTTTCGTGCGAAAGTATGCAGGTAATCTCTGCGGGAGAAAAAT
664157_30677280_HHV-7_JI_U43400.1_144861bp_1_1352	TTAGCAACAGTACAGGTCTTTCGTGCGAAAGTATGCAGGTAATCTCTGCGGGAGAAAAATTGAGTTCTGGCTGCAAATCATTAAAATCCACAGTAAACTGAATTAATTGCGATAATTCAG
664157_30677280_HHV-7_JI_U43400.1_144861bp_1_1353	TGAGTTCTGGCTGCAAATCATTAAAATCCACAGTAAACTGAATTAATTGCGATAATTCAGAGACTGGTGTTGAATTCATTTTTACAAAGAAAACTAGAATATACAAATGATTCTTAATTG
664157_30677280_HHV-7_JI_U43400.1_144861bp_1_1354	AGACTGGTGTTGAATTCATTTTTACAAAGAAAACTAGAATATACAAATGATTCTTAATTGTTTTCACACGTTTTATAGAATCTAGCAAGCTTCTAAGGCTTTTTAAAACCGCAATCTACT
664157_30677280_HHV-7_JI_U43400.1_144861bp_1_1355	TTTTCACACGTTTTATAGAATCTAGCAAGCTTCTAAGGCTTTTTAAAACCGCAATCTACTTTTTGAAGGGGCGTGGTTATTTATATTTGATCCAGATTGCAATTTTTTTTTACGAGCGCT
664157_30677280_HHV-7_JI_U43400.1_144861bp_1_1356	TTTTGAAGGGGCGTGGTTATTTATATTTGATCCAGATTGCAATTTTTTTTTACGAGCGCTCCAACCTTTTCTTAAGATTAAATCTGGCAAATCAGAGATAAAAATTTTGTCTGCTAAAAA
664157_30677280_HHV-7_JI_U43400.1_144861bp_1_1357	CCAACCTTTTCTTAAGATTAAATCTGGCAAATCAGAGATAAAAATTTTGTCTGCTAAAAAATACAGTGCGTCAGATTCTGGATCTTCTGAGTTAAGCTCAATTTCAGCAGCAAAACTATC

664157_30677280_HHV-7_JI_U43400.1_144861bp_1_1358	ATACAGTGCGTCAGATTCTGGATCTTCTGAGTTAAGCTCAATTTCAGCAGCAAAACTATCCGTATAGTTGGGATCAAATTTAGTTCGTAAAATATGCGTTGGGATAAAATAAATGCTGCC
664157_30677280_HHV-7_JI_U43400.1_144861bp_1_1359	CGTATAGTTGGGATCAAATTTAGTTCGTAAAATATGCGTTGGGATAAAATAAATGCTGCCTATTATGTCATTTGTAGTCACTCTCTGCGATCTATCACTAGTCACTTTTGTGGTAAAACT
664157_30677280_HHV-7_JI_U43400.1_144861bp_1_1360	TATTATGTCATTTGTAGTCACTCTCTGCGATCTATCACTAGTCACTTTTGTGGTAAAACTTGTAGTGGGAGACCAGAAAGAAGCAATAGACTGCACTTTGGAATTTGGGTCTGGAACGTA
664157_30677280_HHV-7_JI_U43400.1_144861bp_1_1361	TGTAGTGGGAGACCAGAAAGAAGCAATAGACTGCACTTTGGAATTTGGGTCTGGAACGTAGAGAGCATTAGCATTAAAATTATAAAAAGTGCCTTTAAAATGAAGTTTTTTAGTTTCGCC
664157_30677280_HHV-7_JI_U43400.1_144861bp_1_1362	GAGAGCATTAGCATTAAAATTATAAAAAGTGCCTTTAAAATGAAGTTTTTTAGTTTCGCCCTCATCGAAAAGCATCGTGTCTTGGAAAAAAACGTCAATTTTTCTTTTATCGCTACAGAT
664157_30677280_HHV-7_JI_U43400.1_144861bp_1_1363	CTCATCGAAAAGCATCGTGTCTTGGAAAAAAACGTCAATTTTTCTTTTATCGCTACAGATTTTAAAGTAATGCATATGCACAGTTAGAGAAATTTCGTTTGGGATTATTTCGTTGACAAA
664157_30677280_HHV-7_JI_U43400.1_144861bp_1_1364	TTTAAAGTAATGCATATGCACAGTTAGAGAAATTTCGTTTGGGATTATTTCGTTGACAAAACCAAGTTTAAGCATGCAATCGTTTGGAACCATATACGTGTTATTTTTAGTTACCTCTGT
664157_30677280_HHV-7_JI_U43400.1_144861bp_1_1365	ACCAAGTTTAAGCATGCAATCGTTTGGAACCATATACGTGTTATTTTTAGTTACCTCTGTATAACATCGAAGATAGATTTCGAGTAGAGGAGGAGAAAAGATCATTTTAAGTACGGAAAC
664157_30677280_HHV-7_JI_U43400.1_144861bp_1_1366	ATAACATCGAAGATAGATTTCGAGTAGAGGAGGAGAAAAGATCATTTTAAGTACGGAAACTGTTGGGTCTGAAATAGAAAACACTTTGCACGTTGGAAAATCTGAACCACTCCGAACATC
664157_30677280_HHV-7_JI_U43400.1_144861bp_1_1367	TGTTGGGTCTGAAATAGAAAACACTTTGCACGTTGGAAAATCTGAACCACTCCGAACATCGTGAAACCAAAAAGGTAAATTGTTTATGTTAACAGAGCAGACTGTACATAGAGTTGTTAA
664157_30677280_HHV-7_JI_U43400.1_144861bp_1_1368	GTGAAACCAAAAAGGTAAATTGTTTATGTTAACAGAGCAGACTGTACATAGAGTTGTTAATGGATGGCCAGGTGTTCTTTCAATCGAACGTTGATAAGTACTTAGTTTCCATTCAATGTT
664157_30677280_HHV-7_JI_U43400.1_144861bp_1_1369	TGGATGGCCAGGTGTTCTTTCAATCGAACGTTGATAAGTACTTAGTTTCCATTCAATGTTAAACATCGTGACGGTTATTTCGTTTACTTTATGTGGGGTTTGCGTCACTATGACCTTTTC

664157_30677280_HHV-7_JI_U43400.1_144861bp_1_1370	AAACATCGTGACGGTTATTTCGTTTACTTTATGTGGGGTTTGCGTCACTATGACCTTTTCGTTTTTGAATCTTATCAGACTAGGAAAAACTCCCATCTGCTCTTGTTCTTTAACATCATG
664157_30677280_HHV-7_JI_U43400.1_144861bp_1_1371	GTTTTTGAATCTTATCAGACTAGGAAAAACTCCCATCTGCTCTTGTTCTTTAACATCATGGTTTGGGATGATGACGATAGAGCTGTTAATTTTTAACAACGGAAATCCAAAAAACAAGAC
664157_30677280_HHV-7_JI_U43400.1_144861bp_1_1372	GTTTGGGATGATGACGATAGAGCTGTTAATTTTTAACAACGGAAATCCAAAAAACAAGACTTTTGATGGATAAATGTGTCTATTCAGATTTTTTAAATGTAGTTTTAGTTGTTGTTCTTC
664157_30677280_HHV-7_JI_U43400.1_144861bp_1_1373	TTTTGATGGATAAATGTGTCTATTCAGATTTTTTAAATGTAGTTTTAGTTGTTGTTCTTCCATTAAAGGACGAGCAGAAAAAAAGGAGTCAGAATTCCTTGAATTTATTTCAGCGCTCAA
664157_30677280_HHV-7_JI_U43400.1_144861bp_1_1374	CATTAAAGGACGAGCAGAAAAAAAGGAGTCAGAATTCCTTGAATTTATTTCAGCGCTCAAAAGTAAAACTGGTTCAGTTTTTGATTGGACATACTCGTCAAATAATAATGTAACATTATC
664157_30677280_HHV-7_JI_U43400.1_144861bp_1_1375	AAGTAAAACTGGTTCAGTTTTTGATTGGACATACTCGTCAAATAATAATGTAACATTATCTTTACCCTGGAATTCTTCTTTGTATGGAATTTCAATCTGAACATATTTTAAGATATCAGA
664157_30677280_HHV-7_JI_U43400.1_144861bp_1_1376	TTTACCCTGGAATTCTTCTTTGTATGGAATTTCAATCTGAACATATTTTAAGATATCAGAAGTTGCGAGAGACATGGAGCTGAAATAAAATCTGTTACAACAGACACTATTTCAAAGAAC
664157_30677280_HHV-7_JI_U43400.1_144861bp_1_1377	AGTTGCGAGAGACATGGAGCTGAAATAAAATCTGTTACAACAGACACTATTTCAAAGAACTTTTTATTCACTCTTTAAATATACAAGTTGCCAGCCTATTGTCAGCAGAAACATCAGCCA
664157_30677280_HHV-7_JI_U43400.1_144861bp_1_1378	TTTTTATTCACTCTTTAAATATACAAGTTGCCAGCCTATTGTCAGCAGAAACATCAGCCACAATGAAAAACGTTTTTTCTTGTGTAAGGGAATTGATTTGGTTAATCATGGTGAAAACCG
664157_30677280_HHV-7_JI_U43400.1_144861bp_1_1379	CAATGAAAAACGTTTTTTCTTGTGTAAGGGAATTGATTTGGTTAATCATGGTGAAAACCGTTTTAATTTTCTGTAATTCTGTTTTAATTTCAGTGGCCTTGAAAGAACTAGGTTCTTGAG
664157_30677280_HHV-7_JI_U43400.1_144861bp_1_1380	TTTTAATTTTCTGTAATTCTGTTTTAATTTCAGTGGCCTTGAAAGAACTAGGTTCTTGAGAAAAATTTTTTTTTGCTAGGAGTTCTTGACATTTAATCAATAACATAGACTGATCTTCTA
664157_30677280_HHV-7_JI_U43400.1_144861bp_1_1381	AAAAATTTTTTTTTGCTAGGAGTTCTTGACATTTAATCAATAACATAGACTGATCTTCTAAAGATAATCTTTCTATATATGTTTGAACTATATCAGGAGCCATGGTGGCAATCATAGAAA

664157_30677280_HHV-7_JI_U43400.1_144861bp_1_1382	AAGATAATCTTTCTATATATGTTTGAACTATATCAGGAGCCATGGTGGCAATCATAGAAAAAGAAATGCATGCTGTTTTCATGGAAAACGTGGACTTACAATCATTGATAACATCTGGTA
664157_30677280_HHV-7_JI_U43400.1_144861bp_1_1383	AAGAAATGCATGCTGTTTTCATGGAAAACGTGGACTTACAATCATTGATAACATCTGGTAACTTAATTGTTACATCTCTGTACTGCAAAGTGCGCAGCTCATCTATAATTATTTGAACTT
664157_30677280_HHV-7_JI_U43400.1_144861bp_1_1384	ACTTAATTGTTACATCTCTGTACTGCAAAGTGCGCAGCTCATCTATAATTATTTGAACTTCTTCATATTTTCTATGTATGGAAAGGAGGCTGATGCAAAGCATTCGCACGTTTATTTCCG
664157_30677280_HHV-7_JI_U43400.1_144861bp_1_1385	CTTCATATTTTCTATGTATGGAAAGGAGGCTGATGCAAAGCATTCGCACGTTTATTTCCGTTGCGACGTCTGTAGGGATCAGTAATGGCAGAATCAGCTCCCAGTTAGGTAAGACAAGTA
664157_30677280_HHV-7_JI_U43400.1_144861bp_1_1386	TTGCGACGTCTGTAGGGATCAGTAATGGCAGAATCAGCTCCCAGTTAGGTAAGACAAGTAATTGTTGCGAAGGTTTCAGAAATGGTGGCAGTAAACTCAATTGATCTCCCTTTTCCAGAG
664157_30677280_HHV-7_JI_U43400.1_144861bp_1_1387	ATTGTTGCGAAGGTTTCAGAAATGGTGGCAGTAAACTCAATTGATCTCCCTTTTCCAGAGGAAATGGTCCGGTATTTTTGATTTCATACTGGTGTCCAATTTTTGGACGTATTAATATTA
664157_30677280_HHV-7_JI_U43400.1_144861bp_1_1388	GAAATGGTCCGGTATTTTTGATTTCATACTGGTGTCCAATTTTTGGACGTATTAATATTAATTGGTTTCCTTCTACTTTTTGTAAGATTGTTAACACCATTGTTTTTAAGATATCCCTTA
664157_30677280_HHV-7_JI_U43400.1_144861bp_1_1389	ATTGGTTTCCTTCTACTTTTTGTAAGATTGTTAACACCATTGTTTTTAAGATATCCCTTATTTGAATATAATCGGTGGTTGTAGATAAGACCGTGTAAAGTCCTAAATTTGTGTTGCCAA
664157_30677280_HHV-7_JI_U43400.1_144861bp_1_1390	TTTGAATATAATCGGTGGTTGTAGATAAGACCGTGTAAAGTCCTAAATTTGTGTTGCCAATTAAATGACGGTGCGACGGTATTGGGATGACGGCATTAGTGAGTTTGCATAAAGTTCCAA
664157_30677280_HHV-7_JI_U43400.1_144861bp_1_1391	TTAAATGACGGTGCGACGGTATTGGGATGACGGCATTAGTGAGTTTGCATAAAGTTCCAATGTCAGAAAGAGTAAGCTTTTGATCAAAAGTACAGTAGATTGAATCCATTTCTATAGCAG
664157_30677280_HHV-7_JI_U43400.1_144861bp_1_1392	TGTCAGAAAGAGTAAGCTTTTGATCAAAAGTACAGTAGATTGAATCCATTTCTATAGCAGGGACTCAATAATATTTTGTAACGGAATACACTCCCCTATAGCGAAATTTGCAAAATGCGT
664157_30677280_HHV-7_JI_U43400.1_144861bp_1_1393	GGACTCAATAATATTTTGTAACGGAATACACTCCCCTATAGCGAAATTTGCAAAATGCGTTTCACTTAACTGTGTCATTCCGTTCTTAGATCGACTCTCTAATAGAGCTTGGTTAGAGGA

664157_30677280_HHV-7_JI_U43400.1_144861bp_1_1394	TTCACTTAACTGTGTCATTCCGTTCTTAGATCGACTCTCTAATAGAGCTTGGTTAGAGGAGCAATGGATTGGAAACGCAATTTGTAAAAACTGACAAGGACGATGAGTGACATCGTCTGT
664157_30677280_HHV-7_JI_U43400.1_144861bp_1_1395	GCAATGGATTGGAAACGCAATTTGTAAAAACTGACAAGGACGATGAGTGACATCGTCTGTTCCTTCGGAACAAGAATACTGAATATCAGTGTCGCTGTCCAGGCATGTTTTAGTTTTTAT
664157_30677280_HHV-7_JI_U43400.1_144861bp_1_1396	TCCTTCGGAACAAGAATACTGAATATCAGTGTCGCTGTCCAGGCATGTTTTAGTTTTTATGGAATAATCACTGATAAGTTTAAACATAAACTTATTTGTCTTAAATATTTCAGTGTCTGT
664157_30677280_HHV-7_JI_U43400.1_144861bp_1_1397	GGAATAATCACTGATAAGTTTAAACATAAACTTATTTGTCTTAAATATTTCAGTGTCTGTGAAAAAGACTGCGTTAGGACTGTAACTTTTTGGATTATATCCTAATTGATCTCTGTGATT
664157_30677280_HHV-7_JI_U43400.1_144861bp_1_1398	GAAAAAGACTGCGTTAGGACTGTAACTTTTTGGATTATATCCTAATTGATCTCTGTGATTTATATTATATAAGACATCAGATAAAGATCCTTCTTGAGATGCCCAAGGATTTGTTGTTGC
664157_30677280_HHV-7_JI_U43400.1_144861bp_1_1399	TATATTATATAAGACATCAGATAAAGATCCTTCTTGAGATGCCCAAGGATTTGTTGTTGCCACAAAAGCATCGCTATCAACTCGAGAATGATCATACAAAGATTTTCTAGCTTCTGACTC
664157_30677280_HHV-7_JI_U43400.1_144861bp_1_1400	CACAAAAGCATCGCTATCAACTCGAGAATGATCATACAAAGATTTTCTAGCTTCTGACTCATTATGGGGATCGACACCCATCATACACGAAGCCCTTCCTCTAGGATTTTTAGGTGTTTT
664157_30677280_HHV-7_JI_U43400.1_144861bp_1_1401	ATTATGGGGATCGACACCCATCATACACGAAGCCCTTCCTCTAGGATTTTTAGGTGTTTTATAGAAATTAATATCGGAAGTCACTGGAGTAATGACAACCTCGCAGATTGCTTGTTGGCC
664157_30677280_HHV-7_JI_U43400.1_144861bp_1_1402	ATAGAAATTAATATCGGAAGTCACTGGAGTAATGACAACCTCGCAGATTGCTTGTTGGCCATGTAAAAGTATTGATTGTGATTGCTTATTTATCTTACCGAAAGAAAGGATGTTTAAAGC
664157_30677280_HHV-7_JI_U43400.1_144861bp_1_1403	ATGTAAAAGTATTGATTGTGATTGCTTATTTATCTTACCGAAAGAAAGGATGTTTAAAGCATCGGTTTCGGAAGGATTGGGTTTTTCAATGCCAACGTGATGCCTAACCCAACTATTTAC
664157_30677280_HHV-7_JI_U43400.1_144861bp_1_1404	ATCGGTTTCGGAAGGATTGGGTTTTTCAATGCCAACGTGATGCCTAACCCAACTATTTACAGTAGGGTTGGTGTATGCGTGCATTGGAAAGACCGAAAATAGATCTTGCACTTTACTTCC
664157_30677280_HHV-7_JI_U43400.1_144861bp_1_1405	AGTAGGGTTGGTGTATGCGTGCATTGGAAAGACCGAAAATAGATCTTGCACTTTACTTCCCATATCAGTTTTTACCCTTTTCAAATTTGCGATTGCAGTGCTGGAACTAAATCCTAATCC

664157_30677280_HHV-7_JI_U43400.1_144861bp_1_1406	CATATCAGTTTTTACCCTTTTCAAATTTGCGATTGCAGTGCTGGAACTAAATCCTAATCCCATATCAAGAAAACTTATATGCTGCGTAAGGTTATATGTGGTTGAGATGTCTTTTACTTC
664157_30677280_HHV-7_JI_U43400.1_144861bp_1_1407	CATATCAAGAAAACTTATATGCTGCGTAAGGTTATATGTGGTTGAGATGTCTTTTACTTCTGTAGATACCGTTGGGTCATCAATCATTATGGATGTTGCAGATTTGGAGCTATACAGTAA
664157_30677280_HHV-7_JI_U43400.1_144861bp_1_1408	TGTAGATACCGTTGGGTCATCAATCATTATGGATGTTGCAGATTTGGAGCTATACAGTAAGCAATTAATGTCGAAACAATCTGTTCTTACTAAAGTCGCAGCGAAACCAGGATGAATTTT
664157_30677280_HHV-7_JI_U43400.1_144861bp_1_1409	GCAATTAATGTCGAAACAATCTGTTCTTACTAAAGTCGCAGCGAAACCAGGATGAATTTTTTGTCTGCTTTGAAGAATGATAGCTATTGGAGACAATTTGCAATGCATAGTAGCTAAAGT
664157_30677280_HHV-7_JI_U43400.1_144861bp_1_1410	TTGTCTGCTTTGAAGAATGATAGCTATTGGAGACAATTTGCAATGCATAGTAGCTAAAGTCATGATACTTAGCAATGAATTTGAACAACAAGAAGCATAGACAAAGAACGGTGTTCTTTG
664157_30677280_HHV-7_JI_U43400.1_144861bp_1_1411	CATGATACTTAGCAATGAATTTGAACAACAAGAAGCATAGACAAAGAACGGTGTTCTTTGCCAATTATGGAATTCATGAGATAATGCTTGTGGTAGAGGAAAACCACCGTCATTTCTTTG
664157_30677280_HHV-7_JI_U43400.1_144861bp_1_1412	CCAATTATGGAATTCATGAGATAATGCTTGTGGTAGAGGAAAACCACCGTCATTTCTTTGATAGTGGGGAAATATGTTTAGATAGGTTTGTACTTCAATATTCATTAAGCCACAAATAAT
664157_30677280_HHV-7_JI_U43400.1_144861bp_1_1413	ATAGTGGGGAAATATGTTTAGATAGGTTTGTACTTCAATATTCATTAAGCCACAAATAATAGGGTCTGAGAAAAAACGATTAAAAGGAATTGGCTTGAAATACCGTTTCAACACATTAAT
664157_30677280_HHV-7_JI_U43400.1_144861bp_1_1414	AGGGTCTGAGAAAAAACGATTAAAAGGAATTGGCTTGAAATACCGTTTCAACACATTAATTGGTGATACTAAACACAGTCCATTATATAAAACATGTTGTAGAGATTTGAAGTTTGAAGA
664157_30677280_HHV-7_JI_U43400.1_144861bp_1_1415	TGGTGATACTAAACACAGTCCATTATATAAAACATGTTGTAGAGATTTGAAGTTTGAAGAACTACCATGTCTTTGAGAAGGATCTAAAGAAGATTCAATTTCCAAAATTTTTGTATTCTC
664157_30677280_HHV-7_JI_U43400.1_144861bp_1_1416	ACTACCATGTCTTTGAGAAGGATCTAAAGAAGATTCAATTTCCAAAATTTTTGTATTCTCCGTTAGGTATAATATAGATTTAAAGAGTTGTTTACCAATGCAAGATAAATCTGTGAGTGC
664157_30677280_HHV-7_JI_U43400.1_144861bp_1_1417	CGTTAGGTATAATATAGATTTAAAGAGTTGTTTACCAATGCAAGATAAATCTGTGAGTGCATGTGAGGGACCAACAGCTTCAGTTATCAATTCAATCAATACATTGTTCGTAATCGGTAA

664157_30677280_HHV-7_JI_U43400.1_144861bp_1_1418	ATGTGAGGGACCAACAGCTTCAGTTATCAATTCAATCAATACATTGTTCGTAATCGGTAATTCGCAAAAGTTGTCATCAGGTAATATAAAGGGTTCTGTGTAGAAGAAATCTAGAATGAA
664157_30677280_HHV-7_JI_U43400.1_144861bp_1_1419	TTCGCAAAAGTTGTCATCAGGTAATATAAAGGGTTCTGTGTAGAAGAAATCTAGAATGAAAGATTTCACATCAAAACCTGCACCACACATCTTGTTATTTGTCAGTGCTGGTAGAAAGCA
664157_30677280_HHV-7_JI_U43400.1_144861bp_1_1420	AGATTTCACATCAAAACCTGCACCACACATCTTGTTATTTGTCAGTGCTGGTAGAAAGCAGAAATAAAAAATTTTGCTTAAAACAATTGTTTCAAAAGATGGTCTATCTACGTCTGTGAA
664157_30677280_HHV-7_JI_U43400.1_144861bp_1_1421	GAAATAAAAAATTTTGCTTAAAACAATTGTTTCAAAAGATGGTCTATCTACGTCTGTGAAGATTTCTGTTGAATCCAATAAATTCATTCTATTCAGGTCAGATACTTCATAGTTTCTTAA
664157_30677280_HHV-7_JI_U43400.1_144861bp_1_1422	GATTTCTGTTGAATCCAATAAATTCATTCTATTCAGGTCAGATACTTCATAGTTTCTTAATTTCACTGTATTTTGGGTAAGGGGAGTGTTACCAGCTGTGATTATTGCATTTGCTTCATT
664157_30677280_HHV-7_JI_U43400.1_144861bp_1_1423	TTTCACTGTATTTTGGGTAAGGGGAGTGTTACCAGCTGTGATTATTGCATTTGCTTCATTTTTTGGAAGACTGTTTAGAAATGGAGGGAATAGACGATTATCAAACAAAGCGTTCACAAA
664157_30677280_HHV-7_JI_U43400.1_144861bp_1_1424	TTTTGGAAGACTGTTTAGAAATGGAGGGAATAGACGATTATCAAACAAAGCGTTCACAAATCCAACCAATGGTTCCCCACACAGTTGCTCATTAAAGTTTGAAATAGAAATTGTCCTTTT
664157_30677280_HHV-7_JI_U43400.1_144861bp_1_1425	TCCAACCAATGGTTCCCCACACAGTTGCTCATTAAAGTTTGAAATAGAAATTGTCCTTTTCACTAATTTTAAGATTGAAATTATATTTTTGTAATGTGTATAAGCAATTCCTGGAATTGA
664157_30677280_HHV-7_JI_U43400.1_144861bp_1_1426	CACTAATTTTAAGATTGAAATTATATTTTTGTAATGTGTATAAGCAATTCCTGGAATTGACTCATCTCCAAGATAGGTTGTAATTAACCAAATCATTTCAAAATTATTGCAAAACAATAA
664157_30677280_HHV-7_JI_U43400.1_144861bp_1_1427	CTCATCTCCAAGATAGGTTGTAATTAACCAAATCATTTCAAAATTATTGCAAAACAATAATATGTGCTTTATATTATACCAATACGTAATACATTGGCTGACAACGTCTTTAAGAATCTG
664157_30677280_HHV-7_JI_U43400.1_144861bp_1_1428	TATGTGCTTTATATTATACCAATACGTAATACATTGGCTGACAACGTCTTTAAGAATCTGAAACGCTGTCTTATTACCATGGATGAGAAGTTCTATGATATATGCTAGTTCGGGATAAGC
664157_30677280_HHV-7_JI_U43400.1_144861bp_1_1429	AAACGCTGTCTTATTACCATGGATGAGAAGTTCTATGATATATGCTAGTTCGGGATAAGCTGTGTTAGTTAAACTTTCAACGACGAGTTTCAGGGTGTAGTCATAATTTAAATTATTTGT

664157_30677280_HHV-7_JI_U43400.1_144861bp_1_1430	TGTGTTAGTTAAACTTTCAACGACGAGTTTCAGGGTGTAGTCATAATTTAAATTATTTGTCTTGGCTTGTTCGATCATTTGATTGGTCCTAGCTTCATGAAAAGATGAAGGTGCTAAAGG
664157_30677280_HHV-7_JI_U43400.1_144861bp_1_1431	CTTGGCTTGTTCGATCATTTGATTGGTCCTAGCTTCATGAAAAGATGAAGGTGCTAAAGGCAGAGGTATATTGCCTAACAAAATTCTTGGTGAACACAAAACATCAGTTGATCTGTTTTT
664157_30677280_HHV-7_JI_U43400.1_144861bp_1_1432	CAGAGGTATATTGCCTAACAAAATTCTTGGTGAACACAAAACATCAGTTGATCTGTTTTTTTGAATATAGGTGAAATCAAAAAATGGATGTAGCTCTGTCTTTAAAGTAAAATTTTCAGA
664157_30677280_HHV-7_JI_U43400.1_144861bp_1_1433	TTGAATATAGGTGAAATCAAAAAATGGATGTAGCTCTGTCTTTAAAGTAAAATTTTCAGATTTATAGAAATCTTCTGTGGTAAGTTCATTTTTAAGCATATTCGTCGTCTTAGGTATTTC
664157_30677280_HHV-7_JI_U43400.1_144861bp_1_1434	TTTATAGAAATCTTCTGTGGTAAGTTCATTTTTAAGCATATTCGTCGTCTTAGGTATTTCTTTTTTCATTTCATATAAGTTGTATAAATTTGATAAAAAGGTGCTTGGAGGTTCTCTAAC
664157_30677280_HHV-7_JI_U43400.1_144861bp_1_1435	TTTTTTCATTTCATATAAGTTGTATAAATTTGATAAAAAGGTGCTTGGAGGTTCTCTAACAAATTCAACCTGACACAGTTGGTTAAAAGATTCCCCCGTAGGTTTTGCAGCATTTTTAAA
664157_30677280_HHV-7_JI_U43400.1_144861bp_1_1436	AAATTCAACCTGACACAGTTGGTTAAAAGATTCCCCCGTAGGTTTTGCAGCATTTTTAAAAATTCGCTCTGAGACACTACAATCAAAAATAATTGGATGGCATAATGATGGCAATAGATC
664157_30677280_HHV-7_JI_U43400.1_144861bp_1_1437	AATTCGCTCTGAGACACTACAATCAAAAATAATTGGATGGCATAATGATGGCAATAGATCACTATAGTCTATTCTCTGCAAAAGCTTATCCTTATTATAGAAATAGATAGATGTGGGTAG
664157_30677280_HHV-7_JI_U43400.1_144861bp_1_1438	ACTATAGTCTATTCTCTGCAAAAGCTTATCCTTATTATAGAAATAGATAGATGTGGGTAGATTGTTTTCCATTGTATCATTTAGTTTAAGCCTGCTATCCATAGTGCTAAAACCACTATC
664157_30677280_HHV-7_JI_U43400.1_144861bp_1_1439	ATTGTTTTCCATTGTATCATTTAGTTTAAGCCTGCTATCCATAGTGCTAAAACCACTATCCTTCGAAATGTATAAGCCCATTGGGAAAAAAAACGTCAATTCCAATTTTTGTTCCAAAGG
664157_30677280_HHV-7_JI_U43400.1_144861bp_1_1440	CTTCGAAATGTATAAGCCCATTGGGAAAAAAAACGTCAATTCCAATTTTTGTTCCAAAGGATCCTCAATGTTAGTGTTTTTATACACTTTCTTTAAATGATCTAAGACAACTGTTTTATC
664157_30677280_HHV-7_JI_U43400.1_144861bp_1_1441	ATCCTCAATGTTAGTGTTTTTATACACTTTCTTTAAATGATCTAAGACAACTGTTTTATCACTCAGTTGAATAATGTTTGTCTTTAGATCAGCATGTTGAGTTTGGTTTTCAAAGATTTC

664157_30677280_HHV-7_JI_U43400.1_144861bp_1_1442	ACTCAGTTGAATAATGTTTGTCTTTAGATCAGCATGTTGAGTTTGGTTTTCAAAGATTTCAGATTTTTTGTTTTGGTCTTGTGTTTCAGTCGCAACGTTTTTGGTATAATTTGAAAAATC
664157_30677280_HHV-7_JI_U43400.1_144861bp_1_1443	AGATTTTTTGTTTTGGTCTTGTGTTTCAGTCGCAACGTTTTTGGTATAATTTGAAAAATCCGCCATAATAGCCTGGTATGCTATTGCAGTAACAGCATTTTCTTTCCCCATAACAAATGT
664157_30677280_HHV-7_JI_U43400.1_144861bp_1_1444	CGCCATAATAGCCTGGTATGCTATTGCAGTAACAGCATTTTCTTTCCCCATAACAAATGTGCCATAAGAAACAGGTACAGACATTGTCATTTGTGAAATGTGTTGCGATAGGGCGTTGGA
664157_30677280_HHV-7_JI_U43400.1_144861bp_1_1445	GCCATAAGAAACAGGTACAGACATTGTCATTTGTGAAATGTGTTGCGATAGGGCGTTGGACAAAATCTGAATCGTATTAGGAGTTCCGAGTAAAACGCCGTTAATAGGTGTTCCATCTTT
664157_30677280_HHV-7_JI_U43400.1_144861bp_1_1446	CAAAATCTGAATCGTATTAGGAGTTCCGAGTAAAACGCCGTTAATAGGTGTTCCATCTTTTAAGACATAGCTATTGGTGTTATTTAAAATGCTTTCAGTGGTTGAATCAACCATTTCACA
664157_30677280_HHV-7_JI_U43400.1_144861bp_1_1447	TAAGACATAGCTATTGGTGTTATTTAAAATGCTTTCAGTGGTTGAATCAACCATTTCACACAGATATCTGTATATAAAGCTTACATTAGAAGTTCTGTTCAAAAAAAAATAAGGAATTGAT
664157_30677280_HHV-7_JI_U43400.1_144861bp_1_1448	CAGATATCTGTATATAAAGCTTACATTAGAAGTTCTGTTCAAAAAAAAATAAGGAATTGATGAGTTTGTTTTTAAAGTTTTGAAACATATTGCTTCGCTGTACTTTAGAAAGAATCTGTTT
664157_30677280_HHV-7_JI_U43400.1_144861bp_1_1449	GAGTTTGTTTTTAAAGTTTTGAAACATATTGCTTCGCTGTACTTTAGAAAGAATCTGTTTACTGTTGACTTTGTTTTCTAACATGGTTTTCAGGATAAACTGTGGCGGTGCCTTCTTAAG
664157_30677280_HHV-7_JI_U43400.1_144861bp_1_1450	ACTGTTGACTTTGTTTTCTAACATGGTTTTCAGGATAAACTGTGGCGGTGCCTTCTTAAGAATAGTTTTAATGAAAGCATATATTAACCCTCGTTGTAACGAATCAGCAGAATTCTTCAG
664157_30677280_HHV-7_JI_U43400.1_144861bp_1_1451	AATAGTTTTAATGAAAGCATATATTAACCCTCGTTGTAACGAATCAGCAGAATTCTTCAGCGATCGTAACACCGTATGAATCGCATTGATGTTGAGCATTTGATCTAAAATTGTGTTTTT
664157_30677280_HHV-7_JI_U43400.1_144861bp_1_1452	CGATCGTAACACCGTATGAATCGCATTGATGTTGAGCATTTGATCTAAAATTGTGTTTTTAAAGGTGTTTTCTAAATGCGCTAAACAAGCAGCACTTAATTCAAACGATATATTGATAGG
664157_30677280_HHV-7_JI_U43400.1_144861bp_1_1453	AAAGGTGTTTTCTAAATGCGCTAAACAAGCAGCACTTAATTCAAACGATATATTGATAGGATGCTTTTCTGAATACTTGGTAACCAGTACGGTTGTTTCTTTAGGCGCAGTTACGTCGTT

664157_30677280_HHV-7_JI_U43400.1_144861bp_1_1454	ATGCTTTTCTGAATACTTGGTAACCAGTACGGTTGTTTCTTTAGGCGCAGTTACGTCGTTTCCTGTAGCGACTCTAGGTAATTGTATATAAAACAGAATTTTTCCCAGTGACATTTTGTC
664157_30677280_HHV-7_JI_U43400.1_144861bp_1_1455	TCCTGTAGCGACTCTAGGTAATTGTATATAAAACAGAATTTTTCCCAGTGACATTTTGTCTAGGTCGTTGAAACGGATAACATTTGCAGCAACCGCTATGGACGTATGAAAAAAATCTAT
664157_30677280_HHV-7_JI_U43400.1_144861bp_1_1456	TAGGTCGTTGAAACGGATAACATTTGCAGCAACCGCTATGGACGTATGAAAAAAATCTATCCATTCAGTCCGATTACAGTAAATCCCAAGTAATGCTTCGAAACTTATGTTATAACGATC
664157_30677280_HHV-7_JI_U43400.1_144861bp_1_1457	CCATTCAGTCCGATTACAGTAAATCCCAAGTAATGCTTCGAAACTTATGTTATAACGATCAGAGTCGTCACCGTAATACAATCTTAAGTTTTCAAAAAGTTGTTCAGCAGTTTGTGTTCT
664157_30677280_HHV-7_JI_U43400.1_144861bp_1_1458	AGAGTCGTCACCGTAATACAATCTTAAGTTTTCAAAAAGTTGTTCAGCAGTTTGTGTTCTGATATCATCAAACACGTTTGGAGAAACATCTAGTTTTGGGAAAATTTCAGCTGTGCGCCA
664157_30677280_HHV-7_JI_U43400.1_144861bp_1_1459	GATATCATCAAACACGTTTGGAGAAACATCTAGTTTTGGGAAAATTTCAGCTGTGCGCCAATTTTCCATGGTTAGTAATAATGATAGACTCAATATCTGAAGAAACTTTAATAGTAAAGA
664157_30677280_HHV-7_JI_U43400.1_144861bp_1_1460	ATTTTCCATGGTTAGTAATAATGATAGACTCAATATCTGAAGAAACTTTAATAGTAAAGAGCTACACCGTTAATCACTGTGCTAAAAATGTTCCAGTGTTTATTAACTCCTATGATTTAA
664157_30677280_HHV-7_JI_U43400.1_144861bp_1_1461	GCTACACCGTTAATCACTGTGCTAAAAATGTTCCAGTGTTTATTAACTCCTATGATTTAACCGCAGAAGTGGCCAAAAATGAAGACGTGCGATTAGCGCGACAAGTTCAAATTTCATTAG
664157_30677280_HHV-7_JI_U43400.1_144861bp_1_1462	CCGCAGAAGTGGCCAAAAATGAAGACGTGCGATTAGCGCGACAAGTTCAAATTTCATTAGAGAAAATAGATGAAGTTATAGAATCAATTTTTTCTGCGTCTGGTCCTAGCGTTGAAAATG
664157_30677280_HHV-7_JI_U43400.1_144861bp_1_1463	AGAAAATAGATGAAGTTATAGAATCAATTTTTTCTGCGTCTGGTCCTAGCGTTGAAAATGTAAAAGATCAGGCAAAGTTTGCTTTGTGTCGTTTACTGCTTGGTCCTGTGAGTATTCCGT
664157_30677280_HHV-7_JI_U43400.1_144861bp_1_1464	TAAAAGATCAGGCAAAGTTTGCTTTGTGTCGTTTACTGCTTGGTCCTGTGAGTATTCCGTGCTACTGCGAAGAATGGGATGTCAATTTTTATCTGACAAAATGTAGTTATAATTGCGAAG
664157_30677280_HHV-7_JI_U43400.1_144861bp_1_1465	GCTACTGCGAAGAATGGGATGTCAATTTTTATCTGACAAAATGTAGTTATAATTGCGAAGGCCCGGTTCTATATATCTATAAAAATGCTTCTCAATGCTGTGAAAGCACATATCGTTTTT

664157_30677280_HHV-7_JI_U43400.1_144861bp_1_1466	GCCCGGTTCTATATATCTATAAAAATGCTTCTCAATGCTGTGAAAGCACATATCGTTTTTCTATCATGACTAATTATCATTCCACTCACATCTTTAGAGGATTATTATCATTACAAGAAT
664157_30677280_HHV-7_JI_U43400.1_144861bp_1_1467	CTATCATGACTAATTATCATTCCACTCACATCTTTAGAGGATTATTATCATTACAAGAATGGAATAGTCATCTATCAAATATCTTATGTACTTGTTCGAACGTAACAGGTGATAAATATA
664157_30677280_HHV-7_JI_U43400.1_144861bp_1_1468	GGAATAGTCATCTATCAAATATCTTATGTACTTGTTCGAACGTAACAGGTGATAAATATACTGCAACAATCTTTCCAAACAATGCTTCAATTTACTTGGAATATTATCCGTATTTTCTAT
664157_30677280_HHV-7_JI_U43400.1_144861bp_1_1469	CTGCAACAATCTTTCCAAACAATGCTTCAATTTACTTGGAATATTATCCGTATTTTCTATGCTATCTATGCAAGCATCTGTCTATCATTGATATTGAGCAATGTACTAATGAATTGATAG
664157_30677280_HHV-7_JI_U43400.1_144861bp_1_1470	GCTATCTATGCAAGCATCTGTCTATCATTGATATTGAGCAATGTACTAATGAATTGATAGCTTTTCTTGGTCCAAAGACTTCTCAAAGGATTATAATTCACTATAAACTGTTATTCGGGT
664157_30677280_HHV-7_JI_U43400.1_144861bp_1_1471	CTTTTCTTGGTCCAAAGACTTCTCAAAGGATTATAATTCACTATAAACTGTTATTCGGGTTTCGATCTAAACCAATGAATTTCACTGTTTCTTTGTTAGAACAGGTTTTTACCCTTGAAA
664157_30677280_HHV-7_JI_U43400.1_144861bp_1_1472	TTCGATCTAAACCAATGAATTTCACTGTTTCTTTGTTAGAACAGGTTTTTACCCTTGAAATCCAAAAACTCTACTATTCCGTTAGTAAGCACAACAGTACAACAGCAGATTTTTTCAATG
664157_30677280_HHV-7_JI_U43400.1_144861bp_1_1473	TCCAAAAACTCTACTATTCCGTTAGTAAGCACAACAGTACAACAGCAGATTTTTTCAATGTCATTACCGCTAAATTTGCAGAGGACAAATATTTTGTTCTACGAACATTTAAATTGTCTG
664157_30677280_HHV-7_JI_U43400.1_144861bp_1_1474	TCATTACCGCTAAATTTGCAGAGGACAAATATTTTGTTCTACGAACATTTAAATTGTCTGCGCAAATCACTCCTGGTATTCAAAGTTTTTGTTCATTGAAATTCAAACTCCAGACCTTAT
664157_30677280_HHV-7_JI_U43400.1_144861bp_1_1475	CGCAAATCACTCCTGGTATTCAAAGTTTTTGTTCATTGAAATTCAAACTCCAGACCTTATATCTAAATTTGAAAATTATGAAAAACACAAAATTATCCATTTCTAATAGTTTTTATCATG
664157_30677280_HHV-7_JI_U43400.1_144861bp_1_1476	ATCTAAATTTGAAAATTATGAAAAACACAAAATTATCCATTTCTAATAGTTTTTATCATGGTAAAACTTTATATACACTGGATGAAAAGCAACTTGTTTGGAGAAATTTATTGTTAATTT
664157_30677280_HHV-7_JI_U43400.1_144861bp_1_1477	GTAAAACTTTATATACACTGGATGAAAAGCAACTTGTTTGGAGAAATTTATTGTTAATTTACTATGGTTACAATTTAAAAGACAATGTAAAACAAACACAAGAAGAGAGTTTGTTGTCGA

664157_30677280_HHV-7_JI_U43400.1_144861bp_1_1478	ACTATGGTTACAATTTAAAAGACAATGTAAAACAAACACAAGAAGAGAGTTTGTTGTCGATGCATTACATACGAATATTGGAAAGATTGTCTCTAAAAAGTTTTCGCGAAATTAATCAAC
664157_30677280_HHV-7_JI_U43400.1_144861bp_1_1479	TGCATTACATACGAATATTGGAAAGATTGTCTCTAAAAAGTTTTCGCGAAATTAATCAACAATTTAGCTTTGAAATTCCGAGTTACCAAGAGAAAACTTTGCAGTTTATTCCAGGTGGAA
664157_30677280_HHV-7_JI_U43400.1_144861bp_1_1480	AATTTAGCTTTGAAATTCCGAGTTACCAAGAGAAAACTTTGCAGTTTATTCCAGGTGGAAATGATTTCGCAGAAATCACATCGGTCACGCATGGAGAAACAACTGTGAATGCATTTAATA
664157_30677280_HHV-7_JI_U43400.1_144861bp_1_1481	ATGATTTCGCAGAAATCACATCGGTCACGCATGGAGAAACAACTGTGAATGCATTTAATACAAATAGGGTCATGAATGTGAAAGCTGCTCTTTCAGGAGAAATACACTGTGTTTTACATC
664157_30677280_HHV-7_JI_U43400.1_144861bp_1_1482	CAAATAGGGTCATGAATGTGAAAGCTGCTCTTTCAGGAGAAATACACTGTGTTTTACATCGTATTCCTAAAAGCATGACACATAGTTTTGTGATGTATAAGCGCACTTTTAAAGAACCCT
664157_30677280_HHV-7_JI_U43400.1_144861bp_1_1483	GTATTCCTAAAAGCATGACACATAGTTTTGTGATGTATAAGCGCACTTTTAAAGAACCCTCTTTAACAGTGAGTACCTTCATTTCAAATGATGATTTCACCACAAGTTCATTGAACATTA
664157_30677280_HHV-7_JI_U43400.1_144861bp_1_1484	CTTTAACAGTGAGTACCTTCATTTCAAATGATGATTTCACCACAAGTTCATTGAACATTAACATTCGAGGTCCCTACTGTGATTTTTTATATGCATTGGGTGTGTATAGATTACATGTTA
664157_30677280_HHV-7_JI_U43400.1_144861bp_1_1485	ACATTCGAGGTCCCTACTGTGATTTTTTATATGCATTGGGTGTGTATAGATTACATGTTAACATACAAGATTTTTTTTTACCTGCTTTTGTTTGTAACAGTAATAATTCGATGGATTTGC
664157_30677280_HHV-7_JI_U43400.1_144861bp_1_1486	ACATACAAGATTTTTTTTTACCTGCTTTTGTTTGTAACAGTAATAATTCGATGGATTTGCATGGGCTAGAAAATCAAGGAATTGTGCGAAAGCGTAAAAAGAAGGTTTACTGGATAACTA
664157_30677280_HHV-7_JI_U43400.1_144861bp_1_1487	ATGGGCTAGAAAATCAAGGAATTGTGCGAAAGCGTAAAAAGAAGGTTTACTGGATAACTAATTTTCCTTGTATGATCTCCAATTCTGAAAAAGTTAATGTTGGTTGGTTTAAAGCGGGAA
664157_30677280_HHV-7_JI_U43400.1_144861bp_1_1488	ATTTTCCTTGTATGATCTCCAATTCTGAAAAAGTTAATGTTGGTTGGTTTAAAGCGGGAACAGGCATAATACCCAAGGTTTCTGGAACAGACTTAAAAAATGTTCTTTTGAAAGAACTCA
664157_30677280_HHV-7_JI_U43400.1_144861bp_1_1489	CAGGCATAATACCCAAGGTTTCTGGAACAGACTTAAAAAATGTTCTTTTGAAAGAACTCATAAGCATCGGAGAAATTCCCAATATTACTTTTGATATGGATTTACATGCTTTGTTAACTC

664157_30677280_HHV-7_JI_U43400.1_144861bp_1_1490	TAAGCATCGGAGAAATTCCCAATATTACTTTTGATATGGATTTACATGCTTTGTTAACTCTTTTAGAGAAACGAAATATGCATCAGGTTCCATTTCTTATTAAACAATTTTTTATGTTTC
664157_30677280_HHV-7_JI_U43400.1_144861bp_1_1491	TTTTAGAGAAACGAAATATGCATCAGGTTCCATTTCTTATTAAACAATTTTTTATGTTTCTTCGTTTAGGTCTTTTAGTGGGATATGGACGTAAACAGGAAAGAAAGGTCCATCACATTA
664157_30677280_HHV-7_JI_U43400.1_144861bp_1_1492	TTCGTTTAGGTCTTTTAGTGGGATATGGACGTAAACAGGAAAGAAAGGTCCATCACATTATGCTATTCTTAATACAAAAGGGTTTTTTCGATTTTTCGAAGAATTCAGTTGCCAACAGTA
664157_30677280_HHV-7_JI_U43400.1_144861bp_1_1493	TGCTATTCTTAATACAAAAGGGTTTTTTCGATTTTTCGAAGAATTCAGTTGCCAACAGTAAAATTAAACATGCATGCGCTCTAGTTGGAAGTCGACTTGCCAACAATGTGCCAAAAATTC
664157_30677280_HHV-7_JI_U43400.1_144861bp_1_1494	AAATTAAACATGCATGCGCTCTAGTTGGAAGTCGACTTGCCAACAATGTGCCAAAAATTCTGTCTAAGCAGAAAAAAATGAAACTGGATCATTTGGGTCGAAACGCAAACGCTTTAACCG
664157_30677280_HHV-7_JI_U43400.1_144861bp_1_1495	TGTCTAAGCAGAAAAAAATGAAACTGGATCATTTGGGTCGAAACGCAAACGCTTTAACCGTTTTACGTTTTATTGTAGAAAATGGTTATTATAAAAGGAAGACAATTTTTCGCAAACTTT
664157_30677280_HHV-7_JI_U43400.1_144861bp_1_1496	TTTTACGTTTTATTGTAGAAAATGGTTATTATAAAAGGAAGACAATTTTTCGCAAACTTTTGAAGTATTTAGCTACTACATCTTTTAATGCTCATGTTCAAACTGAAAGTAATCGTTTAC
664157_30677280_HHV-7_JI_U43400.1_144861bp_1_1497	TGAAGTATTTAGCTACTACATCTTTTAATGCTCATGTTCAAACTGAAAGTAATCGTTTACTCAACTTGATGCACAATGACAGCAAAACAAATTTTTCCAGTTTGGAAAGACTGTACACTT
664157_30677280_HHV-7_JI_U43400.1_144861bp_1_1498	TCAACTTGATGCACAATGACAGCAAAACAAATTTTTCCAGTTTGGAAAGACTGTACACTTTACGTTAACAATGAGACAGCAACTGTGCATGAAATCTTGAATTCCGATTTAAGTGAACTG
664157_30677280_HHV-7_JI_U43400.1_144861bp_1_1499	TACGTTAACAATGAGACAGCAACTGTGCATGAAATCTTGAATTCCGATTTAAGTGAACTGTTACAGTTAAAGACGGAATTTGTATCTATGACAGACCTATGTGTTTACATTACTGGATGT
664157_30677280_HHV-7_JI_U43400.1_144861bp_1_1500	TTACAGTTAAAGACGGAATTTGTATCTATGACAGACCTATGTGTTTACATTACTGGATGTATAAATCAGAATATTTCCAGCATCACGATATATTGGCATGCTTACAGTGAAGTAATTTAT
664157_30677280_HHV-7_JI_U43400.1_144861bp_1_1501	ATAAATCAGAATATTTCCAGCATCACGATATATTGGCATGCTTACAGTGAAGTAATTTATGCTTTAACTGGAATTATACACTGTGAAAAGATTTCTATTGAATGTGGAATTAAATCCACG

664157_30677280_HHV-7_JI_U43400.1_144861bp_1_1502	GCTTTAACTGGAATTATACACTGTGAAAAGATTTCTATTGAATGTGGAATTAAATCCACGGACAATAACATTTTGTATGAAAAGCCCAAACTGTTTTTACTTCGAGAAAATTTAGCACCA
664157_30677280_HHV-7_JI_U43400.1_144861bp_1_1503	GACAATAACATTTTGTATGAAAAGCCCAAACTGTTTTTACTTCGAGAAAATTTAGCACCAACTGAATTAAGGTGGAAATCTTTAATAAAAACAAAGACTATAAAAAGTGCTCTGTCTCCA
664157_30677280_HHV-7_JI_U43400.1_144861bp_1_1504	ACTGAATTAAGGTGGAAATCTTTAATAAAAACAAAGACTATAAAAAGTGCTCTGTCTCCAAATCAAAATGAGATTTTTCCCAAAATAGCACACAAGCCGTCAATCCTTTTAGAGATTGAA
664157_30677280_HHV-7_JI_U43400.1_144861bp_1_1505	AATCAAAATGAGATTTTTCCCAAAATAGCACACAAGCCGTCAATCCTTTTAGAGATTGAAGAAGCACCCCGATTTAAGGAAATGGTGTCATGTATTTGGAAGTTAGTTGCCGAAGAGGCG
664157_30677280_HHV-7_JI_U43400.1_144861bp_1_1506	GAAGCACCCCGATTTAAGGAAATGGTGTCATGTATTTGGAAGTTAGTTGCCGAAGAGGCGACCATAACCTCAAAAAGCGAGAATGATATTGTCAAAACATGCAAAAAGCTTGCTGAATCA
664157_30677280_HHV-7_JI_U43400.1_144861bp_1_1507	ACCATAACCTCAAAAAGCGAGAATGATATTGTCAAAACATGCAAAAAGCTTGCTGAATCACAAAGATATACTTTGACAAATGGTACTGTGTTGCAAAATTTTATATTAGTCCATGCCTGC
664157_30677280_HHV-7_JI_U43400.1_144861bp_1_1508	CAAAGATATACTTTGACAAATGGTACTGTGTTGCAAAATTTTATATTAGTCCATGCCTGCTTATTTAAGCTTGGAGCTGTTAATTTTTGGGAGGAAATGAATGGAAAATTACGTCAACGG
664157_30677280_HHV-7_JI_U43400.1_144861bp_1_1509	TTATTTAAGCTTGGAGCTGTTAATTTTTGGGAGGAAATGAATGGAAAATTACGTCAACGGCCAGAACTAATGTCAAAATCATTCACTGGCCATGAGGAATGTTTCTATAATTGTTATTAT
664157_30677280_HHV-7_JI_U43400.1_144861bp_1_1510	CCAGAACTAATGTCAAAATCATTCACTGGCCATGAGGAATGTTTCTATAATTGTTATTATTTATGCACTTTGTTAAATTCCATTTATAGTTACAAAACTTTATTGCCAGAAATTGTAGAC
664157_30677280_HHV-7_JI_U43400.1_144861bp_1_1511	TTATGCACTTTGTTAAATTCCATTTATAGTTACAAAACTTTATTGCCAGAAATTGTAGACAATACCAGATCCATTCATGTAGTAGTGAAAGCATATTATTCAGAGCACATAGATGTTTCT
664157_30677280_HHV-7_JI_U43400.1_144861bp_1_1512	AATACCAGATCCATTCATGTAGTAGTGAAAGCATATTATTCAGAGCACATAGATGTTTCTTACAAAATTCTTTCGTACTCAACAAACATGATGAACTTATTCTCTCAGTATTTAAATTTT
664157_30677280_HHV-7_JI_U43400.1_144861bp_1_1513	TACAAAATTCTTTCGTACTCAACAAACATGATGAACTTATTCTCTCAGTATTTAAATTTTACAGATTTATTGCCATATATAAATAAGCACATTAAAATTGATGTTTCAGCATCTAAGCAA

664157_30677280_HHV-7_JI_U43400.1_144861bp_1_1514	ACAGATTTATTGCCATATATAAATAAGCACATTAAAATTGATGTTTCAGCATCTAAGCAAGATATGATTAAATTCTTAAATGCCTGTTTGGGACTTTAGATTTCTTTAAATGAAGTTTGT
664157_30677280_HHV-7_JI_U43400.1_144861bp_1_1515	GATATGATTAAATTCTTAAATGCCTGTTTGGGACTTTAGATTTCTTTAAATGAAGTTTGTTTACCTTCATGGCACATATAAATAGCCATTATAATAGAAATTAAAAGGTCATCTGAACAG
664157_30677280_HHV-7_JI_U43400.1_144861bp_1_1516	TTACCTTCATGGCACATATAAATAGCCATTATAATAGAAATTAAAAGGTCATCTGAACAGGTTTGTTTTTTGGCATTATAGGTTACGTGTTCATTTATATTAATTTGGTGAAGATTCTTA
664157_30677280_HHV-7_JI_U43400.1_144861bp_1_1517	GTTTGTTTTTTGGCATTATAGGTTACGTGTTCATTTATATTAATTTGGTGAAGATTCTTAATTTGTTCAATCACATACTCTATTGGGTCATACGTTATTTTTATTGTAAAGGAAATTAGT
664157_30677280_HHV-7_JI_U43400.1_144861bp_1_1518	ATTTGTTCAATCACATACTCTATTGGGTCATACGTTATTTTTATTGTAAAGGAAATTAGTTCTTGTGACGCTTTAATGTAACCTGAATTAAAATTCGAAATGAAATATTCTACGGCTAAT
664157_30677280_HHV-7_JI_U43400.1_144861bp_1_1519	TCTTGTGACGCTTTAATGTAACCTGAATTAAAATTCGAAATGAAATATTCTACGGCTAATCTCTTTTCTCTCCCCAGTAAATAAAATGGTTGTGCAATTTGGCTCTGATCTAAAGTATGG
664157_30677280_HHV-7_JI_U43400.1_144861bp_1_1520	CTCTTTTCTCTCCCCAGTAAATAAAATGGTTGTGCAATTTGGCTCTGATCTAAAGTATGGAAAAATGTTATGTGCTTATATCTTATAACCGATATAGTTTGTTTCAATATGCATGCAATT
664157_30677280_HHV-7_JI_U43400.1_144861bp_1_1521	AAAAATGTTATGTGCTTATATCTTATAACCGATATAGTTTGTTTCAATATGCATGCAATTTTCACCGCCGATGATTGATTGGAATTACCCTCGATAATAATTTTGAGCTCAGTAAAAAAA
664157_30677280_HHV-7_JI_U43400.1_144861bp_1_1522	TTCACCGCCGATGATTGATTGGAATTACCCTCGATAATAATTTTGAGCTCAGTAAAAAAAGGGTGTAATTCTAAAACAGCTAGTATCATATGTGAGGCACATTCAGCAATTGAAGCATCG
664157_30677280_HHV-7_JI_U43400.1_144861bp_1_1523	GGGTGTAATTCTAAAACAGCTAGTATCATATGTGAGGCACATTCAGCAATTGAAGCATCGGAGTTTGATAGTAAACTTTCTAGAAAATAGTGCTCCATTCCATATATTATATATTGATCA
664157_30677280_HHV-7_JI_U43400.1_144861bp_1_1524	GAGTTTGATAGTAAACTTTCTAGAAAATAGTGCTCCATTCCATATATTATATATTGATCACCATATGTTCCGATCGCTGCTACCCCTGTTCCAGAAGCACGTCGATTGATTGTGTATGCT
664157_30677280_HHV-7_JI_U43400.1_144861bp_1_1525	CCATATGTTCCGATCGCTGCTACCCCTGTTCCAGAAGCACGTCGATTGATTGTGTATGCTGGATCAATATATATGTAGAGCTCTTTGCCTAAGAATGGAATTATCTGTTTGCTAATTGTG

664157_30677280_HHV-7_JI_U43400.1_144861bp_1_1526	GGATCAATATATATGTAGAGCTCTTTGCCTAAGAATGGAATTATCTGTTTGCTAATTGTGCTATATCGGAAAAGGTCGAATTCAATGAGTCCTTGCTCTGTTATCAAGGTATCATTTACC
664157_30677280_HHV-7_JI_U43400.1_144861bp_1_1527	CTATATCGGAAAAGGTCGAATTCAATGAGTCCTTGCTCTGTTATCAAGGTATCATTTACCACATTGCAGAGAGAACCACCCATTATTTCGTGTTTGAAAGCACCTTCTAAAAATAGGTCG
664157_30677280_HHV-7_JI_U43400.1_144861bp_1_1528	ACATTGCAGAGAGAACCACCCATTATTTCGTGTTTGAAAGCACCTTCTAAAAATAGGTCGGCGGTTTTTTTGACGTCAGCGTTGATGCTGATAAATTTCGGTTTATGTAGTCGGTAACAT
664157_30677280_HHV-7_JI_U43400.1_144861bp_1_1529	GCGGTTTTTTTGACGTCAGCGTTGATGCTGATAAATTTCGGTTTATGTAGTCGGTAACATGCACAGGTTGTTGCATTTCCTCTGTCATTTAGTATGTGCACATGATCTTCACAAACATAA
664157_30677280_HHV-7_JI_U43400.1_144861bp_1_1530	GCACAGGTTGTTGCATTTCCTCTGTCATTTAGTATGTGCACATGATCTTCACAAACATAAGATACTACAGTTAACATTTCAAAGGGGGAATTGCTTAGTTTTGTCAAGAAGGAAGTAGAG
664157_30677280_HHV-7_JI_U43400.1_144861bp_1_1531	GATACTACAGTTAACATTTCAAAGGGGGAATTGCTTAGTTTTGTCAAGAAGGAAGTAGAGTGATTTCCGGAATTTGTGGATGATATAAAAATAATTTTAGTCGAAGATTGTGGCAGAAAT
664157_30677280_HHV-7_JI_U43400.1_144861bp_1_1532	TGATTTCCGGAATTTGTGGATGATATAAAAATAATTTTAGTCGAAGATTGTGGCAGAAATCCAAGAATTGTGCTAAACGCGTCTTTTTTGATAAAATGGCTTTCATCAACGATGAGAAGA
664157_30677280_HHV-7_JI_U43400.1_144861bp_1_1533	CCAAGAATTGTGCTAAACGCGTCTTTTTTGATAAAATGGCTTTCATCAACGATGAGAAGATTAAAGCTCTGACCACGTATACTCTGAGAAGAAAATGAACGGAGTTTTAAACGATATAAA
664157_30677280_HHV-7_JI_U43400.1_144861bp_1_1534	TTAAAGCTCTGACCACGTATACTCTGAGAAGAAAATGAACGGAGTTTTAAACGATATAAAGACTGAGTTTTTATGTAATACTAAAACAGATCTTTTAACGTTGATACAAAAAATTTGTCT
664157_30677280_HHV-7_JI_U43400.1_144861bp_1_1535	GACTGAGTTTTTATGTAATACTAAAACAGATCTTTTAACGTTGATACAAAAAATTTGTCTGAACTGTGATTTCATTCTTGAACCGGTAGAATCTTTTCCTAAAAAAACCGAGTTGGTTGC
664157_30677280_HHV-7_JI_U43400.1_144861bp_1_1536	GAACTGTGATTTCATTCTTGAACCGGTAGAATCTTTTCCTAAAAAAACCGAGTTGGTTGCGGTGATGTATGATACGCTCGCGGTGGAAATTTTTAACGATCTTTTAAAATATAATGAACA
664157_30677280_HHV-7_JI_U43400.1_144861bp_1_1537	GGTGATGTATGATACGCTCGCGGTGGAAATTTTTAACGATCTTTTAAAATATAATGAACAAAAAAAAAGATGGACTTGCCTAAATGTCAGTCAATCACAGTTGCTTGTGAAGGAGAATGTT

664157_30677280_HHV-7_JI_U43400.1_144861bp_1_1538	AAAAAAAGATGGACTTGCCTAAATGTCAGTCAATCACAGTTGCTTGTGAAGGAGAATGTTCGCAAATGTACAATTTGCATAATCCTCTGACATTTGAAATGGGTTTGGGAAACATCTTTA
664157_30677280_HHV-7_JI_U43400.1_144861bp_1_1539	CGCAAATGTACAATTTGCATAATCCTCTGACATTTGAAATGGGTTTGGGAAACATCTTTATATGTGTTCGGTGTTTTAAGATACATTTTTGCAATATGCTAGAAGACTGCAACCTGATAA
664157_30677280_HHV-7_JI_U43400.1_144861bp_1_1540	TATGTGTTCGGTGTTTTAAGATACATTTTTGCAATATGCTAGAAGACTGCAACCTGATAAATACTCATGAAGGATGCGTGTGTTCAAAAACCGGGCTTTTTTATAACGGATGGATGCCAG
664157_30677280_HHV-7_JI_U43400.1_144861bp_1_1541	ATACTCATGAAGGATGCGTGTGTTCAAAAACCGGGCTTTTTTATAACGGATGGATGCCAGCCTATTCACATACCTGTATGGAACCTACTGAAGAGCCAAATATGGAGACCGTTAATGTAG
664157_30677280_HHV-7_JI_U43400.1_144861bp_1_1542	CCTATTCACATACCTGTATGGAACCTACTGAAGAGCCAAATATGGAGACCGTTAATGTAGTTGTAGTGCTGTTATCATACGTTTACAGTTTTTTAATACAAAATAAAGCCAGGTATTCAA
664157_30677280_HHV-7_JI_U43400.1_144861bp_1_1543	TTGTAGTGCTGTTATCATACGTTTACAGTTTTTTAATACAAAATAAAGCCAGGTATTCAAACATTATTCGCGACATTATAAAAGATGGAAAGTTTATAGAACAAGTAGAAAATGCTGTTT
664157_30677280_HHV-7_JI_U43400.1_144861bp_1_1544	ACATTATTCGCGACATTATAAAAGATGGAAAGTTTATAGAACAAGTAGAAAATGCTGTTTTTTGTACATTTAATAAGGTGTTTAAGAACTCCACCTTGAATAAGTTACCTCTTACTACTG
664157_30677280_HHV-7_JI_U43400.1_144861bp_1_1545	TTTGTACATTTAATAAGGTGTTTAAGAACTCCACCTTGAATAAGTTACCTCTTACTACTGTCAGTCAACTTTTTGTTCAGTTAATAATTGGAGGCCACGCGGAAGGCACTATTTACGATA
664157_30677280_HHV-7_JI_U43400.1_144861bp_1_1546	TCAGTCAACTTTTTGTTCAGTTAATAATTGGAGGCCACGCGGAAGGCACTATTTACGATAATAATGTCATTCGTGTTAGTAGAAGAAAGAGGGAGGATAACATACTTAAAAAAATGAGGA
664157_30677280_HHV-7_JI_U43400.1_144861bp_1_1547	ATAATGTCATTCGTGTTAGTAGAAGAAAGAGGGAGGATAACATACTTAAAAAAATGAGGATTGAATATGGAAATGCACTTGCTCTGTGAAACAATGTTTACTTGCAGAAAAAATAATATT
664157_30677280_HHV-7_JI_U43400.1_144861bp_1_1548	TTGAATATGGAAATGCACTTGCTCTGTGAAACAATGTTTACTTGCAGAAAAAATAATATTTTACCGGTACATTTATGTATTTTACTGGATGATGTTATACATAAAGAGAAAGTAAAAGCT
664157_30677280_HHV-7_JI_U43400.1_144861bp_1_1549	TTACCGGTACATTTATGTATTTTACTGGATGATGTTATACATAAAGAGAAAGTAAAAGCTATAGAAGGGATCTTTTTTCAGTGTGTATTTTTTAAAGAAAAGCTTGTATATACGGAATGG

664157_30677280_HHV-7_JI_U43400.1_144861bp_1_1550	ATAGAAGGGATCTTTTTTCAGTGTGTATTTTTTAAAGAAAAGCTTGTATATACGGAATGGACAAAAATAAAGTTTACTTATGTGTTACATGATCTTGTAATTTCTCAAATCTTTAAGAAT
664157_30677280_HHV-7_JI_U43400.1_144861bp_1_1551	ACAAAAATAAAGTTTACTTATGTGTTACATGATCTTGTAATTTCTCAAATCTTTAAGAATGCCTGTATTAAAGAAGTAATACATGGGGCATTAATTCTTTCAGTTCCCATAAATATTGAT
664157_30677280_HHV-7_JI_U43400.1_144861bp_1_1552	GCCTGTATTAAAGAAGTAATACATGGGGCATTAATTCTTTCAGTTCCCATAAATATTGATAACCTACATTTTGATACAGATATTTTAATTCTAAAAATTATTTACCCACATTTTTTGCAC
664157_30677280_HHV-7_JI_U43400.1_144861bp_1_1553	AACCTACATTTTGATACAGATATTTTAATTCTAAAAATTATTTACCCACATTTTTTGCACGATGATATTGTCATAAAATTATCGGAAATTTTGTCTGGAGCACCTCGCATGCAAAAAACA
664157_30677280_HHV-7_JI_U43400.1_144861bp_1_1554	GATGATATTGTCATAAAATTATCGGAAATTTTGTCTGGAGCACCTCGCATGCAAAAAACAGTGGAAAAAAAACAAGAGGTGGAAAAACCTTTTTTCCATATTCCTGCAAAACTTGGAGAT
664157_30677280_HHV-7_JI_U43400.1_144861bp_1_1555	GTGGAAAAAAAACAAGAGGTGGAAAAACCTTTTTTCCATATTCCTGCAAAACTTGGAGATCTCACAAAGGAAGACCCTATTTCGTTCAACCATCATGGTCCGTTAGAACCTCCATCAACT
664157_30677280_HHV-7_JI_U43400.1_144861bp_1_1556	CTCACAAAGGAAGACCCTATTTCGTTCAACCATCATGGTCCGTTAGAACCTCCATCAACTGTTAGAGGATTAAAACAATCTGCGAACGTTAGGCATAGTCATCCAATATCAAGGCCTGAA
664157_30677280_HHV-7_JI_U43400.1_144861bp_1_1557	GTTAGAGGATTAAAACAATCTGCGAACGTTAGGCATAGTCATCCAATATCAAGGCCTGAAAAAGCGAACGTAACCTTTCTAAGTGATTCGTGGTACAGCCAAAATCTAAAGTGTGACTTC
664157_30677280_HHV-7_JI_U43400.1_144861bp_1_1558	AAAGCGAACGTAACCTTTCTAAGTGATTCGTGGTACAGCCAAAATCTAAAGTGTGACTTCATATCTGACATTCAACAAAGACATGTGCTTGTCATTTTTTGGTATGAGTTATCGAAAGGG
664157_30677280_HHV-7_JI_U43400.1_144861bp_1_1559	ATATCTGACATTCAACAAAGACATGTGCTTGTCATTTTTTGGTATGAGTTATCGAAAGGGATACAAATGCAAATTAAAAATATTCAAATTCCTCCTGAAAATTTGTTTTCATCAATAACG
664157_30677280_HHV-7_JI_U43400.1_144861bp_1_1560	ATACAAATGCAAATTAAAAATATTCAAATTCCTCCTGAAAATTTGTTTTCATCAATAACGAATTATTTAGATAGAGTCAACACATATCTAGACGAGATTGCTGAAAGAACTTTTCGATGT
664157_30677280_HHV-7_JI_U43400.1_144861bp_1_1561	AATTATTTAGATAGAGTCAACACATATCTAGACGAGATTGCTGAAAGAACTTTTCGATGTATTACTACTAACATGGAAATTCAGAATAGACATCTTCCACAAAAATTTAATAGTCATTTT

664157_30677280_HHV-7_JI_U43400.1_144861bp_1_1562	ATTACTACTAACATGGAAATTCAGAATAGACATCTTCCACAAAAATTTAATAGTCATTTTCAAATAGAGTTTAATTGTACTCACTTAATTTCTGGTATGGAATTGGCGAGGGATTTTTGG
664157_30677280_HHV-7_JI_U43400.1_144861bp_1_1563	CAAATAGAGTTTAATTGTACTCACTTAATTTCTGGTATGGAATTGGCGAGGGATTTTTGGATTTTGTCTTTAGATAGAAATAGTTGTGTTTTAAAAGCTATGGCCAGTCATTTTCTTCAT
664157_30677280_HHV-7_JI_U43400.1_144861bp_1_1564	ATTTTGTCTTTAGATAGAAATAGTTGTGTTTTAAAAGCTATGGCCAGTCATTTTCTTCATAAAAAAAAAGGGAGAAGCTCACTTAGTTCGAATGAATTTTGGGCTGACTTAATTGATTGC
664157_30677280_HHV-7_JI_U43400.1_144861bp_1_1565	AAAAAAAAAGGGAGAAGCTCACTTAGTTCGAATGAATTTTGGGCTGACTTAATTGATTGCACTACGGGAAAAACCTTATATGGAGAGAAAGTACGGTGGCAATTAAATTCTGAAACGAGC
664157_30677280_HHV-7_JI_U43400.1_144861bp_1_1566	ACTACGGGAAAAACCTTATATGGAGAGAAAGTACGGTGGCAATTAAATTCTGAAACGAGCTTATACTCCACATTCAGAAAAAATCAAAACATTTCATGGGAATTACAGCCTAATTGTTAT
664157_30677280_HHV-7_JI_U43400.1_144861bp_1_1567	TTATACTCCACATTCAGAAAAAATCAAAACATTTCATGGGAATTACAGCCTAATTGTTATGCACTATATATGTCTGAGAATTTAAAGCTGTATTGGGTATTACCCGGGGGGTTCTGCGTG
664157_30677280_HHV-7_JI_U43400.1_144861bp_1_1568	GCACTATATATGTCTGAGAATTTAAAGCTGTATTGGGTATTACCCGGGGGGTTCTGCGTGTCTGGAACTTTTAAATTAAAAGAGAACGATGAATTTTTCTTCGATTGGCAATTTGGGATG
664157_30677280_HHV-7_JI_U43400.1_144861bp_1_1569	TCTGGAACTTTTAAATTAAAAGAGAACGATGAATTTTTCTTCGATTGGCAATTTGGGATGTCTTAGGAGTTTTTTACAAAATGAGTGTAATTGGTTTTCGGTGTGTAAAAAAAAGTTGTA
664157_30677280_HHV-7_JI_U43400.1_144861bp_1_1570	TCTTAGGAGTTTTTTACAAAATGAGTGTAATTGGTTTTCGGTGTGTAAAAAAAAGTTGTACCACGAATATCGTTGTGTGGCAACATCTTCTCCTGTTTTCGCTGTTGATAAATTTAAAGA
664157_30677280_HHV-7_JI_U43400.1_144861bp_1_1571	CCACGAATATCGTTGTGTGGCAACATCTTCTCCTGTTTTCGCTGTTGATAAATTTAAAGATTGCTTGCACTGTAATATAATTATTTTAAAAAAGAATTTGGATTTTGTTTTTAGCTTGGC
664157_30677280_HHV-7_JI_U43400.1_144861bp_1_1572	TTGCTTGCACTGTAATATAATTATTTTAAAAAAGAATTTGGATTTTGTTTTTAGCTTGGCCATAAACGGAATACATGCCGGACAGTTTGCAACAAATTCCATTAAACTAAAAAAAAATTAT
664157_30677280_HHV-7_JI_U43400.1_144861bp_1_1573	CATAAACGGAATACATGCCGGACAGTTTGCAACAAATTCCATTAAACTAAAAAAAAATTATAATAACAAACGATTTGGTGTACTACATATTAGAATTGGGATCTTTAACGGTAACGGATTT

664157_30677280_HHV-7_JI_U43400.1_144861bp_1_1574	AATAACAAACGATTTGGTGTACTACATATTAGAATTGGGATCTTTAACGGTAACGGATTTACATTTCATTCCAAAATACAATAGTGAACATGTGCTGAATGTGCGACCTATTACGCCAAA
664157_30677280_HHV-7_JI_U43400.1_144861bp_1_1575	ACATTTCATTCCAAAATACAATAGTGAACATGTGCTGAATGTGCGACCTATTACGCCAAATCTAATTTATGATACTTGTTCAATTGTTAGTTATGACGAAGCTAAACTTTTAACTGTTAA
664157_30677280_HHV-7_JI_U43400.1_144861bp_1_1576	TCTAATTTATGATACTTGTTCAATTGTTAGTTATGACGAAGCTAAACTTTTAACTGTTAAAGGACCTGGAGAAAATAAATTAATTCCTTTGGGTTGTGGTTCTTGGTGTCTCAACAACAT
664157_30677280_HHV-7_JI_U43400.1_144861bp_1_1577	AGGACCTGGAGAAAATAAATTAATTCCTTTGGGTTGTGGTTCTTGGTGTCTCAACAACATGGGACGTTATTATGTGTACACTTTTGTCCTGGTGTACGATTTATATCTGGCTTGCTTTGA
664157_30677280_HHV-7_JI_U43400.1_144861bp_1_1578	GGGACGTTATTATGTGTACACTTTTGTCCTGGTGTACGATTTATATCTGGCTTGCTTTGAAAAAAACACTTTGCCATCATTATCTAAAGTAGTTTTTGATATGATTTCCTGCAATAATAA
664157_30677280_HHV-7_JI_U43400.1_144861bp_1_1579	AAAAAACACTTTGCCATCATTATCTAAAGTAGTTTTTGATATGATTTCCTGCAATAATAAACATTGTGTTTTTTGTAAGGATCATAGTAAACACGTAGAACAAACTGGCAAAACCGTTGG
664157_30677280_HHV-7_JI_U43400.1_144861bp_1_1580	ACATTGTGTTTTTTGTAAGGATCATAGTAAACACGTAGAACAAACTGGCAAAACCGTTGGATGCACTGATAATCAAGAAACATGTTTTTGTTACACCCCATGCAAGAAAAAAATGGCTAA
664157_30677280_HHV-7_JI_U43400.1_144861bp_1_1581	ATGCACTGATAATCAAGAAACATGTTTTTGTTACACCCCATGCAAGAAAAAAATGGCTAAGATTTCCAACCAAGATCTGTCTTCATTATTATGTGATCAAGAGCTCGATTTACTTGATCT
664157_30677280_HHV-7_JI_U43400.1_144861bp_1_1582	GATTTCCAACCAAGATCTGTCTTCATTATTATGTGATCAAGAGCTCGATTTACTTGATCTAATTTACCCTGAAAAGCCCACTAGTCTTTCAACTGATATAAATGCTTACGTACATGGACA
664157_30677280_HHV-7_JI_U43400.1_144861bp_1_1583	AATTTACCCTGAAAAGCCCACTAGTCTTTCAACTGATATAAATGCTTACGTACATGGACATAAGAATCAAGAACCAGTCGTTTTAAGGAATACAAACTGGATATTAATTCGTCTTGACCC
664157_30677280_HHV-7_JI_U43400.1_144861bp_1_1584	TAAGAATCAAGAACCAGTCGTTTTAAGGAATACAAACTGGATATTAATTCGTCTTGACCCAGCAATTAGCAGATTAATACTCCTTTCCTGTCCAGTCTGTAAACGCATAGTAAGTAGGTA
664157_30677280_HHV-7_JI_U43400.1_144861bp_1_1585	AGCAATTAGCAGATTAATACTCCTTTCCTGTCCAGTCTGTAAACGCATAGTAAGTAGGTAAGAAAAATTTGTTACTTACGTGAGTGTTGTAACAACTAGCAAACAGTGCTGTACTTTTTA

664157_30677280_HHV-7_JI_U43400.1_144861bp_1_1586	AGAAAAATTTGTTACTTACGTGAGTGTTGTAACAACTAGCAAACAGTGCTGTACTTTTTATTGTTTCGTGTTCGATAGTAATCACATTATCTTGACAGGTGATATTCTTTTGCGGGAAAA
664157_30677280_HHV-7_JI_U43400.1_144861bp_1_1587	TTGTTTCGTGTTCGATAGTAATCACATTATCTTGACAGGTGATATTCTTTTGCGGGAAAAAACGTCTACATTTAAATTCAACATCTTTCATAACAAAGTGCGAAACGTGTTTTTGATGTG
664157_30677280_HHV-7_JI_U43400.1_144861bp_1_1588	AACGTCTACATTTAAATTCAACATCTTTCATAACAAAGTGCGAAACGTGTTTTTGATGTGCAACATAACCAATGCTGATTCCTTCTAAATTCTTTAATAAAAAACAAATAACTGGTATCA
664157_30677280_HHV-7_JI_U43400.1_144861bp_1_1589	CAACATAACCAATGCTGATTCCTTCTAAATTCTTTAATAAAAAACAAATAACTGGTATCATGAACCATGTTTTACCGTGTCTTCTGGGAACTAGAAAAACACTAGCTTTCTGTTTAAGTA
664157_30677280_HHV-7_JI_U43400.1_144861bp_1_1590	TGAACCATGTTTTACCGTGTCTTCTGGGAACTAGAAAAACACTAGCTTTCTGTTTAAGTATGTTTACGCTGCTTTCGTTTATAAACTCTATGTCAAATTTATATTTTAAGTAATCAAGGA
664157_30677280_HHV-7_JI_U43400.1_144861bp_1_1591	TGTTTACGCTGCTTTCGTTTATAAACTCTATGTCAAATTTATATTTTAAGTAATCAAGGACATGATTGGCCAGTGTGGGTAATTTCGTAACAGAGATGAAAAAAATTATGTGTATAAGAA
664157_30677280_HHV-7_JI_U43400.1_144861bp_1_1592	CATGATTGGCCAGTGTGGGTAATTTCGTAACAGAGATGAAAAAAATTATGTGTATAAGAATGCTCTTTTGAAATGGCTCTAGTTTAATTCTTTCCTTTTTATTGGTGTTCTCAAAATCAC
664157_30677280_HHV-7_JI_U43400.1_144861bp_1_1593	TGCTCTTTTGAAATGGCTCTAGTTTAATTCTTTCCTTTTTATTGGTGTTCTCAAAATCACCGCAGATCCATCTTTGAAAATCTTGAATAAATTTTTCGATCTGTAAAAACATGGGATTTC
664157_30677280_HHV-7_JI_U43400.1_144861bp_1_1594	CGCAGATCCATCTTTGAAAATCTTGAATAAATTTTTCGATCTGTAAAAACATGGGATTTCTGTAAAAACTTGTTTGTAGCTCTAGATGTTTTTGATATTGTACTTCTGTGTTTTTCTCTA
664157_30677280_HHV-7_JI_U43400.1_144861bp_1_1595	TGTAAAAACTTGTTTGTAGCTCTAGATGTTTTTGATATTGTACTTCTGTGTTTTTCTCTATGATTGCAGTAAATTTGAGCTTTGACAAAGCGCAGTTCAAGGGGTCGCATATTGCAATTT
664157_30677280_HHV-7_JI_U43400.1_144861bp_1_1596	TGATTGCAGTAAATTTGAGCTTTGACAAAGCGCAGTTCAAGGGGTCGCATATTGCAATTTTAGATTTTACGTGCCGTTGGCGATCACAAAAGAGATATAAAGGTTTAACATGCCTGCAAT
664157_30677280_HHV-7_JI_U43400.1_144861bp_1_1597	TAGATTTTACGTGCCGTTGGCGATCACAAAAGAGATATAAAGGTTTAACATGCCTGCAATATGCATGTGTAAAGCCAAGTTCTGGAGTTAATATAATAAATCGTTTTTGGCAAAATATCG

664157_30677280_HHV-7_JI_U43400.1_144861bp_1_1598	ATGCATGTGTAAAGCCAAGTTCTGGAGTTAATATAATAAATCGTTTTTGGCAAAATATCGCGCTGTTTGGAAAAGTTGAAGAAATCTTTACGTCCTGTTCATGTTTCCAAATTATAGACT
664157_30677280_HHV-7_JI_U43400.1_144861bp_1_1599	CGCTGTTTGGAAAAGTTGAAGAAATCTTTACGTCCTGTTCATGTTTCCAAATTATAGACTGATACGCTTTCTGAATTGCATCTATATCGCATGATCGCAACATGGACACAGATATTGCTC
664157_30677280_HHV-7_JI_U43400.1_144861bp_1_1600	GATACGCTTTCTGAATTGCATCTATATCGCATGATCGCAACATGGACACAGATATTGCTCTAGCTGCAATTTATAAAGAAACGACTAAATTAAATGAAAAGGATGCTAAAATTTTCTCGG
664157_30677280_HHV-7_JI_U43400.1_144861bp_1_1601	TAGCTGCAATTTATAAAGAAACGACTAAATTAAATGAAAAGGATGCTAAAATTTTCTCGGAGGCAGTGCAGACCGCACTAACTGTGTGTAAAGCAACCGCTCCTAATACACGTCTAAAAC
664157_30677280_HHV-7_JI_U43400.1_144861bp_1_1602	AGGCAGTGCAGACCGCACTAACTGTGTGTAAAGCAACCGCTCCTAATACACGTCTAAAACTCGTTGAAACACCAACTAATAACTTCTTACTAGTAACAAATGTTGTTCCATCAGAAACTT
664157_30677280_HHV-7_JI_U43400.1_144861bp_1_1603	TCGTTGAAACACCAACTAATAACTTCTTACTAGTAACAAATGTTGTTCCATCAGAAACTTCGAAAGCAACGACTGAAGCAAATCTTAATATTGATGCAGCGTTGGAAAAACTGGCGTCTT
664157_30677280_HHV-7_JI_U43400.1_144861bp_1_1604	CGAAAGCAACGACTGAAGCAAATCTTAATATTGATGCAGCGTTGGAAAAACTGGCGTCTTCCTTTAATACAGCGGTACCTGTAAAATCATCCAAAAAGTATTTGTTGCAAAATGTGAGAA
664157_30677280_HHV-7_JI_U43400.1_144861bp_1_1605	CCTTTAATACAGCGGTACCTGTAAAATCATCCAAAAAGTATTTGTTGCAAAATGTGAGAAAAATGACCAGTGAGAACATCGCTCTAACTGGATCATATATCATCTATACGAAAAAACACA
664157_30677280_HHV-7_JI_U43400.1_144861bp_1_1606	AAATGACCAGTGAGAACATCGCTCTAACTGGATCATATATCATCTATACGAAAAAACACATCGAGGTGGCGTTTCTGTTAGATAAGTCTGATTTTGTTCAGGATATTTTACGTTATGCTG
664157_30677280_HHV-7_JI_U43400.1_144861bp_1_1607	TCGAGGTGGCGTTTCTGTTAGATAAGTCTGATTTTGTTCAGGATATTTTACGTTATGCTGAAACACCCAGTCTTCTAGGACATACCGATGTACGTGATTTAGAATGTTTGTTATGGTTAG
664157_30677280_HHV-7_JI_U43400.1_144861bp_1_1608	AAACACCCAGTCTTCTAGGACATACCGATGTACGTGATTTAGAATGTTTGTTATGGTTAGCTTTTTGTGGTCCTATGAGTTATTGTCAGGCTGATAATTGTTTTGGACTAAATAAGGCGG
664157_30677280_HHV-7_JI_U43400.1_144861bp_1_1609	CTTTTTGTGGTCCTATGAGTTATTGTCAGGCTGATAATTGTTTTGGACTAAATAAGGCGGGGTATAACGCCCCTTTCCCAATATTGTTTCCACCATGCATGTATGAAAGAAATATGAACC

664157_30677280_HHV-7_JI_U43400.1_144861bp_1_1610	GGTATAACGCCCCTTTCCCAATATTGTTTCCACCATGCATGTATGAAAGAAATATGAACCTTAGTGTATTTTTTGGGTTATTGCAAATTTATGTGTTCTCTTTGTATAGAGATTTTAGTG
664157_30677280_HHV-7_JI_U43400.1_144861bp_1_1611	TTAGTGTATTTTTTGGGTTATTGCAAATTTATGTGTTCTCTTTGTATAGAGATTTTAGTGTCGAAAATTCAAATTTACAGCAAGGTATTAAAAAGCGTATTAAGTTGGTTCTGTCAGATT
664157_30677280_HHV-7_JI_U43400.1_144861bp_1_1612	TCGAAAATTCAAATTTACAGCAAGGTATTAAAAAGCGTATTAAGTTGGTTCTGTCAGATTTACGGGCCAAAGAAAGAATTTGTGAGGAAGAGATAGGAAATTTTCCATTGGCGGCTCAAA
664157_30677280_HHV-7_JI_U43400.1_144861bp_1_1613	TACGGGCCAAAGAAAGAATTTGTGAGGAAGAGATAGGAAATTTTCCATTGGCGGCTCAAATATGCCTGTTTTGTGCATTATATAGACAAAATAGGCTCTGTATGGAATATGCCGCAAACA
664157_30677280_HHV-7_JI_U43400.1_144861bp_1_1614	TATGCCTGTTTTGTGCATTATATAGACAAAATAGGCTCTGTATGGAATATGCCGCAAACAATCTAAGTATGAGTGTGTTCAGTCCAATAATATTAAAGGACTGTACATTTATGCAAACAA
664157_30677280_HHV-7_JI_U43400.1_144861bp_1_1615	ATCTAAGTATGAGTGTGTTCAGTCCAATAATATTAAAGGACTGTACATTTATGCAAACAACAGTTACCATAACTCAAATCTTGCCAGGTTCTAAGGAAGCAATAATTTTTCCAGTTTACG
664157_30677280_HHV-7_JI_U43400.1_144861bp_1_1616	CAGTTACCATAACTCAAATCTTGCCAGGTTCTAAGGAAGCAATAATTTTTCCAGTTTACGATATAGGCAAATTACTATCAGCTCTTGTTTTTTCAGAGAACGGTGTACTTTTGAAACTAT
664157_30677280_HHV-7_JI_U43400.1_144861bp_1_1617	ATATAGGCAAATTACTATCAGCTCTTGTTTTTTCAGAGAACGGTGTACTTTTGAAACTATAATGTCACTGCATGAATTAATAAAACAAACTATGTCCAAAAATTTAGAAAAAAAACATTA
664157_30677280_HHV-7_JI_U43400.1_144861bp_1_1618	AATGTCACTGCATGAATTAATAAAACAAACTATGTCCAAAAATTTAGAAAAAAAACATTATGAGTTGTTAAAATTAAAACTTGGTGAAGATCATCCTCTTAGCGTTCGACAGCAAATTCA
664157_30677280_HHV-7_JI_U43400.1_144861bp_1_1619	TGAGTTGTTAAAATTAAAACTTGGTGAAGATCATCCTCTTAGCGTTCGACAGCAAATTCACGCTCTCAATCAAAATCTTGTATCAGAAAATCTCGAACAGTCCCAGATAATTACTTCTTT
664157_30677280_HHV-7_JI_U43400.1_144861bp_1_1620	CGCTCTCAATCAAAATCTTGTATCAGAAAATCTCGAACAGTCCCAGATAATTACTTCTTTGACAAAAATGTTAAAGGATCAAAAGCTGCAGCTGAAAGCGCAAAGGAAAAATGCTGCTCA
664157_30677280_HHV-7_JI_U43400.1_144861bp_1_1621	GACAAAAATGTTAAAGGATCAAAAGCTGCAGCTGAAAGCGCAAAGGAAAAATGCTGCTCAGCTAGAATGTGTAGATTTGGATGACATTTTGGATACGGCAGCGGAAGTGAAATCCGTCAC

664157_30677280_HHV-7_JI_U43400.1_144861bp_1_1622	GCTAGAATGTGTAGATTTGGATGACATTTTGGATACGGCAGCGGAAGTGAAATCCGTCACCGACAATATAAAAGAAACTTTACTGGCCGGATTAGAATCAGACTAAATATGGAGCAGCTT
664157_30677280_HHV-7_JI_U43400.1_144861bp_1_1623	CGACAATATAAAAGAAACTTTACTGGCCGGATTAGAATCAGACTAAATATGGAGCAGCTTAAGACACCCCAAAATCAAAAAACACGTCCAAGAAATATGCTTCCTAAAAAAAAAGGAAAA
664157_30677280_HHV-7_JI_U43400.1_144861bp_1_1624	AAGACACCCCAAAATCAAAAAACACGTCCAAGAAATATGCTTCCTAAAAAAAAAGGAAAAGAACTTAAAAAAAGGCCTTGTAAAGTAAAACGTAAATTATTTGGTTCCGAAAACATCAGA
664157_30677280_HHV-7_JI_U43400.1_144861bp_1_1625	GAACTTAAAAAAAGGCCTTGTAAAGTAAAACGTAAATTATTTGGTTCCGAAAACATCAGACCTAACAAAAAAATACCTCTGGCTTCAGACGTGGATAACGAATTGGAAAAAAAACGGGGC
664157_30677280_HHV-7_JI_U43400.1_144861bp_1_1626	CCTAACAAAAAAATACCTCTGGCTTCAGACGTGGATAACGAATTGGAAAAAAAACGGGGCTCGATGATACGAAAACGGTCTGAGACGGACTTATGTCCAGATCCATCTGTAACAGACCTC
664157_30677280_HHV-7_JI_U43400.1_144861bp_1_1627	TCGATGATACGAAAACGGTCTGAGACGGACTTATGTCCAGATCCATCTGTAACAGACCTCCTATGTCATGAATCTTTGACTGTATCTCCAAAGTTTGAACGAGATGGATTGAGTGCATGC
664157_30677280_HHV-7_JI_U43400.1_144861bp_1_1628	CTATGTCATGAATCTTTGACTGTATCTCCAAAGTTTGAACGAGATGGATTGAGTGCATGCACGGAATTTGAGAATTTTATGGATACAAGGAAAATCGTGTTAAGTCGAAATGAAAAGTCT
664157_30677280_HHV-7_JI_U43400.1_144861bp_1_1629	ACGGAATTTGAGAATTTTATGGATACAAGGAAAATCGTGTTAAGTCGAAATGAAAAGTCTGTGACAGATTTAAGTGCACATTACCCCGTTTTATGTAATCTTGGAATTTTTGAGCGTATT
664157_30677280_HHV-7_JI_U43400.1_144861bp_1_1630	GTGACAGATTTAAGTGCACATTACCCCGTTTTATGTAATCTTGGAATTTTTGAGCGTATTCATTCACCCTTTTTGTTTTCAATACACATTGATACTCAGTCATTTTCAGTTGTCTATGTT
664157_30677280_HHV-7_JI_U43400.1_144861bp_1_1631	CATTCACCCTTTTTGTTTTCAATACACATTGATACTCAGTCATTTTCAGTTGTCTATGTTCCACATAAGGAAAGTTCCTGTTCTCAGTTTTGCGAGCCAGAAAAAAACATGGCACGGATT
664157_30677280_HHV-7_JI_U43400.1_144861bp_1_1632	CCACATAAGGAAAGTTCCTGTTCTCAGTTTTGCGAGCCAGAAAAAAACATGGCACGGATTTTAGGAAGCGGATCATATGGAATGGTATATGATTTGAACAATGTTGCAATTAAAGCTTCT
664157_30677280_HHV-7_JI_U43400.1_144861bp_1_1633	TTAGGAAGCGGATCATATGGAATGGTATATGATTTGAACAATGTTGCAATTAAAGCTTCTGATGACTTAGAGAGCTGCATTTCTTCTTATGTGTCTGGAGTAGTTCGTGCAAAAGCCGGA

664157_30677280_HHV-7_JI_U43400.1_144861bp_1_1634	GATGACTTAGAGAGCTGCATTTCTTCTTATGTGTCTGGAGTAGTTCGTGCAAAAGCCGGAGCTCAATTAACCTCACGCGAATGCGTGTTTAAAAGTCTTTTGATATGTAATTCTGTCTGC
664157_30677280_HHV-7_JI_U43400.1_144861bp_1_1635	GCTCAATTAACCTCACGCGAATGCGTGTTTAAAAGTCTTTTGATATGTAATTCTGTCTGCCTGAACCATAAAATCTCCCTTTCCAAAACTTATGATACAGATTTATATAAATTTACAGAC
664157_30677280_HHV-7_JI_U43400.1_144861bp_1_1636	CTGAACCATAAAATCTCCCTTTCCAAAACTTATGATACAGATTTATATAAATTTACAGACTGGAAATTGGAAAACGTTGAAAATTATTACTCTATTTTTTGCAACCTTGCAGAAGCTGTT
664157_30677280_HHV-7_JI_U43400.1_144861bp_1_1637	TGGAAATTGGAAAACGTTGAAAATTATTACTCTATTTTTTGCAACCTTGCAGAAGCTGTTCGTTTTTTAAACATGGTGTGTAAAATCAACCATTGTGATATTTCACTAGCAAATATTTGA
664157_30677280_HHV-7_JI_U43400.1_144861bp_1_1638	CGTTTTTTAAACATGGTGTGTAAAATCAACCATTGTGATATTTCACTAGCAAATATTTGATACACCACAAGGAAGGTATTATTTTGGAGGCTGTGTTAGCTGATTACAGTTTAGCTGAAG
664157_30677280_HHV-7_JI_U43400.1_144861bp_1_1639	TACACCACAAGGAAGGTATTATTTTGGAGGCTGTGTTAGCTGATTACAGTTTAGCTGAAGTACACCCACAGTATAATGGAAAATGTGGAATACTAAGACAATTTGATCATAGGATCCAGA
664157_30677280_HHV-7_JI_U43400.1_144861bp_1_1640	TACACCCACAGTATAATGGAAAATGTGGAATACTAAGACAATTTGATCATAGGATCCAGATTGTGCCTAAAAGTTATAATAAATTGTGTGACATGTTTAATCCAGGTTTCAGACCCATGA
664157_30677280_HHV-7_JI_U43400.1_144861bp_1_1641	TTGTGCCTAAAAGTTATAATAAATTGTGTGACATGTTTAATCCAGGTTTCAGACCCATGATAGCTCACAAAATAATTTTGGTCGAAGTTTATGCAGAATTTGATGGTAAGGGCAATCCAG
664157_30677280_HHV-7_JI_U43400.1_144861bp_1_1642	TAGCTCACAAAATAATTTTGGTCGAAGTTTATGCAGAATTTGATGGTAAGGGCAATCCAGTGAGACATTGTAATCTAGATCTTTGTGCACTAGCGCAAGTATTTTTATTATGTGTCATCA
664157_30677280_HHV-7_JI_U43400.1_144861bp_1_1643	TGAGACATTGTAATCTAGATCTTTGTGCACTAGCGCAAGTATTTTTATTATGTGTCATCAGAATGTTGGATGAACGCGGATGCCGTGAGGCGCAAAAATATTATGAAAATCGATTGTTCA
664157_30677280_HHV-7_JI_U43400.1_144861bp_1_1644	GAATGTTGGATGAACGCGGATGCCGTGAGGCGCAAAAATATTATGAAAATCGATTGTTCACGTACTCAAATGAAGCTTGTACTTTGAATCCAATCAAATACCCTTTAGAATATAAAGATG
664157_30677280_HHV-7_JI_U43400.1_144861bp_1_1645	CGTACTCAAATGAAGCTTGTACTTTGAATCCAATCAAATACCCTTTAGAATATAAAGATGCTTGTTGCAAAGTTTTAGCTGAGCACTTAGTTTTATTTGGCATTCTTTTTTATCGTGAGG

664157_30677280_HHV-7_JI_U43400.1_144861bp_1_1646	CTTGTTGCAAAGTTTTAGCTGAGCACTTAGTTTTATTTGGCATTCTTTTTTATCGTGAGGTGGTGGATATGTTTGAAAACTTGTATGACTTTCTGCACGCAAGTGGTGATTTAAGCGTAA
664157_30677280_HHV-7_JI_U43400.1_144861bp_1_1647	TGGTGGATATGTTTGAAAACTTGTATGACTTTCTGCACGCAAGTGGTGATTTAAGCGTAAGAGATTTACTCGAGGAAACATATGTAAATGACAGTAGAGATGTTAGAAGACAACCAATCA
664157_30677280_HHV-7_JI_U43400.1_144861bp_1_1648	GAGATTTACTCGAGGAAACATATGTAAATGACAGTAGAGATGTTAGAAGACAACCAATCAGGTATAGGCACGCCCAATTACAAAGACACGAAATTGGTCAAATACTTTTAAATGATCTGC
664157_30677280_HHV-7_JI_U43400.1_144861bp_1_1649	GGTATAGGCACGCCCAATTACAAAGACACGAAATTGGTCAAATACTTTTAAATGATCTGCAACAATTGCTTTCCATTATAACTATTTCAGATTTAGAGAAGGATCCATATTCTGTATTTC
664157_30677280_HHV-7_JI_U43400.1_144861bp_1_1650	AACAATTGCTTTCCATTATAACTATTTCAGATTTAGAGAAGGATCCATATTCTGTATTTCGGGTGTAACATGGCAATAGATTACGCACAAATTTCTTGTAATTTGGCTTCTATTATAGAA
664157_30677280_HHV-7_JI_U43400.1_144861bp_1_1651	GGGTGTAACATGGCAATAGATTACGCACAAATTTCTTGTAATTTGGCTTCTATTATAGAAGAGGACTCGGTCTTTTTATTTCTAATAGACAAATTAAACAATCTGGACATTTCAAGAAGG
664157_30677280_HHV-7_JI_U43400.1_144861bp_1_1652	GAGGACTCGGTCTTTTTATTTCTAATAGACAAATTAAACAATCTGGACATTTCAAGAAGGAAAATTTCATTTAATTTTATCCGTCTGTGTTATACTTATTATATCTTAATAAAGTTTAAT
664157_30677280_HHV-7_JI_U43400.1_144861bp_1_1653	AAAATTTCATTTAATTTTATCCGTCTGTGTTATACTTATTATATCTTAATAAAGTTTAATTCTCGCTTCAAAGATACCTTCTTAGCCAGATCGTTCATTGATTATATGCATCAAAACATA
664157_30677280_HHV-7_JI_U43400.1_144861bp_1_1654	TCTCGCTTCAAAGATACCTTCTTAGCCAGATCGTTCATTGATTATATGCATCAAAACATATCAGATTTTATCGATGAGAATGTTGAGCTATCTGATTTATATAGCAATATTTATGTCCGC
664157_30677280_HHV-7_JI_U43400.1_144861bp_1_1655	TCAGATTTTATCGATGAGAATGTTGAGCTATCTGATTTATATAGCAATATTTATGTCCGCTTACAAGATGCGAGTCCAAAAGTTGTTAAGAATCTATTTAAAATATTAGAACGAGAGACA
664157_30677280_HHV-7_JI_U43400.1_144861bp_1_1656	TTACAAGATGCGAGTCCAAAAGTTGTTAAGAATCTATTTAAAATATTAGAACGAGAGACAAGAGGACAGTCAACAAATCCACTCTGGCACGCTATGCGAAAAAATTGCATCACGGCAACT
664157_30677280_HHV-7_JI_U43400.1_144861bp_1_1657	AGAGGACAGTCAACAAATCCACTCTGGCACGCTATGCGAAAAAATTGCATCACGGCAACTAAAATTTATGACATCTATATCTCTAAATCTTTTTCGGGTATACAGGAGCATTCTTATTTA

664157_30677280_HHV-7_JI_U43400.1_144861bp_1_1658	AAAATTTATGACATCTATATCTCTAAATCTTTTTCGGGTATACAGGAGCATTCTTATTTAGGAGATGCGGTTTTATATGGAATTAAACATGAACGCATCATAGAACACCTGTTAAAGACA
664157_30677280_HHV-7_JI_U43400.1_144861bp_1_1659	GGAGATGCGGTTTTATATGGAATTAAACATGAACGCATCATAGAACACCTGTTAAAGACATTCTTTGTGAAAAAGCCCTGGATATCTAAAACACTTGGTTTATTATTAGATCCTTCATCT
664157_30677280_HHV-7_JI_U43400.1_144861bp_1_1660	TTCTTTGTGAAAAAGCCCTGGATATCTAAAACACTTGGTTTATTATTAGATCCTTCATCTGGAGTGTTTGGTGCATCCATAGATTCTTATTATGGAATCTCTTTTAATGACAACAACCTG
664157_30677280_HHV-7_JI_U43400.1_144861bp_1_1661	GGAGTGTTTGGTGCATCCATAGATTCTTATTATGGAATCTCTTTTAATGACAACAACCTGATAGAAGTAGGGGATAAAGTTGTTATATTTGAATTGAAATTTAGATATAAATATCTGAGA
664157_30677280_HHV-7_JI_U43400.1_144861bp_1_1662	ATAGAAGTAGGGGATAAAGTTGTTATATTTGAATTGAAATTTAGATATAAATATCTGAGAGAAAAAAACGATCTATTTGTTTCCGAGCTGTTACAAAATCCGTCAGAAATTGCTTTAGCT
664157_30677280_HHV-7_JI_U43400.1_144861bp_1_1663	GAAAAAAACGATCTATTTGTTTCCGAGCTGTTACAAAATCCGTCAGAAATTGCTTTAGCTAAATTCATCTTATCACATCCAATACCAGCTATAGAGTATAGAGAAAATGGAAAGATGCCC
664157_30677280_HHV-7_JI_U43400.1_144861bp_1_1664	AAATTCATCTTATCACATCCAATACCAGCTATAGAGTATAGAGAAAATGGAAAGATGCCCTCGGCAAGAGAATATTTAATCACTAACAATCCTCTATACGATTCTGGTAAAAAACGTCGT
664157_30677280_HHV-7_JI_U43400.1_144861bp_1_1665	TCGGCAAGAGAATATTTAATCACTAACAATCCTCTATACGATTCTGGTAAAAAACGTCGTGCTTGCTTGACTCCCAAAAATTTGACGTTCGACATTACACGACTAATTCCCATGAACGAA
664157_30677280_HHV-7_JI_U43400.1_144861bp_1_1666	GCTTGCTTGACTCCCAAAAATTTGACGTTCGACATTACACGACTAATTCCCATGAACGAAAAAAAATGTGTCAACGGCAATTATTTTTGATGTCGTTAAAGACTGTATATTAAACACATTG
664157_30677280_HHV-7_JI_U43400.1_144861bp_1_1667	AAAAATGTGTCAACGGCAATTATTTTTGATGTCGTTAAAGACTGTATATTAAACACATTGGTTGCATACCAAAAAGCTATTTTTACTATTGATGCTTTTATAAATCCTAGGCATAGATAT
664157_30677280_HHV-7_JI_U43400.1_144861bp_1_1668	GTTGCATACCAAAAAGCTATTTTTACTATTGATGCTTTTATAAATCCTAGGCATAGATATTATTTTCAGAGTATTTTACAGCAATATGTAATGACTCAATTTTATATACAGGATCACGAT
664157_30677280_HHV-7_JI_U43400.1_144861bp_1_1669	TATTTTCAGAGTATTTTACAGCAATATGTAATGACTCAATTTTATATACAGGATCACGATAATCCAGAAAATATTGAAAAAGAGAATTTACCCTCGGTTTATATTGTATCTGCCATATTT

664157_30677280_HHV-7_JI_U43400.1_144861bp_1_1670	AATCCAGAAAATATTGAAAAAGAGAATTTACCCTCGGTTTATATTGTATCTGCCATATTTCGAAAAAGAGAAGACGACGAAAAAAACTGTCGCTTGCTAATTGAGGACACAGAATATTTA
664157_30677280_HHV-7_JI_U43400.1_144861bp_1_1671	CGAAAAAGAGAAGACGACGAAAAAAACTGTCGCTTGCTAATTGAGGACACAGAATATTTAGAAGAAGAAATCCCTTTAATTTTATTGATTACTCCAATTACTATTGACGCTGAATTTACT
664157_30677280_HHV-7_JI_U43400.1_144861bp_1_1672	GAAGAAGAAATCCCTTTAATTTTATTGATTACTCCAATTACTATTGACGCTGAATTTACTTCACGAGTAATCAAAGATATATGCTGTATCTGGGAAAATAAAATTGCACAACAGACAAAT
664157_30677280_HHV-7_JI_U43400.1_144861bp_1_1673	TCACGAGTAATCAAAGATATATGCTGTATCTGGGAAAATAAAATTGCACAACAGACAAATTTAAAAATATGGGCTCAAAGTGCTGTAAGACAATACATGGCGGCATCTTCAGCAAGGCCG
664157_30677280_HHV-7_JI_U43400.1_144861bp_1_1674	TTAAAAATATGGGCTCAAAGTGCTGTAAGACAATACATGGCGGCATCTTCAGCAAGGCCGAAGACACCTTAGTAGACTATAAAGGAAAATATATTAATCTTGAAAAAGAATTTTCTGCTT
664157_30677280_HHV-7_JI_U43400.1_144861bp_1_1675	AAGACACCTTAGTAGACTATAAAGGAAAATATATTAATCTTGAAAAAGAATTTTCTGCTTTAAGTGATACTGAATCTGAAGAAGAGTTGCAACTAGAGAAGCCACTTCTAAATAAACAAG
664157_30677280_HHV-7_JI_U43400.1_144861bp_1_1676	TAAGTGATACTGAATCTGAAGAAGAGTTGCAACTAGAGAAGCCACTTCTAAATAAACAAGATTCTAGCGTTTCGTTAACCCAGAAGAAACTTGAAAATCAATCCAAATAAACGTCCATTG
664157_30677280_HHV-7_JI_U43400.1_144861bp_1_1677	ATTCTAGCGTTTCGTTAACCCAGAAGAAACTTGAAAATCAATCCAAATAAACGTCCATTGATTAAAAACCCTTTATTCATTTGCTTCTTCTAATACATCTAGGTCTTCCACTGTTGTAGG
664157_30677280_HHV-7_JI_U43400.1_144861bp_1_1678	ATTAAAAACCCTTTATTCATTTGCTTCTTCTAATACATCTAGGTCTTCCACTGTTGTAGGTAATTTCTTATAATTATGATGTTTCCGCATAAAAAATCTAATTAATCTGCATATAATAAA
664157_30677280_HHV-7_JI_U43400.1_144861bp_1_1679	TAATTTCTTATAATTATGATGTTTCCGCATAAAAAATCTAATTAATCTGCATATAATAAAGGAAAGACAAACTATGGTGATCACAGCTAGATTGACTATGAATTTGACAGTCCATTCCGT
664157_30677280_HHV-7_JI_U43400.1_144861bp_1_1680	GGAAAGACAAACTATGGTGATCACAGCTAGATTGACTATGAATTTGACAGTCCATTCCGTTTTAAATAGTGAATCATATTTTAGTATTGGGTAGGTTAAGCCTAGGATACCAAACAAAAT
664157_30677280_HHV-7_JI_U43400.1_144861bp_1_1681	TTTAAATAGTGAATCATATTTTAGTATTGGGTAGGTTAAGCCTAGGATACCAAACAAAATTCCAAAATGTAAACCAAATTGAACCTTAACATACTGATGTAAAATTAATTCAATCACCAA

664157_30677280_HHV-7_JI_U43400.1_144861bp_1_1682	TCCAAAATGTAAACCAAATTGAACCTTAACATACTGATGTAAAATTAATTCAATCACCAAAGAGTATACAAAAGACATAACAAAAAAGGTGTTGATAGATGCGAAAGCCACTGTTGATGA
664157_30677280_HHV-7_JI_U43400.1_144861bp_1_1683	AGAGTATACAAAAGACATAACAAAAAAGGTGTTGATAGATGCGAAAGCCACTGTTGATGATTTAATATAGAAATTGTTACCTAAACCTAGACAAATCGTAATTGCAAACACCATACTGGA
664157_30677280_HHV-7_JI_U43400.1_144861bp_1_1684	TTTAATATAGAAATTGTTACCTAAACCTAGACAAATCGTAATTGCAAACACCATACTGGAAAAACCCAACATTAGTTCTATCATATTGATTATGATAGTTTTATATTTTATTGTGCCTTT
664157_30677280_HHV-7_JI_U43400.1_144861bp_1_1685	AAAACCCAACATTAGTTCTATCATATTGATTATGATAGTTTTATATTTTATTGTGCCTTTGAGTTTTGGGTGGATCCGTTTCAATACGAAGAAAGATCTTTCAGAGGATTGATACTGTGT
664157_30677280_HHV-7_JI_U43400.1_144861bp_1_1686	GAGTTTTGGGTGGATCCGTTTCAATACGAAGAAAGATCTTTCAGAGGATTGATACTGTGTTATCATAGTCAACGTGAAAGCTGTGAAGCAAATGAAAAACAGGCAATACGTAAAAGCTGC
664157_30677280_HHV-7_JI_U43400.1_144861bp_1_1687	TATCATAGTCAACGTGAAAGCTGTGAAGCAAATGAAAAACAGGCAATACGTAAAAGCTGCGAGGGTGACTAGTCGAAAAGAAAGACAGGTGACAAAGAACTGATATGTATCCATAGTTAA
664157_30677280_HHV-7_JI_U43400.1_144861bp_1_1688	GAGGGTGACTAGTCGAAAAGAAAGACAGGTGACAAAGAACTGATATGTATCCATAGTTAAAACAAATTGGAAACACGATAAACTATCCCCCATCCAAGTTATATCCCTTGTAGATTGATT
664157_30677280_HHV-7_JI_U43400.1_144861bp_1_1689	AACAAATTGGAAACACGATAAACTATCCCCCATCCAAGTTATATCCCTTGTAGATTGATTGATGTTGGGAGTTTTTTCGGAAGAGAATACTTTTACGCAACAAACTATATAGTATACTAA
664157_30677280_HHV-7_JI_U43400.1_144861bp_1_1690	GATGTTGGGAGTTTTTTCGGAAGAGAATACTTTTACGCAACAAACTATATAGTATACTAAGATGAAGAAAAAGCAGATATCCATGAAAACAACATAGCAGATAAGTTGGATAGGATTTAG
664157_30677280_HHV-7_JI_U43400.1_144861bp_1_1691	GATGAAGAAAAAGCAGATATCCATGAAAACAACATAGCAGATAAGTTGGATAGGATTTAGGTAGAGTTGTGGCGTTAGGCTGCGGATGTCATTCCGTATAGAGAGGTTGATGGCTTTCAT
664157_30677280_HHV-7_JI_U43400.1_144861bp_1_1692	GTAGAGTTGTGGCGTTAGGCTGCGGATGTCATTCCGTATAGAGAGGTTGATGGCTTTCATATCATTGATTTCATAATATGCGCATGGGAATCCCAAATTGGGAAAGTGAACAGCCACTAA
664157_30677280_HHV-7_JI_U43400.1_144861bp_1_1693	ATCATTGATTTCATAATATGCGCATGGGAATCCCAAATTGGGAAAGTGAACAGCCACTAAATGCACTGTGACGTTGACATACGTTAAACAAGCACAGATTATACTTAAGACCCAAATTCT

664157_30677280_HHV-7_JI_U43400.1_144861bp_1_1694	ATGCACTGTGACGTTGACATACGTTAAACAAGCACAGATTATACTTAAGACCCAAATTCTCATATTAATCACATCTACTCTGCTCAATGCCATTGTGGGGATCTGTCAAATGGAAACTCA
664157_30677280_HHV-7_JI_U43400.1_144861bp_1_1695	CATATTAATCACATCTACTCTGCTCAATGCCATTGTGGGGATCTGTCAAATGGAAACTCAGCTTCAAAATGATCAATTATTTTTGGAATGGTTTGGACAAAATTTGTTGGATTGCCACTT
664157_30677280_HHV-7_JI_U43400.1_144861bp_1_1696	GCTTCAAAATGATCAATTATTTTTGGAATGGTTTGGACAAAATTTGTTGGATTGCCACTTTGCACAAAACGTTTCAGTGTATTTGCAAGATGCTTCAATGGTTCATTTTAAAACCTTTTC
664157_30677280_HHV-7_JI_U43400.1_144861bp_1_1697	TGCACAAAACGTTTCAGTGTATTTGCAAGATGCTTCAATGGTTCATTTTAAAACCTTTTCTGAACAAATAAAAATTATAAGAGCTCCAATGGGTTCTGGCAAAACCTCTGCATTGATAGA
664157_30677280_HHV-7_JI_U43400.1_144861bp_1_1698	TGAACAAATAAAAATTATAAGAGCTCCAATGGGTTCTGGCAAAACCTCTGCATTGATAGAATTCTTGAAAACTGTTTCATATATTGATTCTGTCCTTGTTATTTCCTGTCGTAAAACTTT
664157_30677280_HHV-7_JI_U43400.1_144861bp_1_1699	ATTCTTGAAAACTGTTTCATATATTGATTCTGTCCTTGTTATTTCCTGTCGTAAAACTTTTGCTGCAGAACTTTTAAATAGATTCAAGAAGAATGATTTGAACGATTTCTATCTGTACAG
664157_30677280_HHV-7_JI_U43400.1_144861bp_1_1700	TGCTGCAGAACTTTTAAATAGATTCAAGAAGAATGATTTGAACGATTTCTATCTGTACAGCGAAATTAAAGAGCGTCAAATCAACAAGAACAAACTGATAATTCAAGTAGAAAGTTTACA
664157_30677280_HHV-7_JI_U43400.1_144861bp_1_1701	CGAAATTAAAGAGCGTCAAATCAACAAGAACAAACTGATAATTCAAGTAGAAAGTTTACATCGTGTTACCAGGAATTATCATGTTTTAATACTAGATGAAATAATGTCTATTATAAAACA
664157_30677280_HHV-7_JI_U43400.1_144861bp_1_1702	TCGTGTTACCAGGAATTATCATGTTTTAATACTAGATGAAATAATGTCTATTATAAAACAGTTCTATTCAAAAACCATGACTAAAGTCAAGGAAGTAGATGCTAAGCTTTTAACGCTTAT
664157_30677280_HHV-7_JI_U43400.1_144861bp_1_1703	GTTCTATTCAAAAACCATGACTAAAGTCAAGGAAGTAGATGCTAAGCTTTTAACGCTTATTAGAAATTCAACACAGATCGTAGCTATGGATGCAACAGTCAATCGTTATGTGGTGGATTT
664157_30677280_HHV-7_JI_U43400.1_144861bp_1_1704	TAGAAATTCAACACAGATCGTAGCTATGGATGCAACAGTCAATCGTTATGTGGTGGATTTTTTCTCTCTCTGTATGCCGCATTTTAAGTCTGCATTGATCATAAATACGTTTGTAAGCGC
664157_30677280_HHV-7_JI_U43400.1_144861bp_1_1705	TTTCTCTCTCTGTATGCCGCATTTTAAGTCTGCATTGATCATAAATACGTTTGTAAGCGCGAATTTTTCTAATAGATCTGCTTATTTTTGTCCAACTTTTATAGATGGGAATCTTGCATT

664157_30677280_HHV-7_JI_U43400.1_144861bp_1_1706	GAATTTTTCTAATAGATCTGCTTATTTTTGTCCAACTTTTATAGATGGGAATCTTGCATTTTATGGAATTCTAAAACAAAAATTAGGCTTAGGAAAAAACATTTGCTTATTTTGTAGCAC
664157_30677280_HHV-7_JI_U43400.1_144861bp_1_1707	TTATGGAATTCTAAAACAAAAATTAGGCTTAGGAAAAAACATTTGCTTATTTTGTAGCACAGTTACATCTGCCGATTTCATGTCAGAATTGTTAAAAACTGATTTCCCGGATAAAAAAAT
664157_30677280_HHV-7_JI_U43400.1_144861bp_1_1708	AGTTACATCTGCCGATTTCATGTCAGAATTGTTAAAAACTGATTTCCCGGATAAAAAAATTTTGCTTTTAACTTCTAAGCAGGGCAAATGTCATTCTGTCGAGAGTTGGATAAACTATAA
664157_30677280_HHV-7_JI_U43400.1_144861bp_1_1709	TTTGCTTTTAACTTCTAAGCAGGGCAAATGTCATTCTGTCGAGAGTTGGATAAACTATAATATTGTTATTTATACATCTATTGTAACGGTCGGGCTCAGTTTTGACTTTCTACATTTTTC
664157_30677280_HHV-7_JI_U43400.1_144861bp_1_1710	TATTGTTATTTATACATCTATTGTAACGGTCGGGCTCAGTTTTGACTTTCTACATTTTTCAGCTATGTTTGTTTACATTCATTTGGTAAAAGGTGGCCCTGATATGGTTTCGGTTTTTCA
664157_30677280_HHV-7_JI_U43400.1_144861bp_1_1711	AGCTATGTTTGTTTACATTCATTTGGTAAAAGGTGGCCCTGATATGGTTTCGGTTTTTCAATCAATGGGTAGAGTTAGAAAGGTCACAGACAACGAAATTTTTATTTATTTGAATCCTGC
664157_30677280_HHV-7_JI_U43400.1_144861bp_1_1712	ATCAATGGGTAGAGTTAGAAAGGTCACAGACAACGAAATTTTTATTTATTTGAATCCTGCATTAATTCAGGTGCCTCTTTCTGTATCCCCTATTTCGATTCCACAATGCTATGATTGGAC
664157_30677280_HHV-7_JI_U43400.1_144861bp_1_1713	ATTAATTCAGGTGCCTCTTTCTGTATCCCCTATTTCGATTCCACAATGCTATGATTGGACACTTTTTGAAAAGTCAATCCTACAATGCAGCTGTATGGATTTTAATAAAAAATGTCTCAG
664157_30677280_HHV-7_JI_U43400.1_144861bp_1_1714	ACTTTTTGAAAAGTCAATCCTACAATGCAGCTGTATGGATTTTAATAAAAAATGTCTCAGTGCACAAAACTACTTGTCTAATTCCATGATAAAACAATTTTTTAGAATTAGGCATTATAT
664157_30677280_HHV-7_JI_U43400.1_144861bp_1_1715	TGCACAAAACTACTTGTCTAATTCCATGATAAAACAATTTTTTAGAATTAGGCATTATATTGAAAAAACTACGCTATTAAATCTTCCTGATAGTCTCTATCTTTTATGTCTTTTATTGGA
664157_30677280_HHV-7_JI_U43400.1_144861bp_1_1716	TGAAAAAACTACGCTATTAAATCTTCCTGATAGTCTCTATCTTTTATGTCTTTTATTGGATAGTAATTCAATTAAAGTTCACATCGATGGAGACGTCTTTCCTATAGCTAAAGAAAAATT
664157_30677280_HHV-7_JI_U43400.1_144861bp_1_1717	TAGTAATTCAATTAAAGTTCACATCGATGGAGACGTCTTTCCTATAGCTAAAGAAAAATTCTACGCATTTACAAAAATGTTGGTTCAAGGTTGCCATTTTTTTGAAAAGAAGAAAACAGA

664157_30677280_HHV-7_JI_U43400.1_144861bp_1_1718	CTACGCATTTACAAAAATGTTGGTTCAAGGTTGCCATTTTTTTGAAAAGAAGAAAACAGACTTTGTAGAAAATACTATGACACTGAAAGAATTGTTTTCTAATACAAATATTACTGTTAA
664157_30677280_HHV-7_JI_U43400.1_144861bp_1_1719	CTTTGTAGAAAATACTATGACACTGAAAGAATTGTTTTCTAATACAAATATTACTGTTAACGGAGAATTCTATGAACTTGGAAATTTCCAAGTTCATAAAGATTACATTGTTAATTTAAA
664157_30677280_HHV-7_JI_U43400.1_144861bp_1_1720	CGGAGAATTCTATGAACTTGGAAATTTCCAAGTTCATAAAGATTACATTGTTAATTTAAATAATTTCCAGAATTTATTTTTGAAAAATGATGTTGACATTTTTGTAATTGAAGAGATTAT
664157_30677280_HHV-7_JI_U43400.1_144861bp_1_1721	TAATTTCCAGAATTTATTTTTGAAAAATGATGTTGACATTTTTGTAATTGAAGAGATTATGCTTACTTTAAAATCTGAAATTAGGAGGTTTGTTTTTATAAATGCTTTGCTACAAAAATA
664157_30677280_HHV-7_JI_U43400.1_144861bp_1_1722	GCTTACTTTAAAATCTGAAATTAGGAGGTTTGTTTTTATAAATGCTTTGCTACAAAAATATGTTGCCACCGGTATCGATGTGGAAAAGATTAAAGCGTTTTTTAAATCCCGAATCAAAAC
664157_30677280_HHV-7_JI_U43400.1_144861bp_1_1723	TGTTGCCACCGGTATCGATGTGGAAAAGATTAAAGCGTTTTTTAAATCCCGAATCAAAACATTTACTCTACCTGAAAACTATATATGTAGCAAGTTTTATTTATTAAGTGATATTTCAGG
664157_30677280_HHV-7_JI_U43400.1_144861bp_1_1724	ATTTACTCTACCTGAAAACTATATATGTAGCAAGTTTTATTTATTAAGTGATATTTCAGGCGTACATGAATGTGGTATGTTGATGGATGTAGCTTTTTTAGCAGAATCTATTAGAGCGGA
664157_30677280_HHV-7_JI_U43400.1_144861bp_1_1725	CGTACATGAATGTGGTATGTTGATGGATGTAGCTTTTTTAGCAGAATCTATTAGAGCGGATTTAAATTTACAGTCCTGTACAGATACGCAAACTGACATCTCAGAAGATGCTATCTTACT
664157_30677280_HHV-7_JI_U43400.1_144861bp_1_1726	TTTAAATTTACAGTCCTGTACAGATACGCAAACTGACATCTCAGAAGATGCTATCTTACTTTGCGCTGCAAGAAGATCATCTGAAATCCTTAGGATTTTACAAATTGTTTTTACAACGCA
664157_30677280_HHV-7_JI_U43400.1_144861bp_1_1727	TTGCGCTGCAAGAAGATCATCTGAAATCCTTAGGATTTTACAAATTGTTTTTACAACGCACGTGCAATTATTTGAAAAATACAACAGTTATACGTTATATTTGTTCAATAGGTTAAAGGG
664157_30677280_HHV-7_JI_U43400.1_144861bp_1_1728	CGTGCAATTATTTGAAAAATACAACAGTTATACGTTATATTTGTTCAATAGGTTAAAGGGAATGCAATTAAATACCTGGTCACTTTCGATTGCAAAATTCAGTGTTTCAATAATAAGGAT
664157_30677280_HHV-7_JI_U43400.1_144861bp_1_1729	AATGCAATTAAATACCTGGTCACTTTCGATTGCAAAATTCAGTGTTTCAATAATAAGGATGTTTTTCAAATGTGCCTTTAATATGAATCTGGTTAAGAGTAAACCCAGATATATCGTTGG

664157_30677280_HHV-7_JI_U43400.1_144861bp_1_1730	GTTTTTCAAATGTGCCTTTAATATGAATCTGGTTAAGAGTAAACCCAGATATATCGTTGGGAAGCCATTTAGAAGTCTAACGAAAAGGGAAATAGAAACTTTACTTGACATGTGGCACGT
664157_30677280_HHV-7_JI_U43400.1_144861bp_1_1731	GAAGCCATTTAGAAGTCTAACGAAAAGGGAAATAGAAACTTTACTTGACATGTGGCACGTATCAAGAACCAATCTGAAAACCTACAAAGAGTTACGTAAGGCTCTGACAGAAGCTTCAAA
664157_30677280_HHV-7_JI_U43400.1_144861bp_1_1732	ATCAAGAACCAATCTGAAAACCTACAAAGAGTTACGTAAGGCTCTGACAGAAGCTTCAAAAAAAAGACAAAGAAAAAAAATTTATAAATTACTAGGTCACAATATAAGTTCTTATATCAG
664157_30677280_HHV-7_JI_U43400.1_144861bp_1_1733	AAAAAGACAAAGAAAAAAAATTTATAAATTACTAGGTCACAATATAAGTTCTTATATCAGTGAAACTGGTTGTCTCTTCCAGCATGCAGATGCGGGGATGTGTTTGTCATCTGGGTGTCT
664157_30677280_HHV-7_JI_U43400.1_144861bp_1_1734	TGAAACTGGTTGTCTCTTCCAGCATGCAGATGCGGGGATGTGTTTGTCATCTGGGTGTCTATTGCGTTCATAACGATTGGAAAAATAAACAATATAGAGTACCAATTTATCAATGCCTGT
664157_30677280_HHV-7_JI_U43400.1_144861bp_1_1735	ATTGCGTTCATAACGATTGGAAAAATAAACAATATAGAGTACCAATTTATCAATGCCTGTTTTTTAATGCTGAGACTCACTCCTTGCATACTTTTTTGGTTATTGGAAATGAGATTTCTG
664157_30677280_HHV-7_JI_U43400.1_144861bp_1_1736	TTTTTAATGCTGAGACTCACTCCTTGCATACTTTTTTGGTTATTGGAAATGAGATTTCTGAAAATCTCCTTGATAAGATTTCCATTTCAAAAGAAAAAATCTTTATGTGGGATTTGAATG
664157_30677280_HHV-7_JI_U43400.1_144861bp_1_1737	AAAATCTCCTTGATAAGATTTCCATTTCAAAAGAAAAAATCTTTATGTGGGATTTGAATGAACAAATAATGAGTATAACTCAAAAGACAGAGTCAATCTGTGAACTGATGTTTGGCAACA
664157_30677280_HHV-7_JI_U43400.1_144861bp_1_1738	AACAAATAATGAGTATAACTCAAAAGACAGAGTCAATCTGTGAACTGATGTTTGGCAACAAACAAATAATTCAAAAACTATCCAGAACATTTATTTTCTTAACAATTATTTTAAATTCTA
664157_30677280_HHV-7_JI_U43400.1_144861bp_1_1739	AACAAATAATTCAAAAACTATCCAGAACATTTATTTTCTTAACAATTATTTTAAATTCTAATATGTATGAAATAATAGATGTCTGCATAGACAGTAATGCGGTTTTGTACATGCCAGACT
664157_30677280_HHV-7_JI_U43400.1_144861bp_1_1740	ATATGTATGAAATAATAGATGTCTGCATAGACAGTAATGCGGTTTTGTACATGCCAGACTTAAGACCAATGATAATAAGATGTGTTGGAAACTATTCGAAAATATCTTCTGAACTATTGT
664157_30677280_HHV-7_JI_U43400.1_144861bp_1_1741	TAAGACCAATGATAATAAGATGTGTTGGAAACTATTCGAAAATATCTTCTGAACTATTGTCAGATTCAGACTGCACACAGGCGACAAAGAAAATGCGATTTGATTATCACGTCACAAACT

664157_30677280_HHV-7_JI_U43400.1_144861bp_1_1742	CAGATTCAGACTGCACACAGGCGACAAAGAAAATGCGATTTGATTATCACGTCACAAACTTTTGTTTCTCATTAAATGTCAATGGTAAAAAAGTACTGTTTTCTAGTACAGAGTCTTCAG
664157_30677280_HHV-7_JI_U43400.1_144861bp_1_1743	TTTGTTTCTCATTAAATGTCAATGGTAAAAAAGTACTGTTTTCTAGTACAGAGTCTTCAGAAAGTATTTTAGAAAAATTGCTTGAGTTTTTCACAATACAAACATATAAATTGGCAGAAG
664157_30677280_HHV-7_JI_U43400.1_144861bp_1_1744	AAAGTATTTTAGAAAAATTGCTTGAGTTTTTCACAATACAAACATATAAATTGGCAGAAGAACAATTCTTAATGGTGACACCTAAAAACTTTTTTACCGTCTTATTTGATGAGGACATGT
664157_30677280_HHV-7_JI_U43400.1_144861bp_1_1745	AACAATTCTTAATGGTGACACCTAAAAACTTTTTTACCGTCTTATTTGATGAGGACATGTGTCTACTTTTGCTGCAAACAGTTGTTAGTTTTTTGTATGACAATCTATTTCGAAATAAGT
664157_30677280_HHV-7_JI_U43400.1_144861bp_1_1746	GTCTACTTTTGCTGCAAACAGTTGTTAGTTTTTTGTATGACAATCTATTTCGAAATAAGTTAGTTGTGAAACAAGTTCATGATTACATTGGTCCAGATTTGTGGCCGCAAGGACATGAGA
664157_30677280_HHV-7_JI_U43400.1_144861bp_1_1747	TAGTTGTGAAACAAGTTCATGATTACATTGGTCCAGATTTGTGGCCGCAAGGACATGAGAGGGCAGTTTATTTTGTAGGGTTTCCTAATATGTGGTTTTTATCGATTTACGATTTGGACA
664157_30677280_HHV-7_JI_U43400.1_144861bp_1_1748	GGGCAGTTTATTTTGTAGGGTTTCCTAATATGTGGTTTTTATCGATTTACGATTTGGACAACAAGATACCTTGTATTAAAAATATTTGCAATAGAATTTTACTTTACTGTGGCCTGCCAG
664157_30677280_HHV-7_JI_U43400.1_144861bp_1_1749	ACAAGATACCTTGTATTAAAAATATTTGCAATAGAATTTTACTTTACTGTGGCCTGCCAGATAGTCTAGGACCAGATGGTTGCCAGAGTGTTCCAAGTACGCAGTGTGTGGATGAATTCG
664157_30677280_HHV-7_JI_U43400.1_144861bp_1_1750	ATAGTCTAGGACCAGATGGTTGCCAGAGTGTTCCAAGTACGCAGTGTGTGGATGAATTCGAAGACCTTCCCAATTTGGGAGCATTACAATATTTGAAATATAATAGTTTGGTTGTTACAA
664157_30677280_HHV-7_JI_U43400.1_144861bp_1_1751	AAGACCTTCCCAATTTGGGAGCATTACAATATTTGAAATATAATAGTTTGGTTGTTACAATGGAAACAGTAGAAAATTCTGAGAATGTGTATTATTTTTTTGGAGAGCAGGATCTGTTTA
664157_30677280_HHV-7_JI_U43400.1_144861bp_1_1752	TGGAAACAGTAGAAAATTCTGAGAATGTGTATTATTTTTTTGGAGAGCAGGATCTGTTTATTGTCAAATTGGTAGACATTATTCAATCTTTGTTGGAACTATATGTTCCTAAAAGTTTTC
664157_30677280_HHV-7_JI_U43400.1_144861bp_1_1753	TTGTCAAATTGGTAGACATTATTCAATCTTTGTTGGAACTATATGTTCCTAAAAGTTTTCTCCCAGATTTTAGCGATGCTTATATAAAAAGTGAAATCTTATTGAAATTTATATCTCGAC

664157_30677280_HHV-7_JI_U43400.1_144861bp_1_1754	TCCCAGATTTTAGCGATGCTTATATAAAAAGTGAAATCTTATTGAAATTTATATCTCGACTTCACAAAAAATCTAACAACATTTTTTGTAAGATCGTAAAAAAAATCACAGAGTTTTTGA
664157_30677280_HHV-7_JI_U43400.1_144861bp_1_1755	TTCACAAAAAATCTAACAACATTTTTTGTAAGATCGTAAAAAAAATCACAGAGTTTTTGAGATGTTTTTTAAACGCGTGTCGATTAATGGATCTTAATTGGATGTTTATTAGAAACATGC
664157_30677280_HHV-7_JI_U43400.1_144861bp_1_1756	GATGTTTTTTAAACGCGTGTCGATTAATGGATCTTAATTGGATGTTTATTAGAAACATGCATATTGTGTATTTTATAGGTCCAAAAAAGGATCCTTCTGTAGTACTTCCTCTGTTAAAAA
664157_30677280_HHV-7_JI_U43400.1_144861bp_1_1757	ATATTGTGTATTTTATAGGTCCAAAAAAGGATCCTTCTGTAGTACTTCCTCTGTTAAAAACGTCTGTGGAAAGTTGTTGGAAAAAAATTCTCAATTCTTCAAGAACTCCAGTAGTGAACA
664157_30677280_HHV-7_JI_U43400.1_144861bp_1_1758	CGTCTGTGGAAAGTTGTTGGAAAAAAATTCTCAATTCTTCAAGAACTCCAGTAGTGAACATAAATTACATCCCTGGTTACAATTTCCTACACAGCTCATGTAGCTTTTTGACAAGCGGAG
664157_30677280_HHV-7_JI_U43400.1_144861bp_1_1759	TAAATTACATCCCTGGTTACAATTTCCTACACAGCTCATGTAGCTTTTTGACAAGCGGAGACATCAATAGTGAAGATTCTCATTGGACTATCACAGCTAGCAAGTGTTTATATAATTGTT
664157_30677280_HHV-7_JI_U43400.1_144861bp_1_1760	ACATCAATAGTGAAGATTCTCATTGGACTATCACAGCTAGCAAGTGTTTATATAATTGTTTAGGGGCAAGTCAGATTACAGTAGATTTTAAAAATATTTACACAAATTTCCAGCAGATGG
664157_30677280_HHV-7_JI_U43400.1_144861bp_1_1761	TAGGGGCAAGTCAGATTACAGTAGATTTTAAAAATATTTACACAAATTTCCAGCAGATGGTTAGCGTTTTTCTAAGTCACAGATACGAAAATAAATATTGGATCGAATATTTTGAGCCCA
664157_30677280_HHV-7_JI_U43400.1_144861bp_1_1762	TTAGCGTTTTTCTAAGTCACAGATACGAAAATAAATATTGGATCGAATATTTTGAGCCCAACAACTATTTTCTAGAGACACACGAAGGTTTACTAGACTGTAATAGATATACAGCTGTCT
664157_30677280_HHV-7_JI_U43400.1_144861bp_1_1763	ACAACTATTTTCTAGAGACACACGAAGGTTTACTAGACTGTAATAGATATACAGCTGTCTGGACAACAGAAAACAAACTAATAAGACAATCTGTTGGTTATCCATTAACGGATAAAATAG
664157_30677280_HHV-7_JI_U43400.1_144861bp_1_1764	GGACAACAGAAAACAAACTAATAAGACAATCTGTTGGTTATCCATTAACGGATAAAATAGATTTTATTCATTACATCCAGGTTGTGATAGAAATTTTCAAAAAGTGGCTCTTAACAAAAT
664157_30677280_HHV-7_JI_U43400.1_144861bp_1_1765	ATTTTATTCATTACATCCAGGTTGTGATAGAAATTTTCAAAAAGTGGCTCTTAACAAAATATAGTCAACAAGAGTATGCAGAAACTGTTAGATTAGGAAGTAAGATCATCTCTGATCATT

664157_30677280_HHV-7_JI_U43400.1_144861bp_1_1766	ATAGTCAACAAGAGTATGCAGAAACTGTTAGATTAGGAAGTAAGATCATCTCTGATCATTTACATTTGTTTAATGTAAACTAGTTTTTTCTATTTCTGTCATCTTATATTCCTTTGAAAA
664157_30677280_HHV-7_JI_U43400.1_144861bp_1_1767	TACATTTGTTTAATGTAAACTAGTTTTTTCTATTTCTGTCATCTTATATTCCTTTGAAAAGGCATCGCATATAGCGCTTTTTAAAAAATCTATTAAATATTTAGAGTCGTCTTGCAAACG
664157_30677280_HHV-7_JI_U43400.1_144861bp_1_1768	GGCATCGCATATAGCGCTTTTTAAAAAATCTATTAAATATTTAGAGTCGTCTTGCAAACGTCTCGTGTTTAGTGATAGAGTACATGTGTCGTCATTGTCGTGAGATTTGAAAAAAAGATT
664157_30677280_HHV-7_JI_U43400.1_144861bp_1_1769	TCTCGTGTTTAGTGATAGAGTACATGTGTCGTCATTGTCGTGAGATTTGAAAAAAAGATTTAACATGTGTATATTTGCTGTTTTTGACTCATAATAGAGCTGAATAATGGGTGGAACTTT
664157_30677280_HHV-7_JI_U43400.1_144861bp_1_1770	TAACATGTGTATATTTGCTGTTTTTGACTCATAATAGAGCTGAATAATGGGTGGAACTTTATCCAGTACGTCTTGCTGTTTGTCATCGTAAAAAATGTATCTTAAAATACTAATCAATTT
664157_30677280_HHV-7_JI_U43400.1_144861bp_1_1771	ATCCAGTACGTCTTGCTGTTTGTCATCGTAAAAAATGTATCTTAAAATACTAATCAATTTTTGACAGGTGCAGGCTAGAAGTTTAGAGACTGGCAGAGTTACACTGATTTTTTTAATAAT
664157_30677280_HHV-7_JI_U43400.1_144861bp_1_1772	TTGACAGGTGCAGGCTAGAAGTTTAGAGACTGGCAGAGTTACACTGATTTTTTTAATAATATCTCGTAATTTAAGTAGTGTCTGCCCTTTTATTGGTAGATTTTCTAGAGCATATACAAT
664157_30677280_HHV-7_JI_U43400.1_144861bp_1_1773	ATCTCGTAATTTAAGTAGTGTCTGCCCTTTTATTGGTAGATTTTCTAGAGCATATACAATACCACAGAGTAAAAACTCCGTGTCTTCCTTTAAAATCAAATAAATGATTCTTCGTTTACA
664157_30677280_HHV-7_JI_U43400.1_144861bp_1_1774	ACCACAGAGTAAAAACTCCGTGTCTTCCTTTAAAATCAAATAAATGATTCTTCGTTTACATTGAGTTTTACTCAGCCGAATCTCTTCTAGCGTATCTTCGGAATCAAGAAGAATAATGCC
664157_30677280_HHV-7_JI_U43400.1_144861bp_1_1775	TTGAGTTTTACTCAGCCGAATCTCTTCTAGCGTATCTTCGGAATCAAGAAGAATAATGCCAATGAAGCCTAGAAACCCTAATGTTTTCTCACATATGCTTTTATAGGATTTGCCATTTAC
664157_30677280_HHV-7_JI_U43400.1_144861bp_1_1776	AATGAAGCCTAGAAACCCTAATGTTTTCTCACATATGCTTTTATAGGATTTGCCATTTACAATTGTGCCATCTTCCAAATGAAACCATACATCATCATTTCCAACCGAAATATCAATTAA
664157_30677280_HHV-7_JI_U43400.1_144861bp_1_1777	AATTGTGCCATCTTCCAAATGAAACCATACATCATCATTTCCAACCGAAATATCAATTAAAGGTAAATTTTTAATGGAAACGTGTTCGGCGTGTTTGTTTAGTTCACAGATATTCCGGAC

664157_30677280_HHV-7_JI_U43400.1_144861bp_1_1778	AGGTAAATTTTTAATGGAAACGTGTTCGGCGTGTTTGTTTAGTTCACAGATATTCCGGACGCTTGATATATTGAATATGTCTAGAGTTTCGAATCCTTTTAAGCTTGCTGGCAACGTCAT
664157_30677280_HHV-7_JI_U43400.1_144861bp_1_1779	GCTTGATATATTGAATATGTCTAGAGTTTCGAATCCTTTTAAGCTTGCTGGCAACGTCATCTTGCGGATATTGAAAATCTTCTGGGTTTGTGGATCCTAAATCAGGTGTCAAAGTCAAAT
664157_30677280_HHV-7_JI_U43400.1_144861bp_1_1780	CTTGCGGATATTGAAAATCTTCTGGGTTTGTGGATCCTAAATCAGGTGTCAAAGTCAAATCACTTTGTTTCTGAGTATGTATTAAATGCGAAAGCTCGTTAATCTGCCGTATATAGATTA
664157_30677280_HHV-7_JI_U43400.1_144861bp_1_1781	CACTTTGTTTCTGAGTATGTATTAAATGCGAAAGCTCGTTAATCTGCCGTATATAGATTAATCTTAAATAATGCTTAATTTTACAGTTTGAGTACACAGCTAACATAATCTCTTTTAGAG
664157_30677280_HHV-7_JI_U43400.1_144861bp_1_1782	ATCTTAAATAATGCTTAATTTTACAGTTTGAGTACACAGCTAACATAATCTCTTTTAGAGAAAGGAACAGAAACTCTTCTGGGAGCAGTTCAAAATGTCTTAATTTTAGAACATGTTTCA
664157_30677280_HHV-7_JI_U43400.1_144861bp_1_1783	AAAGGAACAGAAACTCTTCTGGGAGCAGTTCAAAATGTCTTAATTTTAGAACATGTTTCATGAAGGGTATAAGAAGCAATTCTAGCGTGTAATTTGAATATGAGATGCTGTCTTCCTGTC
664157_30677280_HHV-7_JI_U43400.1_144861bp_1_1784	TGAAGGGTATAAGAAGCAATTCTAGCGTGTAATTTGAATATGAGATGCTGTCTTCCTGTCCTTGATTGTTGACATTTTTTCGTAGACGGAATGTCCTTGTGTATTCAGTTTCCCATAATT
664157_30677280_HHV-7_JI_U43400.1_144861bp_1_1785	CTTGATTGTTGACATTTTTTCGTAGACGGAATGTCCTTGTGTATTCAGTTTCCCATAATTGATCAATTTTTTTGTCTGCGTATTCAATATCTGGAATATATTGTGAAAAAAAACTGTTTG
664157_30677280_HHV-7_JI_U43400.1_144861bp_1_1786	GATCAATTTTTTTGTCTGCGTATTCAATATCTGGAATATATTGTGAAAAAAAACTGTTTGCCACAGCTCTGGTGTCGTCTATTGATACACTAGTGAATGGGAGACTTTGAACTTTGTTCA
664157_30677280_HHV-7_JI_U43400.1_144861bp_1_1787	CCACAGCTCTGGTGTCGTCTATTGATACACTAGTGAATGGGAGACTTTGAACTTTGTTCAGAGCTTTAGCGAGAGACAAGCTTTCAATATCTGAATCTAAAGTAAAGTCATGTTTAGAAG
664157_30677280_HHV-7_JI_U43400.1_144861bp_1_1788	GAGCTTTAGCGAGAGACAAGCTTTCAATATCTGAATCTAAAGTAAAGTCATGTTTAGAAGTTTCGGATAAATTTATTAATGAATGCATGTCTTTAATCTTATTTTCAAATAGCCGATTTG
664157_30677280_HHV-7_JI_U43400.1_144861bp_1_1789	TTTCGGATAAATTTATTAATGAATGCATGTCTTTAATCTTATTTTCAAATAGCCGATTTGCAGTTTTCAGTGTTTCGATTTCGTCCAGTTGTGATTGAATCTGTTCCTCCATGCATTTTA

664157_30677280_HHV-7_JI_U43400.1_144861bp_1_1790	CAGTTTTCAGTGTTTCGATTTCGTCCAGTTGTGATTGAATCTGTTCCTCCATGCATTTTATGATTTGTTTTTTGAAGATATCTCTAATATCGCGATCATTATTGTCCTGCCCACGACGTT
664157_30677280_HHV-7_JI_U43400.1_144861bp_1_1791	TGATTTGTTTTTTGAAGATATCTCTAATATCGCGATCATTATTGTCCTGCCCACGACGTTGCAAATTTCCTTTGCTGATTAATAATTTATTCTGGTCAATTATAGACGGCGTGATGTCTT
664157_30677280_HHV-7_JI_U43400.1_144861bp_1_1792	GCAAATTTCCTTTGCTGATTAATAATTTATTCTGGTCAATTATAGACGGCGTGATGTCTTGTAAAAAACCCTCAATACTATCTGTTATTCCAATTTTAGATTTACTATCGGACAAGTTTA
664157_30677280_HHV-7_JI_U43400.1_144861bp_1_1793	GTAAAAAACCCTCAATACTATCTGTTATTCCAATTTTAGATTTACTATCGGACAAGTTTAATAGAAACTTTATAAGACTTTTTTTCGCATCGCTGGTTTTTTCCTCTCTTTCTATAAGTT
664157_30677280_HHV-7_JI_U43400.1_144861bp_1_1794	ATAGAAACTTTATAAGACTTTTTTTCGCATCGCTGGTTTTTTCCTCTCTTTCTATAAGTTCTAGAATTTTTTTGTTATCAACACTGTTCTTTGATGTAGTTAAAACTTTAATTGGAAAAG
664157_30677280_HHV-7_JI_U43400.1_144861bp_1_1795	CTAGAATTTTTTTGTTATCAACACTGTTCTTTGATGTAGTTAAAACTTTAATTGGAAAAGTATTCAGCAATTGACACATTTTACGGTGCCTGTGTAGGTTTTCGTGTTTATTCAATTCTG
664157_30677280_HHV-7_JI_U43400.1_144861bp_1_1796	TATTCAGCAATTGACACATTTTACGGTGCCTGTGTAGGTTTTCGTGTTTATTCAATTCTGAGTATAAGTGAGCTAGGGGTGTTGTAAACAGCAATTGATTATTTATGAAATATGGTGCAC
664157_30677280_HHV-7_JI_U43400.1_144861bp_1_1797	AGTATAAGTGAGCTAGGGGTGTTGTAAACAGCAATTGATTATTTATGAAATATGGTGCACTGTAAACAAAGTACTCTAGTTCCTTGTTATTGGAGAGACAGGTTATATTGCCGTCTGACT
664157_30677280_HHV-7_JI_U43400.1_144861bp_1_1798	TGTAAACAAAGTACTCTAGTTCCTTGTTATTGGAGAGACAGGTTATATTGCCGTCTGACTTGTTTCGATAAATGTTTATGTTGGTGAAATCGGCAATAGATGTTGAGGAGATCTGTGTTA
664157_30677280_HHV-7_JI_U43400.1_144861bp_1_1799	TGTTTCGATAAATGTTTATGTTGGTGAAATCGGCAATAGATGTTGAGGAGATCTGTGTTAAAATGTGCAGTAGAAAGTTCTGTTTGTTTCGTATGATTTCTTGAATCAATCTAGAGGAAA
664157_30677280_HHV-7_JI_U43400.1_144861bp_1_1800	AAATGTGCAGTAGAAAGTTCTGTTTGTTTCGTATGATTTCTTGAATCAATCTAGAGGAAATACTTTTTGCTAAGGGTGTCAGCGTAACCTTGTTTTTTGGTAATGTGACAATTGGTGTTA
664157_30677280_HHV-7_JI_U43400.1_144861bp_1_1801	TACTTTTTGCTAAGGGTGTCAGCGTAACCTTGTTTTTTGGTAATGTGACAATTGGTGTTATTCCAATCGTACAAATCCAATCAATGTATTTATTATAAATGTCGTTATCGAGATTTTGAA

664157_30677280_HHV-7_JI_U43400.1_144861bp_1_1802	TTCCAATCGTACAAATCCAATCAATGTATTTATTATAAATGTCGTTATCGAGATTTTGAAGGATGTAGTTTATCGTTTGCAAAATGCCTTCCAGGATTACCTGTTTGATTGCATAATACC
664157_30677280_HHV-7_JI_U43400.1_144861bp_1_1803	GGATGTAGTTTATCGTTTGCAAAATGCCTTCCAGGATTACCTGTTTGATTGCATAATACCAGATATTAAAATATTCTTGAAATGACATGCCTTTGCTGCGGAACGGAAATGCTTTCAGAT
664157_30677280_HHV-7_JI_U43400.1_144861bp_1_1804	AGATATTAAAATATTCTTGAAATGACATGCCTTTGCTGCGGAACGGAAATGCTTTCAGATGACGTTGCCATTCAGCATTGATCTGCTGGATGGATATACTATTCAAGAGAGTGTGTGACA
664157_30677280_HHV-7_JI_U43400.1_144861bp_1_1805	GACGTTGCCATTCAGCATTGATCTGCTGGATGGATATACTATTCAAGAGAGTGTGTGACAGTGTGTAAAACATTTGCTTCTTAAAGACAGCCGAGTCTCTAAGAACACTGTGTATAGATT
664157_30677280_HHV-7_JI_U43400.1_144861bp_1_1806	GTGTGTAAAACATTTGCTTCTTAAAGACAGCCGAGTCTCTAAGAACACTGTGTATAGATTGTCCCTTAAAGAATTCTTTTTTACCGTGTAGCATTTCTATAAATTTTATAGTCTTAATAG
664157_30677280_HHV-7_JI_U43400.1_144861bp_1_1807	GTCCCTTAAAGAATTCTTTTTTACCGTGTAGCATTTCTATAAATTTTATAGTCTTAATAGAAGGAAATAAAATGTCATTGTCTTCATTATTCAATGGTAAATATGACACAAAATTCCTTT
664157_30677280_HHV-7_JI_U43400.1_144861bp_1_1808	AAGGAAATAAAATGTCATTGTCTTCATTATTCAATGGTAAATATGACACAAAATTCCTTTTAAACATGTCATCTGCTGCTAAGGTGGAGTTGATTGTTGAGAAAGTTGCAGCTTTAGCTG
664157_30677280_HHV-7_JI_U43400.1_144861bp_1_1809	TAAACATGTCATCTGCTGCTAAGGTGGAGTTGATTGTTGAGAAAGTTGCAGCTTTAGCTGATGCCTGTTTAGAGACGCCGTTACCAACTGACTGGTTTCGTAACATTCTTGATCCTGAAT
664157_30677280_HHV-7_JI_U43400.1_144861bp_1_1810	ATGCCTGTTTAGAGACGCCGTTACCAACTGACTGGTTTCGTAACATTCTTGATCCTGAATTAGAATTTAATAGCAATTTTGAAGAAATTCATTCTATAGGTGATGAAGAATTTGCTCAAC
664157_30677280_HHV-7_JI_U43400.1_144861bp_1_1811	TAGAATTTAATAGCAATTTTGAAGAAATTCATTCTATAGGTGATGAAGAATTTGCTCAACCGTTGCCATTTTTACCTTTTAGAGTATTATTAATAACCGGTACTGCGGGTGCGGGCAAAA
664157_30677280_HHV-7_JI_U43400.1_144861bp_1_1812	CGTTGCCATTTTTACCTTTTAGAGTATTATTAATAACCGGTACTGCGGGTGCGGGCAAAACAAGCAGCATTCAAACCTTAGCAGCTAATAGTGATTGTCTTATAACTGCTACCACTTCCA
664157_30677280_HHV-7_JI_U43400.1_144861bp_1_1813	CAAGCAGCATTCAAACCTTAGCAGCTAATAGTGATTGTCTTATAACTGCTACCACTTCCATTGCTGCTCAAAATCTTAGTGGTTTATTGAACAGAACCAAATCTGCGCAAGTGAAAACAA

664157_30677280_HHV-7_JI_U43400.1_144861bp_1_1814	TTGCTGCTCAAAATCTTAGTGGTTTATTGAACAGAACCAAATCTGCGCAAGTGAAAACAATTTTTAAAACATTTGGTTTTAATAGTTCACATGTATCTATGAATGAAAGAATTAGTTGTT
664157_30677280_HHV-7_JI_U43400.1_144861bp_1_1815	TTTTTAAAACATTTGGTTTTAATAGTTCACATGTATCTATGAATGAAAGAATTAGTTGTTCAGTAACAACTTTAGATTCGATTGCGGATCAGCAAAAACATGATTTATCTACATATTGGA
664157_30677280_HHV-7_JI_U43400.1_144861bp_1_1816	CAGTAACAACTTTAGATTCGATTGCGGATCAGCAAAAACATGATTTATCTACATATTGGAACGTCATCGCAGATATAGCGGAAAGAGCTTTGAATGCGGCAAATGGGAAAACCAAAGTAA
664157_30677280_HHV-7_JI_U43400.1_144861bp_1_1817	ACGTCATCGCAGATATAGCGGAAAGAGCTTTGAATGCGGCAAATGGGAAAACCAAAGTAATACCTGATCTATGTGAAAGCAGTGTGATTGTGATTGATGAAGCAGGAGTGATCTTAAGGC
664157_30677280_HHV-7_JI_U43400.1_144861bp_1_1818	TACCTGATCTATGTGAAAGCAGTGTGATTGTGATTGATGAAGCAGGAGTGATCTTAAGGCACATTTTGCATACAGTTGTCTTTTTTTATTGGTTTTATAACGGTCTTCATAAAACACAGC
664157_30677280_HHV-7_JI_U43400.1_144861bp_1_1819	ACATTTTGCATACAGTTGTCTTTTTTTATTGGTTTTATAACGGTCTTCATAAAACACAGCTCTATAAAAATAGAGTTATTCCTTGTATTGTATGTGTTGGATCTCCTACACAAAGTGGGG
664157_30677280_HHV-7_JI_U43400.1_144861bp_1_1820	TCTATAAAAATAGAGTTATTCCTTGTATTGTATGTGTTGGATCTCCTACACAAAGTGGGGCTTTAATTTCATCGTTTAATCCGTTAACACAAAATAAAGACGTAAAGAAAGGATTTGATA
664157_30677280_HHV-7_JI_U43400.1_144861bp_1_1821	CTTTAATTTCATCGTTTAATCCGTTAACACAAAATAAAGACGTAAAGAAAGGATTTGATATCTTATCCGCTTTAATATGTGACGACATTCTATCAAATTACTGTAAAATATCAGAAAATT
664157_30677280_HHV-7_JI_U43400.1_144861bp_1_1822	TCTTATCCGCTTTAATATGTGACGACATTCTATCAAATTACTGTAAAATATCAGAAAATTGGGTGATTTTTGTTAATAATAAACGATGCACTGATGTGGAATTCGGCGAATTTTTAAAAC
664157_30677280_HHV-7_JI_U43400.1_144861bp_1_1823	GGGTGATTTTTGTTAATAATAAACGATGCACTGATGTGGAATTCGGCGAATTTTTAAAACATATAGAATTTGGTTTGCCATTGAAACCTGAATTGATTGAGTATGTTGATAGGTTTGTTA
664157_30677280_HHV-7_JI_U43400.1_144861bp_1_1824	ATATAGAATTTGGTTTGCCATTGAAACCTGAATTGATTGAGTATGTTGATAGGTTTGTTAGACCGGCAACTTATATTAGAAATCCTACAAACGAAATTGGAATGACGCGTTTATTTTTAT
664157_30677280_HHV-7_JI_U43400.1_144861bp_1_1825	GACCGGCAACTTATATTAGAAATCCTACAAACGAAATTGGAATGACGCGTTTATTTTTATCACATTACGAAGTTAAGTCATATTTTAAAGTTTTACATGAGCAGGTCGAACTGACAAATA

664157_30677280_HHV-7_JI_U43400.1_144861bp_1_1826	CACATTACGAAGTTAAGTCATATTTTAAAGTTTTACATGAGCAGGTCGAACTGACAAATAAAGATAATCTTTTTACTTTTCCTGTTTATTTCATAATTCAGAATAAAGCATTTGAAGATT
664157_30677280_HHV-7_JI_U43400.1_144861bp_1_1827	AAGATAATCTTTTTACTTTTCCTGTTTATTTCATAATTCAGAATAAAGCATTTGAAGATTACAAAAATGAAATTTCAAATTTTACTTTAGAGATTGAACCATGGTTTAAAACTAATTTAC
664157_30677280_HHV-7_JI_U43400.1_144861bp_1_1828	ACAAAAATGAAATTTCAAATTTTACTTTAGAGATTGAACCATGGTTTAAAACTAATTTACACAGATTAAATACTTATTCCCAATTCGCTGATCAAGATTTGTCTAAGACCATACAAATCG
664157_30677280_HHV-7_JI_U43400.1_144861bp_1_1829	ACAGATTAAATACTTATTCCCAATTCGCTGATCAAGATTTGTCTAAGACCATACAAATCGAAGAAATTGTATTAGATGATGGTTCGGTAGAGGAAACTTTGATAACGTGTCATTTGAAAC
664157_30677280_HHV-7_JI_U43400.1_144861bp_1_1830	AAGAAATTGTATTAGATGATGGTTCGGTAGAGGAAACTTTGATAACGTGTCATTTGAAACATATAAAACATAGTTCTATTGGCGTTACTTCCAGAACAAAATCTTCAACTGTTGGGTTTT
664157_30677280_HHV-7_JI_U43400.1_144861bp_1_1831	ATATAAAACATAGTTCTATTGGCGTTACTTCCAGAACAAAATCTTCAACTGTTGGGTTTTCAGGGACATATGAAAAATTTGTGGAGCTTCTGCAAAGTGATTTATTTATTGAAAAAACAG
664157_30677280_HHV-7_JI_U43400.1_144861bp_1_1832	CAGGGACATATGAAAAATTTGTGGAGCTTCTGCAAAGTGATTTATTTATTGAAAAAACAGCATGCGAATATAGCGTGCACGCCTATTCCTTCTTGACAGGTTTAATGTATGGGGGTATGT
664157_30677280_HHV-7_JI_U43400.1_144861bp_1_1833	CATGCGAATATAGCGTGCACGCCTATTCCTTCTTGACAGGTTTAATGTATGGGGGTATGTATTCTTTCTGTTTATCCGAATTCACGACTTCTGAAGTAATGACAGAAATAAGAAAGATCA
664157_30677280_HHV-7_JI_U43400.1_144861bp_1_1834	ATTCTTTCTGTTTATCCGAATTCACGACTTCTGAAGTAATGACAGAAATAAGAAAGATCAAATTGCCCAATATTGATTTTCTACAAACAATGACAGCTGAAGTTTCTTTGCAAACCTTCG
664157_30677280_HHV-7_JI_U43400.1_144861bp_1_1835	AATTGCCCAATATTGATTTTCTACAAACAATGACAGCTGAAGTTTCTTTGCAAACCTTCGATGAATCAGACGAATACTATGATCTACACATTGCACCTACAGATGAAGAAATGTTAGCTT
664157_30677280_HHV-7_JI_U43400.1_144861bp_1_1836	ATGAATCAGACGAATACTATGATCTACACATTGCACCTACAGATGAAGAAATGTTAGCTTCGGATCCGTGCCCAGATCCTTTTTTTTTAAAGTACAAGCAACTTCCATTAACGAATGTTC
664157_30677280_HHV-7_JI_U43400.1_144861bp_1_1837	CGGATCCGTGCCCAGATCCTTTTTTTTTAAAGTACAAGCAACTTCCATTAACGAATGTTCTAACATTTGAAGAAATCAGTTACCTTTATACAGTATTTAAAGAGATTTTCATTTCTAGAT

664157_30677280_HHV-7_JI_U43400.1_144861bp_1_1838	TAACATTTGAAGAAATCAGTTACCTTTATACAGTATTTAAAGAGATTTTCATTTCTAGATTTGCAATTCTACAAAGGCACAGTAAAGAAATGTTCGGCAAGAGTAATTTAATCACATATA
664157_30677280_HHV-7_JI_U43400.1_144861bp_1_1839	TTGCAATTCTACAAAGGCACAGTAAAGAAATGTTCGGCAAGAGTAATTTAATCACATATAATAGGAATAATGTTTCGAGCAAAAGATGTGGGGAGATATGTTCACATGTTAAAAGCTTCT
664157_30677280_HHV-7_JI_U43400.1_144861bp_1_1840	ATAGGAATAATGTTTCGAGCAAAAGATGTGGGGAGATATGTTCACATGTTAAAAGCTTCTACGGCATGTTAACATATGCTGTGCCTGCTAATAATTATACTTTGGAAGGATATACTTATG
664157_30677280_HHV-7_JI_U43400.1_144861bp_1_1841	ACGGCATGTTAACATATGCTGTGCCTGCTAATAATTATACTTTGGAAGGATATACTTATGATAATGTGATTTTCTTAGGGACGGATAAAATGCTTCCTCCGATAATTTACAAAAGAGGTT
664157_30677280_HHV-7_JI_U43400.1_144861bp_1_1842	ATAATGTGATTTTCTTAGGGACGGATAAAATGCTTCCTCCGATAATTTACAAAAGAGGTTTACCCAAAATTGTTATCAAGGATGAAATGGGCTTTATTTCGATCTTAGACAACAATGTAT
664157_30677280_HHV-7_JI_U43400.1_144861bp_1_1843	TACCCAAAATTGTTATCAAGGATGAAATGGGCTTTATTTCGATCTTAGACAACAATGTATCAAAACTTACTGACACTGTCAATGGTAACAGTTTTCATATCTGTACAACCATAGATTACG
664157_30677280_HHV-7_JI_U43400.1_144861bp_1_1844	CAAAACTTACTGACACTGTCAATGGTAACAGTTTTCATATCTGTACAACCATAGATTACGCCATAGTTTCTAAAGTTGCAATGACTGTGACAAAAAGTCAAGGCTTATCTATACAAAGGG
664157_30677280_HHV-7_JI_U43400.1_144861bp_1_1845	CCATAGTTTCTAAAGTTGCAATGACTGTGACAAAAAGTCAAGGCTTATCTATACAAAGGGTTGCCTTAGATTTTGGAAATGATCCTAAAAATTTAAAGTTGAGTTCTATATATGTTGGTA
664157_30677280_HHV-7_JI_U43400.1_144861bp_1_1846	TTGCCTTAGATTTTGGAAATGATCCTAAAAATTTAAAGTTGAGTTCTATATATGTTGGTATGTCAAGAGTTGTTGATCCAAATAATTTAATTATGAATCTGAACCCTTTACGGTTGAACT
664157_30677280_HHV-7_JI_U43400.1_144861bp_1_1847	TGTCAAGAGTTGTTGATCCAAATAATTTAATTATGAATCTGAACCCTTTACGGTTGAACTATGAAAATGATAATATTATTGCTTCACATATTGTAAAGGCCTTAAAAAATAAAGATACTA
664157_30677280_HHV-7_JI_U43400.1_144861bp_1_1848	ATGAAAATGATAATATTATTGCTTCACATATTGTAAAGGCCTTAAAAAATAAAGATACTATGCTTATTTTTTAAATGCCAGAGTTTGTGTTGATTGACGTATTTTTTTAAACTAAGTTGA
664157_30677280_HHV-7_JI_U43400.1_144861bp_1_1849	TGCTTATTTTTTAAATGCCAGAGTTTGTGTTGATTGACGTATTTTTTTAAACTAAGTTGAAATTAAGTTTTTCATGTATCGATGTGCTTTTTTCTGGTATGTTTTTAATGGTTGGCAATT

664157_30677280_HHV-7_JI_U43400.1_144861bp_1_1850	AATTAAGTTTTTCATGTATCGATGTGCTTTTTTCTGGTATGTTTTTAATGGTTGGCAATTGTAAAAATAAAGACATGAATCAATGATTTGGTATACACTATTTTATTGCGAAAATGATAA
664157_30677280_HHV-7_JI_U43400.1_144861bp_1_1851	GTAAAAATAAAGACATGAATCAATGATTTGGTATACACTATTTTATTGCGAAAATGATAAATACTCTGGGTCCGGAAGTTGAGGCTTTTAGAAATCTTTCATCTTCTGAATCTGCAAAGA
664157_30677280_HHV-7_JI_U43400.1_144861bp_1_1852	ATACTCTGGGTCCGGAAGTTGAGGCTTTTAGAAATCTTTCATCTTCTGAATCTGCAAAGAAAAAAATGCTTAGAATCTTTTGCACATCTAGATAAAGTAGGTAAAAAATAAATATAAACA
664157_30677280_HHV-7_JI_U43400.1_144861bp_1_1853	AAAAAATGCTTAGAATCTTTTGCACATCTAGATAAAGTAGGTAAAAAATAAATATAAACAAAATTTACCGTCTGCGTAGTTATTTGTTCCAGTTGAATGAGGACTGCAGTATTCTTATGC
664157_30677280_HHV-7_JI_U43400.1_144861bp_1_1854	AAATTTACCGTCTGCGTAGTTATTTGTTCCAGTTGAATGAGGACTGCAGTATTCTTATGCCAGGTTTTTCAAAGACGCAGTCTAAAAGTAAAGGGAAAAACTTTTCCGGAGTATACAAAT
664157_30677280_HHV-7_JI_U43400.1_144861bp_1_1855	CAGGTTTTTCAAAGACGCAGTCTAAAAGTAAAGGGAAAAACTTTTCCGGAGTATACAAATGTACTCCGGATTGCGCAGGCGTGTTTATTCTACATTTAAAATTGATGGCAGTTAGCGGTG
664157_30677280_HHV-7_JI_U43400.1_144861bp_1_1856	GTACTCCGGATTGCGCAGGCGTGTTTATTCTACATTTAAAATTGATGGCAGTTAGCGGTGTCAGGTGGTATGTATGGTGTCTATTTTAAGTATGTCAGAAATCAACTAAAGTTCCTTTTC
664157_30677280_HHV-7_JI_U43400.1_144861bp_1_1857	TCAGGTGGTATGTATGGTGTCTATTTTAAGTATGTCAGAAATCAACTAAAGTTCCTTTTCCACACACTACTGAAGGTTGTATGAATATCACAATTCACAATTTTATAACAATCTAGAGTT
664157_30677280_HHV-7_JI_U43400.1_144861bp_1_1858	CACACACTACTGAAGGTTGTATGAATATCACAATTCACAATTTTATAACAATCTAGAGTTGCTTTTTAAGGTATGGAAAAAGCCTATGAAATCATGCAAAATGCTGATAATTGTACATTC
664157_30677280_HHV-7_JI_U43400.1_144861bp_1_1859	GCTTTTTAAGGTATGGAAAAAGCCTATGAAATCATGCAAAATGCTGATAATTGTACATTCAGAAAGCTAAAACTACTTATTCTTAATATCTATGACTTAACCTTAAAAATCTATGACAAA
664157_30677280_HHV-7_JI_U43400.1_144861bp_1_1860	AGAAAGCTAAAACTACTTATTCTTAATATCTATGACTTAACCTTAAAAATCTATGACAAAACCTATTGATTATTCTTAACAGCTATTATTAAAATTAAAAGTGATTAACTATTCTTAAAA
664157_30677280_HHV-7_JI_U43400.1_144861bp_1_1861	ACCTATTGATTATTCTTAACAGCTATTATTAAAATTAAAAGTGATTAACTATTCTTAAAAACTATGATTTTACCCCATTCCCCACCCCCACCCCCACCCTTCTTGATTATATCCCGGACC

664157_30677280_HHV-7_JI_U43400.1_144861bp_1_1862	ACTATGATTTTACCCCATTCCCCACCCCCACCCCCACCCTTCTTGATTATATCCCGGACCCCACTAAATGGATTGTCTTTGTATTATGTTGGTGTGTTGCCCACTACACACTGAAACATA
664157_30677280_HHV-7_JI_U43400.1_144861bp_1_1863	CCACTAAATGGATTGTCTTTGTATTATGTTGGTGTGTTGCCCACTACACACTGAAACATAGTACAAGGGCTAACTCTTAATGGTTACAAGTATGCAGCTCAAAATCGTAATGGTTCTGAT
664157_30677280_HHV-7_JI_U43400.1_144861bp_1_1864	GTACAAGGGCTAACTCTTAATGGTTACAAGTATGCAGCTCAAAATCGTAATGGTTCTGATCTTTCTTGTACATGTTCAAGTTTTCGGCGGTTAAGATGCTTTAGAGATAGTTCAAGATTT
664157_30677280_HHV-7_JI_U43400.1_144861bp_1_1865	CTTTCTTGTACATGTTCAAGTTTTCGGCGGTTAAGATGCTTTAGAGATAGTTCAAGATTTTAGATTCAAAACATCACTTATCTCCACAGATCATTTCCAAGCAATTGTACAGAAAGATAG
664157_30677280_HHV-7_JI_U43400.1_144861bp_1_1866	TAGATTCAAAACATCACTTATCTCCACAGATCATTTCCAAGCAATTGTACAGAAAGATAGAATGTCAATGAGAACAGCCGTTCTCATTGTCTTCACAAAGTGAGGTTCAGATCATGACAT
664157_30677280_HHV-7_JI_U43400.1_144861bp_1_1867	AATGTCAATGAGAACAGCCGTTCTCATTGTCTTCACAAAGTGAGGTTCAGATCATGACATAGTAGCCGTAAATGGCTACTGTGGTTTACATTATAAATGAGAACAGCCGTTCCCATAGTT
664157_30677280_HHV-7_JI_U43400.1_144861bp_1_1868	AGTAGCCGTAAATGGCTACTGTGGTTTACATTATAAATGAGAACAGCCGTTCCCATAGTTTTAAAATGAGGTTTAAAACATACAGTAGCCATTACATGGTTACTGTGGTCCAAAACGCGG
664157_30677280_HHV-7_JI_U43400.1_144861bp_1_1869	TTAAAATGAGGTTTAAAACATACAGTAGCCATTACATGGTTACTGTGGTCCAAAACGCGGTATCCGAATCGCAATCCACAATACACAGTCCCGTTGTTCAAAAAGTTATGCAGTTGGCGG
664157_30677280_HHV-7_JI_U43400.1_144861bp_1_1870	TATCCGAATCGCAATCCACAATACACAGTCCCGTTGTTCAAAAAGTTATGCAGTTGGCGGTCTTAATCAGGTATGAGATGGTATGGCAATACACGTTGTAAAAATTCTGAGATTTGAAAC
664157_30677280_HHV-7_JI_U43400.1_144861bp_1_1871	TCTTAATCAGGTATGAGATGGTATGGCAATACACGTTGTAAAAATTCTGAGATTTGAAACAATGATGTCACTGTTGTAGGATATCCTGTAGTTTTTGATGGTTTTTTGGTTGATGACTTA
664157_30677280_HHV-7_JI_U43400.1_144861bp_1_1872	AATGATGTCACTGTTGTAGGATATCCTGTAGTTTTTGATGGTTTTTTGGTTGATGACTTACACAAGATCAAAAGTTACTGGTAATCCTTAGTGTTAGATCTTACATGATATGACAAGAGA
664157_30677280_HHV-7_JI_U43400.1_144861bp_1_1873	CACAAGATCAAAAGTTACTGGTAATCCTTAGTGTTAGATCTTACATGATATGACAAGAGAAAAAAAGCTATATATGGGAAGGGATAACCCATATCAAACAAGAAAAGTTAAAGAGAAGAA

664157_30677280_HHV-7_JI_U43400.1_144861bp_1_1874	AAAAAAGCTATATATGGGAAGGGATAACCCATATCAAACAAGAAAAGTTAAAGAGAAGAAACTATGAGATGGGAGAACCTATAACAAATACACTTTTAAGATTGGGGTGGGTGAACAGGG
664157_30677280_HHV-7_JI_U43400.1_144861bp_1_1875	ACTATGAGATGGGAGAACCTATAACAAATACACTTTTAAGATTGGGGTGGGTGAACAGGGCATAACTGTAGCAATAAAACCAGTGACCGACAGTTACATTCCTTCATAAAAACTAACGTG
664157_30677280_HHV-7_JI_U43400.1_144861bp_1_1876	CATAACTGTAGCAATAAAACCAGTGACCGACAGTTACATTCCTTCATAAAAACTAACGTGATAGCAAAAAAAATATACATAAATAAAGAAGATGGCTTTTCAAGTGATAATGGCAGTCTA
664157_30677280_HHV-7_JI_U43400.1_144861bp_1_1877	ATAGCAAAAAAAATATACATAAATAAAGAAGATGGCTTTTCAAGTGATAATGGCAGTCTAGATACACTTATATTCGTGAGAGGTGTGTCAGTCCATATACAACTTTTCCTATTACCTCGT
664157_30677280_HHV-7_JI_U43400.1_144861bp_1_1878	GATACACTTATATTCGTGAGAGGTGTGTCAGTCCATATACAACTTTTCCTATTACCTCGTCTAAAAAAGGCAACCTAAAACTGGCTGTTTTACCTGGGATGATTTAAATATAGAATAAAC
664157_30677280_HHV-7_JI_U43400.1_144861bp_1_1879	CTAAAAAAGGCAACCTAAAACTGGCTGTTTTACCTGGGATGATTTAAATATAGAATAAACATTCACCTACCTTGTTTTCTTCTCTGAAGAGTAGTAGTGTTGACTGCATTGTCTCCGTGT
664157_30677280_HHV-7_JI_U43400.1_144861bp_1_1880	ATTCACCTACCTTGTTTTCTTCTCTGAAGAGTAGTAGTGTTGACTGCATTGTCTCCGTGTATCTTGATCCAATAGAGCTTTATCTAATAAATACAAAAAAGGAAAAATCCGTATATTCCA
664157_30677280_HHV-7_JI_U43400.1_144861bp_1_1881	ATCTTGATCCAATAGAGCTTTATCTAATAAATACAAAAAAGGAAAAATCCGTATATTCCATAACTTGTTAAGTACAATTTCCATAAAAATTGAAAAAATATATCGTTTAGATGTTATACA
664157_30677280_HHV-7_JI_U43400.1_144861bp_1_1882	TAACTTGTTAAGTACAATTTCCATAAAAATTGAAAAAATATATCGTTTAGATGTTATACAAAAGTTTATCGTTTTAACTAATCATTGTCTATTTTCAGACATCGATACAGAGTTTGATAT
664157_30677280_HHV-7_JI_U43400.1_144861bp_1_1883	AAAGTTTATCGTTTTAACTAATCATTGTCTATTTTCAGACATCGATACAGAGTTTGATATTTACGAGTTTTTCGAAAGATTAAATTTCAATCTCACCTCTTTCGCTGTCTTAACAGTTCA
664157_30677280_HHV-7_JI_U43400.1_144861bp_1_1884	TTACGAGTTTTTCGAAAGATTAAATTTCAATCTCACCTCTTTCGCTGTCTTAACAGTTCATGTAGATCGCGGGCTTTATAAACAAATGCTTTCCAGTTTTCCACGCCCTGGGGGATATGA
664157_30677280_HHV-7_JI_U43400.1_144861bp_1_1885	TGTAGATCGCGGGCTTTATAAACAAATGCTTTCCAGTTTTCCACGCCCTGGGGGATATGACGCAATTTCCTTCATCCGTGAACTTGTCGTGTTTGCGGTTAAACTCGCGGAAGTAAACCG

664157_30677280_HHV-7_JI_U43400.1_144861bp_1_1886	CGCAATTTCCTTCATCCGTGAACTTGTCGTGTTTGCGGTTAAACTCGCGGAAGTAAACCGTTATTTTTCTCGAACGGTAAAATACAAAAACGGTTTATGCGAAACAATTTCAGGCAAAT
664157_30677280_HHV-7_JI_U43400.1_144861bp_1_1887	TTATTTTTCTCGAACGGTAAAATACAAAAACGGTTTATGCGAAACAATTTCAGGCAAATATCATTGCGACTTTTTAAAAACGATTTAAGTTATGAGGGTTTTTGTTGGCTACATTCCGG
664157_30677280_HHV-7_JI_U43400.1_144861bp_1_1888	ATCATTGCGACTTTTTAAAAACGATTTAAGTTATGAGGGTTTTTGTTGGCTACATTCCGGAAATCAATTTTCATGTCAGAAAGCATAAAAGGGAGATGCTTTTGTGTCAGGAAGCAACTT
664157_30677280_HHV-7_JI_U43400.1_144861bp_1_1889	AAATCAATTTTCATGTCAGAAAGCATAAAAGGGAGATGCTTTTGTGTCAGGAAGCAACTTTATCAGTCAAGAACTGTGAAGTACATTCCTTGAGAAATCACTTAGACTCGTAGTAAGAAG
664157_30677280_HHV-7_JI_U43400.1_144861bp_1_1890	TATCAGTCAAGAACTGTGAAGTACATTCCTTGAGAAATCACTTAGACTCGTAGTAAGAAGTGAGTTTTTCAGTGAGTGGAAGCAGGTAAGTATAAAATTTCCCTATTCTATTTCAACCTG
664157_30677280_HHV-7_JI_U43400.1_144861bp_1_1891	TGAGTTTTTCAGTGAGTGGAAGCAGGTAAGTATAAAATTTCCCTATTCTATTTCAACCTGTACTCGACTCTGAATACGTTATTTCTTTTATTGACAGAATGATTGCGGAGGATAGAGAAT
664157_30677280_HHV-7_JI_U43400.1_144861bp_1_1892	TACTCGACTCTGAATACGTTATTTCTTTTATTGACAGAATGATTGCGGAGGATAGAGAATATGGAACGTTTGAATCTGTAACCCAGGCTTATCAGCAGATCATTAGTCATACTTTACAGC
664157_30677280_HHV-7_JI_U43400.1_144861bp_1_1893	ATGGAACGTTTGAATCTGTAACCCAGGCTTATCAGCAGATCATTAGTCATACTTTACAGCTGAGACGATATGAATTTGAAACTGGGTGCATGATTATGTTTTCTGCTAATTCTGGAAAAT
664157_30677280_HHV-7_JI_U43400.1_144861bp_1_1894	TGAGACGATATGAATTTGAAACTGGGTGCATGATTATGTTTTCTGCTAATTCTGGAAAATGTGAGATGCTCTCTAATGGTTGGATTTCAATGATTTCATGGACTTCAGAAACCGATACGG
664157_30677280_HHV-7_JI_U43400.1_144861bp_1_1895	GTGAGATGCTCTCTAATGGTTGGATTTCAATGATTTCATGGACTTCAGAAACCGATACGGCCGGCTCATTGACATTGGATATTTGTACTGAGGGAGGGCAGTGCAAAACTTACAGTGCCA
664157_30677280_HHV-7_JI_U43400.1_144861bp_1_1896	CCGGCTCATTGACATTGGATATTTGTACTGAGGGAGGGCAGTGCAAAACTTACAGTGCCAGAGGTCATATATTATGTTCTAAAAACATCACTTCAATTTCTCAGAAAAACGAGGGAAAGG
664157_30677280_HHV-7_JI_U43400.1_144861bp_1_1897	GAGGTCATATATTATGTTCTAAAAACATCACTTCAATTTCTCAGAAAAACGAGGGAAAGGAAAAAGTTTTGACGATTTGTCATGACAATGGAAAATTGCATTTAACTTATATCACAGTTC

664157_30677280_HHV-7_JI_U43400.1_144861bp_1_1898	AAAAAGTTTTGACGATTTGTCATGACAATGGAAAATTGCATTTAACTTATATCACAGTTCTAAAAAGCGGCCTTGATTGTGATATCAAAGACCAAAAACTGGAGAAACTGTTTGAAAAAG
664157_30677280_HHV-7_JI_U43400.1_144861bp_1_1899	TAAAAAGCGGCCTTGATTGTGATATCAAAGACCAAAAACTGGAGAAACTGTTTGAAAAAGAACATGCAGAACGAAAAAAGCAAGATGATGATTATAAAAAAAAAGCTCTTAAACAAAAAG
664157_30677280_HHV-7_JI_U43400.1_144861bp_1_1900	AACATGCAGAACGAAAAAAGCAAGATGATGATTATAAAAAAAAAGCTCTTAAACAAAAAGATAAACGCCGTTCTGAGCAAAAAATTTTGGAAGACTGTGATAAAAAAGATGAAAAAAAAA
664157_30677280_HHV-7_JI_U43400.1_144861bp_1_1901	ATAAACGCCGTTCTGAGCAAAAAATTTTGGAAGACTGTGATAAAAAAGATGAAAAAAAAAGAATGGATGACACCGAAAAAAGAAAACTACAAGAGGACCGTCGAAACGAAAAACAAGACC
664157_30677280_HHV-7_JI_U43400.1_144861bp_1_1902	GAATGGATGACACCGAAAAAAGAAAACTACAAGAGGACCGTCGAAACGAAAAACAAGACCTAAAAAAAAGAGTAGATGACACCGAAAAAAGAAAATTAGAAGATGACCGTCGAAACGAAA
664157_30677280_HHV-7_JI_U43400.1_144861bp_1_1903	TAAAAAAAAGAGTAGATGACACCGAAAAAAGAAAATTAGAAGATGACCGTCGAAACGAAAAACAAGACCTCGAAGGTTAGTTGCCTTTTTTCTAAATCACTAGTCCTAAATATGACTTGT
664157_30677280_HHV-7_JI_U43400.1_144861bp_1_1904	AACAAGACCTCGAAGGTTAGTTGCCTTTTTTCTAAATCACTAGTCCTAAATATGACTTGTTGAATTTTTGTAGTGTCTAAGGTTTTTACATAAACATGTTTTCTTAGATGCATCCAAAGA
664157_30677280_HHV-7_JI_U43400.1_144861bp_1_1905	TGAATTTTTGTAGTGTCTAAGGTTTTTACATAAACATGTTTTCTTAGATGCATCCAAAGAAAAAAGGATGAAAGTGCATCACGAAAAACGTCATGCGGAAGAACAAGCAAACGAAGAGGT
664157_30677280_HHV-7_JI_U43400.1_144861bp_1_1906	AAAAAGGATGAAAGTGCATCACGAAAAACGTCATGCGGAAGAACAAGCAAACGAAGAGGTTGCTTCTTCGAGTCAGTTATCAAGTAGAATACCAGAGGGTGCGTTATCGCCCACTATTTC
664157_30677280_HHV-7_JI_U43400.1_144861bp_1_1907	TGCTTCTTCGAGTCAGTTATCAAGTAGAATACCAGAGGGTGCGTTATCGCCCACTATTTCTATTGATCTTCAGGAATATCAAGAATTTGAGGATTTTGACAAGCGCATTTGTGGGCAGGT
664157_30677280_HHV-7_JI_U43400.1_144861bp_1_1908	TATTGATCTTCAGGAATATCAAGAATTTGAGGATTTTGACAAGCGCATTTGTGGGCAGGTGGGTGGAGTTTTGGGTTTATGATGCATTTTTATTGCAGTTTTTTATGTGGTTAAAGATGT
664157_30677280_HHV-7_JI_U43400.1_144861bp_1_1909	GGGTGGAGTTTTGGGTTTATGATGCATTTTTATTGCAGTTTTTTATGTGGTTAAAGATGTTTCTTTCTTTTAGGGGAAGAATCAAGACGCTGTATGCAAGAAAGTCCAAAGTGATGAAAG

664157_30677280_HHV-7_JI_U43400.1_144861bp_1_1910	TTCTTTCTTTTAGGGGAAGAATCAAGACGCTGTATGCAAGAAAGTCCAAAGTGATGAAAGTTTTTGTATAAATAAACCGTTAGAGCAGTTTAGAGAGAAACTAATAAAAATTACTCATGA
664157_30677280_HHV-7_JI_U43400.1_144861bp_1_1911	TTTTTGTATAAATAAACCGTTAGAGCAGTTTAGAGAGAAACTAATAAAAATTACTCATGAAGCTGTACAACAGTCACTGTTACAATCGCGGGGAAAAAATGAGGATAATAAAAAAGATGT
664157_30677280_HHV-7_JI_U43400.1_144861bp_1_1912	AGCTGTACAACAGTCACTGTTACAATCGCGGGGAAAAAATGAGGATAATAAAAAAGATGTTACCCAAAATGTAAAATTCGCTGATGAAAATATGAATTTCGCAGGGGGTTCTAAATGTAC
664157_30677280_HHV-7_JI_U43400.1_144861bp_1_1913	TACCCAAAATGTAAAATTCGCTGATGAAAATATGAATTTCGCAGGGGGTTCTAAATGTACTTCTAAAACTAAGCATATTGAAGATCAACAAATACAGTTTGGGGCACAAAATAGATTTGT
664157_30677280_HHV-7_JI_U43400.1_144861bp_1_1914	TTCTAAAACTAAGCATATTGAAGATCAACAAATACAGTTTGGGGCACAAAATAGATTTGTTCCTATATGTGAAATAAAACCATTTATTGATGTCAACCTTTTAAAATCAAATGTAAGAGG
664157_30677280_HHV-7_JI_U43400.1_144861bp_1_1915	TCCTATATGTGAAATAAAACCATTTATTGATGTCAACCTTTTAAAATCAAATGTAAGAGGTAGAAGGAGTACTCGTGGAAGAAGAAACTCAAACTCCGCTAGATTTTCGGCCTCTACTTT
664157_30677280_HHV-7_JI_U43400.1_144861bp_1_1916	TAGAAGGAGTACTCGTGGAAGAAGAAACTCAAACTCCGCTAGATTTTCGGCCTCTACTTTTGAACAACCAATTGAAACTTCTGAGATGCTGACGGTTTCGACTCGATCCAGAGGACGTTC
664157_30677280_HHV-7_JI_U43400.1_144861bp_1_1917	TGAACAACCAATTGAAACTTCTGAGATGCTGACGGTTTCGACTCGATCCAGAGGACGTTCCAGGGGAAGGCCAAGAGGCAGAGGCAGATCCAGAAACATGTCAATGAGACAAACTCCAAG
664157_30677280_HHV-7_JI_U43400.1_144861bp_1_1918	CAGGGGAAGGCCAAGAGGCAGAGGCAGATCCAGAAACATGTCAATGAGACAAACTCCAAGAGAAGTTGAAGACATGTTACCGATTGTTTTGGACAGTGACAGTGACACGGAGACTTTAAG
664157_30677280_HHV-7_JI_U43400.1_144861bp_1_1919	AGAAGTTGAAGACATGTTACCGATTGTTTTGGACAGTGACAGTGACACGGAGACTTTAAGGCGTAATGAAGATTTATTGGCGTCTTCCATATTACAGACTTTATAAATCGTTTGTTATGT
664157_30677280_HHV-7_JI_U43400.1_144861bp_1_1920	GCGTAATGAAGATTTATTGGCGTCTTCCATATTACAGACTTTATAAATCGTTTGTTATGTTCCAATCAATTGGACATTTGTTGTGGATTTGTAAATATTTATTTGCTTTTAAAAAATGAC
664157_30677280_HHV-7_JI_U43400.1_144861bp_1_1921	TCCAATCAATTGGACATTTGTTGTGGATTTGTAAATATTTATTTGCTTTTAAAAAATGACCACAACCGATAAAAGGTGTTTTTGATCCTTTTGTTCTCGGAGATGGATGTGCACATTCCA

664157_30677280_HHV-7_JI_U43400.1_144861bp_1_1922	CACAACCGATAAAAGGTGTTTTTGATCCTTTTGTTCTCGGAGATGGATGTGCACATTCCAAAATCAAGTGTTTCTTTGAATCTATTAAAGACACGAGTTTGCGAGCCTGTGATCCCCACA
664157_30677280_HHV-7_JI_U43400.1_144861bp_1_1923	AAATCAAGTGTTTCTTTGAATCTATTAAAGACACGAGTTTGCGAGCCTGTGATCCCCACAACATAAAAACTAAGTTTTGCATTTTTTCTGATAGTTGGTTAATAATTCGGTTGCTCAAAA
664157_30677280_HHV-7_JI_U43400.1_144861bp_1_1924	ACATAAAAACTAAGTTTTGCATTTTTTCTGATAGTTGGTTAATAATTCGGTTGCTCAAAACTTGCCATCCAATCGCTTCATGGGACATTGGTACTCCCCTTATAACTGTGAAAACAGTGT
664157_30677280_HHV-7_JI_U43400.1_144861bp_1_1925	CTTGCCATCCAATCGCTTCATGGGACATTGGTACTCCCCTTATAACTGTGAAAACAGTGTTGAGTAACAAAACGCCTTGGGCACACCAAGATTTTAAAGATCCGTGGGCTGGAGCTTTAA
664157_30677280_HHV-7_JI_U43400.1_144861bp_1_1926	TGAGTAACAAAACGCCTTGGGCACACCAAGATTTTAAAGATCCGTGGGCTGGAGCTTTAAAATTCTCTATTGTTCTTTCAAGTTCTGCAAAAATGGTTTTTAAAGAATTCGGAGGGGAAC
664157_30677280_HHV-7_JI_U43400.1_144861bp_1_1927	AATTCTCTATTGTTCTTTCAAGTTCTGCAAAAATGGTTTTTAAAGAATTCGGAGGGGAACATCCTCTCACAGTGCTGAAAGCTAATCCGTGACCTCTACCATCGGGATAAGGATCTTGGC
664157_30677280_HHV-7_JI_U43400.1_144861bp_1_1928	ATCCTCTCACAGTGCTGAAAGCTAATCCGTGACCTCTACCATCGGGATAAGGATCTTGGCCCACTATTATCACCTTAATCTCTTCAGGCGAACATAAATAACTCCAGCTGTGTACATTCT
664157_30677280_HHV-7_JI_U43400.1_144861bp_1_1929	CCACTATTATCACCTTAATCTCTTCAGGCGAACATAAATAACTCCAGCTGTGTACATTCTGAGGATCGGGGTAGATAATTAATCTTTCTCTGTCACGCTGAACCAATTTGTATACATTTT
664157_30677280_HHV-7_JI_U43400.1_144861bp_1_1930	GAGGATCGGGGTAGATAATTAATCTTTCTCTGTCACGCTGAACCAATTTGTATACATTTTGTAATTGTACAATGTCAGAATCTGATAAGTTTAAAAACTTAAGCCACTTCACATTGATTT
664157_30677280_HHV-7_JI_U43400.1_144861bp_1_1931	GTAATTGTACAATGTCAGAATCTGATAAGTTTAAAAACTTAAGCCACTTCACATTGATTTGAAACGTTTCATGTTGTTCTTCAAGAGACATTTTTATTGAGTTGTCATCTGAAATGTTTT
664157_30677280_HHV-7_JI_U43400.1_144861bp_1_1932	GAAACGTTTCATGTTGTTCTTCAAGAGACATTTTTATTGAGTTGTCATCTGAAATGTTTTCCAGCATCCACTGTAGTAATGCCATTATTGAATGGGAGAAGCAGTGTTCATGGGCTTTTT
664157_30677280_HHV-7_JI_U43400.1_144861bp_1_1933	CCAGCATCCACTGTAGTAATGCCATTATTGAATGGGAGAAGCAGTGTTCATGGGCTTTTTATTAATGTTGCCTGAACACGTGACACATAAGTTGAGCGATCTATAAAGCATGCGTCAGTA

664157_30677280_HHV-7_JI_U43400.1_144861bp_1_1934	ATTAATGTTGCCTGAACACGTGACACATAAGTTGAGCGATCTATAAAGCATGCGTCAGTAAGTTCTAATGAGCAAATTCGAAAACTCGCTTCGGAATGGAAGCACTTCATTATATTTTTC
664157_30677280_HHV-7_JI_U43400.1_144861bp_1_1935	AGTTCTAATGAGCAAATTCGAAAACTCGCTTCGGAATGGAAGCACTTCATTATATTTTTCCATTTCTTGAATTAACGGGAAACTAAAGTTGTAATACCTAGAAAAATATCTCAAACAATT
664157_30677280_HHV-7_JI_U43400.1_144861bp_1_1936	CATTTCTTGAATTAACGGGAAACTAAAGTTGTAATACCTAGAAAAATATCTCAAACAATTTAACAAAGCATCAAAAACATTAATTCCATGGTGATCAATTCTCACAATTCTCTGTGTGCG
664157_30677280_HHV-7_JI_U43400.1_144861bp_1_1937	TAACAAAGCATCAAAAACATTAATTCCATGGTGATCAATTCTCACAATTCTCTGTGTGCGAGTCTCTTCATTTTCAAGGTAAATCAAAATTGACATGTTAAAATTTATCATATTTGATGG
664157_30677280_HHV-7_JI_U43400.1_144861bp_1_1938	AGTCTCTTCATTTTCAAGGTAAATCAAAATTGACATGTTAAAATTTATCATATTTGATGGTATATTGAATTCAAATCCGATTATATTTTCTACAAAGATGTTGCCCGTGAATTTGAGTTT
664157_30677280_HHV-7_JI_U43400.1_144861bp_1_1939	TATATTGAATTCAAATCCGATTATATTTTCTACAAAGATGTTGCCCGTGAATTTGAGTTTTTCATAAGAGTATGATTTACACACATTATCTCTACAGGTTAAAAGTGTGTTATCTGAAAG
664157_30677280_HHV-7_JI_U43400.1_144861bp_1_1940	TTCATAAGAGTATGATTTACACACATTATCTCTACAGGTTAAAAGTGTGTTATCTGAAAGACATCTTTCAAAGTTATTTAAAAAGCCAAGCCAGTTGTGCCCTGATTCTGAATCTTTAAT
664157_30677280_HHV-7_JI_U43400.1_144861bp_1_1941	ACATCTTTCAAAGTTATTTAAAAAGCCAAGCCAGTTGTGCCCTGATTCTGAATCTTTAATTAAGGAAAGTAAGGTGTGTAGTTGATTAGGATTATTATACAGTAGGTAAAATGTTTCTAA
664157_30677280_HHV-7_JI_U43400.1_144861bp_1_1942	TAAGGAAAGTAAGGTGTGTAGTTGATTAGGATTATTATACAGTAGGTAAAATGTTTCTAAAAAATCTTCATCAATTAAAACTTGATCTCTCCTTGAGATATAGGGAGTATGATATAATAA
664157_30677280_HHV-7_JI_U43400.1_144861bp_1_1943	AAAATCTTCATCAATTAAAACTTGATCTCTCCTTGAGATATAGGGAGTATGATATAATAAGCGAGAAATTATATCCGATTTTGGTATAGGTGCCTGATCAATAGGTTCCAGAAGCGACAG
664157_30677280_HHV-7_JI_U43400.1_144861bp_1_1944	GCGAGAAATTATATCCGATTTTGGTATAGGTGCCTGATCAATAGGTTCCAGAAGCGACAGAGTTTTTGATTGGGTACAATTTAGAATGTTTTTGATACATTCTTGTTCTAAATTAGAATA
664157_30677280_HHV-7_JI_U43400.1_144861bp_1_1945	AGTTTTTGATTGGGTACAATTTAGAATGTTTTTGATACATTCTTGTTCTAAATTAGAATAATAAGAGTTATTAAATAAAGCATATATTTGGTTTAGAATGGAGATAAGAAATATAAAAAA

664157_30677280_HHV-7_JI_U43400.1_144861bp_1_1946	ATAAGAGTTATTAAATAAAGCATATATTTGGTTTAGAATGGAGATAAGAAATATAAAAAAGATGTTAGTTTTCATGTTCCATAAAGTTGTATACAAAACACCAGGAACTTAAATATGGAA
664157_30677280_HHV-7_JI_U43400.1_144861bp_1_1947	GATGTTAGTTTTCATGTTCCATAAAGTTGTATACAAAACACCAGGAACTTAAATATGGAAGACACATTTAAATGGAATCTAATAATTTACATACCGTTTTTGAAGTGAGCTTTCTAATTA
664157_30677280_HHV-7_JI_U43400.1_144861bp_1_1948	GACACATTTAAATGGAATCTAATAATTTACATACCGTTTTTGAAGTGAGCTTTCTAATTATTTCGTGATTATAGAAATTATCATTCTGTTCTTAAATACTCTGAAACAGAAAATCTGGAA
664157_30677280_HHV-7_JI_U43400.1_144861bp_1_1949	TTTCGTGATTATAGAAATTATCATTCTGTTCTTAAATACTCTGAAACAGAAAATCTGGAATTCTTAACAGTAGGTTTTCCTTACCTGTATTAGTAGAAGTTGTATTTTTAGAAGTCGAAT
664157_30677280_HHV-7_JI_U43400.1_144861bp_1_1950	TTCTTAACAGTAGGTTTTCCTTACCTGTATTAGTAGAAGTTGTATTTTTAGAAGTCGAATCTGAGTCAAGATCTATAGAAAATATAAACTTTATATCATTCCAAAAGTTAAGCAATAAAT
664157_30677280_HHV-7_JI_U43400.1_144861bp_1_1951	CTGAGTCAAGATCTATAGAAAATATAAACTTTATATCATTCCAAAAGTTAAGCAATAAATATATAACGTAATTCGAGTCAATCAGAGTACATAAGTTTTAAAATATACTATGTCTTTAAT
664157_30677280_HHV-7_JI_U43400.1_144861bp_1_1952	ATATAACGTAATTCGAGTCAATCAGAGTACATAAGTTTTAAAATATACTATGTCTTTAATAGTTTACCATAATCGTCCCAGTAGATGTATCTATTGAAAGTATTATAGATGATTAAATGC
664157_30677280_HHV-7_JI_U43400.1_144861bp_1_1953	AGTTTACCATAATCGTCCCAGTAGATGTATCTATTGAAAGTATTATAGATGATTAAATGCTCTTTAAGTAATAAAGGTTGTGTTAACTTTGAGACATTTAGTAAAATGTCAGCAGGGTAA
664157_30677280_HHV-7_JI_U43400.1_144861bp_1_1954	TCTTTAAGTAATAAAGGTTGTGTTAACTTTGAGACATTTAGTAAAATGTCAGCAGGGTAACGAATTGAAAATTGGACACAGAGATGAGCAGCACAAAATAGATCTTTTTCAGTTGGGGCA
664157_30677280_HHV-7_JI_U43400.1_144861bp_1_1955	CGAATTGAAAATTGGACACAGAGATGAGCAGCACAAAATAGATCTTTTTCAGTTGGGGCATTTATGTACAGAGAGTAGACACCACTATTTTTCCTGTGTTTGCTGTTGACATTCACAGGC
664157_30677280_HHV-7_JI_U43400.1_144861bp_1_1956	TTTATGTACAGAGAGTAGACACCACTATTTTTCCTGTGTTTGCTGTTGACATTCACAGGCATCATAGCTTCTTTTCTAGTCAGTTCAGTATGTGGTACTGTTCCTCTGAGAAATCTGTTA
664157_30677280_HHV-7_JI_U43400.1_144861bp_1_1957	ATCATAGCTTCTTTTCTAGTCAGTTCAGTATGTGGTACTGTTCCTCTGAGAAATCTGTTAATTGCTATGGCAGTTTTCTGTTCATGGAAAAAGGTCTCAGAAACGGTTTGTTCGCCTTTG

664157_30677280_HHV-7_JI_U43400.1_144861bp_1_1958	ATTGCTATGGCAGTTTTCTGTTCATGGAAAAAGGTCTCAGAAACGGTTTGTTCGCCTTTGTTAACAATCAGATTAGCTATTTCCATCAATTTTTCGTGAAGTGAGTTTACAAGTAATCCG
664157_30677280_HHV-7_JI_U43400.1_144861bp_1_1959	TTAACAATCAGATTAGCTATTTCCATCAATTTTTCGTGAAGTGAGTTTACAAGTAATCCGTTACGTGAATAAACTTGAATTTGAACTACAGTCGAATTATAAAATCGAAGTAATTTATAA
664157_30677280_HHV-7_JI_U43400.1_144861bp_1_1960	TTACGTGAATAAACTTGAATTTGAACTACAGTCGAATTATAAAATCGAAGTAATTTATAAACTTCCGATAAGAAATTTTTCGGCGAACATAAACCGCATGGATGCATAAAATCACAATTA
664157_30677280_HHV-7_JI_U43400.1_144861bp_1_1961	ACTTCCGATAAGAAATTTTTCGGCGAACATAAACCGCATGGATGCATAAAATCACAATTAAAATCTGTATAATTTTTATTTCAGGAGAGTGGATAGGTTTGGAATCGTTAAAATTGCTG
664157_30677280_HHV-7_JI_U43400.1_144861bp_1_1962	AAATCTGTATAATTTTTATTTCAGGAGAGTGGATAGGTTTGGAATCGTTAAAATTGCTGTGAACAATACCCTTAGATTTTGTTGCAAAGTTTGTCGTTGAGAGATTTTCTTTTACAGCC
664157_30677280_HHV-7_JI_U43400.1_144861bp_1_1963	TGAACAATACCCTTAGATTTTGTTGCAAAGTTTGTCGTTGAGAGATTTTCTTTTACAGCCTTAAATATTTTTCTCAATTTAGAATGTTTATAGATGGCATTCGTATATTTGTCCGCCTTA
664157_30677280_HHV-7_JI_U43400.1_144861bp_1_1964	TTAAATATTTTTCTCAATTTAGAATGTTTATAGATGGCATTCGTATATTTGTCCGCCTTAAGTAACATTTCTGATTTTGAAATGTTTATTTCAGAATTTATTTTTTCAGGAATTGTACAT
664157_30677280_HHV-7_JI_U43400.1_144861bp_1_1965	AGTAACATTTCTGATTTTGAAATGTTTATTTCAGAATTTATTTTTTCAGGAATTGTACATTTTATGGCTTTTTCATTAAACGGTAACGTTTTTGAAAAACAGTTACATGTATATCGAGCA
664157_30677280_HHV-7_JI_U43400.1_144861bp_1_1966	TTTATGGCTTTTTCATTAAACGGTAACGTTTTTGAAAAACAGTTACATGTATATCGAGCATTGGCTTTAGTACACATGCAGCTTTTAGAAGTGAGTTTACCTTTGGCCTTACAAACAGTT
664157_30677280_HHV-7_JI_U43400.1_144861bp_1_1967	TTGGCTTTAGTACACATGCAGCTTTTAGAAGTGAGTTTACCTTTGGCCTTACAAACAGTTTTACGTTTTTTGCCCCTCATATTCAAGTACTGAAGCTTTCAAAATTTTCAATGTTGATGA
664157_30677280_HHV-7_JI_U43400.1_144861bp_1_1968	TTACGTTTTTTGCCCCTCATATTCAAGTACTGAAGCTTTCAAAATTTTCAATGTTGATGAGTTCCTAGTTTACACAGAAGGGTTGAAGCTATTATAACGAAAAGCAGGACACTGGAGATT
664157_30677280_HHV-7_JI_U43400.1_144861bp_1_1969	GTTCCTAGTTTACACAGAAGGGTTGAAGCTATTATAACGAAAAGCAGGACACTGGAGATTATTAAAATTATCCACGCCAAATCGTTGTTAATTAACTCTGACTGTTTAAGAGAATCATCA

664157_30677280_HHV-7_JI_U43400.1_144861bp_1_1970	ATTAAAATTATCCACGCCAAATCGTTGTTAATTAACTCTGACTGTTTAAGAGAATCATCACTTAATATTGTATCATTATAGGGGTTTTCAAATGGTTGAATAATCGAGGTAACATTCTCG
664157_30677280_HHV-7_JI_U43400.1_144861bp_1_1971	CTTAATATTGTATCATTATAGGGGTTTTCAAATGGTTGAATAATCGAGGTAACATTCTCGTCTATCTCTCCGCCGTAAGACAGACAAGTGATCGGTTTGCCAAAGAACGTATTGCTGCCT
664157_30677280_HHV-7_JI_U43400.1_144861bp_1_1972	TCTATCTCTCCGCCGTAAGACAGACAAGTGATCGGTTTGCCAAAGAACGTATTGCTGCCTTGTCTTTTAAAAAGAAAGCTGAGTGTCGTTGTCTGCGAGCCGTTGTAATTTTCACGCACT
664157_30677280_HHV-7_JI_U43400.1_144861bp_1_1973	TGTCTTTTAAAAAGAAAGCTGAGTGTCGTTGTCTGCGAGCCGTTGTAATTTTCACGCACTGTTGGACTGTCCTGTCTATATATCTTATCTAAAAATAAAATGGCTACTAATGGCTTTGGA
664157_30677280_HHV-7_JI_U43400.1_144861bp_1_1974	GTTGGACTGTCCTGTCTATATATCTTATCTAAAAATAAAATGGCTACTAATGGCTTTGGATACGACGTCACAGAACATGTTACATCCAAAAAATTTTGCAGATGGCGATAGTACAAAGCA
664157_30677280_HHV-7_JI_U43400.1_144861bp_1_1975	TACGACGTCACAGAACATGTTACATCCAAAAAATTTTGCAGATGGCGATAGTACAAAGCAATTATTGGCTTCATTGAAAGATGCACACAACTCATAGTAGACAATCGAGTCTTGGCGAAG
664157_30677280_HHV-7_JI_U43400.1_144861bp_1_1976	ATTATTGGCTTCATTGAAAGATGCACACAACTCATAGTAGACAATCGAGTCTTGGCGAAGAATGCACATGTTAAGCAACCAGCATCGTTAATTGTTCGAGATTTAAATTTCAGCAATGCT
664157_30677280_HHV-7_JI_U43400.1_144861bp_1_1977	AATGCACATGTTAAGCAACCAGCATCGTTAATTGTTCGAGATTTAAATTTCAGCAATGCTGTGATATTGTTAGCATTGAAGTAAATAATGCTTTCTTTGTCACGATGTTTAAATTCATGA
664157_30677280_HHV-7_JI_U43400.1_144861bp_1_1978	GTGATATTGTTAGCATTGAAGTAAATAATGCTTTCTTTGTCACGATGTTTAAATTCATGAACTATTTTTTCACTCATGTTTTTCAGGTTTTTGGTATTTATGGTATGATTTAATATCAGT
664157_30677280_HHV-7_JI_U43400.1_144861bp_1_1979	ACTATTTTTTCACTCATGTTTTTCAGGTTTTTGGTATTTATGGTATGATTTAATATCAGTTCGTAATGTTTAAACCATATTACAGAGCTGAGATTTTTAGTTGTATCAACAGTACACTGA
664157_30677280_HHV-7_JI_U43400.1_144861bp_1_1980	TCGTAATGTTTAAACCATATTACAGAGCTGAGATTTTTAGTTGTATCAACAGTACACTGAAATACAATTGTGTTTCCTAGTTCTACTCTATGATGCATTATTGGATATAATGCATATATG
664157_30677280_HHV-7_JI_U43400.1_144861bp_1_1981	AATACAATTGTGTTTCCTAGTTCTACTCTATGATGCATTATTGGATATAATGCATATATGATTACAGAAAATCCAATCATCTTTATGAAAATTGCACCTAAGAAAGGCATTGTGTGAAAT

664157_30677280_HHV-7_JI_U43400.1_144861bp_1_1982	ATTACAGAAAATCCAATCATCTTTATGAAAATTGCACCTAAGAAAGGCATTGTGTGAAATTGTCTGATTTTGGATTTTTCTGCATCTATATTATCTGGACACACCCTAAAAAACCACTAT
664157_30677280_HHV-7_JI_U43400.1_144861bp_1_1983	TGTCTGATTTTGGATTTTTCTGCATCTATATTATCTGGACACACCCTAAAAAACCACTATTTTTTCTCTTTTACTTTGATTTTTGTGGTTTTTTTTTTAAATGTGGACCCACCCATTCAA
664157_30677280_HHV-7_JI_U43400.1_144861bp_1_1984	TTTTTCTCTTTTACTTTGATTTTTGTGGTTTTTTTTTTAAATGTGGACCCACCCATTCAAAAATAAGGCGTTTGGGGTAAAGCAAATGTAATCTTTATTTTTTTTTTTTAAAATTTACAA
664157_30677280_HHV-7_JI_U43400.1_144861bp_1_1985	AAATAAGGCGTTTGGGGTAAAGCAAATGTAATCTTTATTTTTTTTTTTTAAAATTTACAAAGTGCTGTGTATTTTGAAAGAAAATCTCTACTTACAGGATTATAGATGGAAATCGGATTA
664157_30677280_HHV-7_JI_U43400.1_144861bp_1_1986	AGTGCTGTGTATTTTGAAAGAAAATCTCTACTTACAGGATTATAGATGGAAATCGGATTATCCCCATCGGTGATAGAGCTTAATCGCAGAACAATTTCTTTTGGTCTCTTTTTTGCATAA
664157_30677280_HHV-7_JI_U43400.1_144861bp_1_1987	TCCCCATCGGTGATAGAGCTTAATCGCAGAACAATTTCTTTTGGTCTCTTTTTTGCATAATTGTTACAGATTTTAGCTGCCATGAAAAAATCTATTGGTGTTGCAGCGTATATTATAATT
664157_30677280_HHV-7_JI_U43400.1_144861bp_1_1988	TTGTTACAGATTTTAGCTGCCATGAAAAAATCTATTGGTGTTGCAGCGTATATTATAATTGTTTTGAGATCTGAATGTCTTGTATACAGAGTATGTGCACTATTACTAATGTTCTCCCAC
664157_30677280_HHV-7_JI_U43400.1_144861bp_1_1989	GTTTTGAGATCTGAATGTCTTGTATACAGAGTATGTGCACTATTACTAATGTTCTCCCACAATTCATGCGGTCCTTTTTTTTGTAATTCGATGAAATTCTTTATGGAATCTTTGTCATAA
664157_30677280_HHV-7_JI_U43400.1_144861bp_1_1990	AATTCATGCGGTCCTTTTTTTTGTAATTCGATGAAATTCTTTATGGAATCTTTGTCATAATATCGCGGGGGGGCGTGGTTCATTTTAAAGGGTTCAGACATGGTGATATTAGGTCTTACC
664157_30677280_HHV-7_JI_U43400.1_144861bp_1_1991	TATCGCGGGGGGGCGTGGTTCATTTTAAAGGGTTCAGACATGGTGATATTAGGTCTTACCATTAGCTCTTTTTGCATGGCTTTTATGTCTTCGGCAACAGTTTTTGACCTGGTAAAATAT
664157_30677280_HHV-7_JI_U43400.1_144861bp_1_1992	ATTAGCTCTTTTTGCATGGCTTTTATGTCTTCGGCAACAGTTTTTGACCTGGTAAAATATATTATAAGCGCACCGGATTTAGAGCTTCCATTGCAGCACAAAGCATAAACCGATTCTTTC
664157_30677280_HHV-7_JI_U43400.1_144861bp_1_1993	ATTATAAGCGCACCGGATTTAGAGCTTCCATTGCAGCACAAAGCATAAACCGATTCTTTCAATTTGGCTTCCGGAAACGGTTTTTTTGGAATTTGTTTATATTCAAAAGCGCGGATTGTT

664157_30677280_HHV-7_JI_U43400.1_144861bp_1_1994	AATTTGGCTTCCGGAAACGGTTTTTTTGGAATTTGTTTATATTCAAAAGCGCGGATTGTTTTTCGAAACAGTTTTTCAAAATCTTTAGTGGGAACACGACCACGTTGTACTTTAAATTTA
664157_30677280_HHV-7_JI_U43400.1_144861bp_1_1995	TTTCGAAACAGTTTTTCAAAATCTTTAGTGGGAACACGACCACGTTGTACTTTAAATTTAGCTGATTGCTTTTGCTCTTGTAATTTTACATACTCCCTGTAGGATAAATCATTATCAGAT
664157_30677280_HHV-7_JI_U43400.1_144861bp_1_1996	GCTGATTGCTTTTGCTCTTGTAATTTTACATACTCCCTGTAGGATAAATCATTATCAGATAGTTCTGAGTCAGATTTTTGGGTACCAGATATCCGCTTTTTGTAGAAAAGCTGCATGCTT
664157_30677280_HHV-7_JI_U43400.1_144861bp_1_1997	AGTTCTGAGTCAGATTTTTGGGTACCAGATATCCGCTTTTTGTAGAAAAGCTGCATGCTTGAGTCCGAATCATCAGAAAAACGTTTCCTCGCATTTCTGCGTTTATTTTTAAAAAATTTA
664157_30677280_HHV-7_JI_U43400.1_144861bp_1_1998	GAGTCCGAATCATCAGAAAAACGTTTCCTCGCATTTCTGCGTTTATTTTTAAAAAATTTATTAGTACTTTTCTTTTGTTCATACTCATTCGGAATACTTTTGGCCCCATGAGTAAAATCT
664157_30677280_HHV-7_JI_U43400.1_144861bp_1_1999	TTAGTACTTTTCTTTTGTTCATACTCATTCGGAATACTTTTGGCCCCATGAGTAAAATCTATATCTACACGCTTGCTTTTTTGTGTAGATCCAGAAGACCACGAATGATTAGAACAGCTT
664157_30677280_HHV-7_JI_U43400.1_144861bp_1_2000	ATATCTACACGCTTGCTTTTTTGTGTAGATCCAGAAGACCACGAATGATTAGAACAGCTTCTTTCCGATCTTTTTTTGCAATCTGATTCAGAAGACCTGCTTCTACTTCTTAGAGTTTCT
664157_30677280_HHV-7_JI_U43400.1_144861bp_1_2001	CTTTCCGATCTTTTTTTGCAATCTGATTCAGAAGACCTGCTTCTACTTCTTAGAGTTTCTGAATTCTTATTTTTACAGGATTGATAAGTGCTTTCAGAAATACTGTGTTCTGAATTTGAT
664157_30677280_HHV-7_JI_U43400.1_144861bp_1_2002	GAATTCTTATTTTTACAGGATTGATAAGTGCTTTCAGAAATACTGTGTTCTGAATTTGATCTAGATATAGATATCAAATGTTTGCATTGTGTTTCCGACTGAGAATCTTGTTTATTGTTA
664157_30677280_HHV-7_JI_U43400.1_144861bp_1_2003	CTAGATATAGATATCAAATGTTTGCATTGTGTTTCCGACTGAGAATCTTGTTTATTGTTAGAACACACTTTACATGTTCTGGATTTAGATGCAGATCTAGATCTCGATGTATCAGAGCTG
664157_30677280_HHV-7_JI_U43400.1_144861bp_1_2004	GAACACACTTTACATGTTCTGGATTTAGATGCAGATCTAGATCTCGATGTATCAGAGCTGCTTCTAGATCTGTGTTTTTGGCGATGTCTTGTTTTAGTACTTTCTGAACATGATTCTGAC
664157_30677280_HHV-7_JI_U43400.1_144861bp_1_2005	CTTCTAGATCTGTGTTTTTGGCGATGTCTTGTTTTAGTACTTTCTGAACATGATTCTGACTCAGTGTTTCTGTGTTCGTGCCGTCTCTTTTTAGAAGAGGAAAAGGATTGGGATTGTAAA

664157_30677280_HHV-7_JI_U43400.1_144861bp_1_2006	TCAGTGTTTCTGTGTTCGTGCCGTCTCTTTTTAGAAGAGGAAAAGGATTGGGATTGTAAATCTGAATTGGTATCCGATTCTGACTCTGTGCTCGGTGGTCTCTTTTTCCTTATGTGAGGG
664157_30677280_HHV-7_JI_U43400.1_144861bp_1_2007	TCTGAATTGGTATCCGATTCTGACTCTGTGCTCGGTGGTCTCTTTTTCCTTATGTGAGGGCGAAATCTTGGATTATACAAATGTTCATTGTTTTGATCTAATTGAAGTCTAGATTCAACA
664157_30677280_HHV-7_JI_U43400.1_144861bp_1_2008	CGAAATCTTGGATTATACAAATGTTCATTGTTTTGATCTAATTGAAGTCTAGATTCAACATGTTTATACAAATTTAGCTCTTTTCCACACCTGAATTCAAATTCTGACATATCAACTTTG
664157_30677280_HHV-7_JI_U43400.1_144861bp_1_2009	TGTTTATACAAATTTAGCTCTTTTCCACACCTGAATTCAAATTCTGACATATCAACTTTGCTAACAAAATCACACTGTCTATTGCCATTAGAATTAGGAGATTCTGTATATGAAGGTTTT
664157_30677280_HHV-7_JI_U43400.1_144861bp_1_2010	CTAACAAAATCACACTGTCTATTGCCATTAGAATTAGGAGATTCTGTATATGAAGGTTTTAACTCTGTAGAGGAACAACTATTTCTACTTAATACAGAATTTTTAGAAGATGCTGAGCTA
664157_30677280_HHV-7_JI_U43400.1_144861bp_1_2011	AACTCTGTAGAGGAACAACTATTTCTACTTAATACAGAATTTTTAGAAGATGCTGAGCTAGATGACTTACAACTGCATTTACTGCTTGAACTGCTGCTTGAACTCGATGTATCCGTATCA
664157_30677280_HHV-7_JI_U43400.1_144861bp_1_2012	GATGACTTACAACTGCATTTACTGCTTGAACTGCTGCTTGAACTCGATGTATCCGTATCAGAGTCACTGGATGAACTTCGTGTAGATTTTGGACTCATAGATCTATCACTTATAGGAAAC
664157_30677280_HHV-7_JI_U43400.1_144861bp_1_2013	GAGTCACTGGATGAACTTCGTGTAGATTTTGGACTCATAGATCTATCACTTATAGGAAACTTTTTTGTTTCAGAGGAGTTCTGTGTGCATTGTAATTCATTATCAAAGAATTCAAATAGC
664157_30677280_HHV-7_JI_U43400.1_144861bp_1_2014	TTTTTTGTTTCAGAGGAGTTCTGTGTGCATTGTAATTCATTATCAAAGAATTCAAATAGCCCATTCTCTGTGTCTGATTTAGCTAAAAATTCTTGTTCAGGACTTACAAAATTTTGTGCT
664157_30677280_HHV-7_JI_U43400.1_144861bp_1_2015	CCATTCTCTGTGTCTGATTTAGCTAAAAATTCTTGTTCAGGACTTACAAAATTTTGTGCTATTTCATCTGTGGTTTGAGAAATGATCTGTTCCATGTGTTGATTTTCTATGCTGTGTACA
664157_30677280_HHV-7_JI_U43400.1_144861bp_1_2016	ATTTCATCTGTGGTTTGAGAAATGATCTGTTCCATGTGTTGATTTTCTATGCTGTGTACATGTTCAACTTCCGACTGTGAATAATCATTTACTGGTGATTTATATTGTCCTTCCTCAGTA
664157_30677280_HHV-7_JI_U43400.1_144861bp_1_2017	TGTTCAACTTCCGACTGTGAATAATCATTTACTGGTGATTTATATTGTCCTTCCTCAGTACTACTTGAATCAGATCGTGTCCTTTGTGTAAGATTGTCGTTACACATGGAATCTGTTGTA

664157_30677280_HHV-7_JI_U43400.1_144861bp_1_2018	CTACTTGAATCAGATCGTGTCCTTTGTGTAAGATTGTCGTTACACATGGAATCTGTTGTATTTGATTTTTCAGATTCTACAGGGGGATTATCGATAGAAGGTATATTACATGGTGCTGTG
664157_30677280_HHV-7_JI_U43400.1_144861bp_1_2019	TTTGATTTTTCAGATTCTACAGGGGGATTATCGATAGAAGGTATATTACATGGTGCTGTGACTTTCCCCTCAAAATCGTTTTCCTTAGCATCTATTTTGGAATTAACAGTAGTTTTTGCT
664157_30677280_HHV-7_JI_U43400.1_144861bp_1_2020	ACTTTCCCCTCAAAATCGTTTTCCTTAGCATCTATTTTGGAATTAACAGTAGTTTTTGCTTGTCTAGGTCTACCTACACGATATGAATTTTTTCCTGTACATTTTGTATCAGAGAAAACA
664157_30677280_HHV-7_JI_U43400.1_144861bp_1_2021	TGTCTAGGTCTACCTACACGATATGAATTTTTTCCTGTACATTTTGTATCAGAGAAAACAGTACTAGAATTACAATTTAAGGCAGTACTTACTGTACAGCGTGAAGATTGCTTAATGAAT
664157_30677280_HHV-7_JI_U43400.1_144861bp_1_2022	GTACTAGAATTACAATTTAAGGCAGTACTTACTGTACAGCGTGAAGATTGCTTAATGAATTCTATATCAGAAGCAGAAATGTCTTTGTCATTTTTTTCTAGGAGTCTTTTAAAATTAATC
664157_30677280_HHV-7_JI_U43400.1_144861bp_1_2023	TCTATATCAGAAGCAGAAATGTCTTTGTCATTTTTTTCTAGGAGTCTTTTAAAATTAATCAACTTAGTTGGAGCTCGTTGATTTGATTCCTGTACGTCTGGTTTAGGAGTTAATGGCAGT
664157_30677280_HHV-7_JI_U43400.1_144861bp_1_2024	AACTTAGTTGGAGCTCGTTGATTTGATTCCTGTACGTCTGGTTTAGGAGTTAATGGCAGTGGAGAAAGCATTGGTTCTAGTTTTGATAATTTTCTGAGATTTTCTATAGCTTCATCTGGT
664157_30677280_HHV-7_JI_U43400.1_144861bp_1_2025	GGAGAAAGCATTGGTTCTAGTTTTGATAATTTTCTGAGATTTTCTATAGCTTCATCTGGTGATTTGTCCTTTTTCTTTAAGTTTGAAGAACCTTTGTCCAAGTTGGGACAATGCTTAGAA
664157_30677280_HHV-7_JI_U43400.1_144861bp_1_2026	GATTTGTCCTTTTTCTTTAAGTTTGAAGAACCTTTGTCCAAGTTGGGACAATGCTTAGAATCTGTAGTCGATGTGATTTGTTTTTCTTTTGATGTACTAGGTCTTGTTCTAGCTTTCATA
664157_30677280_HHV-7_JI_U43400.1_144861bp_1_2027	TCTGTAGTCGATGTGATTTGTTTTTCTTTTGATGTACTAGGTCTTGTTCTAGCTTTCATAAAAGTCTTTTCCTGTGAAGACTTGTTATATTTACATTTGTCTACATCTGAAGTCGAAGAT
664157_30677280_HHV-7_JI_U43400.1_144861bp_1_2028	AAAGTCTTTTCCTGTGAAGACTTGTTATATTTACATTTGTCTACATCTGAAGTCGAAGATAGACTGTCTTTAGCCAGGGGAACTATATCAGCATTATAAATTCTTTTAATTAGATCCATC
664157_30677280_HHV-7_JI_U43400.1_144861bp_1_2029	AGACTGTCTTTAGCCAGGGGAACTATATCAGCATTATAAATTCTTTTAATTAGATCCATCTTATGAGAACTTGGAATATATGACAAGCTTACAACATCCTCATTTTGTGGTGTTTTGTCT

664157_30677280_HHV-7_JI_U43400.1_144861bp_1_2030	TTATGAGAACTTGGAATATATGACAAGCTTACAACATCCTCATTTTGTGGTGTTTTGTCTTTGTCTGTCTTTTTCCTGGCAGGTTTTTCCAAATTGTTCACGTTTGAGATTAATTTGGTT
664157_30677280_HHV-7_JI_U43400.1_144861bp_1_2031	TTGTCTGTCTTTTTCCTGGCAGGTTTTTCCAAATTGTTCACGTTTGAGATTAATTTGGTTTCCTTGTGATTTGTCTGAATTGGTGTATTTTGACTGTATTCACTTTTCTTTGATAATGTA
664157_30677280_HHV-7_JI_U43400.1_144861bp_1_2032	TCCTTGTGATTTGTCTGAATTGGTGTATTTTGACTGTATTCACTTTTCTTTGATAATGTAAGACCTGATGAAATACATTCTTTTTGAAGGTTTTGACTATCTGTGATCATAGAGCTGCTT
664157_30677280_HHV-7_JI_U43400.1_144861bp_1_2033	AGACCTGATGAAATACATTCTTTTTGAAGGTTTTGACTATCTGTGATCATAGAGCTGCTTGGAATTTGTGAAAAAGCCATGCTGTAATCTCTGCTTTGAAAGCCATAGTTAGGACAAAGT
664157_30677280_HHV-7_JI_U43400.1_144861bp_1_2034	GGAATTTGTGAAAAAGCCATGCTGTAATCTCTGCTTTGAAAGCCATAGTTAGGACAAAGTTGTTGATAATTGTCTCTGTAAGGATTATGTCTAAAATCTGAATTGTATGAGTCATATGAC
664157_30677280_HHV-7_JI_U43400.1_144861bp_1_2035	TGTTGATAATTGTCTCTGTAAGGATTATGTCTAAAATCTGAATTGTATGAGTCATATGACTGATCTCGTTGCATATATTCCGGAAAGGTAAATTCATTTCTGTTGGAAAAGTCATGAAAT
664157_30677280_HHV-7_JI_U43400.1_144861bp_1_2036	TGATCTCGTTGCATATATTCCGGAAAGGTAAATTCATTTCTGTTGGAAAAGTCATGAAATTTAGCATAGCCACGTCCACGTCTGTTGTAAGGTCTGTGAAAATGTTTACCATAATGATTT
664157_30677280_HHV-7_JI_U43400.1_144861bp_1_2037	TTAGCATAGCCACGTCCACGTCTGTTGTAAGGTCTGTGAAAATGTTTACCATAATGATTTTGGTATTTTGAGTTAGGGTTACAATAATTTCTACTGTCATATTTTCCTCTGTTGTTGCGA
664157_30677280_HHV-7_JI_U43400.1_144861bp_1_2038	TGGTATTTTGAGTTAGGGTTACAATAATTTCTACTGTCATATTTTCCTCTGTTGTTGCGACCCCTGTAATGATGTTGTCGATAGGTTGTATCGAACGAATTGTGACAAAGTTCTCTATTT
664157_30677280_HHV-7_JI_U43400.1_144861bp_1_2039	CCCCTGTAATGATGTTGTCGATAGGTTGTATCGAACGAATTGTGACAAAGTTCTCTATTTTTATAGTCCAAATTGTTAAAAATATCAACTGAAGATTGGGGTTGATATGGTAAAGAGATA
664157_30677280_HHV-7_JI_U43400.1_144861bp_1_2040	TTATAGTCCAAATTGTTAAAAATATCAACTGAAGATTGGGGTTGATATGGTAAAGAGATAAAATTTTCTCCACTTGCTTGAGGATAATTATAATACAATGATTGCAGTCCTTGATGTGGC
664157_30677280_HHV-7_JI_U43400.1_144861bp_1_2041	AAATTTTCTCCACTTGCTTGAGGATAATTATAATACAATGATTGCAGTCCTTGATGTGGCAATTCTGATGCAAATCGAAATTGTCCAGTTTCAGTTGTATTTGGAGAAGATTCAGAAGTA

664157_30677280_HHV-7_JI_U43400.1_144861bp_1_2042	AATTCTGATGCAAATCGAAATTGTCCAGTTTCAGTTGTATTTGGAGAAGATTCAGAAGTAAAAGTACTTCTTGATTTAAGAGGAATAGCACGTCGCGGATCTTGAAGAGTTTTTGATGTG
664157_30677280_HHV-7_JI_U43400.1_144861bp_1_2043	AAAGTACTTCTTGATTTAAGAGGAATAGCACGTCGCGGATCTTGAAGAGTTTTTGATGTGACTATTTCTTGGTTGTTAAAATTTGAATAAGTTATTTCATTTTCTGGTTTTGTATGATCG
664157_30677280_HHV-7_JI_U43400.1_144861bp_1_2044	ACTATTTCTTGGTTGTTAAAATTTGAATAAGTTATTTCATTTTCTGGTTTTGTATGATCGTGAATTTCATTATATAAACCCGAAAGAGTCATGGCTTCACGAACCACGTCAGCTGTAGTG
664157_30677280_HHV-7_JI_U43400.1_144861bp_1_2045	TGAATTTCATTATATAAACCCGAAAGAGTCATGGCTTCACGAACCACGTCAGCTGTAGTGCTCATGGGTGCATGCAATGGTCTTTTTTCTGAAAATGAAAAATAAAAATTGTTTATTTTA
664157_30677280_HHV-7_JI_U43400.1_144861bp_1_2046	CTCATGGGTGCATGCAATGGTCTTTTTTCTGAAAATGAAAAATAAAAATTGTTTATTTTAAAATGTTATTAAATTAGCGATAATACTAAGATGATATATACCTTTAAATAGAGTGGAAAT
664157_30677280_HHV-7_JI_U43400.1_144861bp_1_2047	AAATGTTATTAAATTAGCGATAATACTAAGATGATATATACCTTTAAATAGAGTGGAAATGTACTTTTAATTGTCTAGAAAATATAACGCAATATGTTAGTTAAAAATGTAACTTGTTAG
664157_30677280_HHV-7_JI_U43400.1_144861bp_1_2048	GTACTTTTAATTGTCTAGAAAATATAACGCAATATGTTAGTTAAAAATGTAACTTGTTAGATTGATATTTCATGGGCCCTGAAACCTGCATATGAGCTAATGTGTGTACAAATTTGTGAC
664157_30677280_HHV-7_JI_U43400.1_144861bp_1_2049	ATTGATATTTCATGGGCCCTGAAACCTGCATATGAGCTAATGTGTGTACAAATTTGTGACAATAAATATTAAAGTGGAAACATTCCAAATGTAGCAATTTACATGTAAACGGATATGTAA
664157_30677280_HHV-7_JI_U43400.1_144861bp_1_2050	AATAAATATTAAAGTGGAAACATTCCAAATGTAGCAATTTACATGTAAACGGATATGTAAATGTAATAAAGATGTGTATGGTTATGTCAAAAGAGCTAATTTGCAAACATGCAGTTATTA
664157_30677280_HHV-7_JI_U43400.1_144861bp_1_2051	ATGTAATAAAGATGTGTATGGTTATGTCAAAAGAGCTAATTTGCAAACATGCAGTTATTAATTGTAATGGATGTACAAATGTTGATACATGTTTGTGTCTGTTGCTGTGTATATGAGTTT
664157_30677280_HHV-7_JI_U43400.1_144861bp_1_2052	ATTGTAATGGATGTACAAATGTTGATACATGTTTGTGTCTGTTGCTGTGTATATGAGTTTATGTTAAATGTTTATGTAAATATTATATGTTGGTACATATCAATGTATTTATGTTGATAA
664157_30677280_HHV-7_JI_U43400.1_144861bp_1_2053	ATGTTAAATGTTTATGTAAATATTATATGTTGGTACATATCAATGTATTTATGTTGATAAGCAGATGTTAGTATGTATTAGTATCAATGGATTGTAGTATGCTACTGCATGCTATTAAAC

664157_30677280_HHV-7_JI_U43400.1_144861bp_1_2054	GCAGATGTTAGTATGTATTAGTATCAATGGATTGTAGTATGCTACTGCATGCTATTAAACAAGTAGAGTGTTTACTTGTATTAGATTATATTTACATGACATTGTCATGTAAATATATAG
664157_30677280_HHV-7_JI_U43400.1_144861bp_1_2055	AAGTAGAGTGTTTACTTGTATTAGATTATATTTACATGACATTGTCATGTAAATATATAGTTGCATACATTTTTCATGTAACAACACATATGTGTTCATGTACATATTACATAATATATG
664157_30677280_HHV-7_JI_U43400.1_144861bp_1_2056	TTGCATACATTTTTCATGTAACAACACATATGTGTTCATGTACATATTACATAATATATGCTAGTAAATGATTACATGCACTAGTAAATATTTGCACATGCTAATGTGTTCATGTGGGTA
664157_30677280_HHV-7_JI_U43400.1_144861bp_1_2057	CTAGTAAATGATTACATGCACTAGTAAATATTTGCACATGCTAATGTGTTCATGTGGGTATATGTACATATTACATAATATATGCTAGTAAATGATTACATGCACTAGCATATATTTGCA
664157_30677280_HHV-7_JI_U43400.1_144861bp_1_2058	TATGTACATATTACATAATATATGCTAGTAAATGATTACATGCACTAGCATATATTTGCACATACTAATGTGTTCATGTGGGTATATGTACATATTACATAATATATGCTAGTAAATGAT
664157_30677280_HHV-7_JI_U43400.1_144861bp_1_2059	CATACTAATGTGTTCATGTGGGTATATGTACATATTACATAATATATGCTAGTAAATGATTACATGCACTAGCATATATTTGCACATACTAGCATATATTATGTAATATGTACATATTAC
664157_30677280_HHV-7_JI_U43400.1_144861bp_1_2060	TACATGCACTAGCATATATTTGCACATACTAGCATATATTATGTAATATGTACATATTACATAATATATGCTAGTAAATGATTACATGCACTAGTAAATATTTGCACATGCTAATACATA
664157_30677280_HHV-7_JI_U43400.1_144861bp_1_2061	ATAATATATGCTAGTAAATGATTACATGCACTAGTAAATATTTGCACATGCTAATACATAACAGTACATGTTAATAACTTTTGATCTATGCCGCTACATGTTAGTACATGCTATACTGAT
664157_30677280_HHV-7_JI_U43400.1_144861bp_1_2062	ACAGTACATGTTAATAACTTTTGATCTATGCCGCTACATGTTAGTACATGCTATACTGATAATGCGTGGAAGTTTATTATTTGGATTTCTGTAAGGTCATGTGTATTCTATGAATGAGTA
664157_30677280_HHV-7_JI_U43400.1_144861bp_1_2063	AATGCGTGGAAGTTTATTATTTGGATTTCTGTAAGGTCATGTGTATTCTATGAATGAGTATATAAATGGGTATGTTAATATATGTTAGTATGGTTGCAAGTAAAGATATGTTAATGTGTA
664157_30677280_HHV-7_JI_U43400.1_144861bp_1_2064	TATAAATGGGTATGTTAATATATGTTAGTATGGTTGCAAGTAAAGATATGTTAATGTGTATTAGTAAGTACGTAATGTATGTGAATGTATTCAATTTTCAAAGAAATGATATAGAAATTT
664157_30677280_HHV-7_JI_U43400.1_144861bp_1_2065	TTAGTAAGTACGTAATGTATGTGAATGTATTCAATTTTCAAAGAAATGATATAGAAATTTCTAAACATGTTATTGTGGAAAATGTAGATCTTGTGATCTATTATAATAATATAAACATAA

664157_30677280_HHV-7_JI_U43400.1_144861bp_1_2066	CTAAACATGTTATTGTGGAAAATGTAGATCTTGTGATCTATTATAATAATATAAACATAAATTAAATTTTTAAGTTATAGCTACCAATCTTTGCCTATTTTTCTTGGGGGGCATACTTTT
664157_30677280_HHV-7_JI_U43400.1_144861bp_1_2067	ATTAAATTTTTAAGTTATAGCTACCAATCTTTGCCTATTTTTCTTGGGGGGCATACTTTTTATATGATTTTATATGTACGGAAATATATGTACGTAGAATAAAGAATAAAATACTTGAAA
664157_30677280_HHV-7_JI_U43400.1_144861bp_1_2068	TATATGATTTTATATGTACGGAAATATATGTACGTAGAATAAAGAATAAAATACTTGAAATCAAACCCTGTTATAAGTAAATTTACTTAGTATCATATATGTATAAATTTTGATAATTTA
664157_30677280_HHV-7_JI_U43400.1_144861bp_1_2069	TCAAACCCTGTTATAAGTAAATTTACTTAGTATCATATATGTATAAATTTTGATAATTTATGAAGATGGCATAAATGTTGAAAATATTCATGTGTTTGTTATAAGAATTTCAATGTTACT
664157_30677280_HHV-7_JI_U43400.1_144861bp_1_2070	TGAAGATGGCATAAATGTTGAAAATATTCATGTGTTTGTTATAAGAATTTCAATGTTACTAAAATAAAACCAATCACTAATTATCTTTTATATGCAAAAATGTGTCTTTGTATATATTCA
664157_30677280_HHV-7_JI_U43400.1_144861bp_1_2071	AAAATAAAACCAATCACTAATTATCTTTTATATGCAAAAATGTGTCTTTGTATATATTCAAATTAAAATTTAAAACTTATGTTTTAATCATGTGAAATACAGATGTATGAATATGTAGCA
664157_30677280_HHV-7_JI_U43400.1_144861bp_1_2072	AATTAAAATTTAAAACTTATGTTTTAATCATGTGAAATACAGATGTATGAATATGTAGCATGGAAACTTCAAAAATATAAATCATATAGACCTATTGCTTTAACTATATTTAAGATTTAC
664157_30677280_HHV-7_JI_U43400.1_144861bp_1_2073	TGGAAACTTCAAAAATATAAATCATATAGACCTATTGCTTTAACTATATTTAAGATTTACATATTTAAATATTACATAATAAAAATTTTAATATTTAAATAAAAATATGTATTATAAATA
664157_30677280_HHV-7_JI_U43400.1_144861bp_1_2074	ATATTTAAATATTACATAATAAAAATTTTAATATTTAAATAAAAATATGTATTATAAATATGTTATTTACTAGTACAGCTTTATAAATTAAAGAACATATGTATTTAAAGTTTAAAAACT
664157_30677280_HHV-7_JI_U43400.1_144861bp_1_2075	TGTTATTTACTAGTACAGCTTTATAAATTAAAGAACATATGTATTTAAAGTTTAAAAACTAATGAAATGTGCTTCTATGAGTTAATTGTAAATGTCAATTTTGTGATTTTAATATTTTTC
664157_30677280_HHV-7_JI_U43400.1_144861bp_1_2076	AATGAAATGTGCTTCTATGAGTTAATTGTAAATGTCAATTTTGTGATTTTAATATTTTTCTAAATAATGAGATTCAATGTTGTTAATTAGATGTGTGCAGTTTGAGAACATGTTTATATT
664157_30677280_HHV-7_JI_U43400.1_144861bp_1_2077	TAAATAATGAGATTCAATGTTGTTAATTAGATGTGTGCAGTTTGAGAACATGTTTATATTTACAAAAATTTGTATATATTTAAAAATATGTTAATCAGCATAGCCTTATTTATAATGATT

664157_30677280_HHV-7_JI_U43400.1_144861bp_1_2078	TACAAAAATTTGTATATATTTAAAAATATGTTAATCAGCATAGCCTTATTTATAATGATTAATTTTAAATTCTATGTAATTAAATCTTATGGACAAAATAAATTTCAAACCATTTATTTT
664157_30677280_HHV-7_JI_U43400.1_144861bp_1_2079	AATTTTAAATTCTATGTAATTAAATCTTATGGACAAAATAAATTTCAAACCATTTATTTTGAAAAACATAAATGCAGTTTAGAACTGATACAATTTTTTTTTTCAAAAGGGGTACATACT
664157_30677280_HHV-7_JI_U43400.1_144861bp_1_2080	GAAAAACATAAATGCAGTTTAGAACTGATACAATTTTTTTTTTCAAAAGGGGTACATACTTAAAAGATTTTTTACAGTGGTTATAAAAGTATTAGAAAAGGAATTAAGATATATATGAAA
664157_30677280_HHV-7_JI_U43400.1_144861bp_1_2081	TAAAAGATTTTTTACAGTGGTTATAAAAGTATTAGAAAAGGAATTAAGATATATATGAAAAAATTTCCATCCTGCTTATGGAACTATAATATATCAAAGCAATTTATTTATGAAAATTCC
664157_30677280_HHV-7_JI_U43400.1_144861bp_1_2082	AAATTTCCATCCTGCTTATGGAACTATAATATATCAAAGCAATTTATTTATGAAAATTCCAAGTCTATGAAAGTAAAGATACCCATTCAAGTGAAAGGCTATTATAGTTAGGAGGGCTAG
664157_30677280_HHV-7_JI_U43400.1_144861bp_1_2083	AAGTCTATGAAAGTAAAGATACCCATTCAAGTGAAAGGCTATTATAGTTAGGAGGGCTAGCAATATAAGAAAACTTATATTCAATGTATTTTTTTTTAAGCTATGAAAGTAAAGATACCT
664157_30677280_HHV-7_JI_U43400.1_144861bp_1_2084	CAATATAAGAAAACTTATATTCAATGTATTTTTTTTTAAGCTATGAAAGTAAAGATACCTATTCAAGTGAAAGGCTATTATAGTTAGGAGGGCTAGCAATATAAGAAAACTTATATTCAA
664157_30677280_HHV-7_JI_U43400.1_144861bp_1_2085	ATTCAAGTGAAAGGCTATTATAGTTAGGAGGGCTAGCAATATAAGAAAACTTATATTCAATGTATTTTTTAAGCTATGAGATAAATTTGATAGGTCTAAAACATTTTATTTATAAGAAAA
664157_30677280_HHV-7_JI_U43400.1_144861bp_1_2086	TGTATTTTTTAAGCTATGAGATAAATTTGATAGGTCTAAAACATTTTATTTATAAGAAAAAAATGAAAAATTTTATTTCATAGATACAATAACACAGATGAAAGTTACATTTATAATGTA
664157_30677280_HHV-7_JI_U43400.1_144861bp_1_2087	AAATGAAAAATTTTATTTCATAGATACAATAACACAGATGAAAGTTACATTTATAATGTATCTATCCAAATTCTTATAATGTGTAAACAGTAAGTTAAACTAATATGTAAAATATTTAAG
664157_30677280_HHV-7_JI_U43400.1_144861bp_1_2088	TCTATCCAAATTCTTATAATGTGTAAACAGTAAGTTAAACTAATATGTAAAATATTTAAGTAAAAAAAGTATAAAAAACTTAAGGTTTATTTAAAAGTCCATATTTTATAAAAGTTAATG
664157_30677280_HHV-7_JI_U43400.1_144861bp_1_2089	TAAAAAAAGTATAAAAAACTTAAGGTTTATTTAAAAGTCCATATTTTATAAAAGTTAATGAAAAAGAAACTGAATAGAAATAGATTGATAAGTCCATATGCAAAAACTACAATTTCAACA

664157_30677280_HHV-7_JI_U43400.1_144861bp_1_2090	AAAAAGAAACTGAATAGAAATAGATTGATAAGTCCATATGCAAAAACTACAATTTCAACAAAAGGAAAAGTGATGTTTAATAGATTTGAGCATTTTCCAAAAATGGATCATATCCCTGTG
664157_30677280_HHV-7_JI_U43400.1_144861bp_1_2091	AAAGGAAAAGTGATGTTTAATAGATTTGAGCATTTTCCAAAAATGGATCATATCCCTGTGGATAACCTTCAAGAAAGTCAACATAAGTTTGATCAGAATCTGTGTTTTCTTTCCCATAAT
664157_30677280_HHV-7_JI_U43400.1_144861bp_1_2092	GATAACCTTCAAGAAAGTCAACATAAGTTTGATCAGAATCTGTGTTTTCTTTCCCATAATTTTGAGTGTCAACACTCTGTTCTAAAATAAATGAGTTCATCTCACTTACATTTTTATTTG
664157_30677280_HHV-7_JI_U43400.1_144861bp_1_2093	TTTGAGTGTCAACACTCTGTTCTAAAATAAATGAGTTCATCTCACTTACATTTTTATTTGCATATCTCTTTTTTAATTCTTCATAATGTCTATTAATACCATTTTTAATCATTTCTGTGA
664157_30677280_HHV-7_JI_U43400.1_144861bp_1_2094	CATATCTCTTTTTTAATTCTTCATAATGTCTATTAATACCATTTTTAATCATTTCTGTGATTTCCATAAAATTATCCATGATAATATTTGTTACCATGGTTTTAGTCTCTGAAATGCTGT
664157_30677280_HHV-7_JI_U43400.1_144861bp_1_2095	TTTCCATAAAATTATCCATGATAATATTTGTTACCATGGTTTTAGTCTCTGAAATGCTGTCAACCTCAATAAAATTTTCTTGGAGTTTTGGAAATATATATGAGACATTTTCCCGCCTAT
664157_30677280_HHV-7_JI_U43400.1_144861bp_1_2096	CAACCTCAATAAAATTTTCTTGGAGTTTTGGAAATATATATGAGACATTTTCCCGCCTATATTCTTCATCATTACTTCTTATTGGAATAAAATGATTATTAAAACAATAACTTGCTTGTT
664157_30677280_HHV-7_JI_U43400.1_144861bp_1_2097	ATTCTTCATCATTACTTCTTATTGGAATAAAATGATTATTAAAACAATAACTTGCTTGTTTGGTCAGTTCTTTATTTTCATCTTTTTTCATGCCTGTGTCTGGAATGTTAACTTTATGAG
664157_30677280_HHV-7_JI_U43400.1_144861bp_1_2098	TGGTCAGTTCTTTATTTTCATCTTTTTTCATGCCTGTGTCTGGAATGTTAACTTTATGAGTCTTACGTAAAAACCACAGGCAGTTTTTTTTAGGAACAATTTTAACTCTGACACCTCTTG
664157_30677280_HHV-7_JI_U43400.1_144861bp_1_2099	TCTTACGTAAAAACCACAGGCAGTTTTTTTTAGGAACAATTTTAACTCTGACACCTCTTGTCACCCCCCGTATGGATCTGATAGGAATTGTGAAAACAACACTAGGGAATCCATTGTTTT
664157_30677280_HHV-7_JI_U43400.1_144861bp_1_2100	TCACCCCCCGTATGGATCTGATAGGAATTGTGAAAACAACACTAGGGAATCCATTGTTTTCACAGTTATCAGAATTGTTAATTAGTTTTGATCTGTAAGGAATAGAATCTGTAGACATCT
664157_30677280_HHV-7_JI_U43400.1_144861bp_1_2101	CACAGTTATCAGAATTGTTAATTAGTTTTGATCTGTAAGGAATAGAATCTGTAGACATCTCTGTATTAAAATTACTCATGTTTTCATTGTCTGTATCAGTGCAAGAATTTAACTCCATAT

664157_30677280_HHV-7_JI_U43400.1_144861bp_1_2102	CTGTATTAAAATTACTCATGTTTTCATTGTCTGTATCAGTGCAAGAATTTAACTCCATATTATTGCAGCCCTCTTTAACAATTAGTTTTTTTTTTTTTGCTTTGCTAGTTTTCAACTTGC
664157_30677280_HHV-7_JI_U43400.1_144861bp_1_2103	TATTGCAGCCCTCTTTAACAATTAGTTTTTTTTTTTTTGCTTTGCTAGTTTTCAACTTGCTTTCACTTTTTCTCTTTAATTGTTTTTTTTTAACAGCACTGAAATCATGTGCTGTAGTCT
664157_30677280_HHV-7_JI_U43400.1_144861bp_1_2104	TTTCACTTTTTCTCTTTAATTGTTTTTTTTTAACAGCACTGAAATCATGTGCTGTAGTCTGTGAGTCACTGAAGGATGTTAGACTGACACTACTGAATGTAGAATTTGTATCAGTGCTGG
664157_30677280_HHV-7_JI_U43400.1_144861bp_1_2105	GTGAGTCACTGAAGGATGTTAGACTGACACTACTGAATGTAGAATTTGTATCAGTGCTGGAGCTCTCACTTTCATATATGGTTGATTTATCTGTATCATAACCATATGTTTCCATCTCAG
664157_30677280_HHV-7_JI_U43400.1_144861bp_1_2106	AGCTCTCACTTTCATATATGGTTGATTTATCTGTATCATAACCATATGTTTCCATCTCAGAAATCACCAGATTTGTATTATCTGTATCTCTACTGTTATTATTGGAATTCTGCTCCAATA
664157_30677280_HHV-7_JI_U43400.1_144861bp_1_2107	AAATCACCAGATTTGTATTATCTGTATCTCTACTGTTATTATTGGAATTCTGCTCCAATAAAATCAATCCTTCAGCGGCCACTTTTTCCACATCACTTTCTGTGTCAACATCAGCTGTTG
664157_30677280_HHV-7_JI_U43400.1_144861bp_1_2108	AAATCAATCCTTCAGCGGCCACTTTTTCCACATCACTTTCTGTGTCAACATCAGCTGTTGATCTTTCTACAGCTCTATTAGTTGAAATATCCTTTTTCTTATCTCTGATGTAAAAGGTTT
664157_30677280_HHV-7_JI_U43400.1_144861bp_1_2109	ATCTTTCTACAGCTCTATTAGTTGAAATATCCTTTTTCTTATCTCTGATGTAAAAGGTTTCATTAGGAAATTTATCTGGAATAATTAAATCTTTTCCATCTTTATATTTGTTTTCATTTC
664157_30677280_HHV-7_JI_U43400.1_144861bp_1_2110	CATTAGGAAATTTATCTGGAATAATTAAATCTTTTCCATCTTTATATTTGTTTTCATTTCTGTTAAGGTTATGAAAATTTACATTGGAGGTTTTATTGCCCTGACTAACACTCTGTTTAT
664157_30677280_HHV-7_JI_U43400.1_144861bp_1_2111	TGTTAAGGTTATGAAAATTTACATTGGAGGTTTTATTGCCCTGACTAACACTCTGTTTATTTTTAATCATTTCTTCTTGCCCTGATAAAGACTTATCTGTTTTAATTTTAACATCTGTTT
664157_30677280_HHV-7_JI_U43400.1_144861bp_1_2112	TTTTAATCATTTCTTCTTGCCCTGATAAAGACTTATCTGTTTTAATTTTAACATCTGTTTTGTCTTCAGACAGATTGTGACCTTTGTTCTGATTATCTAATGCATGATTTGTCATGGTAA
664157_30677280_HHV-7_JI_U43400.1_144861bp_1_2113	TGTCTTCAGACAGATTGTGACCTTTGTTCTGATTATCTAATGCATGATTTGTCATGGTAAAAAGTTCAAAGTACTGTTCTTTATCTTGGGTGTCCTTATTAAATATTGACCTTTCCATGA

664157_30677280_HHV-7_JI_U43400.1_144861bp_1_2114	AAAGTTCAAAGTACTGTTCTTTATCTTGGGTGTCCTTATTAAATATTGACCTTTCCATGAGTATTCCAGCTGGGCTATTTGTTTTTTTATCTGTTCTGGAAAAGGTAGAAGGAAATTCAT
664157_30677280_HHV-7_JI_U43400.1_144861bp_1_2115	GTATTCCAGCTGGGCTATTTGTTTTTTTATCTGTTCTGGAAAAGGTAGAAGGAAATTCATTTGAAAATGTCAAAGAGTTATTGACGCAAATTTCCTCAGTTACTGACCTTTCCATGAGTA
664157_30677280_HHV-7_JI_U43400.1_144861bp_1_2116	TTGAAAATGTCAAAGAGTTATTGACGCAAATTTCCTCAGTTACTGACCTTTCCATGAGTATTCCAGCTTGGTTATCTGTTTTTTTATCTCTCCTGGAAAAGGTTGTATGAGGAAATTCAT
664157_30677280_HHV-7_JI_U43400.1_144861bp_1_2117	TTCCAGCTTGGTTATCTGTTTTTTTATCTCTCCTGGAAAAGGTTGTATGAGGAAATTCATTTGAAAAGTTCAAAGAGTTCTTATCTTGATAAGTTTCCTCATTTCTTTTAAGGTTATACA
664157_30677280_HHV-7_JI_U43400.1_144861bp_1_2118	TTGAAAAGTTCAAAGAGTTCTTATCTTGATAAGTTTCCTCATTTCTTTTAAGGTTATACAAACTCATGTTAAAGGTTTCATTGTTCTTACTGACTTTCTGTTTGTTTTTAATAGTCTTTC
664157_30677280_HHV-7_JI_U43400.1_144861bp_1_2119	AACTCATGTTAAAGGTTTCATTGTTCTTACTGACTTTCTGTTTGTTTTTAATAGTCTTTCTCTGTCCTGTTAGAATTTGATCTGCTTTAGTATCCCTGCAGCTTTTCATAGTAGCCTCTT
664157_30677280_HHV-7_JI_U43400.1_144861bp_1_2120	TCTGTCCTGTTAGAATTTGATCTGCTTTAGTATCCCTGCAGCTTTTCATAGTAGCCTCTTTGAAAAAATCCTGTGTGGCTGCTGCTTCGGAAAGCCCCGTCTCATTTTTTATAGCATTCA
664157_30677280_HHV-7_JI_U43400.1_144861bp_1_2121	TGAAAAAATCCTGTGTGGCTGCTGCTTCGGAAAGCCCCGTCTCATTTTTTATAGCATTCACTTCAGAATTTTTCTCAACTGAGATTATAGGATTATCAGATAAGAATCTGCAAATAGTAT
664157_30677280_HHV-7_JI_U43400.1_144861bp_1_2122	CTTCAGAATTTTTCTCAACTGAGATTATAGGATTATCAGATAAGAATCTGCAAATAGTATTCCTGGAAAAATCCTGTGTGTCTGTTCCTTCAGAAACATTTACCTCACTTTCTTCAGTAC
664157_30677280_HHV-7_JI_U43400.1_144861bp_1_2123	TCCTGGAAAAATCCTGTGTGTCTGTTCCTTCAGAAACATTTACCTCACTTTCTTCAGTACTCATTCCAGGATTTGTTTCAGCTGGATACATGAGACAGTCAGGTGAGTCTTTTGAAACAA
664157_30677280_HHV-7_JI_U43400.1_144861bp_1_2124	TCATTCCAGGATTTGTTTCAGCTGGATACATGAGACAGTCAGGTGAGTCTTTTGAAACAACCTTTTTGGCAAGATTCTGTGTATCTATCTCTTCAAAGAAATCTAATTTATATTCTCTGG
664157_30677280_HHV-7_JI_U43400.1_144861bp_1_2125	CCTTTTTGGCAAGATTCTGTGTATCTATCTCTTCAAAGAAATCTAATTTATATTCTCTGGTACAAACTTCAGGATTTTTTCCAGTTGTGTTTTTAGAGTGTGATTCATCTGATGCTTGTG

664157_30677280_HHV-7_JI_U43400.1_144861bp_1_2126	TACAAACTTCAGGATTTTTTCCAGTTGTGTTTTTAGAGTGTGATTCATCTGATGCTTGTGAAACAGCCAGAGTTGTTTTAATGGTTTGGCATATGTCTGAAAAATTTGATTGAACATTGT
664157_30677280_HHV-7_JI_U43400.1_144861bp_1_2127	AAACAGCCAGAGTTGTTTTAATGGTTTGGCATATGTCTGAAAAATTTGATTGAACATTGTACTGTTCAGACTTTGAAACACACTGTGTAGGACTGACATTCTGATGTACACTTTGGTAGG
664157_30677280_HHV-7_JI_U43400.1_144861bp_1_2128	ACTGTTCAGACTTTGAAACACACTGTGTAGGACTGACATTCTGATGTACACTTTGGTAGGATTGTTCAGATGTTTCAGAACATTCCTCAGAATACTTTCCATAATCATTAAAATCTTCAA
664157_30677280_HHV-7_JI_U43400.1_144861bp_1_2129	ATTGTTCAGATGTTTCAGAACATTCCTCAGAATACTTTCCATAATCATTAAAATCTTCAATTTCTGGTGGAGTCGAGTCAGAATTATATAAGTGTGACTCAAGCAAAGTGGAAGAGTGAT
664157_30677280_HHV-7_JI_U43400.1_144861bp_1_2130	TTTCTGGTGGAGTCGAGTCAGAATTATATAAGTGTGACTCAAGCAAAGTGGAAGAGTGATGTCCATTTCCTGTGACATATCCATTTCTAATGGATATTGAATTTAACACTGTAACTTCTC
664157_30677280_HHV-7_JI_U43400.1_144861bp_1_2131	GTCCATTTCCTGTGACATATCCATTTCTAATGGATATTGAATTTAACACTGTAACTTCTCCTGTGACATCATATAGCTTTTCTATTTGATCACAAGCATCATTAATCATATGCATAAGTG
664157_30677280_HHV-7_JI_U43400.1_144861bp_1_2132	CTGTGACATCATATAGCTTTTCTATTTGATCACAAGCATCATTAATCATATGCATAAGTGTGATCTTAAATTCTTTTCTATTTGCCCTGAATTTTTTGCATGAAAATTCTTTAGAGCATA
664157_30677280_HHV-7_JI_U43400.1_144861bp_1_2133	TGATCTTAAATTCTTTTCTATTTGCCCTGAATTTTTTGCATGAAAATTCTTTAGAGCATATTACAATACACTGCAGTACAGCAGCTCCTAGGTTTTTTGCTGCCTCTATTAAATTTATAC
664157_30677280_HHV-7_JI_U43400.1_144861bp_1_2134	TTACAATACACTGCAGTACAGCAGCTCCTAGGTTTTTTGCTGCCTCTATTAAATTTATACAATTGAAATGTATTTTAAGGAATGTTTCGTCACACATTGACATGAACATCTGATTCATCT
664157_30677280_HHV-7_JI_U43400.1_144861bp_1_2135	AATTGAAATGTATTTTAAGGAATGTTTCGTCACACATTGACATGAACATCTGATTCATCTGTTTGATTGCAAAGTCTTTCATAGTTTGAGCTATTCTGCTGTTTTTTTCAATTATTTCAA
664157_30677280_HHV-7_JI_U43400.1_144861bp_1_2136	GTTTGATTGCAAAGTCTTTCATAGTTTGAGCTATTCTGCTGTTTTTTTCAATTATTTCAAATTTACATTCCAACAAATCTTTTTTAATCTTATTTGTTTTTATGTAGAATGCCTGATTGG
664157_30677280_HHV-7_JI_U43400.1_144861bp_1_2137	ATTTACATTCCAACAAATCTTTTTTAATCTTATTTGTTTTTATGTAGAATGCCTGATTGGAAATGTTTATATCTGTTATGTCTCTGTTTTTTCCCATCATTAATAACATTGTTGCACATC

664157_30677280_HHV-7_JI_U43400.1_144861bp_1_2138	AAATGTTTATATCTGTTATGTCTCTGTTTTTTCCCATCATTAATAACATTGTTGCACATCTGTTAAGTTTCTCATTACTGCTTATAAAGTTGCCCATACTTATGATCTTTTTTATACATT
664157_30677280_HHV-7_JI_U43400.1_144861bp_1_2139	TGTTAAGTTTCTCATTACTGCTTATAAAGTTGCCCATACTTATGATCTTTTTTATACATTTGATAAATTCTCTATCCATATTATTTCTCAAATCTGATAAATATTCTCTACAATCTGCAA
664157_30677280_HHV-7_JI_U43400.1_144861bp_1_2140	TGATAAATTCTCTATCCATATTATTTCTCAAATCTGATAAATATTCTCTACAATCTGCAATCACTTTTTTAACAATTTCATCAAATTTATCACAGGCATATACAGTGTATCTTGATTCAC
664157_30677280_HHV-7_JI_U43400.1_144861bp_1_2141	TCACTTTTTTAACAATTTCATCAAATTTATCACAGGCATATACAGTGTATCTTGATTCACACCAAAGAAAAAATTCTAATTGTTCTCCAGATAAGATATCTTCATAAAAAGATATAAATC
664157_30677280_HHV-7_JI_U43400.1_144861bp_1_2142	ACCAAAGAAAAAATTCTAATTGTTCTCCAGATAAGATATCTTCATAAAAAGATATAAATCCAGAGGAAGCACCACTAATTAAATTAACATTTAGCATCTGAGATAATTGTTCTAAAGCAA
664157_30677280_HHV-7_JI_U43400.1_144861bp_1_2143	CAGAGGAAGCACCACTAATTAAATTAACATTTAGCATCTGAGATAATTGTTCTAAAGCAAGATCTGTAAGCATGTGTCTGTCATTCGTTAAAATTCCAGCGTGGTTAATTAAACAATCTT
664157_30677280_HHV-7_JI_U43400.1_144861bp_1_2144	GATCTGTAAGCATGTGTCTGTCATTCGTTAAAATTCCAGCGTGGTTAATTAAACAATCTTCAATGTCTGCTTTTTCCTGATCCATGGCAGCATTCACGGATTTAAGAATACCTTGGATGC
664157_30677280_HHV-7_JI_U43400.1_144861bp_1_2145	CAATGTCTGCTTTTTCCTGATCCATGGCAGCATTCACGGATTTAAGAATACCTTGGATGCAGGCACTAATGGACTGAGCTAGAATTTAAAGAAAAAAGTCTAAATATGAAATCTGTACAC
664157_30677280_HHV-7_JI_U43400.1_144861bp_1_2146	AGGCACTAATGGACTGAGCTAGAATTTAAAGAAAAAAGTCTAAATATGAAATCTGTACACATATATCATATTTTATATGTAAGAAAATGAAAAAAAAATACCTGCTGCCAGTTTAATATC
664157_30677280_HHV-7_JI_U43400.1_144861bp_1_2147	ATATATCATATTTTATATGTAAGAAAATGAAAAAAAAATACCTGCTGCCAGTTTAATATCCGAGGAAGCTCCTTCTTCAACATTTGTGGAATAGGAAGCATTTGCCGTTGCAATATCATT
664157_30677280_HHV-7_JI_U43400.1_144861bp_1_2148	CGAGGAAGCTCCTTCTTCAACATTTGTGGAATAGGAAGCATTTGCCGTTGCAATATCATTAGATGATATTGAGTCAAGGAAATCAAAAGAATCCAATTCTTGGAAGAGAGTATTATCAAA
664157_30677280_HHV-7_JI_U43400.1_144861bp_1_2149	AGATGATATTGAGTCAAGGAAATCAAAAGAATCCAATTCTTGGAAGAGAGTATTATCAAAGGAAGGGTTGCATTTTAAACTGTCTTGCAATATGTCTCTGATGGTCTCTTCCACGGTGGT

664157_30677280_HHV-7_JI_U43400.1_144861bp_1_2150	GGAAGGGTTGCATTTTAAACTGTCTTGCAATATGTCTCTGATGGTCTCTTCCACGGTGGTATCTGGGTGATATCTGTAATAAAATTTATAAATTTTATTACAGAGAAATTAAATCTGTCT
664157_30677280_HHV-7_JI_U43400.1_144861bp_1_2151	ATCTGGGTGATATCTGTAATAAAATTTATAAATTTTATTACAGAGAAATTAAATCTGTCTTTTTTGTATTAAAATATTTAGGTTTACCTACTCAATATGTCTTTCGTCATTCGATGCCAT
664157_30677280_HHV-7_JI_U43400.1_144861bp_1_2152	TTTTTGTATTAAAATATTTAGGTTTACCTACTCAATATGTCTTTCGTCATTCGATGCCATGGTTTCAGATGGTTGTCCAACAATCAACATAGGTGAAGTAGCTCCACTTCTTTCCATAGT
664157_30677280_HHV-7_JI_U43400.1_144861bp_1_2153	GGTTTCAGATGGTTGTCCAACAATCAACATAGGTGAAGTAGCTCCACTTCTTTCCATAGTTTTATCTGTATGTTTAGCCACACCTACAATAACAAACCAATCAGAAATATTAACCCCATT
664157_30677280_HHV-7_JI_U43400.1_144861bp_1_2154	TTTATCTGTATGTTTAGCCACACCTACAATAACAAACCAATCAGAAATATTAACCCCATTCCAAACTCAATATAAGGCCAGCCTAAAAATACTTTCAAGCAGAGTTTTAAAACTCTGCTT
664157_30677280_HHV-7_JI_U43400.1_144861bp_1_2155	CCAAACTCAATATAAGGCCAGCCTAAAAATACTTTCAAGCAGAGTTTTAAAACTCTGCTTGAAAAAAATGTATACTCTGGAATATGAAAAACGTGTATCAAGGCCAAAACTTACTTATTG
664157_30677280_HHV-7_JI_U43400.1_144861bp_1_2156	GAAAAAAATGTATACTCTGGAATATGAAAAACGTGTATCAAGGCCAAAACTTACTTATTGGATCATTTTGGCAATATTATTTGTTTTTTTAATAATAACTGGATCTGTATTAATTGTAAT
664157_30677280_HHV-7_JI_U43400.1_144861bp_1_2157	GATCATTTTGGCAATATTATTTGTTTTTTTAATAATAACTGGATCTGTATTAATTGTAATTGAAACTCTGTCAATTCAAAGAACGACTTTGAATGCTCAAAATGATAAAACAAGTACTGT
664157_30677280_HHV-7_JI_U43400.1_144861bp_1_2158	TGAAACTCTGTCAATTCAAAGAACGACTTTGAATGCTCAAAATGATAAAACAAGTACTGTTGTTCCAGAATTAACATCTAATTCTCCAGGTTTGTTCAGCTATAAAATGTCATTGTTTAA
664157_30677280_HHV-7_JI_U43400.1_144861bp_1_2159	TGTTCCAGAATTAACATCTAATTCTCCAGGTTTGTTCAGCTATAAAATGTCATTGTTTAAAAAAAAAAGTTTGTAACTCTGTTAATATGAATATTTTTTCTTGTAGATCAAACAACTGTGA
664157_30677280_HHV-7_JI_U43400.1_144861bp_1_2160	AAAAAAAGTTTGTAACTCTGTTAATATGAATATTTTTTCTTGTAGATCAAACAACTGTGACAAATTTTTCTGCAAGTTCTAAACCAACTCTTAGCAGTAAACAACCCGGATGGATACAGG
664157_30677280_HHV-7_JI_U43400.1_144861bp_1_2161	CAAATTTTTCTGCAAGTTCTAAACCAACTCTTAGCAGTAAACAACCCGGATGGATACAGGCACTAACTACAGCTTTTGGAATTTTAACACTGTTTTCAGTTATGATGATTATTATAACTT

664157_30677280_HHV-7_JI_U43400.1_144861bp_1_2162	CACTAACTACAGCTTTTGGAATTTTAACACTGTTTTCAGTTATGATGATTATTATAACTTGTAATTTTTGGCTAACTGAGAAAAATGACAAAACTGCAAATCCAACAGAATACTACTCAG
664157_30677280_HHV-7_JI_U43400.1_144861bp_1_2163	GTAATTTTTGGCTAACTGAGAAAAATGACAAAACTGCAAATCCAACAGAATACTACTCAGAAGACATATTAGATTATACAAATCCAAGTTTTACAGAAATTGATGAAGACAGCTCAAAAG
664157_30677280_HHV-7_JI_U43400.1_144861bp_1_2164	AAGACATATTAGATTATACAAATCCAAGTTTTACAGAAATTGATGAAGACAGCTCAAAAGTATAACATTGCTTCAGAAATAACAAAATTGTAAGTAAATTTACTTGATTTTCTTACACTA
664157_30677280_HHV-7_JI_U43400.1_144861bp_1_2165	TATAACATTGCTTCAGAAATAACAAAATTGTAAGTAAATTTACTTGATTTTCTTACACTATGAGATATTTAATGGTTTTAAAATAGAAAATGTCAGAGTTTAAAGTAAAATTAATGTAAT
664157_30677280_HHV-7_JI_U43400.1_144861bp_1_2166	TGAGATATTTAATGGTTTTAAAATAGAAAATGTCAGAGTTTAAAGTAAAATTAATGTAATTTATGATTTTTTAAATGTGTCTTACAACTAGAAAATAACTTTAAATTATTATTAGTATTG
664157_30677280_HHV-7_JI_U43400.1_144861bp_1_2167	TTATGATTTTTTAAATGTGTCTTACAACTAGAAAATAACTTTAAATTATTATTAGTATTGTAATAATTTAAAATTATTAATATTAGTATTGTAATAATTTAAAATTAATAATATTAGTAT
664157_30677280_HHV-7_JI_U43400.1_144861bp_1_2168	TAATAATTTAAAATTATTAATATTAGTATTGTAATAATTTAAAATTAATAATATTAGTATTATAAACAGTTTTTAAAATATAAAAAAAATAAATTTAAAATTTATATTAAATGATTTTAA
664157_30677280_HHV-7_JI_U43400.1_144861bp_1_2169	TATAAACAGTTTTTAAAATATAAAAAAAATAAATTTAAAATTTATATTAAATGATTTTAAATTTACTTACCAGATGGCAGCGATATATTGGAAATTCTGTGTAGCAGAACTGTATCTTCA
664157_30677280_HHV-7_JI_U43400.1_144861bp_1_2170	ATTTACTTACCAGATGGCAGCGATATATTGGAAATTCTGTGTAGCAGAACTGTATCTTCAAGCTCTTGAAGAAAAAGTGGCAAAATAGTTGATCTGTCTTCTTATTATATACATTTTGAA
664157_30677280_HHV-7_JI_U43400.1_144861bp_1_2171	AGCTCTTGAAGAAAAAGTGGCAAAATAGTTGATCTGTCTTCTTATTATATACATTTTGAAGCGGATGTGATGCACAGGAAAATGACGTTAATATATCAACCAATTAAAAAAAGCCAAAAA
664157_30677280_HHV-7_JI_U43400.1_144861bp_1_2172	GCGGATGTGATGCACAGGAAAATGACGTTAATATATCAACCAATTAAAAAAAGCCAAAAAATTATAATACTAAAAAAATTAGGGTTTAACCTTATTTGTACATGTATTTTTTTAAAATAT
664157_30677280_HHV-7_JI_U43400.1_144861bp_1_2173	ATTATAATACTAAAAAAATTAGGGTTTAACCTTATTTGTACATGTATTTTTTTAAAATATTATTTTTCCAATAAAAACCACAATGGATTTATATTTTACGTAAACATGATTTTTGCGGTT

664157_30677280_HHV-7_JI_U43400.1_144861bp_1_2174	TATTTTTCCAATAAAAACCACAATGGATTTATATTTTACGTAAACATGATTTTTGCGGTTATTTTATTTCGAATTGGTATTTAAATCTTTGAAACAACAGGAATTGCGGTTGTCTTCTCA
664157_30677280_HHV-7_JI_U43400.1_144861bp_1_2175	ATTTTATTTCGAATTGGTATTTAAATCTTTGAAACAACAGGAATTGCGGTTGTCTTCTCAGCAAGTTCTACACATTCCCACTCGCTTTAGGCAGGAAAGACCCCAACCACATAACCCACT
664157_30677280_HHV-7_JI_U43400.1_144861bp_1_2176	GCAAGTTCTACACATTCCCACTCGCTTTAGGCAGGAAAGACCCCAACCACATAACCCACTGACATTTAAACCCGTGAAAACAACAGGAACTGCGGTTGCCTTCTCAGCAGGTTTTACGGG
664157_30677280_HHV-7_JI_U43400.1_144861bp_1_2177	GACATTTAAACCCGTGAAAACAACAGGAACTGCGGTTGCCTTCTCAGCAGGTTTTACGGGTTCTCACCCGTTTAGGCAGGAAAGACCCCAACCACATAACCCACTAACATTTTAATATGT
664157_30677280_HHV-7_JI_U43400.1_144861bp_1_2178	TTCTCACCCGTTTAGGCAGGAAAGACCCCAACCACATAACCCACTAACATTTTAATATGTGAAAACAACAGGAACTGCGGTTGCCTTCTCAGCAGGTTTTACGGGTTCTCACCCGTTTAG
664157_30677280_HHV-7_JI_U43400.1_144861bp_1_2179	GAAAACAACAGGAACTGCGGTTGCCTTCTCAGCAGGTTTTACGGGTTCTCACCCGTTTAGGCAGGAAAGACCCCAACCACATAACCCACTGACATTTAAACCCGTGAAAACAACAGGAAC
664157_30677280_HHV-7_JI_U43400.1_144861bp_1_2180	GCAGGAAAGACCCCAACCACATAACCCACTGACATTTAAACCCGTGAAAACAACAGGAACTGCGGTTGCCTTCTCAGCAGGTTTTATGAGTTCTCACCCGTTTAGGCAGGAAAGACCCCA
664157_30677280_HHV-7_JI_U43400.1_144861bp_1_2181	TGCGGTTGCCTTCTCAGCAGGTTTTATGAGTTCTCACCCGTTTAGGCAGGAAAGACCCCAACCACATAACCCACTGACATTTAAACCCGTGAAAACAACAGGAACTGCGGTTGCCTTCTC
664157_30677280_HHV-7_JI_U43400.1_144861bp_1_2182	ACCACATAACCCACTGACATTTAAACCCGTGAAAACAACAGGAACTGCGGTTGCCTTCTCAGCAGGTTTTATGGGTTCTCACCCGTTTAGGCAGGAAAGACCCCAACCACATAACCCACT
664157_30677280_HHV-7_JI_U43400.1_144861bp_1_2183	AGCAGGTTTTATGGGTTCTCACCCGTTTAGGCAGGAAAGACCCCAACCACATAACCCACTGACATTTAAACCCGTGAAAACAACAGGAACTGCGGTTGTCTTCTCAGCAGGTTTTAGAGT
664157_30677280_HHV-7_JI_U43400.1_144861bp_1_2184	GACATTTAAACCCGTGAAAACAACAGGAACTGCGGTTGTCTTCTCAGCAGGTTTTAGAGTTCTCACCCGTTTAGGCAGGAAAGACCCCAACCACATAACCCACTGACATTTAAACCCGTG
664157_30677280_HHV-7_JI_U43400.1_144861bp_1_2185	TCTCACCCGTTTAGGCAGGAAAGACCCCAACCACATAACCCACTGACATTTAAACCCGTGAAAACAACAGGAACTGCGGTTGCCTTCTCAGCAGGTTTTATGGGTTCTCACCCGTTTAGG

664157_30677280_HHV-7_JI_U43400.1_144861bp_1_2186	AAAACAACAGGAACTGCGGTTGCCTTCTCAGCAGGTTTTATGGGTTCTCACCCGTTTAGGCAGGAAAGACCCCAACCACATAACCCACTGACATTTAAACCCGTGAAAACAACAGGAACT
664157_30677280_HHV-7_JI_U43400.1_144861bp_1_2187	CAGGAAAGACCCCAACCACATAACCCACTGACATTTAAACCCGTGAAAACAACAGGAACTGCGGTTGTCTTCTCAGCAGGTTTTATGAGTTCTCACCCGTTTAGGCAGGAAAGACCCCAA
664157_30677280_HHV-7_JI_U43400.1_144861bp_1_2188	GCGGTTGTCTTCTCAGCAGGTTTTATGAGTTCTCACCCGTTTAGGCAGGAAAGACCCCAACCACATAACCCACTGACATTTAAACCCGTGAAAACAACAGGAACTGCGGTTGCCTTCTCA
664157_30677280_HHV-7_JI_U43400.1_144861bp_1_2189	CCACATAACCCACTGACATTTAAACCCGTGAAAACAACAGGAACTGCGGTTGCCTTCTCAGCAGGTTTTATGAGTTCTCACCCGTTTAGGCAGGAAAGACCCCAACCACATAACCCACTG
664157_30677280_HHV-7_JI_U43400.1_144861bp_1_2190	GCAGGTTTTATGAGTTCTCACCCGTTTAGGCAGGAAAGACCCCAACCACATAACCCACTGACATTTAAACCCGTGAAAACAACAGGAACTGCGGTTGCCTTCTCAGCAGGTTTTATGGGT
664157_30677280_HHV-7_JI_U43400.1_144861bp_1_2191	ACATTTAAACCCGTGAAAACAACAGGAACTGCGGTTGCCTTCTCAGCAGGTTTTATGGGTTCTCACCCGTTTAGGCAGGAAAGACCCCAACCACATAACCCACTGACATTTAAACCCGTG
664157_30677280_HHV-7_JI_U43400.1_144861bp_1_2192	TCTCACCCGTTTAGGCAGGAAAGACCCCAACCACATAACCCACTGACATTTAAACCCGTGAAAACAACAGGAACTGCGGTTGTCTTCTCAGCAGGTTTTATGAGTTCTCACCCGTTTAGG
664157_30677280_HHV-7_JI_U43400.1_144861bp_1_2193	AAAACAACAGGAACTGCGGTTGTCTTCTCAGCAGGTTTTATGAGTTCTCACCCGTTTAGGCAGGAAAGACCCCAACCACATAACCCACTGACATTTAAACCCGTGAAAACAACAGGAACT
664157_30677280_HHV-7_JI_U43400.1_144861bp_1_2194	CAGGAAAGACCCCAACCACATAACCCACTGACATTTAAACCCGTGAAAACAACAGGAACTGCGGTTGCCTTCTCAGCAGGTTTTATGGGTTCTCACCCGTTTAGGCAGGAAAGACCCCAA
664157_30677280_HHV-7_JI_U43400.1_144861bp_1_2195	GCGGTTGCCTTCTCAGCAGGTTTTATGGGTTCTCACCCGTTTAGGCAGGAAAGACCCCAACCACATAATCCACTGACATTTAAACCCGTGAAAACAACAGGAACTGCGGTTGCCTTCTCA
664157_30677280_HHV-7_JI_U43400.1_144861bp_1_2196	CCACATAATCCACTGACATTTAAACCCGTGAAAACAACAGGAACTGCGGTTGCCTTCTCAGCAGGTTTTATGGGTTCTCACCCGTTTAGGCAGGAAAGACCCGAACCACATAACCCACTG
664157_30677280_HHV-7_JI_U43400.1_144861bp_1_2197	GCAGGTTTTATGGGTTCTCACCCGTTTAGGCAGGAAAGACCCGAACCACATAACCCACTGACATTTAAACCCGTGAAAACAACAGGAACTGCGGTTGCCTTCTCAGCAGGTTTTATGGGT

664157_30677280_HHV-7_JI_U43400.1_144861bp_1_2198	ACATTTAAACCCGTGAAAACAACAGGAACTGCGGTTGCCTTCTCAGCAGGTTTTATGGGTTCTCATCCGTTTAGGCAGGAAAGACCCCGACCACATAACCCACTGACATTTAAACCCGTG
664157_30677280_HHV-7_JI_U43400.1_144861bp_1_2199	TCTCATCCGTTTAGGCAGGAAAGACCCCGACCACATAACCCACTGACATTTAAACCCGTGAAAACAACAGGAACTGCGGTTGCCTTCTCAGCAGGTTTTATGGGTTCTCACCCGTTTAGG
664157_30677280_HHV-7_JI_U43400.1_144861bp_1_2200	AAAACAACAGGAACTGCGGTTGCCTTCTCAGCAGGTTTTATGGGTTCTCACCCGTTTAGGCAGGAAACACCCAGACCGCATAACCCACTGACATTTAAACCCGTGAAAACAACAGGAACT
664157_30677280_HHV-7_JI_U43400.1_144861bp_1_2201	CAGGAAACACCCAGACCGCATAACCCACTGACATTTAAACCCGTGAAAACAACAGGAACTGCGGTTGACTTCTCAGCAGGTTTTATGGGTTCTCACCCGTTTAGGTAGGAAAGACCCAGA
664157_30677280_HHV-7_JI_U43400.1_144861bp_1_2202	GCGGTTGACTTCTCAGCAGGTTTTATGGGTTCTCACCCGTTTAGGTAGGAAAGACCCAGACCGCATAACCCACTAACATTTTAATATGTGAAAATAAACAGGAACTGCGGTTATAGTTTC
664157_30677280_HHV-7_JI_U43400.1_144861bp_1_2203	CCGCATAACCCACTAACATTTTAATATGTGAAAATAAACAGGAACTGCGGTTATAGTTTCAGTACATTCTAGAGATTACATCCTGCTAAGGCGAAAAACACTTTAACCGCAAAAGCCACT
664157_30677280_HHV-7_JI_U43400.1_144861bp_1_2204	AGTACATTCTAGAGATTACATCCTGCTAAGGCGAAAAACACTTTAACCGCAAAAGCCACTGATTTTTAAACTTGTGAAAATAACAGGAAACCACAGAAACGTCAGGAAAAAAACGTTGTT
664157_30677280_HHV-7_JI_U43400.1_144861bp_1_2205	GATTTTTAAACTTGTGAAAATAACAGGAAACCACAGAAACGTCAGGAAAAAAACGTTGTTTTATAATTATGTCCATAAAACATGCAACATAATCCAAACCGGCCAATGATTTTTCTGCTT
664157_30677280_HHV-7_JI_U43400.1_144861bp_1_2206	TTATAATTATGTCCATAAAACATGCAACATAATCCAAACCGGCCAATGATTTTTCTGCTTAAATGTAATTTAAATAATTTATTCATACGTAAGTTGTTATAGCCACGCCTACGCAGCAAT
664157_30677280_HHV-7_JI_U43400.1_144861bp_1_2207	AAATGTAATTTAAATAATTTATTCATACGTAAGTTGTTATAGCCACGCCTACGCAGCAATGTATATAAGCAGACACCGTCATTTCGCAGTTAGTCTGCTGGAAAGCTTGTGAAGACTACA
664157_30677280_HHV-7_JI_U43400.1_144861bp_1_2208	GTATATAAGCAGACACCGTCATTTCGCAGTTAGTCTGCTGGAAAGCTTGTGAAGACTACATAAAGGTAAAAATTTGACATTTGCAAATGTATTTTATAATTACATTATTTTAGTTTAAAA
664157_30677280_HHV-7_JI_U43400.1_144861bp_1_2209	TAAAGGTAAAAATTTGACATTTGCAAATGTATTTTATAATTACATTATTTTAGTTTAAAAAATCCAAAATTCTTAATAGAAATTTATTGTGAAATTTAAAAGATTTTGTAAATATGCATT

664157_30677280_HHV-7_JI_U43400.1_144861bp_1_2210	AATCCAAAATTCTTAATAGAAATTTATTGTGAAATTTAAAAGATTTTGTAAATATGCATTAATATCTATTAAATAATTGTTATTTGACAGATTTACTAAATGTAGTGTAAAATATCAAAA
664157_30677280_HHV-7_JI_U43400.1_144861bp_1_2211	AATATCTATTAAATAATTGTTATTTGACAGATTTACTAAATGTAGTGTAAAATATCAAAAAAATTTAAAATTAATTTATTCTGAAGTTTAACATTTTTTAAATATGTATTTAATTATTAC
664157_30677280_HHV-7_JI_U43400.1_144861bp_1_2212	AAATTTAAAATTAATTTATTCTGAAGTTTAACATTTTTTAAATATGTATTTAATTATTACTGTTTCATCTATTTTTAGTTTCATTTGATACATTGCGTAGGTTTGGAAATGTGTTTCGAA
664157_30677280_HHV-7_JI_U43400.1_144861bp_1_2213	TGTTTCATCTATTTTTAGTTTCATTTGATACATTGCGTAGGTTTGGAAATGTGTTTCGAAAATTGCTGTAAAAATTATTGACTCTTTTTTAAATTTATAGAACATTGGATGTAATTGCAC
664157_30677280_HHV-7_JI_U43400.1_144861bp_1_2214	AATTGCTGTAAAAATTATTGACTCTTTTTTAAATTTATAGAACATTGGATGTAATTGCACATTCCTGGAAAATTCAAAAAAAGAAAATACAGTAAGTCAATTTATATGTATTGATTTTAA
664157_30677280_HHV-7_JI_U43400.1_144861bp_1_2215	ATTCCTGGAAAATTCAAAAAAAGAAAATACAGTAAGTCAATTTATATGTATTGATTTTAAATGGCCTCCAATAAATATTTTTAGCCAGTTTAATGTAAAACAAAGCATAAAGTTTATGTA
664157_30677280_HHV-7_JI_U43400.1_144861bp_1_2216	ATGGCCTCCAATAAATATTTTTAGCCAGTTTAATGTAAAACAAAGCATAAAGTTTATGTAGTTTTAAAATGATGCCATATTTTAACGAAATTCATTTAAAGACAGTATCTATTAAGAATT
664157_30677280_HHV-7_JI_U43400.1_144861bp_1_2217	GTTTTAAAATGATGCCATATTTTAACGAAATTCATTTAAAGACAGTATCTATTAAGAATTTAAATGCAGTTCTATGGCTTTAAAAATTTGTACGTTTTATTTGAAAAGTTTGCTCTATTT
664157_30677280_HHV-7_JI_U43400.1_144861bp_1_2218	TAAATGCAGTTCTATGGCTTTAAAAATTTGTACGTTTTATTTGAAAAGTTTGCTCTATTTGAAATCAAATGTTTTAGATATTTCTTTTCATAAATTCAGTTGAAATATGTTTCATTTATA
664157_30677280_HHV-7_JI_U43400.1_144861bp_1_2219	GAAATCAAATGTTTTAGATATTTCTTTTCATAAATTCAGTTGAAATATGTTTCATTTATAATAGGTATTTTTCTGTTTCTAAAAATAGATGATAAAAGTCACTAGATAGAAAATATCTTA
664157_30677280_HHV-7_JI_U43400.1_144861bp_1_2220	ATAGGTATTTTTCTGTTTCTAAAAATAGATGATAAAAGTCACTAGATAGAAAATATCTTATAAATTGTTATTTAGTAGAATAAACATCTTTTTATATAACAAAAACACAAAGTACATGAC
664157_30677280_HHV-7_JI_U43400.1_144861bp_1_2221	TAAATTGTTATTTAGTAGAATAAACATCTTTTTATATAACAAAAACACAAAGTACATGACTTTAGAAAGCCAACTAAATGCATTATTATTTAAAAATTACAGAATCAACGAGTAATGTCA

664157_30677280_HHV-7_JI_U43400.1_144861bp_1_2222	TTTAGAAAGCCAACTAAATGCATTATTATTTAAAAATTACAGAATCAACGAGTAATGTCACTATAGTTTTTTAAAGTAACTTTTAATTGTTCACTTCTAGTTGTAATCAAAAGAAGGATG
664157_30677280_HHV-7_JI_U43400.1_144861bp_1_2223	CTATAGTTTTTTAAAGTAACTTTTAATTGTTCACTTCTAGTTGTAATCAAAAGAAGGATGGAGAGTGGGGATAGTTTCGGAAATAATCACCAAGTCTCATCGAATTCAGATGCTTTTCAA
664157_30677280_HHV-7_JI_U43400.1_144861bp_1_2224	GAGAGTGGGGATAGTTTCGGAAATAATCACCAAGTCTCATCGAATTCAGATGCTTTTCAATTCCGGCAATATTCAACAACTGTAGATACTTTTGCATATTCATCAATGGATCCTTCAAAT
664157_30677280_HHV-7_JI_U43400.1_144861bp_1_2225	TTCCGGCAATATTCAACAACTGTAGATACTTTTGCATATTCATCAATGGATCCTTCAAATTGCATGCTTAATGAACAAACTCATTTAGAAACGGTACTAGTTTATCCTGTTTACAACATG
664157_30677280_HHV-7_JI_U43400.1_144861bp_1_2226	TGCATGCTTAATGAACAAACTCATTTAGAAACGGTACTAGTTTATCCTGTTTACAACATGTCTTCCCAGCAAACAGTGGAGAGAAATCTCTATGCCAATAATATAGGAAATGGAAATGTG
664157_30677280_HHV-7_JI_U43400.1_144861bp_1_2227	TCTTCCCAGCAAACAGTGGAGAGAAATCTCTATGCCAATAATATAGGAAATGGAAATGTGCTGAGTTATTGTAGACAAGATAGTTTGCAAGGAAACCAAGGAACATATTTTCCAACATAT
664157_30677280_HHV-7_JI_U43400.1_144861bp_1_2228	CTGAGTTATTGTAGACAAGATAGTTTGCAAGGAAACCAAGGAACATATTTTCCAACATATCAGGGATTTCACTCAAACACTCCTCATAACTTTTCTATAGAGAATTTTAATTTTGGCACC
664157_30677280_HHV-7_JI_U43400.1_144861bp_1_2229	CAGGGATTTCACTCAAACACTCCTCATAACTTTTCTATAGAGAATTTTAATTTTGGCACCAATGTCATCAGGCCTATACCATTTAGAAATGAGGGAGTAGACGCATCAACATCCAAAGAT
664157_30677280_HHV-7_JI_U43400.1_144861bp_1_2230	AATGTCATCAGGCCTATACCATTTAGAAATGAGGGAGTAGACGCATCAACATCCAAAGATTATGGCTTTAAACCTTGCATTACAACGATGCAAATACAGTTTAGTCAGCCATCTGCTTTT
664157_30677280_HHV-7_JI_U43400.1_144861bp_1_2231	TATGGCTTTAAACCTTGCATTACAACGATGCAAATACAGTTTAGTCAGCCATCTGCTTTTCAAACTTATTCTTTAATGAATGGAAATTTTAGTGAAAATGGTTATGGATACAATACTAAG
664157_30677280_HHV-7_JI_U43400.1_144861bp_1_2232	CAAACTTATTCTTTAATGAATGGAAATTTTAGTGAAAATGGTTATGGATACAATACTAAGATTGGTGAAAATTGTGCTATGTCTTATGTTGAGCAAGAGTCTGTAGACTCAAAATATCCA
664157_30677280_HHV-7_JI_U43400.1_144861bp_1_2233	ATTGGTGAAAATTGTGCTATGTCTTATGTTGAGCAAGAGTCTGTAGACTCAAAATATCCAAACAAAGAAGTTAGTATCTCTTATCAAAGAAATGAACATTATGATGAGAATCTCCCGACG

664157_30677280_HHV-7_JI_U43400.1_144861bp_1_2234	AACAAAGAAGTTAGTATCTCTTATCAAAGAAATGAACATTATGATGAGAATCTCCCGACGAGAGAGCTTAATACAGATTTTCCGCAATATAATCAAAGTTCTGTGTTACAGCACACCTTT
664157_30677280_HHV-7_JI_U43400.1_144861bp_1_2235	AGAGAGCTTAATACAGATTTTCCGCAATATAATCAAAGTTCTGTGTTACAGCACACCTTTGCGAATGTCCAAAGCATTATTTCACAGCAAAGTCCATATCAGTTGATTGGAAAAGAAAAC
664157_30677280_HHV-7_JI_U43400.1_144861bp_1_2236	GCGAATGTCCAAAGCATTATTTCACAGCAAAGTCCATATCAGTTGATTGGAAAAGAAAACAGCTTTAACAACTATCTGGAAACAGCTAACGTTGATAGTGATTGTCAATCTAACGGGGGG
664157_30677280_HHV-7_JI_U43400.1_144861bp_1_2237	AGCTTTAACAACTATCTGGAAACAGCTAACGTTGATAGTGATTGTCAATCTAACGGGGGGCCTAAAATTGGCCTAGACACAGATCAATCTGTTTTTTCAGATGAAGTGACTGGGGCGTGT
664157_30677280_HHV-7_JI_U43400.1_144861bp_1_2238	CCTAAAATTGGCCTAGACACAGATCAATCTGTTTTTTCAGATGAAGTGACTGGGGCGTGTGTAGAAAATGTTCATTTTCCAATGAATCCTAAAAATGAGTATGTAAATAACATATCACTT
664157_30677280_HHV-7_JI_U43400.1_144861bp_1_2239	GTAGAAAATGTTCATTTTCCAATGAATCCTAAAAATGAGTATGTAAATAACATATCACTTGATGTACAAACTGAATTTCCTGTGACTGAGAATCCTCTTGTAGGAGAAAGTCAGGCTACA
664157_30677280_HHV-7_JI_U43400.1_144861bp_1_2240	GATGTACAAACTGAATTTCCTGTGACTGAGAATCCTCTTGTAGGAGAAAGTCAGGCTACAAAGAACAAGGATGTGGACAGTAACGCAGAGGTTAATAATCATTCTAAATATCGTCTATTG
664157_30677280_HHV-7_JI_U43400.1_144861bp_1_2241	AAGAACAAGGATGTGGACAGTAACGCAGAGGTTAATAATCATTCTAAATATCGTCTATTGAAACGCAATATTACACCGACCATGGGTAATATTAAACATTTAAATTTTAGTTGCAAGATA
664157_30677280_HHV-7_JI_U43400.1_144861bp_1_2242	AAACGCAATATTACACCGACCATGGGTAATATTAAACATTTAAATTTTAGTTGCAAGATATCTACCGAAGAAGAAAGGAAAACATTTTTTAACAGGTTGAGTGAGCTATTGAAAATAAGG
664157_30677280_HHV-7_JI_U43400.1_144861bp_1_2243	TCTACCGAAGAAGAAAGGAAAACATTTTTTAACAGGTTGAGTGAGCTATTGAAAATAAGGGATGATATAAAAAACACTAAAATTTCTTCAAAAGTGGATTTTATTCAATCTGAAGCTTCT
664157_30677280_HHV-7_JI_U43400.1_144861bp_1_2244	GATGATATAAAAAACACTAAAATTTCTTCAAAAGTGGATTTTATTCAATCTGAAGCTTCTACATCATTGGACATTTGTAAAAATACATTCAATAACAATTCGGATTCATCGGATGTGGAC
664157_30677280_HHV-7_JI_U43400.1_144861bp_1_2245	ACATCATTGGACATTTGTAAAAATACATTCAATAACAATTCGGATTCATCGGATGTGGACACTGATATCCTTGCAGACAACCCATTAGTGATATGTGAAAATGAACTGATTTGTGATAAC

664157_30677280_HHV-7_JI_U43400.1_144861bp_1_2246	ACTGATATCCTTGCAGACAACCCATTAGTGATATGTGAAAATGAACTGATTTGTGATAACAATGAGGAAAACATCAAGTTTCCGCCTAACGTTGAAAAAGAGGCTGTCCCCATGCAGACG
664157_30677280_HHV-7_JI_U43400.1_144861bp_1_2247	AATGAGGAAAACATCAAGTTTCCGCCTAACGTTGAAAAAGAGGCTGTCCCCATGCAGACGGTTAAACGCAGCTTTCCAGAAATCTGTCCAGAGCATTTTAAAAAAAGAAGATTTATAAAT
664157_30677280_HHV-7_JI_U43400.1_144861bp_1_2248	GTTAAACGCAGCTTTCCAGAAATCTGTCCAGAGCATTTTAAAAAAAGAAGATTTATAAATGGTGATGTGATTTATGAAGATTTAAATTCTGTTTATAAAGTGATGCCAGCAAGTGCGACA
664157_30677280_HHV-7_JI_U43400.1_144861bp_1_2249	GGTGATGTGATTTATGAAGATTTAAATTCTGTTTATAAAGTGATGCCAGCAAGTGCGACATATGACGATGTGCGTTTCGGAGAAGTGGATTATCAAACCTCTTCGGCTCAGACAAAAATT
664157_30677280_HHV-7_JI_U43400.1_144861bp_1_2250	TATGACGATGTGCGTTTCGGAGAAGTGGATTATCAAACCTCTTCGGCTCAGACAAAAATTTCCAACCACCAGTTAGCGTTGTTGCCAACAAATTATCAGCACATGATAGGGCAGGAAACA
664157_30677280_HHV-7_JI_U43400.1_144861bp_1_2251	TCCAACCACCAGTTAGCGTTGTTGCCAACAAATTATCAGCACATGATAGGGCAGGAAACAGACATTTCATCGCGGGATTATCACAATGACAGTGCTCAAATTATTTACAATTGCTCATTT
664157_30677280_HHV-7_JI_U43400.1_144861bp_1_2252	GACATTTCATCGCGGGATTATCACAATGACAGTGCTCAAATTATTTACAATTGCTCATTTCAACGACAGGGAAAAAGGCTTCTTGCTGATATTCCATATAGACCTTGGTTAAAAGAAAAT
664157_30677280_HHV-7_JI_U43400.1_144861bp_1_2253	CAACGACAGGGAAAAAGGCTTCTTGCTGATATTCCATATAGACCTTGGTTAAAAGAAAATGTTCCCACAGGGGAATATGGAAAAAGATTTGTCAAAACAGTCCCTAGACCAGCCAGTGTT
664157_30677280_HHV-7_JI_U43400.1_144861bp_1_2254	GTTCCCACAGGGGAATATGGAAAAAGATTTGTCAAAACAGTCCCTAGACCAGCCAGTGTTTTTATTTTTGGAAGATGTCGAAAAAAAATGCTGAGGGAAAGCGTTAAATCTTTTATGCAT
664157_30677280_HHV-7_JI_U43400.1_144861bp_1_2255	TTTATTTTTGGAAGATGTCGAAAAAAAATGCTGAGGGAAAGCGTTAAATCTTTTATGCATTTAAGCAAGTTACAGTATACTAAAGAAAGTCTAGAAGCATATGTACTTAGAAATTGTAAC
664157_30677280_HHV-7_JI_U43400.1_144861bp_1_2256	TTAAGCAAGTTACAGTATACTAAAGAAAGTCTAGAAGCATATGTACTTAGAAATTGTAACAAGTTTTTAGATTTGAGTTGGCCGATACGACACAAGATCTATATTATGCCTGATGTGAGT
664157_30677280_HHV-7_JI_U43400.1_144861bp_1_2257	AAGTTTTTAGATTTGAGTTGGCCGATACGACACAAGATCTATATTATGCCTGATGTGAGTAGAAATTATAACATTGAAGAGGTTAAGAATTTATTTCCCGTACCGGAAGGCTGGATGGTA

664157_30677280_HHV-7_JI_U43400.1_144861bp_1_2258	AGAAATTATAACATTGAAGAGGTTAAGAATTTATTTCCCGTACCGGAAGGCTGGATGGTAACTCTAGGTTTTGTAGGAACAGAAGAACCGGTGACCAAAATTTATAACATTGCTGTATTG
664157_30677280_HHV-7_JI_U43400.1_144861bp_1_2259	ACTCTAGGTTTTGTAGGAACAGAAGAACCGGTGACCAAAATTTATAACATTGCTGTATTGTTATGTGAAAATGGGTGGGTCATGATTCACAAGAATGATAAAGTGGAACCTGAACTTTAT
664157_30677280_HHV-7_JI_U43400.1_144861bp_1_2260	TTATGTGAAAATGGGTGGGTCATGATTCACAAGAATGATAAAGTGGAACCTGAACTTTATTTAGCTGCTTCGAATCTCAATGAACTTATAGAGGATGGACTGGCACGCTGTGATTGTATT
664157_30677280_HHV-7_JI_U43400.1_144861bp_1_2261	TTAGCTGCTTCGAATCTCAATGAACTTATAGAGGATGGACTGGCACGCTGTGATTGTATTTACGAGAAAAGGTCTGTACCATATGGAATTGTCATGGAAGGAAAATTAAGACAGTTTATG
664157_30677280_HHV-7_JI_U43400.1_144861bp_1_2262	TACGAGAAAAGGTCTGTACCATATGGAATTGTCATGGAAGGAAAATTAAGACAGTTTATGGACAATTTTGGCTCACTGCAAAGTGTGCTAGCCTACAGAAAGTATTTACATGGTTTTCTG
664157_30677280_HHV-7_JI_U43400.1_144861bp_1_2263	GACAATTTTGGCTCACTGCAAAGTGTGCTAGCCTACAGAAAGTATTTACATGGTTTTCTGTGGGCTTTTAATGGCACACCTGGAAGATTGGCAGACAGAGTGTTCCATACTTGTGTACCT
664157_30677280_HHV-7_JI_U43400.1_144861bp_1_2264	TGGGCTTTTAATGGCACACCTGGAAGATTGGCAGACAGAGTGTTCCATACTTGTGTACCTGGGGTTCATAATGCCCTTCCCTTAGATGCTGTGATTAAACATGAAAATAATCCCTTGTAT
664157_30677280_HHV-7_JI_U43400.1_144861bp_1_2265	GGGGTTCATAATGCCCTTCCCTTAGATGCTGTGATTAAACATGAAAATAATCCCTTGTATTTCATTGGATATGTAACCACCTTTAAACAGCAAAATGATTTCAATGCTAATGTTTTCATC
664157_30677280_HHV-7_JI_U43400.1_144861bp_1_2266	TTCATTGGATATGTAACCACCTTTAAACAGCAAAATGATTTCAATGCTAATGTTTTCATCGCGGTAGATGGAAATTTAAGCATTTATGGCTATCATCTTATATCTCAAAAAACTTGGTTT
664157_30677280_HHV-7_JI_U43400.1_144861bp_1_2267	GCGGTAGATGGAAATTTAAGCATTTATGGCTATCATCTTATATCTCAAAAAACTTGGTTTCTAGCAAAAACATTTTCAACTTTTCTGAAAATGGGAACTAGAAAGATGTACTATGATTAT
664157_30677280_HHV-7_JI_U43400.1_144861bp_1_2268	CTAGCAAAAACATTTTCAACTTTTCTGAAAATGGGAACTAGAAAGATGTACTATGATTATGAGATTCCACTTAAAATTCATTTAGGAGATAGTGCAGATTCATTTCTGTCTTGTTTTAAA
664157_30677280_HHV-7_JI_U43400.1_144861bp_1_2269	GAGATTCCACTTAAAATTCATTTAGGAGATAGTGCAGATTCATTTCTGTCTTGTTTTAAAAATGCCCCTTGTCTGCTGCTTAAGCCAGAAGTACTTAGAAAGCAATTTCCTAAACCTTAG

664157_30677280_HHV-7_JI_U43400.1_144861bp_1_2270	AATGCCCCTTGTCTGCTGCTTAAGCCAGAAGTACTTAGAAAGCAATTTCCTAAACCTTAGATTGTTAAAACTTGGATAACCAAACTATATTGAATTATGTATATCTTTTTATTGAATGGA
664157_30677280_HHV-7_JI_U43400.1_144861bp_1_2271	ATTGTTAAAACTTGGATAACCAAACTATATTGAATTATGTATATCTTTTTATTGAATGGAACATGTTTGTAAACATTTTTGAAGCTTAAATAAAGAGTGTTTTCAAATATCTCTGTTTTG
664157_30677280_HHV-7_JI_U43400.1_144861bp_1_2272	ACATGTTTGTAAACATTTTTGAAGCTTAAATAAAGAGTGTTTTCAAATATCTCTGTTTTGTCTCTTTACAGAGGACTTGAAAAATTTTTTACTGTCTTTAGAACTTGCGAATACTTGTTG
664157_30677280_HHV-7_JI_U43400.1_144861bp_1_2273	TCTCTTTACAGAGGACTTGAAAAATTTTTTACTGTCTTTAGAACTTGCGAATACTTGTTGAGCTTTTCTTTCCGGGTAACGCATCTCCACAGTGGTGCAGGTCGCTTCGTAAATATAATC
664157_30677280_HHV-7_JI_U43400.1_144861bp_1_2274	AGCTTTTCTTTCCGGGTAACGCATCTCCACAGTGGTGCAGGTCGCTTCGTAAATATAATCAAAAAATTCTTCTACTTCTAGTAGTTTTGGTGTCGGATCTTCATTGGCGCCATAATTTGA
664157_30677280_HHV-7_JI_U43400.1_144861bp_1_2275	AAAAAATTCTTCTACTTCTAGTAGTTTTGGTGTCGGATCTTCATTGGCGCCATAATTTGAATTAATTCTAGCAGCTTTTATTCCTTCCCAACTAAAAGAGAAAATGTTTAAATTACAGTA
664157_30677280_HHV-7_JI_U43400.1_144861bp_1_2276	ATTAATTCTAGCAGCTTTTATTCCTTCCCAACTAAAAGAGAAAATGTTTAAATTACAGTAAATTGCAACTGGCATTCCTTGGAAAAATGATGCTCACCTGAAGGCTTTGTTTAAATCAAT
664157_30677280_HHV-7_JI_U43400.1_144861bp_1_2277	AATTGCAACTGGCATTCCTTGGAAAAATGATGCTCACCTGAAGGCTTTGTTTAAATCAATCCACGGTGGCATTGGTTGAGTCTTTGTAGGTTTGTTGTTAGGCGTAGGTTTAATTTCATG
664157_30677280_HHV-7_JI_U43400.1_144861bp_1_2278	CCACGGTGGCATTGGTTGAGTCTTTGTAGGTTTGTTGTTAGGCGTAGGTTTAATTTCATGTATGATTGTTGTACTCGTATATATAGGAGTGCAGTATGTAATACCTCCGGCCTGATATAT
664157_30677280_HHV-7_JI_U43400.1_144861bp_1_2279	TATGATTGTTGTACTCGTATATATAGGAGTGCAGTATGTAATACCTCCGGCCTGATATATGCTGCGAACGAGAAATTAAGAAATGAGTTTGTTTCTAAGACGAAAAGTATTGTCCCATAC
664157_30677280_HHV-7_JI_U43400.1_144861bp_1_2280	GCTGCGAACGAGAAATTAAGAAATGAGTTTGTTTCTAAGACGAAAAGTATTGTCCCATACAGTAAAAACTCACATGTTTCCGTCGTCACATGTCAGTTCTATCACATCACCGTCTGTAAA
664157_30677280_HHV-7_JI_U43400.1_144861bp_1_2281	AGTAAAAACTCACATGTTTCCGTCGTCACATGTCAGTTCTATCACATCACCGTCTGTAAATCGCAAGTATTCATCACAGCAGGATTCTAGTTTAGGTAATTGAAGTTCGCTAGCTAGAAT

664157_30677280_HHV-7_JI_U43400.1_144861bp_1_2282	TCGCAAGTATTCATCACAGCAGGATTCTAGTTTAGGTAATTGAAGTTCGCTAGCTAGAATTAAAAAAATTCTATACAATATACAGATTAATCTATATATGCACATCATGGACGCATTTTA
664157_30677280_HHV-7_JI_U43400.1_144861bp_1_2283	TAAAAAAATTCTATACAATATACAGATTAATCTATATATGCACATCATGGACGCATTTTATGGCTTCGAGCTATACACTCTTTTGTTTTATTCATTTCTTTAATACTTTGTATTTTATAG
664157_30677280_HHV-7_JI_U43400.1_144861bp_1_2284	TGGCTTCGAGCTATACACTCTTTTGTTTTATTCATTTCTTTAATACTTTGTATTTTATAGAAACTAAATAAGAAGTTTTAATGAAATGCATTTATTAACCACCCATAACATTTTGTAACT
664157_30677280_HHV-7_JI_U43400.1_144861bp_1_2285	AAACTAAATAAGAAGTTTTAATGAAATGCATTTATTAACCACCCATAACATTTTGTAACTTTTCGGATACAGTATTGTTAAAGGCTTGAGTTATATTTCCACTGTTACACAGTTGAGCAT
664157_30677280_HHV-7_JI_U43400.1_144861bp_1_2286	TTTCGGATACAGTATTGTTAAAGGCTTGAGTTATATTTCCACTGTTACACAGTTGAGCATTTAAAGATTTGTTCGAGATGAGTTTTTTTGTTGTCCGGTCATCTTTTTTTAAGAAAGTCA
664157_30677280_HHV-7_JI_U43400.1_144861bp_1_2287	TTAAAGATTTGTTCGAGATGAGTTTTTTTGTTGTCCGGTCATCTTTTTTTAAGAAAGTCAATAACAGCGTATCAATAGTGAGTAGTCTTCCGTTGTCAAACTCTGTTTTTGCACTGTCGG
664157_30677280_HHV-7_JI_U43400.1_144861bp_1_2288	ATAACAGCGTATCAATAGTGAGTAGTCTTCCGTTGTCAAACTCTGTTTTTGCACTGTCGGTTTTAATTTTAATTGAGCTGGTATGAAAAAAAAACACATTAAGTTTTCTTGTTAGATTCT
664157_30677280_HHV-7_JI_U43400.1_144861bp_1_2289	TTTTAATTTTAATTGAGCTGGTATGAAAAAAAAACACATTAAGTTTTCTTGTTAGATTCTTATCCCCCCCCCCCCCGACAGGAGAGTTTTCAAATTACCAAGGTCCGAAATCTTTAGATG
664157_30677280_HHV-7_JI_U43400.1_144861bp_1_2290	TATCCCCCCCCCCCCCGACAGGAGAGTTTTCAAATTACCAAGGTCCGAAATCTTTAGATGGTGTTAGTAGTACATCGTATTGGAGATCCGTTGTAGATATCTTAGATGGAAAGTCTTGCC
664157_30677280_HHV-7_JI_U43400.1_144861bp_1_2291	GTGTTAGTAGTACATCGTATTGGAGATCCGTTGTAGATATCTTAGATGGAAAGTCTTGCCACATAGCACTTACGATGGCTGGATCGAATTCTCTAGGATGAAGAAGCGAAAATTAGATTA
664157_30677280_HHV-7_JI_U43400.1_144861bp_1_2292	ACATAGCACTTACGATGGCTGGATCGAATTCTCTAGGATGAAGAAGCGAAAATTAGATTATATCTAGTTTTGCTTTCTAAGTTAATAATATTTACAATTTACCTGTTACTAAACGACTGT
664157_30677280_HHV-7_JI_U43400.1_144861bp_1_2293	TATCTAGTTTTGCTTTCTAAGTTAATAATATTTACAATTTACCTGTTACTAAACGACTGTCGGTGCGGATGTGGAAACATGTCTTGTACAGTCTTTTGTCCAATAGTGTCGAATAAGCGT

664157_30677280_HHV-7_JI_U43400.1_144861bp_1_2294	CGGTGCGGATGTGGAAACATGTCTTGTACAGTCTTTTGTCCAATAGTGTCGAATAAGCGTTCTATTTCTAGTAGTGCTATTGGAAAAAGACTGCGAAACACAATTTATTTGCTTATTCTC
664157_30677280_HHV-7_JI_U43400.1_144861bp_1_2295	TCTATTTCTAGTAGTGCTATTGGAAAAAGACTGCGAAACACAATTTATTTGCTTATTCTCTCCAGTTTTTAAATTTCGAGTAATAAAAGACACTTGCTTTTCTTACATTACGGTGGTGAC
664157_30677280_HHV-7_JI_U43400.1_144861bp_1_2296	TCCAGTTTTTAAATTTCGAGTAATAAAAGACACTTGCTTTTCTTACATTACGGTGGTGACGTAGTTTCGTGCAGTTGTATCGTTTTTAGGCGAGCACCAAATTTGTCCATATGTAGACTG
664157_30677280_HHV-7_JI_U43400.1_144861bp_1_2297	GTAGTTTCGTGCAGTTGTATCGTTTTTAGGCGAGCACCAAATTTGTCCATATGTAGACTGGAATCTAAAAAAAAAAATATTGTCAACTCAACCTTTTTGTCATTTTGTTGATTGCATGTA
664157_30677280_HHV-7_JI_U43400.1_144861bp_1_2298	GAATCTAAAAAAAAAAATATTGTCAACTCAACCTTTTTGTCATTTTGTTGATTGCATGTATGTGCGGTTTTCCTCTTTTTTAGTGTGCTTGTTAATATGAGCAGTTTTGTGCGACAGTAC
664157_30677280_HHV-7_JI_U43400.1_144861bp_1_2299	TGTGCGGTTTTCCTCTTTTTTAGTGTGCTTGTTAATATGAGCAGTTTTGTGCGACAGTACTTACACTGTAGTGTTTGTTAAGCACCTTTCGTCCGTGGGTATCTGTCTAATAATGTCTAT
664157_30677280_HHV-7_JI_U43400.1_144861bp_1_2300	TTACACTGTAGTGTTTGTTAAGCACCTTTCGTCCGTGGGTATCTGTCTAATAATGTCTATGTCTCTCCAGGGTTCGATGGTTGTCTCTGTCCAGGGATGTTCTGACCATCGCCACGCGTA
664157_30677280_HHV-7_JI_U43400.1_144861bp_1_2301	GTCTCTCCAGGGTTCGATGGTTGTCTCTGTCCAGGGATGTTCTGACCATCGCCACGCGTATGTCGTGTTTTCTCGACTTTTGTTCTTATCCGGGCAACAAGCGTCATACAATGGTTTTTC
664157_30677280_HHV-7_JI_U43400.1_144861bp_1_2302	TGTCGTGTTTTCTCGACTTTTGTTCTTATCCGGGCAACAAGCGTCATACAATGGTTTTTCTGAAGTCATGTTCACTTTAGGTATGTTAAAATCTTCTTCTTTCCATTGTGAAAATTGCAT
664157_30677280_HHV-7_JI_U43400.1_144861bp_1_2303	TGAAGTCATGTTCACTTTAGGTATGTTAAAATCTTCTTCTTTCCATTGTGAAAATTGCATGCAAGTTTCCCATTCGTAGCCCTTGTTGTCTTTTCGGACGATTGCCAGTGATATTTGTGT
664157_30677280_HHV-7_JI_U43400.1_144861bp_1_2304	GCAAGTTTCCCATTCGTAGCCCTTGTTGTCTTTTCGGACGATTGCCAGTGATATTTGTGTCCGAAAGGCTATTCCGCTGTAGCTACGCATCTTTTTAAAATCTGCGCGTATCTGTAATTC
664157_30677280_HHV-7_JI_U43400.1_144861bp_1_2305	CCGAAAGGCTATTCCGCTGTAGCTACGCATCTTTTTAAAATCTGCGCGTATCTGTAATTCACTAATAAGCGGGTACTTTACATTCAAATTCTTGGAGAAAAAAATAACTATAGATAGTAC

664157_30677280_HHV-7_JI_U43400.1_144861bp_1_2306	ACTAATAAGCGGGTACTTTACATTCAAATTCTTGGAGAAAAAAATAACTATAGATAGTACCTTTTTTTGCTTACCATTGAGCAGACGACTGGGTGTATTGCTCACGTCTTTCCCAACGGT
664157_30677280_HHV-7_JI_U43400.1_144861bp_1_2307	CTTTTTTTGCTTACCATTGAGCAGACGACTGGGTGTATTGCTCACGTCTTTCCCAACGGTGGCGAGCAGGGAACAAAACCAGGCTCGTCAAAATGTTTGCGGTGTTCATTCTTTTCACAT
664157_30677280_HHV-7_JI_U43400.1_144861bp_1_2308	GGCGAGCAGGGAACAAAACCAGGCTCGTCAAAATGTTTGCGGTGTTCATTCTTTTCACATACAACAAACTGTTTTGTTTACTTCCGAATCCTCCATTAAGTAGAGAATAGTGTAAGCCTG
664157_30677280_HHV-7_JI_U43400.1_144861bp_1_2309	ACAACAAACTGTTTTGTTTACTTCCGAATCCTCCATTAAGTAGAGAATAGTGTAAGCCTGTAGTTACTGACAAGACTTCAGACAGCCCTTTACTCATAATTTTTTCTCTTAATCCGCGCA
664157_30677280_HHV-7_JI_U43400.1_144861bp_1_2310	TAGTTACTGACAAGACTTCAGACAGCCCTTTACTCATAATTTTTTCTCTTAATCCGCGCACTCCCAATGAGTTCTCTTTTAGTGGAATGCAGAGTGTTGTTTCTAATAATATAACCCTTA
664157_30677280_HHV-7_JI_U43400.1_144861bp_1_2311	CTCCCAATGAGTTCTCTTTTAGTGGAATGCAGAGTGTTGTTTCTAATAATATAACCCTTAGAAGAATTAAAAGTAAGGTGTGAGAAATTTACTTAAAAAGCTCTTCAAAGTCGTCATATT
664157_30677280_HHV-7_JI_U43400.1_144861bp_1_2312	GAAGAATTAAAAGTAAGGTGTGAGAAATTTACTTAAAAAGCTCTTCAAAGTCGTCATATTGTAGAAATCAGTGTAACCATTTTTACTTACTGTGCTTCGTCTGGCCATTCACATTCGCAG
664157_30677280_HHV-7_JI_U43400.1_144861bp_1_2313	GTAGAAATCAGTGTAACCATTTTTACTTACTGTGCTTCGTCTGGCCATTCACATTCGCAGGAAAACGATGTACATCTTGTGTATTCTAACAAGCTTTGTCTTTCCTCGGAACTTAGACCA
664157_30677280_HHV-7_JI_U43400.1_144861bp_1_2314	GAAAACGATGTACATCTTGTGTATTCTAACAAGCTTTGTCTTTCCTCGGAACTTAGACCAATATATCTAGTAGAGCTTGCGTTGTGCATGTTTTCATTTATTAAGTTTTCGATTATATCT
664157_30677280_HHV-7_JI_U43400.1_144861bp_1_2315	ATATATCTAGTAGAGCTTGCGTTGTGCATGTTTTCATTTATTAAGTTTTCGATTATATCTTCTAGAGTTATTGCCGTGCGTGCTTTTTCAAGTGGGACCGTATTGGTACATATCTTTATG
664157_30677280_HHV-7_JI_U43400.1_144861bp_1_2316	TCTAGAGTTATTGCCGTGCGTGCTTTTTCAAGTGGGACCGTATTGGTACATATCTTTATGAGTGATGCGAAAATTATATAATGCAGTTGCACCATTATCTGCGAAGAGATTTTTTTTGCA
664157_30677280_HHV-7_JI_U43400.1_144861bp_1_2317	AGTGATGCGAAAATTATATAATGCAGTTGCACCATTATCTGCGAAGAGATTTTTTTTGCAGCGTCTTTATGTGCAGGCTTATTCCCCCCCGTTTCGTATTTCAAATCCTAAATAACCCCC

664157_30677280_HHV-7_JI_U43400.1_144861bp_1_2318	GCGTCTTTATGTGCAGGCTTATTCCCCCCCGTTTCGTATTTCAAATCCTAAATAACCCCCGGGGGGTAAAAAAAGGGGGGGAGCTAACCCTAACCCTAACCCTAACCCTAGCTCTAAGCC
664157_30677280_HHV-7_JI_U43400.1_144861bp_1_2320	TAACCCCAGCCCTAACCCTAACCCTAGCTCTAAGCCTAACCCCAACCCTAACCCTAGCTCTCACTGTCACCCTAACACTAGCTCCAAGTCATCTGTTCTAGATCCTATCCATATCTGCCC
664157_30677280_HHV-7_JI_U43400.1_144861bp_1_2321	TCACTGTCACCCTAACACTAGCTCCAAGTCATCTGTTCTAGATCCTATCCATATCTGCCCTGACTCCTGGTTCCTTACCGCTCCGAGCCCCACCCTCCGTCCCGCCCTCCTCCTGTTCTC
664157_30677280_HHV-7_JI_U43400.1_144861bp_1_2322	TGACTCCTGGTTCCTTACCGCTCCGAGCCCCACCCTCCGTCCCGCCCTCCTCCTGTTCTCCATGCCCTGCCTTCTCAACCCTTCCTCTTCCACGCCCACATTGCCTCTGCACTCCGCGCT
664157_30677280_HHV-7_JI_U43400.1_144861bp_1_2323	CATGCCCTGCCTTCTCAACCCTTCCTCTTCCACGCCCACATTGCCTCTGCACTCCGCGCTCTCTTGGCTGTGCGCCCTGCCTTTCCGTGACCTACTGGGAGCGCCGCCAAATCTGTTTTG
664157_30677280_HHV-7_JI_U43400.1_144861bp_1_2324	CTCTTGGCTGTGCGCCCTGCCTTTCCGTGACCTACTGGGAGCGCCGCCAAATCTGTTTTGCCCCGCCCCTGCGCGCGCGGGAACTGTCGGCGCCGCGCTGCTGCTAGCCCGCCTTCCAGA
664157_30677280_HHV-7_JI_U43400.1_144861bp_1_2325	CCCCGCCCCTGCGCGCGCGGGAACTGTCGGCGCCGCGCTGCTGCTAGCCCGCCTTCCAGAGCTCCCTCCCTCCGTCTGCCTCCTCACCCTTGCCACTCACCCTTCCATCTCTTCTATCAC
664157_30677280_HHV-7_JI_U43400.1_144861bp_1_2326	GCTCCCTCCCTCCGTCTGCCTCCTCACCCTTGCCACTCACCCTTCCATCTCTTCTATCACAGACTCTGTGTTACACCACCTATGACTGCTGCAACCACAGAACATTTTGCTCTCCGCGCG
664157_30677280_HHV-7_JI_U43400.1_144861bp_1_2327	AGACTCTGTGTTACACCACCTATGACTGCTGCAACCACAGAACATTTTGCTCTCCGCGCGGCACTCAATCGTTACTGGTGGCTGCTTCTGGGACGACACAAGCTCAGTTTGGTATGCAAC
664157_30677280_HHV-7_JI_U43400.1_144861bp_1_2328	GCACTCAATCGTTACTGGTGGCTGCTTCTGGGACGACACAAGCTCAGTTTGGTATGCAACTACGTCACAGCTCATCGCCAACAGTTACTGCCGCTGCCGTGGCCCGAACAGGAATTTCTC
664157_30677280_HHV-7_JI_U43400.1_144861bp_1_2329	TACGTCACAGCTCATCGCCAACAGTTACTGCCGCTGCCGTGGCCCGAACAGGAATTTCTCCAACTTGACCCGGCCCCCTACTCCAATCTCCGCAACCGTGTCGCTCACCATCTCCATCGC
664157_30677280_HHV-7_JI_U43400.1_144861bp_1_2330	CAACTTGACCCGGCCCCCTACTCCAATCTCCGCAACCGTGTCGCTCACCATCTCCATCGCGGCTGGCCAGCGGCACACAACACATGTAAGCTACCGTACATCTCTTTCACAAACCCAGGG

664157_30677280_HHV-7_JI_U43400.1_144861bp_1_2331	GGCTGGCCAGCGGCACACAACACATGTAAGCTACCGTACATCTCTTTCACAAACCCAGGGCTCACATAGAGACAAGCACAAGCTCGCGCAATGACATTAAAACCTCCCATCATTGTCCTT
664157_30677280_HHV-7_JI_U43400.1_144861bp_1_2332	CTCACATAGAGACAAGCACAAGCTCGCGCAATGACATTAAAACCTCCCATCATTGTCCTTTCCTGTCGCTTTGCCGATAACGTCTTTGCTCTATCGCAGGTTTCGACCCCCGTCCTTACT
664157_30677280_HHV-7_JI_U43400.1_144861bp_1_2333	TCCTGTCGCTTTGCCGATAACGTCTTTGCTCTATCGCAGGTTTCGACCCCCGTCCTTACTTCCCCAATGCTAAAGTCAAGCTGCTTCCGCTCGGCTCCATCACCCTTACCAGATCATTCT
664157_30677280_HHV-7_JI_U43400.1_144861bp_1_2334	TCCCCAATGCTAAAGTCAAGCTGCTTCCGCTCGGCTCCATCACCCTTACCAGATCATTCTCCAGTGACGAGCCTCATCCTATTGGTGATGATGTGCATCACAGTCATGACCGGGGTGACT
664157_30677280_HHV-7_JI_U43400.1_144861bp_1_2335	CCAGTGACGAGCCTCATCCTATTGGTGATGATGTGCATCACAGTCATGACCGGGGTGACTACCATACTGTTATCTGCAGCTGGCTCACAGGAACCTCCCCGATCCTAGTGCTGCTTCAAG
664157_30677280_HHV-7_JI_U43400.1_144861bp_1_2336	ACCATACTGTTATCTGCAGCTGGCTCACAGGAACCTCCCCGATCCTAGTGCTGCTTCAAGGACCGGACGGCAGCATCTATTGCCACGACGTGTACCGCGGCCGATTGTATCTCGTGGCCC
664157_30677280_HHV-7_JI_U43400.1_144861bp_1_2337	GACCGGACGGCAGCATCTATTGCCACGACGTGTACCGCGGCCGATTGTATCTCGTGGCCCACTCTGTATCGTTGTTCGCCAGGCTAGGCCTTCGCCACTGCGAACCTTTATATGCGGCAC
664157_30677280_HHV-7_JI_U43400.1_144861bp_1_2338	ACTCTGTATCGTTGTTCGCCAGGCTAGGCCTTCGCCACTGCGAACCTTTATATGCGGCACCCAGATGGAAGCACGTTCCTCTGCCCAACATGTGGGTGGCGAGCCCGCCAGCGTCCGCCA
664157_30677280_HHV-7_JI_U43400.1_144861bp_1_2339	CCAGATGGAAGCACGTTCCTCTGCCCAACATGTGGGTGGCGAGCCCGCCAGCGTCCGCCACCCTCACGCAAACACTCGCCGTGAGTGCCACGCACGGTCTGGACGCGTTATACTCGCTGC
664157_30677280_HHV-7_JI_U43400.1_144861bp_1_2340	CCCTCACGCAAACACTCGCCGTGAGTGCCACGCACGGTCTGGACGCGTTATACTCGCTGCTAAAAATCCACAGAGGAACTCCGTGTTCGCTAATCCACCCCGTGAACGGCTACGTCCTGG
664157_30677280_HHV-7_JI_U43400.1_144861bp_1_2341	TAAAAATCCACAGAGGAACTCCGTGTTCGCTAATCCACCCCGTGAACGGCTACGTCCTGGACATGATACTGACGGGCCGCTCATTCCAAGAAGCACCCTGCCAAAACACTCGCACGTCCG
664157_30677280_HHV-7_JI_U43400.1_144861bp_1_2342	ACATGATACTGACGGGCCGCTCATTCCAAGAAGCACCCTGCCAAAACACTCGCACGTCCGTTAAAACAACGCCACATGTAATGGACGCAGTCTGCGGTGGCCGCGGGTCATGGCTGTCCA

664157_30677280_HHV-7_JI_U43400.1_144861bp_1_2343	TTAAAACAACGCCACATGTAATGGACGCAGTCTGCGGTGGCCGCGGGTCATGGCTGTCCATCGGCTACCTAGTAAAGATGCCGCACATTCACCTGGCGGTGACCCGAACATGTCTGGTCA
664157_30677280_HHV-7_JI_U43400.1_144861bp_1_2344	TCGGCTACCTAGTAAAGATGCCGCACATTCACCTGGCGGTGACCCGAACATGTCTGGTCACCGCCATAGATGTCCGACAAAACTTTCTGTGGCGCGTGGCGGACGACGCGCTGCTATTCC
664157_30677280_HHV-7_JI_U43400.1_144861bp_1_2345	CCGCCATAGATGTCCGACAAAACTTTCTGTGGCGCGTGGCGGACGACGCGCTGCTATTCCTGGTCACCGGTAGTCTTTTACTACTGTCGCGGCCGACCGCAGACTTGACGTCTTGGTCAT
664157_30677280_HHV-7_JI_U43400.1_144861bp_1_2346	TGGTCACCGGTAGTCTTTTACTACTGTCGCGGCCGACCGCAGACTTGACGTCTTGGTCATGTTTACAGCAAGAACCTGTGTGGAGGAACTGTCTAGATACGCGCGGAGAACAGGATGAGA
664157_30677280_HHV-7_JI_U43400.1_144861bp_1_2347	GTTTACAGCAAGAACCTGTGTGGAGGAACTGTCTAGATACGCGCGGAGAACAGGATGAGACAGAAGACCAAGAGATGAAACAAAGCACAAGCAAAAAGCAAAATGAGAATAAAAAACTCA
664157_30677280_HHV-7_JI_U43400.1_144861bp_1_2348	CAGAAGACCAAGAGATGAAACAAAGCACAAGCAAAAAGCAAAATGAGAATAAAAAACTCAACACCTCAAAAAAACACACCCGCGTATCGTCGGCAATTCCGACCTTTCCCCTCAGTCTCC
664157_30677280_HHV-7_JI_U43400.1_144861bp_1_2349	ACACCTCAAAAAAACACACCCGCGTATCGTCGGCAATTCCGACCTTTCCCCTCAGTCTCCGAGAAACGCCGCCAGAAGCCAGGAGCCCAGCCGTCCTCGCCGCCGCCACCCAGTCTCACA
664157_30677280_HHV-7_JI_U43400.1_144861bp_1_2350	GAGAAACGCCGCCAGAAGCCAGGAGCCCAGCCGTCCTCGCCGCCGCCACCCAGTCTCACAAAACTCGAGCGATCTCGACGCATAATGCCACGACAACAATAAGAATACCGCGCCTTCCCA
664157_30677280_HHV-7_JI_U43400.1_144861bp_1_2351	AAACTCGAGCGATCTCGACGCATAATGCCACGACAACAATAAGAATACCGCGCCTTCCCAGTTACCTGCTGGAAGCGCGTCTCTTGTCCGTGACAGCTATCCTGAAAGACACAAAGAAAA
664157_30677280_HHV-7_JI_U43400.1_144861bp_1_2352	GTTACCTGCTGGAAGCGCGTCTCTTGTCCGTGACAGCTATCCTGAAAGACACAAAGAAAAAAAAAACCCAGCCTCAGGCGTAGCAGCTGCGACGTCAGCGCGGTGTCTGAAAGCTCGCCA
664157_30677280_HHV-7_JI_U43400.1_144861bp_1_2353	AAAAAACCCAGCCTCAGGCGTAGCAGCTGCGACGTCAGCGCGGTGTCTGAAAGCTCGCCAAGGTCTCGCGTAAAAGAACAGATGTGAACTTCAGATGTACCAACCAAATAATACGGGTTC
664157_30677280_HHV-7_JI_U43400.1_144861bp_1_2354	AGGTCTCGCGTAAAAGAACAGATGTGAACTTCAGATGTACCAACCAAATAATACGGGTTCCGCTATAAAAAGTGCACCTCTATTCCCGTTCTTATCCCCGTTCTAACTCTTCCTTGTATC

664157_30677280_HHV-7_JI_U43400.1_144861bp_1_2355	CGCTATAAAAAGTGCACCTCTATTCCCGTTCTTATCCCCGTTCTAACTCTTCCTTGTATCATACCTTGCATGTTAACCGGATCCCGTGGATCTTACACACATACACACACACACAAACTT
664157_30677280_HHV-7_JI_U43400.1_144861bp_1_2356	ATACCTTGCATGTTAACCGGATCCCGTGGATCTTACACACATACACACACACACAAACTTGGTGAGGTAAACACAGAGATCTCACTAACTCATAATCCCCTACACGCTTACCACCACCTA
664157_30677280_HHV-7_JI_U43400.1_144861bp_1_2357	GGTGAGGTAAACACAGAGATCTCACTAACTCATAATCCCCTACACGCTTACCACCACCTAAAAATGGTTACAACCAAAACTGGCAATAGTCTATCTTCTTTTTCTTTCCATTCACAGCCA
664157_30677280_HHV-7_JI_U43400.1_144861bp_1_2358	AAAATGGTTACAACCAAAACTGGCAATAGTCTATCTTCTTTTTCTTTCCATTCACAGCCAATGTGCAGTACTCGTGGGTCCACAACAACGAAAGAGACTGTAGAGACAGTTCCTTTAAGT
664157_30677280_HHV-7_JI_U43400.1_144861bp_1_2359	ATGTGCAGTACTCGTGGGTCCACAACAACGAAAGAGACTGTAGAGACAGTTCCTTTAAGTAGACCTTAGAGACACACCAAATACAACCACAACCAAAAAAAAAACAGAAAACAACACAAA
664157_30677280_HHV-7_JI_U43400.1_144861bp_1_2360	AGACCTTAGAGACACACCAAATACAACCACAACCAAAAAAAAAACAGAAAACAACACAAAGCCAATGAGTGCAGAAATGCTCCGCGCTGTTCAGCTCCAGCCAAGACGCCGGGGACATTC
664157_30677280_HHV-7_JI_U43400.1_144861bp_1_2361	GCCAATGAGTGCAGAAATGCTCCGCGCTGTTCAGCTCCAGCCAAGACGCCGGGGACATTCCTCATCTCCCACTTCCCCTCCACTCGAAGGAGAGCCCAGTCCCAAGAGACTCCAATCGAG
664157_30677280_HHV-7_JI_U43400.1_144861bp_1_2362	CTCATCTCCCACTTCCCCTCCACTCGAAGGAGAGCCCAGTCCCAAGAGACTCCAATCGAGCGACAGTCACCAAGGGCGTAGAGGCAGACCTAAACCCAGAGCTAAAACATGGAGCGAAGC
664157_30677280_HHV-7_JI_U43400.1_144861bp_1_2363	CGACAGTCACCAAGGGCGTAGAGGCAGACCTAAACCCAGAGCTAAAACATGGAGCGAAGCTTTATCCCACCGGTCCTTCCTCAACATTTACGCGTGGCTGTCTTTGAGTCGAGGGTCTCC
664157_30677280_HHV-7_JI_U43400.1_144861bp_1_2364	TTTATCCCACCGGTCCTTCCTCAACATTTACGCGTGGCTGTCTTTGAGTCGAGGGTCTCCGCGAAAAGTGTACGGATATGCCTTCAGGCACAGAGGAGAACTCGTAGCATTGCCATGGCC
664157_30677280_HHV-7_JI_U43400.1_144861bp_1_2365	GCGAAAAGTGTACGGATATGCCTTCAGGCACAGAGGAGAACTCGTAGCATTGCCATGGCCGCCTAACTGGAGCCTGGAACTTCACCACGATCCCTATCGAGACGCCAGAGCACAAACCGT
664157_30677280_HHV-7_JI_U43400.1_144861bp_1_2366	GCCTAACTGGAGCCTGGAACTTCACCACGATCCCTATCGAGACGCCAGAGCACAAACCGTTTGGAGTCACCGCTGGGGATGGCCTGCAACACACGTGACAGCTCGCACGGTGCGGGACTG

664157_30677280_HHV-7_JI_U43400.1_144861bp_1_2367	TTGGAGTCACCGCTGGGGATGGCCTGCAACACACGTGACAGCTCGCACGGTGCGGGACTGCGGTGAGTGTAAGCAGTGTGACACATTGTTATCGCAATTGTCTTACCCGATTAACTTTTT
664157_30677280_HHV-7_JI_U43400.1_144861bp_1_2368	CGGTGAGTGTAAGCAGTGTGACACATTGTTATCGCAATTGTCTTACCCGATTAACTTTTTATTAATGTATTAAGCACTCTTTCTTCACGTGTGACTGTTGTGTTTTTTGTTGTTATCTAC
664157_30677280_HHV-7_JI_U43400.1_144861bp_1_2369	ATTAATGTATTAAGCACTCTTTCTTCACGTGTGACTGTTGTGTTTTTTGTTGTTATCTACATCCCGGCAGCCCTCGACACGCATATGTACGTGTGCTGCGGACGCGGAGAAAAGTTGCAG
664157_30677280_HHV-7_JI_U43400.1_144861bp_1_2370	ATCCCGGCAGCCCTCGACACGCATATGTACGTGTGCTGCGGACGCGGAGAAAAGTTGCAGCCCGTCGGATACGTACGCAACAGAGCCGCGCCTTCAGACCTGAACTCGTTACGCGTCCTC
664157_30677280_HHV-7_JI_U43400.1_144861bp_1_2371	CCCGTCGGATACGTACGCAACAGAGCCGCGCCTTCAGACCTGAACTCGTTACGCGTCCTCCTCATAGCCAGGGACGGAGCAATGTATGTGCATCACATGAGAACGGCGCGACTGTGCCGC
664157_30677280_HHV-7_JI_U43400.1_144861bp_1_2372	CTCATAGCCAGGGACGGAGCAATGTATGTGCATCACATGAGAACGGCGCGACTGTGCCGCCTGGCCAGCAGTGTGACCGAATTCGCGCGACGAGGGCTGCAGCGAGAATCCGAGGTTTAT
664157_30677280_HHV-7_JI_U43400.1_144861bp_1_2373	CTGGCCAGCAGTGTGACCGAATTCGCGCGACGAGGGCTGCAGCGAGAATCCGAGGTTTATGAAGATGATGTTTCCTTGCCAGACCGTCGAGTAGGTTCGGCAACGGCCATTCACCTGTTT
664157_30677280_HHV-7_JI_U43400.1_144861bp_1_2374	GAAGATGATGTTTCCTTGCCAGACCGTCGAGTAGGTTCGGCAACGGCCATTCACCTGTTTGACGTAATTACCCAGGCAGCCGATGTCCACGACCTACTCACCGTGGCCGGACTGTGTCAG
664157_30677280_HHV-7_JI_U43400.1_144861bp_1_2375	GACGTAATTACCCAGGCAGCCGATGTCCACGACCTACTCACCGTGGCCGGACTGTGTCAGACTCACACCGGCGTCAGCTGCCAACTGTGGTATACAGACCACGATCCCCACACCGTCGCT
664157_30677280_HHV-7_JI_U43400.1_144861bp_1_2376	ACTCACACCGGCGTCAGCTGCCAACTGTGGTATACAGACCACGATCCCCACACCGTCGCTGGGGCGGCACGCTTCACACTGACGGTCGCACGGCAGCAGTATCGATTGTGGCCAAACGCA
664157_30677280_HHV-7_JI_U43400.1_144861bp_1_2377	GGGGCGGCACGCTTCACACTGACGGTCGCACGGCAGCAGTATCGATTGTGGCCAAACGCACGACGCAAACTGCTGCAGCACCTACATCCGGACCACCCACTTGGGCTGTGGCTGTTGTGT
664157_30677280_HHV-7_JI_U43400.1_144861bp_1_2378	CGACGCAAACTGCTGCAGCACCTACATCCGGACCACCCACTTGGGCTGTGGCTGTTGTGTGCCGTGCTCACGTACGATGCAAAAGAGACGAATCGCGCAGTGCCACCCGTAACGCCAGGG

664157_30677280_HHV-7_JI_U43400.1_144861bp_1_2379	GCCGTGCTCACGTACGATGCAAAAGAGACGAATCGCGCAGTGCCACCCGTAACGCCAGGGGCCGAAACCGTGTGGGTGATAGTTACTGGCAGGGGTGCCATTCTAGGATTCTGGCCAGAG
664157_30677280_HHV-7_JI_U43400.1_144861bp_1_2380	GCCGAAACCGTGTGGGTGATAGTTACTGGCAGGGGTGCCATTCTAGGATTCTGGCCAGAGAGCGCCAAAATGTGCAGATTGGCCTCGTCTATGAAAGGACTCTGGAAAAACGGAGCGCGG
664157_30677280_HHV-7_JI_U43400.1_144861bp_1_2381	AGCGCCAAAATGTGCAGATTGGCCTCGTCTATGAAAGGACTCTGGAAAAACGGAGCGCGGGCGCTAAAAGGTCACTGGACATACGCAGCACCCGGCCGGCATAGAGCGGGAGAGGCCTGG
664157_30677280_HHV-7_JI_U43400.1_144861bp_1_2382	GCGCTAAAAGGTCACTGGACATACGCAGCACCCGGCCGGCATAGAGCGGGAGAGGCCTGGCCTTTGTGTGCACACTACCAATCTCCTAGATAGAACAAAATTAAAAAGATTAAAAAAAAA
664157_30677280_HHV-7_JI_U43400.1_144861bp_1_2383	CCTTTGTGTGCACACTACCAATCTCCTAGATAGAACAAAATTAAAAAGATTAAAAAAAAAAGAAAAAAAGTACAAGAGTGTTATCGCGAAACAGCGTGTCAAAAAAAAAACAATCCACAT
664157_30677280_HHV-7_JI_U43400.1_144861bp_1_2384	AGAAAAAAAGTACAAGAGTGTTATCGCGAAACAGCGTGTCAAAAAAAAAACAATCCACATACTCTAGAACAAACTGTACCCAAAAATAAGTCCGTGTGCAAAACTGGGAAAAAAAAATCA
664157_30677280_HHV-7_JI_U43400.1_144861bp_1_2385	ACTCTAGAACAAACTGTACCCAAAAATAAGTCCGTGTGCAAAACTGGGAAAAAAAAATCACCTTCCTCGTTGCCACTAGAGGGAGTACCGAAAGTGTAGGCAAGAAGGCCACGCTGTAAA
664157_30677280_HHV-7_JI_U43400.1_144861bp_1_2386	CCTTCCTCGTTGCCACTAGAGGGAGTACCGAAAGTGTAGGCAAGAAGGCCACGCTGTAAATGACTGTCAGCGTTTGGCGCTGAAAACATTGCTGTTCTTGCTGGCTCAAGCACAATCACG
664157_30677280_HHV-7_JI_U43400.1_144861bp_1_2387	TGACTGTCAGCGTTTGGCGCTGAAAACATTGCTGTTCTTGCTGGCTCAAGCACAATCACGTGATTAAGATTCCTTTCGTTTTCAAAGTGTGCCCGGGAGGCAGACATGCCCTTTCTCGTG
664157_30677280_HHV-7_JI_U43400.1_144861bp_1_2388	TGATTAAGATTCCTTTCGTTTTCAAAGTGTGCCCGGGAGGCAGACATGCCCTTTCTCGTGAGACATTATGAGATTTGCCTGCCAGAGAACCACGTGACTTGGACTTACTTTCGTTTTCTA
664157_30677280_HHV-7_JI_U43400.1_144861bp_1_2389	AGACATTATGAGATTTGCCTGCCAGAGAACCACGTGACTTGGACTTACTTTCGTTTTCTAAACGTGCCCTCTAGGCATGAATGCTCTTTAGCGTTAGCCATGAGGCTAGCGTGATCCTGT
664157_30677280_HHV-7_JI_U43400.1_144861bp_1_2390	AACGTGCCCTCTAGGCATGAATGCTCTTTAGCGTTAGCCATGAGGCTAGCGTGATCCTGTATAGTACATAAGTTTCTAAGAATATGTTTTTAACAATAATCATGTCCCAAAAAGTCGCGA

664157_30677280_HHV-7_JI_U43400.1_144861bp_1_2391	ATAGTACATAAGTTTCTAAGAATATGTTTTTAACAATAATCATGTCCCAAAAAGTCGCGAGTGACTAAAATTCTCTGTAAATGAAGGCAAATTAAACAGGATACAGACAGTTGTGGCAGT
664157_30677280_HHV-7_JI_U43400.1_144861bp_1_2392	GTGACTAAAATTCTCTGTAAATGAAGGCAAATTAAACAGGATACAGACAGTTGTGGCAGTGGTCCGTTTCGTCTTTCTGTGTTTTCCTTACGCGGCTGACGAGGTAAAGTGTCTCAGTCC
664157_30677280_HHV-7_JI_U43400.1_144861bp_1_2393	GGTCCGTTTCGTCTTTCTGTGTTTTCCTTACGCGGCTGACGAGGTAAAGTGTCTCAGTCCATATTGTTGTCTGTGCCACCGTAGTTAGCGGTGGCATACTAAAAACTCCGATAGATGCAG
664157_30677280_HHV-7_JI_U43400.1_144861bp_1_2394	ATATTGTTGTCTGTGCCACCGTAGTTAGCGGTGGCATACTAAAAACTCCGATAGATGCAGAACAATAACACCGAAAACCACGCTGTGGAACCAGACCACACTTTATAAACAAAACGGCCT
664157_30677280_HHV-7_JI_U43400.1_144861bp_1_2395	AACAATAACACCGAAAACCACGCTGTGGAACCAGACCACACTTTATAAACAAAACGGCCTTATCACCTGGAAAAAAAACTAAAAATAAGGCAATGATACACCTGACTTTCCATTGGAAAC
664157_30677280_HHV-7_JI_U43400.1_144861bp_1_2396	TATCACCTGGAAAAAAAACTAAAAATAAGGCAATGATACACCTGACTTTCCATTGGAAACCTGCCGTAACCCTGACCACAAATCCCATGCTAAATCCCCTGAAACACTGCCAAACGTCGC
664157_30677280_HHV-7_JI_U43400.1_144861bp_1_2397	CTGCCGTAACCCTGACCACAAATCCCATGCTAAATCCCCTGAAACACTGCCAAACGTCGCTACAAGGTTTTTCCGGGATCGAGCCGCAGCAAGCTTAAACTGAGGTCACACACGACTTTA
664157_30677280_HHV-7_JI_U43400.1_144861bp_1_2398	TACAAGGTTTTTCCGGGATCGAGCCGCAGCAAGCTTAAACTGAGGTCACACACGACTTTAATTACGGCAACGCACAGCTGTAAGCTGCAGGAAAGATACGATCGTAAGCAAATGTAGTCC
664157_30677280_HHV-7_JI_U43400.1_144861bp_1_2399	ATTACGGCAACGCACAGCTGTAAGCTGCAGGAAAGATACGATCGTAAGCAAATGTAGTCCTACAATCAAGCGAGGTTGTAGACGTTACCTACAATGAACTACACCTCTAAGCATAACCTG
664157_30677280_HHV-7_JI_U43400.1_144861bp_1_2400	TACAATCAAGCGAGGTTGTAGACGTTACCTACAATGAACTACACCTCTAAGCATAACCTGTCGGGCACAGTGAGACACGCAGCCGTAAATTCAAAACTCAACCCAAACCGAAGTCTAAGT
664157_30677280_HHV-7_JI_U43400.1_144861bp_1_2401	TCGGGCACAGTGAGACACGCAGCCGTAAATTCAAAACTCAACCCAAACCGAAGTCTAAGTCTCACCCTAATCGTAACAGTAACCCTACAACTCTAATCCTAGTCCGTAACCGTAACCCCA
664157_30677280_HHV-7_JI_U43400.1_144861bp_1_2402	CTCACCCTAATCGTAACAGTAACCCTACAACTCTAATCCTAGTCCGTAACCGTAACCCCAATCCTAGCCCTTAGCCCTAACCCTAGCCCTAACCCTAGCTCTAACCTTAGCTCTAACTCT

664157_30677280_HHV-7_JI_U43400.1_144861bp_1_2403	ATCCTAGCCCTTAGCCCTAACCCTAGCCCTAACCCTAGCTCTAACCTTAGCTCTAACTCTGACCCTAGGCCTAACCCTAAGCCTAACCCTAACCGTAGCTCTAAGTTTAACCCTAACCCT
664157_30677280_HHV-7_JI_U43400.1_144861bp_1_2404	GACCCTAGGCCTAACCCTAAGCCTAACCCTAACCGTAGCTCTAAGTTTAACCCTAACCCTAACCCTAACCATGACCCTGACCCTAACCCTAGGCTGCGGCCCTAACCCTAGCCCTAACCC
664157_30677280_HHV-7_JI_U43400.1_144861bp_1_2405	AACCCTAACCATGACCCTGACCCTAACCCTAGGCTGCGGCCCTAACCCTAGCCCTAACCCTAACCCTAATCCTAATCCTAGCCCTAACCCTAGGGCTGCGGCCCTAACCCTAGCCCTAAC
664157_30677280_HHV-7_JI_U43400.1_144861bp_1_2406	TAACCCTAATCCTAATCCTAGCCCTAACCCTAGGGCTGCGGCCCTAACCCTAGCCCTAACCCTAACCCTAACCCTAGGGCTGCGGCCCTAACCCTAACCCTAGGGCTGCGGCCCGAACCC
664157_30677280_HHV-7_JI_U43400.1_144861bp_1_2407	CCTAACCCTAACCCTAGGGCTGCGGCCCTAACCCTAACCCTAGGGCTGCGGCCCGAACCCTAACCCTAACCCTAACCCTAACCCTAGGGCTGCGGCCCTAACCCTAACCCTAGGGCTGCG
664157_30677280_HHV-7_JI_U43400.1_144861bp_1_2408	TAACCCTAACCCTAACCCTAACCCTAGGGCTGCGGCCCTAACCCTAACCCTAGGGCTGCGGCCCTAACCCTAACCCTAGGGCTGCGGCCCGAACCCTAACCCTAACCCTAACCCTAGGGC
664157_30677280_HHV-7_JI_U43400.1_144861bp_1_2409	GCCCTAACCCTAACCCTAGGGCTGCGGCCCGAACCCTAACCCTAACCCTAACCCTAGGGCTGCGGCCCTAACCCTAACCCTAGGGCTGCGGCCCTAACCCTAACCCTAACTCTAGGGCTG
664157_30677280_HHV-7_JI_U43400.1_144861bp_1_2410	TGCGGCCCTAACCCTAACCCTAGGGCTGCGGCCCTAACCCTAACCCTAACTCTAGGGCTGCGGCCCTAACCCTAACCCTAACCCTAACCCTAGGGCTGCGGCCCGAACCCTAGCCCTAAC
664157_30677280_HHV-7_JI_U43400.1_144861bp_1_2414	CCCACTGGCAGCCAATGTCTTGTAATGCCTTCAAGGCACTTTTTCTGCGAGCCGCGCGCAGCACTCAGTGAAAAACA
