# Supplementary material for: Integration of Viral Genome to Human Genomic DNA in Nails of Patients with Chronic Hepatitis B Virus Infection
Source: JMA J. 2023 Sep 29;6(4):426–36. doi: 10.31662/jmaj.2023-0082 (PMC10628332; doi:10.31662/jmaj.2023-0082)
Supplement: Supplementary Table 3 [file 2433-3298-6-4-426-s006.pdf]

Supplementary Table 3. Positive control for capture-based next-generation sequencing

ID	Source	Level of HBV DNA (Log copies/mL)	Level of HHV-7 DNA (Log copies/mL)	Concentration of extracted tissue DNA (ng/μL)*
Ig18206	Liver tissue	8.8	undetectable	114.2
Ig18807	Liver tissue	9	3	38.7
Ig18207	Peripheral whole blood cells	>9.0	4.3	88.7

* NanoDrop system
