# Supplementary material for: Integration of Viral Genome to Human Genomic DNA in Nails of Patients with Chronic Hepatitis B Virus Infection
Source: JMA J. 2023 Sep 29;6(4):426–36. doi: 10.31662/jmaj.2023-0082 (PMC10628332; doi:10.31662/jmaj.2023-0082)
Supplement: Supplementary Table 4 [file 2433-3298-6-4-426-s007.pdf]

Supplementary Table 4. Negative control for capture-based next-generation sequencing

ID	Source	Level of HBV DNA (Log copies/mL)	Level of HHV-7 DNA (Log copies/mL)	Concentration of extracted tissue DNA (ng/µL)*
Ig18208	Nails	undetectable	undetectable	17.4
Ig18808	Liver tissue	undetectable	undetectable	67.8

* NanoDrop system
