# Supplementary material for: Integration of Viral Genome to Human Genomic DNA in Nails of Patients with Chronic Hepatitis B Virus Infection
Source: JMA J. 2023 Sep 29;6(4):426–36. doi: 10.31662/jmaj.2023-0082 (PMC10628332; doi:10.31662/jmaj.2023-0082)
Supplement: Supplementary Table 5 [file 2433-3298-6-4-426-s008.pdf]

Supplementary Table 5. Summary of paired-end reads in positive and negative controls

Positive/Negative control	ID	Yield (M bases)	Number of reads	≧ Q30 bases,%	Quality score mean	Number of trimmed reads	Read length mean (Trimmed reads)
Positive control	Ig18206	3,455	34,209,892	97	38.52	33,321,064	99.66
Positive control	Ig18807	2,480	16,531,814	93.74	35.92	16,281,874	132.43
Positive control	Ig18207	1,575	15,592,540	97.31	38.6	15,289,284	99.65
Negative control	Ig18208	795	7,875,900	93.69	37.76	7,097,968	97.92
Negative control	Ig18808	335	2,232,788	92.53	35.65	2,135,244	130.39
