# Supplementary material for: Integration of Viral Genome to Human Genomic DNA in Nails of Patients with Chronic Hepatitis B Virus Infection
Source: JMA J. 2023 Sep 29;6(4):426–36. doi: 10.31662/jmaj.2023-0082 (PMC10628332; doi:10.31662/jmaj.2023-0082)
Supplement: Supplementary Table 6 [file 2433-3298-6-4-426-s009.pdf]

Supplementary Table 6. Summary of mapped and unmapped reads in the positive and negative controls

ID	Processed reads (trimmed paired reads)	Number of mapped reads	Mapped reads/processed reads, %	Number of duplicated reads	Duplicated reads/processed reads, %	Number of unmapped reads	Unmapped reads/processed reads, %
Ig18206	33,321,064	33,092,926	99.32	30,348,509	91.08	228,138	0.68
Ig18807	16,281,874	15,922,256	97.79	12,950,898	79.54	359,618	2.21
Ig18207	15,289,284	15,153,196	99.11	13,586,863	88.87	136,088	0.89
Ig18208	7,097,968	6,941,060	97.79	5,770,454	81.3	156,908	2.21
Ig18808	2,135,244	2,127,662	99.64	530,735	24.86	7,582	0.36
