# Supplementary material for: Integration of Viral Genome to Human Genomic DNA in Nails of Patients with Chronic Hepatitis B Virus Infection
Source: JMA J. 2023 Sep 29;6(4):426–36. doi: 10.31662/jmaj.2023-0082 (PMC10628332; doi:10.31662/jmaj.2023-0082)
Supplement: Supplementary Table 7 [file 2433-3298-6-4-426-s010.pdf]

Supplementary Table 7. Summary of mapped deduplicated reads in the positive and negative controls

Reference	Chromosome	Length (bp)	Positive control			Negative control	
			Ig18206 (Liver)	Ig18807 (Liver)	Ig18207 (Blood)	Ig18208 (Nails)	Ig18808 (Liver)
Host DNA: Human (GRCh38)	1	248,956,422	205,158	170,195	123,120	138,788	139,353
	2	242,193,529	165,178	131,091	74,845	59,998	110,285
	3	198,295,559	159,552	99,757	75,887	52,139	84,658
	4	190,214,555	162,301	109,249	93,151	61,752	84,930
	5	181,538,259	153,871	105,748	74,631	64,745	88,568
	6	170,805,979	143,346	87,525	70,222	47,852	75,111
	7	159,345,973	132,568	99,354	75,860	60,254	78,458
	8	145,138,636	119,708	78,392	60,175	42,929	65,251
	9	138,394,717	109,055	73,321	58,524	38,590	63,156
	10	133,797,422	101,770	94,534	59,241	53,216	74,606
	11	135,086,622	113,357	81,910	58,701	47,305	70,513
	12	133,275,309	103,277	78,713	55,245	42,348	63,940
	13	114,364,328	81,637	46,710	37,181	30,124	42,445
	14	107,043,718	71,423	53,452	34,476	29,848	41,171
	15	101,991,189	80,093	53,127	44,522	28,790	45,529
	16	90,338,345	70,867	73,016	46,734	40,115	59,502
	17	83,257,441	78,188	75,607	41,359	35,531	61,403
	18	80,373,285	79,686	45,916	61,123	49,229	41,019
	19	58,617,616	65,080	64,425	41,467	44,803	50,486
	20	64,444,167	95,893	52,914	48,191	42,642	43,425
	21	46,709,983	84,606	71,633	53,991	55,598	65,329
	22	50,818,468	49,118	44,045	27,116	26,508	34,992
	X	156,040,895	79,498	85,720	41,196	40,849	73,318
	Y	57,227,415	85,906	13,950	84,450	34,880	7,992
	Mitochondria	16,569	18,114	14,183	690	1,417	31,483
Exogenous DNA:	HBV_AB033550.1	3,215	134,951	1,065,952	116,346	274	0
	HHV7JI_HHU43400.1	144,861	216	919	7,889	82	4
Mapped deduplicated reads (mapped reads minus duplicated reads)			2,744,417	2,971,358	1,566,333	1,170,606	1,596,927
