# Supplementary material for: Integration of Viral Genome to Human Genomic DNA in Nails of Patients with Chronic Hepatitis B Virus Infection
Source: JMA J. 2023 Sep 29;6(4):426–36. doi: 10.31662/jmaj.2023-0082 (PMC10628332; doi:10.31662/jmaj.2023-0082)
Supplement: Supplementary Table 8 [file 2433-3298-6-4-426-s011.pdf]

Supplementary Table 8. Ig18206 HBV integration breakpoints

Chrom	Start	End	Insert_Seq Breakpoint	Seqcode	# Junction Reads	Fraction of MQ0 Reads	# Junction Reads (Dedup)	Fraction of MQ0 Reads (Dedup)	Feature	Gene Name	Trasncript Biotype
1	2,965,277	2,965,278	1,805	3prime(HBV)-2965278-3prime(Human)	20	0.00	1	0.00	intron	'AL589702.1	lncRNA
1	5,045,620	5,045,621	1,827	3prime(HBV)-5045621-3prime(Human)	7	1.00	1	1.00	gene	'LINC02782	lncRNA
1	10,205,941	10,205,942	1,826	3prime(HBV)-10205942-5prime(Human)	33	0.00	1	0.00	gene	'KIF1B	protein_coding
1	22,569,199	22,569,200	1,818	3prime(Human)-22569200-5prime(HBV)	20	0.00	1	0.00	intron	'EPHA8	protein_coding
1	23,635,145	23,635,146	1,826	3prime(HBV)-23635146-5prime(Human)	24	0.00	1	0.00	intron	'MDS2	lncRNA
1	23,635,159	23,635,160	848	3prime(Human)-23635160-5prime(HBV)	26	0.00	1	0.00	intron	'MDS2	lncRNA
1	34,261,468	34,261,469	2,390	3prime(Human)-34261469-5prime(HBV)	18	0.00	1	0.00	gene	'AC115286.1	processed_pseudogene
1	35,464,594	35,464,595	1,826	3prime(HBV)-35464595-5prime(Human)	1	1.00	1	1.00	intron	'KIAA0319L	protein_coding
1	42,550,587	42,550,588	2,897	3prime(HBV)-42550588-3prime(Human)	31	0.00	1	0.00	intron	'CCDC30	protein_coding
1	43,118,434	43,118,435	1,977	5prime(Human)-43118435-5prime(HBV)	2	1.00	1	1.00	gene	'AL161637.1	processed_pseudogene
1	43,818,862	43,818,863	1,823	5prime(Human)-43818863-5prime(HBV)	83	0.00	2	0.00	intron	'ST3GAL3	protein_coding
1	43,818,862	43,818,863	1,824	5prime(Human)-43818863-5prime(HBV)	1	0.00	.	.	intron	'ST3GAL3	protein_coding
1	43,818,862	43,818,863	1,827	5prime(Human)-43818863-5prime(HBV)	1	0.00	.	.	intron	'ST3GAL3	protein_coding
1	46,360,470	46,360,471	1,857	5prime(Human)-46360471-5prime(HBV)	17	0.24	1	0.00	intron	'NSUN4	protein_coding
1	48,040,350	48,040,351	2,882	3prime(Human)-48040351-5prime(HBV)	21	0.00	1	0.00	gene	'LINC02794	lncRNA
1	53,975,455	53,975,456	346	3prime(HBV)-53975456-3prime(Human)	29	0.00	1	0.00	exon	'HNRNPA3P12	processed_pseudogene
1	56,175,797	56,175,798	3,172	3prime(HBV)-56175798-5prime(Human)	22	0.00	1	0.00	intron	'AC119674.1	lncRNA
1	58,981,013	58,981,014	1,813	3prime(HBV)-58981014-5prime(Human)	1	1.00	.	.	intron	'LINC01358	lncRNA
1	58,996,977	58,996,978	1,823	3prime(HBV)-58996978-3prime(Human)	1	1.00	.	.	intron	'LINC01358	lncRNA
1	62,606,484	62,606,485	1,839	5prime(Human)-62606485-5prime(HBV)	1	0.00	1	0.00	intron	'DOCK7	protein_coding

1	62,606,487	62,606,488	1,839	5prime(Human)-62606488-5prime(HBV)	129	0.00	5	0.00	intron	'DOCK7	protein_coding
1	62,606,503	62,606,504	1,823	3prime(HBV)-62606504-3prime(Human)	1	0.00	.	.	intron	'DOCK7	protein_coding
1	62,606,503	62,606,504	1,827	3prime(HBV)-62606504-3prime(Human)	357	0.13	10	0.20	intron	'DOCK7	protein_coding
1	62,606,507	62,606,508	1,827	3prime(HBV)-62606508-3prime(Human)	1	0.00	.	.	intron	'DOCK7	protein_coding
1	70,373,000	70,373,001	2,533	3prime(HBV)-70373001-3prime(Human)	1	1.00	.	.	intron	'HHLA3	protein_coding
1	72,196,462	72,196,463	1,783	3prime(HBV)-72196463-5prime(Human)	129	0.00	3	0.00	intron	'NEGR1	protein_coding
1	72,196,465	72,196,466	2,080	3prime(Human)-72196466-5prime(HBV)	22	0.00	1	0.00	intron	'NEGR1	protein_coding
1	72,984,653	72,984,654	1,827	3prime(HBV)-72984654-5prime(Human)	4	1.00	.	.	gene	'AL732618.1	TEC
1	82,518,472	82,518,473	223	3prime(HBV)-82518473-5prime(Human)	31	0.00	1	0.00	intron	'AL157944.1	lncRNA
1	82,518,473	82,518,474	223	3prime(HBV)-82518474-5prime(Human)	1	0.00	.	.	intron	'AL157944.1	lncRNA
1	82,518,477	82,518,478	1,825	3prime(Human)-82518478-5prime(HBV)	77	0.00	2	0.00	intron	'AL157944.1	lncRNA
1	84,056,303	84,056,304	1,977	5prime(Human)-84056304-5prime(HBV)	2	1.00	1	1.00	intron	'AC104454.2	lncRNA
1	84,879,162	84,879,163	1,768	3prime(HBV)-84879163-3prime(Human)	33	0.00	2	0.00	intron	'LPAR3	protein_coding
1	85,399,888	85,399,889	1,789	3prime(HBV)-85399889-3prime(Human)	29	0.00	1	0.00	intron	'DDAH1	protein_coding
1	85,585,553	85,585,554	1,843	3prime(Human)-85585554-5prime(HBV)	15	0.00	1	0.00	gene	'CCN1	protein_coding
1	85,723,974	85,723,975	1,834	3prime(Human)-85723975-5prime(HBV)	4	1.00	.	.	gene	'COL24A1	protein_coding
1	86,066,517	86,066,518	1,566	3prime(Human)-86066518-5prime(HBV)	24	0.00	1	0.00	intron	'COL24A1	protein_coding
1	86,883,362	86,883,363	1,881	5prime(Human)-86883363-5prime(HBV)	55	0.00	2	0.00	intron	'SELENOF	protein_coding
1	87,946,564	87,946,565	1,828	5prime(Human)-87946565-5prime(HBV)	26	0.00	1	0.00	intron	'PKN2-AS1	lncRNA
1	87,946,571	87,946,572	1,812	3prime(HBV)-87946572-3prime(Human)	21	0.00	1	0.00	intron	'PKN2-AS1	lncRNA
1	94,469,728	94,469,729	2,161	3prime(Human)-94469729-5prime(HBV)	3	1.00	.	.	intron	'ABCD3	protein_coding
1	96,755,213	96,755,214	2,023	5prime(Human)-96755214-5prime(HBV)	1	0.00	.	.	intron	'PTBP2	protein_coding
1	96,755,220	96,755,221	2,023	5prime(Human)-96755221-5prime(HBV)	30	0.00	2	0.00	intron	'PTBP2	protein_coding
1	103,992,808	103,992,809	1,791	3prime(HBV)-103992809-3prime(Human)	37	0.00	1	0.00	gene	'AL136455.1	processed_pseudogene
1	112,861,366	112,861,367	1,813	3prime(HBV)-112861367-5prime(Human)	2	1.00	.	.	intron	'LINC01357	lncRNA
1	114,031,48	114,031,48	2,533	3prime(HBV)-114031488-3prime(Human)	1	1.00	.	.	intron	'OLFML3	lncRNA

	7	8									
1	120,885,058	120,885,059	2,210	3prime(Human)-120885059-5prime(HBV)	9	1.00	1	1.00	gene	'PPIAL4A	protein_coding
1	144,378,250	144,378,251	2,210	5prime(Human)-144378251-5prime(HBV)	6	1.00	.	.	gene	'PPIAL4E	protein_coding
1	144,588,270	144,588,271	2,210	3prime(Human)-144588271-5prime(HBV)	3	1.00	.	.	gene	'PPIAL4F	protein_coding
1	145,246,784	145,246,785	2,210	5prime(Human)-145246785-5prime(HBV)	3	1.00	.	.	gene	'PPIAL4D	protein_coding
1	148,487,920	148,487,921	2,210	5prime(Human)-148487921-5prime(HBV)	3	1.00	.	.	intron	'LINC01138	lncRNA
1	149,579,161	149,579,162	2,210	3prime(Human)-149579162-5prime(HBV)	7	1.00	.	.	gene	'PPIAL4C	protein_coding
1	150,297,464	150,297,465	2,211	3prime(Human)-150297465-5prime(HBV)	1	1.00	1	1.00	intron	'MRPS21	protein_coding
1	164,211,904	164,211,905	1,826	3prime(HBV)-164211905-5prime(Human)	31	0.00	1	0.00	gene	'NMNAT1P2	processed_pseudogene
1	170,061,777	170,061,778	1,845	5prime(Human)-170061778-5prime(HBV)	2	1.00	.	.	intron	'KIFAP3	protein_coding
1	175,023,295	175,023,296	1,820	3prime(HBV)-175023296-5prime(Human)	109	0.00	3	0.00	intron	'MRPS14	protein_coding
1	175,023,352	175,023,353	2,842	3prime(Human)-175023353-5prime(HBV)	94	0.00	3	0.00	intron	'MRPS14	protein_coding
1	176,361,598	176,361,599	1,845	5prime(Human)-176361599-5prime(HBV)	1	1.00	.	.	intron	'AL591043.2	lncRNA
1	180,298,806	180,298,807	1,798	3prime(HBV)-180298807-5prime(Human)	1	0.00	.	.	intron	'ACBD6	protein_coding
1	180,298,80	180,298,80	1,799	3prime(HBV)-180298807-5prime(Human)	1	0.00	.	.	intron	'ACBD6	protein_coding

	6	7									
1	180,298,806	180,298,807	1,800	3prime(HBV)-180298807-5prime(Human)	49	0.00	3	0.00	intron	'ACBD6	protein_coding
1	190,440,556	190,440,557	1,820	5prime(Human)-190440557-5prime(HBV)	24	0.00	1	0.00	intron	'BRINP3	protein_coding
1	190,704,656	190,704,657	1,871	5prime(Human)-190704657-5prime(HBV)	34	0.00	1	0.00	intron	'LINC01720	lncRNA
1	192,624,570	192,624,571	1,834	5prime(Human)-192624571-5prime(HBV)	2	1.00	.	.	intron	'AL390957.1	lncRNA
1	196,749,527	196,749,528	1,498	3prime(HBV)-196749528-5prime(Human)	37	1.00	2	1.00	gene	'CFH	protein_coding
1	196,756,127	196,756,128	1,549	3prime(HBV)-196756128-5prime(Human)	30	0.00	1	0.00	gene	'CFH	protein_coding
1	196,756,148	196,756,149	1,929	3prime(Human)-196756149-5prime(HBV)	63	0.00	3	0.00	gene	'CFH	protein_coding
1	196,792,230	196,792,231	1,822	3prime(HBV)-196792231-3prime(Human)	76	1.00	4	1.00	intron	'CFHR3	protein_coding
1	196,792,231	196,792,232	1,823	3prime(HBV)-196792232-3prime(Human)	1	1.00	.	.	intron	'CFHR3	protein_coding
1	196,834,213	196,834,214	1,498	3prime(HBV)-196834214-5prime(Human)	25	1.00	1	1.00	gene	'CFHR1	protein_coding
1	196,917,119	196,917,120	1,817	3prime(HBV)-196917120-3prime(Human)	1	1.00	.	.	intron	'CFHR4	protein_coding
1	196,917,119	196,917,120	1,822	3prime(HBV)-196917120-3prime(Human)	75	1.00	4	1.00	intron	'CFHR4	protein_coding
1	205,885,576	205,885,577	2,533	3prime(HBV)-205885577-5prime(Human)	1	1.00	.	.	intron	'AC119673.2	lncRNA
1	212,845,79	212,845,79	2,533	3prime(HBV)-212845795-3prime(Human)	1	1.00	.	.	intron	'SPATA45	protein_coding

	4	5									
1	214,009,997	214,009,998	1,819	3prime(Human)-214009998-5prime(HBV)	61	0.00	2	0.00	intron	'PROX1	protein_coding
1	214,009,997	214,009,998	1,821	3prime(Human)-214009998-5prime(HBV)	1	0.00	1	0.00	intron	'PROX1	protein_coding
1	214,974,875	214,974,876	204	3prime(Human)-214974876-5prime(HBV)	31	0.00	1	0.00	intron	'AC099563.2	lncRNA
1	227,080,190	227,080,191	1,826	3prime(HBV)-227080191-3prime(Human)	54	0.00	2	0.00	intron	'CDC42BPA	protein_coding
1	233,942,143	233,942,144	1,809	3prime(HBV)-233942144-3prime(Human)	3	1.00	.	.	intron	'SLC35F3	protein_coding
1	240,435,796	240,435,797	1,820	3prime(HBV)-240435797-5prime(Human)	1	0.00	.	.	intron	'FMN2	protein_coding
1	240,435,797	240,435,798	1,820	3prime(HBV)-240435798-5prime(Human)	21	0.00	1	0.00	intron	'FMN2	protein_coding
1	242,590,474	242,590,475	1,827	3prime(HBV)-242590475-5prime(Human)	1	1.00	1	1.00	gene	'PLD5	protein_coding
1	243,176,773	243,176,774	1,826	3prime(HBV)-243176774-5prime(Human)	31	1.00	2	1.00	intron	'CEP170	protein_coding
1	245,628,028	245,628,029	1,827	3prime(HBV)-245628029-5prime(Human)	1	1.00	1	1.00	intron	'KIF26B	protein_coding
1	245,867,451	245,867,452	1,814	3prime(HBV)-245867452-3prime(Human)	29	0.00	1	0.00	intron	'SMYD3	protein_coding
1	248,940,986	248,940,987	1,824	3prime(HBV)-248940987-3prime(Human)	7	1.00	2	1.00	gene	'RPL23AP25	processed_pseudogene
2	3,350,106	3,350,107	1,851	3prime(Human)-3350107-5prime(HBV)	1	0.00	1	0.00	intron	'EIPR1	protein_coding
2	5,356,517	5,356,518	1,828	5prime(Human)-5356518-5prime(HBV)	27	0.00	1	0.00	gene	'AC073143.1	processed_pseudogene
2	5,356,517	5,356,518	1,833	5prime(Human)-5356518-5prime(HBV)	1	0.00	.	.	gene	'AC073143.1	processed_pseudogene

2	8,053,754	8,053,755	1,199	5prime(Human)-8053755-5prime(HBV)	34	0.00	1	0.00	intron	'LINC00299	lncRNA
2	11,661,697	11,661,698	1,826	3prime(HBV)-11661698-3prime(Human)	13	0.00	1	0.00	intron	'NTSR2	protein_coding
2	25,872,718	25,872,719	1,878	3prime(HBV)-25872719-5prime(Human)	21	0.00	1	0.00	intron	'ASXL2	protein_coding
2	27,073,442	27,073,443	2,011	3prime(Human)-27073443-5prime(HBV)	5	1.00	.	.	gene	'OST4	protein_coding
2	28,588,998	28,588,999	1,809	3prime(HBV)-28588999-5prime(Human)	3	1.00	.	.	intron	'PLB1	protein_coding
2	32,586,462	32,586,463	1,851	5prime(Human)-32586463-5prime(HBV)	1	1.00	1	1.00	intron	'BIRC6	protein_coding
2	34,092,520	34,092,521	1,826	3prime(HBV)-34092521-3prime(Human)	107	0.00	2	0.00	intron	'LINC01320	lncRNA
2	40,143,767	40,143,768	443	3prime(HBV)-40143768-3prime(Human)	23	0.00	1	0.00	intron	'SLC8A1	protein_coding
2	44,019,315	44,019,316	2,533	3prime(HBV)-44019316-5prime(Human)	1	1.00	.	.	gene	'LRPPRC	protein_coding
2	45,113,552	45,113,553	1,831	3prime(HBV)-45113553-5prime(Human)	18	0.00	1	0.00	gene	'LINC01121	lncRNA
2	48,819,477	48,819,478	1,807	5prime(Human)-48819478-5prime(HBV)	1	0.00	.	.	intron	'AC009975.1	lncRNA
2	48,819,480	48,819,481	1,807	5prime(Human)-48819481-5prime(HBV)	76	0.00	2	0.00	intron	'AC009975.1	lncRNA
2	61,926,033	61,926,034	2,161	3prime(Human)-61926034-5prime(HBV)	3	1.00	.	.	intron	'COMMD1	protein_coding
2	69,460,124	69,460,125	1,794	3prime(HBV)-69460125-5prime(Human)	24	0.00	1	0.00	exon	'AAK1	protein_coding
2	77,875,448	77,875,449	1,826	3prime(HBV)-77875449-3prime(Human)	86	0.00	2	0.00	intron	'AC012494.1	lncRNA
2	84,595,151	84,595,152	1,784	3prime(HBV)-84595152-3prime(Human)	51	0.00	1	0.00	intron	'DNAH6	protein_coding
2	84,595,155	84,595,156	1,784	3prime(HBV)-84595156-3prime(Human)	1	0.00	.	.	intron	'DNAH6	protein_coding
2	94,901,221	94,901,222	2,910	3prime(HBV)-94901222-3prime(Human)	4	1.00	.	.	exon	'MTCO3P45	processed_pseudogene
2	96,743,990	96,743,991	2,011	3prime(Human)-96743991-5prime(HBV)	1	1.00	.	.	gene	'LMAN2L	protein_coding
2	113,648,373	113,648,374	1,813	3prime(HBV)-113648374-5prime(Human)	2	1.00	.	.	gene	'RABL2A	protein_coding
2	115,797,940	115,797,941	1,823	3prime(HBV)-115797941-3prime(Human)	2	0.50	2	0.50	intron	'DPP10	protein_coding
2	115,797,942	115,797,943	1,823	3prime(HBV)-115797943-3prime(Human)	2	1.00	.	.	intron	'DPP10	protein_coding
2	119,366,603	119,366,604	1,815	3prime(HBV)-119366604-5prime(Human)	17	0.00	1	0.00	exon	'C2orf76	protein_coding
2	123,194,94	123,194,94	1,812	3prime(HBV)-123194947-5prime(Human)	33	0.00	1	0.00	gene	'LINC01826	lncRNA

	6	7									
2	123,194,953	123,194,954	1,825	3prime(Human)-123194954-5prime(HBV)	32	0.00	1	0.00	gene	'LINC01826	lncRNA
2	123,252,428	123,252,429	1,820	3prime(Human)-123252429-5prime(HBV)	68	0.00	1	0.00	gene	'AC073409.2	lncRNA
2	123,252,431	123,252,432	1,826	3prime(HBV)-123252432-5prime(Human)	60	0.00	1	0.00	gene	'AC073409.2	lncRNA
2	124,958,716	124,958,717	1,814	3prime(HBV)-124958717-5prime(Human)	1	1.00	1	1.00	gene	'RNA5SP102	rRNA_pseudogene
2	126,410,988	126,410,989	1,554	3prime(HBV)-126410989-5prime(Human)	1	0.00	.	.	gene	'AC023347.1	lncRNA
2	126,410,992	126,410,993	1,554	3prime(HBV)-126410993-5prime(Human)	86	0.00	2	0.00	gene	'AC023347.1	lncRNA
2	127,244,513	127,244,514	2,349	5prime(Human)-127244514-5prime(HBV)	1	1.00	.	.	gene	'AC110926.1	processed_pseudogene
2	129,825,350	129,825,351	1,816	3prime(HBV)-129825351-3prime(Human)	42	0.00	1	0.00	exon	'LINC02572	lncRNA
2	137,998,308	137,998,309	1,922	5prime(Human)-137998309-5prime(HBV)	24	0.00	1	0.00	intron	'HNMT	protein_coding
2	140,824,259	140,824,260	1,828	5prime(Human)-140824260-5prime(HBV)	22	0.00	1	0.00	intron	'LRP1B	protein_coding
2	141,541,001	141,541,002	739	3prime(Human)-141541002-5prime(HBV)	21	0.00	1	0.00	intron	'LRP1B	protein_coding
2	155,180,454	155,180,455	1,824	3prime(HBV)-155180455-3prime(Human)	80	0.00	2	0.00	gene	'MTCO1P45	processed_pseudogene
2	164,603,498	164,603,499	1,809	3prime(HBV)-164603499-5prime(Human)	2	1.00	.	.	intron	'GRB14	protein_coding
2	168,394,39	168,394,39	1,534	3prime(Human)-168394392-5prime(HBV)	1	0.00	1	0.00	gene	'RN7SL813P	misc_RNA

	1	2									
2	180,080,250	180,080,251	1,819	3prime(Human)-180080251-5prime(HBV)	34	0.00	1	0.00	gene	'CWC22	protein_coding
2	180,787,435	180,787,436	1,798	3prime(HBV)-180787436-3prime(Human)	20	0.00	1	0.00	intron	'SCHLAP1	lncRNA
2	184,568,658	184,568,659	1,825	3prime(HBV)-184568659-5prime(Human)	18	0.00	1	0.00	gene	'AC096667.1	lncRNA
2	184,569,344	184,569,345	1,818	3prime(Human)-184569345-5prime(HBV)	8	0.00	1	0.00	gene	'AC096667.1	lncRNA
2	189,640,866	189,640,867	1,829	3prime(Human)-189640867-5prime(HBV)	1	0.00	.	.	gene	'AC012488.1	unprocessed_pseudogene
2	193,067,271	193,067,272	2,325	3prime(Human)-193067272-5prime(HBV)	1	1.00	1	1.00	gene	'AC096647.1	lncRNA
2	193,759,282	193,759,283	1,817	3prime(HBV)-193759283-3prime(Human)	23	0.00	1	0.00	gene	'AC074290.1	processed_pseudogene
2	193,942,783	193,942,784	2,608	3prime(Human)-193942784-5prime(HBV)	23	0.00	1	0.00	gene	'AC068135.2	lncRNA
2	194,478,097	194,478,098	1,810	3prime(HBV)-194478098-3prime(Human)	1	0.00	.	.	intron	'LINC01821	lncRNA
2	194,478,097	194,478,098	1,815	3prime(HBV)-194478098-3prime(Human)	81	0.48	4	0.50	intron	'LINC01821	lncRNA
2	194,492,534	194,492,535	1,829	5prime(Human)-194492535-5prime(HBV)	1	0.00	.	.	intron	'LINC01821	lncRNA
2	204,970,608	204,970,609	1,845	3prime(Human)-204970609-5prime(HBV)	35	0.00	1	0.00	intron	'PARD3B	protein_coding
2	210,434,160	210,434,161	1,841	5prime(Human)-210434161-5prime(HBV)	27	0.00	1	0.00	exon	'LANCL1	protein_coding
2	210,434,16	210,434,16	1,844	5prime(Human)-210434161-5prime(HBV)	1	0.00	.	.	exon	'LANCL1	protein_coding

	0	1									
2	211,039,394	211,039,395	1,845	5prime(Human)-211039395-5prime(HBV)	1	1.00	.	.	gene	'AC012491.1	processed_pseudogene
2	215,418,186	215,418,187	1,818	3prime(Human)-215418187-5prime(HBV)	329	0.07	13	0.08	intron	'FN1	protein_coding
2	215,418,186	215,418,187	1,823	3prime(Human)-215418187-5prime(HBV)	1	0.00	.	.	intron	'FN1	protein_coding
2	215,418,187	215,418,188	1,130	3prime(HBV)-215418188-5prime(Human)	48	0.40	1	0.00	intron	'FN1	protein_coding
2	215,418,188	215,418,189	1,828	3prime(Human)-215418189-5prime(HBV)	1	0.00	.	.	intron	'FN1	protein_coding
2	216,565,351	216,565,352	2,161	3prime(Human)-216565352-5prime(HBV)	4	1.00	.	.	intron	'RPL37A	protein_coding
2	234,199,580	234,199,581	1,802	5prime(Human)-234199581-5prime(HBV)	1	1.00	.	.	gene	'AC122134.1	lncRNA
2	238,674,077	238,674,078	1,802	3prime(Human)-238674078-5prime(HBV)	3	1.00	.	.	intron	'LINC01937	lncRNA
2	240,342,329	240,342,330	929	5prime(Human)-240342330-5prime(HBV)	1	0.00	1	0.00	gene	'U3	snoRNA
2	242,003,144	242,003,145	1,826	3prime(HBV)-242003145-3prime(Human)	1	0.00	.	.	exon	'AC093642.2	TEC
2	242,003,144	242,003,145	1,827	3prime(HBV)-242003145-3prime(Human)	97	0.00	3	0.00	exon	'AC093642.2	TEC
3	538,666	538,667	1,825	3prime(HBV)-538667-5prime(Human)	1	0.00	.	.	intron	'LINC01266	lncRNA
3	538,668	538,669	1,825	3prime(HBV)-538669-5prime(Human)	25	0.00	2	0.00	intron	'LINC01266	lncRNA
3	538,670	538,671	1,841	3prime(Human)-538671-5prime(HBV)	24	0.00	1	0.00	intron	'LINC01266	lncRNA
3	12,606,871	12,606,872	1,809	3prime(HBV)-12606872-3prime(Human)	4	1.00	.	.	intron	'RAF1	protein_coding
3	14,590,465	14,590,466	1,817	3prime(HBV)-14590466-3prime(Human)	55	0.00	1	0.00	gene	'AC090952.2	lncRNA

3	14,590,469	14,590,470	1,817	3prime(HBV)-14590470-3prime(Human)	1	0.00	.	.	gene	'AC090952.2	lncRNA
3	14,590,477	14,590,478	2,121	5prime(Human)-14590478-5prime(HBV)	71	0.23	3	0.33	gene	'AC090952.2	lncRNA
3	14,590,477	14,590,478	2,123	5prime(Human)-14590478-5prime(HBV)	1	0.00	.	.	gene	'AC090952.2	lncRNA
3	16,438,572	16,438,573	1,847	3prime(Human)-16438573-5prime(HBV)	6	1.00	6	1.00	intron	'RFTN1	protein_coding
3	17,642,902	17,642,903	1,827	5prime(Human)-17642903-5prime(HBV)	96	0.00	3	0.00	intron	'TBC1D5	protein_coding
3	17,642,914	17,642,915	1,815	3prime(HBV)-17642915-3prime(Human)	37	0.00	2	0.00	intron	'TBC1D5	protein_coding
3	17,859,289	17,859,290	1,977	5prime(Human)-17859290-5prime(HBV)	1	1.00	1	1.00	intron	'TBC1D5	lncRNA
3	24,276,298	24,276,299	1,855	5prime(Human)-24276299-5prime(HBV)	18	0.00	1	0.00	intron	'THRB	protein_coding
3	24,276,298	24,276,299	1,858	5prime(Human)-24276299-5prime(HBV)	1	0.00	.	.	intron	'THRB	protein_coding
3	31,958,809	31,958,810	1,781	5prime(Human)-31958810-5prime(HBV)	55	0.00	1	0.00	intron	'OSBPL10	protein_coding
3	32,473,768	32,473,769	2,866	3prime(HBV)-32473769-5prime(Human)	34	0.00	1	0.00	intron	'CMTM7	nonsense_mediated_decay
3	35,731,894	35,731,895	1,750	3prime(Human)-35731895-5prime(HBV)	16	1.00	1	1.00	intron	'ARPP21	protein_coding
3	35,731,896	35,731,897	1,799	3prime(HBV)-35731897-5prime(Human)	29	0.00	1	0.00	intron	'ARPP21	protein_coding
3	37,208,927	37,208,928	2,533	3prime(HBV)-37208928-3prime(Human)	2	1.00	.	.	gene	'AC097359.3	lncRNA
3	43,665,139	43,665,140	1,845	5prime(Human)-43665140-5prime(HBV)	1	1.00	.	.	intron	'ANO10	protein_coding
3	44,788,963	44,788,964	1,793	3prime(HBV)-44788964-5prime(Human)	12	0.00	1	0.00	intron	'KIF15	protein_coding
3	44,908,408	44,908,409	207	3prime(HBV)-44908409-5prime(Human)	1	0.00	.	.	intron	'TGM4	protein_coding
3	44,908,408	44,908,409	210	3prime(HBV)-44908409-5prime(Human)	20	0.00	1	0.00	intron	'TGM4	protein_coding
3	44,908,410	44,908,411	210	3prime(HBV)-44908411-5prime(Human)	1	0.00	.	.	intron	'TGM4	protein_coding
3	49,196,733	49,196,734	2,161	3prime(Human)-49196734-5prime(HBV)	4	1.00	.	.	gene	'CCDC36	protein_coding
3	53,255,391	53,255,392	1,778	3prime(HBV)-53255392-5prime(Human)	1	0.00	.	.	intron	'TKT	protein_coding
3	53,255,394	53,255,395	1,778	3prime(HBV)-53255395-5prime(Human)	105	0.00	3	0.00	intron	'TKT	protein_coding
3	58,933,010	58,933,011	1,977	3prime(Human)-58933011-5prime(HBV)	2	1.00	1	1.00	intron	'C3orf67	protein_coding
3	77,598,515	77,598,516	1,823	3prime(HBV)-77598516-5prime(Human)	1	0.00	1	0.00	intron	'ROBO2	protein_coding
3	80,390,480	80,390,481	2,533	3prime(HBV)-80390481-3prime(Human)	1	1.00	.	.	gene	'AC108740.1	unprocessed_pseudogene
3	86,538,084	86,538,085	2,533	3prime(HBV)-86538085-5prime(Human)	2	1.00	.	.	gene	'LINC02070	lncRNA
3	87,574,809	87,574,810	2,533	3prime(HBV)-87574810-3prime(Human)	2	1.00	.	.	gene	'APOOP2	processed_pseudogene
3	87,764,720	87,764,721	2,652	3prime(Human)-87764721-5prime(HBV)	20	0.00	1	0.00	intron	'AC108749.1	lncRNA

3	88,233,192	88,233,193	1,837	3prime(Human)-88233193-5prime(HBV)	35	0.00	1	0.00	gene	'C3orf38	lncRNA
3	88,819,243	88,819,244	1,822	3prime(HBV)-88819244-5prime(Human)	1	0.00	.	.	gene	'ICE2P2	processed_pseudogene
3	88,819,248	88,819,249	1,823	3prime(HBV)-88819249-5prime(Human)	128	0.01	4	0.00	gene	'ICE2P2	processed_pseudogene
3	88,819,249	88,819,250	1,823	3prime(HBV)-88819250-5prime(Human)	1	0.00	.	.	gene	'ICE2P2	processed_pseudogene
3	95,661,235	95,661,236	1,977	5prime(Human)-95661236-5prime(HBV)	1	1.00	1	1.00	intron	'MTHFD2P1	lncRNA
3	98,534,851	98,534,852	1,825	5prime(Human)-98534852-5prime(HBV)	39	0.00	1	0.00	intron	'AC021660.3	nonsense_mediated_decay
3	101,693,383	101,693,384	1,834	5prime(Human)-101693384-5prime(HBV)	29	0.00	2	0.00	intron	'AC084198.2	lncRNA
3	101,693,404	101,693,405	1,802	3prime(HBV)-101693405-3prime(Human)	36	0.50	4	0.75	intron	'AC084198.2	lncRNA
3	101,693,406	101,693,407	1,800	3prime(HBV)-101693407-3prime(Human)	1	0.00	.	.	intron	'AC084198.2	lncRNA
3	101,738,139	101,738,140	1,834	3prime(Human)-101738140-5prime(HBV)	5	1.00	.	.	intron	'CEP97	protein_coding
3	108,349,541	108,349,542	1,827	3prime(HBV)-108349542-5prime(Human)	1	1.00	1	1.00	intron	'HHLA2	protein_coding
3	114,663,627	114,663,628	1,977	3prime(Human)-114663628-5prime(HBV)	1	1.00	1	1.00	intron	'ZBTB20	protein_coding
3	117,414,906	117,414,907	1,767	3prime(HBV)-117414907-5prime(Human)	1	0.00	.	.	gene	'RNU6-1200P	snRNA
3	117,414,909	117,414,910	1,767	3prime(HBV)-117414910-5prime(Human)	113	0.00	3	0.00	gene	'RNU6-1200P	snRNA
3	117,427,432	117,427,433	2,279	3prime(Human)-117427433-5prime(HBV)	68	0.00	1	0.00	gene	'RNU6-1200P	snRNA
3	134,949,388	134,949,389	1,802	3prime(HBV)-134949389-5prime(Human)	1	0.00	.	.	intron	'EPHB1	protein_coding
3	134,949,388	134,949,389	1,807	3prime(HBV)-134949389-5prime(Human)	84	0.00	1	0.00	intron	'EPHB1	protein_coding

3	134,949,394	134,949,395	2,348	3prime(Human)-134949395-5prime(HBV)	80	0.00	3	0.00	intron	'EPHB1	protein_coding
3	134,949,397	134,949,398	2,348	3prime(Human)-134949398-5prime(HBV)	1	0.00	.	.	intron	'EPHB1	protein_coding
3	137,682,725	137,682,726	1,779	3prime(HBV)-137682726-3prime(Human)	27	0.00	1	0.00	gene	'NPM1P17	processed_pseudogene
3	138,564,245	138,564,246	1,827	3prime(HBV)-138564246-5prime(Human)	1	1.00	1	1.00	intron	'CEP70	protein_coding
3	146,419,071	146,419,072	2,302	5prime(Human)-146419072-5prime(HBV)	89	0.15	4	0.25	intron	'PLSCR2	nonsense_mediated_decay
3	146,419,100	146,419,101	1,793	3prime(HBV)-146419101-3prime(Human)	25	0.00	1	0.00	intron	'PLSCR2	nonsense_mediated_decay
3	154,391,138	154,391,139	1,811	3prime(HBV)-154391139-5prime(Human)	28	0.00	1	0.00	intron	'GPR149	protein_coding
3	157,726,797	157,726,798	1,977	3prime(Human)-157726798-5prime(HBV)	1	1.00	1	1.00	gene	'SLC66A1L	lncRNA
3	162,154,411	162,154,412	2,533	3prime(HBV)-162154412-5prime(Human)	1	1.00	.	.	gene	'TOMM22P6	processed_pseudogene
3	162,363,282	162,363,283	1,701	3prime(HBV)-162363283-3prime(Human)	25	0.00	1	0.00	gene	'TOMM22P6	processed_pseudogene
3	162,363,364	162,363,365	1,798	3prime(HBV)-162363365-5prime(Human)	1	1.00	.	.	gene	'TOMM22P6	processed_pseudogene
3	162,363,367	162,363,368	1,792	3prime(HBV)-162363368-5prime(Human)	2	0.50	1	0.00	gene	'TOMM22P6	processed_pseudogene
3	162,363,367	162,363,368	1,797	3prime(HBV)-162363368-5prime(Human)	123	0.09	3	0.33	gene	'TOMM22P6	processed_pseudogene
3	162,697,239	162,697,240	1,835	5prime(Human)-162697240-5prime(HBV)	1	0.00	.	.	gene	'AC128716.1	processed_pseudogene

3	162,697,239	162,697,240	1,840	5prime(Human)-162697240-5prime(HBV)	1	0.00	.	.	gene	'AC128716.1	processed_pseudogene
3	162,697,241	162,697,242	1,835	5prime(Human)-162697242-5prime(HBV)	50	0.00	1	0.00	gene	'AC128716.1	processed_pseudogene
3	162,699,461	162,699,462	1,827	3prime(HBV)-162699462-3prime(Human)	35	0.00	1	0.00	gene	'AC128716.1	processed_pseudogene
3	164,231,969	164,231,970	1,819	5prime(Human)-164231970-5prime(HBV)	27	0.00	.	.	gene	'MIR1263	miRNA
3	165,226,491	165,226,492	1,821	3prime(HBV)-165226492-3prime(Human)	63	0.00	1	0.00	intron	'LINC01322	lncRNA
3	174,709,784	174,709,785	2,062	3prime(HBV)-174709785-5prime(Human)	12	0.00	1	0.00	intron	'NAALADL2	protein_coding
3	174,709,794	174,709,795	2,239	3prime(Human)-174709795-5prime(HBV)	50	0.00	2	0.00	intron	'NAALADL2	protein_coding
3	179,369,859	179,369,860	2,546	3prime(HBV)-179369860-5prime(Human)	1	1.00	.	.	intron	'MFN1	protein_coding
3	179,369,861	179,369,862	2,546	3prime(HBV)-179369862-5prime(Human)	1	0.00	.	.	intron	'MFN1	protein_coding
3	179,369,863	179,369,864	2,546	3prime(HBV)-179369864-5prime(Human)	40	0.00	2	0.00	intron	'MFN1	protein_coding
3	181,045,035	181,045,036	1,819	3prime(Human)-181045036-5prime(HBV)	9	1.00	.	.	intron	'SOX2-OT	lncRNA
3	186,678,151	186,678,152	1,677	5prime(Human)-186678152-5prime(HBV)	1	0.00	.	.	exon	'HRG	protein_coding
3	186,678,158	186,678,159	1,677	5prime(Human)-186678159-5prime(HBV)	1	0.00	.	.	exon	'HRG	protein_coding
3	186,678,159	186,678,160	1,677	5prime(Human)-186678160-5prime(HBV)	72	0.00	3	0.00	exon	'HRG	protein_coding

3	186,678,165	186,678,166	1,601	3prime(HBV)-186678166-3prime(Human)	77	0.01	3	0.00	exon	'HRG	protein_coding
4	6,106,034	6,106,035	887	3prime(HBV)-6106035-5prime(Human)	1	0.00	1	0.00	intron	'JAKMIP1	protein_coding
4	20,686,781	20,686,782	1,894	5prime(Human)-20686782-5prime(HBV)	29	0.00	1	0.00	gene	'PACRGL	nonsense_mediated_decay
4	29,639,014	29,639,015	1,793	3prime(HBV)-29639015-5prime(Human)	21	0.00	1	0.00	gene	'EEF1A1P21	processed_pseudogene
4	29,639,015	29,639,016	1,829	3prime(Human)-29639016-5prime(HBV)	127	0.00	6	0.00	gene	'EEF1A1P21	processed_pseudogene
4	34,964,090	34,964,091	1,805	3prime(HBV)-34964091-5prime(Human)	1	0.00	.	.	gene	'AC020589.1	snoRNA
4	34,964,090	34,964,091	1,806	3prime(HBV)-34964091-5prime(Human)	104	0.00	4	0.00	gene	'AC020589.1	snoRNA
4	34,964,105	34,964,106	1,676	3prime(HBV)-34964106-3prime(Human)	69	0.00	2	0.00	gene	'AC020589.1	snoRNA
4	34,964,110	34,964,111	1,676	3prime(HBV)-34964111-3prime(Human)	1	0.00	.	.	gene	'AC020589.1	snoRNA
4	42,983,857	42,983,858	1,834	3prime(Human)-42983858-5prime(HBV)	1	1.00	1	1.00	intron	'GRXCR1	protein_coding
4	45,691,245	45,691,246	2,533	3prime(HBV)-45691246-3prime(Human)	1	1.00	.	.	gene	'RNU6-931P	snRNA
4	48,005,605	48,005,606	1,824	5prime(Human)-48005606-5prime(HBV)	1	0.00	.	.	intron	'CNGA1	protein_coding
4	48,005,606	48,005,607	1,821	5prime(Human)-48005607-5prime(HBV)	25	0.04	1	0.00	intron	'CNGA1	protein_coding
4	49,246,163	49,246,164	2,910	3prime(HBV)-49246164-3prime(Human)	3	1.00	.	.	exon	'MTCO3P39	processed_pseudogene
4	52,807,764	52,807,765	1,845	5prime(Human)-52807765-5prime(HBV)	3	1.00	.	.	intron	'LINC01618	lncRNA
4	53,249,675	53,249,676	1,819	5prime(Human)-53249676-5prime(HBV)	12	1.00	.	.	intron	'SCFD2	protein_coding
4	53,879,218	53,879,219	1,977	3prime(Human)-53879219-5prime(HBV)	1	1.00	1	1.00	intron	'AC058822.1	protein_coding
4	59,220,471	59,220,472	1,829	3prime(Human)-59220472-5prime(HBV)	56	0.00	1	0.00	gene	'AC108517.2	lncRNA
4	59,220,477	59,220,478	1,829	3prime(Human)-59220478-5prime(HBV)	1	0.00	1	0.00	gene	'AC108517.2	lncRNA
4	60,276,303	60,276,304	1,819	3prime(Human)-60276304-5prime(HBV)	35	0.00	1	0.00	gene	'AC097655.1	processed_pseudogene
4	62,222,372	62,222,373	1,821	3prime(HBV)-62222373-3prime(Human)	1	0.00	.	.	gene	'RPL21P47	processed_pseudogene
4	62,222,372	62,222,373	1,826	3prime(HBV)-62222373-3prime(Human)	118	0.00	3	0.00	gene	'RPL21P47	processed_pseudogene
4	62,222,373	62,222,374	1,953	5prime(Human)-62222374-5prime(HBV)	26	0.00	1	0.00	gene	'RPL21P47	processed_pseudogene
4	62,222,376	62,222,377	1,826	3prime(HBV)-62222377-3prime(Human)	1	0.00	.	.	gene	'RPL21P47	processed_pseudogene
4	63,956,929	63,956,930	1,778	3prime(HBV)-63956930-5prime(Human)	20	0.00	1	0.00	gene	'TECRL	protein_coding
4	63,956,929	63,956,930	1,823	3prime(Human)-63956930-5prime(HBV)	49	0.00	2	0.00	gene	'TECRL	protein_coding
4	63,956,929	63,956,930	1,828	3prime(Human)-63956930-5prime(HBV)	1	0.00	.	.	gene	'TECRL	protein_coding

4	65,271,980	65,271,981	1,845	3prime(Human)-65271981-5prime(HBV)	1	1.00	.	.	gene	'LINC02835	lncRNA
4	66,356,195	66,356,196	1,804	3prime(HBV)-66356196-5prime(Human)	1	0.00	.	.	gene	'AC110809.1	lncRNA
4	66,356,197	66,356,198	1,803	3prime(HBV)-66356198-5prime(Human)	29	0.00	1	0.00	gene	'AC110809.1	lncRNA
4	66,367,894	66,367,895	1,825	3prime(HBV)-66367895-5prime(Human)	34	0.00	1	0.00	gene	'AC110809.1	lncRNA
4	68,666,959	68,666,960	1,931	5prime(Human)-68666960-5prime(HBV)	32	0.00	1	0.00	intron	'UGT2B15	protein_coding
4	68,993,108	68,993,109	1,822	3prime(HBV)-68993109-5prime(Human)	14	0.00	1	0.00	gene	'AC021146.7	processed_pseudogene
4	69,078,410	69,078,411	1,827	3prime(HBV)-69078411-3prime(Human)	1	1.00	1	1.00	intron	'UGT2B7	protein_coding
4	74,978,402	74,978,403	1,834	3prime(Human)-74978403-5prime(HBV)	6	1.00	.	.	intron	'PARM1	protein_coding
4	77,544,142	77,544,143	1,845	3prime(Human)-77544143-5prime(HBV)	1	1.00	.	.	intron	'CXCL13	protein_coding
4	79,162,799	79,162,800	1,787	3prime(HBV)-79162800-3prime(Human)	1	0.00	1	0.00	intron	'LINC01088	lncRNA
4	79,162,799	79,162,800	1,791	3prime(HBV)-79162800-3prime(Human)	29	0.00	1	0.00	intron	'LINC01088	lncRNA
4	81,477,485	81,477,486	1,827	3prime(HBV)-81477486-5prime(Human)	1	1.00	1	1.00	intron	'RASGEF1B	protein_coding
4	81,477,486	81,477,487	1,827	3prime(HBV)-81477487-5prime(Human)	1	1.00	.	.	intron	'RASGEF1B	protein_coding
4	83,674,549	83,674,550	1,818	3prime(Human)-83674550-5prime(HBV)	27	0.00	1	0.00	intron	'AC021192.1	lncRNA
4	83,674,549	83,674,550	1,821	3prime(Human)-83674550-5prime(HBV)	2	0.00	.	.	intron	'AC021192.1	lncRNA
4	85,940,412	85,940,413	1,763	3prime(HBV)-85940413-5prime(Human)	26	0.00	1	0.00	intron	'ARHGAP24	protein_coding
4	94,334,371	94,334,372	1,827	3prime(HBV)-94334372-3prime(Human)	26	0.00	1	0.00	intron	'HPGDS	protein_coding
4	94,567,025	94,567,026	1,796	3prime(HBV)-94567026-5prime(Human)	27	1.00	1	1.00	intron	'PDLIM5	protein_coding
4	94,567,040	94,567,041	1,802	3prime(Human)-94567041-5prime(HBV)	82	0.01	3	0.00	intron	'PDLIM5	protein_coding
4	94,567,043	94,567,044	1,802	3prime(Human)-94567044-5prime(HBV)	1	0.00	.	.	intron	'PDLIM5	protein_coding
4	98,101,228	98,101,229	1,822	5prime(Human)-98101229-5prime(HBV)	61	0.00	1	0.00	intron	'STPG2	protein_coding
4	98,978,033	98,978,034	1,769	3prime(HBV)-98978034-5prime(Human)	16	0.00	.	.	intron	'AC019131.1	lncRNA
4	98,978,034	98,978,035	1,769	3prime(HBV)-98978035-5prime(Human)	7	0.00	1	0.00	intron	'AC019131.1	lncRNA
4	98,978,035	98,978,036	1,764	3prime(HBV)-98978036-5prime(Human)	1	0.00	.	.	intron	'AC019131.1	lncRNA
4	98,978,036	98,978,037	1,769	3prime(HBV)-98978037-5prime(Human)	7	0.00	.	.	intron	'AC019131.1	lncRNA
4	99,229,728	99,229,729	1,827	3prime(HBV)-99229729-3prime(Human)	2	1.00	1	1.00	intron	'AP002026.1	lncRNA
4	99,847,109	99,847,110	312	3prime(HBV)-99847110-5prime(Human)	1	0.00	.	.	intron	'DAPP1	protein_coding
4	99,847,109	99,847,110	316	3prime(HBV)-99847110-5prime(Human)	10	0.00	1	0.00	intron	'DAPP1	protein_coding

4	103,922,123	103,922,124	1,821	3prime(HBV)-103922124-3prime(Human)	23	0.00	1	0.00	gene	'RNU6-635P	snRNA
4	104,315,340	104,315,341	1,840	5prime(Human)-104315341-5prime(HBV)	117	0.01	3	0.00	intron	'AC004052.1	lncRNA
4	104,315,358	104,315,359	1,792	3prime(HBV)-104315359-3prime(Human)	82	0.01	3	0.00	intron	'AC004052.1	lncRNA
4	104,690,810	104,690,811	1,977	5prime(Human)-104690811-5prime(HBV)	1	1.00	1	1.00	intron	'AC004053.1	lncRNA
4	104,695,063	104,695,064	2,331	5prime(Human)-104695064-5prime(HBV)	1	0.00	.	.	intron	'AC004053.1	lncRNA
4	104,695,066	104,695,067	2,331	5prime(Human)-104695067-5prime(HBV)	59	0.00	1	0.00	intron	'AC004053.1	lncRNA
4	112,712,989	112,712,990	2,059	5prime(Human)-112712990-5prime(HBV)	27	0.00	1	0.00	gene	'AC106864.1	lncRNA
4	112,924,669	112,924,670	1,888	3prime(Human)-112924670-5prime(HBV)	17	0.00	1	0.00	intron	'ANK2	protein_coding
4	116,152,655	116,152,656	1,823	3prime(HBV)-116152656-3prime(Human)	31	0.00	1	0.00	intron	'AC027613.1	lncRNA
4	116,152,658	116,152,659	1,824	3prime(HBV)-116152659-3prime(Human)	1	0.00	.	.	intron	'AC027613.1	lncRNA
4	118,502,875	118,502,876	1,826	3prime(HBV)-118502876-3prime(Human)	32	1.00	1	1.00	intron	'CEP170P1	transcribed_unprocessed_pseudogene
4	145,805,612	145,805,613	1,827	3prime(HBV)-145805613-5prime(Human)	2	0.00	1	0.00	intron	'ZNF827	protein_coding
4	145,981,182	145,981,183	2,161	5prime(Human)-145981183-5prime(HBV)	2	1.00	.	.	gene	'Y_RNA	misc_RNA
4	146,680,677	146,680,678	1,812	3prime(HBV)-146680678-3prime(Human)	1	0.00	.	.	gene	'TTC29	protein_coding

4	146,680,677	146,680,678	1,814	3prime(HBV)-146680678-3prime(Human)	34	0.00	1	0.00	gene	'TTC29	protein_coding
4	148,058,486	148,058,487	1,825	3prime(HBV)-148058487-3prime(Human)	32	0.00	3	0.00	intron	'ARHGAP10	protein_coding
4	148,058,486	148,058,487	2,608	5prime(Human)-148058487-5prime(HBV)	21	0.00	1	0.00	intron	'ARHGAP10	protein_coding
4	153,165,779	153,165,780	1,800	3prime(HBV)-153165780-3prime(Human)	28	0.00	1	0.00	intron	'TRIM2	protein_coding
4	154,062,782	154,062,783	2,533	3prime(HBV)-154062783-3prime(Human)	1	1.00	.	.	intron	'AC079298.3	lncRNA
4	160,177,779	160,177,780	1,810	3prime(HBV)-160177780-5prime(Human)	8	1.00	1	1.00	gene	'AC093853.1	processed_pseudogene
4	160,177,863	160,177,864	1,810	3prime(HBV)-160177864-5prime(Human)	8	1.00	.	.	gene	'AC093853.1	processed_pseudogene
4	160,177,947	160,177,948	1,810	3prime(HBV)-160177948-5prime(Human)	10	1.00	.	.	gene	'AC093853.1	processed_pseudogene
4	160,178,031	160,178,032	1,810	3prime(HBV)-160178032-5prime(Human)	8	1.00	.	.	gene	'AC093853.1	processed_pseudogene
4	160,694,244	160,694,245	1,823	5prime(Human)-160694245-5prime(HBV)	104	0.01	4	0.00	gene	'LINC02477	lncRNA
4	161,077,464	161,077,465	1,785	3prime(HBV)-161077465-3prime(Human)	20	1.00	.	.	gene	'RPS14P7	processed_pseudogene
4	170,868,360	170,868,361	1,826	3prime(HBV)-170868361-5prime(Human)	137	0.01	4	0.00	gene	'RNU6ATAC13P	snRNA
4	171,990,545	171,990,546	1,814	3prime(HBV)-171990546-5prime(Human)	1	0.00	.	.	intron	'GALNTL6	protein_coding
4	171,990,547	171,990,548	1,814	3prime(HBV)-171990548-5prime(Human)	41	0.00	1	0.00	intron	'GALNTL6	protein_coding

4	173,150,658	173,150,659	1,847	3prime(Human)-173150659-5prime(HBV)	1	1.00	1	1.00	intron	'AC105285.1	lncRNA
4	179,865,753	179,865,754	1,809	3prime(HBV)-179865754-5prime(Human)	44	0.00	2	0.00	gene	'AC021193.1	processed_pseudogene
4	180,952,665	180,952,666	1,821	5prime(Human)-180952666-5prime(HBV)	14	0.00	1	0.00	gene	'LINC00290	lncRNA
4	187,454,485	187,454,486	1,794	3prime(HBV)-187454486-5prime(Human)	1	1.00	.	.	gene	'AC093763.2	processed_pseudogene
4	187,454,485	187,454,486	1,799	3prime(HBV)-187454486-5prime(Human)	49	0.29	2	0.50	gene	'AC093763.2	processed_pseudogene
4	188,721,559	188,721,560	2,422	3prime(Human)-188721560-5prime(HBV)	25	0.00	1	0.00	gene	'RNU7-192P	snRNA
4	189,939,923	189,939,924	1,812	3prime(HBV)-189939924-3prime(Human)	26	1.00	1	1.00	exon	'FRG1-DT	lncRNA
5	9,604,197	9,604,198	1,826	3prime(Human)-9604198-5prime(HBV)	28	0.00	2	0.00	gene	'AC026787.1	lncRNA
5	15,766,876	15,766,877	1,820	3prime(HBV)-15766877-5prime(Human)	31	0.00	1	0.00	intron	'FBXL7	protein_coding
5	15,766,897	15,766,898	1,817	3prime(Human)-15766898-5prime(HBV)	16	0.00	1	0.00	intron	'FBXL7	protein_coding
5	23,859,270	23,859,271	1,820	3prime(Human)-23859271-5prime(HBV)	20	0.00	1	0.00	gene	'C5orf17	lncRNA
5	23,859,271	23,859,272	1,820	3prime(Human)-23859272-5prime(HBV)	1	0.00	.	.	gene	'C5orf17	lncRNA
5	45,515,889	45,515,890	1,834	5prime(Human)-45515890-5prime(HBV)	23	0.00	1	0.00	intron	'HCN1	protein_coding
5	52,159,133	52,159,134	1,834	3prime(Human)-52159134-5prime(HBV)	2	1.00	.	.	gene	'LINC02118	lncRNA
5	57,017,467	57,017,468	2,533	3prime(HBV)-57017468-5prime(Human)	1	1.00	.	.	gene	'AC114973.1	processed_pseudogene
5	58,703,942	58,703,943	1,808	3prime(HBV)-58703943-3prime(Human)	67	0.00	2	0.00	intron	'RAB3C	protein_coding
5	63,379,363	63,379,364	1,827	3prime(HBV)-63379364-5prime(Human)	2	1.00	1	1.00	gene	'AC025445.1	processed_pseudogene
5	67,732,417	67,732,418	1,808	3prime(HBV)-67732418-5prime(Human)	34	0.00	1	0.00	intron	'AC106798.1	lncRNA
5	74,883,755	74,883,756	2,533	3prime(HBV)-74883756-5prime(Human)	1	1.00	.	.	gene	'AC010501.1	snoRNA
5	75,687,093	75,687,094	2,161	3prime(Human)-75687094-5prime(HBV)	3	1.00	.	.	intron	'POC5	protein_coding
5	77,354,174	77,354,175	1,818	3prime(HBV)-77354175-5prime(Human)	31	0.00	1	0.00	intron	'PDE8B	protein_coding

5	77,354,178	77,354,179	1,867	3prime(Human)-77354179-5prime(HBV)	62	0.00	1	0.00	intron	'PDE8B	protein_coding
5	79,672,328	79,672,329	2,333	3prime(Human)-79672329-5prime(HBV)	6	1.00	1	1.00	intron	'TENT2	protein_coding
5	80,994,645	80,994,646	1,818	3prime(Human)-80994646-5prime(HBV)	50	0.00	2	0.00	intron	'RASGRF2	protein_coding
5	83,656,239	83,656,240	1,813	3prime(HBV)-83656240-5prime(Human)	1	1.00	.	.	intron	'HAPLN1	protein_coding
5	83,801,699	83,801,700	2,011	5prime(Human)-83801700-5prime(HBV)	1	1.00	1	1.00	gene	'RNU4-11P	snRNA
5	84,160,873	84,160,874	1,790	3prime(HBV)-84160874-3prime(Human)	21	0.00	1	0.00	intron	'EDIL3	protein_coding
5	85,237,923	85,237,924	1,816	3prime(HBV)-85237924-3prime(Human)	42	0.00	1	0.00	intron	'AC117522.1	processed_pseudogene
5	85,390,081	85,390,082	1,819	5prime(Human)-85390082-5prime(HBV)	61	0.49	2	0.50	gene	'AC010486.1	lncRNA
5	89,277,520	89,277,521	1,863	5prime(Human)-89277521-5prime(HBV)	30	0.00	1	0.00	intron	'MEF2C-AS1	lncRNA
5	91,004,472	91,004,473	1,823	3prime(Human)-91004473-5prime(HBV)	47	0.00	1	0.00	intron	'ADGRV1	protein_coding
5	105,676,230	105,676,231	1,784	3prime(HBV)-105676231-3prime(Human)	28	0.00	1	0.00	gene	'RNA5SP189	rRNA_pseudogene
5	105,676,233	105,676,234	1,782	3prime(HBV)-105676234-3prime(Human)	1	0.00	.	.	gene	'RNA5SP189	rRNA_pseudogene
5	105,928,473	105,928,474	1,825	3prime(HBV)-105928474-5prime(Human)	33	0.00	1	0.00	gene	'RNA5SP189	rRNA_pseudogene
5	112,170,075	112,170,076	2,200	5prime(Human)-112170076-5prime(HBV)	24	0.00	1	0.00	intron	'EPB41L4A	protein_coding
5	118,486,413	118,486,414	1,826	3prime(HBV)-118486414-5prime(Human)	40	0.00	2	0.00	intron	'LINC02208	lncRNA
5	119,053,368	119,053,369	1,809	3prime(HBV)-119053369-3prime(Human)	1	1.00	.	.	intron	'AC008629.1	lncRNA
5	134,233,607	134,233,608	1,028	5prime(Human)-134233608-5prime(HBV)	1	0.00	.	.	intron	'AC104109.4	lncRNA
5	134,233,620	134,233,621	1,028	5prime(Human)-134233621-5prime(HBV)	8	0.00	1	0.00	intron	'AC104109.4	lncRNA
5	134,233,622	134,233,623	1,028	5prime(Human)-134233623-5prime(HBV)	12	0.00	.	.	intron	'AC104109.4	lncRNA

5	134,414,802	134,414,803	2,161	5prime(Human)-134414803-5prime(HBV)	3	1.00	.	.	gene	'CDKN2AIPNL	protein_coding
5	140,711,273	140,711,274	415	3prime(HBV)-140711274-3prime(Human)	15	0.00	1	0.00	gene	'VTRNA1-1	misc_RNA
5	150,286,202	150,286,203	3,118	3prime(HBV)-150286203-5prime(Human)	21	0.00	1	0.00	intron	'CAMK2A	protein_coding
5	155,504,478	155,504,479	1,427	3prime(HBV)-155504479-3prime(Human)	31	0.00	1	0.00	gene	'AC008725.1	processed_pseudogene
5	174,718,688	174,718,689	599	3prime(HBV)-174718689-3prime(Human)	28	0.00	1	0.00	gene	'MSX2	protein_coding
5	174,718,691	174,718,692	599	3prime(HBV)-174718692-3prime(Human)	1	0.00	.	.	gene	'MSX2	protein_coding
6	20,785,542	20,785,543	2,011	5prime(Human)-20785543-5prime(HBV)	1	1.00	.	.	intron	'CDKAL1	protein_coding
6	26,188,828	26,188,829	1,810	3prime(HBV)-26188829-5prime(Human)	42	0.00	2	0.00	CDS	'HIST1H4D	protein_coding
6	27,898,776	27,898,777	1,826	3prime(HBV)-27898777-5prime(Human)	29	0.00	1	0.00	gene	'RNU7-26P	snRNA
6	32,005,160	32,005,161	1,823	3prime(HBV)-32005161-3prime(Human)	9	1.00	1	1.00	gene	'CYP21A1P	retained_intron
6	32,005,163	32,005,164	1,828	5prime(Human)-32005164-5prime(HBV)	14	1.00	1	1.00	gene	'CYP21A1P	retained_intron
6	32,037,896	32,037,897	1,823	3prime(HBV)-32037897-3prime(Human)	6	1.00	1	1.00	gene	'CYP21A2	nonsense_mediated_decay
6	32,037,899	32,037,900	1,828	5prime(Human)-32037900-5prime(HBV)	16	1.00	1	1.00	gene	'CYP21A2	nonsense_mediated_decay
6	32,706,393	32,706,394	2,910	3prime(HBV)-32706394-5prime(Human)	2	1.00	.	.	exon	'MTCO3P1	unprocessed_pseudogene
6	36,347,479	36,347,480	2,984	3prime(HBV)-36347480-3prime(Human)	25	0.00	1	0.00	gene	'ETV7	protein_coding
6	39,121,959	39,121,960	2,533	3prime(HBV)-39121960-3prime(Human)	1	1.00	.	.	gene	'SAYSD1	protein_coding
6	42,467,340	42,467,341	2,360	3prime(HBV)-42467341-5prime(Human)	27	0.00	1	0.00	gene	'TRERF1	protein_coding
6	49,461,456	49,461,457	2,830	3prime(Human)-49461457-5prime(HBV)	41	0.00	2	0.00	intron	'MMUT	protein_coding
6	51,712,747	51,712,748	1,852	3prime(Human)-51712748-5prime(HBV)	59	0.00	3	0.00	intron	'PKHD1	protein_coding
6	52,446,851	52,446,852	1,819	3prime(Human)-52446852-5prime(HBV)	6	1.00	.	.	intron	'EFHC1	protein_coding
6	62,235,293	62,235,294	1,869	5prime(Human)-62235294-5prime(HBV)	29	0.00	1	0.00	intron	'KHDRBS2	protein_coding
6	62,235,408	62,235,409	1,825	3prime(HBV)-62235409-3prime(Human)	65	0.00	1	0.00	intron	'KHDRBS2	protein_coding

6	63,365,484	63,365,485	1,827	3prime(HBV)-63365485-5prime(Human)	1	1.00	1	1.00	gene	'AL121949.3	processed_pseudogene
6	63,955,570	63,955,571	2,533	3prime(HBV)-63955571-3prime(Human)	1	1.00	.	.	intron	'EYS	protein_coding
6	64,260,937	64,260,938	2,572	3prime(HBV)-64260938-3prime(Human)	35	1.00	1	1.00	intron	'EYS	protein_coding
6	66,310,634	66,310,635	2,534	3prime(HBV)-66310635-5prime(Human)	24	1.00	1	1.00	gene	'NUFIP1P1	processed_pseudogene
6	70,427,490	70,427,491	1,829	3prime(Human)-70427491-5prime(HBV)	1	1.00	.	.	intron	'FAM135A	protein_coding
6	71,359,605	71,359,606	1,819	3prime(Human)-71359606-5prime(HBV)	12	1.00	2	1.00	intron	'AL136164.2	lncRNA
6	72,511,212	72,511,213	2,689	3prime(Human)-72511213-5prime(HBV)	20	0.00	1	0.00	gene	'FO393414.2	processed_pseudogene
6	72,511,215	72,511,216	2,685	3prime(Human)-72511216-5prime(HBV)	1	0.00	.	.	gene	'FO393414.2	processed_pseudogene
6	80,120,257	80,120,258	1,845	3prime(Human)-80120258-5prime(HBV)	1	1.00	.	.	intron	'BCKDHB	protein_coding
6	80,238,996	80,238,997	1,845	3prime(Human)-80238997-5prime(HBV)	1	1.00	.	.	intron	'BCKDHB	protein_coding
6	81,873,146	81,873,147	1,827	3prime(HBV)-81873147-5prime(Human)	1	1.00	1	1.00	intron	'LINC02542	lncRNA
6	83,687,046	83,687,047	1,829	3prime(Human)-83687047-5prime(HBV)	27	0.00	1	0.00	intron	'SNAP91	protein_coding
6	85,104,324	85,104,325	1,816	3prime(HBV)-85104325-5prime(Human)	1	0.00	.	.	gene	'AL139806.1	unprocessed_pseudogene
6	85,104,327	85,104,328	1,816	3prime(HBV)-85104328-5prime(Human)	56	0.00	2	0.00	gene	'AL139806.1	unprocessed_pseudogene
6	88,221,928	88,221,929	1,823	3prime(Human)-88221929-5prime(HBV)	24	0.00	1	0.00	intron	'AL139042.1	lncRNA
6	88,221,932	88,221,933	1,823	3prime(Human)-88221933-5prime(HBV)	1	0.00	.	.	intron	'AL139042.1	lncRNA
6	107,828,698	107,828,699	1,798	3prime(HBV)-107828699-3prime(Human)	2	0.00	1	0.00	gene	'SCML4	protein_coding
6	110,838,300	110,838,301	1,813	3prime(HBV)-110838301-5prime(Human)	1	1.00	.	.	gene	'SNORA40C	snoRNA
6	111,082,230	111,082,231	1,841	5prime(Human)-111082231-5prime(HBV)	24	0.00	1	0.00	gene	'SLC16A10	protein_coding
6	114,191,446	114,191,447	1,834	3prime(Human)-114191447-5prime(HBV)	27	0.00	1	0.00	intron	'HS3ST5	protein_coding
6	115,346,983	115,346,984	2,042	3prime(HBV)-115346984-5prime(Human)	66	0.00	1	0.00	gene	'AL606845.1	processed_pseudogene
6	119,062,549	119,062,550	2,284	3prime(Human)-119062550-5prime(HBV)	28	0.00	1	0.00	intron	'FAM184A	protein_coding

6	121,012,090	121,012,091	1,845	5prime(Human)-121012091-5prime(HBV)	1	1.00	.	.	gene	'TBC1D32	protein_coding
6	123,266,230	123,266,231	1,829	3prime(Human)-123266231-5prime(HBV)	2	1.00	1	1.00	intron	'TRDN	protein_coding
6	123,266,230	123,266,231	1,831	3prime(Human)-123266231-5prime(HBV)	2	1.00	1	1.00	intron	'TRDN	protein_coding
6	134,201,123	134,201,124	2,161	3prime(Human)-134201124-5prime(HBV)	1	1.00	.	.	intron	'SGK1	protein_coding
6	139,793,673	139,793,674	2,333	3prime(Human)-139793674-5prime(HBV)	8	1.00	.	.	intron	'FILNC1	lncRNA
6	141,568,208	141,568,209	1,977	3prime(Human)-141568209-5prime(HBV)	1	1.00	1	1.00	gene	'RPS3AP23	lncRNA
6	148,558,565	148,558,566	2,011	5prime(Human)-148558566-5prime(HBV)	3	1.00	.	.	gene	'SASH1	protein_coding
6	153,659,529	153,659,530	1,995	3prime(Human)-153659530-5prime(HBV)	48	0.00	1	0.00	gene	'MTCO2P31	processed_pseudogene
6	159,018,690	159,018,691	1,875	3prime(Human)-159018691-5prime(HBV)	34	0.00	2	0.00	intron	'AL035530.2	lncRNA
6	160,060,819	160,060,820	334	3prime(HBV)-160060820-5prime(Human)	24	0.00	2	0.00	intron	'IGF2R	protein_coding
6	160,377,270	160,377,271	2,011	3prime(Human)-160377271-5prime(HBV)	1	1.00	1	1.00	intron	'SLC22A3	protein_coding
6	160,664,172	160,664,173	1,957	5prime(Human)-160664173-5prime(HBV)	18	1.00	1	1.00	CDS	'LPA	protein_coding
6	160,702,344	160,702,345	1,957	3prime(Human)-160702345-5prime(HBV)	14	1.00	1	1.00	CDS	'PLG	protein_coding
6	169,279,826	169,279,827	1,828	3prime(Human)-169279827-5prime(HBV)	1	1.00	1	1.00	gene	'BX322234.2	lncRNA

7	1,753,998	1,753,999	1,814	3prime(HBV)-1753999-3prime(Human)	91	0.11	2	0.00	gene	'ELFN1	protein_coding
7	1,754,001	1,754,002	1,814	3prime(HBV)-1754002-3prime(Human)	1	0.00	.	.	gene	'ELFN1	protein_coding
7	1,754,002	1,754,003	1,814	3prime(HBV)-1754003-3prime(Human)	1	0.00	.	.	gene	'ELFN1	protein_coding
7	2,686,818	2,686,819	1,813	3prime(HBV)-2686819-3prime(Human)	1	1.00	.	.	intron	'AMZ1	protein_coding
7	10,250,260	10,250,261	1,834	3prime(Human)-10250261-5prime(HBV)	3	1.00	.	.	gene	'AC004879.1	lncRNA
7	10,310,222	10,310,223	3,126	3prime(HBV)-10310223-3prime(Human)	31	0.00	2	0.00	gene	'AC004879.1	lncRNA
7	12,011,518	12,011,519	2,635	3prime(Human)-12011519-5prime(HBV)	19	0.00	1	0.00	gene	'THSD7A	protein_coding
7	12,011,559	12,011,560	2,554	3prime(HBV)-12011560-5prime(Human)	1	0.00	.	.	gene	'THSD7A	protein_coding
7	12,011,559	12,011,560	2,563	3prime(HBV)-12011560-5prime(Human)	194	0.00	7	0.00	gene	'THSD7A	protein_coding
7	25,174,787	25,174,788	2,406	3prime(Human)-25174788-5prime(HBV)	34	0.00	1	0.00	intron	'C7orf31	protein_coding
7	42,955,630	42,955,631	2,168	5prime(Human)-42955631-5prime(HBV)	22	0.00	3	0.00	intron	'AC005537.1	lncRNA
7	45,287,543	45,287,544	1,765	3prime(HBV)-45287544-5prime(Human)	28	0.00	1	0.00	intron	'AC073968.2	lncRNA
7	52,169,228	52,169,229	2,139	3prime(HBV)-52169229-5prime(Human)	1	0.00	.	.	intron	'AC079763.1	lncRNA
7	52,169,228	52,169,229	2,141	3prime(HBV)-52169229-5prime(Human)	29	0.00	1	0.00	intron	'AC079763.1	lncRNA
7	59,227,512	59,227,513	2,965	3prime(HBV)-59227513-5prime(Human)	6	1.00	1	1.00	gene	'AC023141.7	unprocessed_pseudogene
7	59,599,679	59,599,680	2,965	3prime(HBV)-59599680-5prime(Human)	7	1.00	1	1.00	gene	'AC023141.7	unprocessed_pseudogene
7	59,730,837	59,730,838	2,958	3prime(HBV)-59730838-5prime(Human)	1	1.00	.	.	gene	'AC023141.7	unprocessed_pseudogene
7	59,730,837	59,730,838	2,965	3prime(HBV)-59730838-5prime(Human)	5	1.00	1	1.00	gene	'AC023141.7	unprocessed_pseudogene
7	60,251,750	60,251,751	2,965	3prime(HBV)-60251751-5prime(Human)	8	1.00	1	1.00	gene	'AC128676.1	processed_pseudogene
7	69,455,910	69,455,911	2,011	3prime(Human)-69455911-5prime(HBV)	1	1.00	.	.	gene	'AC092100.1	lncRNA
7	73,919,770	73,919,771	1,832	5prime(Human)-73919771-5prime(HBV)	88	0.00	4	0.00	gene	'TMEM270	protein_coding
7	73,919,784	73,919,785	1,820	3prime(Human)-73919785-5prime(HBV)	58	0.31	3	0.33	gene	'TMEM270	protein_coding
7	81,001,027	81,001,028	2,161	5prime(Human)-81001028-5prime(HBV)	4	1.00	.	.	gene	'AC004972.1	TEC
7	81,427,380	81,427,381	1,807	3prime(HBV)-81427381-3prime(Human)	139	0.00	4	0.00	gene	'AC004866.2	unprocessed_pseudogene
7	81,427,385	81,427,386	1,807	3prime(HBV)-81427386-3prime(Human)	1	0.00	.	.	gene	'AC004866.2	unprocessed_pseudogene
7	81,431,028	81,431,029	1,816	5prime(Human)-81431029-5prime(HBV)	82	0.00	3	0.00	gene	'AC004866.2	unprocessed_pseudogene
7	83,903,083	83,903,084	1,824	3prime(HBV)-83903084-5prime(Human)	1	0.00	.	.	gene	'SEMA3A	protein_coding
7	83,903,085	83,903,086	1,821	3prime(HBV)-83903086-5prime(Human)	1	0.00	.	.	gene	'SEMA3A	protein_coding

7	83,903,085	83,903,086	1,824	3prime(HBV)-83903086-5prime(Human)	34	0.00	1	0.00	gene	'SEMA3A	protein_coding
7	83,903,091	83,903,092	1,835	3prime(Human)-83903092-5prime(HBV)	23	1.00	1	1.00	gene	'SEMA3A	protein_coding
7	88,669,403	88,669,404	1,847	5prime(Human)-88669404-5prime(HBV)	5	1.00	4	1.00	intron	'AC002069.2	lncRNA
7	91,710,011	91,710,012	1,845	3prime(Human)-91710012-5prime(HBV)	1	1.00	.	.	intron	'MTERF1	lncRNA
7	101,305,886	101,305,887	1,813	3prime(HBV)-101305887-5prime(Human)	25	1.00	1	1.00	gene	'AC006329.1	lncRNA
7	107,412,874	107,412,875	974	5prime(Human)-107412875-5prime(HBV)	26	0.00	1	0.00	intron	'COG5	protein_coding
7	110,816,888	110,816,889	1,977	5prime(Human)-110816889-5prime(HBV)	1	1.00	1	1.00	intron	'IMMP2L	protein_coding
7	120,457,241	120,457,242	1,821	5prime(Human)-120457242-5prime(HBV)	29	0.00	1	0.00	intron	'KCND2	protein_coding
7	120,457,339	120,457,340	1,818	3prime(HBV)-120457340-3prime(Human)	1	0.00	.	.	intron	'KCND2	protein_coding
7	125,419,762	125,419,763	1,824	3prime(HBV)-125419763-5prime(Human)	1	0.00	.	.	intron	'POT1-AS1	lncRNA
7	125,419,769	125,419,770	1,826	3prime(HBV)-125419770-5prime(Human)	83	0.01	4	0.00	intron	'POT1-AS1	lncRNA
7	125,419,782	125,419,783	1,819	3prime(HBV)-125419783-3prime(Human)	1	0.00	1	0.00	intron	'POT1-AS1	lncRNA
7	125,419,782	125,419,783	1,820	3prime(HBV)-125419783-3prime(Human)	206	0.00	7	0.00	intron	'POT1-AS1	lncRNA
7	125,419,785	125,419,786	1,820	3prime(HBV)-125419786-3prime(Human)	1	0.00	.	.	intron	'POT1-AS1	lncRNA
7	135,732,604	135,732,605	1,813	3prime(HBV)-135732605-5prime(Human)	1	1.00	.	.	intron	'FAM180A	protein_coding
7	151,525,960	151,525,961	1,809	3prime(HBV)-151525961-3prime(Human)	1	0.00	.	.	gene	'RHEB	protein_coding

7	152,622,094	152,622,095	1,823	3prime(HBV)-152622095-5prime(Human)	1	1.00	.	.	gene	'AC003109.1	snoRNA
7	155,329,554	155,329,555	1,117	3prime(HBV)-155329555-3prime(Human)	28	1.00	1	1.00	gene	'INSIG1	protein_coding
7	155,329,752	155,329,753	1,116	3prime(HBV)-155329753-3prime(Human)	1	1.00	.	.	gene	'INSIG1	protein_coding
8	8,207,893	8,207,894	1,818	5prime(Human)-8207894-5prime(HBV)	9	1.00	1	1.00	intron	'FAM85B	lncRNA
8	11,097,930	11,097,931	2,991	3prime(HBV)-11097931-3prime(Human)	28	0.00	1	0.00	intron	'XKR6	protein_coding
8	12,215,263	12,215,264	1,818	3prime(Human)-12215264-5prime(HBV)	11	1.00	1	1.00	gene	'ENPP7P12	processed_pseudogene
8	12,457,785	12,457,786	1,818	3prime(Human)-12457786-5prime(HBV)	12	1.00	1	1.00	intron	'ENPP7P6	transcribed_unprocessed_pseudogene
8	15,444,425	15,444,426	272	3prime(Human)-15444426-5prime(HBV)	12	0.00	1	0.00	intron	'TUSC3	lncRNA
8	23,066,849	23,066,850	2,161	5prime(Human)-23066850-5prime(HBV)	3	1.00	.	.	intron	'TNFRSF10B	protein_coding
8	26,461,716	26,461,717	1,825	3prime(Human)-26461717-5prime(HBV)	52	0.00	1	0.00	intron	'BNIP3L	protein_coding
8	38,508,999	38,509,000	1,829	3prime(Human)-38509000-5prime(HBV)	81	0.00	3	0.00	gene	'C8orf86	protein_coding
8	38,508,999	38,509,000	1,833	3prime(Human)-38509000-5prime(HBV)	1	0.00	.	.	gene	'C8orf86	protein_coding
8	40,115,326	40,115,327	1,788	3prime(HBV)-40115327-5prime(Human)	21	0.00	1	0.00	intron	'AC022733.1	lncRNA
8	42,090,829	42,090,830	2,161	3prime(Human)-42090830-5prime(HBV)	5	1.00	.	.	gene	'AC103724.1	processed_pseudogene
8	52,485,997	52,485,998	1,001	3prime(Human)-52485998-5prime(HBV)	10	0.00	1	0.00	gene	'ST18	retained_intron
8	65,046,046	65,046,047	2,028	3prime(Human)-65046047-5prime(HBV)	36	0.00	1	0.00	gene	'AC087808.1	processed_pseudogene
8	65,046,046	65,046,047	2,032	3prime(Human)-65046047-5prime(HBV)	1	0.00	.	.	gene	'AC087808.1	processed_pseudogene
8	65,046,049	65,046,050	2,029	3prime(Human)-65046050-5prime(HBV)	1	0.00	.	.	gene	'AC087808.1	processed_pseudogene
8	65,177,136	65,177,137	1,814	5prime(Human)-65177137-5prime(HBV)	49	0.00	2	0.00	intron	'LINC00251	lncRNA
8	72,151,369	72,151,370	1,977	5prime(Human)-72151370-5prime(HBV)	3	1.00	1	1.00	gene	'MSC-AS1	lncRNA
8	73,505,642	73,505,643	2,386	3prime(HBV)-73505643-3prime(Human)	33	0.00	3	0.00	intron	'STAU2	protein_coding
8	78,096,881	78,096,882	1,827	3prime(HBV)-78096882-3prime(Human)	25	0.00	1	0.00	gene	'AC084706.1	lncRNA
8	81,898,974	81,898,975	2,533	3prime(HBV)-81898975-5prime(Human)	1	1.00	.	.	intron	'LINC02235	lncRNA
8	90,047,474	90,047,475	1,834	3prime(Human)-90047475-5prime(HBV)	2	1.00	1	1.00	intron	'DECR1	protein_coding

8	90,495,621	90,495,622	2,161	3prime(Human)-90495622-5prime(HBV)	3	1.00	.	.	intron	'LINC00534	lncRNA
8	91,263,070	91,263,071	1,854	3prime(Human)-91263071-5prime(HBV)	25	0.00	1	0.00	intron	'SLC26A7	protein_coding
8	98,162,938	98,162,939	1,820	3prime(HBV)-98162939-3prime(Human)	16	0.00	1	0.00	gene	'POP1	protein_coding
8	104,096,255	104,096,256	1,801	3prime(HBV)-104096256-5prime(Human)	38	0.00	2	0.00	intron	'RIMS2	protein_coding
8	104,096,256	104,096,257	1,823	3prime(Human)-104096257-5prime(HBV)	23	0.00	1	0.00	intron	'RIMS2	protein_coding
8	106,309,814	106,309,815	1,821	3prime(Human)-106309815-5prime(HBV)	20	0.00	1	0.00	gene	'AC027031.2	lncRNA
8	108,486,138	108,486,139	1,918	3prime(Human)-108486139-5prime(HBV)	25	0.00	1	0.00	intron	'EMC2	protein_coding
8	108,486,142	108,486,143	1,918	3prime(Human)-108486143-5prime(HBV)	1	0.00	.	.	intron	'EMC2	protein_coding
8	112,170,916	112,170,917	1,756	3prime(HBV)-112170917-3prime(Human)	1	0.00	.	.	gene	'RNU4-37P	snRNA
8	112,170,917	112,170,918	1,757	3prime(HBV)-112170918-3prime(Human)	53	0.00	2	0.00	gene	'RNU4-37P	snRNA
8	113,168,508	113,168,509	1,847	3prime(Human)-113168509-5prime(HBV)	1	1.00	1	1.00	intron	'CSMD3	protein_coding
8	113,221,404	113,221,405	1,810	3prime(HBV)-113221405-5prime(Human)	1	0.00	.	.	intron	'CSMD3	protein_coding
8	113,221,408	113,221,409	1,810	3prime(HBV)-113221409-5prime(Human)	6	0.00	.	.	intron	'CSMD3	protein_coding
8	113,221,411	113,221,412	1,808	3prime(HBV)-113221412-5prime(Human)	1	0.00	.	.	intron	'CSMD3	protein_coding
8	113,221,412	113,221,413	1,810	3prime(HBV)-113221413-5prime(Human)	35	0.00	4	0.00	intron	'CSMD3	protein_coding
8	113,231,65	113,231,65	1,837	5prime(Human)-113231658-5prime(HBV)	102	0.01	3	0.00	intron	'CSMD3	protein_coding

	7	8									
8	113,231,657	113,231,658	1,838	5prime(Human)-113231658-5prime(HBV)	1	0.00	.	.	intron	'CSMD3	protein_coding
8	113,231,657	113,231,658	1,840	5prime(Human)-113231658-5prime(HBV)	1	0.00	.	.	intron	'CSMD3	protein_coding
8	116,287,581	116,287,582	1,843	5prime(Human)-116287582-5prime(HBV)	188	0.10	7	0.43	intron	'LINC00536	lncRNA
8	116,287,583	116,287,584	1,809	3prime(HBV)-116287584-3prime(Human)	62	0.00	2	0.00	intron	'LINC00536	lncRNA
8	118,245,407	118,245,408	1,794	3prime(HBV)-118245408-5prime(Human)	22	0.00	1	0.00	intron	'SAMD12	protein_coding
8	125,910,956	125,910,957	1,812	3prime(HBV)-125910957-3prime(Human)	1	1.00	.	.	intron	'LINC00861	lncRNA
8	131,509,657	131,509,658	1,802	3prime(Human)-131509658-5prime(HBV)	3	1.00	.	.	gene	'SNORA72	snoRNA
8	133,866,901	133,866,902	1,827	3prime(HBV)-133866902-5prime(Human)	2	1.00	1	1.00	gene	'AC110741.1	lncRNA
9	3,348,072	3,348,073	1,790	3prime(HBV)-3348073-3prime(Human)	40	0.00	1	0.00	intron	'RFX3	protein_coding
9	4,062,307	4,062,308	1,648	5prime(Human)-4062308-5prime(HBV)	16	1.00	1	1.00	intron	'GLIS3	protein_coding
9	8,045,328	8,045,329	1,834	5prime(Human)-8045329-5prime(HBV)	2	1.00	.	.	gene	'AL135923.2	lncRNA
9	8,839,731	8,839,732	2,447	3prime(HBV)-8839732-3prime(Human)	20	0.00	1	0.00	intron	'PTPRD	protein_coding
9	12,442,751	12,442,752	1,818	5prime(Human)-12442752-5prime(HBV)	35	0.00	1	0.00	gene	'RNU2-47P	snRNA
9	12,442,751	12,442,752	1,822	5prime(Human)-12442752-5prime(HBV)	1	0.00	.	.	gene	'RNU2-47P	snRNA
9	12,934,975	12,934,976	1,851	5prime(Human)-12934976-5prime(HBV)	1	0.00	1	0.00	gene	'AL161449.2	lncRNA
9	13,301,894	13,301,895	1,845	3prime(Human)-13301895-5prime(HBV)	1	1.00	.	.	gene	'AL162386.1	processed_pseudogene
9	16,492,896	16,492,897	1,826	3prime(HBV)-16492897-3prime(Human)	23	0.00	1	0.00	intron	'BNC2	protein_coding
9	17,541,662	17,541,663	1,977	3prime(Human)-17541663-5prime(HBV)	1	1.00	1	1.00	gene	'SH3GL2	protein_coding
9	23,544,924	23,544,925	1,802	5prime(Human)-23544925-5prime(HBV)	4	1.00	.	.	intron	'AL445623.2	lncRNA

9	27,733,953	27,733,954	2,940	3prime(HBV)-27733954-3prime(Human)	20	0.00	1	0.00	gene	'AL360014.1	lncRNA
9	30,556,315	30,556,316	1,942	5prime(Human)-30556316-5prime(HBV)	32	0.00	1	0.00	intron	'LINC01242	lncRNA
9	30,556,338	30,556,339	1,805	3prime(HBV)-30556339-3prime(Human)	53	0.00	2	0.00	intron	'LINC01242	lncRNA
9	39,809,623	39,809,624	1,826	3prime(HBV)-39809624-5prime(Human)	6	1.00	1	1.00	exon	'BX664615.1	processed_pseudogene
9	39,874,330	39,874,331	1,826	3prime(HBV)-39874331-3prime(Human)	7	1.00	1	1.00	CDS	'BX664615.2	protein_coding
9	40,991,403	40,991,404	1,812	3prime(HBV)-40991404-3prime(Human)	23	1.00	1	1.00	gene	'FRG1HP	lncRNA
9	41,042,635	41,042,636	623	3prime(HBV)-41042636-3prime(Human)	25	0.00	1	0.00	intron	'FRG1HP	transcribed_unprocessed_pseudogene
9	42,568,922	42,568,923	1,826	3prime(HBV)-42568923-5prime(Human)	9	1.00	1	1.00	exon	'BX088651.4	lncRNA
9	42,725,475	42,725,476	1,826	3prime(HBV)-42725476-3prime(Human)	4	1.00	1	1.00	intron	'BX664718.2	lncRNA
9	43,149,880	43,149,881	1,771	3prime(HBV)-43149881-5prime(Human)	1	0.00	1	0.00	gene	'FP325317.2	lncRNA
9	62,376,390	62,376,391	1,826	3prime(HBV)-62376391-5prime(Human)	5	1.00	1	1.00	exon	'FGF7P6	lncRNA
9	62,532,792	62,532,793	1,826	3prime(HBV)-62532793-3prime(Human)	7	1.00	1	1.00	exon	'BX005266.1	unprocessed_pseudogene
9	63,860,610	63,860,611	1,812	3prime(HBV)-63860611-5prime(Human)	26	1.00	.	.	gene	'FRG1JP	unprocessed_pseudogene
9	64,046,232	64,046,233	2,910	3prime(HBV)-64046233-5prime(Human)	3	1.00	.	.	gene	'U6	snRNA
9	72,928,027	72,928,028	2,023	5prime(Human)-72928028-5prime(HBV)	33	0.00	1	0.00	intron	'ALDH1A1	protein_coding
9	74,108,701	74,108,702	1,802	3prime(HBV)-74108702-3prime(Human)	22	0.00	1	0.00	gene	'AL513124.1	processed_pseudogene
9	78,166,562	78,166,563	859	3prime(HBV)-78166563-5prime(Human)	1	0.00	1	0.00	gene	'RN7SKP59	misc_RNA
9	83,925,153	83,925,154	1,809	3prime(HBV)-83925154-5prime(Human)	4	1.00	1	1.00	gene	'KIF27	protein_coding
9	93,705,951	93,705,952	1,817	3prime(Human)-93705952-5prime(HBV)	58	0.00	2	0.00	gene	'PHF2	protein_coding
9	101,441,241	101,441,242	1,798	3prime(HBV)-101441242-5prime(Human)	2	0.00	.	.	intron	'ALDOB	protein_coding
9	101,441,245	101,441,246	1,798	3prime(HBV)-101441246-5prime(Human)	137	0.00	5	0.00	intron	'ALDOB	protein_coding
9	106,292,651	106,292,652	2,325	3prime(Human)-106292652-5prime(HBV)	9	1.00	9	1.00	intron	'LINC01505	lncRNA
9	106,518,181	106,518,182	2,486	3prime(HBV)-106518182-3prime(Human)	29	0.00	1	0.00	intron	'LINC01505	lncRNA

9	113,677,227	113,677,228	1,100	3prime(Human)-113677228-5prime(HBV)	44	0.00	1	0.00	intron	'AL157702.2	lncRNA
9	114,069,518	114,069,519	1,783	3prime(HBV)-114069519-5prime(Human)	1	0.00	1	0.00	intron	'AMBP	protein_coding
9	114,069,567	114,069,568	1,844	3prime(Human)-114069568-5prime(HBV)	109	0.01	4	0.00	intron	'AMBP	protein_coding
9	114,069,567	114,069,568	1,847	3prime(Human)-114069568-5prime(HBV)	1	0.00	.	.	intron	'AMBP	protein_coding
9	118,881,535	118,881,536	1,824	3prime(HBV)-118881536-5prime(Human)	57	0.00	1	0.00	gene	'TUBB4BP6	processed_pseudogene
9	119,567,901	119,567,902	1,813	3prime(HBV)-119567902-5prime(Human)	2	1.00	.	.	gene	'AC006288.1	lncRNA
9	127,681,807	127,681,808	1,781	3prime(HBV)-127681808-5prime(Human)	20	0.00	1	0.00	intron	'STXBP1	protein_coding
9	132,411,736	132,411,737	1,687	3prime(Human)-132411737-5prime(HBV)	29	0.00	2	0.00	intron	'CFAP77	protein_coding
9	132,411,738	132,411,739	1,687	3prime(Human)-132411739-5prime(HBV)	1	0.00	.	.	intron	'CFAP77	protein_coding
9	136,074,421	136,074,422	1,802	5prime(Human)-136074422-5prime(HBV)	2	1.00	1	1.00	intron	'NACC2	protein_coding
9	136,810,645	136,810,646	1,813	3prime(HBV)-136810646-3prime(Human)	1	1.00	.	.	intron	'RABL6	protein_coding
10	9,186,977	9,186,978	1,961	3prime(HBV)-9186978-5prime(Human)	67	0.00	1	0.00	gene	'LINC00709	lncRNA
10	9,645,564	9,645,565	2,982	3prime(HBV)-9645565-5prime(Human)	29	0.00	1	0.00	gene	'HSP90AB7P	processed_pseudogene
10	11,976,262	11,976,263	1,805	3prime(HBV)-11976263-5prime(Human)	28	0.00	1	0.00	intron	'UPF2	protein_coding
10	15,484,734	15,484,735	2,161	3prime(Human)-15484735-5prime(HBV)	4	1.00	.	.	gene	'ITGA8	protein_coding
10	19,672,483	19,672,484	2,161	3prime(Human)-19672484-5prime(HBV)	2	1.00	.	.	intron	'MALRD1	protein_coding
10	21,712,561	21,712,562	1,565	3prime(HBV)-21712562-5prime(Human)	2	0.00	.	.	intron	'MLLT10	protein_coding

10	22,407,059	22,407,060	1,977	5prime(Human)-22407060-5prime(HBV)	1	1.00	1	1.00	intron	'SPAG6	protein_coding
10	29,689,515	29,689,516	2,011	5prime(Human)-29689516-5prime(HBV)	2	1.00	.	.	intron	'SVIL	protein_coding
10	35,402,600	35,402,601	2,533	3prime(HBV)-35402601-5prime(Human)	1	1.00	1	1.00	intron	'CCNY	protein_coding
10	36,104,247	36,104,248	3,066	3prime(HBV)-36104248-3prime(Human)	10	0.00	1	0.00	gene	'AL355300.1	lncRNA
10	38,931,683	38,931,684	1,806	3prime(HBV)-38931684-5prime(Human)	1	0.00	.	.	gene	'AL590623.1	processed_pseudogene
10	38,931,686	38,931,687	1,806	3prime(HBV)-38931687-5prime(Human)	31	0.00	1	0.00	gene	'AL590623.1	processed_pseudogene
10	66,530,758	66,530,759	1,807	3prime(HBV)-66530759-5prime(Human)	22	0.00	1	0.00	intron	'CTNNA3	protein_coding
10	68,683,341	68,683,342	2,161	3prime(Human)-68683342-5prime(HBV)	3	1.00	.	.	intron	'TET1	protein_coding
10	75,867,985	75,867,986	1,830	3prime(Human)-75867986-5prime(HBV)	29	0.00	1	0.00	intron	'LRMDA	protein_coding
10	85,147,945	85,147,946	1,845	5prime(Human)-85147946-5prime(HBV)	27	0.00	1	0.00	gene	'AL358787.2	processed_pseudogene
10	93,590,866	93,590,867	1,781	3prime(HBV)-93590867-5prime(Human)	13	0.00	1	0.00	intron	'FFAR4	protein_coding
10	99,136,296	99,136,297	930	5prime(Human)-99136297-5prime(HBV)	36	0.00	2	0.00	intron	'HPSE2	protein_coding
10	99,136,296	99,136,297	931	5prime(Human)-99136297-5prime(HBV)	1	0.00	.	.	intron	'HPSE2	protein_coding
10	100,161,707	100,161,708	2,370	5prime(Human)-100161708-5prime(HBV)	28	0.00	1	0.00	intron	'ERLIN1	protein_coding
10	100,611,317	100,611,318	2,161	5prime(Human)-100611318-5prime(HBV)	6	1.00	1	1.00	gene	'HIF1AN	protein_coding
10	101,255,779	101,255,780	1,809	3prime(HBV)-101255780-3prime(Human)	4	1.00	.	.	intron	'LBX1-AS1	lncRNA
10	102,299,755	102,299,756	1,771	3prime(HBV)-102299756-5prime(Human)	1	1.00	1	1.00	intron	'GBF1	protein_coding
10	105,732,828	105,732,829	2,533	3prime(HBV)-105732829-3prime(Human)	1	1.00	.	.	gene	'YWHAZP5	processed_pseudogene
10	122,307,226	122,307,227	3,095	3prime(HBV)-122307227-5prime(Human)	29	0.00	1	0.00	CDS	'BTBD16	protein_coding
10	133,542,237	133,542,238	1,825	5prime(Human)-133542238-5prime(HBV)	30	0.00	1	0.00	exon	'AL161645.2	lncRNA
10	133,542,24	133,542,24	1,816	3prime(HBV)-133542241-3prime(Human)	44	0.05	2	0.00	exon	'AL161645.2	lncRNA

	0	1									
11	6,350,035	6,350,036	2,558	3prime(HBV)-6350036-3prime(Human)	34	0.00	1	0.00	intron	'AC068733.3	lncRNA
11	7,204,184	7,204,185	1,822	3prime(HBV)-7204185-3prime(Human)	6	0.00	1	0.00	gene	'AC027804.1	lncRNA
11	13,476,909	13,476,910	1,823	3prime(HBV)-13476910-5prime(Human)	3	0.00	1	0.00	gene	'AC021269.1	processed_pseudogene
11	14,528,036	14,528,037	2,533	3prime(HBV)-14528037-3prime(Human)	3	1.00	.	.	intron	'PSMA1	protein_coding
11	16,444,553	16,444,554	1,803	3prime(HBV)-16444554-3prime(Human)	1	0.00	.	.	intron	'SOX6	protein_coding
11	16,444,553	16,444,554	1,808	3prime(HBV)-16444554-3prime(Human)	23	0.00	1	0.00	intron	'SOX6	protein_coding
11	16,747,320	16,747,321	2,001	3prime(HBV)-16747321-5prime(Human)	31	0.00	1	0.00	intron	'C11orf58	protein_coding
11	18,672,823	18,672,824	1,834	3prime(Human)-18672824-5prime(HBV)	6	1.00	.	.	gene	'AP006296.1	processed_pseudogene
11	18,923,571	18,923,572	1,834	3prime(Human)-18923572-5prime(HBV)	2	1.00	.	.	gene	'MRGPRX1	protein_coding
11	22,410,634	22,410,635	1,817	5prime(Human)-22410635-5prime(HBV)	29	0.00	1	0.00	gene	'SLC17A6	protein_coding
11	23,614,401	23,614,402	1,931	3prime(Human)-23614402-5prime(HBV)	49	0.00	2	0.00	gene	'AC068472.1	processed_pseudogene
11	23,614,402	23,614,403	1,935	3prime(Human)-23614403-5prime(HBV)	1	0.00	.	.	gene	'AC068472.1	processed_pseudogene
11	24,258,503	24,258,504	2,108	3prime(Human)-24258504-5prime(HBV)	91	0.00	3	0.00	intron	'LINC02686	lncRNA
11	31,216,207	31,216,208	1,916	5prime(Human)-31216208-5prime(HBV)	28	0.00	1	0.00	intron	'DCDC1	protein_coding
11	31,216,211	31,216,212	1,776	3prime(HBV)-31216212-3prime(Human)	23	0.00	1	0.00	intron	'DCDC1	protein_coding
11	35,544,020	35,544,021	1,823	3prime(HBV)-35544021-5prime(Human)	4	0.00	1	0.00	gene	'PAMR1	protein_coding
11	35,544,022	35,544,023	1,823	3prime(HBV)-35544023-5prime(Human)	1	1.00	.	.	gene	'PAMR1	protein_coding
11	35,544,026	35,544,027	1,823	3prime(HBV)-35544027-5prime(Human)	69	0.00	5	0.00	gene	'PAMR1	protein_coding
11	41,340,679	41,340,680	1,718	3prime(HBV)-41340680-3prime(Human)	25	1.00	1	1.00	intron	'LRRC4C	protein_coding
11	44,395,495	44,395,496	1,819	3prime(Human)-44395496-5prime(HBV)	24	1.00	1	1.00	gene	'AC010768.4	lncRNA
11	47,803,682	47,803,683	1,823	3prime(HBV)-47803683-5prime(Human)	1	0.00	.	.	intron	'NUP160	protein_coding
11	47,803,682	47,803,683	1,826	3prime(HBV)-47803683-5prime(Human)	49	0.00	2	0.00	intron	'NUP160	protein_coding
11	47,860,979	47,860,980	1,821	3prime(HBV)-47860980-3prime(Human)	45	0.00	3	0.00	gene	'NUP160	protein_coding
11	48,305,968	48,305,969	1,833	3prime(Human)-48305969-5prime(HBV)	1	0.00	.	.	gene	'OR4S1	protein_coding
11	49,600,512	49,600,513	2,211	5prime(Human)-49600513-5prime(HBV)	35	1.00	1	1.00	intron	'AC136759.1	lncRNA
11	49,600,567	49,600,568	1,785	3prime(HBV)-49600568-3prime(Human)	47	1.00	2	1.00	intron	'AC136759.1	lncRNA
11	63,254,416	63,254,417	1,827	3prime(HBV)-63254417-5prime(Human)	3	1.00	1	1.00	intron	'SLC22A10	lncRNA

11	78,028,514	78,028,515	1,813	3prime(HBV)-78028515-5prime(Human)	1	1.00	.	.	intron	'KCTD14	protein_coding
11	89,023,569	89,023,570	1,785	3prime(HBV)-89023570-5prime(Human)	38	1.00	2	1.00	intron	'GRM5	protein_coding
11	93,685,891	93,685,892	1,809	3prime(HBV)-93685892-3prime(Human)	3	1.00	.	.	intron	'CEP295	protein_coding
11	94,923,353	94,923,354	921	3prime(HBV)-94923354-3prime(Human)	1	0.00	.	.	intron	'AP002383.2	lncRNA
11	97,218,024	97,218,025	1,592	3prime(Human)-97218025-5prime(HBV)	50	0.00	1	0.00	gene	'LINC02553	lncRNA
11	97,286,006	97,286,007	1,768	3prime(Human)-97286007-5prime(HBV)	127	0.00	5	0.00	gene	'LINC02553	lncRNA
11	99,121,498	99,121,499	1,830	3prime(HBV)-99121499-5prime(Human)	1	0.00	.	.	intron	'CNTN5	protein_coding
11	99,121,499	99,121,500	1,828	3prime(HBV)-99121500-5prime(Human)	1	0.00	.	.	intron	'CNTN5	protein_coding
11	99,121,501	99,121,502	1,826	3prime(HBV)-99121502-5prime(Human)	1	0.00	.	.	intron	'CNTN5	protein_coding
11	99,121,501	99,121,502	1,829	3prime(HBV)-99121502-5prime(Human)	100	0.00	2	0.00	intron	'CNTN5	protein_coding
11	99,121,517	99,121,518	2,084	3prime(Human)-99121518-5prime(HBV)	10	0.00	1	0.00	intron	'CNTN5	protein_coding
11	111,608,904	111,608,905	1,817	3prime(Human)-111608905-5prime(HBV)	13	0.00	1	0.00	intron	'SIK2	protein_coding
11	111,790,337	111,790,338	1,825	3prime(HBV)-111790338-5prime(Human)	1	0.00	.	.	intron	'ALG9	protein_coding
11	111,790,340	111,790,341	1,825	3prime(HBV)-111790341-5prime(Human)	116	0.00	4	0.00	intron	'ALG9	protein_coding
11	111,790,365	111,790,366	3,105	3prime(Human)-111790366-5prime(HBV)	40	0.00	2	0.00	intron	'ALG9	protein_coding
11	113,591,213	113,591,214	1,814	3prime(HBV)-113591214-3prime(Human)	20	0.00	1	0.00	gene	'TMPRSS5	protein_coding
11	116,834,503	116,834,504	1,825	3prime(HBV)-116834504-3prime(Human)	28	0.00	1	0.00	gene	'APOA1	protein_coding
11	116,834,504	116,834,505	1,825	3prime(HBV)-116834505-3prime(Human)	1	0.00	.	.	gene	'APOA1	protein_coding
11	124,163,159	124,163,160	1,828	3prime(HBV)-124163160-5prime(Human)	66	0.00	3	0.00	gene	'OR10D1P	unprocessed_pseudogene
11	124,163,16	124,163,16	1,833	3prime(Human)-124163161-5prime(HBV)	126	0.00	4	0.00	gene	'OR10D1P	unprocessed_pseudogene

	0	1									
11	124,220,764	124,220,765	2,213	5prime(Human)-124220765-5prime(HBV)	20	0.00	1	0.00	gene	'OR8G2P	unprocessed_pseudogene
11	127,091,461	127,091,462	1,823	3prime(HBV)-127091462-5prime(Human)	1	1.00	1	1.00	intron	'AP001993.1	lncRNA
12	4,584,109	4,584,110	974	5prime(Human)-4584110-5prime(HBV)	27	0.00	1	0.00	intron	'DYRK4	protein_coding
12	10,913,216	10,913,217	1,398	5prime(Human)-10913217-5prime(HBV)	36	0.00	1	0.00	intron	'PRH1	protein_coding
12	19,856,378	19,856,379	131	3prime(HBV)-19856379-3prime(Human)	24	0.00	1	0.00	intron	'AC024901.1	lncRNA
12	19,990,939	19,990,940	1,796	5prime(Human)-19990940-5prime(HBV)	34	0.00	1	0.00	intron	'AC024901.1	lncRNA
12	20,692,252	20,692,253	1,805	3prime(HBV)-20692253-3prime(Human)	19	0.00	1	0.00	gene	'SLCO1C1	lncRNA
12	21,299,798	21,299,799	1,823	3prime(HBV)-21299799-5prime(Human)	23	0.04	1	0.00	intron	'SLCO1A2	protein_coding
12	23,348,614	23,348,615	1,828	3prime(Human)-23348615-5prime(HBV)	24	0.00	1	0.00	gene	'AC087235.1	lncRNA
12	23,348,614	23,348,615	1,831	3prime(Human)-23348615-5prime(HBV)	1	0.00	.	.	gene	'AC087235.1	lncRNA
12	29,351,779	29,351,780	2,359	5prime(Human)-29351780-5prime(HBV)	1	0.00	.	.	intron	'ERGIC2	protein_coding
12	29,351,780	29,351,781	2,359	5prime(Human)-29351781-5prime(HBV)	89	0.00	5	0.00	intron	'ERGIC2	protein_coding
12	29,351,780	29,351,781	2,360	5prime(Human)-29351781-5prime(HBV)	1	0.00	1	0.00	intron	'ERGIC2	protein_coding
12	29,351,791	29,351,792	1,828	3prime(HBV)-29351792-3prime(Human)	10	0.00	1	0.00	intron	'ERGIC2	protein_coding
12	30,713,631	30,713,632	1,827	3prime(HBV)-30713632-3prime(Human)	29	0.00	1	0.00	intron	'CAPRIN2	protein_coding
12	30,713,636	30,713,637	1,827	3prime(HBV)-30713637-3prime(Human)	1	1.00	.	.	intron	'CAPRIN2	protein_coding
12	32,346,668	32,346,669	1,823	3prime(HBV)-32346669-5prime(Human)	1	1.00	.	.	intron	'BICD1	protein_coding
12	38,725,921	38,725,922	383	3prime(HBV)-38725922-3prime(Human)	25	0.00	1	0.00	intron	'CPNE8	protein_coding
12	46,588,289	46,588,290	1,827	3prime(HBV)-46588290-3prime(Human)	1	1.00	1	1.00	intron	'AC008035.1	lncRNA
12	54,972,314	54,972,315	2,066	5prime(Human)-54972315-5prime(HBV)	13	0.00	2	0.00	intron	'TESPA1	protein_coding
12	57,933,056	57,933,057	1,827	5prime(Human)-57933057-5prime(HBV)	28	0.00	1	0.00	intron	'GIHCG	lncRNA
12	57,933,064	57,933,065	1,794	3prime(HBV)-57933065-3prime(Human)	62	0.00	2	0.00	intron	'GIHCG	lncRNA
12	58,723,888	58,723,889	1,816	3prime(Human)-58723889-5prime(HBV)	18	0.00	1	0.00	intron	'AC020637.1	lncRNA
12	59,604,574	59,604,575	1,817	5prime(Human)-59604575-5prime(HBV)	33	0.00	1	0.00	intron	'SLC16A7	protein_coding
12	62,519,291	62,519,292	2,004	3prime(HBV)-62519292-3prime(Human)	12	0.00	1	0.00	intron	'MON2	protein_coding

12	74,216,248	74,216,249	255	3prime(HBV)-74216249-5prime(Human)	24	0.00	1	0.00	intron	'AC090015.1	lncRNA
12	78,480,821	78,480,822	1,814	5prime(Human)-78480822-5prime(HBV)	103	0.00	5	0.00	intron	'AC079362.1	lncRNA
12	78,480,821	78,480,822	1,821	5prime(Human)-78480822-5prime(HBV)	1	0.00	.	.	intron	'AC079362.1	lncRNA
12	78,480,834	78,480,835	1,803	3prime(HBV)-78480835-3prime(Human)	1	0.00	.	.	intron	'AC079362.1	lncRNA
12	78,480,834	78,480,835	1,805	3prime(HBV)-78480835-3prime(Human)	115	0.00	3	0.00	intron	'AC079362.1	lncRNA
12	78,480,835	78,480,836	1,805	3prime(HBV)-78480836-3prime(Human)	1	0.00	.	.	intron	'AC079362.1	lncRNA
12	85,071,097	85,071,098	1,809	3prime(HBV)-85071098-5prime(Human)	1	0.00	.	.	intron	'LRRIQ1	protein_coding
12	85,071,097	85,071,098	1,811	3prime(HBV)-85071098-5prime(Human)	26	0.00	1	0.00	intron	'LRRIQ1	protein_coding
12	85,087,339	85,087,340	1,845	3prime(Human)-85087340-5prime(HBV)	1	1.00	1	1.00	intron	'LRRIQ1	protein_coding
12	85,140,100	85,140,101	2,282	5prime(Human)-85140101-5prime(HBV)	23	0.00	1	0.00	intron	'LRRIQ1	protein_coding
12	85,140,100	85,140,101	2,291	5prime(Human)-85140101-5prime(HBV)	1	0.00	.	.	intron	'LRRIQ1	protein_coding
12	95,875,422	95,875,423	1,828	5prime(Human)-95875423-5prime(HBV)	1	0.00	.	.	intron	'CCDC38	protein_coding
12	95,875,424	95,875,425	1,824	3prime(HBV)-95875425-3prime(Human)	1	0.00	.	.	intron	'CCDC38	protein_coding
12	95,875,424	95,875,425	1,827	3prime(HBV)-95875425-3prime(Human)	42	0.00	2	0.00	intron	'CCDC38	protein_coding
12	95,875,424	95,875,425	1,828	5prime(Human)-95875425-5prime(HBV)	19	0.00	1	0.00	intron	'CCDC38	protein_coding
12	95,875,427	95,875,428	1,827	3prime(HBV)-95875428-3prime(Human)	1	0.00	.	.	intron	'CCDC38	protein_coding
12	99,885,404	99,885,405	1,813	3prime(HBV)-99885405-3prime(Human)	1	1.00	.	.	intron	'ANKS1B	protein_coding
12	102,311,684	102,311,685	1,977	5prime(Human)-102311685-5prime(HBV)	2	1.00	1	1.00	exon	'HELLPAR	lncRNA
12	106,046,809	106,046,810	1,759	3prime(HBV)-106046810-3prime(Human)	1	0.00	.	.	gene	'AC011595.2	lncRNA
12	106,046,809	106,046,810	1,764	3prime(HBV)-106046810-3prime(Human)	33	0.00	1	0.00	gene	'AC011595.2	lncRNA
12	114,529,609	114,529,610	1,782	3prime(HBV)-114529610-3prime(Human)	29	0.00	1	0.00	gene	'AC069240.1	processed_pseudogene
12	117,195,864	117,195,865	2,326	3prime(Human)-117195865-5prime(HBV)	46	0.00	2	0.00	gene	'FBXO21	protein_coding
12	117,396,49	117,396,49	2,365	3prime(Human)-117396497-5prime(HBV)	11	0.00	1	0.00	intron	'NOS1	lncRNA

	6	7									
12	117,863,990	117,863,991	278	5prime(Human)-117863991-5prime(HBV)	16	0.00	1	0.00	intron	'KSR2	protein_coding
12	122,263,150	122,263,151	2,349	5prime(Human)-122263151-5prime(HBV)	132	0.00	5	0.00	intron	'VPS33A	protein_coding
12	122,263,150	122,263,151	2,354	5prime(Human)-122263151-5prime(HBV)	1	0.00	.	.	intron	'VPS33A	protein_coding
12	122,263,174	122,263,175	1,809	3prime(HBV)-122263175-3prime(Human)	92	0.26	4	0.25	intron	'VPS33A	protein_coding
12	122,263,177	122,263,178	1,810	3prime(HBV)-122263178-3prime(Human)	1	0.00	.	.	intron	'VPS33A	protein_coding
12	125,728,103	125,728,104	2,011	3prime(Human)-125728104-5prime(HBV)	4	1.00	.	.	gene	'TMEM132B	protein_coding
12	128,573,216	128,573,217	1,845	5prime(Human)-128573217-5prime(HBV)	1	1.00	.	.	intron	'TMEM132C	protein_coding
13	22,375,920	22,375,921	1,822	5prime(Human)-22375921-5prime(HBV)	21	0.00	1	0.00	gene	'AL136962.1	lncRNA
13	29,272,230	29,272,231	2,071	5prime(Human)-29272231-5prime(HBV)	31	0.00	2	0.00	intron	'MTUS2	protein_coding
13	29,272,240	29,272,241	274	3prime(HBV)-29272241-3prime(Human)	35	0.00	1	0.00	intron	'MTUS2	protein_coding
13	33,230,296	33,230,297	2,974	3prime(Human)-33230297-5prime(HBV)	22	0.00	1	0.00	intron	'STARD13	protein_coding
13	33,580,350	33,580,351	319	3prime(Human)-33580351-5prime(HBV)	19	0.00	1	0.00	intron	'AL139383.1	lncRNA
13	48,203,292	48,203,293	1,834	3prime(Human)-48203293-5prime(HBV)	4	1.00	.	.	gene	'ITM2B	protein_coding
13	54,735,364	54,735,365	1,815	3prime(HBV)-54735365-3prime(Human)	32	0.00	1	0.00	gene	'AL442636.1	lncRNA
13	57,107,835	57,107,836	1,804	3prime(HBV)-57107836-5prime(Human)	36	0.00	2	0.00	gene	'PRR20C	protein_coding
13	62,378,338	62,378,339	1,860	3prime(HBV)-62378339-3prime(Human)	45	0.00	1	0.00	gene	'AL356102.1	unprocessed_pseudogene
13	63,371,921	63,371,922	1,826	3prime(HBV)-63371922-3prime(Human)	24	0.00	1	0.00	gene	'AL359208.1	lncRNA
13	79,389,318	79,389,319	1,639	3prime(HBV)-79389319-3prime(Human)	71	0.00	2	0.00	intron	'RBM26	protein_coding
13	79,389,322	79,389,323	1,639	3prime(HBV)-79389323-3prime(Human)	1	0.00	.	.	intron	'RBM26	protein_coding
13	83,234,808	83,234,809	1,834	5prime(Human)-83234809-5prime(HBV)	24	0.00	1	0.00	gene	'RNU6-67P	snRNA

13	83,236,876	83,236,877	1,771	3prime(HBV)-83236877-3prime(Human)	26	0.00	1	0.00	gene	'RNU6-67P	snRNA
13	83,979,084	83,979,085	1,805	3prime(HBV)-83979085-5prime(Human)	23	0.00	1	0.00	gene	'AL590681.1	lncRNA
13	83,979,104	83,979,105	1,935	3prime(Human)-83979105-5prime(HBV)	112	0.00	3	0.00	gene	'AL590681.1	lncRNA
13	84,979,547	84,979,548	1,800	3prime(HBV)-84979548-5prime(Human)	24	0.00	1	0.00	gene	'AL356313.1	lncRNA
13	84,979,549	84,979,550	1,845	3prime(Human)-84979550-5prime(HBV)	134	0.18	5	0.20	gene	'AL356313.1	lncRNA
13	84,979,551	84,979,552	1,847	3prime(Human)-84979552-5prime(HBV)	1	0.00	.	.	gene	'AL356313.1	lncRNA
13	84,979,553	84,979,554	1,843	3prime(Human)-84979554-5prime(HBV)	1	0.00	.	.	gene	'AL356313.1	lncRNA
13	84,979,553	84,979,554	1,845	3prime(Human)-84979554-5prime(HBV)	1	0.00	.	.	gene	'AL356313.1	lncRNA
13	87,029,195	87,029,196	1,793	3prime(HBV)-87029196-3prime(Human)	16	0.00	1	0.00	gene	'LINC00430	lncRNA
13	87,106,601	87,106,602	1,817	3prime(Human)-87106602-5prime(HBV)	14	0.00	1	0.00	gene	'UBBP5	processed_pseudogene
13	95,782,199	95,782,200	1,845	3prime(Human)-95782200-5prime(HBV)	1	1.00	.	.	intron	'DNAJC3	protein_coding
13	101,259,765	101,259,766	1,823	3prime(HBV)-101259766-5prime(Human)	1	0.00	.	.	intron	'NALCN	protein_coding
13	104,110,855	104,110,856	1,817	3prime(Human)-104110856-5prime(HBV)	56	0.00	6	0.00	gene	'AL136524.1	lncRNA
13	106,023,441	106,023,442	1,789	3prime(HBV)-106023442-3prime(Human)	29	0.00	1	0.00	intron	'AL138701.2	lncRNA
13	109,502,704	109,502,705	1,845	3prime(Human)-109502705-5prime(HBV)	1	1.00	.	.	intron	'AL163541.1	lncRNA
13	109,762,655	109,762,656	1,874	5prime(Human)-109762656-5prime(HBV)	97	0.00	7	0.00	intron	'IRS2	protein_coding
13	109,762,663	109,762,664	1,800	3prime(HBV)-109762664-3prime(Human)	119	0.00	3	0.00	intron	'IRS2	protein_coding
13	109,762,666	109,762,667	1,795	3prime(HBV)-109762667-3prime(Human)	1	0.00	.	.	intron	'IRS2	protein_coding
14	28,829,816	28,829,817	1,813	3prime(HBV)-28829817-3prime(Human)	1	1.00	.	.	intron	'LINC02281	lncRNA
14	37,840,387	37,840,388	1,827	3prime(HBV)-37840388-5prime(Human)	1	0.00	1	0.00	intron	'TTC6	protein_coding
14	42,783,385	42,783,386	1,805	3prime(HBV)-42783386-3prime(Human)	1	0.00	.	.	gene	'AL356800.1	processed_pseudogene

14	42,783,385	42,783,386	1,809	3prime(HBV)-42783386-3prime(Human)	25	0.00	1	0.00	gene	'AL356800.1	processed_pseudogene
14	46,025,805	46,025,806	1,847	5prime(Human)-46025806-5prime(HBV)	1	1.00	.	.	intron	'LINC00871	lncRNA
14	46,025,808	46,025,809	1,845	5prime(Human)-46025809-5prime(HBV)	20	0.00	1	0.00	intron	'LINC00871	lncRNA
14	47,204,790	47,204,791	1,785	3prime(HBV)-47204791-3prime(Human)	49	0.00	1	0.00	intron	'MDGA2	protein_coding
14	49,130,061	49,130,062	1,819	3prime(Human)-49130062-5prime(HBV)	1	0.00	.	.	gene	'AL110505.1	lncRNA
14	68,792,458	68,792,459	1,791	3prime(HBV)-68792459-5prime(Human)	1	0.00	.	.	intron	'ZFP36L1	protein_coding
14	68,792,458	68,792,459	1,794	3prime(HBV)-68792459-5prime(Human)	1	0.00	.	.	intron	'ZFP36L1	protein_coding
14	68,792,458	68,792,459	1,799	3prime(HBV)-68792459-5prime(Human)	212	0.00	8	0.00	intron	'ZFP36L1	protein_coding
14	68,794,145	68,794,146	1,795	3prime(HBV)-68794146-3prime(Human)	212	0.00	7	0.00	exon	'ZFP36L1	protein_coding
14	74,571,166	74,571,167	1,806	3prime(HBV)-74571167-5prime(Human)	1	0.00	1	0.00	intron	'LTBP2	protein_coding
14	81,976,535	81,976,536	2,330	3prime(Human)-81976536-5prime(HBV)	1	0.00	1	0.00	intron	'AL355838.1	lncRNA
14	81,976,536	81,976,537	2,329	3prime(Human)-81976537-5prime(HBV)	24	0.00	1	0.00	intron	'AL355838.1	lncRNA
14	81,976,539	81,976,540	2,329	3prime(Human)-81976540-5prime(HBV)	1	0.00	.	.	intron	'AL355838.1	lncRNA
14	94,000,121	94,000,122	2,157	3prime(HBV)-94000122-3prime(Human)	7	0.00	1	0.00	intron	'CCDC197	protein_coding
14	96,562,323	96,562,324	1,818	3prime(HBV)-96562324-3prime(Human)	29	0.00	1	0.00	intron	'PAPOLA	protein_coding
14	103,388,486	103,388,487	1,823	3prime(HBV)-103388487-3prime(Human)	15	0.00	1	0.00	intron	'MARK3	protein_coding
14	106,150,391	106,150,392	1,826	3prime(HBV)-106150392-5prime(Human)	12	1.00	1	1.00	gene	'IGHV3-15	IG_V_gene
15	20,011,473	20,011,474	1,745	3prime(HBV)-20011474-5prime(Human)	13	1.00	1	1.00	gene	'IGHD1OR15-1A	IG_D_gene
15	20,573,516	20,573,517	1,819	3prime(Human)-20573517-5prime(HBV)	40	1.00	2	1.00	exon	'GOLGA8CP	unprocessed_pseudogene
15	21,018,120	21,018,121	1,745	3prime(HBV)-21018121-5prime(Human)	8	1.00	1	1.00	intron	'LINC01193	lncRNA
15	22,426,854	22,426,855	1,819	5prime(Human)-22426855-5prime(HBV)	1	1.00	.	.	gene	'GOLGA8DP	lncRNA
15	22,426,856	22,426,857	1,819	5prime(Human)-22426857-5prime(HBV)	48	1.00	3	1.00	gene	'GOLGA8DP	lncRNA
15	23,168,527	23,168,528	1,819	3prime(Human)-23168528-5prime(HBV)	61	1.00	3	1.00	exon	'GOLGA8EP	lncRNA
15	23,568,758	23,568,759	1,822	3prime(HBV)-23568759-3prime(Human)	162	0.00	5	0.00	intron	'MKRN3	protein_coding
15	24,072,619	24,072,620	1,362	3prime(HBV)-24072620-5prime(Human)	39	0.00	1	0.00	intron	'PWRN4	lncRNA

15	32,894,319	32,894,320	1,809	3prime(HBV)-32894320-5prime(Human)	1	0.00	.	.	intron	'FMN1	protein_coding
15	35,510,334	35,510,335	1,829	5prime(Human)-35510335-5prime(HBV)	29	0.00	1	0.00	intron	'DPH6	protein_coding
15	35,752,809	35,752,810	1,826	3prime(HBV)-35752810-3prime(Human)	32	0.00	1	0.00	intron	'DPH6-DT	lncRNA
15	35,752,811	35,752,812	1,826	3prime(HBV)-35752812-3prime(Human)	1	0.00	.	.	intron	'DPH6-DT	lncRNA
15	35,752,813	35,752,814	1,826	3prime(HBV)-35752814-3prime(Human)	1	0.00	.	.	intron	'DPH6-DT	lncRNA
15	43,857,179	43,857,180	1,810	3prime(HBV)-43857180-5prime(Human)	31	0.00	1	0.00	intron	'WDR76	protein_coding
15	44,345,314	44,345,315	1,817	3prime(Human)-44345315-5prime(HBV)	1	1.00	1	1.00	intron	'CASC4	protein_coding
15	50,427,016	50,427,017	2,161	3prime(Human)-50427017-5prime(HBV)	3	1.00	.	.	intron	'USP8	protein_coding
15	50,542,920	50,542,921	1,808	3prime(HBV)-50542921-3prime(Human)	62	0.00	2	0.00	intron	'USP50	protein_coding
15	64,195,138	64,195,139	1,999	3prime(HBV)-64195139-3prime(Human)	56	0.00	2	0.00	intron	'CSNK1G1	protein_coding
15	69,078,385	69,078,386	2,161	5prime(Human)-69078386-5prime(HBV)	4	1.00	.	.	intron	'EWSAT1	lncRNA
15	73,045,617	73,045,618	1,825	3prime(HBV)-73045618-5prime(Human)	1	0.00	.	.	gene	'NEO1	protein_coding
15	73,045,619	73,045,620	1,826	3prime(HBV)-73045620-5prime(Human)	32	0.00	1	0.00	gene	'NEO1	protein_coding
15	74,983,747	74,983,748	1,768	3prime(HBV)-74983748-3prime(Human)	33	0.00	1	0.00	intron	'SCAMP5	nonsense_mediated_decay
15	74,983,748	74,983,749	1,768	3prime(HBV)-74983749-3prime(Human)	1	0.00	.	.	intron	'SCAMP5	nonsense_mediated_decay
15	78,986,202	78,986,203	1,947	5prime(Human)-78986203-5prime(HBV)	72	0.00	2	0.00	intron	'RASGRF1	protein_coding
15	82,814,832	82,814,833	2,161	3prime(Human)-82814833-5prime(HBV)	5	1.00	.	.	intron	'WHAMM	protein_coding
15	87,128,743	87,128,744	1,797	3prime(HBV)-87128744-5prime(Human)	88	0.14	5	0.20	gene	'AC078905.1	lncRNA
15	87,128,744	87,128,745	1,796	3prime(HBV)-87128745-5prime(Human)	1	0.00	.	.	gene	'AC078905.1	lncRNA
15	87,128,778	87,128,779	1,841	3prime(Human)-87128779-5prime(HBV)	1	0.00	.	.	gene	'AC078905.1	lncRNA
15	87,128,781	87,128,782	1,838	3prime(Human)-87128782-5prime(HBV)	84	0.00	3	0.00	gene	'AC078905.1	lncRNA
15	87,128,791	87,128,792	1,838	3prime(Human)-87128792-5prime(HBV)	1	0.00	1	0.00	gene	'AC078905.1	lncRNA
15	87,128,800	87,128,801	1,922	3prime(HBV)-87128801-3prime(Human)	1	0.00	1	0.00	gene	'AC078905.1	lncRNA
15	93,973,837	93,973,838	1,827	3prime(HBV)-93973838-3prime(Human)	52	0.00	1	0.00	exon	'LINC01580	lncRNA
15	99,966,645	99,966,646	1,814	3prime(HBV)-99966646-3prime(Human)	17	1.00	1	1.00	gene	'AC084855.1	lncRNA
15	100,419,170	100,419,171	1,977	3prime(Human)-100419171-5prime(HBV)	1	1.00	1	1.00	intron	'CERS3	protein_coding
16	1,862,773	1,862,774	1,814	3prime(HBV)-1862774-5prime(Human)	13	1.00	1	1.00	intron	'MEIOB	protein_coding

16	4,252,721	4,252,722	1,519	3prime(Human)-4252722-5prime(HBV)	31	0.00	1	0.00	intron	'LINC01569	lncRNA
16	4,933,189	4,933,190	1,929	3prime(Human)-4933190-5prime(HBV)	26	0.00	1	0.00	intron	'PPL	protein_coding
16	5,400,158	5,400,159	1,829	5prime(Human)-5400159-5prime(HBV)	23	1.00	1	1.00	intron	'RBFOX1	protein_coding
16	14,545,694	14,545,695	1,823	3prime(HBV)-14545695-3prime(Human)	1	0.00	.	.	intron	'PARN	protein_coding
16	14,545,694	14,545,695	1,826	3prime(HBV)-14545695-3prime(Human)	263	0.00	9	0.00	intron	'PARN	protein_coding
16	14,545,698	14,545,699	1,826	3prime(HBV)-14545699-3prime(Human)	1	0.00	.	.	intron	'PARN	protein_coding
16	20,483,563	20,483,564	1,834	5prime(Human)-20483564-5prime(HBV)	5	1.00	.	.	intron	'ACSM2A	protein_coding
16	21,046,395	21,046,396	1,834	5prime(Human)-21046396-5prime(HBV)	5	1.00	.	.	intron	'DNAH3	protein_coding
16	32,029,067	32,029,068	1,826	3prime(HBV)-32029068-3prime(Human)	14	1.00	1	1.00	intron	'AC142381.4	lncRNA
16	32,972,629	32,972,630	1,822	3prime(HBV)-32972630-3prime(Human)	1	1.00	.	.	intron	'AC142086.5	lncRNA
16	32,972,629	32,972,630	1,826	3prime(HBV)-32972630-3prime(Human)	9	1.00	1	1.00	intron	'AC142086.5	lncRNA
16	33,881,322	33,881,323	1,826	3prime(HBV)-33881323-5prime(Human)	11	1.00	1	1.00	gene	'AC140658.3	unprocessed_pseudogene
16	49,111,215	49,111,216	3,012	5prime(Human)-49111216-5prime(HBV)	38	0.00	2	0.00	intron	'AC044798.3	lncRNA
16	54,537,596	54,537,597	1,847	5prime(Human)-54537597-5prime(HBV)	1	1.00	1	1.00	gene	'LINC02183	lncRNA
16	56,937,413	56,937,414	1,560	3prime(HBV)-56937414-3prime(Human)	25	0.00	1	0.00	intron	'HERPUD1	protein_coding
16	59,231,514	59,231,515	1,819	5prime(Human)-59231515-5prime(HBV)	19	1.00	.	.	gene	'AC106793.1	lncRNA
16	59,557,861	59,557,862	1,827	3prime(HBV)-59557862-5prime(Human)	34	0.00	1	0.00	gene	'RNU4-58P	snRNA
16	71,532,151	71,532,152	1,813	3prime(HBV)-71532152-3prime(Human)	1	1.00	.	.	intron	'CHST4	protein_coding
16	72,067,410	72,067,411	2,835	5prime(Human)-72067411-5prime(HBV)	59	0.00	1	0.00	intron	'HPR	protein_coding
16	72,919,820	72,919,821	1,847	5prime(Human)-72919821-5prime(HBV)	4	1.00	4	1.00	intron	'ZFHX3	protein_coding
16	81,899,307	81,899,308	1,823	3prime(HBV)-81899308-5prime(Human)	1	0.00	.	.	intron	'PLCG2	protein_coding
16	82,929,563	82,929,564	1,813	3prime(HBV)-82929564-5prime(Human)	1	1.00	.	.	intron	'CDH13	protein_coding
16	87,843,397	87,843,398	1,847	5prime(Human)-87843398-5prime(HBV)	7	1.00	6	1.00	intron	'SLC7A5	protein_coding
17	4,839,047	4,839,048	1,813	3prime(HBV)-4839048-3prime(Human)	1	1.00	.	.	intron	'MINK1	protein_coding
17	14,897,691	14,897,692	1,824	3prime(HBV)-14897692-3prime(Human)	1	0.00	.	.	intron	'LINC02096	lncRNA
17	14,897,691	14,897,692	1,826	3prime(HBV)-14897692-3prime(Human)	35	0.00	1	0.00	intron	'LINC02096	lncRNA
17	26,971,034	26,971,035	2,037	3prime(Human)-26971035-5prime(HBV)	1	0.00	.	.	gene	'AC069061.1	processed_pseudogene
17	26,971,036	26,971,037	2,034	3prime(Human)-26971037-5prime(HBV)	34	0.00	1	0.00	gene	'AC069061.1	processed_pseudogene

17	26,971,037	26,971,038	2,036	3prime(Human)-26971038-5prime(HBV)	1	0.00	.	.	gene	'AC069061.1	processed_pseudogene
17	27,507,166	27,507,167	1,619	3prime(HBV)-27507167-5prime(Human)	15	0.00	1	0.00	intron	'KSR1	protein_coding
17	30,586,051	30,586,052	2,349	5prime(Human)-30586052-5prime(HBV)	1	1.00	.	.	intron	'AC005562.1	lncRNA
17	32,533,247	32,533,248	408	3prime(Human)-32533248-5prime(HBV)	19	0.00	1	0.00	intron	'MYO1D	protein_coding
17	36,122,537	36,122,538	1,837	3prime(Human)-36122538-5prime(HBV)	30	1.00	1	1.00	intron	'AC243829.2	lncRNA
17	39,268,047	39,268,048	1,781	3prime(HBV)-39268048-5prime(Human)	1	0.00	.	.	intron	'FBXL20	protein_coding
17	39,268,050	39,268,051	1,781	3prime(HBV)-39268051-5prime(Human)	117	0.00	5	0.00	intron	'FBXL20	protein_coding
17	39,269,224	39,269,225	1,817	3prime(Human)-39269225-5prime(HBV)	127	0.00	5	0.00	intron	'FBXL20	protein_coding
17	39,269,224	39,269,225	1,820	3prime(Human)-39269225-5prime(HBV)	1	0.00	.	.	intron	'FBXL20	protein_coding
17	39,476,751	39,476,752	1,847	5prime(Human)-39476752-5prime(HBV)	1	1.00	1	1.00	intron	'CDK12	protein_coding
17	44,069,151	44,069,152	2,161	5prime(Human)-44069152-5prime(HBV)	4	1.00	.	.	gene	'LSM12	protein_coding
17	46,236,434	46,236,435	1,817	3prime(Human)-46236435-5prime(HBV)	105	0.18	4	0.25	gene	'MAPK8IP1P1	processed_pseudogene
17	46,236,434	46,236,435	1,822	3prime(Human)-46236435-5prime(HBV)	1	1.00	.	.	gene	'MAPK8IP1P1	processed_pseudogene
17	47,197,202	47,197,203	1,813	3prime(HBV)-47197203-3prime(Human)	2	1.00	.	.	gene	'MYL4	nonsense_mediated_decay
17	53,678,415	53,678,416	2,868	3prime(Human)-53678416-5prime(HBV)	31	0.00	1	0.00	intron	'AC034268.2	lncRNA
17	53,678,415	53,678,416	2,870	3prime(Human)-53678416-5prime(HBV)	1	0.00	.	.	intron	'AC034268.2	lncRNA
17	55,264,117	55,264,118	1,746	3prime(HBV)-55264118-3prime(Human)	20	0.00	1	0.00	gene	'HLF	protein_coding
17	83,245,057	83,245,058	1,824	3prime(HBV)-83245058-3prime(Human)	4	1.00	1	1.00	gene	'RPL23AP87	lncRNA
18	3,120,871	3,120,872	1,827	3prime(HBV)-3120872-3prime(Human)	46	0.00	2	0.00	intron	'MYOM1	protein_coding
18	28,916,189	28,916,190	1,778	3prime(HBV)-28916190-3prime(Human)	37	0.00	1	0.00	gene	'AC023932.1	processed_pseudogene
18	29,734,069	29,734,070	2,065	3prime(Human)-29734070-5prime(HBV)	20	0.00	2	0.00	gene	'AC117569.2	lncRNA
18	30,666,637	30,666,638	1,845	3prime(Human)-30666638-5prime(HBV)	18	0.00	1	0.00	gene	'AC090506.1	lncRNA
18	30,815,321	30,815,322	2,412	5prime(Human)-30815322-5prime(HBV)	31	0.00	1	0.00	gene	'AC090506.2	TEC
18	30,815,325	30,815,326	1,790	3prime(HBV)-30815326-3prime(Human)	45	0.00	2	0.00	gene	'AC090506.2	TEC
18	30,815,327	30,815,328	1,789	3prime(HBV)-30815328-3prime(Human)	1	0.00	.	.	gene	'AC090506.2	TEC
18	40,982,169	40,982,170	1,813	3prime(HBV)-40982170-5prime(Human)	1	1.00	.	.	gene	'AC079052.1	lncRNA
18	40,982,184	40,982,185	1,828	3prime(Human)-40982185-5prime(HBV)	119	0.00	2	0.00	gene	'AC079052.1	lncRNA
18	40,982,187	40,982,188	1,828	3prime(Human)-40982188-5prime(HBV)	1	0.00	.	.	gene	'AC079052.1	lncRNA

18	51,718,451	51,718,452	1,817	3prime(Human)-51718452-5prime(HBV)	35	0.00	1	0.00	gene	'AC027216.1	processed_pseudogene
18	58,450,314	58,450,315	1,676	3prime(HBV)-58450315-3prime(Human)	95	0.00	2	0.00	exon	'MIR122HG	lncRNA
18	58,452,115	58,452,116	1,825	3prime(HBV)-58452116-3prime(Human)	16	0.00	1	0.00	exon	'MIR122HG	lncRNA
18	65,022,790	65,022,791	1,813	3prime(HBV)-65022791-5prime(Human)	49	0.00	1	0.00	gene	'AC090348.1	lncRNA
18	68,189,087	68,189,088	1,819	3prime(Human)-68189088-5prime(HBV)	33	0.00	2	0.00	gene	'AC005909.2	lncRNA
18	68,189,087	68,189,088	1,820	3prime(Human)-68189088-5prime(HBV)	1	0.00	.	.	gene	'AC005909.2	lncRNA
19	1,507,822	1,507,823	1,776	3prime(HBV)-1507823-5prime(Human)	1	0.00	1	0.00	intron	'ADAMTSL5	protein_coding
19	1,507,825	1,507,826	1,776	3prime(HBV)-1507826-5prime(Human)	73	0.00	3	0.00	intron	'ADAMTSL5	protein_coding
19	3,883,159	3,883,160	2,011	3prime(Human)-3883160-5prime(HBV)	1	1.00	.	.	intron	'ATCAY	protein_coding
19	6,614,586	6,614,587	1,847	5prime(Human)-6614587-5prime(HBV)	1	1.00	.	.	gene	'CD70	protein_coding
19	12,402,348	12,402,349	1,534	3prime(HBV)-12402349-5prime(Human)	14	1.00	1	1.00	intron	'AC008758.5	protein_coding
19	12,442,152	12,442,153	1,534	3prime(HBV)-12442153-5prime(Human)	15	1.00	1	1.00	intron	'AC008758.6	nonsense_mediated_decay
19	23,490,059	23,490,060	2,333	5prime(Human)-23490060-5prime(HBV)	9	1.00	.	.	gene	'ZNF725P	unprocessed_pseudogene
19	29,823,082	29,823,083	1,834	3prime(Human)-29823083-5prime(HBV)	31	0.00	2	0.00	intron	'CCNE1	protein_coding
19	29,900,152	29,900,153	1,817	3prime(HBV)-29900153-5prime(Human)	14	1.00	1	1.00	gene	'AC008798.1	unprocessed_pseudogene
19	29,900,556	29,900,557	1,817	3prime(HBV)-29900557-5prime(Human)	13	1.00	1	1.00	gene	'AC008798.1	unprocessed_pseudogene
19	31,748,815	31,748,816	1,825	3prime(HBV)-31748816-5prime(Human)	1	0.00	.	.	gene	'RNU6-967P	snRNA
19	31,748,815	31,748,816	1,827	3prime(HBV)-31748816-5prime(Human)	25	0.00	1	0.00	gene	'RNU6-967P	snRNA
19	32,568,169	32,568,170	2,161	3prime(Human)-32568170-5prime(HBV)	2	1.00	.	.	gene	'PDCD5	protein_coding
19	33,367,597	33,367,598	2,161	3prime(Human)-33367598-5prime(HBV)	2	1.00	.	.	gene	'CEBPG	nonsense_mediated_decay
19	40,847,985	40,847,986	1,797	3prime(HBV)-40847986-5prime(Human)	49	0.35	2	0.50	intron	'CYP2A6	protein_coding
19	41,025,370	41,025,371	1,793	3prime(HBV)-41025371-5prime(Human)	1	1.00	.	.	intron	'CYP2A7P1	unprocessed_pseudogene
19	41,025,370	41,025,371	1,797	3prime(HBV)-41025371-5prime(Human)	20	1.00	1	1.00	intron	'CYP2A7P1	unprocessed_pseudogene
19	41,090,794	41,090,795	1,797	3prime(HBV)-41090795-3prime(Human)	25	1.00	1	1.00	intron	'CYP2A13	protein_coding
19	42,747,867	42,747,868	1,977	5prime(Human)-42747868-5prime(HBV)	2	1.00	1	1.00	gene	'PSG8	protein_coding
19	45,941,638	45,941,639	2,918	3prime(HBV)-45941639-3prime(Human)	16	0.00	1	0.00	intron	'NOVA2	protein_coding
19	47,912,315	47,912,316	1,802	3prime(HBV)-47912316-5prime(Human)	15	1.00	1	1.00	gene	'SULT2A1	protein_coding
19	47,928,083	47,928,084	1,802	3prime(HBV)-47928084-5prime(Human)	8	1.00	1	1.00	gene	'BSPH1	protein_coding

19	47,938,796	47,938,797	1,799	3prime(HBV)-47938797-5prime(Human)	1	1.00	.	.	gene	'BSPH1	protein_coding
19	47,938,796	47,938,797	1,802	3prime(HBV)-47938797-5prime(Human)	10	1.00	1	1.00	gene	'BSPH1	protein_coding
19	47,954,941	47,954,942	1,802	3prime(HBV)-47954942-5prime(Human)	9	1.00	1	1.00	gene	'BSPH1	protein_coding
19	53,832,239	53,832,240	1,826	3prime(HBV)-53832240-3prime(Human)	1	1.00	.	.	gene	'NLRP12	protein_coding
19	53,832,239	53,832,240	1,827	3prime(HBV)-53832240-3prime(Human)	9	1.00	.	.	gene	'NLRP12	protein_coding
19	56,120,525	56,120,526	1,826	3prime(HBV)-56120526-5prime(Human)	14	0.00	1	0.00	intron	'ZNF787	protein_coding
19	57,783,283	57,783,284	1,771	3prime(HBV)-57783284-5prime(Human)	24	0.00	1	0.00	intron	'ZNF586	protein_coding
19	58,603,280	58,603,281	1,824	3prime(HBV)-58603281-3prime(Human)	4	1.00	1	1.00	exon	'CENPBD1P1	lncRNA
20	3,976,073	3,976,074	1,817	3prime(HBV)-3976074-3prime(Human)	15	0.00	2	0.00	intron	'RNF24	protein_coding
20	5,250,899	5,250,900	1,815	3prime(HBV)-5250900-5prime(Human)	40	0.00	1	0.00	gene	'UBE2D3P1	processed_pseudogene
20	5,250,915	5,250,916	942	3prime(Human)-5250916-5prime(HBV)	22	0.00	1	0.00	gene	'UBE2D3P1	processed_pseudogene
20	10,414,761	10,414,762	1,852	5prime(Human)-10414762-5prime(HBV)	29	0.00	1	0.00	intron	'MKKS	protein_coding
20	13,506,820	13,506,821	1,837	3prime(Human)-13506821-5prime(HBV)	24	0.00	2	0.00	intron	'TASP1	protein_coding
20	13,506,823	13,506,824	1,837	3prime(Human)-13506824-5prime(HBV)	1	0.00	.	.	intron	'TASP1	protein_coding
20	18,311,954	18,311,955	1,821	3prime(HBV)-18311955-3prime(Human)	1	0.00	.	.	intron	'ZNF133	protein_coding
20	18,311,954	18,311,955	1,826	3prime(HBV)-18311955-3prime(Human)	21	0.00	.	.	intron	'ZNF133	protein_coding
20	24,893,588	24,893,589	1,360	5prime(Human)-24893589-5prime(HBV)	51	0.00	2	0.00	gene	'AL035661.1	lncRNA
20	28,603,781	28,603,782	1,812	3prime(HBV)-28603782-5prime(Human)	30	1.00	.	.	gene	'FRG1CP	lncRNA
20	30,746,561	30,746,562	2,910	3prime(HBV)-30746562-3prime(Human)	2	1.00	.	.	gene	'ANKRD20A2 1P	lncRNA
20	63,740,157	63,740,158	1,820	3prime(HBV)-63740158-5prime(Human)	62	0.00	2	0.00	intron	'SLC2A4RG	protein_coding
21	5,064,025	5,064,026	1,802	5prime(Human)-5064026-5prime(HBV)	1	1.00	.	.	gene	'FP565260.5	lncRNA
21	8,212,599	8,212,600	2,300	3prime(HBV)-8212600-3prime(Human)	7	1.00	1	1.00	exon	'RNA5-8SN2	rRNA
21	8,224,530	8,224,531	1,817	3prime(Human)-8224531-5prime(HBV)	9	1.00	1	1.00	intron	'FP671120.4	lncRNA
21	8,238,024	8,238,025	1,826	5prime(Human)-8238025-5prime(HBV)	8	1.00	.	.	gene	'FP671120.11	TEC
21	8,238,024	8,238,025	1,828	5prime(Human)-8238025-5prime(HBV)	1	1.00	.	.	gene	'FP671120.11	TEC
21	8,256,808	8,256,809	2,300	3prime(HBV)-8256809-3prime(Human)	9	1.00	1	1.00	exon	'5_8S_rRNA	rRNA
21	8,395,634	8,395,635	2,300	3prime(HBV)-8395635-3prime(Human)	9	1.00	1	1.00	exon	'RNA5-8SN3	rRNA

21	8,407,529	8,407,530	1,817	3prime(Human)-8407530-5prime(HBV)	11	1.00	1	1.00	intron	'FP236383.3	lncRNA
21	8,421,048	8,421,049	1,826	5prime(Human)-8421049-5prime(HBV)	9	1.00	1	1.00	intron	'FP236383.3	lncRNA
21	8,439,850	8,439,851	2,300	3prime(HBV)-8439851-3prime(Human)	5	1.00	1	1.00	exon	'RNA5-8SN1	rRNA
21	8,451,704	8,451,705	1,817	3prime(Human)-8451705-5prime(HBV)	12	1.00	1	1.00	intron	'FP236383.3	lncRNA
21	8,465,631	8,465,632	1,826	5prime(Human)-8465632-5prime(HBV)	10	1.00	1	1.00	gene	'FP236383.6	TEC
21	14,144,812	14,144,813	1,835	3prime(HBV)-14144813-5prime(Human)	38	0.00	1	0.00	intron	'LIPI	protein_coding
21	15,192,831	15,192,832	1,802	3prime(Human)-15192832-5prime(HBV)	3	1.00	.	.	gene	'AF127577.5	lncRNA
21	18,933,648	18,933,649	1,822	5prime(Human)-18933649-5prime(HBV)	19	0.00	1	0.00	intron	'AL157359.2	lncRNA
21	31,656,400	31,656,401	1,759	5prime(Human)-31656401-5prime(HBV)	1	0.00	.	.	intron	'AP000253.1	lncRNA
21	31,656,401	31,656,402	1,759	5prime(Human)-31656402-5prime(HBV)	1	0.00	.	.	intron	'AP000253.1	lncRNA
21	31,656,402	31,656,403	1,759	5prime(Human)-31656403-5prime(HBV)	27	0.00	1	0.00	intron	'AP000253.1	lncRNA
21	44,198,121	44,198,122	1,802	3prime(Human)-44198122-5prime(HBV)	2	1.00	.	.	intron	'GATD3A	protein_coding
21	46,694,882	46,694,883	1,824	3prime(HBV)-46694883-3prime(Human)	1	1.00	1	1.00	gene	'RPL23AP4	processed_pseudogene
22	12,137,520	12,137,521	2,910	3prime(HBV)-12137521-3prime(Human)	3	1.00	1	1.00	gene	'AC138776.1	lncRNA
22	12,601,337	12,601,338	1,814	5prime(Human)-12601338-5prime(HBV)	1	0.00	.	.	gene	'FRG1GP	unprocessed_pseudogene
22	12,601,340	12,601,341	1,814	5prime(Human)-12601341-5prime(HBV)	35	0.00	2	0.00	gene	'FRG1GP	unprocessed_pseudogene
22	12,601,341	12,601,342	1,810	5prime(Human)-12601342-5prime(HBV)	1	0.00	.	.	gene	'FRG1GP	unprocessed_pseudogene
22	12,601,346	12,601,347	1,812	3prime(HBV)-12601347-3prime(Human)	31	0.94	1	1.00	gene	'FRG1GP	unprocessed_pseudogene
22	22,160,637	22,160,638	2,325	3prime(Human)-22160638-5prime(HBV)	14	1.00	14	1.00	intron	'IGLV10-54	protein_coding
22	25,845,428	25,845,429	1,813	3prime(HBV)-25845429-5prime(Human)	1	1.00	.	.	intron	'MYO18B	protein_coding
22	39,092,393	39,092,394	827	3prime(HBV)-39092394-5prime(Human)	61	0.00	1	0.00	intron	'AL031846.1	lncRNA
22	40,557,705	40,557,706	1,747	3prime(HBV)-40557706-5prime(Human)	1	0.00	.	.	intron	'MRTFA	protein_coding
22	40,557,705	40,557,706	1,752	3prime(HBV)-40557706-5prime(Human)	54	0.00	2	0.00	intron	'MRTFA	protein_coding
22	43,174,226	43,174,227	1,840	5prime(Human)-43174227-5prime(HBV)	29	0.00	2	0.00	CDS	'TTLL12	protein_coding
22	43,174,226	43,174,227	1,844	5prime(Human)-43174227-5prime(HBV)	1	0.00	.	.	CDS	'TTLL12	protein_coding
22	47,932,581	47,932,582	2,011	3prime(Human)-47932582-5prime(HBV)	1	0.00	.	.	intron	'AL117329.1	lncRNA
22	50,762,540	50,762,541	1,813	3prime(HBV)-50762541-3prime(Human)	1	1.00	1	1.00	intron	'RPL23AP82	lncRNA
22	50,803,242	50,803,243	1,824	3prime(HBV)-50803243-3prime(Human)	4	1.00	2	1.00	gene	'RPL23AP82	lncRNA

X	4,535,030	4,535,031	2,314	5prime(Human)-4535031-5prime(HBV)	25	0.00	2	0.00	gene	'AC074035.1	lncRNA
X	4,535,030	4,535,031	2,317	5prime(Human)-4535031-5prime(HBV)	1	0.00	.	.	gene	'AC074035.1	lncRNA
X	28,387,521	28,387,522	1,808	3prime(HBV)-28387522-5prime(Human)	67	0.00	3	0.00	gene	'MIR6134	miRNA
X	28,387,541	28,387,542	1,922	3prime(Human)-28387542-5prime(HBV)	26	0.00	1	0.00	gene	'MIR6134	miRNA
X	30,677,736	30,677,737	1,813	3prime(HBV)-30677737-5prime(Human)	3	1.00	.	.	intron	'GK	protein_coding
X	32,076,436	32,076,437	1,829	5prime(Human)-32076437-5prime(HBV)	1	1.00	.	.	intron	'DMD	protein_coding
X	32,789,115	32,789,116	1,828	3prime(Human)-32789116-5prime(HBV)	55	0.00	3	0.00	intron	'DMD	protein_coding
X	32,789,116	32,789,117	1,828	3prime(Human)-32789117-5prime(HBV)	1	0.00	.	.	intron	'DMD	protein_coding
X	51,987,439	51,987,440	2,315	5prime(Human)-51987440-5prime(HBV)	1	0.00	1	0.00	gene	'TPMTP3	processed_pseudogene
X	55,789,334	55,789,335	2,533	3prime(HBV)-55789335-5prime(Human)	2	1.00	.	.	gene	'RRAGB	protein_coding
X	65,560,776	65,560,777	1,813	3prime(HBV)-65560777-3prime(Human)	1	0.00	.	.	gene	'FRMD8P1	processed_pseudogene
X	66,347,819	66,347,820	1,819	5prime(Human)-66347820-5prime(HBV)	8	1.00	.	.	gene	'AL157698.1	processed_pseudogene
X	71,714,366	71,714,367	1,791	3prime(Human)-71714367-5prime(HBV)	17	1.00	1	1.00	gene	'CXorf49	retained_intron
X	71,767,206	71,767,207	1,791	5prime(Human)-71767207-5prime(HBV)	16	1.00	1	1.00	gene	'CXorf49B	retained_intron
X	71,901,362	71,901,363	1,819	3prime(Human)-71901363-5prime(HBV)	1	0.00	.	.	gene	'NHSL2	protein_coding
X	75,856,999	75,857,000	2,533	3prime(HBV)-75857000-5prime(Human)	1	1.00	.	.	gene	'SAR1AP4	processed_pseudogene
X	92,148,811	92,148,812	1,823	3prime(HBV)-92148812-5prime(Human)	10	0.00	2	0.00	intron	'PCDH11X	protein_coding
X	95,648,801	95,648,802	1,745	3prime(HBV)-95648802-5prime(Human)	19	0.00	1	0.00	gene	'HNRNPDLP1	processed_pseudogene
X	98,237,459	98,237,460	1,827	3prime(HBV)-98237460-3prime(Human)	1	1.00	1	1.00	gene	'RPL6P29	processed_pseudogene
X	111,753,821	111,753,822	1,827	3prime(HBV)-111753822-5prime(Human)	1	1.00	1	1.00	intron	'ALG13	protein_coding
X	113,026,863	113,026,864	2,333	3prime(Human)-113026864-5prime(HBV)	1	1.00	.	.	intron	'AC002072.1	lncRNA
X	121,176,306	121,176,307	2,161	3prime(Human)-121176307-5prime(HBV)	3	1.00	.	.	intron	'AC002377.1	lncRNA
X	131,127,136	131,127,137	1,977	5prime(Human)-131127137-5prime(HBV)	2	1.00	1	1.00	gene	'RN7SL191P	misc_RNA
X	143,503,17	143,503,17	2,533	3prime(HBV)-143503173-5prime(Human)	4	1.00	.	.	intron	'AC239727.1	lncRNA

	2	3									
X	151,669,163	151,669,164	1,823	3prime(HBV)-151669164-3prime(Human)	1	0.00	.	.	intron	'PASD1	protein_coding
X	152,140,455	152,140,456	1,805	3prime(HBV)-152140456-5prime(Human)	41	0.00	2	0.00	intron	'AC116666.1	lncRNA
X	152,140,456	152,140,457	1,850	3prime(Human)-152140457-5prime(HBV)	78	0.00	3	0.00	intron	'AC116666.1	lncRNA
X	155,330,186	155,330,187	2,333	5prime(Human)-155330187-5prime(HBV)	6	1.00	.	.	intron	'CLIC2	protein_coding
X	155,330,186	155,330,187	2,338	5prime(Human)-155330187-5prime(HBV)	1	1.00	.	.	intron	'CLIC2	protein_coding
X	156,030,231	156,030,232	645	5prime(Human)-156030232-5prime(HBV)	38	0.00	1	0.00	gene	'DDX11L16	unprocessed_pseudogene
X	156,030,649	156,030,650	645	5prime(Human)-156030650-5prime(HBV)	1	1.00	1	1.00	gene	'DDX11L16	unprocessed_pseudogene
Y	5,941,945	5,941,946	1,827	3prime(HBV)-5941946-3prime(Human)	2	1.00	.	.	gene	'TUSC2P1	processed_pseudogene
Y	11,132,824	11,132,825	2,910	3prime(HBV)-11132825-5prime(Human)	6	1.00	.	.	gene	'AC134878.1	processed_pseudogene
Y	18,946,015	18,946,016	2,533	3prime(HBV)-18946016-5prime(Human)	1	1.00	.	.	intron	'TTTY14	lncRNA
Y	21,635,083	21,635,084	2,309	3prime(Human)-21635084-5prime(HBV)	33	0.00	1	0.00	gene	'CDY10P	unprocessed_pseudogene
Y	56,680,020	56,680,021	1,249	3prime(HBV)-56680021-3prime(Human)	1	1.00	1	1.00	gene	'CTBP2P1	processed_pseudogene
Y	56,694,300	56,694,301	1,249	3prime(HBV)-56694301-3prime(Human)	2	1.00	1	1.00	gene	'CTBP2P1	processed_pseudogene
Y	56,697,879	56,697,880	1,249	3prime(HBV)-56697880-3prime(Human)	3	1.00	1	1.00	gene	'CTBP2P1	processed_pseudogene
Y	56,705,042	56,705,043	1,249	3prime(HBV)-56705043-3prime(Human)	1	1.00	1	1.00	gene	'CTBP2P1	processed_pseudogene
Y	56,708,571	56,708,572	1,249	3prime(HBV)-56708572-3prime(Human)	1	1.00	1	1.00	gene	'CTBP2P1	processed_pseudogene
Y	56,715,699	56,715,700	1,249	3prime(HBV)-56715700-3prime(Human)	3	1.00	1	1.00	gene	'CTBP2P1	processed_pseudogene
Y	56,719,258	56,719,259	1,249	3prime(HBV)-56719259-3prime(Human)	2	1.00	1	1.00	gene	'CTBP2P1	processed_pseudogene
Y	56,722,832	56,722,833	1,249	3prime(HBV)-56722833-3prime(Human)	2	1.00	1	1.00	gene	'CTBP2P1	processed_pseudogene
Y	56,730,008	56,730,009	1,249	3prime(HBV)-56730009-3prime(Human)	4	1.00	1	1.00	gene	'CTBP2P1	processed_pseudogene

Y	56,733,582	56,733,583	1,249	3prime(HBV)-56733583-3prime(Human)	1	1.00	1	1.00	gene	'CTBP2P1	processed_pseudogene
Y	56,740,705	56,740,706	1,249	3prime(HBV)-56740706-3prime(Human)	3	1.00	1	1.00	gene	'CTBP2P1	processed_pseudogene
Y	56,758,461	56,758,462	1,249	3prime(HBV)-56758462-3prime(Human)	1	1.00	1	1.00	gene	'CTBP2P1	processed_pseudogene
Y	56,765,599	56,765,600	1,249	3prime(HBV)-56765600-3prime(Human)	1	1.00	1	1.00	gene	'CTBP2P1	processed_pseudogene
Y	56,769,172	56,769,173	1,249	3prime(HBV)-56769173-3prime(Human)	1	1.00	1	1.00	gene	'CTBP2P1	processed_pseudogene
