# Supplementary material for: Integration of Viral Genome to Human Genomic DNA in Nails of Patients with Chronic Hepatitis B Virus Infection
Source: JMA J. 2023 Sep 29;6(4):426–36. doi: 10.31662/jmaj.2023-0082 (PMC10628332; doi:10.31662/jmaj.2023-0082)
Supplement: Supplementary Table 9 [file 2433-3298-6-4-426-s012.pdf]

Supplementary Table 9. lg18807 HHV-7 integration breakpoints

Chrom	Start	End	Insert_Seq Breakpoint	Seqcode	# Junction Reads	Fraction of MQ0 Reads	# Junction Reads (Dedup)	Fraction of MQ0 Reads (Dedup)	Feature	Gene Name	Transcript Biotype
1	48,804,187	48,804,188	144,678	5prime(Human)-48804188-5prime(HHV)	1	0.00	1	0.00	intron	'AGBL4	protein_coding
5	73,571,363	73,571,364	139,115	5prime(Human)-73571364-5prime(HHV)	1	1.00	1	1.00	intron	'UTP15	protein_coding
5	73,571,363	73,571,364	68	5prime(Human)-73571364-5prime(HHV)	1	0.00	1	0.00	intron	'UTP15	protein_coding
14	18,573,060	18,573,061	5,734	3prime(HHV)-18573061-5prime(Human)	1	1.00	1	1.00	intron	'CR383656.10	lncRNA
17	80,061,876	80,061,877	5,688	5prime(Human)-80061877-5prime(HHV)	1	0.00	1	0.00	intron	'CCDC40	protein_coding
22	15,500,117	15,500,118	144,781	3prime(HHV)-15500118-5prime(Human)	1	1.00	1	1.00	gene	'YME1L1P1	unprocessed_pseudogene
22	15,500,117	15,500,118	5,734	3prime(HHV)-15500118-5prime(Human)	1	1.00	1	1.00	gene	'YME1L1P1	unprocessed_pseudogene
22	44,626,572	44,626,573	150	3prime(HHV)-44626573-5prime(Human)	1	1.00	1	1.00	gene	'LINC00229	lncRNA
MT	14,426	14,427	5,628	5prime(Human)-14427-5prime(HHV)	1	0.00	1	0.00	CDS	'MT-ND6	protein_coding
