# Supplementary material for: Integration of Viral Genome to Human Genomic DNA in Nails of Patients with Chronic Hepatitis B Virus Infection
Source: JMA J. 2023 Sep 29;6(4):426–36. doi: 10.31662/jmaj.2023-0082 (PMC10628332; doi:10.31662/jmaj.2023-0082)
Supplement: Supplementary Table 10 [file 2433-3298-6-4-426-s013.pdf]

Supplementary Table 10. Ig18207 HBV integration breakpoints

Chrom	Start	End	Insert_Seq Breakpoint	Seqcode	# Junction Reads	Fraction of MQ0 Reads	# Junction Reads (Dedup)	Fraction of MQ0 Reads (Dedup)	Feature	Gene Name	Trasncript Biotype
1	37,956,661	37,956,662	2,727	3prime(Human)-37956662-5prime(HBV)	1	1.00	.	.	gene	'SF3A3	protein_coding
1	72,990,394	72,990,395	3,033	3prime(HBV)-72990395-5prime(Human)	1	1.00	.	.	gene	'AL732618.1	TEC
1	81,248,589	81,248,590	3,033	3prime(HBV)-81248590-3prime(Human)	1	1.00	.	.	gene	'RN7SKP247	misc_RNA
1	91,366,636	91,366,637	3,082	3prime(HBV)-91366637-3prime(Human)	11	1.00	1	1.00	intron	'HFM1	protein_coding
1	186,937,211	186,937,212	1,094	5prime(Human)-186937212-5prime(HBV)	13	1.00	1	1.00	intron	'PLA2G4A	protein_coding
1	197,394,924	197,394,925	74	3prime(HBV)-197394925-5prime(Human)	34	0.00	1	0.00	intron	'CRB1	protein_coding
1	229,564,066	229,564,067	3,018	5prime(Human)-229564067-5prime(HBV)	1	0.00	.	.	gene	'ABCB10	protein_coding
1	229,564,070	229,564,071	3,018	5prime(Human)-229564071-5prime(HBV)	34	0.00	1	0.00	gene	'ABCB10	protein_coding
1	229,564,070	229,564,071	3,023	5prime(Human)-229564071-5prime(HBV)	1	0.00	.	.	gene	'ABCB10	protein_coding
2	25,251,880	25,251,881	2,023	3prime(HBV)-25251881-3prime(Human)	15	0.00	2	0.00	intron	'DNMT3A	protein_coding
2	25,251,883	25,251,884	2,023	3prime(HBV)-25251884-3prime(Human)	1	0.00	.	.	intron	'DNMT3A	protein_coding
2	37,252,888	37,252,889	459	3prime(HBV)-37252889-3prime(Human)	25	0.08	1	0.00	exon	'PRKD3	protein_coding
2	37,252,893	37,252,894	459	3prime(HBV)-37252894-3prime(Human)	1	0.00	.	.	exon	'PRKD3	protein_coding
2	40,954,174	40,954,175	2,636	3prime(Human)-40954175-5prime(HBV)	2	0.00	1	0.00	gene	'LINC01794	lncRNA
2	57,196,107	57,196,108	3,033	3prime(HBV)-57196108-3prime(Human)	1	1.00	1	1.00	gene	'AC132153.1	lncRNA
2	58,116,002	58,116,003	3,033	3prime(HBV)-58116003-5prime(Human)	1	1.00	.	.	intron	'VRK2	protein_coding
2	123,855,129	123,855,130	2,035	3prime(HBV)-123855130-3prime(Human)	1	0.00	.	.	gene	'AC064859.1	TEC
2	123,855,129	123,855,130	2,036	3prime(HBV)-123855130-3prime(Human)	26	0.00	1	0.00	gene	'AC064859.1	TEC
2	197,298,813	197,298,814	2,727	5prime(Human)-197298814-5prime(HBV)	1	0.00	.	.	intron	'ANKRD44	protein_coding
2	229,467,359	229,467,360	277	3prime(HBV)-229467360-3prime(Human)	31	0.00	2	0.00	intron	'DNER	protein_coding
3	26,162,953	26,162,954	3,033	3prime(HBV)-26162954-5prime(Human)	1	1.00	.	.	gene	'HMGB3P12	processed_pseudogene
3	27,620,824	27,620,825	1,429	3prime(Human)-27620825-5prime(HBV)	22	0.00	2	0.00	gene	'AC098614.1	processed_pseudogene
3	122,641,842	122,641,843	2,187	5prime(Human)-122641843-5prime(HBV)	1	0.00	1	0.00	gene	'PARP15	protein_coding

3	122,641,843	122,641,844	2,187	5prime(Human)-122641844-5prime(HBV)	1	0.00	.	.	gene	'PARP15	protein_coding
3	122,641,846	122,641,847	2,187	5prime(Human)-122641847-5prime(HBV)	3	0.00	.	.	gene	'PARP15	protein_coding
3	122,641,847	122,641,848	2,187	5prime(Human)-122641848-5prime(HBV)	3	0.00	.	.	gene	'PARP15	protein_coding
3	122,641,851	122,641,852	2,187	5prime(Human)-122641852-5prime(HBV)	2	0.00	.	.	gene	'PARP15	protein_coding
3	191,669,065	191,669,066	3,033	3prime(HBV)-191669066-5prime(Human)	1	1.00	.	.	gene	'Y_RNA	misc_RNA
4	12,712,384	12,712,385	2,759	5prime(Human)-12712385-5prime(HBV)	6	1.00	1	1.00	gene	'ECM1P2	processed_pseudogene
4	92,274,083	92,274,084	3,033	3prime(HBV)-92274084-5prime(Human)	1	1.00	.	.	intron	'LNCPRESS 2	lncRNA
4	92,946,155	92,946,156	1,905	5prime(Human)-92946156-5prime(HBV)	31	0.00	1	0.00	intron	'GRID2	protein_coding
4	94,295,813	94,295,814	3,033	3prime(HBV)-94295814-5prime(Human)	1	1.00	.	.	gene	'HPGDS	protein_coding
4	94,386,051	94,386,052	3,033	3prime(HBV)-94386052-5prime(Human)	1	1.00	.	.	gene	'AC109925.1	processed_pseudogene
4	128,649,683	128,649,684	1,871	3prime(HBV)-128649684-5prime(Human)	23	0.00	1	0.00	gene	'AC078850.2	lncRNA
4	137,637,347	137,637,348	705	3prime(Human)-137637348-5prime(HBV)	47	0.00	1	0.00	gene	'AC116563.1	lncRNA
4	137,637,347	137,637,348	708	3prime(Human)-137637348-5prime(HBV)	2	0.00	.	.	gene	'AC116563.1	lncRNA
4	137,637,355	137,637,356	705	3prime(Human)-137637356-5prime(HBV)	2	0.00	1	0.00	gene	'AC116563.1	lncRNA
4	138,931,012	138,931,013	3,033	3prime(HBV)-138931013-3prime(Human)	1	1.00	.	.	intron	'AC109927.1	lncRNA
4	182,817,667	182,817,668	3,033	3prime(HBV)-182817668-3prime(Human)	1	1.00	.	.	gene	'AC114798.1	lncRNA
5	44,013,798	44,013,799	3,082	3prime(HBV)-44013799-3prime(Human)	27	1.00	1	1.00	gene	'RNU6-381P	snRNA
5	67,047,626	67,047,627	1,799	5prime(Human)-67047627-5prime(HBV)	1	0.00	.	.	intron	'MAST4	protein_coding
5	67,047,629	67,047,630	1,799	5prime(Human)-67047630-5prime(HBV)	17	0.00	1	0.00	intron	'MAST4	protein_coding
5	76,360,503	76,360,504	3,033	3prime(HBV)-76360504-3prime(Human)	1	1.00	.	.	gene	'SV2C	protein_coding
5	83,326,648	83,326,649	401	3prime(HBV)-83326649-3prime(Human)	25	0.00	1	0.00	intron	'XRCC4	protein_coding
5	97,249,614	97,249,615	279	3prime(Human)-97249615-5prime(HBV)	35	0.00	2	0.00	intron	'LIX1-AS1	lncRNA
5	103,956,361	103,956,362	1,764	3prime(HBV)-103956362-3prime(Human)	24	0.00	.	.	gene	'AC008505.1	lncRNA
6	12,670,506	12,670,507	3,033	3prime(HBV)-12670507-3prime(Human)	1	1.00	.	.	gene	'PHACTR1	protein_coding
6	51,941,657	51,941,658	3,033	3prime(HBV)-51941658-3prime(Human)	1	1.00	.	.	intron	'PKHD1	protein_coding
6	72,714,913	72,714,914	1,985	3prime(Human)-72714914-5prime(HBV)	11	0.00	1	0.00	intron	'KCNQ5	protein_coding
6	72,714,917	72,714,918	1,989	3prime(Human)-72714918-5prime(HBV)	1	0.00	.	.	intron	'KCNQ5	protein_coding

6	75,536,157	75,536,158	1,820	3prime(Human)-75536158-5prime(HBV)	1	1.00	1	1.00	gene	'RPL26P20	processed_pseudogene
6	115,035,028	115,035,029	3,033	3prime(HBV)-115035029-3prime(Human)	1	1.00	.	.	gene	'AL590550.1	lncRNA
6	145,105,735	145,105,736	3,033	3prime(HBV)-145105736-5prime(Human)	1	1.00	.	.	gene	'AL023283.1	lncRNA
6	150,385,893	150,385,894	1,964	5prime(Human)-150385894-5prime(HBV)	28	0.00	1	0.00	intron	'IYD	protein_coding
6	150,385,893	150,385,894	1,967	5prime(Human)-150385894-5prime(HBV)	1	0.00	.	.	intron	'IYD	protein_coding
8	65,022,131	65,022,132	2,727	5prime(Human)-65022132-5prime(HBV)	1	0.00	.	.	gene	'AC087808.1	processed_pseudogene
8	68,976,319	68,976,320	3,033	3prime(HBV)-68976320-3prime(Human)	1	1.00	.	.	intron	'LINC01592	lncRNA
8	77,140,297	77,140,298	2,123	3prime(HBV)-77140298-3prime(Human)	1	1.00	1	1.00	gene	'HIGD1AP18	processed_pseudogene
8	85,651,583	85,651,584	2,445	3prime(HBV)-85651584-3prime(Human)	3	1.00	1	1.00	gene	'REXO1L3P	processed_pseudogene
8	85,718,939	85,718,940	2,445	3prime(HBV)-85718940-5prime(Human)	1	1.00	.	.	gene	'REXO1L12P	processed_pseudogene
8	85,759,806	85,759,807	2,445	3prime(HBV)-85759807-3prime(Human)	1	1.00	.	.	gene	'REXO1L9P	processed_pseudogene
8	85,772,000	85,772,001	2,445	3prime(HBV)-85772001-3prime(Human)	2	1.00	.	.	gene	'REXO1L2P	processed_pseudogene
8	85,787,275	85,787,276	2,445	3prime(HBV)-85787276-3prime(Human)	3	1.00	.	.	gene	'AC232323.1	processed_pseudogene
8	85,799,468	85,799,469	2,445	3prime(HBV)-85799469-3prime(Human)	2	1.00	.	.	gene	'REXO1L4P	processed_pseudogene
8	85,811,662	85,811,663	2,445	3prime(HBV)-85811663-3prime(Human)	1	1.00	.	.	gene	'REXO1L5P	processed_pseudogene
8	85,823,849	85,823,850	2,445	3prime(HBV)-85823850-3prime(Human)	2	1.00	.	.	gene	'REXO1L6P	lncRNA
8	89,410,201	89,410,202	963	3prime(HBV)-89410202-3prime(Human)	22	0.00	1	0.00	gene	'KRT8P4	processed_pseudogene
8	115,215,373	115,215,374	3,033	3prime(HBV)-115215374-3prime(Human)	1	1.00	.	.	gene	'TRPS1	protein_coding
8	117,459,649	117,459,650	887	3prime(HBV)-117459650-3prime(Human)	19	0.00	1	0.00	gene	'MED30	protein_coding
9	85,461,791	85,461,792	3,028	3prime(HBV)-85461792-3prime(Human)	1	1.00	.	.	gene	'AL583827.1	lncRNA
9	97,217,980	97,217,981	3,033	3prime(HBV)-97217981-3prime(Human)	1	1.00	.	.	intron	'ANKRD18CP	unprocessed_pseudogene
10	63,025,023	63,025,024	297	3prime(Human)-63025024-5prime(HBV)	14	0.00	1	0.00	gene	'RNU6-543P	snRNA
10	91,304,323	91,304,324	3,033	3prime(HBV)-91304324-5prime(Human)	1	1.00	.	.	gene	'PCGF5	protein_coding
11	20,457,398	20,457,399	393	3prime(HBV)-20457399-5prime(Human)	30	0.00	1	0.00	intron	'PRMT3	protein_coding
11	30,876,290	30,876,291	3,212	3prime(HBV)-30876291-3prime(Human)	1	0.00	1	0.00	intron	'DCDC1	protein_coding
12	51,925,115	51,925,116	389	5prime(Human)-51925116-5prime(HBV)	1	0.00	1	0.00	gene	'ACVRL1	lncRNA

13	42,869,999	42,870,000	3,033	3prime(HBV)-42870000-5prime(Human)	1	1.00	.	.	gene	'EPSTI1	protein_coding
13	64,073,357	64,073,358	717	3prime(HBV)-64073358-3prime(Human)	22	0.00	1	0.00	intron	'LINC00355	lncRNA
13	64,073,358	64,073,359	704	3prime(HBV)-64073359-3prime(Human)	1	1.00	.	.	intron	'LINC00355	lncRNA
14	38,193,942	38,193,943	3,033	3prime(HBV)-38193943-3prime(Human)	1	1.00	.	.	intron	'AL392023.2	lncRNA
14	96,321,588	96,321,589	3,161	3prime(HBV)-96321589-5prime(Human)	1	0.00	.	.	intron	'ATG2B	protein_coding
14	96,321,592	96,321,593	3,156	3prime(HBV)-96321593-5prime(Human)	1	0.00	.	.	intron	'ATG2B	protein_coding
14	96,321,592	96,321,593	3,161	3prime(HBV)-96321593-5prime(Human)	30	0.00	1	0.00	intron	'ATG2B	protein_coding
14	98,988,346	98,988,347	3,033	3prime(HBV)-98988347-3prime(Human)	1	1.00	.	.	gene	'AL162151.3	lncRNA
16	51,975,012	51,975,013	3,093	3prime(HBV)-51975013-5prime(Human)	28	0.00	1	0.00	gene	'C16orf97	lncRNA
16	65,277,834	65,277,835	3,033	3prime(HBV)-65277835-5prime(Human)	1	1.00	.	.	intron	'AC009055.1	lncRNA
17	12,963,681	12,963,682	705	5prime(Human)-12963682-5prime(HBV)	27	1.00	1	1.00	intron	'ARHGAP44	protein_coding
17	44,543,785	44,543,786	1,967	3prime(HBV)-44543786-5prime(Human)	39	0.00	1	0.00	gene	'FZD2	protein_coding
17	52,457,871	52,457,872	2,467	5prime(Human)-52457872-5prime(HBV)	27	0.00	1	0.00	intron	'LINC01982	lncRNA
17	69,213,115	69,213,116	149	3prime(HBV)-69213116-5prime(Human)	25	0.00	1	0.00	intron	'ABCA10	protein_coding
17	76,067,714	76,067,715	2,759	3prime(Human)-76067715-5prime(HBV)	4	1.00	.	.	intron	'SRP68	protein_coding
17	76,991,031	76,991,032	1,273	5prime(Human)-76991032-5prime(HBV)	25	0.00	1	0.00	gene	'AC016168.3	TEC
18	17,244,377	17,244,378	1,938	3prime(HBV)-17244378-5prime(Human)	4	1.00	.	.	gene	'AP005901.1	transcribed_unprocessed_pseudogene
18	17,610,948	17,610,949	1,938	3prime(HBV)-17610949-5prime(Human)	3	1.00	.	.	gene	'AP005901.1	transcribed_unprocessed_pseudogene
18	17,638,760	17,638,761	1,938	3prime(HBV)-17638761-5prime(Human)	3	1.00	.	.	gene	'AP005901.1	transcribed_unprocessed_pseudogene
18	19,676,459	19,676,460	1,938	3prime(HBV)-19676460-5prime(Human)	3	1.00	.	.	gene	'ROCK1	protein_coding
18	19,699,851	19,699,852	1,938	3prime(HBV)-19699852-5prime(Human)	4	1.00	1	1.00	gene	'ROCK1	protein_coding
18	20,246,233	20,246,234	1,938	3prime(HBV)-20246234-5prime(Human)	4	1.00	.	.	gene	'ROCK1	protein_coding
18	26,582,033	26,582,034	2,768	5prime(Human)-26582034-5prime(HBV)	1	1.00	.	.	intron	'KCTD1	protein_coding
18	29,629,322	29,629,323	27	3prime(Human)-29629323-5prime(HBV)	26	0.00	2	0.00	gene	'AC117569.2	lncRNA
18	51,215,981	51,215,982	2,759	3prime(Human)-51215982-5prime(HBV)	2	1.00	.	.	intron	'MEX3C	lncRNA
18	51,572,616	51,572,617	3,033	3prime(HBV)-51572617-5prime(Human)	1	1.00	.	.	intron	'LINC01630	lncRNA
19	17,596,578	17,596,579	3,033	3prime(HBV)-17596579-3prime(Human)	1	1.00	.	.	gene	'UNC13A	protein_coding
19	20,158,685	20,158,686	3,033	3prime(HBV)-20158686-3prime(Human)	1	1.00	.	.	intron	'AC011447.3	lncRNA

19	36,076,324	36,076,325	2,759	5prime(Human)-36076325-5prime(HBV)	5	1.00	.	.	intron	'WDR62	protein_coding
20	29,563,508	29,563,509	2,759	5prime(Human)-29563509-5prime(HBV)	8	1.00	1	1.00	gene	'FAM242B	lncRNA
20	30,320,950	30,320,951	2,759	3prime(Human)-30320951-5prime(HBV)	3	1.00	.	.	gene	'FAM242A	lncRNA
21	9,039,828	9,039,829	1,825	3prime(Human)-9039829-5prime(HBV)	24	0.00	1	0.00	gene	'CR392039.3	processed_pseudogene
21	9,118,916	9,118,917	1,085	3prime(Human)-9118917-5prime(HBV)	8	1.00	.	.	intron	'TEKT4P2	lncRNA
21	9,787,766	9,787,767	2,643	3prime(HBV)-9787767-3prime(Human)	24	0.00	1	0.00	intron	'LINC01667	lncRNA
22	12,798,752	12,798,753	1,085	3prime(Human)-12798753-5prime(HBV)	9	1.00	.	.	gene	'FRG1GP	unprocessed_pseudogene
22	31,031,093	31,031,094	2,727	3prime(Human)-31031094-5prime(HBV)	26	0.00	2	0.00	gene	'AC005005.4	lncRNA
X	101,366,104	101,366,105	2,123	3prime(HBV)-101366105-3prime(Human)	20	0.00	1	0.00	intron	'BTK	protein_coding
Y	11,204,217	11,204,218	1,085	3prime(Human)-11204218-5prime(HBV)	13	1.00	1	1.00	intron	'AC134878.2	lncRNA
