# Supplementary material for: Integration of Viral Genome to Human Genomic DNA in Nails of Patients with Chronic Hepatitis B Virus Infection
Source: JMA J. 2023 Sep 29;6(4):426–36. doi: 10.31662/jmaj.2023-0082 (PMC10628332; doi:10.31662/jmaj.2023-0082)
Supplement: Supplementary Table 11 [file 2433-3298-6-4-426-s014.pdf]

Supplementary Table 11. Ig18207 HHV-7 integration breakpoints

Chrom	Start	End	Insert_Seq Breakpoint	Seqcode	# Junction Reads	Fraction of MQ0 Reads	# Junction Reads (Dedup)	Fraction of MQ0 Reads (Dedup)	Feature	Gene Name	Trasncript Biotype
4	40,983,878	40,983,879	28,017	3prime(Human)-40983879-5prime(HHV)	38	0.00	1	0.00	intron	'APBB2	protein_coding
5	49,520	49,521	5,603	3prime(Human)-49521-5prime(HHV)	1	1.00	.	.	gene	'AC113430.1	processed_pseudogene
7	153,676,002	153,676,003	117,412	5prime(Human)-153676003-5prime(HHV)	4	0.00	1	0.00	gene	'AC005998.1	lncRNA
9	104,407,448	104,407,449	142,420	3prime(HHV)-104407449-3prime(Human)	11	0.00	1	0.00	gene	'AL512646.1	processed_pseudogene
9	104,407,448	104,407,449	3,373	3prime(HHV)-104407449-3prime(Human)	12	0.00	1	0.00	gene	'AL512646.1	processed_pseudogene
12	107,555,399	107,555,400	33,934	3prime(HHV)-107555400-5prime(Human)	1	0.00	.	.	intron	'BTBD11	protein_coding
12	107,555,401	107,555,402	33,934	3prime(HHV)-107555402-5prime(Human)	24	0.00	1	0.00	intron	'BTBD11	protein_coding
19	53,455,783	53,455,784	98,353	3prime(HHV)-53455784-3prime(Human)	23	0.04	1	0.00	CDS	'ZNF761	protein_coding
