# Supplementary material for: Integration of Viral Genome to Human Genomic DNA in Nails of Patients with Chronic Hepatitis B Virus Infection
Source: JMA J. 2023 Sep 29;6(4):426–36. doi: 10.31662/jmaj.2023-0082 (PMC10628332; doi:10.31662/jmaj.2023-0082)
Supplement: Supplementary Table 12 [file 2433-3298-6-4-426-s015.pdf]

Supplementary Table 12. PCR primers for the confirmation of integration points

**Ig18206**		
GN215	Ig18206, HBV, 62,606,503 chrom 1+ HBV, forward	TTGTGAAAGCAAAATGAAGGTCAA
GN216	Ig18206, HBV, 62,606,503 chrom 1+ HBV, reverse	GAGGAGTTGGGGGAGGAGATT
GN217	Ig18206, HBV, 215,418,187 chrom 2+ HBV,forward	ATGGGAAGATTGTAGCTGATTACC
GN218	Ig18206, HBV, 215,418,187 chrom 2+ HBV,reverse	AACTCCGCAGAAGCTCCAAA
GN219	Ig18206, HBV, 68,792,458 HBV+chrom14, forward	GAGGACTCTTGGACTTTCAGCA
GN220	Ig18206, HBV, 68,792,458 HBV+chrom14, reverse	TTGGCCTTTCTTCCTCGCAT
GN221	Ig18206, HBV, 14,545,694 HBV+chrom16, forward	AGCCAGTGATTGGTAAGAGGTG
GN222	Ig18206, HBV, 14,545,694 HBV+chrom16, reverse	TGTGTGTTTACTGAGTGGGAGG
GN237	Ig18206 HBV, Chrom 4: 180,952,665 forward	CTCCATCCTGATGCCAAAGTGAGCA
GN238	Ig18206 HBV, Chrom 4: 180,952,665 reverse	GAGCAGAGGCGGTGTCGAGG
GN239	Ig18206 HBV, Chrom 4: 189,939,923 forward	TGCACGTCGCATGGAGACCA
GN240	Ig18206 HBV, Chrom 4: 189,939,923 reverse	CACCCCACTCAAGCTGTGGCT
GN241	Ig18206 HBV, Chrom 12: 95,875,424 forward	AAAGACTGGGAGGAGTTGGGGGA
GN242	Ig18206 HBV, Chrom 12: 95,875,424 reverse	GTGAAATGTGGGGTGGGGAAGG
GN243	Ig18206 HBV, Chrom 6: 107,828,698 forward	CGTGAACGCCCACCAGGTCT
GN244	Ig18206 HBV, Chrom 6: 107,828,698 reverse	ATCTGGCCCCATGAAAGGCAGG
GN245	Ig18206 HBV, Chrom 8: 15,444,425 forward	GTTCGTCATCTTCCACCATCCCCAG
GN246	Ig18206 HBV, Chrom 8: 15,444,425 reverse	TGGAAGTAGAGGACAAACGGGCAAC
GN247	Ig18206 HBV, Chrom 1: 85,585,553 forward	AAGGTCTTTACTGTGCATGGGAAGGT
GN248	Ig18206 HBV, Chrom 1: 85,585,553 reverse	TGGTGAGGTGAACAATGTTCCGGAG
GN249	Ig18206 HBV, Chrom 20: 3,976,073 forward	GACTCTCAGCAATGTCAACGACCGA
GN250	Ig18206 HBV, Chrom 20: 3,976,073 revserse	TTCCTAGGGCTGCCTTAACAACGTG
**Ig18807**		
GN577	Ig18807, HBV, Chrom 18: 59,672,521 outer forward	CATCCTTTCCATGGCTGCTAG
GN578	Ig18807, HBV, Chrom 18: 59,672,521 outer revserse	ATTAGAGAAGGTTTGGCTTGGTTC

GN579	Ig18807, HBV, Chrom 18: 59,672,521 inner forward	CGCGGGACGTCCTTTGTTTA
GN580	Ig18807, HBV, Chrom 18: 59,672,521 inner reverse	AGAGGGCCTCACTCTTGTGT
GN581	Ig18807, HBV, Chrom 10: 114,442,363 outer forward	TCGGTGGTTTACAAATCTTTAGAGT
GN582	Ig18807, HBV, Chrom 10: 114,442,363 outer revserse	GAAGGAAAGAAGTCAGAAGGCAAAA
GN583	Ig18807, HBV, Chrom 10: 114,442,363 inner forward	TGTATAACATTGTACCTGCATAGATGA
GN584	Ig18807, HBV, Chrom 10: 114,442,363 inner reverse	GAAGCGCCAAATTCTTTATACGG
GN585	Ig18807, HHV-7, Chrom 5: 73,571,363 HHV-7, 139115 outer forward	AACCCCAGAAATTTAGCTCATACTC
GN586	Ig18807, HHV-7, Chrom 5: 73,571,363 HHV-7, 139115 outer revserse	GGGCAGATATGGATAGGATCTAGAA
GN587	Ig18807, HHV-7, Chrom 5: 73,571,363 HHV-7, 139115 inner forward	CAGCAGCTTTTGAAAATCAGTTATT
GN588	Ig18807, HHV-7, Chrom 5: 73,571,363 HHV-7, 139115 nner reverse	GTTAGGGTGACAGTGAGAGCTAG
GN589	Ig18807, HHV-7, Chrom 17: 80,061,876 outer forward	CTACAGAGTTGGCAAAGATAAGGAG
GN590	Ig18807, HHV-7, Chrom 17: 80,061,876 outer revserse	TTATGGGACTAAAGCCAAAATTGTG
GN591	Ig18807, HHV-7, Chrom 17: 80,061,876 inner forward	GAGAGATGAGCCCTCCCTGC
GN592	Ig18807, HHV-7, Chrom 17: 80,061,876 inner reverse	GTCTTTCTCGTGCACAAACTTG
**Ig18207,HBV**		
GN1	Ig18207, HBV chrom 17: 44,543,785 outer forward	TGTTCAAGCCTCCAAGCTGT
GN2	Ig18207, HBV chrom 17: 44,543,785 outer reverse	GGAGGAGGGGCACTAAGGTA
GN3	Ig18207, HBV chrom 17: 44,543,785 inner forward	CCTCCAAGCTGTGCCTTG
GN4	Ig18207, HBV chrom 17: 44,543,785 inner reverse	AAGGTATGCACAACCAGATGC
GN29	Ig18207, HBV, chrom 1:197,394,924 outer forward	CAATCGGCAGTCAGGGAGAC
GN30	Ig18207, HBV, chrom 1:197,394,924 outer reverse	GGATTAGCTGCCAGTCCCTA
GN31	Ig18207, HBV, chrom 1:197,394,924 inner forward	GACAGTCATCCTCAGGCCAT
GN32	Ig18207, HBV, chrom 1:197,394,924 inner revsrse	AGCAGGGAGTCTAAACCATTCA
GN33	Ig18207, HBV, chrom 2: 37,252,888 outer forward	AGTCCCCAACCTCCAATCAC
GN34	Ig18207, HBV, chrom 2: 37,252,888 outer revverse	AGTAGGTACAGGCGGGAAAC
GN35	Ig18207, HBV, chrom 2: 37,252,888 inner forward	TGTCCTGGCTATCGCTGGAT
GN36	Ig18207, HBV, chrom 2: 37,252,889 inner revsrse	GGTACAGGCGGGAAACTGAATAA
GN37	Ig18207, HBV, chrom 2: 123,855,129 outer forward	GCATGGACATTGACCCGTAT

GN38	Ig18207, HBV, chrom 2: 123,855,129 outer revverse	CCCCACCTATTCTTTGCTTTTCA
GN39	Ig18207, HBV, chrom 2: 123,855,129 inner forward	TGGAGCTTCTGTGGAGTTACT
GN40	Ig18207, HBV, chrom 2: 123,855,129 inner revsrse	TTTCAGGTTGTTTATTGTCAGCA
GN41	Ig18207, HBV, chrom 4: 92 946,155 outer forward	ACTGCCTTTAATCTCACCGTT
GN42	Ig18207, HBV, chrom 4: 92,946,155 outer revverse	TCAACTCACCCCAACACAGA
GN43	Ig18207, HBV, chrom 4: 92,946,155 inner forward	CCTTTAATCTCACCGTTTAGGTCT
GN44	Ig18207, HBV, chrom 4: 92,946,155 inner revsrse	AGACTCTAAGGCCTCCCGAT
GN45	Ig18207, HBV, chrom 1: 229,564,071 outer forward	GTGATGAGGAAGGCAGAGGT
GN46	Ig18207, HBV, chrom 1: 229,564,071 outer reverse	AGAGGTGGAGAGATGGGAGT
GN47	Ig18207, HBV, chrom 1: 229,564,071 inner forward	GCAAATCACCAGAGGCTACG
GN48	Ig18207, HBV, chrom 1: 229,564,071 inner reverse	TCTCCCTGACTGCCGATTG
GN49	Ig18207, HBV, chrom 4: 137,637,347 outer forward	ACAACTCGCTGTCACATTAGC
GN50	Ig18207, HBV, chrom 4: 137,637,347 outer reverse	GTGGTAATGTTCCCCAACTTCC
GN51	Ig18207, HBV, chrom 4: 137,637,347 inner forward	CGCTGTCACATTAGCAATTATACC
GN52	Ig18207, HBV, chrom 4: 137,637,347 inner reverse	GTTGTACAGACTTGGCCCCC
GN53	Ig18207, HBV, chrom 5: 83,326,648 outer forward	CACGTGTCCTGGCCAAAATTC
GN54	Ig18207, HBV, chrom 5: 83,326,648 outer reverse	TGCTAGTGTCAAATTGTCAAGGGA
GN55	Ig18207, HBV, chrom 5: 83,326,648 inner forward	AAAATTCGCGGTCCCCAACC
GN56	Ig18207, HBV, chrom 5: 83,326,648 inner reverse	ATAGATTACAAGCTGCTTTCTGGC
GN57	Ig18207, HBV, chrom 6: 15,085,893 outer forward	TTGGAGGGTGTTTACTCTTTTTCTA
GN58	Ig18207, HBV, chrom 6: 15,085,893 outer reverse	TCAACTCACCCCAACACAGA
GN59	Ig18207, HBV, chrom 6: 15,085,893 inner forward	TCTGGACCTAGAGTTGCTTTCT
GN60	Ig18207, HBV, chrom 6: 15,085,893 inner reverse	GCTTGCCTGAGTGCTGTATG
GN61	Ig18207, HBV, chrom 8: 89,410,201 outer forward	CAAACGTTGGGGCTACTCC
GN62	Ig18207, HBV, chrom 8: 89,410,201 outer reverse	AAAATCTTTTCCCAGGCCAGT
GN63	Ig18207, HBV, chrom 8: 89,410,201 inner forward	TGGGCTATGTAATTGGAAGTTGG
GN64	Ig18207, HBV, chrom 8: 89,410,201 inner reverse	GGCCAGTGTCCTAAAGCATT
GN65	Ig18207, HBV, chrom 11: 20,457,398 outer forward	GTCTAGACTCGTGGTGGACTTC

GN66	Ig18207, HBV, chrom 11: 20,457,398 outer reverse	AACCCCCTTTCTCCTTCCTTCT
GN67	Ig18207, HBV, chrom 11: 20,457,398 inner forward	CTGGCCAAAATTCGCAGTCC
GN68	Ig18207, HBV, chrom 11: 20,457,398 inner reverse	CTGGGGTTCCTCTTTCCGTT
GN69	Ig18207, HBV, chrom 17: 12,963,681 outer forward	TCAGACCACCTCTGCCAATG
GN70	Ig18207, HBV, chrom 17: 12,963,681 outer reverse	GGGTTCAGATGTATACCCAAAGAC
GN71	Ig18207, HBV, chrom 17: 12,963,681 inner forward	GCCGGCCTTGCACCAA
GN72	Ig18207, HBV, chrom 17: 12,963,681 inner reverse	AGAGGTAAAAAGGGACTCAAGATGT
GN73	Ig18207, HBV, chrom 18: 29,629,322 outer forward	GTTCACTTGCCCTGCTTCAG
GN74	Ig18207, HBV, chrom 18: 29,629,322 outer reverse	TGTGCTCTCCATGTTCGGTG
GN75	Ig18207, HBV, chrom 18: 29,629,322 inner forward	GGAGGATCAAAGGAAGTAACCGATA
GN76	Ig18207, HBV, chrom 18: 29,629,322 inner reverse	TTGACGATATGGGTGAGGCAG
GN77	Ig18207, HBV, chrom 21: 9,039,828 outer forward	GGCCCATTAGAGGAAGGTGA
GN78	Ig18207, HBV, chrom 21: 9,039,828 outer reverse	GAAGGAAAGAAGTCAGAAGGCA
GN79	Ig18207, HBV, chrom 21: 9,039,828 inner forward	GCACCCGGGCAGAGAAAA
GN80	Ig18207, HBV, chrom 21: 9,039,828 inner reverse	AGTAACTCCACAGAAGCTCCA
GN81	Ig18207, HBV, chrom X: 101,366,104 outer forward	CTCTGCTCTGTATCGGGAGG
GN82	Ig18207, HBV, chrom X: 101,366,104 outer reverse	CTCTATCGTGCAGGCTGGAG
GN83	Ig18207, HBV, chrom X: 101,366,104 inner forward	GCCTTAGAGTCTCCGGAACA
GN84	Ig18207, HBV, chrom X: 101,366,104 inner reverse	GCACAATCTCGGCTCACTG
GN85	Ig18207, HBV, chrom Y: 11,204,217 outer forward	TTTAGAAGGGGCAGGTCTCC
GN86	Ig18207, HBV, chrom Y: 11,204,217 outer reverse	CAGTATGGATCGGCAGAGGA
GN87	Ig18207, HBV, chrom Y: 11,204,217 inner forward	TCTGAGGAGTGCACAGCC
GN88	Ig18207, HBV, chrom Y: 11,204,217 inner reverse	AATAGCCAAGCCCCATCCAG
GN89	Ig18207, HBV chrom 17: 44,543,785 outer forward	TGTCCTACTGTTCAAGCCTCC
GN90	Ig18207, HBV chrom 17: 44,543,785 outer reverse	AGGTATGCACAACCAGATGCT
GN91	Ig18207, HBV chrom 17: 44,543,785 inner forward	GTGGCTTTGGGGCATGGAC
GN92	Ig18207, HBV chrom 17: 44,543,785 inner reverse	CCCCAGCTTCAAAGAGGCTATATCA
GN96	Ig18207, HBV chrom 17: 52,457,871 outer forward	AGCAAACTAACCCAGAAACAGA

GN97	Ig18207, HBV chrom 17: 52,457,871 outer reverse	AAGAGGGCCCACATACTGTT
GN98	Ig18207, HBV chrom 17: 52,457,871 inner forward	AGCAAACTAACCCAGAAACAGA
GN99	Ig18207, HBV chrom 17: 52,457,871 inner reverse	AGAGGGAGTTTGCCAATCAG
GN100	Ig18207, HBV, chrom 14: 96,321,592 outer forward	AACAAGGATCAATGGCCAGC
GN101	Ig18207, HBV, chrom 14: 96,321,592 outer reverse	TGGGAGCCAGGATCATCTC
GN102	Ig18207, HBV, chrom 14: 96,321,592 inner forward	AGTGGGATCATTCGGGCC
GN103	Ig18207, HBV, chrom 14: 96,321,592 inner reverse	TCCATTTTACAGAAGAGGATGCT
GN104	Ig18207, HBV, chrom 4: 128,649,683 outer forward	CAGCAATGTCAACGACCGAC
GN105	Ig18207, HBV, chrom 4: 128,649,683 outer reverse	CACCCCTGCCACATAACACT
GN106	Ig18207, HBV, chrom 4: 128,649,683 inner forward	GGGGAGGAGCCTAGGTTAATG
GN107	Ig18207, HBV, chrom 4: 128,649,683 inner reverse	GCCACTGGAAAATATGACCAGG
GN122	Ig18207, HBV, chrom 1: 197,394,924 outer forward	CTAGATCCCAGAGTGAGGGGC
GN123	Ig18207, HBV, chrom 1: 197,394,924 outer reverse	GGTTGCACTCAAATAAGATGGGGT
GN124	Ig18207, HBV, chrom 1: 197,394,924 inner forward	CCCAGAGTGAGGGGCCTAT
GN125	Ig18207, HBV, chrom 1: 197,394,924 inner reverse	TGCACTCAAATAAGATGGGGTA
GN126	Ig18207, HBV, chrom 1: 229,564,071 outer/inner forward	CTGCAGCCCTGCTGCTAATA
GN127	Ig18207, HBV, chrom 1: 229,564,071 outer revverse	AGACCGCCGTGTGGTG
GN128	Ig18207, HBV, chrom 1: 229,564,071 inner reverse	GTGGTGGGGTGAACCCTG
GN129	Ig18207, HBV, chrom 2: 37,252,888, outer forward	GCCTCATCTTCTTGTTGGTTCT
GN130	Ig18207, HBV, chrom 2: 37,252,888, outer reverse	AGCCTAAGCAATAACTGGTTTTGTA
GN131	Ig18207, HBV, chrom 2: 37,252,888, inner forward	TGTTGGTTCTTCTGGACTACC
GN132	Ig18207, HBV, chrom 2: 37,252,888, inner reverse	GCCTAAGCAATAACTGGTTTTGTA
GN133	Ig18207, HBV, chrom 4: 128,649,683 outer/inner forward	GCAACTTTTTCACCTCTGCCTA
GN134	Ig18207, HBV, chrom 4: 128,649,683 outer reverse	TTCCAGAAACTAGTGTCCCTTATC
GN135	Ig18207, HBV, chrom 4: 128,649,683 inner reverse	CTAGTGTCCCTTATCTTTCCACTGA
GN136	Ig18207, HBV, chrom 4: 92,946,155 outer forward	TATTCTTCAACCGCTGTTTCCTT
GN137	Ig18207, HBV, chrom 4: 92,946,155 outer reverse	AAAGAGAGTAACTCCACAGAAGCTC
GN138	Ig18207, HBV, chrom 4: 92,946,155 inner forward	TTCTTCAACCGCTGTTTCCTT

GN139	Ig18207, HBV, chrom 4: 92,946,155 inner reverse	AGTAACTCCACAGAAGCTCCAA
GN140	Ig18207, HBV, chrom 4: 137,637,347 outer/inner forward	GAGTGACTAAATTGCACCCTTTTT
GN141	Ig18207, HBV, chrom 4: 137,637,347 outer revverse	TCCATATAACTGAAAGCCAAACAGT
GN142	Ig18207, HBV, chrom 4: 137,637,347 inner revsrse	TATAACTGAAAGCCAAACAGTGGG
GN143	Ig18207, HBV, chrom 11: 20,457,398 outer/inner forward	TCCTCCAATTTGTCCTGGCT
GN144	Ig18207, HBV, chrom 11: 20,457,398 outer revverse	TCATGCATAATGGAAAGCATATGGA
GN145	Ig18207, HBV, chrom 11: 20,457,398 inner revsrse	TGGAAAGCATATGGATTTCACAAT
GN146	Ig18207, HBV, chrom 14: 96,321,592 outer forward	ACAACAGTGCCAGCAGCG
GN147	Ig18207, HBV, chrom 14: 96,321,592 outer/inner reverse	GGCTAGATAAACTGCTCAAGGC
GN148	Ig18207, HBV, chrom 14: 96,321,592 inner forward	AGCAGCGCCTCCTCCT
GN149	Ig18207, HBV, chrom 17: 12,963,681 outer/inner forward	CTTGCACCAGTGCCGTGT
GN150	Ig18207, HBV, chrom 17: 12,963,681 outer reverse	TAGTACAGACTTGGCCCCCA
GN151	Ig18207, HBV, chrom 17: 12,963,681 inner reverse	CTTGGCCCCCAATACCACAT
GN152	Ig18207, HBV chrom 17: 44,543,785 outer/inner forward	AGCTTCTGTGGAGTTACTCTCTTT
GN153	Ig18207, HBV chrom 17: 44,543,785 outer reverse	AACCAGTATTCACATATTTGTCCCT
GN154	Ig18207, HBV chrom 17: 44,543,785 inner reverse	CATATTTGTCCCTCTAGACTACCA
GN155	Ig18207, HBV chrom 17: 52,457,871 outer forward	GCACCAAGAAGGGAAAAACACA
GN156	Ig18207, HBV chrom 17: 52,457,871 outer reverse	GCCCAGTAAAGTTTCCCACCT
GN157	Ig18207, HBV chrom 17: 52,457,871 inner forward	GAAGGGAAAAACACATATGGGGG
GN158	Ig18207, HBV chrom 17: 52,457,871 inner reverse	CCAGTAAAGTTTCCCACCTTAATCC
GN159	Ig18207, HBV, chrom 18: 29,629,322 outer forward	TACTGCTTGGAATTGCTTGGAC
GN160	Ig18207, HBV, chrom 18: 29,629,322 outer reverse	GGAGCCACCAGCAGGAAAATA
GN161	Ig18207, HBV, chrom 18: 29,629,322 inner forward	TGCTTGGAATTGCTTGGACAT
GN162	Ig18207, HBV, chrom 18: 29,629,322 inner reverse	ACCAGCAGGAAAATATAGGCCC
GN163	Ig18207, HBV, chrom Y: 11,204,217 outer forward	AGCCAGTGTCTCCATAGCAAC
GN164	Ig18207, HBV, chrom Y: 11,204,217 outer reverse	AACGGGGTAAAGGTGCAGATATT
GN165	Ig18207, HBV, chrom Y: 11,204,217 inner forward	GTCTCCATAGCAACTGCTCCA
GN166	Ig18207, HBV, chrom Y: 11,204,217 inner reverse	GGGGTAAAGGTGCAGATATTGTT

GN183	Ig18207, HBV, chrom 4: 92,946,155 outer forward	AAGGTTCATGGTAGCTGCTTCA
GN184	Ig18207, HBV, chrom 4: 92,946,155 outer reverse	TGCCATCCATTCTAATGCACC
GN185	Ig18207, HBV, chrom 4: 92,946,155 inner forward	TCTGCCTCCCAACCCTCTAA
NG186	Ig18207, HBV, chrom 4: 92,946,155 inner reverse	TCTAATGCACCCTTTTATTGTTTCT
GN187	Ig18207, HBV chrom 17: 52,457,871 outer forward	GGGAACACTTGCACACTGTT
GN188	Ig18207, HBV chrom 17: 52,457,871 outer reverse	CTAGCCAGTTTTCCCAGCAC
GN189	Ig18207, HBV chrom 17: 52,457,871 inner forward	GGCAATTCCTCAAAGACCTAAAAC
GN190	Ig18207, HBV chrom 17: 52,457,871 inner reverse	GTTTGAAGTCAGGTAGCGTGA
GN223	Ig18207 HBV, chrom 2: 37,252,888 outer forward	TCCCCAACCTCCAATCACTCACC
GN224	Ig18207 HBV, chrom 2: 37,252,888 outer/inner reverse	ATGAGTAGGTACAGGCGGGAAACTG
GN225	Ig18207 HBV, chrom 2: 37,252,888 inner forward	GCTGGATGTGTCTGCGGCGT
GN226	Ig18207 HBV, chrom 2: 25,251,880 outer forward	ATCTCATGTTCATGTCCTACTGTTCAAGCC
GN227	Ig18207 HBV, chrom 2: 25,251,880 outer reverse	TTCTGGAGATGCTGACTTAGTGCTGG
GN228	Ig18207 HBV, chrom 2: 25,251,880 inner forward	CTTGGGTGGCTCTGGGGCAT
GN229	Ig18207 HBV, chrom 2: 25,251,880 inner reverse	AGATGCTGACTTAGTGCTGGCCC
GN230	Ig18207 HBV, chrom 5: 44,013,798 outer forward	GGACGAATCTTTCTGTTCCCAATCCTCTG
GN231	Ig18207 HBV, chrom 5: 44,013,798 outer reverse	GGTTGGAAATGCAGAAATCACCCATCTTCT
GN232	Ig18207 HBV, chrom 5: 44,013,798 inner forward	GAATCTTTCTGTTCCCAATCCTCTG
GN233	Ig18207 HBV, chrom 5: 44,013,798 inner reverse	GAGCTGTTCCTATTTGTCCATCTTG
GN234	Ig18207 HBV, chrom 6: 72,714,913 outer /inner forword	GAAAGACAATTTATGACAGGAACAAATACTATG
GN235	Ig18207 HBV, chrom 6: 72,714,913 outer reverse	GATCTTCCAAATTACTTCCCACCCAG
GN236	Ig18207 HBV, chrom 6: 72,714,913 inner reverse	TCATCAATTCACCCCAACACAGAATAGCTT
GN251	Ig18207 HBV, chrom 2: 37,252,888 outer forward	ATTCCTCTTCATCCCGCTGC
GN252	Ig18207 HBV, chrom 2: 37,252,888 outer reverse	AGCCTAAGCAATAACTGGTTTTGTA
GN253	Ig18207 HBV, chrom 2: 37,252,888 inner forward	TCTTCATCCCGCTGCTATGC
GN254	Ig18207 HBV, chrom 2: 37,252,888 inner reverse	GCCTAAGCAATAACTGGTTTTGTA
GN255	Ig18207 HBV, chrom 5: 44,013,798 outer forward	AGGCAAATCAGGTAGGAGCG
GN256	Ig18207 HBV, chrom 5: 44,013,798 outer reverse	TGGGAGCTGTAGACTGGAGC

GN257	Ig18207 HBV, chrom 5: 44,013,798 inner forward	CAAATCAGGTAGGAGCGGGA
GN258	Ig18207 HBV, chrom 5: 44,013,798 inner reverse	GCTGTAGACTGGAGCTGTTC
**Ig18207, HHV-7**		
GN5	Ig18207, HHV-7, chrom 4: 40,983,878 outer forward	CCAAGTTTTGCATAGTCTGAGG
GN6	Ig18207, HHV-7, chrom 4: 40,983,878 outer reverse	TGTGGCACATAAGTTGACAATG
GN7	Ig18207, HHV-7, chrom 4: 40,983,878 inner forward	GGATCATTTAATTAACCATTGAACTC
GN8	Ig18207, HHV-7, chrom 4: 40,983,878 inner reverse	TTGAGAAATATGAATTGATGATGGA
GN13	Ig18207, HHV-7, chrom 7: 153,676,002 outer forward	AGTGTGCTCCCACAGTAAGA
GN14	Ig18207, HHV-7, chrom 7: 153,676,002 outer reverse	ACGTAACGGATTACTTGTAAACTCA
GN15	Ig18207, HHV-7, chrom 7: 153,676,002 inner forward	TGCTTGAGCGTATAGCCTTCAT
GN16	Ig18207, HHV-7, chrom 7: 153,676,002 inner reverse	ACTCACTTCACGAAAAATTGATGGA
GN17	Ig18207, HHV-7, chrom 9: 104,407,448 HHV-7,142420, outer forward	GTGCATCACATGAGAACGGC
GN18	Ig18207, HHV-7, chrom 9: 104,407,448 HHV-7,142420, outer reverse	CCTGGCTACAGAGAGGAGAGT
GN19	Ig18207, HHV-7, chrom 9: 104,407,448 HHV-7,142420, inner forward	GAGGGCTGCAGCGAGAAT
GN20	Ig18207, HHV-7, chrom 9: 104,407,448 HHV-7,142420, inner reverse	ACAGAGAGGAGAGTGAATTGGT
GN21	Ig18207, HHV-7, chrom 12: 107,555,401 outer forward	TGCCGGTTGAGGAGTAAAAGT
GN22	Ig18207, HHV-7, chrom 12: 107,555,401 outer reverse	CACCCACTTCAGGGCTGTTA
GN23	Ig18207, HHV-7, chrom 12: 107,555,401 inner forward	AACTGTAGTATTTTCTCGCAAAAGC
GN24	Ig18207, HHV-7, chrom 12: 107,555,401 inner reverse	ACTCGGTTGCCAAGGAGTTC
GN25	Ig18207, HHV-7, chrom 19: 53,455,783 outer forward	ACCTTGCAGAAGCTGTTCGT
GN26	Ig18207, HHV-7, chrom 19: 53,455,783 outer reverse	AGGTTGCTCTCCAGTATGACG
GN27	Ig18207, HHV-7, chrom 19: 53,455,783 inner forward	TGGTGTGTAAAATCAACCATTGTG
GN28	Ig18207, HHV-7, chrom 19: 53,455,783 inner reverse	TGTAAGGGATGATGTCCGGC
GN108	Ig18207, HHV-7, chrom 4: 40,983,878 outer forward	GAAACCTCATGATTCAGGGATCAAA
GN109	Ig18207, HHV-7, chrom 4: 40,983,878 outer reverse	ATAAGAATCTGTCTGTCGGCAA
GN110	Ig18207, HHV-7, chrom 4: 40,983,878 inner forward	TGATTCAGGGATCAAAAACACCA
GN111	Ig18207, HHV-7, chrom 4: 40,983,878 inner reverse	AAGAATCTGTCTGTCGGCAA
GN118	Ig18207, HHV-7, chrom 19: 53,455,783 outer forward	AGGAAGGTATTATTTTGGAGGCTGT

GN119	Ig18207, HHV-7, chrom 19: 53,455,783 outer reverse	GCATTGTCTTCAGTATGAATTTTCC
GN120	Ig18207, HHV-7, chrom 19: 53,455,783 inner forward	TTATTTTGGAGGCTGTGTTAGCTG
GN121	Ig18207, HHV-7, chrom 19: 53,455,783 inner reverse	TGTCTTCAGTATGAATTTTCCGATG
GN167	Ig18207, HHV-7, chrom 4: 40,983,878 outer forward	TGTAGCAAAACCAGCTCCCA
GN168	Ig18207, HHV-7, chrom 4: 40,983,878 outer reverse	CCATGATCAACTTCTGTTTTTCTCA
GN169	Ig18207, HHV-7, chrom 4: 40,983,878 inner forward	CAGCTCCCAATGTAGCAAAAC
GN170	Ig18207, HHV-7, chrom 4: 40,983,878 inner reverse	CTCTGGGCTCAGTCACATGAA
GN171	Ig18207, HHV-7, chrom 9: 104,407,448 HHV-7,142420, outer forward	CCTTCCCAATGGTGGACACA
GN172	Ig18207, HHV-7, chrom 9: 104,407,448 HHV-7,142420, outer reverse	GGAGCAAAGCACATGGGGAA
GN173	Ig18207, HHV-7, chrom 9: 104,407,448 HHV-7,142420, inner forward	TGTCATGGCACAGTCTTCATCA
GN174	Ig18207, HHV-7, chrom 9: 104,407,448 HHV-7,142420, inner reverse	AAGCACGATGACATGAGGGAA
GN175	Ig18207, HHV-7, chrom 12: 107,555,401 outer forward	GGATCCCTCTGCAGTTTGCT
GN176	Ig18207, HHV-7, chrom 12: 107,555,401 outer reverse	CAAAGGCTGTCCAATCGGATG
GN177	Ig18207, HHV-7, chrom 12: 107,555,401 inner forward	GCAGCCAGCTCCTCTAACAT
GN178	Ig18207, HHV-7, chrom 12: 107,555,401 inner reverse	CAATCGGATGGAGTGATTTGATT
GN179	Ig18207, HHV-7, chrom 19: 53,455,783 outer forward	TCCTTACATGAGCTTGTTGTGGA
GN180	Ig18207, HHV-7, chrom 19: 53,455,783 outer reverse	ACGATGGCGTTTAAGGGTTA
GN181	Ig18207, HHV-7, chrom 19: 53,455,783 inner forward	TCGTGTGGTCCTTTAGCATT
GN182	Ig18207, HHV-7, chrom 19: 53,455,783 inner reverse	GGGTTAACTGCTGATTGAAGGT
GN203	Ig18207, HHV-7, chrom 4: 40,983,878 outer forward	ACCTCATGATTCAGGGATCAAAAAC
GN204	Ig18207, HHV-7, chrom 4: 40,983,878 outer reverse	TTACAGCACATACAAAAAGAACCA
GN205	Ig18207, HHV-7, chrom 4: 40,983,878 inner forward	TGATTCAGGGATCAAAAACACCA
GN206	Ig18207, HHV-7, chrom 4: 40,983,878 inner reverse	TGTGGCACATAAGTTGACAATG
GN207	Ig18207, HHV-7, chrom 12: 107,555,401 outer forward	AGCTCTTAGAGGCTTATTGATTGT
GN208	Ig18207, HHV-7, chrom 12: 107,555,401 outer reverse	GTAGAAATTGTTTCTGACCACAGGT
GN209	Ig18207, HHV-7, chrom 12: 107,555,401 inner forward	TGATTGTTTTCCAAGTTTTCATGTG
GN210	Ig18207, HHV-7, chrom 12: 107,555,401 inner reverse	GAAATTGTTTCTGACCACAGGTGA
GN211	Ig18207, HHV-7, chrom 19: 53,455,783 outer forward	CAAGGAAGGTATTATTTTGGAGGCT

GN212	Ig18207, HHV-7, chrom 19: 53,455,783 outer reverse	TCAAGGTTTGATTTGAAACGGAA
GN213	Ig18207, HHV-7, chrom 19: 53,455,783 inner forward	TTATTTTGGAGGCTGTGTTAGCTG
GN214	Ig18207, HHV-7, chrom 19: 53,455,783 inner reverse	TCACATTTGTAAGGTTGCTCTCC
GN259	Ig18207, HHV-7, chrom 4: 40,983,878 outer forward	ATTAACCATTGAACTCACTAGCAG
GN260	Ig18207, HHV-7, chrom 4: 40,983,878 outer reverse	TGATGATGGATAACTATGAACTGGC
GN261	Ig18207, HHV-7, chrom 4: 40,983,878 inner forward	CCATTGAACTCACTAGCAGTTTT
GN262	Ig18207, HHV-7, chrom 4: 40,983,878 inner reverse	TGGATAACTATGAACTGGCACATT
**Ig18203**		
GN367	Ig18203 HBV chrom13: 60,418,226 forward	TGGTGTCTTTTGGAGTGTGGA
GN368	Ig18203 HBV chrom13: 60,418,226 reverse	AAACCATGGTACTGACCTCAAA
GN369	Ig18203 HBV chrom13: 60,418,226 forward	CGCTTACAGACCACCAAATGC
GN370	Ig18203 HBV chrom13: 60,418,226 reverse	TTTAGTCCCAGTGAAGCTTGGT
GN371	Ig18203 HBV chrom13: 60,418,226 forward	TATTAGACGACGCAGCAGGT
GN372	Ig18203 HBV chrom13: 60,418,226 reverse	GCACAATGAGCTAGAGACAGA
GN373	Ig18203 HBV chrom13: 60,418,226 forward	AAGAAGAACTCCCTCGCCTC
GN374	Ig18203 HBV chrom13: 60,418,226 reverse	AGCACAATGAGCTAGAGACAGAA
GN375	Ig18203 HBV chrom13: 60,418,226 forward	GATCTCAATCGCCGCGTC
GN376	Ig18203 HBV chrom13: 60,418,226 reverse	GCACAATGAGCTAGAGACAGA
GN377	Ig18203 HBV chrom13: 60,418,226 forward	CCTTGGACTCATAAGGTGGGA
GN378	Ig18203 HBV chrom13: 60,418,226 reverse	GGTACCTACTTTCTATCAGGGAAGA
GN379	Ig18203 HBV chrom13: 60,418,226 forward	CGCAGAAGATCTAAATCTCGGGAA
GN380	Ig18203 HBV chrom13: 60,418,226 reverse	TCCAAGCAGAAAGGACAAGATCA
GN518	Ig18203 HBV chrom13: 60,418,226 forward	GGAATCTCAATGTTAGTATCCCTTG
GN519	Ig18203 HBV chrom13: 60,418,226 reverse	TCCAAGCAGAAAGGACAAGATCA
GN381	Ig18203 HHV-7 chrom 2: 87,648,781 forward	AAGAGATGTTTGAAATCATTGTGTTTTGAC
GN382	Ig18203 HHV-7 chrom 2: 87,648,781 reverse	AAATTTTATCTCTTTACCATATCAACCCCAATC
GN383	Ig18203 HHV-7 chrom 2: 87,648,781 forward	TTTTGACCAGAAACTTCATTAGCCTGA
GN384	Ig18203 HHV-7 chrom 2: 87,648,781 reverse	GTAGAAATTATTGTAACCCTAACTCAAAATACC

GN385	Ig18203 HHV-7 chrom 2: 87,648,781 forward	AGAGTGCTGGGGACTTGGAT
GN386	Ig18203 HHV-7 chrom 2: 87,648,781 reverse	TGGTAAACATTTTCACAGACCTTAC
GN387	Ig18203 HHV-7 chrom 2: 87,648,781 forward	ACAAGTGGCCCTGTCACCTC
GN388	Ig18203 HHV-7 chrom 2: 87,648,781 reverse	CACAGACCTTACAACAGACGTGG
**Ig18204**		
GN344	Ig18204 HHV-7, chrom11: 4,493,831 forward	GTATTCTCTTAATATGGGCTCTTAATTTAGTTTGAG
GN345	Ig18204 HHV-7, chrom11: 4,493,831 reverse	TATGCTTATAATAAAGATCAACATAGCTGGGAAAAG
GN346	Ig18204 HHV-7, chrom11: 4,493,831 forward	GGGCTCTTAATTTAGTTTGAGATCCATAAAGAATAA
GN347	Ig18204 HHV-7, chrom11: 4,493,831 reverse	CTGGGATGATAACTTAACCTTAGATATTTCAAAACT
GN348	Ig18204 HHV-7, chrom11: 4,493,831 forward	TTTAGTTTGAGATCCATAAAGAATAAATTGAGCACG
GN349	Ig18204 HHV-7, chrom11: 4,493,831 reverse	TCCCTTTAAATGGATCAAACCCCTT
GN350	Ig18204 HHV-7, chrom11: 4,493,831 forward	GAGTTGTTTGTAAATCTATCCAAAATTCACAC
GN351	Ig18204 HHV-7, chrom11: 4,493,831 reverse	CTTATGCTTATAATAAAGATCAACATAGCTGGGA
GN352	Ig18204 HHV-7, chrom11: 4,493,831 forward	GTTGTAATTCTGTCCAGCGTATTCTCTTAATA
GN353	Ig18204 HHV-7, chrom11: 4,493,831 reverse	AAGACATTTAAAAGCCTGGGATGATAACTTAA
GN354	Ig18204 HHV-7, chrom11: 4,493,831 forward	AAGAATAAATTGAGCACGGCACT
GN355	Ig18204 HHV-7, chrom11: 4,493,831 reverse	TCCCTTTAAATGGATCAAACCCCTT
**Ig18205**		
GN314	Ig18205 HHV-7, chrom 20: 25,781,706 forward	TTCTTATTCTCTGCTATCATACAATTACTACTGATG
GN315	Ig18205 HHV-7, chrom 20: 25,781,706 reverse	GAATCATGTCACCAATACAAAGTTTTAAGGGGAAC
GN316	Ig18205 HHV-7, chrom 20: 25,781,706 forward	ATATGACCTCACAAGAATCAGTTACTTCACCAAC
GN317	Ig18205 HHV-7, chrom 20: 25,781,706 reverse	TTAGAAAATAAGACCAAAGTTACTAAGCCTACAG
GN318	Ig18205 HHV-7, chrom 20: 25,781,706 forward	TCATACAATTACTACTGATGATGGAGCTTATTTTAC
GN319	Ig18205 HHV-7, chrom 20: 25,781,706 reverse (and reverse for GN 320)	CTTAGAAAATAAGACCAAAGTTACTAAGCCTACAG
GN320	Ig18205 HHV-7, chrom 20: 25,781,706 forward	TTATTTTACAAGAAAAGAAGTGATGCGATGGG
GN321	Ig18205 HHV-7, chrom 20: 25,781,706 reverse	GGGCTGGGTTTCTATCCTACAAGATGTG
GN322	Ig18205 HHV-7, chrom 20: 25,781,706 forward	ATATGACCTCACAAGAATCAGTTACTTCACCAAC
GN323	Ig18205 HHV-7, chrom 20: 25,781,706 reverse	GCTGTTACTTCCGAACCAACAAAATTTT

GN324	Ig18205 HHV-7, chrom 20: 25,781,706 forward	CCCTGAGGGGCTGGGTTTCTA
GN325	Ig18205 HHV-7, chrom 20: 25,781,706 reverse	GCAATTCCGAACGCTGTTACTTCCG
GN326	Ig18205 HHV-7, chrom 20: 25,781,706 forward	AGAAAAGAAGTGATGCGATGGGCTT
GN327	Ig18205 HHV-7, chrom 20: 25,781,706 reverse	TGTGAACACGCGGAATGTGTCAAC
GN328	Ig18205 HHV-7, chrom 20: 25,781,706 forward	AAGAAGTGATGCGATGGGCT
GN329	Ig18205 HHV-7, chrom 20: 25,781,706 reverse	GTGAACACGCGGAATGTGTC
GN330	Ig18205 HHV-7, chrom 20: 25,781,706 forward (and forward for GN332)	AGGGGCTGGGTTTCTATCCT
GN331	Ig18205 HHV-7, chrom 20: 25,781,706 reverse	TTCACGCGAAAATTCTGAGCA
GN332	Ig18205 HHV-7, chrom 20: 25,781,706 reverse	GGTAAGGGGAATCATGTCACCA
GN333	Ig18205 HHV-7, chrom 20: 25,781,706 forward	TGTGTTAGAAGCCCTGACCC
GN334	Ig18205 HHV-7, chrom 20: 25,781,706 reverse	GGTGTTACCAACTTCTCCTTGGT
GN335	Ig18205 HHV-7, chrom 20: 25,781,706 forward	CAAGAGCCCTGCTACCCAGA
GN336	Ig18205 HHV-7, chrom 20: 25,781,706 reverse	AGCAATTCCGAACGCTGTTAC
GN337	Ig18205 HHV-7, chrom 20: 25,781,706 forward	GCATGGTTTCATGTAGCAAGGA
GN338	Ig18205 HHV-7, chrom 20: 25,781,706 reverse	CCGAACGCTGTTACTTCCGA
GN339	Ig18205 HHV-7, chrom 20: 25,781,706 forward (and forward for GN341)	CTTGTGATGGTGGTGATGATGATGG
GN340	Ig18205 HHV-7, chrom 20: 25,781,706 reverse	CTTAGAAAATAAGACCAAAGTTACTAAGCCTAC
GN341	Ig18205 HHV-7, chrom 20: 25,781,706 reverse	GCTGTTACTTCCGAACCAACAAAAT
GN342	Ig18205 HHV-7, chrom 20: 25,781,706 forward	CATACAATTACTACTGATGATGGAGCTTATTTTAC
GN343	Ig18205 HHV-7, chrom 20: 25,781,706 reverse	CGAACAGATTGCAAGAATTCTATTTCAAAA
**Ig18208, HBV**		
GN600	Ig18208 HBV, chrom 2: 21,5418,186 outer forward	AGGTAGTGTTAGCCAAGCCTC
GN601	Ig18208 HBV, chrom 2: 21,5418,186 outer reverse	AGACTCTAAGGCCTCCCGAT
GN602	Ig18208 HBV, chrom 2: 21,5418,186 inner forward	TCCTAAATACTAAACACCCAGATGC
GN603	Ig18208 HBV, chrom 2: 21,5418,186 inner reverse	CAAAGCCACCCAAGGCACA
**Ig18808, HHV-7**		
Becase the nucleotide basic local alignment search tool (BLAST) of National Library of Medicine could not find any highly similar sequence, we could not design PCR primers		
