# Supplementary material for: Integration of Viral Genome to Human Genomic DNA in Nails of Patients with Chronic Hepatitis B Virus Infection
Source: JMA J. 2023 Sep 29;6(4):426–36. doi: 10.31662/jmaj.2023-0082 (PMC10628332; doi:10.31662/jmaj.2023-0082)
Supplement: Supplementary Table 13 [file 2433-3298-6-4-426-s016.pdf]

Supplementary Table 13. Summary of paired-end reads in the nail samples

**ID**	Yield (M bases)	Number of reads	≧ Q30 bases,%	Quality score mean	Number of trimmed reads	Read length mean (Trimmed reads)
Ig18203	604	5,977,846	91.9	37.4	5,343,500	98.4
Ig18204	650	6,435,316	91.63	37.29	5,744,014	98.33
Ig18205	899	8,898,058	89.28	36.76	7,660,758	98.38
