# Supplementary material for: Integration of Viral Genome to Human Genomic DNA in Nails of Patients with Chronic Hepatitis B Virus Infection
Source: JMA J. 2023 Sep 29;6(4):426–36. doi: 10.31662/jmaj.2023-0082 (PMC10628332; doi:10.31662/jmaj.2023-0082)
Supplement: Supplementary Table 14 [file 2433-3298-6-4-426-s017.pdf]

Supplementary Table 14. Summary of mapped and unmapped reads in the nail samples

**ID**	Processed reads (trimmed paired reads)	Number of mapped reads	Mapped reads/processed reads, %	Number of duplicated reads	Duplicated reads/processed reads, %	Number of unmapped reads	Unmapped reads/processed reads, %
Ig18203	5,343,500	5,223,720	97.76	4,305,140	80.57	119,780	2.24
Ig18204	5,744,014	5,574,570	97.05	4,550,019	79.21	169,444	2.95
Ig18205	7,660,758	7,260,935	94.78	5,919,880	77.28	399,823	5.22
