# Supplementary material for: Integration of Viral Genome to Human Genomic DNA in Nails of Patients with Chronic Hepatitis B Virus Infection
Source: JMA J. 2023 Sep 29;6(4):426–36. doi: 10.31662/jmaj.2023-0082 (PMC10628332; doi:10.31662/jmaj.2023-0082)
Supplement: Supplementary Table 15 [file 2433-3298-6-4-426-s018.pdf]

Supplementary Table 15. Summary of mapped deduplicated reads in the nail samples

Reference	Chromosome	Length (bp)	Ig18203 (Nails)	Ig18204 (Nails)	Ig18205 (Nails)
Host DNA: Human (GRCh38)	1	248,956,422	111,456	143,835	233,699
	2	242,193,529	45,564	54,571	62,918
	3	198,295,559	43,689	42,235	50,709
	4	190,214,555	42,291	49,829	67,833
	5	181,538,259	48,794	61,804	66,992
	6	170,805,979	40,944	32,548	50,776
	7	159,345,973	48,903	43,183	52,135
	8	145,138,636	31,219	33,776	44,460
	9	138,394,717	29,004	35,171	43,858
	10	133,797,422	42,465	40,991	57,138
	11	135,086,622	34,076	39,729	39,749
	12	133,275,309	32,237	36,817	41,808
	13	114,364,328	20,923	22,597	32,604
	14	107,043,718	20,388	22,245	31,343
	15	101,991,189	22,854	21,778	34,605
	16	90,338,345	27,608	37,885	41,281
	17	83,257,441	25,286	29,758	37,998
	18	80,373,285	37,401	43,370	58,728
	19	58,617,616	35,741	43,219	42,492
	20	64,444,167	37,167	34,493	40,886
	21	46,709,983	48,090	42,715	61,331
	22	50,818,468	17,687	19,193	27,984
	X	156,040,895	24,101	24,005	34,871
	Y	57,227,415	44,984	61,753	76,118
	Mitochondria	16,569	1,224	1,082	1,475
Exogenous DNA:	HBV_AB033550.1	3,215	3,529	604	421
	HHV7JI_HHU43400.1	144,861	955	5,365	6,843
Mapped deduplicated reads (mapped reads minus duplicated reads)			918,580	1,024,551	1,341,055
