# Supplementary material for: Integration of Viral Genome to Human Genomic DNA in Nails of Patients with Chronic Hepatitis B Virus Infection
Source: JMA J. 2023 Sep 29;6(4):426–36. doi: 10.31662/jmaj.2023-0082 (PMC10628332; doi:10.31662/jmaj.2023-0082)
Supplement: Supplementary Table 16 [file 2433-3298-6-4-426-s019.pdf]

Supplementary Table 16. Ig18203 HBV integration breakpoints

Chrom	Start	End	Insert_Seq Breakpoint	Seqcode	# Junction Reads	Fraction of MQ0 Reads	# Junction Reads (Dedup)	Fraction of MQ0 Reads (Dedup)	Feature	Gene Name	Trasncript Biotype
11	75,553,673	75,553,674	2,983	5prime(Human)-75553674-5prime(HBV)	1	0.00	.	.	gene	'SERPINH1	protein_coding
11	75,553,674	75,553,675	2,983	5prime(Human)-75553675-5prime(HBV)	15	0.00	1	0.00	gene	'SERPINH1	protein_coding
12	8,844,791	8,844,792	1,581	3prime(Human)-8844792-5prime(HBV)	31	0.00	1	0.00	intron	'A2ML1	protein_coding
13	60,418,226	60,418,227	2,509	3prime(HBV)-60418227-3prime(Human)	26	0.00	1	0.00	intron	'TDRD3	protein_coding
15	24,413,717	24,413,718	2,102	3prime(HBV)-24413718-5prime(Human)	12	0.00	1	0.00	intron	'PWRN1	lncRNA
21	8,234,008	8,234,009	2,355	3prime(Human)-8234009-5prime(HBV)	10	1.00	3	1.00	gene	'FP671120.10	TEC
21	8,416,599	8,416,600	2,355	3prime(Human)-8416600-5prime(HBV)	6	1.00	2	1.00	intron	'FP236383.3	lncRNA
21	8,461,161	8,461,162	2,355	3prime(Human)-8461162-5prime(HBV)	11	1.00	1	1.00	gene	'FP236383.11	TEC
21	8,461,161	8,461,162	2,357	3prime(Human)-8461162-5prime(HBV)	1	1.00	.	.	gene	'FP236383.11	TEC
