# Supplementary material for: Integration of Viral Genome to Human Genomic DNA in Nails of Patients with Chronic Hepatitis B Virus Infection
Source: JMA J. 2023 Sep 29;6(4):426–36. doi: 10.31662/jmaj.2023-0082 (PMC10628332; doi:10.31662/jmaj.2023-0082)
Supplement: Supplementary Table 17 [file 2433-3298-6-4-426-s020.pdf]

Supplementary Table 17. Ig18203 HHV-7 integration breakpoints

Chrom	Start	End	Insert_Seq Breakpoint	Seqcode	# Junction Reads	Fraction of MQ0 Reads	# Junction Reads (Dedup)	Fraction of MQ0 Reads (Dedup)	Feature	Gene Name	Trasncript Biotype
2	87,648,765	87,648,766	122,161	3prime(Human)-87648766-5prime(HHV)	11	1.00	1	1.00	intron	'AC133644.3	lncRNA
2	87,648,781	87,648,782	122,145	3prime(Human)-87648782-5prime(HHV)	19	1.00	1	1.00	intron	'AC133644.3	lncRNA
2	111,302,120	111,302,121	122,149	5prime(Human)-111302121-5prime(HHV)	1	1.00	.	.	intron	'MIR4435-2HG	lncRNA
2	111,302,121	111,302,122	122,145	5prime(Human)-111302122-5prime(HHV)	18	1.00	.	.	intron	'MIR4435-2HG	lncRNA
2	111,302,137	111,302,138	122,161	5prime(Human)-111302138-5prime(HHV)	17	1.00	1	1.00	intron	'MIR4435-2HG	lncRNA
4	10,185	10,186	74	5prime(Human)-10186-5prime(HHV)	1	1.00	1	1.00	gene	'BNIP3P41	lncRNA
10	5,744,298	5,744,299	103,299	3prime(HHV)-5744299-3prime(Human)	26	0.00	2	0.00	intron	'TASOR2	protein_coding
21	15,650,878	15,650,879	52,631	3prime(Human)-15650879-5prime(HHV)	34	0.00	1	0.00	gene	'AJ009632.2	lncRNA
21	34,712,902	34,712,903	139,110	5prime(Human)-34712903-5prime(HHV)	1	1.00	1	1.00	intron	'CLIC6	protein_coding
