# Supplementary material for: Integration of Viral Genome to Human Genomic DNA in Nails of Patients with Chronic Hepatitis B Virus Infection
Source: JMA J. 2023 Sep 29;6(4):426–36. doi: 10.31662/jmaj.2023-0082 (PMC10628332; doi:10.31662/jmaj.2023-0082)
Supplement: Supplementary Table 18 [file 2433-3298-6-4-426-s021.pdf]

Supplementary Table 18. Ig18204 HHV-7 integration breakpoints

Chrom	Start	End	Insert_Seq Breakpoint	Seqcode	# Junction Reads	Fraction of MQ0 Reads	# Junction Reads (Dedup)	Fraction of MQ0 Reads (Dedup)	Feature	Gene Name	Trasncript Biotype
1	10,254	10,255	139,200	3prime(HHV)-10255-3prime(Human)	1	1.00	.	.	gene	'DDX11L1	lncRNA
1	10,254	10,255	153	3prime(HHV)-10255-3prime(Human)	1	1.00	1	1.00	gene	'DDX11L1	lncRNA
1	248,945,731	248,945,732	144,566	3prime(HHV)-248945732-5prime(Human)	2	1.00	.	.	gene	'RPL23AP25	processed_pseudogene
1	248,945,731	248,945,732	5,519	3prime(HHV)-248945732-5prime(Human)	2	1.00	1	1.00	gene	'RPL23AP25	processed_pseudogene
1	248,946,106	248,946,107	139,200	3prime(HHV)-248946107-5prime(Human)	3	1.00	2	1.00	gene	'RPL23AP25	processed_pseudogene
1	248,946,106	248,946,107	153	3prime(HHV)-248946107-5prime(Human)	1	1.00	.	.	gene	'RPL23AP25	processed_pseudogene
3	10,021	10,022	5,417	3prime(HHV)-10022-3prime(Human)	1	0.00	1	0.00	gene	'LINC01986	lncRNA
3	10,218	10,219	153	3prime(HHV)-10219-3prime(Human)	1	0.00	.	.	gene	'LINC01986	lncRNA
3	10,221	10,222	139,177	3prime(HHV)-10222-3prime(Human)	1	1.00	1	1.00	gene	'LINC01986	lncRNA
3	10,393	10,394	144,566	3prime(HHV)-10394-3prime(Human)	1	1.00	.	.	gene	'LINC01986	lncRNA
3	10,529	10,530	5,519	3prime(HHV)-10530-3prime(Human)	1	1.00	1	1.00	gene	'LINC01986	lncRNA
3	10,750	10,751	5,513	3prime(HHV)-10751-3prime(Human)	1	1.00	1	1.00	gene	'LINC01986	lncRNA
3	3,132,713	3,132,714	142,975	5prime(Human)-3132714-5prime(HHV)	1	1.00	.	.	intron	'TRNT1	protein_coding
3	198,173,417	198,173,418	60	3prime(Human)-198173418-5prime(HHV)	1	1.00	1	1.00	intron	'FAM157A	lncRNA
3	198,173,865	198,173,866	139,198	3prime(HHV)-198173866-5prime(Human)	1	1.00	1	1.00	intron	'FAM157A	lncRNA
4	7,453,688	7,453,689	144,676	3prime(Human)-7453689-5prime(HHV)	2	0.00	1	0.00	intron	'SORCS2	protein_coding
4	49,274,531	49,274,532	139,107	5prime(Human)-49274532-5prime(HHV)	6	1.00	2	1.00	gene	'MTND3P22	processed_pseudogene
4	49,274,531	49,274,532	60	5prime(Human)-49274532-5prime(HHV)	6	1.00	2	1.00	gene	'MTND3P22	processed_pseudogene
4	49,335,727	49,335,728	139,107	3prime(Human)-49335728-5prime(HHV)	4	1.00	2	1.00	gene	'MTND3P22	processed_pseudogene
4	49,335,727	49,335,728	60	3prime(Human)-49335728-5prime(HHV)	7	1.00	2	1.00	gene	'MTND3P22	processed_pseudogene

4	104,464,381	104,464,382	136,393	3prime(HHV)-104464382-5prime(Human)	1	0.00	.	.	gene	'CXXC4	protein_coding
4	190,122,447	190,122,448	60	3prime(Human)-190122448-5prime(HHV)	2	1.00	1	1.00	gene	'DUX4L2	unprocessed_pseudogene
4	190,122,606	190,122,607	139,200	3prime(HHV)-190122607-5prime(Human)	1	1.00	1	1.00	gene	'DUX4L2	unprocessed_pseudogene
4	190,122,701	190,122,702	139,107	3prime(Human)-190122702-5prime(HHV)	1	0.00	1	0.00	gene	'DUX4L2	unprocessed_pseudogene
4	190,122,789	190,122,790	139,200	3prime(HHV)-190122790-5prime(Human)	37	1.00	1	1.00	gene	'DUX4L2	unprocessed_pseudogene
4	190,122,789	190,122,790	153	3prime(HHV)-190122790-5prime(Human)	32	1.00	1	1.00	gene	'DUX4L2	unprocessed_pseudogene
4	190,122,801	190,122,802	139,178	3prime(HHV)-190122802-5prime(Human)	1	1.00	.	.	gene	'DUX4L2	unprocessed_pseudogene
4	190,122,802	190,122,803	131	3prime(HHV)-190122803-5prime(Human)	1	0.00	.	.	gene	'DUX4L2	unprocessed_pseudogene
4	190,122,802	190,122,803	139,198	3prime(HHV)-190122803-5prime(Human)	34	0.03	.	.	gene	'DUX4L2	unprocessed_pseudogene
4	190,122,802	190,122,803	139,199	3prime(HHV)-190122803-5prime(Human)	1	0.00	.	.	gene	'DUX4L2	unprocessed_pseudogene
4	190,122,802	190,122,803	139,200	3prime(HHV)-190122803-5prime(Human)	1	0.00	.	.	gene	'DUX4L2	unprocessed_pseudogene
4	190,122,802	190,122,803	147	3prime(HHV)-190122803-5prime(Human)	1	0.00	.	.	gene	'DUX4L2	unprocessed_pseudogene
4	190,122,802	190,122,803	151	3prime(HHV)-190122803-5prime(Human)	30	0.03	.	.	gene	'DUX4L2	unprocessed_pseudogene
4	190,122,802	190,122,803	153	3prime(HHV)-190122803-5prime(Human)	1	0.00	.	.	gene	'DUX4L2	unprocessed_pseudogene
5	14,125,735	14,125,736	3,928	3prime(Human)-14125736-5prime(HHV)	1	1.00	.	.	gene	'TRIO	protein_coding
5	16,348,081	16,348,082	139,115	3prime(Human)-16348082-5prime(HHV)	1	1.00	.	.	gene	'LINC02150	lncRNA
8	13,615,474	13,615,475	142,975	5prime(Human)-13615475-5prime(HHV)	1	1.00	1	1.00	gene	'DLC1	lncRNA
9	2,824,041	2,824,042	139,111	3prime(Human)-2824042-5prime(HHV)	1	1.00	1	1.00	intron	'PUM3	protein_coding
9	134,210,369	134,210,370	139,195	3prime(HHV)-134210370-3prime(Human)	1	1.00	.	.	gene	'BX649601.1	lncRNA
9	134,210,371	134,210,372	139,200	3prime(HHV)-134210372-3prime(Human)	1	1.00	.	.	gene	'BX649601.1	lncRNA
10	9,996	9,997	144,636	3prime(HHV)-9997-3prime(Human)	1	0.00	1	0.00	gene	'AC215217.1	lncRNA
10	10,426	10,427	60	5prime(Human)-10427-5prime(HHV)	1	1.00	1	1.00	gene	'AC215217.1	lncRNA
10	4,069,189	4,069,190	144,566	3prime(HHV)-4069190-3prime(Human)	1	1.00	1	1.00	intron	'AC025822.2	lncRNA

10	8,468,445	8,468,446	139,200	3prime(HHV)-8468446-3prime(Human)	2	1.00	1	1.00	gene	'AC025946.2	processed_pseudogene
10	8,468,513	8,468,514	139,200	3prime(HHV)-8468514-3prime(Human)	4	1.00	.	.	gene	'AC025946.2	processed_pseudogene
10	8,468,513	8,468,514	153	3prime(HHV)-8468514-3prime(Human)	1	1.00	.	.	gene	'AC025946.2	processed_pseudogene
10	108,410,065	108,410,066	70,499	3prime(Human)-108410066-5prime(HHV)	59	0.00	.	.	gene	'PTGES3P5	processed_pseudogene
10	108,410,066	108,410,067	70,503	3prime(Human)-108410067-5prime(HHV)	1	0.00	.	.	gene	'PTGES3P5	processed_pseudogene
11	175,795	175,796	139,107	5prime(Human)-175796-5prime(HHV)	1	1.00	1	1.00	intron	'BET1L	protein_coding
11	4,493,826	4,493,827	79,594	3prime(HHV)-4493827-3prime(Human)	71	0.00	.	.	gene	'OR52K1	protein_coding
11	4,493,831	4,493,832	79,594	3prime(HHV)-4493832-3prime(Human)	44	0.00	1	0.00	gene	'OR52K1	protein_coding
11	4,493,832	4,493,833	79,589	3prime(HHV)-4493833-3prime(Human)	1	0.00	.	.	gene	'OR52K1	protein_coding
11	6,173,146	6,173,147	3,928	5prime(Human)-6173147-5prime(HHV)	1	1.00	1	1.00	intron	'AC022762.1	lncRNA
11	130,665,098	130,665,099	136,387	3prime(HHV)-130665099-3prime(Human)	2	0.50	.	.	gene	'MIR8052	miRNA
11	130,665,098	130,665,099	136,397	3prime(HHV)-130665099-3prime(Human)	3	0.00	2	0.00	gene	'MIR8052	miRNA
11	130,665,099	130,665,100	136,397	3prime(HHV)-130665100-3prime(Human)	1	0.00	.	.	gene	'MIR8052	miRNA
11	130,665,101	130,665,102	136,397	3prime(HHV)-130665102-3prime(Human)	1	0.00	.	.	gene	'MIR8052	miRNA
11	130,665,102	130,665,103	136,397	3prime(HHV)-130665103-3prime(Human)	10	0.00	2	0.00	gene	'MIR8052	miRNA
11	130,665,106	130,665,107	136,397	3prime(HHV)-130665107-3prime(Human)	4	0.25	1	0.00	gene	'MIR8052	miRNA
15	101,981,031	101,981,032	60	3prime(Human)-101981032-5prime(HHV)	1	1.00	.	.	gene	'DDX11L9	lncRNA
16	25,760,104	25,760,105	139,198	3prime(HHV)-25760105-3prime(Human)	1	1.00	.	.	intron	'HS3ST4	protein_coding
16	75,334,205	75,334,206	144,566	3prime(HHV)-75334206-3prime(Human)	1	1.00	.	.	intron	'CFDP1	protein_coding
16	75,334,205	75,334,206	5,519	3prime(HHV)-75334206-3prime(Human)	1	1.00	.	.	intron	'CFDP1	protein_coding
17	30,919,726	30,919,727	3,928	3prime(Human)-30919727-5prime(HHV)	1	1.00	.	.	intron	'ADAP2	lncRNA
18	94,656	94,657	153	3prime(HHV)-94657-3prime(Human)	1	1.00	.	.	gene	'AP001005.2	processed_pseudogene
20	64,287,021	64,287,022	60	3prime(Human)-64287022-5prime(HHV)	1	1.00	1	1.00	intron	'PCMTD2	protein_coding

21	46,699,958	46,699,959	139,200	3prime(HHV)-46699959-5prime(Human)	1	1.00	1	1.00	gene	'RPL23AP4	processed_pseudogene
22	12,166,194	12,166,195	139,107	5prime(Human)-12166195-5prime(HHV)	4	1.00	1	1.00	gene	'AC138776.1	lncRNA
22	12,166,194	12,166,195	60	5prime(Human)-12166195-5prime(HHV)	11	1.00	3	1.00	gene	'AC138776.1	lncRNA
22	33,254,926	33,254,927	136,397	3prime(HHV)-33254927-3prime(Human)	2	1.00	1	1.00	intron	'LARGE1	protein_coding
22	44,626,521	44,626,522	139,107	3prime(Human)-44626522-5prime(HHV)	1	1.00	1	1.00	gene	'LINC00229	lncRNA
22	44,626,572	44,626,573	139,198	3prime(HHV)-44626573-5prime(Human)	1	1.00	.	.	gene	'LINC00229	lncRNA
22	50,808,161	50,808,162	153	3prime(HHV)-50808162-5prime(Human)	1	1.00	.	.	gene	'RPL23AP82	lncRNA
