# Supplementary material for: Integration of Viral Genome to Human Genomic DNA in Nails of Patients with Chronic Hepatitis B Virus Infection
Source: JMA J. 2023 Sep 29;6(4):426–36. doi: 10.31662/jmaj.2023-0082 (PMC10628332; doi:10.31662/jmaj.2023-0082)
Supplement: Supplementary Table 19 [file 2433-3298-6-4-426-s022.pdf]

Supplementary Table 19. Ig18205 HHV-7 integration breakpoints

Chrom	Start	End	Insert_Seq Breakpoint	Seqcode	# Junction Reads	Fraction of MQ0 Reads	# Junction Reads (Dedup)	Fraction of MQ0 Reads (Dedup)	Feature	Gene Name	Trasncript Biotype
1	11,440,134	11,440,135	139,200	3prime(HHV)-11440135-3prime(Human)	1	1.00	1	1.00	gene	'MTCYBP45	processed_pseudogene
1	248,946,106	248,946,107	139,200	3prime(HHV)-248946107-5prime(Human)	1	1.00	.	.	gene	'RPL23AP25	processed_pseudogene
2	43,403,799	43,403,800	139,205	3prime(HHV)-43403800-3prime(Human)	13	0.00	1	0.00	intron	'THADA	protein_coding
2	43,403,799	43,403,800	158	3prime(HHV)-43403800-3prime(Human)	8	0.00	1	0.00	intron	'THADA	protein_coding
2	87,397,261	87,397,262	139,117	5prime(Human)-87397262-5prime(HHV)	2	1.00	.	.	gene	'AC068279.1	processed_pseudogene
2	87,397,261	87,397,262	70	5prime(Human)-87397262-5prime(HHV)	1	1.00	.	.	gene	'AC068279.1	processed_pseudogene
2	87,397,686	87,397,687	139,117	5prime(Human)-87397687-5prime(HHV)	1	1.00	.	.	gene	'AC068279.1	processed_pseudogene
2	87,397,686	87,397,687	70	5prime(Human)-87397687-5prime(HHV)	3	1.00	1	1.00	gene	'AC068279.1	processed_pseudogene
2	87,397,980	87,397,981	101	5prime(Human)-87397981-5prime(HHV)	5	1.00	1	1.00	gene	'AC068279.1	processed_pseudogene
2	87,397,980	87,397,981	139,117	5prime(Human)-87397981-5prime(HHV)	3	1.00	1	1.00	gene	'AC068279.1	processed_pseudogene
2	87,397,980	87,397,981	139,148	5prime(Human)-87397981-5prime(HHV)	1	1.00	.	.	gene	'AC068279.1	processed_pseudogene
2	87,397,980	87,397,981	70	5prime(Human)-87397981-5prime(HHV)	5	1.00	.	.	gene	'AC068279.1	processed_pseudogene
2	87,398,442	87,398,443	101	5prime(Human)-87398443-5prime(HHV)	2	1.00	1	1.00	gene	'AC068279.1	processed_pseudogene
2	87,398,442	87,398,443	139,117	5prime(Human)-87398443-5prime(HHV)	6	1.00	.	.	gene	'AC068279.1	processed_pseudogene
2	87,398,442	87,398,443	70	5prime(Human)-87398443-5prime(HHV)	3	1.00	.	.	gene	'AC068279.1	processed_pseudogene
2	87,398,863	87,398,864	101	5prime(Human)-87398864-5prime(HHV)	1	1.00	.	.	gene	'AC068279.1	processed_pseudogene
2	87,398,863	87,398,864	139,117	5prime(Human)-87398864-5prime(HHV)	3	1.00	1	1.00	gene	'AC068279.1	processed_pseudogene
2	87,398,863	87,398,864	139,148	5prime(Human)-87398864-5prime(HHV)	4	1.00	.	.	gene	'AC068279.1	processed_pseudogene
2	87,398,863	87,398,864	70	5prime(Human)-87398864-5prime(HHV)	6	1.00	1	1.00	gene	'AC068279.1	processed_pseudogene
2	87,429,561	87,429,562	101	5prime(Human)-87429562-5prime(HHV)	2	1.00	1	1.00	gene	'LINC01943	lncRNA
2	87,429,561	87,429,562	139,117	5prime(Human)-87429562-5prime(HHV)	2	1.00	1	1.00	gene	'LINC01943	lncRNA
2	87,429,561	87,429,562	70	5prime(Human)-87429562-5prime(HHV)	1	1.00	.	.	gene	'LINC01943	lncRNA
2	87,429,934	87,429,935	101	5prime(Human)-87429935-5prime(HHV)	2	1.00	2	1.00	gene	'LINC01943	lncRNA
2	87,429,934	87,429,935	139,117	5prime(Human)-87429935-5prime(HHV)	6	1.00	2	1.00	gene	'LINC01943	lncRNA

2	87,429,934	87,429,935	70	5prime(Human)-87429935-5prime(HHV)	3	1.00	.	.	gene	'LINC01943	lncRNA
2	90,288,801	90,288,802	139,117	5prime(Human)-90288802-5prime(HHV)	1	1.00	.	.	gene	'AC233263.2	unprocessed_pseudogene
2	90,288,801	90,288,802	139,148	5prime(Human)-90288802-5prime(HHV)	3	1.00	2	1.00	gene	'AC233263.2	unprocessed_pseudogene
2	90,288,801	90,288,802	70	5prime(Human)-90288802-5prime(HHV)	2	1.00	.	.	gene	'AC233263.2	unprocessed_pseudogene
2	90,292,225	90,292,226	139,119	5prime(Human)-90292226-5prime(HHV)	1	1.00	.	.	gene	'AC233263.2	unprocessed_pseudogene
2	90,292,231	90,292,232	101	5prime(Human)-90292232-5prime(HHV)	5	1.00	.	.	gene	'AC233263.2	unprocessed_pseudogene
2	90,292,231	90,292,232	139,117	5prime(Human)-90292232-5prime(HHV)	6	1.00	1	1.00	gene	'AC233263.2	unprocessed_pseudogene
2	90,292,231	90,292,232	139,148	5prime(Human)-90292232-5prime(HHV)	2	1.00	1	1.00	gene	'AC233263.2	unprocessed_pseudogene
2	90,292,231	90,292,232	70	5prime(Human)-90292232-5prime(HHV)	3	1.00	1	1.00	gene	'AC233263.2	unprocessed_pseudogene
2	90,292,663	90,292,664	139,117	5prime(Human)-90292664-5prime(HHV)	1	1.00	1	1.00	gene	'AC233263.2	unprocessed_pseudogene
2	90,292,671	90,292,672	101	5prime(Human)-90292672-5prime(HHV)	1	1.00	1	1.00	gene	'AC233263.2	unprocessed_pseudogene
2	90,292,671	90,292,672	139,117	5prime(Human)-90292672-5prime(HHV)	4	1.00	1	1.00	gene	'AC233263.2	unprocessed_pseudogene
2	90,292,671	90,292,672	139,148	5prime(Human)-90292672-5prime(HHV)	2	1.00	1	1.00	gene	'AC233263.2	unprocessed_pseudogene
2	90,292,671	90,292,672	70	5prime(Human)-90292672-5prime(HHV)	5	1.00	2	1.00	gene	'AC233263.2	unprocessed_pseudogene
2	91,504,919	91,504,920	139,117	3prime(Human)-91504920-5prime(HHV)	1	1.00	1	1.00	gene	'AC233266.1	processed_pseudogene
2	91,504,919	91,504,920	70	3prime(Human)-91504920-5prime(HHV)	3	1.00	2	1.00	gene	'AC233266.1	processed_pseudogene
2	91,507,326	91,507,327	139,117	3prime(Human)-91507327-5prime(HHV)	2	1.00	1	1.00	gene	'AC233266.1	processed_pseudogene
2	91,507,326	91,507,327	70	3prime(Human)-91507327-5prime(HHV)	1	1.00	.	.	gene	'AC233266.1	processed_pseudogene
2	91,511,486	91,511,487	139,117	3prime(Human)-91511487-5prime(HHV)	1	1.00	1	1.00	gene	'AC233266.1	processed_pseudogene
2	91,513,691	91,513,692	139,117	3prime(Human)-91513692-5prime(HHV)	3	1.00	.	.	gene	'AC233266.1	processed_pseudogene
2	91,513,691	91,513,692	70	3prime(Human)-91513692-5prime(HHV)	2	1.00	1	1.00	gene	'AC233266.1	processed_pseudogene
2	91,514,739	91,514,740	139,117	3prime(Human)-91514740-5prime(HHV)	1	1.00	.	.	gene	'AC233266.1	processed_pseudogene
2	91,514,739	91,514,740	70	3prime(Human)-91514740-5prime(HHV)	1	1.00	.	.	gene	'AC233266.1	processed_pseudogene
2	91,519,656	91,519,657	139,117	3prime(Human)-91519657-5prime(HHV)	5	1.00	1	1.00	gene	'AC233266.1	processed_pseudogene
2	91,519,656	91,519,657	70	3prime(Human)-91519657-5prime(HHV)	2	1.00	.	.	gene	'AC233266.1	processed_pseudogene
2	91,522,476	91,522,477	139,117	3prime(Human)-91522477-5prime(HHV)	1	1.00	1	1.00	gene	'AC233266.1	processed_pseudogene
2	91,522,476	91,522,477	70	3prime(Human)-91522477-5prime(HHV)	1	1.00	.	.	gene	'AC233266.1	processed_pseudogene
2	91,523,585	91,523,586	139,117	3prime(Human)-91523586-5prime(HHV)	1	1.00	.	.	gene	'AC233266.1	processed_pseudogene

2	91,523,585	91,523,586	70	3prime(Human)-91523586-5prime(HHV)	4	1.00	1	1.00	gene	'AC233266.1	processed_pseudogene
2	91,524,694	91,524,695	139,117	3prime(Human)-91524695-5prime(HHV)	1	1.00	.	.	gene	'AC233266.1	processed_pseudogene
2	91,524,694	91,524,695	70	3prime(Human)-91524695-5prime(HHV)	1	1.00	.	.	gene	'AC233266.1	processed_pseudogene
2	97,220,755	97,220,756	3,928	3prime(Human)-97220756-5prime(HHV)	1	1.00	.	.	intron	'ANKRD36	protein_coding
2	109,024,098	109,024,099	120,093	3prime(Human)-109024099-5prime(HHV)	95	0.00	1	0.00	gene	'EDAR	protein_coding
2	109,024,104	109,024,105	120,093	3prime(Human)-109024105-5prime(HHV)	1	0.00	.	.	gene	'EDAR	protein_coding
2	109,024,108	109,024,109	120,093	3prime(Human)-109024109-5prime(HHV)	1	1.00	.	.	gene	'EDAR	protein_coding
3	10,223	10,224	153	3prime(HHV)-10224-3prime(Human)	1	1.00	.	.	gene	'LINC01986	lncRNA
3	10,228	10,229	139,200	3prime(HHV)-10229-3prime(Human)	1	1.00	.	.	gene	'LINC01986	lncRNA
3	10,228	10,229	153	3prime(HHV)-10229-3prime(Human)	1	1.00	.	.	gene	'LINC01986	lncRNA
3	10,229	10,230	139,205	3prime(HHV)-10230-3prime(Human)	2	1.00	1	1.00	gene	'LINC01986	lncRNA
3	10,512	10,513	139,205	3prime(HHV)-10513-3prime(Human)	2	1.00	1	1.00	gene	'LINC01986	lncRNA
3	10,574	10,575	139,200	3prime(HHV)-10575-3prime(Human)	1	1.00	1	1.00	gene	'LINC01986	lncRNA
4	705,157	705,158	153	3prime(HHV)-705158-3prime(Human)	1	1.00	.	.	gene	'PCGF3	protein_coding
4	190,122,606	190,122,607	139,200	3prime(HHV)-190122607-5prime(Human)	36	1.00	1	1.00	gene	'DUX4L2	unprocessed_pseudogene
4	190,122,606	190,122,607	139,205	3prime(HHV)-190122607-5prime(Human)	1	1.00	1	1.00	gene	'DUX4L2	unprocessed_pseudogene
4	190,122,606	190,122,607	153	3prime(HHV)-190122607-5prime(Human)	37	1.00	4	1.00	gene	'DUX4L2	unprocessed_pseudogene
4	190,122,789	190,122,790	139,200	3prime(HHV)-190122790-5prime(Human)	40	1.00	4	1.00	gene	'DUX4L2	unprocessed_pseudogene
4	190,122,789	190,122,790	139,205	3prime(HHV)-190122790-5prime(Human)	1	1.00	.	.	gene	'DUX4L2	unprocessed_pseudogene
4	190,122,789	190,122,790	153	3prime(HHV)-190122790-5prime(Human)	35	1.00	2	1.00	gene	'DUX4L2	unprocessed_pseudogene
5	83,516,151	83,516,152	62	5prime(Human)-83516152-5prime(HHV)	1	1.00	1	1.00	intron	'VCAN	protein_coding
6	1,114,753	1,114,754	153	3prime(HHV)-1114754-3prime(Human)	1	1.00	.	.	gene	'AL033381.3	lncRNA
7	16,421	16,422	139,195	3prime(HHV)-16422-3prime(Human)	1	1.00	.	.	intron	'AC215522.3	lncRNA
9	138,129,059	138,129,060	62	3prime(Human)-138129060-5prime(HHV)	1	1.00	1	1.00	gene	'AL591424.2	unprocessed_pseudogene
9	138,129,060	138,129,061	139,107	3prime(Human)-138129061-5prime(HHV)	1	1.00	1	1.00	gene	'AL591424.2	unprocessed_pseudogene
9	138,159,868	138,159,869	139,200	3prime(HHV)-138159869-5prime(Human)	1	1.00	1	1.00	intron	'TUBBP5	lncRNA
9	138,159,868	138,159,869	139,216	3prime(HHV)-138159869-5prime(Human)	1	1.00	.	.	intron	'TUBBP5	lncRNA
10	9,996	9,997	5,429	3prime(HHV)-9997-3prime(Human)	1	1.00	1	1.00	gene	'AC215217.1	lncRNA

10	8,468,548	8,468,549	139,119	5prime(Human)-8468549-5prime(HHV)	1	1.00	1	1.00	gene	'AC025946.2	processed_pseudogene
10	109,841,704	109,841,705	142,350	3prime(HHV)-109841705-5prime(Human)	1	1.00	.	.	gene	'XPNPEP1	protein_coding
10	109,841,707	109,841,708	142,350	3prime(HHV)-109841708-5prime(Human)	56	1.00	1	1.00	gene	'XPNPEP1	protein_coding
10	109,841,707	109,841,708	3,303	3prime(HHV)-109841708-5prime(Human)	74	1.00	1	1.00	gene	'XPNPEP1	protein_coding
11	3,920,778	3,920,779	142,975	3prime(Human)-3920779-5prime(HHV)	1	1.00	.	.	intron	'STIM1	protein_coding
11	128,930,684	128,930,685	5,640	3prime(Human)-128930685-5prime(HHV)	1	1.00	1	1.00	gene	'TP53AIP1	protein_coding
11	135,076,619	135,076,620	139,200	3prime(HHV)-135076620-5prime(Human)	1	1.00	1	1.00	gene	'LINC02684	lncRNA
11	135,076,619	135,076,620	153	3prime(HHV)-135076620-5prime(Human)	1	1.00	1	1.00	gene	'LINC02684	lncRNA
12	10,547	10,548	139,205	3prime(HHV)-10548-3prime(Human)	1	0.00	.	.	gene	'DDX11L8	unprocessed_pseudogene
12	108,094	108,095	139,200	3prime(HHV)-108095-3prime(Human)	1	1.00	.	.	intron	'IQSEC3	protein_coding
15	40,870,396	40,870,397	5,742	3prime(HHV)-40870397-5prime(Human)	2	1.00	2	1.00	gene	'RHOV	protein_coding
15	101,981,026	101,981,027	139,107	3prime(Human)-101981027-5prime(HHV)	1	1.00	1	1.00	gene	'DDX11L9	lncRNA
15	101,981,026	101,981,027	139,109	3prime(Human)-101981027-5prime(HHV)	8	0.00	1	0.00	gene	'DDX11L9	lncRNA
15	101,981,026	101,981,027	62	3prime(Human)-101981027-5prime(HHV)	7	0.00	1	0.00	gene	'DDX11L9	lncRNA
16	25,760,100	25,760,101	139,200	3prime(HHV)-25760101-3prime(Human)	1	1.00	.	.	intron	'HS3ST4	protein_coding
16	25,760,101	25,760,102	139,200	3prime(HHV)-25760102-3prime(Human)	1	1.00	.	.	intron	'HS3ST4	protein_coding
16	25,760,101	25,760,102	153	3prime(HHV)-25760102-3prime(Human)	1	1.00	.	.	intron	'HS3ST4	protein_coding
16	25,760,104	25,760,105	5,643	3prime(HHV)-25760105-3prime(Human)	1	0.00	1	0.00	intron	'HS3ST4	protein_coding
20	5,729,894	5,729,895	120,093	3prime(Human)-5729895-5prime(HHV)	59	0.00	1	0.00	gene	'SHLD1	protein_coding
20	25,781,706	25,781,707	69,688	5prime(Human)-25781707-5prime(HHV)	63	1.00	1	1.00	intron	'FAM182B	lncRNA
20	26,074,364	26,074,365	69,688	3prime(Human)-26074365-5prime(HHV)	60	1.00	.	.	intron	'FAM182A	lncRNA
20	32,411,064	32,411,065	3,928	5prime(Human)-32411065-5prime(HHV)	1	1.00	.	.	intron	'ASXL1	protein_coding
21	46,699,958	46,699,959	139,200	3prime(HHV)-46699959-5prime(Human)	2	1.00	1	1.00	gene	'RPL23AP4	processed_pseudogene
22	44,626,573	44,626,574	153	3prime(HHV)-44626574-5prime(Human)	1	1.00	1	1.00	gene	'LINC00229	lncRNA
22	50,808,132	50,808,133	153	3prime(HHV)-50808133-5prime(Human)	1	1.00	.	.	gene	'RPL23AP82	lncRNA
22	50,808,364	50,808,365	139,107	3prime(Human)-50808365-5prime(HHV)	2	1.00	.	.	gene	'RPL23AP82	lncRNA
